# *Cis*-difluoromethyl hetarylative dearomatization by a radical docking-migration cascade

**DOI:** 10.1039/d5sc07904g

**Published:** 2025-11-10

**Authors:** Jie Wang, Hao Kang, Shan Yang, Zhu Cao, Xiangyang Chen, Chen Zhu

**Affiliations:** a Frontiers Science Center for Transformative Molecules, School of Chemistry and Chemical Engineering, State Key Laboratory of Synergistic Chem-Bio Synthesis, Shanghai Key Laboratory for Molecular Engineering of Chiral Drugs, Shanghai Jiao Tong University 800 Dongchuan Road Shanghai 200240 China chzhu@sjtu.edu.cn chenxiangyang@sjtu.edu.cn; b Key Laboratory of Organic Synthesis of Jiangsu Province, College of Chemistry, Chemical Engineering and Materials Science, Soochow University 199 Ren-Ai Road Suzhou Jiangsu 215123 China

## Abstract

Despite the significant advances in dearomatization reactions, the challenge of achieving uncyclized dearomatization to produce thermodynamically unstable 1,2-*cis*-products remains unresolved. Here, we present a novel approach for uncyclized *cis*-selective dearomatization reaction *via* a radical docking-migration cascade. The reaction proceeds under mild photochemical conditions, simultaneously incorporating a CF_2_H and a hetaryl group into indoline backbones. A wide range of indoles with diverse functional groups are compatible with the reaction. Furthermore, this method is also suitable for the dearomatization of benzothiophenes, furans, thiophenes and a few polycyclic aromatic hydrocarbons. This protocol features excellent selectivities, broad product diversity, and does not require photosensitizers. DFT calculations rationalize the observed regioselectivities for various heteroarenes and *cis*-stereoselectivities.

## Abstract 2

We report the first example of an uncyclized *cis*-selective dearomatization reaction using a radical docking-migration strategy. The reaction proceeds readily under photochemical conditions without the use of extra photosensitizers.

## Graphical abstract

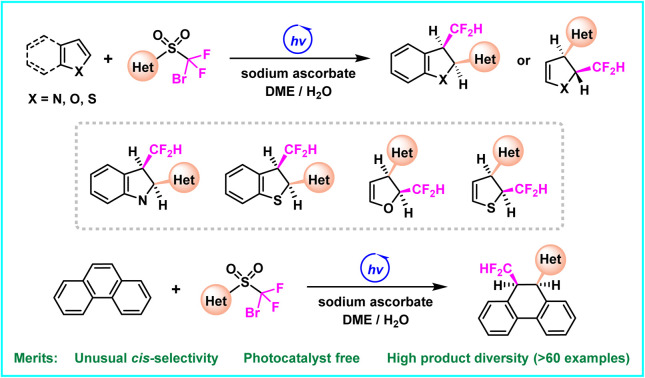

## Introduction

**Fig. 1:**
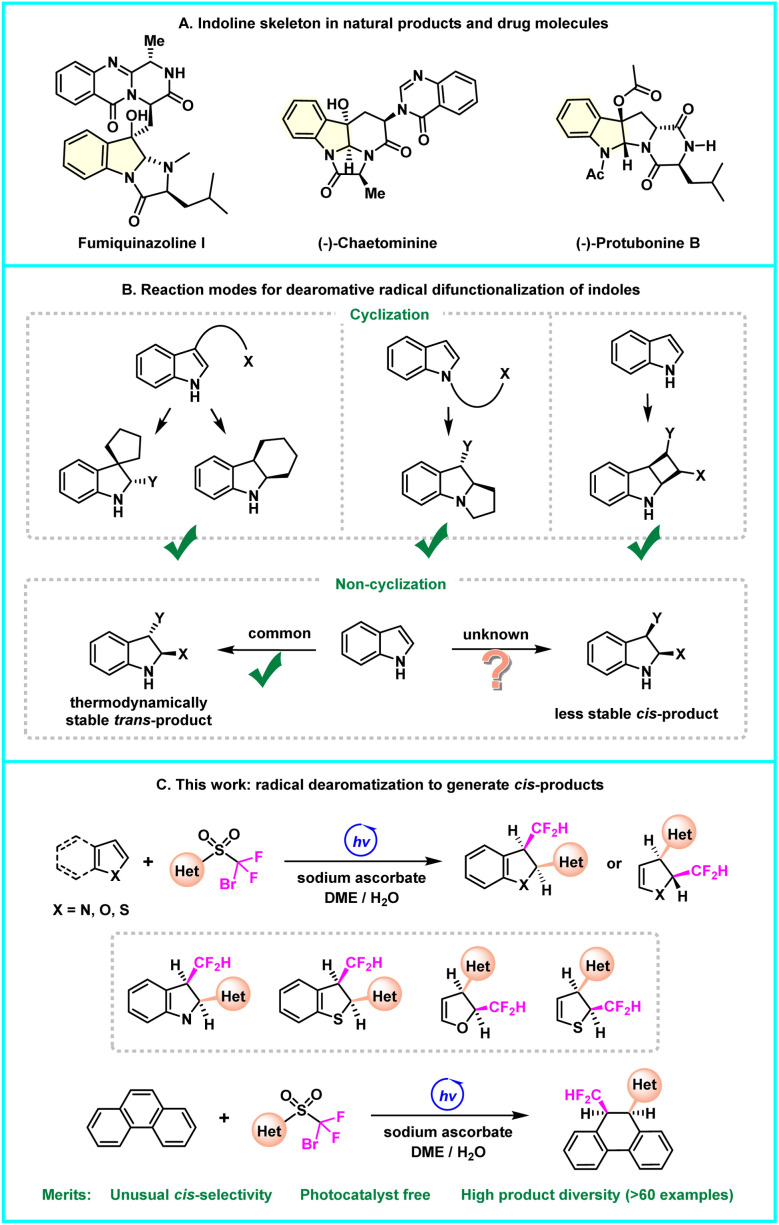
(A) Indoline skeleton in natural products and drug molecules. (B) Reaction modes for dearomative radical difunctionalization of indoles. (C) Radical dearomatization to generate *cis*-products.

Heteroarenes are predominant moieties in numerous biologically active compounds, but their flat structural features often cause the problem of poor solubility and metabolic instability,^1^ thus increasing the demand for an “escape from flatland” imperative for medicinal chemists.^2^ The dearomatization reaction represents a straightforward approach to convert planar heteroarenes into three-dimensional heterocyclic frameworks, providing more space for new drug discovery and structural modification of drug molecules.^3^ In this scenario, the last few decades have witnessed great progress in the field of radical-mediated dearomatization of heteroarenes,^4^ where indole serves as one of the most privileged precursors, giving rise to valuable indoline scaffolds that are abundant in natural products and drugs (Fig. 1A). Among these efforts, the indoles with pendant aliphatic alkene or halide capable of generating an alkyl radical are frequently utilized, engaging in kinetically favoured intramolecular cyclization (Fig. 1B).^5^ Moreover, intermolecular approaches proceed mainly *via* thermodynamically preferred *trans*-difunctionalization^6^ or [2 + 2] photocycloaddition.^7^ Nevertheless, the uncyclized dearomatization of indoles leading to unusual and thermodynamically less stable *cis*-products remains unmet.

The radical docking-migration cascade is proving to be a robust strategy for the structural modification of organic molecules.^8^ Nevertheless, its versatility has only been demonstrated in the conversion of alkenes and alkynes.^9^ We conceive to explore the feasibility of using it to address the elusive dearomative *cis*-difunctionalization. Here we disclose the proof-of-principle studies. The uncyclized radical dearomatization of indoles, leading to *cis*-disubstituted indolines, is accomplished for the first time (Fig. 1C). The reaction is facilitated by visible-light irradiation, albeit in the absence of a photocatalyst. Besides indoles, this protocol can also be adapted to other electron-rich heteroarenes, including benzothiophenes, furans and thiophenes. Notably, it can be extended to the conversion of certain polycyclic aromatic hydrocarbons (PAHs), which are typically recognized as challenging substrates.

## Results and discussion

Initially, the reaction of *N*-benzyl indole (1a) with a bifunctional sulfone reagent (2a) designed for the radical docking-migration cascade was carried out to obtain the optimal reaction conditions. Extensive investigations (see the SI for details) indicated that the reaction gave rise to a good yield of the *cis*-difluoromethyl hetarylated product (3a) using sodium ascorbate as an additive and DME/H_2_O as a co-solvent under blue light irradiation (Table 1, entry 1). It is noteworthy that this photochemical reaction proceeded efficiently without the need for a photocatalyst. Replacing sodium ascorbate with ascorbic acid resulted in a lower yield (Table 1, entries 2–6). Control experiments further confirmed that both the additive and light were essential for the conversion (Table 1, entries 7 and 8). The addition of water serves to dissolve the sodium ascorbate and to maintain high light permeability. Consequently, the reaction without or with insufficient water resulted in poor yields (Table 1, entries 9 and 10). Conversely, the addition of excess water hindered the conversion (Table 1, entry 11).

**Table 1:** Reaction parameter survey^a^

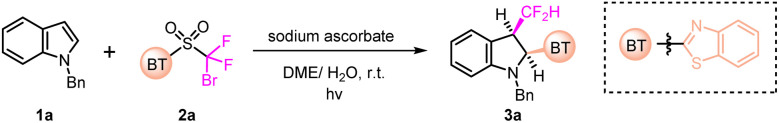		
Entry	Variation from the standard conditions	Yield^b^ (%)
1	None	72
2	Ascorbic acid as an additive	40
3	TTMSS as an additive	15
4	HCO_2_Cs as an additive	<10
5	Hantzsch ester as an additive	<10
6	*tert*-Dodecylthiol as an additive	Trace
7	No sodium ascorbate	Trace
8	No light	0
9	No H_2_O	Trace
10	0.2 mL H_2_O	35
11	1.0 mL H_2_O	41

a 1a (0.2 mmol), 2a (0.1 mmol), and sodium ascorbate (0.25 mmol) in DME/H_2_O (v/v 4/0.5 mL), irradiated by 456 nm Kessil light at r.t. under N_2_ for 6 h.

b Yields of isolated products. TTMSS = tris(trimethylsilyl)silane.

**Fig. 2:**
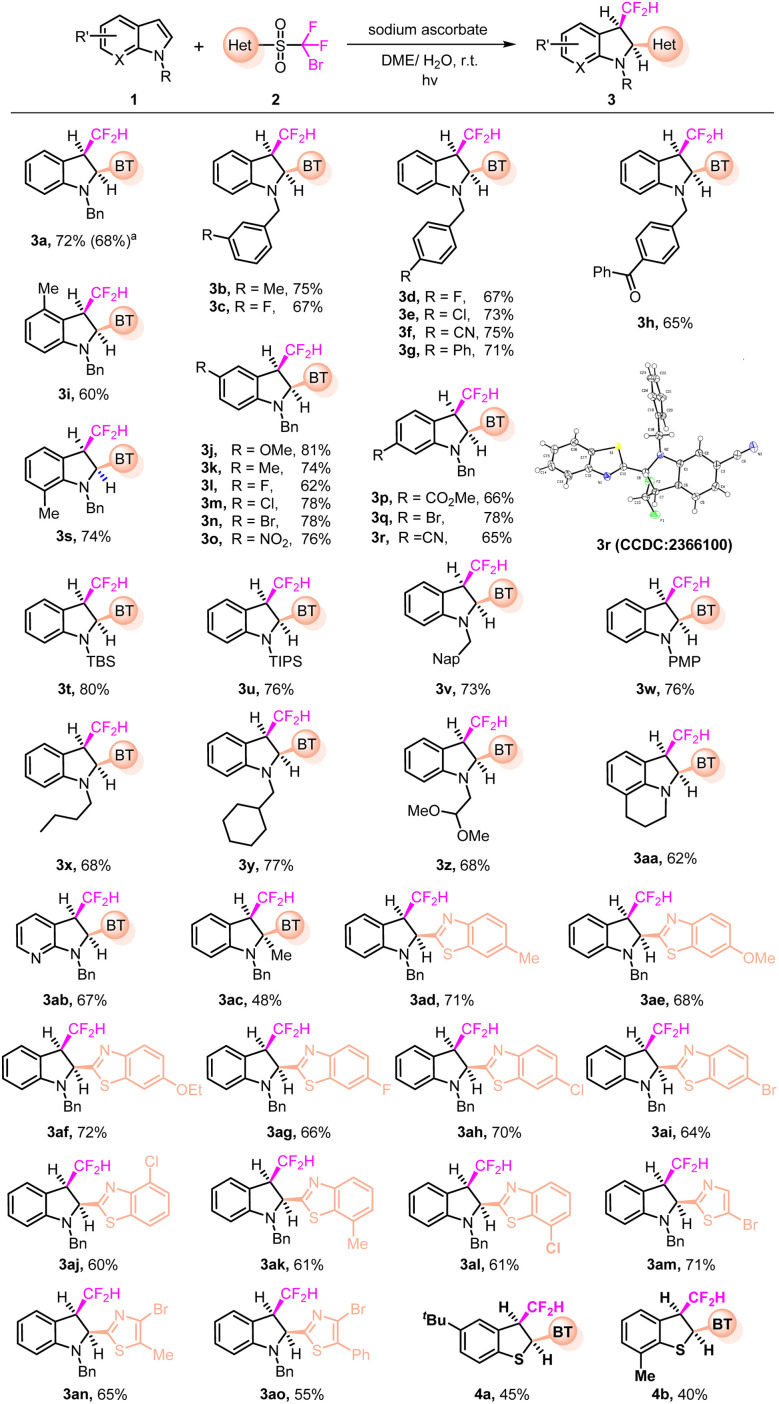
Dearomatization of indoles and benzothiophenes. Reaction conditions: 1 (0.4 mmol), 2 (0.2 mmol), sodium ascorbate (0.5 mmol) in DME/H_2_O (v/v 8/1 mL), irradiated by 456 nm Kessil light at r.t. under N_2_ for 6–10 h. Yields of isolated products are given. ^*a*^Scaled-up preparation with 1a (4 mmol) and 2a (2 mmol).

With the optimized reaction conditions in hand, we first investigated the dearomatization of various indoles (Fig. 2). The reaction adapted to a broad spectrum of indole derivatives, giving the corresponding *cis*-products (3a–3ao) in generally good yields. The reaction could be easily scaled up, giving rise to a synthetically useful yield of 3a. A variety of reactive substituents on the substrate such as bromide, cyano, nitro, carbonyl, and ester were well tolerated. In particular, the position of the strong electron-withdrawing nitro group did not impact the reaction outcome (3o). The reaction of indoles with the substitution at the 4-, 5-, 6- or 7-position readily afforded the corresponding products (3i–3s), regardless of distinct steric environments. The *cis*-relative configuration for the product was unambiguously assigned by the single-crystal structure of 3r (see the SI for details). The benzyl protecting group could be changed to silyl (*e.g.*, TBS and TIPS), aromatic (*e.g.*, PMP) or aliphatic groups (3t–3y). Notably, the susceptible acetal group also remained intact in the transformation (3z). The structure of 3aa was intriguing, presenting a polycyclic product with a useful yield. Azaindole was also a suitable substrate for the reaction (3ab). The reaction of the 2-substituted indole proceeded in moderate yield (3ac) due to the increased steric hindrance around the reaction site. The bifunctional sulfone reagents could be varied. Installing an extra functional group on the benzothiazolyl moiety did not impede the transformation, regardless of the electronic properties of the substituents (3ad–3al). When replacing benzothiazolyl with thiazolyl, the corresponding products were also obtained in synthetically useful yields (3am–3ao). Under standard conditions, benzothiophenes were also readily converted to the corresponding *cis*-products (4a and 4b) in moderate yields, with the same regio- and stereoselectivities.

**Fig. 3:**
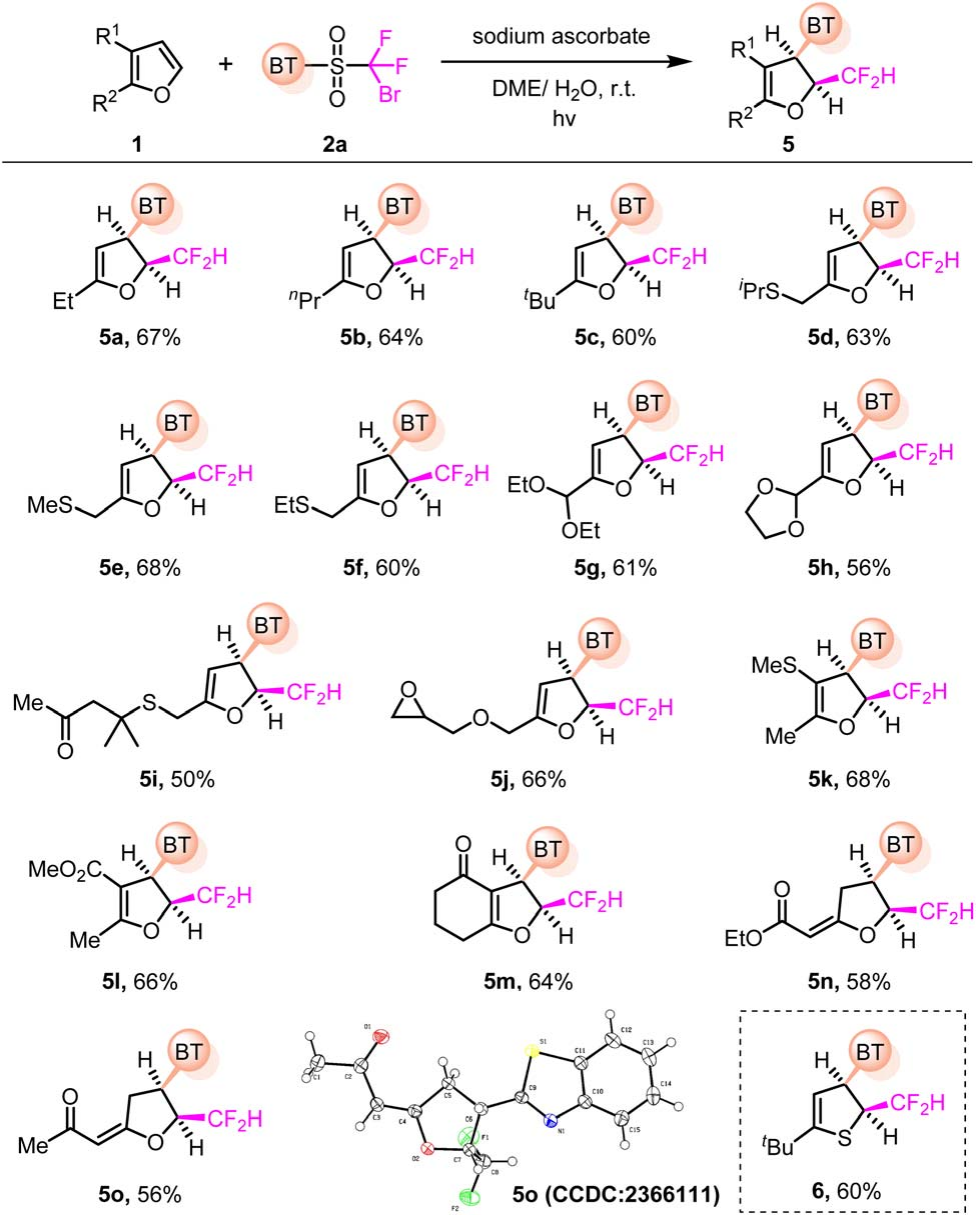
Dearomatization of furans and thiophenes. Reaction conditions: 1 (0.4 mmol), 2a (0.2 mmol), sodium ascorbate (0.5 mmol) in DME/H_2_O (v/v 8/1 mL), irradiated by 456 nm Kessil light at r.t. under N_2_ for 6–10 h. Yields of isolated products are given.

This protocol was further extended to the dearomatization of furans and thiophenes (Fig. 3), which mainly underwent intramolecular 2,5-cyclizations in previous reports.^10^ A set of C5-substituted furans was apt to give the 2,3-*cis*-difunctionalized dihydrofuran products (5a–5h), with sensitive groups such as acetal and epoxide remaining intact under the mild photochemical conditions (5g, 5h, and 5j). Moreover, 4,5-disubstituted furans were also amenable to afford the target products in useful yields (5k–5m). The reaction with furyl-substituted ethyl acetate or acetone proceeded through the anticipated 2,3-difunctionalization of furan followed by alkene isomerization, leading to the tetrahydrofuran products in moderate yields (5n and 5o). The *cis*-relative configuration of 5o was verified by single-crystal X-ray diffraction analysis (see the SI for details). Furthermore, thiophene also proved to be a suitable substrate for the 2,3-*cis*-difunctionalization (6). Of note, the regioselectivity obtained is reversed compared to that of indoles and benzothiophenes, presumably dictated by the electronic properties of the substrates.

**Fig. 4:**
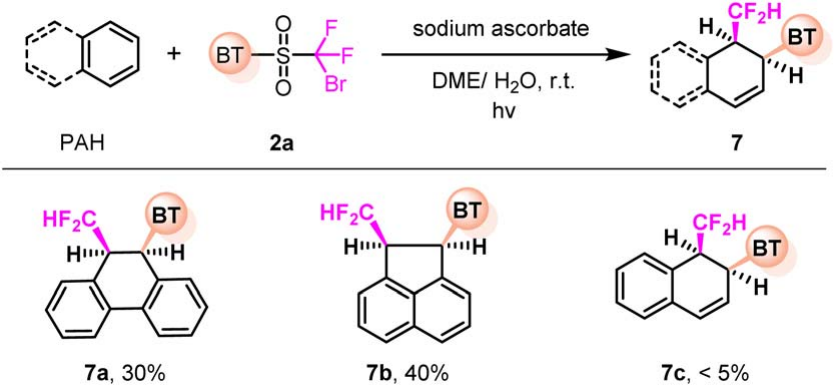
Dearomatization of PAHs. Reaction conditions: arene (0.4 mmol), 2a (0.2 mmol), sodium ascorbate (0.5 mmol) in DME/H_2_O (v/v 8/1 mL), irradiated by 456 nm Kessil light at r.t. under N_2_ for 6–10 h. Yields of isolated products are given.

Simple arenes are challenging substrates, which have been rarely harnessed for dearomatization,^11^ due to their low reactivities and the poor selectivity in the presence of multiple reaction sites. This method offers the potential for the dearomative 1,2-difunctionalization of PAHs (Fig. 4). For instance, the dearomatization of phenanthrene and acenaphthylene readily took place, yielding the corresponding *cis*-products (7a and 7b). Unfortunately, naphthalene could not be efficiently converted to the dearomative product (7c).

**Fig. 5:**
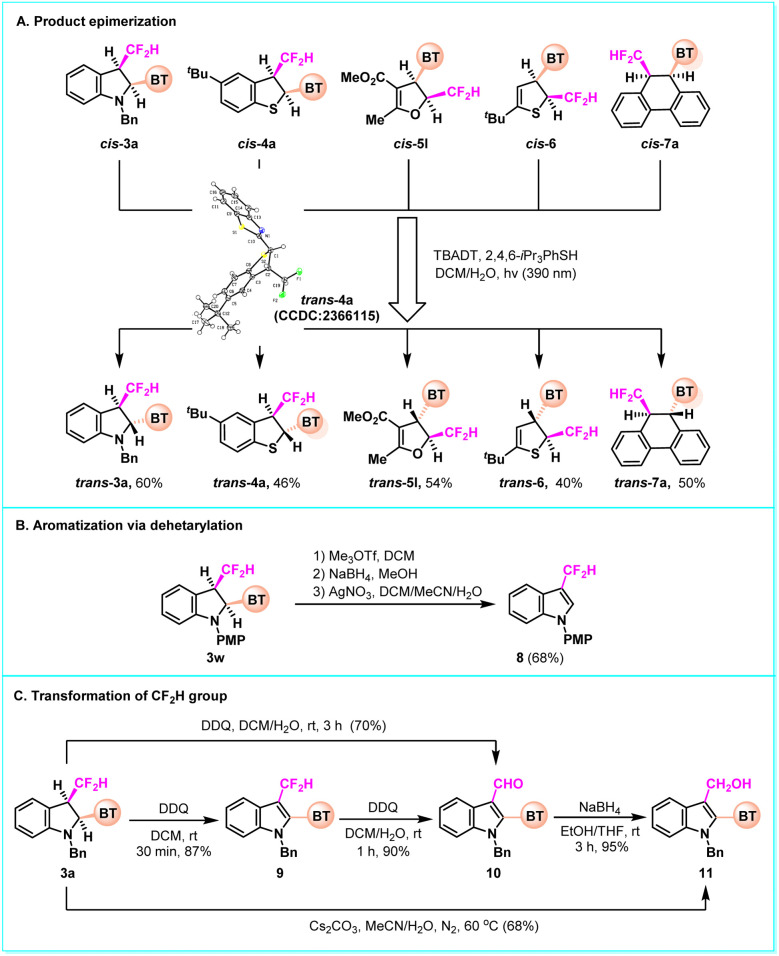
Product transformations. (A) Product epimerization. (B) Aromatization *via* dehetarylation. (C) Transformation of CF_2_H group.

When treating the *cis*-products (3a, 4a, 5l, 6 and 7a) with photocatalytic HAT conditions,^12^ these compounds were epimerized to the corresponding *trans*-products (Fig. 5A). The relative configuration of *trans*-4a was confirmed by the single-crystal X-ray diffraction analysis (see the SI for details). This result further supports that the *cis*-products obtained *via* the docking-migration cascade are thermodynamically unstable. In 3w, the benzothiazolyl group could be readily removed, facilitating rearomatization and the formation of 3-difluoromethyl indole (8) (Fig. 5B). On treatment with DDQ, 3a could be oxidized first to the disubstituted indole 9 and then further to the 3-formylindole 10 by converting CF_2_H into a formyl group. Alternatively, the compound 10 could be directly obtained from 3a in one step with an extended reaction time. The reduction of 10 afforded the indole-3-carbinol 11; or the product 11 was obtained directly from 3a under heating conditions using Cs_2_CO_3_ as the base (Fig. 5C).

**Fig. 6:**
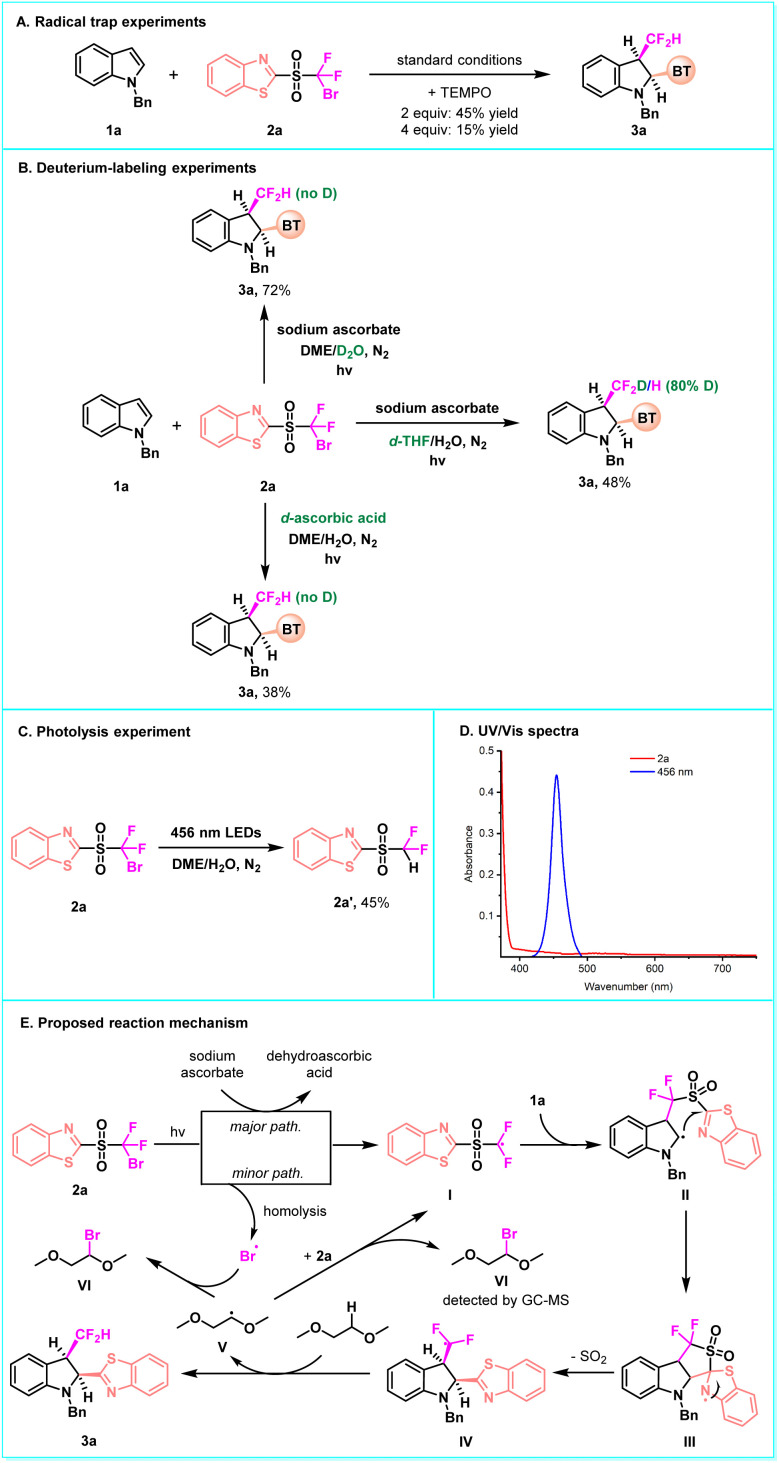
Mechanistic studies and proposed mechanism.

The addition of the radical scavenger TEMPO to the reaction effectively inhibited the conversion (Fig. 6A), potentially indicating the existence of radical pathways. Then, a series of deuterium-labeling experiments was performed. When using D_2_O instead of H_2_O as cosolvent or deuterated ascorbic acid instead of sodium ascorbate as a reducing agent, neither of the products was deuterated. However, using deuterated THF instead of DME as the organic solvent, the deuterated product was obtained. This indicates that the hydrogen atom in the CF_2_H group comes from the organic solvent (Fig. 6B). The UV-Vis absorption experiments displayed that no EDA complex is formed between the substrates and sodium ascorbate. Moreover, irradiation of 2a under blue LEDs in the absence of a photocatalyst produced 2a′ in 45% yield (Fig. 6C), suggesting that the reaction is initiated by light-promoted homolytic cleavage of the C–Br bond of the bifunctional reagents to generate an active difluoroalkyl radical intermediate. 2a exhibited weak absorption in the range of 400 to 450 nm in the blue region (Fig. 6D), which might trigger the photolysis of 2a to initiate the transformation. In addition, the quantum yield test (*Φ* = 4.2) demonstrated that the radical chain process is involved in the reaction (see the SI for details). Accordingly, a possible reaction mechanism is proposed in Fig. 6E. Initially, the single-electron transfer (SET) between 2a and sodium ascorbate forms an electrophilic difluoroalkyl radical species I, which rapidly adds to the C=C bond of heteroarene, generating radical intermediate II. Sodium ascorbate acts as a reducing agent and transforms itself into dehydroascorbic acid. Alternatively, the C–Br bond of 2a can undergo homolytic cleavage under blue light irradiation, which offers a minor pathway to produce radical I. Subsequently, intramolecular heteroaryl migration *via* a kinetically favorable five-membered cyclic transition state followed by SO_2_ extrusion takes place, leading to the difluoromethyl radical IV. This radical then abstracts the H-atom from DME to furnish the final product 3a and meanwhile generates a new alkyl radical V. This radical either abstracts the Br-atom from 2a to regenerate intermediate I, thus perpetuating the radical chain, or reacts with the *in situ* generated Br-atom. The resulting alkyl bromide can be detected by GC-MS analysis.

**Fig. 7:**
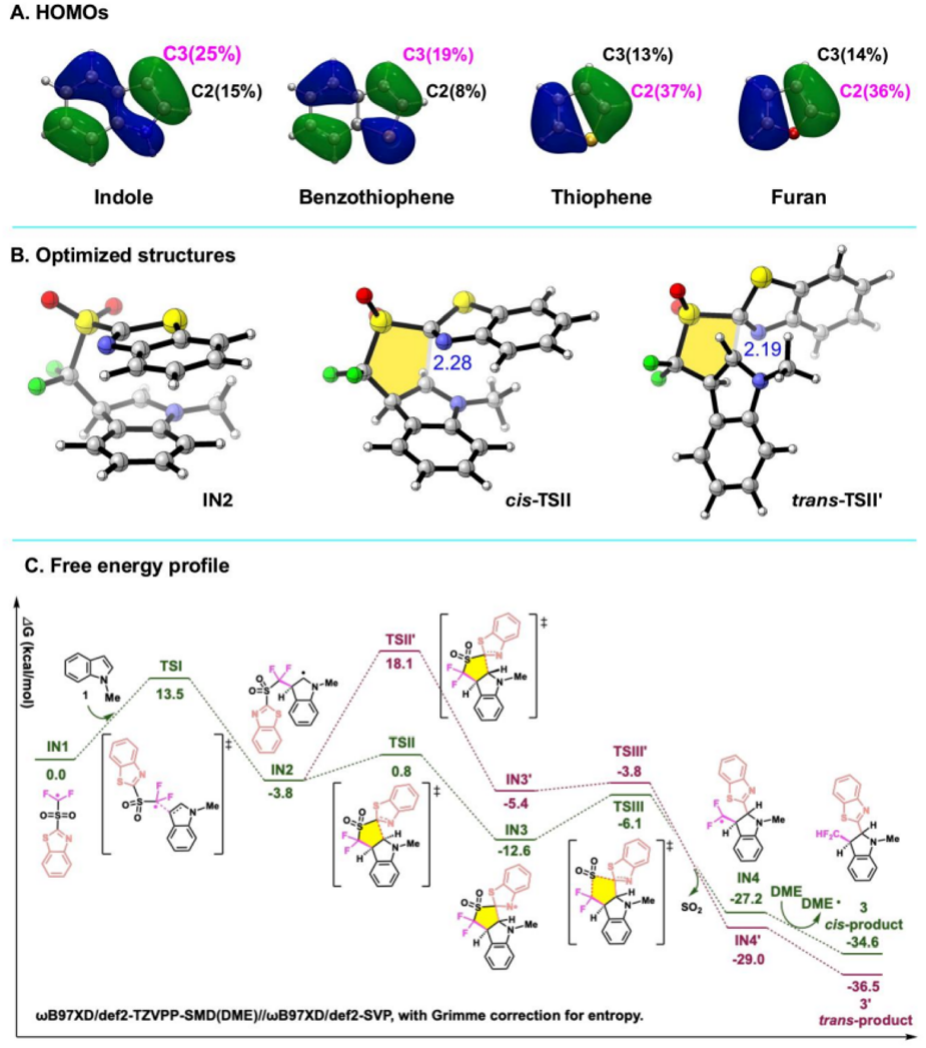
DFT calculations. (A) HOMOs. (B) Optimized structures. (C) Free energy profile. Distances are in Å. All energies are given in kcal mol^−1^.

Detailed mechanisms were further investigated by density functional theory (DFT) calculations at the ωB97XD/def2-TZVPP-SMD(DME)//ωB97XD/def2-SVP theory level. As shown in Fig. 7A, the HOMO coefficient at C3 is significantly larger than at C2 in indole and benzothiophene, whereas the opposite trend is observed in thiophene and furan. This difference in electronic distribution ultimately governs the regioselectivity for the reactions, which is consistent with the experimentally observed products. The free energy profile of the reaction and the optimized transition state structures are presented in Fig. 7B and C. The addition of radical intermediate IN1 to the C=C bond of heteroarenes 1*via*TSI is 13.5 kcal mol^−1^ high in energy, generating radical intermediate IN2. Subsequent intramolecular heteroaryl migration proceeds through five-membered cyclic transition states, TSII and TSII′, leading to the formation of *cis* and *trans* products, respectively. TSII is 17.3 kcal mol^−1^ lower in energy than TSII′, attributed to reduced ring twisting (highlighted in yellow) and smaller structural distortion (Fig. S229). Finally, SO_2_ is released through TSIII with an energy of −6.1 kcal mol^−1^, followed by hydrogen abstraction from the solvent DME, leading to the final *cis*-product *via* kinetic pathways.

## Conclusions

We have described the first example of an uncyclized *cis*-selective dearomatization reaction using a radical docking-migration strategy. The reaction proceeds readily under photochemical conditions without the use of extra photosensitizers. The protocol is applicable to a vast array of electron-rich heteroarenes, such as indoles, benzothiophenes, furans and thiophenes, and certain PAHs, giving the 1,2-*cis*-difluoromethyl hetarylative products. In particular, the *cis*-products obtained can be conveniently epimerized to the *trans*-products. The protocol also features mild conditions, broad functional group compatibility, and high product diversity. Mechanistic studies reveal the involvement of a radical chain process in the transformation. DFT calculations provide an explanation for the regioselectivities observed for various heteroarenes and the consistent *cis*-stereoselectivities.

## Author contributions

CZ conceived the idea and designed the experiments. JW performed most of the laboratory experiments. HK and XC performed DFT calculations. SY helped with the analysis of the data. SY and ZC prepared some of the starting materials. CZ supervised the project. The manuscript was written through the contributions of all authors. All authors have given approval to the final version of the manuscript.

## Conflicts of interest

There are no conflicts to declare.

## Supplementary Material

SC-017-D5SC07904G-s001

SC-017-D5SC07904G-s002

## Data Availability

CCDC 2366100 (3r), 2366111 (5o) and 2366115 (*trans*-4a) contain the supplementary crystallographic data for this paper.^13*a*–*c*^ The data supporting this article have been included as part of the supplementary information (SI). Supplementary information: experimental and computational details, materials, methods, NMR spectra, and characterization data of products. See DOI: https://doi.org/10.1039/d5sc07904g.

## References

[cit1] Ritchie T. J., Macdonald S. J. F. (2009). Drug Discov. Today.

[cit2] Lovering F., Bikker J., Humblet C. (2009). J. Med. Chem..

[cit3] Zhuo C.-X., Zheng C., You S.-L. (2014). Acc. Chem. Res..

[cit4] Takayama H., Misawa K., Okada N., Ishikawa H., Kitajima M., Hatori Y., Murayama T., Wongseripipatana S., Tashima K., Matsumoto K., Horie S. (2006). Org. Lett..

[cit5] Ye J.-H., Zhu L., Yan S.-S., Miao M., Zhang W.-J., Li J., Lan Y., Yu D.-G. (2017). ACS Catal..

[cit6] Wu J., Dou Y., Guillot R., Kouklovsky C., Vincent G. (2019). J. Am. Chem. Soc..

[cit7] Hu N., Jung H., Zheng Y., Lee J., Zhang L., Ullah Z., Xie X., Harms K., Baik M.-H., Meggers E. (2018). Angew. Chem., Int. Ed..

[cit8] Chen Z.-M., Zhang X.-M., Tu Y.-Q. (2015). Chem. Soc. Rev..

[cit9] Yu J., Wu X., Zhu C. (2018). Angew. Chem., Int. Ed..

[cit10] Adams K., Ball A. K., Birkett J., Brown L., Chappell B., Gill D. M., Lo P. K. T., Patmore N. J., Rice C. R., Ryan J., Raubo P., Sweeney J. B. (2017). Nat. Chem..

[cit11] Wiesenfeldt M. P., Nairoukh Z., Dalton T., Glorius F. (2019). Angew. Chem., Int. Ed..

[cit12] Waele V. D., Poizat O., Fagnoni M., Bagno A., Ravelli D. (2016). ACS Catal..

[cit13] (a) CCDC 2366100: Experimental Crystal Structure Determination, 2025, 10.5517/ccdc.csd.cc2kf3ty

