# Supplementary material for: Cis-difluoromethyl hetarylative dearomatization by a radical docking-migration cascade
Source: Chem Sci. 2025 Nov 10;17(1):318–24. doi: 10.1039/d5sc07904g (PMC12621134; doi:10.1039/d5sc07904g)
Supplement: SC-017-D5SC07904G-s001 [file SC-017-D5SC07904G-s001.pdf]

Supplementary Information (SI) for Chemical Science.
This journal is © The Royal Society of Chemistry 2025

Supporting Information

Cis-Difluoromethyl Hetarylative Dearomatization by Radical Docking-Migration Cascade

Jie Wang,[1,2] Hao Kang,[1] Shan Yang,[1] Zhu Cao,[1] Xiangyang Chen,*[,1] Chen Zhu*[,1,2]

[1] Frontiers Science Center for Transformative Molecules, School of Chemistry and Chemical Engineering, State Key Laboratory of Synergistic Chem-Bio Synthesis, and Shanghai Key Laboratory for Molecular Engineering of Chiral Drugs, Shanghai Jiao Tong University, 800 Dongchuan Road, Shanghai 200240, China

[2] Key Laboratory of Organic Synthesis of Jiangsu Province, College of Chemistry, Chemical Engineering and Materials Science, Soochow University, 199 Ren-Ai Road, Suzhou, Jiangsu 215123, China

Table of Contents

1. General experimental details 2
2. Reaction condition optimization 2
3. Preparation of starting materials 10
4. General procedures for dearomatization of (hetero)arenes 13
5. Product transformations 32
6. Single-crystal X-ray diffraction analysis 38
7. Mechanistic studies 41
8. NMR spectra 45
9. DFT calculations 157
Reference 174

1. General experimental details

All reactions were maintained under a nitrogen atmosphere unless otherwise stated. Commercially available reagents were used without further purification. DMF was distilled from CaH_2 under reduced pressure, and DCM was distilled from CaH_2, and THF was distilled from sodium. Infrared (FT-IR) spectra were recorded on a BRUKER VERTEX 70, ν_{max} in cm^{-1}.^{1}H-NMR spectra were recorded on a BRUKER AVANCE III HD (400 MHz) spectrometer. Chemical shifts are reported in ppm from tetramethylsilane with the solvent resonance as internal standard ($CDCl_3$: δ 7.26, (CD_3CN: δ 1.94). Data are reported as follows: chemical shift, multiplicity (s = singlet, d = doublet, t = triplet, q = quadruplet, m = multiplet), coupling constants (Hz) and integration. ^{13}C-NMR spectra were recorded on a BRUKER AVANCE III HD (100 MHz) spectrometer with complete proton decoupling. Chemical shifts are reported in ppm from tetramethylsilane with the solvent resonance as the internal standard ($CDCl_3$: δ 77.16, CD_3CN: δ 118.26, 1.32). ^{19}F-NMR spectra were recorded on a BRUKER AVANCE III HD (376 MHz) spectrometer. Mass spectra were measured with an Agilent Technologies 6120 Quadrupole LC/MS. High resolution mass spectrometry (HRMS) were measured with a GCT Premier™ and BRUKER micrOTF-Q III. Melting points were measured using INESA WRR and values are uncorrected. Quantum yield was measured by using Cary 5000 UV-Vis-NIR spectrophotometer.

2. Reaction condition optimization

2.1 Reaction setups

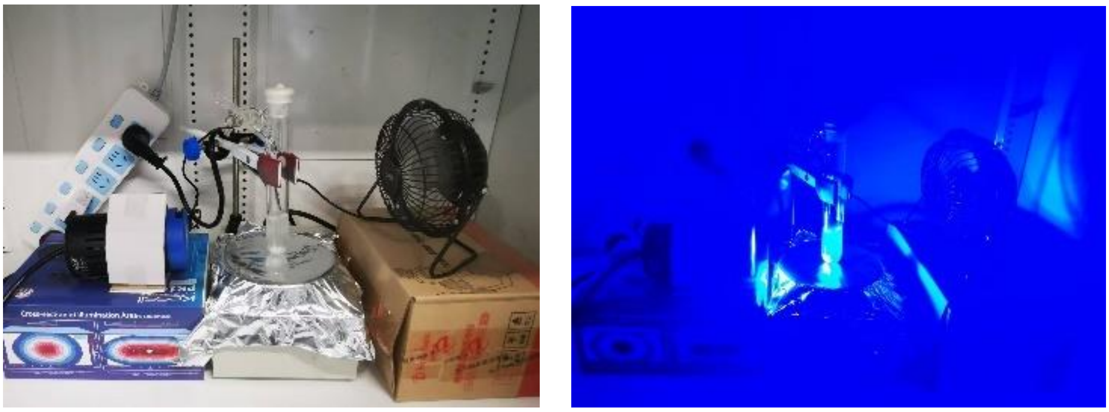

a **b**

Figure S1. (a) Reaction apparatus. (b) Reaction under 456 nm blue LED light.

2.2 Reaction parameters survey

Table S1. Evaluation of photosensitizers[a]

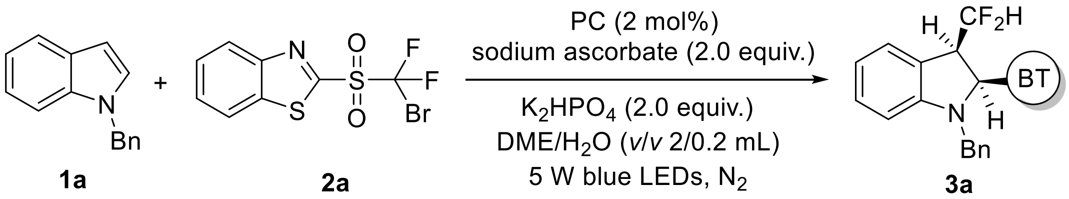

Entry	PC	Yield (%)[b]
1	*fac*-Ir(ppy)$_3$	41
2	Rhodamine 6G	49
**3**	**Fluorescein**	**60**
4	Eosin B	59
5	Ru(bpy)$_3$Cl$_2$·6H$_2$O	49
6	Eosin Y	50
7	4CzIPN	47
8	Ir[dF(CF$_3$)ppy]$_2$(dtbbpy)PF$_6$	42
9	[Ir(dtbbpy)(ppy)$_2$]PF$_6$	44
10	Mes-Acr-ClO$_4$	41

[a] Standard reaction conditions: **1a** (0.2 mmol), **2a** (0.1 mmol), PC (2 mol%), sodium ascorbate (2.0 equiv.) and K_2HPO_4 (2.0 equiv.) in DME/H_2O (*v*/*v* 2.0/0.2 mL), irradiated by 5 W blue LEDs at r.t. under N_2 for 6 h. [b] Yields of isolated products.

Table S2. Evaluation of solvents[a]

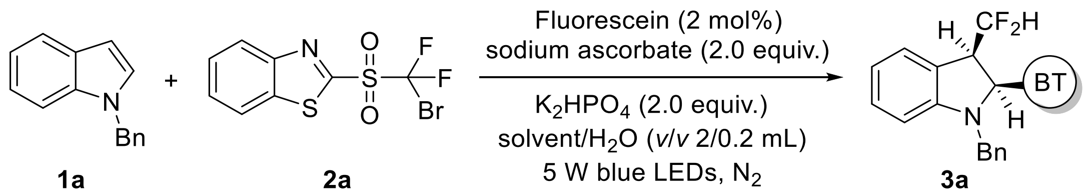

Entry	Solvent (mL)	Yield (%)[b]
**1**	**DME**	**60**
2	DMF	51
3	THF	31
4	MeOH	36
5	DMAc	46
6	DMSO	28
7	MeCN	27

8	EtOAc	14
9	Acetone	15
10	$PhCF_3$	ND

[a] Standard reaction conditions: **1a** (0.2 mmol), **2a** (0.1 mmol), Fluorescein (2 mol%), sodium ascorbate (2.0 equiv.) and K_2HPO_4 (2.0 equiv.) in solvent/H_2O (*v*/*v* 2.0 mL/0.2 mL), irradiated by 5 W blue LEDs at r.t. under N_2 for 6 h. [b] Yields of isolated products.

Table S3. Evaluation of bases[a]

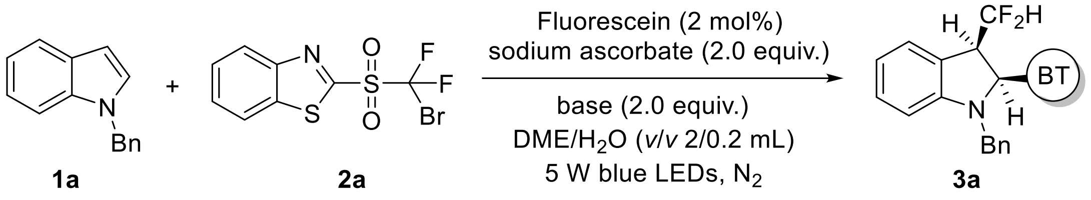

Entry	Base	Yield (%)[b]
1	$KHCO_3$	56
2	KH_2PO_4	56
3	K_3PO_4	44
4	$NaHCO_3$	54
5	Na_2HPO_4	55
6	PhCOONa	52
**7**	**K_2HPO_4**	**60**
8	NaH_2PO_4	54
9	2,6-Lutidine	54
10	DBU	47
11	DABCO	41

[a] Standard reaction conditions: **1a** (0.2 mmol), **2a** (0.1 mmol), Fluorescein (2 mol%), sodium ascorbate (2.0 equiv.) and base (2.0 equiv.) in DME/H_2O (*v*/*v* 2.0 mL/0.2 mL), irradiated by 5 W blue LEDs at r.t. under N_2 for 6 h. [b] Yields of isolated products.

Table S4. Evaluation of the equivalent of base[a]

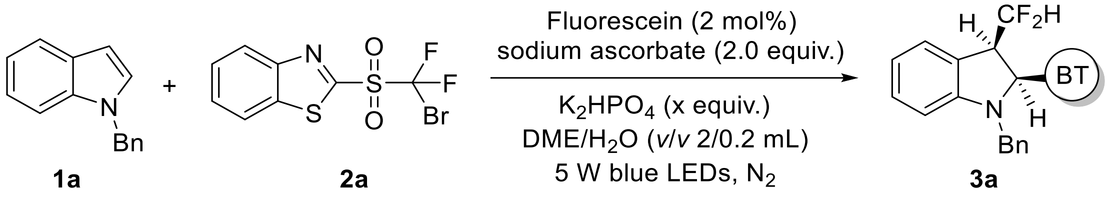

Entry	Base (equiv.)	Yield (%)[b]
1	0.5	56
**2**	**1.0**	**65**
3	1.5	59
4	2.0	60
5	2.5	54

[a] Standard reaction conditions: **1a** (0.2 mmol), **2a** (0.1 mmol), Fluorescein (2 mol%), sodium ascorbate (2.0 equiv.) and K_2HPO_4 (x equiv.) in DME/H_2O (*v*/*v* 2.0 mL/0.2 mL), irradiated by 5 W blue LEDs at r.t. under N_2 for 6 h. [b] Yields of isolated products.

Table S5. Evaluation of the equivalent of sodium ascorbate[a]

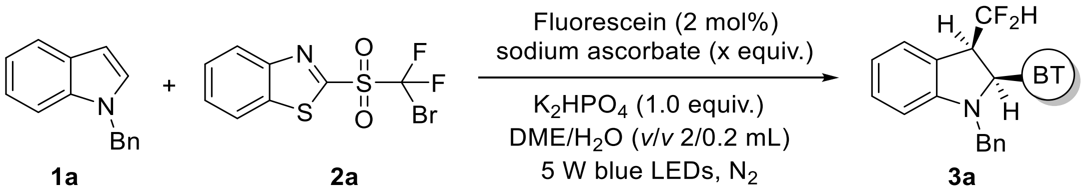

Entry	Sodium ascorbate (equiv.)	Yield (%)[b]
1	0.5	46
2	1.0	55
**3**	**1.5**	**65**
4	2.0	65
4	2.5	54

[a] Standard reaction conditions: **1a** (0.2 mmol), **2a** (0.1 mmol), Fluorescein (2 mol%), sodium ascorbate (x equiv.) and K_2HPO_4 (1.0 equiv.) in DME/H_2O (*v*/*v* 2.0 mL/0.2 mL), irradiated by 5 W blue LEDs at r.t. under N_2 for 6 h. [b] Yields of isolated products.

Table S6. Evaluation of concentrations[a]

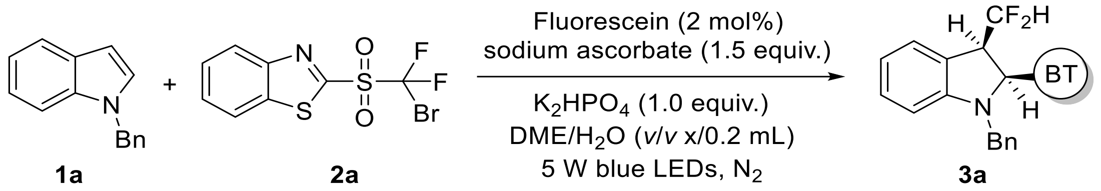

Entry	DME (mL)	Yield (%)[b]
1	0.5	25
2	1.0	48
3	1.5	52
**4**	**2.5**	**65**
5	3.0	50
6	4.0	54

[a] Standard reaction conditions: **1a** (0.2 mmol), **2a** (0.1 mmol), Fluorescein (2 mol%), sodium ascorbate (1.5 equiv.) and K_2HPO_4 (1.0 equiv.) in DME/H_2O (*v*/*v* x mL/0.2 mL), irradiated by 5 W blue LEDs at r.t. under N_2 for 6 h. [b] Yields of isolated products.

Table S7. Evaluation of light sources[a]

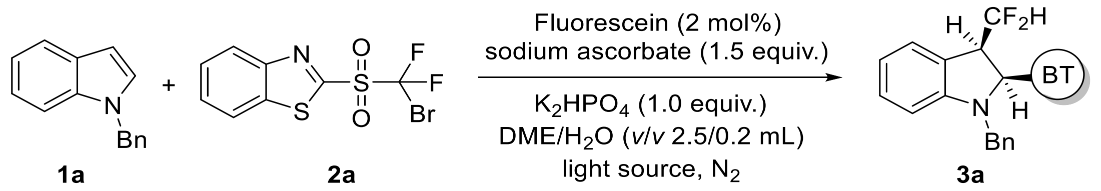

Entry	Light source	Yield (%)[b]
**1**	**5 W blue**	**65**
2	18 W blue	52
3	30 W blue	51
4	5 W green	46
5	30 W white	44

[a] Standard reaction conditions: **1a** (0.2 mmol), **2a** (0.1 mmol), Fluorescein (2 mol%), sodium ascorbate (1.5 equiv.) and K_2HPO_4 (1.0 equiv.) in DME/H_2O (*v*/*v* 2.5 mL/0.2 mL), irradiated by visible light at r.t. under N_2 for 6 h. [b] Yields of isolated products.

Table S8. Control experiments[a]

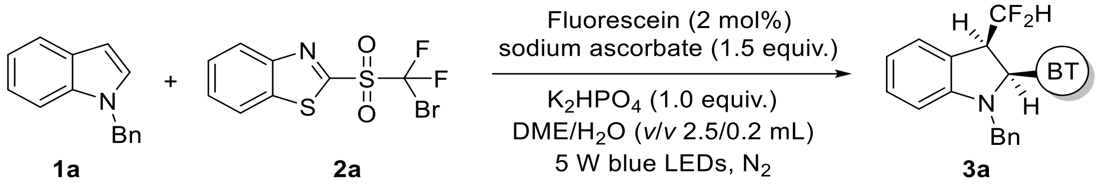

Entry	PC	Base	Sodium ascorbate	Light source	Yield (%)[b]
1	-	+	+	+	49
**2**	-	-	+	+	**52**
3	-	+	-	+	27
4	-	-	-	+	ND
5	+	+	+	-	ND

[a] Standard reaction conditions: **1a** (0.2 mmol), **2a** (0.1 mmol), Fluorescein (2 mol%), sodium ascorbate (1.5 equiv.) and K_2HPO_4 (1.0 equiv.) in DME/H_2O (*v*/*v* 2.5 mL/0.2 mL), irradiated by 5 W blue LEDs at r.t. under N_2 for 6 h. [b] Yields of isolated products. “-” equals “with”, “+” equals “without”.

Table S9. Evaluation of the equivalent of sodium ascorbate[a]

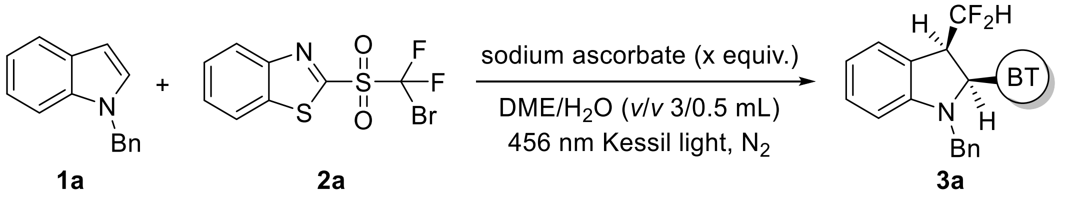

Entry	Sodium ascorbate (equiv.)	Yield (%)[b]
1	1.0	51
2	1.5	49
3	2.0	50
**4**	**2.5**	**56**
5	3.0	49

[a] Standard reaction conditions: **1a** (0.2 mmol), **2a** (0.1 mmol), sodium ascorbate (x equiv.) in DME/H_2O (*v*/*v* 3.0 mL/0.5 mL), irradiated by 456 nm Kessil light at r.t. under N_2 for 6 h. [b] Yields of isolated products.

Table S10. Evaluation of solvents[a]

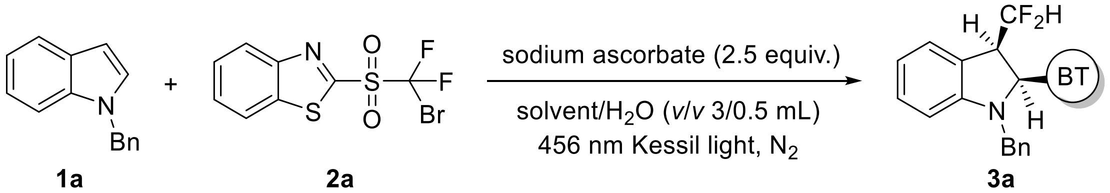

Entry	Sovent (mL)	Yield (%)[b]
**1**	**DME**	**56**
2	DMF	48
3	NMP	40
4	DCE	31
5	EtOAc	28
6	EtOH	35
7	1,4-Dioxane	38
8	THF	44
9	MeCN	Trace
10	Acetone	Trace
11	DMSO	ND

[a] Standard reaction conditions: **1a** (0.2 mmol), **2a** (0.1 mmol), sodium ascorbate (2.5 equiv.) in solvent/H_2O (*v/v* 3.0 mL/0.5 mL), irradiated by 456 nm Kessil light at r.t. under N_2 for 6 h. [b] Yields of isolated products.

Table S11. Evaluation of the equivalent of water[a]

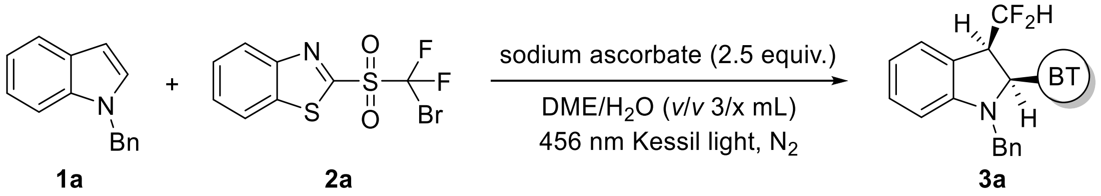

Entry	H_2O (mL)	Yield (%)[b]
1	0	Trace
2	0.2	35

3	0.4	44
**4**	**0.5**	**56**
5	0.6	49
6	0.8	47
7	1.0	40

[a] Standard reaction conditions: **1a** (0.2 mmol), **2a** (0.1 mmol), sodium ascorbate (2.5 equiv.) in solvent/H_2O (*v*/*v* 3.0 mL/x mL), irradiated by 456 nm Kessil light at r.t. under N_2 for 6 h. [b] Yields of isolated products.

Table S12. Evaluation of concentrations[a]

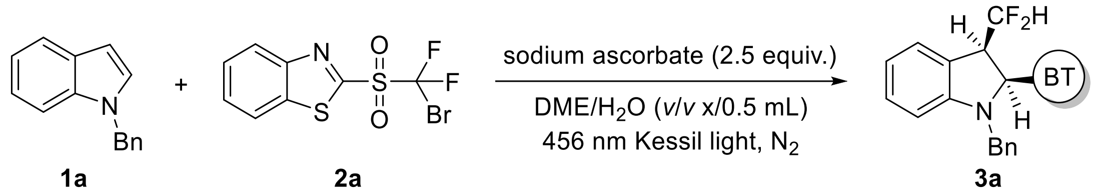

Entry	DME (mL)	Yield (%)[b]
1	2.0	44
2	3.0	56
**3**	**4.0**	**72**
4	5.0	56

[a] Standard reaction conditions: **1a** (0.2 mmol), **2a** (0.1 mmol), sodium ascorbate (2.5 equiv.) in DME/H_2O (*v*/*v* x mL/0.5 mL), irradiated by 456 nm Kessil light at r.t. under N_2 for 6 h. [b] Yields of isolated products.

Table S13. Evaluation of light sources[a]

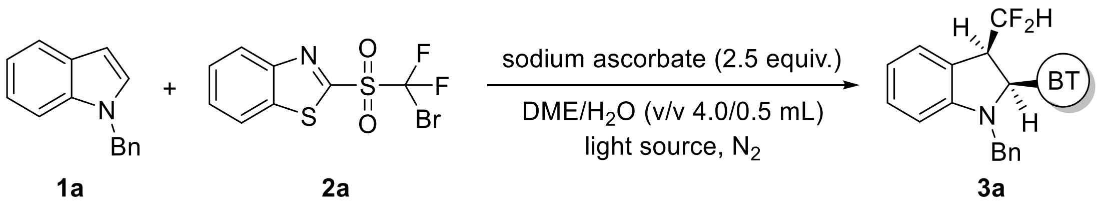

Entry	Light source	Yield (%)[b]
**1**	**456 nm Kessil light**	**72**

2	427 nm Kessil light	62
2	390 nm Kessil light	45
3	30 W blue LEDs	50
4	30 W white LEDs	44

[a] Standard reaction conditions: **1a** (0.2 mmol), **2a** (0.1 mmol), sodium ascorbate (2.5 equiv.) in DME/H_2O (*v*/*v* 4.0 mL/0.5 mL), irradiated by visible light at r.t. under N_2 for 6 h. [b] Yields of isolated products.

Table S14. Evaluation of reductants[a]

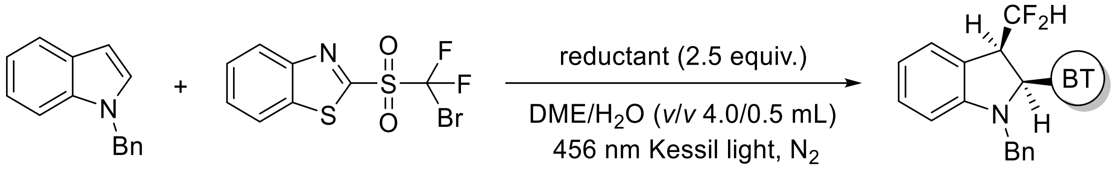

1a **2a** **3a**

Entry	Reductant	Yield (%)[b]
1	TTMSS	15
2	HCOOCs	<10
3	Hantzsch ester	<10
4	*tert*-dodecylthiol	Trace
5	ascorbic acid	40

[a] Standard reaction conditions: **1a** (0.2 mmol), **2a** (0.1 mmol), additive (2.5 equiv.) in DME/H_2O (*v*/*v* 4.0 mL/0.5 mL), irradiated by 456 nm Kessil light at r.t. under N_2 for 6 h. [b] Yields of isolated products.

3. Preparation of starting materials

3.1 Synthesis of indole and benzofuran derivatives

Indole derivatives (**1a**[1], **1b** and **3x**[2], **1c**、**1d**、**1e** and **1f**[3], **1g**[4], **1j**、**3l**、**3m**、**3o**、**3p** and **3q**[5], **1k**、**1n** and **1u**[6], **1s**[7], **1t**[8], **1v**[9], **1w**[10], **1ab**[11]) were prepared following reported procedures, and characterization data are in agreement with the corresponding literatures. The unreported indoles and benzofurans are prepared according to the following procedures. Other indole, furan, (benzo)thiophene derivatives and polyaromatic hydrocarbons, which are not mentioned herein, are commercially available.

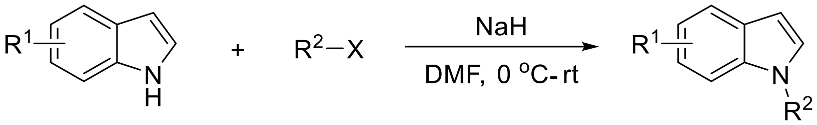

A solution of indole (1.0 equiv.) in DMF (0.1 M) was cooled to 0 °C, NaH (1.2 equiv.) was then added portionwise under ice-cooling. The resulting suspension was stirred at 0 °C for 15 min then allowed to warm up to room temperature and stirred for 1 h. The electrophile (1.2 equiv.) was then added and the resulting mixture was stirred until completion of the reaction. Then, the reaction mixture was cooled to 0 °C and quenched with water. Ethyl acetate was added and the organic phase was separated. The aqueous layer was extracted 3 times with ethyl acetate. The combined organic layers were washed with brine, dried over $MgSO_4$ and evaporated to give the crude product, which was further purified with flash column chromatography on silica gel.

Indole **1h**, **1y**, and **1z** are new compounds.

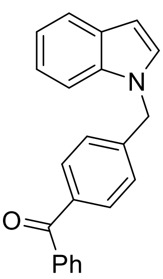

(4-((1*H*-indol-1-yl)methyl)phenyl)(phenyl)methanone (1h): ^{1}H NMR (500 MHz, $CDCl_3$) δ 7.77 (d, *J* = 7.5 Hz, 2H), 7.74 (d, *J* = 8.0 Hz, 2H), 7.69 (d, *J* = 7.5 Hz, 1H), 7.60-7.55 (m, 1H), 7.49-7.44 (m, 2H), 7.28 (d, *J* = 8.0 Hz, 1H), 7.23-7.17 (m, 4H), 7.17-7.13 (m, 1H), 6.61 (d, *J* = 3.0 Hz, 1H), 5.42 (s, 2H); ^{13}C NMR (125 MHz, $CDCl_3$) δ 196.3, 142.4, 137.5, 137.0, 136.3, 132.6, 130.7, 130.1, 128.9, 128.4, 128.4, 126.6, 122.0, 121.2, 119.9, 109.7, 102.3, 50.0. FT-IR: ν (cm^{-1}) 3034, 2145, 1509, 1378, 1223, 995. HRMS [ESI] calcd for $C_{22}H_{18}NO$ $[M+H]^+$ 312.1383, found 312.1386.

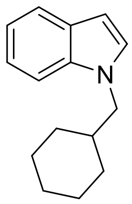

1-(cyclohexylmethyl)-1H-indole (1y): ^{1}H NMR (400 MHz, $CDCl_3$) δ 7.63 (d, *J* = 8.0 Hz, 1H), 7.34 (d, *J* = 8.4 Hz, 1H), 7.22-7.18 (m, 1H), 7.12-7.08 (m, 1H), 7.07 (d, *J* = 2.8 Hz, 1H), 6.48 (d, *J* = 2.8 Hz, 1H), 3.94 (d, *J* = 7.2 Hz, 2H), 1.92-1.80 (m, 1H), 1.75-1.68 (m, 2H), 1.67-1.60 (m, 3H), 1.21-1.13 (m, 3H), 1.05-0.95 (m, 2H); ^{13}C NMR (125 MHz, $CDCl_3$) δ 136.4, 128.7, 128.6, 121.3, 121.0, 119.2, 109.8, 100.7, 53.1, 39.0, 31.2, 26.4, 25.9. FT-IR: ν (cm^{-1}) 3089, 2345, 1567, 1456, 1178, 889. HRMS [ESI] calcd for $C_{15}H_{20}N$ $[M+H]^+$ 214.1590, found 214.1593.

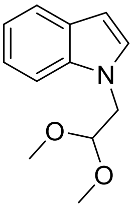

1-(2,2-dimethoxyethyl)-1*H*-indole (1z): ^{1}H NMR (500 MHz, $CDCl_3$) δ 7.63 (d, *J* = 8.0 Hz, 1H), 7.39 (d, *J* = 8.5 Hz, 1H), 7.24-7.20 (m, 1H), 7.15 (d, *J* = 3.0 Hz, 1H), 7.13-7.10 (m, 1H), 6.51 (d, *J* = 3.0 Hz, 1H), 4.56 (t, *J* = 5.0 Hz, 1H), 4.22 (d, *J* = 5.0 Hz, 2H), 3.35 (s, 6H); ^{13}C NMR (125 MHz, $CDCl_3$) δ 136.4, 128.7, 128.7, 121.6, 121.0, 119.5, 109.3, 103.6, 101.7, 55.0, 48.9; FT-IR: ν (cm^{-1}) 3123, 2890, 1654, 1324, 1178, 985. HRMS [ESI] calcd for $C_{12}H_{16}NO_2$ $[M+H]^+$ 206.1176, found 206.1180.

3.2 Synthesis of sulfone bifunctional reagents

Compound **2ad-2am**[12] were prepared following the reported procedures, and the characterization data agree with the literatures. Other sulfone reagents are new and prepared according to the following procedures:

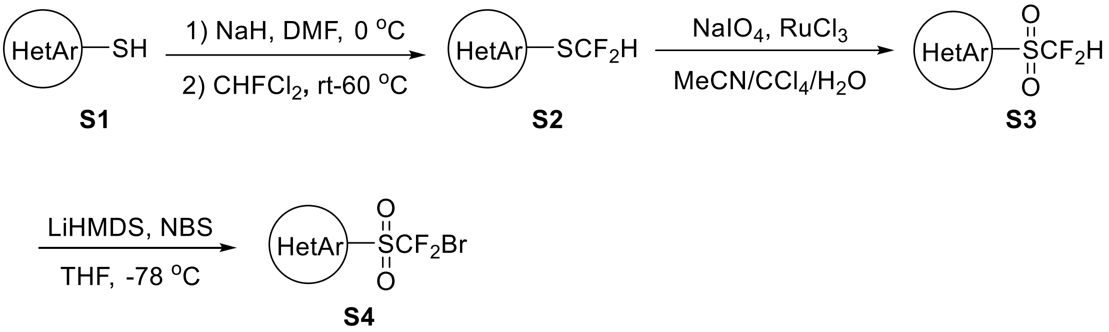

First Step: A dry flask was evacuated and backfilled with pure N_2 for 3 times. NaH (33 mol, 60 wt % in mineral oil) followed by DMF (80 mL) was added at 0°C. Then **S1** (30 mol, 1.0 equiv.) in DMF (20 mL) was added dropwise with syringe at 0°C and the mixture was stirred at rt for 0.5 h. $CHClF_2$ gas was bubbled to the system for 2 h. The mixture was stirred at rt for 12 h (monitored by TLC). After the reaction was complete, the mixture was quenched dropwise by H_2O at 0°C. The mixture was extracted with Et_2O for 3 times. Then the organic phase was combined and dried over anhydrous $MgSO_4$. The solvent was removed under vacuum and the residue was purified by flash column chromatography on silica gel to provide **S2**.

Second Step: To a flask **S2** (20 mmol, 1.0 equiv.), CH_3CN (20 mL), CCl_4 (20 mL), H_2O (40 mL), and $RuCl_3{\cdot}3H_2O$ (10 mg) were added. Then $NaIO_4$ (50 mol, 2.5 equiv.) was added. The mixture was stirred at rt for 12 h (monitored by TLC). After the reaction was complete, the mixture was neutralized by saturated aq. $NaHCO_3$. After filtration, the filter residue was washed with EtOAc. The filtrate mixture was extracted with EtOAc for 3 times. Then the organic phase was combined and dried over anhydrous $MgSO_4$. The solvent was removed under vacuum and the residue was purified by flash column chromatography on silica gel to provide **S3**.

Third Step: Sulfones **S3** (5 mmol, 1.0 equiv.) and NBS (15 mmol, 3.0 equiv.) were added to a dry Schlenk tube. The flask was evacuated and backfilled with pure N_2 for 3 times. Then THF (20 mL) was added with syringe under N_2 atmosphere. The mixture was cooled to –78 °C. LiHMDS (1.0 M in THF, 15 mmol, 15 mL, 3.0 equiv.) was added with syringe under N2 atmosphere in 15 min. The mixture was stirred at -78°C for 3 h (monitored by TLC). After the reaction was complete, the mixture was quenched by saturated aq. NH_4Cl (10 mL). After the mixture was warmed to rt, the aqueous layer was extracted with Et_2O for 3 times. Then the organic phase was combined and dried over anhydrous $MgSO_4$. The solvent was removed under vacuum and the residue was purified by flash column chromatography on silica gel to provide the sulfones **S4**.

Reagent **2an** and **2ao** are new compounds.

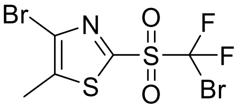

4-bromo-2-((bromodifluoromethyl)sulfonyl)-5-methylthiazole (2an): ^{1}H NMR (500 MHz, $CDCl_3$) δ 2.57 (s, 3H); ^{13}C NMR (125 MHz, $CDCl_3$) δ 157.7, 152.7, 119.6 (t, $J_{C\text{-}F}$ = 347.5 Hz), 118.7, 16.0; ^{19}F NMR (470 MHz, $CDCl_3$) δ -56.0. FT-IR: ν (cm^{-1}) 1567, 1323, 1245, 1204, 895, 765. HRMS [ESI] calcd for $C_5H_4Br_2F_2NO_2S_2$ $[M+H]^+$ 371.7992, found 371.7990.

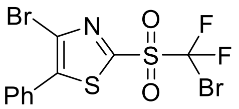

4-bromo-2-((bromodifluoromethyl)sulfonyl)-5-phenylthiazole (2ao): ^{1}H NMR (500 MHz, $CDCl_3$) δ 7.98-7.92 (m, 2H), 7.54-7.46 (m, 3H); ^{13}C NMR (125 MHz, $CDCl_3$) δ 157.5, 153.8, 131.2, 130.1, 129.0, 128.8, 120.0 (t, J_{C-F} = 347.8 Hz), 116.5; ^{19}F NMR (470 MHz, $CDCl_3$) δ -55.8. FT-IR: ν (cm^{-1}) 1567, 1323, 1245, 1204, 895, 765. HRMS [ESI] calcd for $C_{10}H_6Br_2F_2NO_2S_2$ $[M+H]^+$ 433.8149, found 433.8146.

4. General procedures for dearomatization of (hetero)arenes

To a flame-dried Schlenk tube was added heteroarene **1** or PAH (0.4 mmol), sulfone bifunctional reagent **2** (0.2 mmol) and sodium ascorbate (0.5 mmol), which was subjected to evacuation/ flushing with N_2 for 3 times. Dry DME (8.0 mL)/ H_2O (1.0 mL) was added to the mixture via syringe, which was irradiated by 456 nm Kessil light and stirred at r.t. until the starting material had been consumed as determined by TLC. The mixture was quenched with H_2O. The aqueous layer was extracted with DCM. The combine organic layers were washed with brine, dried over Na_2SO_4, concentrated in vacuo, and purified by flash column chromatography on silica gel (eluent: ethyl acetate/petroleum ether) to give the corresponding product **3-7**.

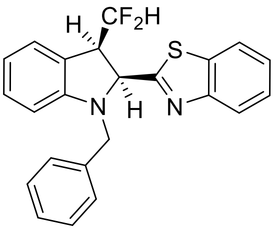

2-(1-benzyl-3-(difluoromethyl)indolin-2-yl)benzo[*d*]thiazole (3a): Colorless oil. Purification by flash column chromatography (eluent: EtOAc/Petroleum ether = 1/20). ^{1}H NMR (400 MHz, $CDCl_3$) δ 8.05 (d, *J* = 8.0 Hz, 1H), 7.86 (d, *J* = 8.0 Hz, 1H), 7.54-7.48 (m, 1H), 7.44-7.39 (m, 1H), 7.36-7.27 (m, 6H), 7.22-7.17 (m, 1H), 6.89-6.84 (m, 1H), 6.57 (d, *J* = 8.0 Hz, 1H), 5.82 (td, *J* = 55.6, 5.6 Hz, 1H), 5.39 (d, *J* = 9.6 Hz, 1H), 4.56 (d, *J* = 15.6 Hz, 1H), 4.20-4.08 (m, 2H); ^{13}C NMR (100 MHz, $CDCl_3$) δ 169.1, 153.2, 152.2, 136.9, 135.0, 129.7, 128.8, 127.8, 127.6, 126.7 (d, J_{C-F} = 2.3 Hz), 126.4, 125.7, 123.7 (d, J_{C-F} = 6.6 Hz), 123.4, 122.0, 120.1, 115.1 (t, J_{C-F} = 241.2 Hz), 109.7, 67.8 (dd, J_{C-F} = 6.6, 3.1 Hz), 52.5, 50.1 (t, J_{C-F} = 21.9 Hz); ^{19}F NMR (470 MHz, $CDCl_3$) δ -115.8 (d, *J* = 287.6 Hz), -121.1 (d, *J* = 287.6 Hz). FT-IR: ν (cm^{-1}) 2953, 1454, 1205, 1145, 752, 687. HRMS [ESI] calcd for $C_{23}H_{19}F_2N_2S$ $[M+H]^+$ 393.1232, found 393.1238.

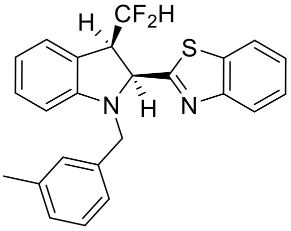

2-(3-(difluoromethyl)-1-(3-methylbenzyl)indolin-2-yl)benzo[*d*]thiazole (3b): Colorless oil. Purification by flash column chromatography (eluent: EtOAc/Petroleum ether = 1/20). ^{1}H NMR (400 MHz, $CDCl_3$) δ 8.05 (d, *J* = 8.4 Hz, 1H), 7.86 (d, *J* = 8.4 Hz, 1H), 7.54-7.49 (m, 1H), 7.44-7.39 (m, 1H), 7.32 (d, *J* = 7.2 Hz, 1H), 7.25-7.17 (m, 2H), 7.16-7.07 (m, 3H), 6.87 (td, *J* = 7.6, 1.2 Hz, 1H), 6.58 (d, *J* = 8.0 Hz, 1H), 5.83 (td, *J* = 55.6, 5.6 Hz, 1H), 5.39 (d, *J* = 9.6 Hz, 1H), 4.51 (d, *J* = 15.6 Hz, 1H), 4.21-4.07 (m, 2H), 2.33 (s, 3H); ^{13}C NMR (100 MHz, $CDCl_3$) δ 169.2, 153.1, 152.2, 138.5, 136.9, 135.0, 129.7, 128.7, 128.5, 128.4, 126.6 (d, J_{C-F} = 2.1 Hz), 126.4, 125.8, 124.8, 123.7 (d, J_{C-F} = 6.5 Hz), 123.3, 122.0, 120.0, 115.1 (t, J_{C-F} = 241.2 Hz), 109.7, 67.8 (dd, J_{C-F} = 6.6, 2.9 Hz), 52.6, 50.1 (t, J_{C-F} = 21.9 Hz), 21.6; ^{19}F NMR (376 MHz, $CDCl_3$) δ -115.6 (d, *J* = 287.3 Hz), -121.0 (d, *J* = 287.3 Hz). FT-IR: ν (cm^{-1}) 3055, 1435, 1244, 1057, 906, 756. HRMS [ESI] calcd for $C_{24}H_{21}F_2N_2S$ $[M+H]^+$ 407.1388, found 407.1385.

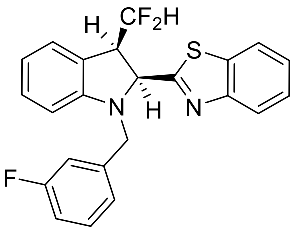

2-(3-(difluoromethyl)-1-(3-fluorobenzyl)indolin-2-yl)benzo[*d*]thiazole (3c): Colorless oil. Purification by flash column chromatography (eluent: EtOAc/Petroleum ether = 1/20). ^{1}H NMR (500 MHz, $CDCl_3$) δ 8.04 (d, *J* = 8.5 Hz, 1H), 7.87 (d, *J* = 8.0 Hz, 1H), 7.54-7.49 (m, 1H), 7.44-7.40 (m, 1H), 7.35-7.28 (m, 2H), 7.22-7.17 (m, 1H), 7.13-7.09 (m, 2H), 6.98 (td, *J* = 8.5, 2.5 Hz, 1H), 6.89 (td, *J* = 7.5, 1.0 Hz, 1H), 6.51 (d, *J* = 7.5 Hz, 1H), 5.83 (td, *J* = 55.0, 5.5 Hz, 1H), 5.39 (d, *J* = 9.0 Hz, 1H), 4.49 (d, *J* = 16.0 Hz, 1H), 4.20-4.10 (m, 2H); ^{13}C NMR (125 MHz, $CDCl_3$) δ 168.7, 163.3 (d, $J_{C\text{-}F}$ = 244.9 Hz), 153.2, 152.0, 139.9 (d, $J_{C\text{-}F}$ = 7.1 Hz), 134.9, 130.4 (d, $J_{C\text{-}F}$ = 8.1 Hz), 129.8, 126.7 (d, $J_{C\text{-}F}$ = 1.6 Hz), 126.5, 125.8, 123.8 (d, $J_{C\text{-}F}$ = 6.4 Hz), 123.4, 123.2 (d, $J_{C\text{-}F}$ = 2.9 Hz), 122.0, 120.4, 114.7 (t, $J_{C\text{-}F}$ = 241.3 Hz), 114.6, 114.5, 109.7, 68.1 (dd, $J_{C\text{-}F}$ = 6.4, 2.8 Hz), 52.4 (d, $J_{C\text{-}F}$ = 1.3 Hz), 50.1 (t, $J_{C\text{-}F}$ = 22.0 Hz); ^{19}F NMR (470 MHz, $CDCl_3$) δ -112.5 (s), -115.8 (d, *J* = 287.6 Hz), -120.8 (d, *J* = 287.6 Hz). FT-IR: ν (cm^{-1}) 3005, 1467, 1245, 1045, 757, 729. HRMS [ESI] calcd for $C_{23}H_{18}F_3N_2S$ $[M+H]^+$ 411.1137, found 411.1136.

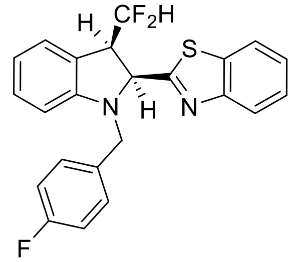

2-(3-(difluoromethyl)-1-(4-fluorobenzyl)indolin-2-yl)benzo[*d*]thiazole (3d): Colorless oil. Purification by flash column chromatography (eluent: EtOAc/Petroleum ether = 1/20). ^{1}H NMR (400 MHz, $CDCl_3$) δ 8.04 (d, *J* = 8.0 Hz, 1H), 7.87 (d, *J* = 8.0 Hz, 1H), 7.54-7.49 (m, 1H), 7.45-7.40 (m, 1H), 7.34-7.27 (m, 3H), 7.23-7.17 (m, 1H), 7.05-6.99 (m, 2H), 6.90-6.85 (m, 1H), 6.55 (d, *J* = 8.0 Hz, 1H), 5.81 (td, *J* = 55.6, 5.6 Hz, 1H), 5.36 (d, *J* = 9.6 Hz, 1H), 4.50 (d, *J* = 16.0 Hz, 1H), 4.19-4.07 (m, 2H); ^{13}C NMR (100 MHz, $CDCl_3$) δ 168.8, 162.4 (d, $J_{C\text{-}F}$ = 244.7 Hz), 153.1, 152.0, 135.0, 132.6 (d, $J_{C\text{-}F}$ = 3.0 Hz), 129.8, 129.4 (d, $J_{C\text{-}F}$ = 7.9 Hz), 126.7 (d, $J_{C\text{-}F}$ = 2.2 Hz), 126.5, 125.9, 123.8 (d, $J_{C\text{-}F}$ = 6.3 Hz), 123.4, 122.0, 120.3, 115.7 (d, $J_{C\text{-}F}$ = 21.0 Hz), 115.5 (t, $J_{C\text{-}F}$ = 241.3 Hz), 109.6, 67.7 (dd, $J_{C\text{-}F}$ = 6.6, 3.1 Hz), 51.9, 50.1 (t, $J_{C\text{-}F}$ = 22.2 Hz); ^{19}F NMR (376 MHz, $CDCl_3$) δ -115.0 (s), -115.8 (d, *J* = 288.0 Hz), -121.1 (d, *J* = 288.0 Hz). FT-IR: ν (cm^{-1}) 2928, 1434, 1211, 1056, 978, 765. HRMS [ESI] calcd for $C_{23}H_{18}F_3N_2S$ $[M+H]^+$ 411.1137, found 411.1144.

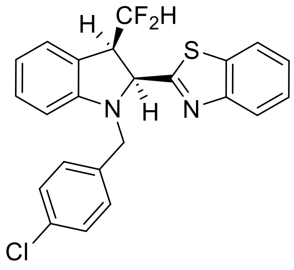

2-(1-(4-chlorobenzyl)-3-(difluoromethyl)indolin-2-yl)benzo[*d*]thiazole (3e): Colorless oil. Purification by flash column chromatography (eluent: EtOAc/Petroleum ether = 1/20). ^{1}H NMR (500 MHz, $CDCl_3$) δ 8.04 (d, *J* = 8.5 Hz, 1H), 7.86 (d, *J* = 8.0 Hz, 1H), 7.54-7.49 (m, 1H), 7.45-7.40 (m, 1H), 7.34-7.27 (m, 5H), 7.19 (t, *J* = 7.5 Hz, 1H), 6.89 (t, *J* = 7.5 Hz, 1H), 6.51 (d, *J* = 8.0 Hz, 1H), 5.81 (td, *J* = 55.5, 6.0 Hz, 1H), 5.36 (d, *J* = 9.0 Hz, 1H), 4.48 (d, *J* = 16.0 Hz, 1H), 4.18-4.08 (m, 2H); ^{13}C NMR (125 MHz, $CDCl_3$) δ 168.7, 153.2, 151.9, 135.5, 134.9, 133.4, 129.8, 129.0, 129.0, 126.7 (d, $J_{C\text{-}F}$ = 2.4 Hz), 126.5, 125.9, 123.8 (d, $J_{C\text{-}F}$ = 6.3 Hz), 123.4, 122.0, 120.4, 115.0 (t, $J_{C\text{-}F}$ = 241.3 Hz), 109.7, 68.0 (dd, $J_{C\text{-}F}$ = 6.5, 2.9 Hz), 52.1, 50.1 (t, $J_{C\text{-}F}$ = 22.0 Hz); ^{19}F NMR (376 MHz, $CDCl_3$) δ -115.9 (d, *J* = 287.6 Hz), -121.2 (d, *J* = 287.6 Hz). FT-IR: ν (cm^{-1}) 2870, 1598, 1455, 1205, 752, 687. HRMS [ESI] calcd for $C_{23}H_{18}ClF_2N_2S$ $[M+H]^+$ 427.0842, found 427.0846.

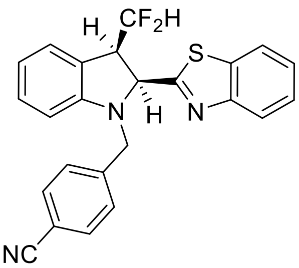

4-((2-(benzo[*d*]thiazol-2-yl)-3-(difluoromethyl)indolin-1-yl)methyl)benzonitrile (3f): Colorless oil. Purification by flash column chromatography (eluent: EtOAc/Petroleum ether = 1/20). ^{1}H NMR (500 MHz, $CDCl_3$) δ 8.03 (d, *J* = 8.5 Hz, 1H), 7.85 (d, *J* = 8.0 Hz, 1H), 7.68-7.62 (m, 2H), 7.54-7.48 (m, 3H), 7.45-7.41 (m, 1H), 7.35 (d, *J* = 7.5 Hz, 1H), 7.21-7.17 (m, 1H), 6.91 (td, *J* = 7.5, 1.0 Hz, 1H), 6.41 (d, *J* = 8.0 Hz, 1H), 5.83 (td, *J* = 55.0, 5.5 Hz, 1H), 5.40 (d, *J* = 9.5 Hz, 1H), 4.50 (d, *J* = 17.0 Hz, 1H), 4.24-4.12 (m, 2H); ^{13}C NMR (125 MHz, $CDCl_3$) δ 168.2, 153.1, 151.7, 142.9, 134.8, 132.7, 129.9, 128.2, 126.8 (d, $J_{C\text{-}F}$ = 1.3 Hz), 126.6, 126.0, 123.8 (d, $J_{C\text{-}F}$ = 6.3 Hz), 123.4, 122.0, 120.8, 118.8, 114.9 (t, $J_{C\text{-}F}$ = 241.4 Hz), 111.5, 109.5, 68.5 (dd, $J_{C\text{-}F}$ = 6.4, 3.4 Hz), 52.8, 50.1 (t, $J_{C\text{-}F}$ = 22.0 Hz); ^{19}F NMR (376 MHz, $CDCl_3$) δ -116.1 (d, *J* = 288.1 Hz), -121.2 (d, *J* = 288.1 Hz). FT-IR: ν (cm^{-1}) 2997, 1598, 1466, 1205, 752, 687. HRMS [ESI] calcd for $C_{24}H_{18}F_2N_3S$ [M+H]$^+$ 418.1184, found 418.1182.

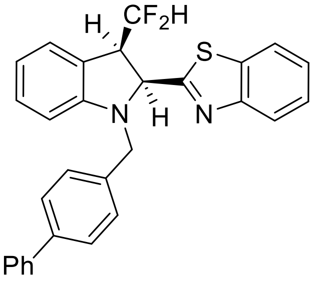

2-(1-([1,1'-biphenyl]-4-ylmethyl)-3-(difluoromethyl)indolin-2-yl)benzo[*d*]thiazole (3g): Colorless oil. Purification by flash column chromatography (eluent: EtOAc/Petroleum ether = 1/20). ^{1}H NMR (400 MHz, $CDCl_3$) δ 8.06 (d, *J* = 8.0 Hz, 1H), 7.87 (d, *J* = 8.0 Hz, 1H), 7.62-7.56 (m, 4H), 7.54-7.50 (m, 1H), 7.48-7.40 (m, 5H), 7.38-7.32 (m, 2H), 7.24-7.20 (m, 1H), 6.89 (td, *J* = 7.6, 1.2 Hz, 1H), 6.63 (d, *J* = 8.0 Hz, 1H), 5.84 (td, *J* = 55.2, 5.6 Hz, 1H), 5.43 (d, *J* = 9.6 Hz, 1H), 4.60 (d, *J* = 15.6 Hz, 1H), 4.23-4.11 (m, 2H); ^{13}C NMR (100 MHz, $CDCl_3$) δ 169.1, 153.2, 152.2, 140.9, 140.6, 136.0, 135.0, 129.8, 128.9, 128.2, 127.5, 127.5, 127.2, 126.7 (d, $J_{C\text{-}F}$ = 2.2 Hz), 126.4, 125.8, 123.8 (d, $J_{C\text{-}F}$ = 6.5 Hz), 123.4, 122.0, 120.2, 115.1 (t, $J_{C\text{-}F}$ = 241.2 Hz), 109.8, 67.8 (dd, $J_{C\text{-}F}$ = 6.8, 3.2 Hz), 52.3, 50.1 (t, $J_{C\text{-}F}$ = 22.2 Hz); ^{19}F NMR (376 MHz, $CDCl_3$) δ -115.8 (d, *J* = 287.6 Hz), -121.1 (d, *J* = 287.6 Hz). FT-IR: ν (cm^{-1}) 2923, 1516, 1433, 1059, 906, 727. HRMS [ESI] calcd for $C_{29}H_{23}F_2N_2S$ [M+H]$^+$ 469.1545, found 469.1555.

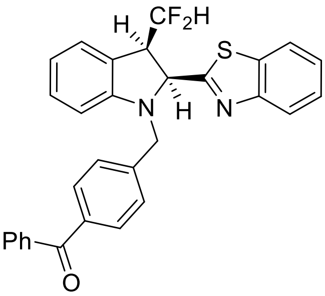

(4-((2-(benzo[*d*]thiazol-2-yl)-3-(difluoromethyl)indolin-1-yl)methyl)phenyl)(phenyl)methanone (3h): Colorless oil. Purification by flash column chromatography (eluent: EtOAc/Petroleum ether = 1/20). ^{1}H NMR (400 MHz, $CDCl_3$) δ 8.05 (d, *J* = 8.4 Hz, 1H), 7.87 (d, *J* = 8.0 Hz, 1H), 7.82-7.76 (m, 4H), 7.62-7.56 (m, 1H), 7.54-7.46 (m, 5H), 7.44-7.40 (m, 1H), 7.34 (d, *J* = 7.2 Hz, 1H), 7.24-7.18 (m, 1H), 6.90 (t, *J* = 7.2 Hz, 1H), 6.53 (d, *J* = 8.0 Hz, 1H), 5.84 (td, *J* = 55.6, 5.6 Hz, 1H), 5.43 (d, *J* = 9.6 Hz, 1H), 4.57 (d, *J* = 16.4 Hz, 1H), 4.24 (d, *J* = 16.0 Hz, 1H), 4.20-4.10 (m, 1H); ^{13}C NMR (100 MHz, $CDCl_3$) δ 196.4, 168.7, 153.1, 152.0, 142.0, 137.7, 137.0, 134.9, 132.6, 130.7, 130.2, 129.8, 128.4, 127.5, 126.8 (d, $J_{C\text{-}F}$ = 2.1 Hz), 126.5, 125.9, 123.8 (d, $J_{C\text{-}F}$ = 6.1 Hz), 123.4, 122.0, 120.5, 115.0 (t, $J_{C\text{-}F}$ = 241.6 Hz), 109.7, 68.2 (dd, $J_{C\text{-}F}$ = 6.3, 3.0 Hz), 52.7, 50.2 (t, $J_{C\text{-}F}$ = 22.2 Hz); ^{19}F NMR (376 MHz, $CDCl_3$) δ -115.9 (d, *J* = 288.0 Hz), -121.1 (d, *J* = 288.0 Hz). FT-IR: ν (cm^{-1}) 3056, 1482, 1247, 1057, 906, 726. HRMS [ESI] calcd for $C_{30}H_{22}F_2N_2OSNa$ [M+Na]$^+$ 519.1313, found 519.1299.

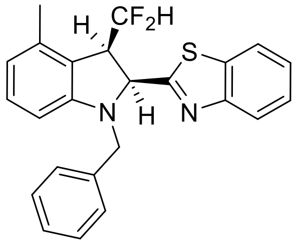

2-(1-benzyl-3-(difluoromethyl)-4-methylindolin-2-yl)benzo[*d*]thiazole (3i): Colorless oil. Purification by flash column chromatography (eluent: EtOAc/Petroleum ether = 1/20). ^{1}H NMR (500 MHz, $CDCl_3$) δ 8.05 (d, *J* = 8.5 Hz, 1H), 7.88 (d, *J* = 8.0 Hz, 1H), 7.54-7.50 (m, 1H), 7.44-7.40 (m, 3H), 7.37-7.34 (m, 2H), 7.31-7.27 (m, 1H), 7.06 (t, *J* = 7.5 Hz, 1H), 6.72 (d, *J* = 7.5 Hz, 1H), 6.37 (d, *J* = 8.0 Hz, 1H), 5.90 (td, *J* = 55.0, 5.0 Hz, 1H), 5.31 (d, *J* = 8.5 Hz, 1H), 4.55 (d, *J* = 16.0 Hz, 1H), 4.14 (d, *J* = 16.0 Hz, 1H), 4.08-4.00 (m, 1H), 2.33 (s, 3H); ^{13}C NMR (125 MHz, $CDCl_3$) δ 169.4, 153.4, 153.2, 137.2, 136.9, 135.1, 129.6, 128.8, 127.6, 127.4, 126.4, 125.7, 123.2, 123.0 (d, J_{C-F} = 1.6 Hz), 122.7, 122.1, 115.5 (t, J_{C-F} = 243.1 Hz), 108.3, 69.7 (dd, J_{C-F} = 4.6, 3.5 Hz), 54.2, 49.2 (t, J_{C-F} = 21.8 Hz), 19.5 (dd, J_{C-F} = 4.1, 2.9 Hz); ^{19}F NMR (470 MHz, $CDCl_3$) δ -114.5 (d, *J* = 281.5 Hz), -120.4 (d, *J* = 281.5 Hz). FT-IR: ν (cm^{-1}) 2945, 1598, 1487, 1205, 752, 687. HRMS [ESI] calcd for $C_{24}H_{21}F_2N_2S$ $[M+H]^+$ 407.1388, found 407.1391.

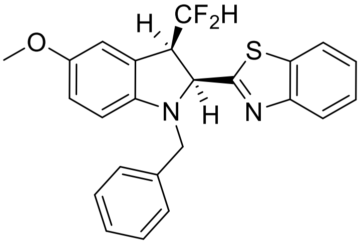

2-(1-benzyl-3-(difluoromethyl)-5-methoxyindolin-2-yl)benzo[*d*]thiazole (3j): Colorless oil. Purification by flash column chromatography (eluent: EtOAc/Petroleum ether = 1/20). ^{1}H NMR (500 MHz, $CDCl_3$) δ 8.03 (d, *J* = 8.0 Hz, 1H), 7.86 (d, *J* = 8.0 Hz, 1H), 7.53-7.48 (m, 1H), 7.43-7.39 (m, 1H), 7.38-7.32 (m, 4H), 7.31-7.25 (m, 1H), 6.93 (s, 1H), 6.73 (dd, *J* = 8.5, 2.5 Hz, 1H), 6.44 (d, *J* = 8.5 Hz, 1H), 5.81 (td, *J* = 55.0, 5.0 Hz, 1H), 5.33 (d, *J* = 9.0 Hz, 1H), 4.48 (d, *J* = 15.5 Hz, 1H), 4.13-4.04 (m, 2H), 3.76 (s, 3H); ^{13}C NMR (125 MHz, $CDCl_3$) δ 169.5, 154.4, 153.3, 146.3, 137.2, 135.1, 128.8, 127.8, 127.6, 126.4, 125.7, 125.2 (d, J_{C-F} = 6.6 Hz), 123.3, 122.0, 114.8, 114.4 (t, J_{C-F} = 241.3 Hz), 113.3, 68.5 (dd, J_{C-F} = 6.1, 3.5 Hz), 56.1, 53.8, 50.3 (t, J_{C-F} = 21.4 Hz); ^{19}F NMR (470 MHz, $CDCl_3$) δ -116.0 (d, *J* = 287.6 Hz), -121.2 (d, *J* = 287.6 Hz). FT-IR: ν (cm^{-1}) 2954, 1598, 1467, 1205, 752, 687. HRMS [ESI] calcd for $C_{24}H_{21}F_2N_2OS$ $[M+H]^+$ 423.1337, found 423.1340.

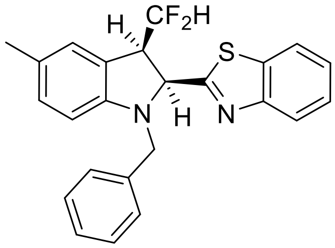

2-(1-benzyl-3-(difluoromethyl)-5-methylindolin-2-yl)benzo[*d*]thiazole (3k): Colorless oil. Purification by flash column chromatography (eluent: EtOAc/Petroleum ether = 1/20). ^{1}H NMR (400 MHz, $CDCl_3$) δ 8.04 (d, *J* = 8.0 Hz, 1H), 7.86 (d, *J* = 8.0 Hz, 1H), 7.54-7.48 (m, 1H), 7.44-7.38 (m, 1H), 7.36-7.28 (m, 5H), 7.14 (s, 1H), 6.99 (d, *J* = 8.4 Hz, 1H), 6.45 (d, *J* = 8.0 Hz, 1H), 5.81 (td, *J* = 55.6, 5.6 Hz, 1H), 5.35 (d, *J* = 9.6 Hz, 1H), 4.52 (d, *J* = 15.6 Hz, 1H), 4.15-4.03 (m, 2H), 2.30 (s, 3H); ^{13}C NMR (100 MHz, $CDCl_3$) δ 169.5, 153.2, 150.0, 137.2, 135.0, 130.1, 129.7, 128.8, 127.8, 127.5, 127.4 (d, J_{C-F} = 2.1 Hz), 126.4, 125.7, 124.0 (d, J_{C-F} = 6.2 Hz), 123.3, 122.0, 115.1 (t, J_{C-F} = 241.3 Hz), 109.8, 68.1 (dd, J_{C-F} = 6.5, 3.5 Hz), 53.1, 50.1 (t, J_{C-F} = 21.9 Hz), 20.9; ^{19}F NMR (376 MHz, $CDCl_3$) δ -115.9 (d, *J* = 286.5 Hz), -121.2 (d, *J* = 286.5 Hz). FT-IR: ν (cm^{-1}) 2919, 1493, 1311, 906, 758, 726. HRMS [ESI] calcd for $C_{24}H_{21}F_2N_2S$ $[M+H]^+$ 407.1388, found 407.1389.

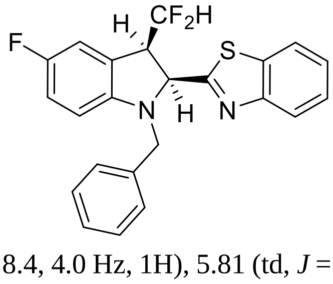

2-(1-benzyl-3-(difluoromethyl)-5-fluoroindolin-2-yl)benzo[*d*]thiazole (3l): Colorless oil. Purification by flash column chromatography (eluent: EtOAc/Petroleum ether = 1/20). ^{1}H NMR (400 MHz, $CDCl_3$) δ 8.04 (d, *J* = 8.0 Hz, 1H), 7.87 (d, *J* = 8.4 Hz, 1H), 7.54-7.48 (m, 1H), 7.46-7.40 (m, 1H), 7.38-7.28 (m, 5H), 7.08-7.03 (m, 1H), 6.87 (td, *J* = 8.8, 2.8 Hz, 1H), 6.44 (dd, *J* = 8.4, 4.0 Hz, 1H), 5.81 (td, *J* = 55.6, 5.6 Hz, 1H), 5.38 (d, *J* = 9.6 Hz, 1H), 4.51 (d, *J* = 15.6 Hz, 1H), 4.17-4.05 (m, 2H); ^{13}C NMR (100 MHz, $CDCl_3$) δ 168.8, 157.6 (d, $J_{C\text{-}F}$ = 236.8 Hz), 153.3, 148.4, 136.8, 135.0, 128.9, 127.8, 127.7, 126.5, 125.8, 125.2 (t, $J_{C\text{-}F}$ = 7.1 Hz), 123.4, 122.0, 115.9 (d, $J_{C\text{-}F}$ = 22.7 Hz), 115.1 (t, $J_{C\text{-}F}$ = 241.6 Hz), 114.3 (dd, $J_{C\text{-}F}$ = 24.6, 2.4 Hz), 110.1 (d, $J_{C\text{-}F}$ = 8.2 Hz), 68.2 (dd, $J_{C\text{-}F}$ = 5.9, 3.5 Hz), 53.2, 50.0 (t, $J_{C\text{-}F}$ = 21.9 Hz); ^{19}F NMR (376 MHz, $CDCl_3$) δ -116.3 (d, *J* = 288.0 Hz), -121.5 (d, *J* = 288.0 Hz), -124.3 (s). FT-IR: ν (cm^{-1}) 2970, 1486, 1226, 1008, 760, 727. HRMS [ESI] calcd for $C_{23}H_{18}F_3N_2S$ $[M+H]^+$ 411.1137, found 411.1138.

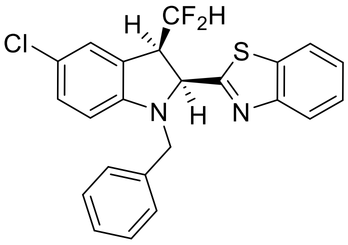

2-(1-benzyl-5-chloro-3-(difluoromethyl)indolin-2yl)benzo[*d*]thiazole (3m): Colorless oil. Purification by flash column chromatography (eluent: EtOAc/Petroleum ether = 1/20). ^{1}H NMR (400 MHz, $CDCl_3$) δ 8.04 (d, *J* = 8.4 Hz, 1H), 7.87 (d, *J* = 8.0 Hz, 1H), 7.54-7.48 (m, 1H), 7.46-7.40 (m, 1H), 7.36-7.26 (m, 6H), 7.16-7.12 (m, 1H), 6.47 (d, *J* = 8.4 Hz, 1H), 5.80 (td, *J* = 55.2, 5.6 Hz, 1H), 5.38 (d, *J* = 9.6 Hz, 1H), 4.53 (d, *J* = 15.6 Hz, 1H), 4.18-4.06 (m, 2H); ^{13}C NMR (100 MHz, $CDCl_3$) δ 168.3, 153.2, 150.7, 136.4, 135.0, 129.6, 128.9, 127.8, 127.8, 126.8 (d, $J_{C\text{-}F}$ = 2.7 Hz), 126.5, 125.9, 125.4 (d, $J_{C\text{-}F}$ = 6.4 Hz), 124.8, 123.5, 122.0, 114.8 (t, $J_{C\text{-}F}$ = 241.4 Hz), 110.3, 67.6 (dd, $J_{C\text{-}F}$ = 6.4, 3.5 Hz), 52.3, 49.9 (t, $J_{C\text{-}F}$ = 22.2 Hz); ^{19}F NMR (376 MHz, $CDCl_3$) δ -116.0 (d, *J* = 288.0 Hz), -121.2 (d, *J* = 288.0 Hz). FT-IR: ν (cm^{-1}) 2970, 1479, 1256, 1054, 759, 727. HRMS [ESI] calcd for $C_{23}H_{18}ClF_2N_2S$ $[M+H]^+$ 427.0842, found 427.0851.

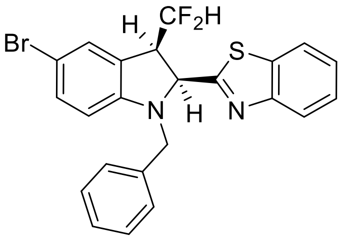

2-(1-benzyl-5-bromo-3-(difluoromethyl)indolin-2-yl)benzo[*d*]thiazole (3n): Colorless oil. Purification by flash column chromatography (eluent: EtOAc/Petroleum ether = 1/20). ^{1}H NMR (400 MHz, $CDCl_3$) δ 8.04 (d, *J* = 8.4 Hz, 1H), 7.87 (d, *J* = 7.6 Hz, 1H), 7.524-7.49 (m, 1H), 7.45-7.39 (m, 2H), 7.36-7.27 (m, 6H), 6.44 (d, *J* = 8.4 Hz, 1H), 5.80 (td, *J* = 55.2, 5.6 Hz, 1H), 5.38 (d, *J* = 9.6 Hz, 1H), 4.53 (d, *J* = 16.0 Hz, 1H), 4.18-4.06 (m, 2H); ^{13}C NMR (100 MHz, $CDCl_3$) δ 168.2, 153.2, 151.2, 136.4, 135.0, 132.5, 129.5 (d, $J_{C\text{-}F}$ = 2.7 Hz), 128.9, 127.8, 127.8, 126.5, 125.9, 125.9 (d, $J_{C\text{-}F}$ = 6.4 Hz), 123.5, 122.0, 114.8 (t, $J_{C\text{-}F}$ = 241.3 Hz), 111.7, 110.8, 67.4 (dd, $J_{C\text{-}F}$ = 6.3, 3.7 Hz), 52.1, 49.8 (t, $J_{C\text{-}F}$ = 22.3 Hz); ^{19}F NMR (376 MHz, $CDCl_3$) δ -116.0 (d, *J* = 289.1 Hz), -121.1 (d, *J* = 289.1 Hz). FT-IR: ν (cm^{-1}) 2925, 1507, 1376, 1058, 760, 728. HRMS [ESI] calcd for $C_{23}H_{18}BrF_2N_2S$ $[M+H]^+$ 471.0337, found 471.0327.

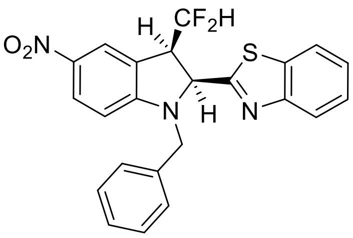

2-(1-benzyl-3-(difluoromethyl)-5-nitroindolin-2-yl)benzo[*d*]thiazole (3o): Colorless oil. Purification by flash column chromatography (eluent: EtOAc/Petroleum ether = 1/20). ^{1}H NMR (400 MHz, $CDCl_3$) δ 8.21 (dd, *J* = 8.8, 2.4 Hz, 1H), 8.18 (s, 1H), 8.05 (d, *J* = 8.0 Hz, 1H), 7.89 (d, *J* = 7.6 Hz, 1H), 7.56-7.51 (m, 1H), 7.48-7.44 (m, 1H), 7.37-7.31 (m, 3H), 7.22-7.18 (m, 2H), 6.60 (d, *J* = 8.8 Hz, 1H), 5.85 (td, *J* = 55.2, 6.0 Hz, 1H), 5.52 (d, *J* = 10.0 Hz, 1H), 4.70 (d, *J* = 15.6 Hz, 1H), 4.30-4.15 (m, 2H); ^{13}C NMR (100 MHz, $CDCl_3$) δ 166.1, 156.6, 153.2, 140.4, 135.1, 135.0, 129.2, 128.4, 127.8, 127.8, 126.8, 126.3, 123.9 (d, J_{C-F} = 6.4 Hz), 123.8, 122.8 (d, J_{C-F} = 3.1 Hz), 122.1, 114.7 (t, J_{C-F} = 241.3 Hz), 106.5, 66.2 (dd, J_{C-F} = 7.2, 3.2 Hz), 50.1, 49.1 (t, J_{C-F} = 22.9 Hz); ^{19}F NMR (376 MHz, $CDCl_3$) δ -115.1 (d, *J* = 292.5 Hz), -120.8 (d, *J* = 292.5 Hz). FT-IR: ν (cm^{-1}) 2925, 1604, 1483, 1059, 996, 748. HRMS [ESI] calcd for $C_{23}H_{18}F_2N_3O_2S$ $[M+H]^+$ 438.1082, found 438.1081

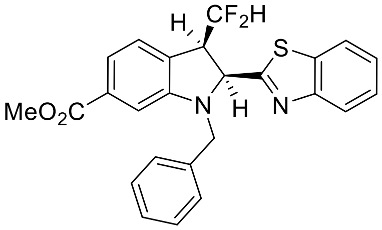

methyl 2-(benzo[*d*]thiazol-2-yl)-1-benzyl-3-(difluoromethyl)indoline-6-carboxylate (3p): Colorless oil. Purification by flash column chromatography (eluent: EtOAc/Petroleum ether = 1/20). ^{1}H NMR (400 MHz, $CDCl_3$) δ 8.04 (d, *J* = 8.0 Hz, 1H), 7.86 (d, *J* = 7.6 Hz, 1H), 7.56 (dd, *J* = 7.6, 1.6 Hz, 1H), 7.54-7.48 (m, 1H), 7.46-7.40 (m, 1H), 7.38-7.22 (m, 7H), 5.80 (td, *J* = 55.2, 5.2 Hz, 1H), 5.40 (d, *J* = 10.0 Hz, 1H), 4.68 (d, *J* = 15.6 Hz, 1H), 4.24-4.10 (m, 2H), 3.89 (s, 3H); ^{13}C NMR (100 MHz, $CDCl_3$) δ 168.2, 167.2, 153.2, 152.1, 136.1, 135.0, 131.8, 128.9, 128.7 (d, J_{C-F} = 6.3 Hz), 128.1, 127.9, 126.5, 126.3 (d, J_{C-F} = 2.7 Hz), 125.9, 123.5, 122.0, 121.7, 114.9 (t, J_{C-F} = 241.4 Hz), 109.4, 66.7 (dd, J_{C-F} = 6.4, 3.5 Hz), 52.3, 51.3, 50.0 (t, J_{C-F} = 22.4 Hz); ^{19}F NMR (376 MHz, $CDCl_3$) δ -115.6 (d, *J* = 289.1 Hz), -120.9 (d, *J* = 289.1 Hz). FT-IR: ν (cm^{-1}) 2934, 1521, 1389, 1211, 794, 758. HRMS [ESI] calcd for $C_{25}H_{21}F_2N_2O_2S$ $[M+H]^+$ 451.1286, found 451.1282.

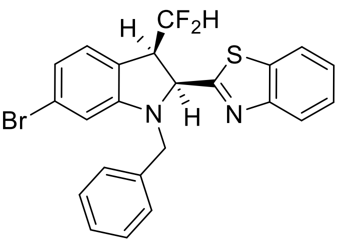

2-(1-benzyl-6-bromo-3-(difluoromethyl)indolin-2-yl)benzo[*d*]thiazole (3q): Colorless oil. Purification by flash column chromatography (eluent: EtOAc/Petroleum ether = 1/20). ^{1}H NMR (400 MHz, $CDCl_3$) δ 8.04 (d, *J* = 8.4 Hz, 1H), 7.87 (d, *J* = 8.0 Hz, 1H), 7.55-7.49 (m, 1H), 7.45-7.41 (m, 1H), 7.37-7.29 (m, 3H), 7.28-7.25 (m, 2H), 7.14 (d, *J* = 7.6 Hz, 1H), 6.97 (dd, *J* = 8.0, 1.6 Hz, 1H), 6.72 (d, *J* = 2.0 Hz, 1H), 5.78 (td, *J* = 55.2, 5.6 Hz, 1H), 5.39 (d, *J* = 9.6 Hz, 1H), 4.54 (d, *J* = 16.0 Hz, 1H), 4.15-4.02 (m, 2H); ^{13}C NMR (125 MHz, $CDCl_3$) δ 168.1, 153.3, 153.1, 136.1, 135.0, 129.0, 127.9, 127.8, 127.6 (d, J_{C-F} = 2.5 Hz), 126.5, 125.9, 123.7, 123.5, 122.7, 122.7 (d, J_{C-F} = 6.3 Hz), 122.0, 114.8 (t, J_{C-F} = 241.3 Hz), 112.3, 67.3 (dd, J_{C-F} = 6.9, 3.5 Hz), 51.6, 49.6 (t, J_{C-F} = 22.1 Hz); ^{19}F NMR (376 MHz, $CDCl_3$) δ -115.8 (d, *J* = 288.4 Hz), -121.2 (d, *J* = 288.4 Hz). FT-IR: ν (cm^{-1}) 2912, 1478, 1323, 1056, 782, 728. HRMS [ESI] calcd for $C_{23}H_{18}BrF_2N_2S$ $[M+H]^+$ 471.0337, found 471.0330.

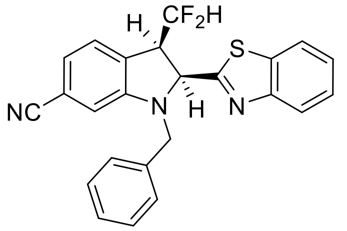

2-(benzo[*d*]thiazol-2-yl)-1-benzyl-3-(difluoromethyl)indoline-6-carbonitrile (3r): White solid, m.p. 112-113 °C. Purification by flash column chromatography (eluent: EtOAc/Petroleum ether = 1/20). ^{1}H NMR (400 MHz, $CDCl_3$) δ 8.04 (d, *J* = 8.0 Hz, 1H), 7.88 (d, *J* = 7.6 Hz, 1H), 7.55-7.50 (m, 1H), 7.47-7.42 (m, 1H), 7.38-7.31 (m, 4H), 7.28-7.25 (m, 2H), 7.14 (dd, *J* = 7.6, 1.2 Hz, 1H), 6.73 (d, *J* = 1.2 Hz, 1H), 5.81 (td, *J* = 55.2, 5.2 Hz, 1H), 5.45 (d, *J* = 10.0 Hz, 1H), 4.56 (d, *J* = 15.6 Hz, 1H), 4.26-4.13 (m, 2H); ^{13}C NMR (125 MHz, $CDCl_3$) δ 167.3, 153.2, 152.3, 135.6, 134.9, 129.1, 129.0 (d, $J_{C\text{-}F}$ = 6.1 Hz), 128.1, 127.7, 127.0 (d, $J_{C\text{-}F}$ = 1.6 Hz), 126.7, 126.1, 124.2, 123.5, 122.1, 119.3, 114.8 (t, $J_{C\text{-}F}$ = 241.5 Hz), 113.3, 111.2, 66.9 (dd, $J_{C\text{-}F}$ = 5.5, 4.1 Hz), 51.6, 49.9 (t, $J_{C\text{-}F}$ = 22.5 Hz); ^{19}F NMR (376 MHz, $CDCl_3$) δ -115.9 (d, *J* = 291.0 Hz), -121.1 (d, *J* = 291.0 Hz). FT-IR: ν (cm^{-1}) 2922, 1493, 1276, 1055, 761, 730. HRMS [ESI] calcd for $C_{24}H_{18}F_2N_3S$ $[M+H]^+$ 418.1184, found 418.1185.

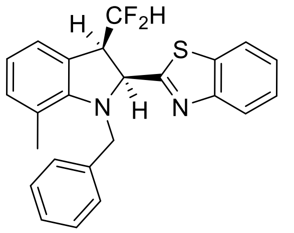

2-(1-benzyl-3-(difluoromethyl)-7-methylindolin-2-yl)benzo[*d*]thiazole (3s): Colorless oil. Purification by flash column chromatography (eluent: EtOAc/Petroleum ether = 1/20). ^{1}H NMR (400 MHz, $CDCl_3$) δ 7.96 (d, *J* = 8.4 Hz, 1H), 7.83 (d, *J* = 8.0 Hz, 1H), 7.49-7.43 (m, 1H), 7.39-7.33 (m, 1H), 7.27-7.23 (m, 3H), 7.22-7.18 (m, 2H), 7.16 (d, *J* = 7.6 Hz, 1H), 7.11 (d, *J* = 7.6 Hz, 1H), 6.90 (t, *J* = 7.6 Hz, 1H), 6.01 (td, *J* = 55.6, 5.2 Hz, 1H), 5.17 (d, *J* = 9.2 Hz, 1H), 4.73 (d, *J* = 15.6 Hz, 1H), 4.30 (d, *J* = 15.2 Hz, 1H), 4.24-4.13 (m, 1H), 2.53 (s, 3H); ^{13}C NMR (100 MHz, $CDCl_3$) δ 172.8, 153.7, 150.2, 137.5, 135.3, 132.3, 128.8, 128.3, 127.9, 126.7 (d, $J_{C\text{-}F}$ = 6.3 Hz), 126.0, 125.2, 123.8 (d, $J_{C\text{-}F}$ = 3.6 Hz), 123.4, 123.2, 122.5, 121.8, 115.9 (t, $J_{C\text{-}F}$ = 239.7 Hz), 65.1 (dd, $J_{C\text{-}F}$ = 6.2, 3.6 Hz), 54.7, 50.4 (t, $J_{C\text{-}F}$ = 21.3 Hz), 19.0; ^{19}F NMR (376 MHz, $CDCl_3$) δ -114.0 (d, *J* = 288.0 Hz), -121.3 (d, *J* = 288.0 Hz). FT-IR: ν (cm^{-1}) 2921, 1468, 1312, 1049, 907, 727. HRMS [ESI] calcd for $C_{24}H_{21}F_2N_2S$ $[M+H]^+$ 407.1388, found 407.1384.

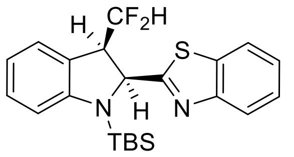

2-(1-(*tert*-butyldimethylsilyl)-3-(difluoromethyl)indolin-2-yl)benzo[*d*]thiazole (3t): Colorless oil. Purification by flash column chromatography (eluent: EtOAc/Petroleum ether = 1/20). ^{1}H NMR (400 MHz, $CDCl_3$) δ 8.00 (d, *J* = 8.0 Hz, 1H), 7.75 (d, *J* = 7.6 Hz, 1H), 7.48-7.42 (m, 1H), 7.38-7.32 (m, 1H), 7.28-7.24 (m, 1H), 7.21-7.15 (m, 1H), 6.96 (d, *J* = 8.0 Hz, 1H), 6.81 (td, *J* = 7.6, 1.2 Hz, 1H), 5.84 (td, *J* = 55.6, 6.4 Hz, 1H), 5.48 (d, *J* = 8.8 Hz, 1H), 4.40-4.30 (m, 1H), 0.91 (s, 9H), 0.52 (s, 3H), 0.10 (s, 3H); ^{13}C NMR (100 MHz, $CDCl_3$) δ 174.0, 153.1, 152.1, 134.9, 129.1, 126.1, 125.6 (d, $J_{C\text{-}F}$ = 4.7 Hz), 125.5, 125.2 (d, $J_{C\text{-}F}$ = 7.9 Hz), 123.4, 121.8, 119.8, 116.3 (t, $J_{C\text{-}F}$ = 238.9 Hz), 112.0, 64.7 (dd, $J_{C\text{-}F}$ = 8.7, 2.3 Hz), 52.3 (dd, $J_{C\text{-}F}$ = 23.6, 21.3 Hz), 26.7, 20.6, -3.2, -4.7; ^{19}F NMR (376 MHz, $CDCl_3$) δ -111.5 (d, *J* = 289.9 Hz), -120.1 (d, *J* = 289.9 Hz). FT-IR: ν (cm^{-1}) 2952, 1474, 1254, 1044, 809, 670. HRMS [ESI] calcd for $C_{22}H_{26}F_2N_2SSiNa$ $[M+Na]^+$ 439.1446, found 439.1454.

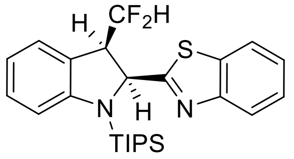

2-(3-(difluoromethyl)-1-(triisopropylsilyl)indolin-2-yl)benzo[*d*]thiazole (3u): Colorless oil. Purification by flash column chromatography (eluent: EtOAc/Petroleum ether = 1/20). ^{1}H NMR (400

MHz, $CDCl_3$) δ 8.00 (d, J = 8.4 Hz, 1H), 7.72 (d, J = 8.0 Hz, 1H), 7.48-7.42 (m, 1H), 7.37-7.32 (m, 1H), 7.28 (d, J = 6.8 Hz, 1H), 7.23-7.17 (m, 1H), 6.97 (d, J = 8.0 Hz, 1H), 6.89-6.84 (m, 1H), 5.72 (dt, J = 55.6, 7.2 Hz, 1H), 5.54 (d, J = 8.8 Hz, 1H), 5.40-5.30 (m, 1H), 1.50-1.41 (m, 3H), 1.15 (d, J = 7.6 Hz, 9H), 0.90 (d, J = 7.6 Hz, 9H); ^{13}C NMR (100 MHz, $CDCl_3$) δ 171.8, 152.2, 152.1, 134.6, 129.2, 126.1, 126.1 (d, $J_{C\text{-}F}$ = 9.0 Hz), 125.7, 125.5 (d, $J_{C\text{-}F}$ = 4.7 Hz), 123.4, 121.8, 120.2, 116.7 (t, $J_{C\text{-}F}$ = 238.9 Hz), 113.1, 65.1 (d, $J_{C\text{-}F}$ = 9.8 Hz), 52.7 (dd, $J_{C\text{-}F}$ = 23.6, 21.2 Hz), 18.6, 18.2, 12.8; ^{19}F NMR (376 MHz, $CDCl_3$) δ -111.4 (d, J = 295.9 Hz), -118.8 (d, J = 295.9 Hz). FT-IR: ν (cm^{-1}) 2946, 1599, 1458, 1100, 908, 729. HRMS [ESI] calcd for $C_{25}H_{33}F_2N_2SSi$ $[M+H]^+$ 459.2096, found 459.2103.

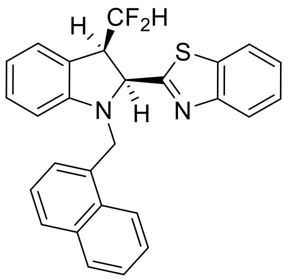

2-(3-(difluoromethyl)-1-(naphthalen-1-ylmethyl)indolin-2-yl)benzo[*d*]thiazole (3v): Colorless oil. Purification by flash column chromatography (eluent: EtOAc/Petroleum ether = 1/20). ^1H NMR (400 MHz, $CDCl_3$) δ 8.05 (d, J = 8.0 Hz, 1H), 7.87-7.78 (m, 5H), 7.54-7.45 (m, 4H), 7.44-7.39 (m, 1H), 7.34 (d, J = 7.6 Hz, 1H), 7.20-7.15 (m, 1H), 6.91-6.86 (m, 1H), 6.60 (d, J = 8.0 Hz, 1H), 5.87 (td, J = 55.2, 5.6 Hz, 1H), 5.44 (d, J = 9.6 Hz, 1H), 4.69 (d, J = 16.0 Hz, 1H), 4.31 (d, J = 16.0 Hz, 1H), 4.23-4.12 (m, 1H); ^{13}C NMR (100 MHz, $CDCl_3$) δ 169.0, 153.3, 152.3, 135.0, 134.6, 133.6, 133.0, 129.8, 128.6, 128.0, 127.8, 126.7 (d, $J_{C\text{-}F}$ = 2.3 Hz), 126.5, 126.4, 126.4, 126.0, 125.8, 125.7, 123.8 (d, $J_{C\text{-}F}$ = 6.3 Hz), 123.4, 122.0, 120.2, 115.1 (t, $J_{C\text{-}F}$ = 241.2 Hz), 109.8, 68.1 (dd, $J_{C\text{-}F}$ = 6.6, 3.4 Hz), 53.0, 50.2 (t, $J_{C\text{-}F}$ = 21.8 Hz); ^{19}F NMR (376 MHz, $CDCl_3$) δ -115.7 (d, J = 287.6 Hz), -121.1 (d, J = 287.6 Hz). FT-IR: ν (cm^{-1}) 2971, 1433, 1241, 1066, 759, 746. HRMS [ESI] calcd for $C_{27}H_{21}F_2N_2S$ $[M+H]^+$ 443.1388, found 443.1381.

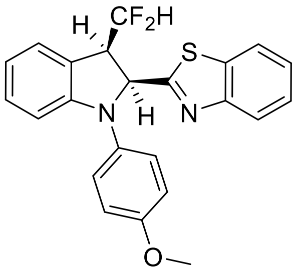

2-(3-(difluoromethyl)-1-(4-methoxyphenyl)indolin-2-yl)benzo[*d*]thiazole (3w): Colorless oil. Purification by flash column chromatography (eluent: EtOAc/Petroleum ether = 1/20). ^1H NMR (400 MHz, $CDCl_3$) δ 8.01 (d, J = 8.0 Hz, 1H), 7.80 (d, J = 8.0 Hz, 1H), 7.50-7.44 (m, 1H), 7.40-7.34 (m, 2H), 7.26-7.18 (m, 3H), 6.93-6.88 (m, 1H), 6.87-6.83 (m, 2H), 6.75 (d, J = 8.0 Hz, 1H), 6.01-5.71 (m, 2H), 4.37-4.25 (m, 1H), 3.75 (s, 3H); ^{13}C NMR (100 MHz, $CDCl_3$) δ 169.7, 157.1, 152.9, 151.0, 135.8, 135.2, 129.6, 126.9 (d, $J_{C\text{-}F}$ = 2.6 Hz), 126.3, 125.6, 124.9, 123.5 (d, $J_{C\text{-}F}$ = 5.9 Hz), 123.3, 122.0, 120.5, 114.9 (t, $J_{C\text{-}F}$ = 241.2 Hz), 114.9, 109.5, 68.0 (dd, $J_{C\text{-}F}$ = 6.5, 3.5 Hz), 55.5, 50.2 (t, $J_{C\text{-}F}$ = 22.2 Hz); ^{19}F NMR (376 MHz, $CDCl_3$) δ -115.6 (d, J = 288.0 Hz), -121.5 (d, J = 288.0 Hz). FT-IR: ν (cm^{-1}) 2958, 1546, 1247, 1048, 794, 728. HRMS [ESI] calcd for $C_{23}H_{19}F_2N_2OS$ $[M+H]^+$ 409.1181, found 409.1182.

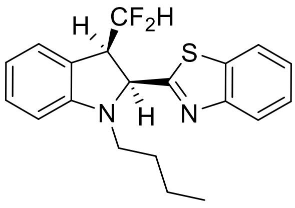

2-(1-butyl-3-(difluoromethyl)indolin-2-yl)benzo[*d*]thiazole (3x): Colorless oil. Purification by flash column chromatography (eluent: EtOAc/Petroleum ether = 1/20). ^1H NMR (400 MHz, $CDCl_3$) δ 8.03 (d, J = 8.0 Hz, 1H), 7.85 (d, J = 8.4 Hz, 1H), 7.52-7.47 (m, 1H), 7.43-7.38 (m, 1H), 7.29-7.23 (m, 2H), 6.82 (t, J = 7.6 Hz, 1H), 6.65 (d, J = 8.0 Hz, 1H), 5.75 (td, J = 55.6, 6.0 Hz, 1H), 5.40 (d, J = 10.0 Hz, 1H), 4.19-4.07 (m, 1H), 3.35-3.25 (m, 1H), 3.05-2.95 (m, 1H), 1.63-1.54 (m, 2H), 1.34-1.25 (m, 2H),

0.89 (t, J = 7.2 Hz, 3H); ^{13}C NMR (100 MHz, $CDCl_3$) δ 169.6, 153.1, 151.9, 135.0, 129.8, 126.5 (d, $J_{C\text{-}F}$ = 2.9 Hz), 126.4, 125.7, 123.5 (d, $J_{C\text{-}F}$ = 7.0 Hz), 123.3, 122.0, 119.4, 115.3 (t, $J_{C\text{-}F}$ = 241.0 Hz), 108.6, 66.9 (dd, $J_{C\text{-}F}$ = 7.0, 2.8 Hz), 50.2 (t, $J_{C\text{-}F}$ = 21.9 Hz), 47.2, 28.1, 20.5, 14.0; ^{19}F NMR (376 MHz, $CDCl_3$) δ -115.2 (d, J = 288.4 Hz), -120.8 (d, J = 288.4 Hz). FT-IR: ν (cm^{-1}) 2921, 1483, 1313, 1054, 906, 726. HRMS [ESI] calcd for $C_{20}H_{21}F_2N_2S$ [M+H]$^+$ 359.1388, found 359.1377.

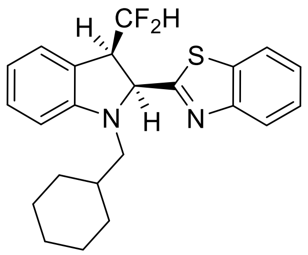

2-(1-(cyclohexylmethyl)-3-(difluoromethyl)indolin-2-yl)benzo[*d*]thiazole (3y): Colorless oil. Purification by flash column chromatography (eluent: EtOAc/Petroleum ether = 1/20). ^{1}H NMR (400 MHz, $CDCl_3$) δ 8.04 (d, J = 8.0 Hz, 1H), 7.79 (d, J = 8.0 Hz, 1H), 7.52-7.46 (m, 1H), 7.42-7.37 (m, 1H), 7.29-7.22 (m, 2H), 6.81-6.76 (m, 1H), 6.60 (d, J = 8.0 Hz, 1H), 5.78 (td, J = 55.6, 6.4 Hz, 1H), 5.40 (d, J = 9.2 Hz, 1H), 4.30-4.18 (m, 1H), 3.07 (dd, J = 14.4, 7.2 Hz, 1H), 2.72 (dd, J = 14.0, 6.8 Hz, 1H), 1.80-1.65 (m, 5H), 1.30-1.10 (m, 4H), 0.98-0.84 (m, 2H); ^{13}C NMR (100 MHz, $CDCl_3$) δ 168.5, 152.7, 152.0, 134.8, 129.6, 126.3, 125.9 (d, $J_{C\text{-}F}$ = 3.4 Hz), 125.8, 123.5, 122.8 (d, $J_{C\text{-}F}$ = 8.5 Hz), 121.9, 118.6, 115.8 (t, $J_{C\text{-}F}$ = 240.0 Hz), 107.5, 67.7 (dd, $J_{C\text{-}F}$ = 8.8, 2.2 Hz), 53.2, 50.4 (t, $J_{C\text{-}F}$ = 22.5 Hz), 36.8, 31.7, 31.5, 26.6, 26.0, 26.0; ^{19}F NMR (376 MHz, $CDCl_3$) δ -113.3 (d, J = 293.3 Hz), -119.5 (d, J = 293.3 Hz). FT-IR: ν (cm^{-1}) 2922, 1605, 1133, 1048, 756, 726. HRMS [ESI] calcd for $C_{23}H_{25}F_2N_2S$ [M+H]$^+$ 399.1701, found 399.1693.

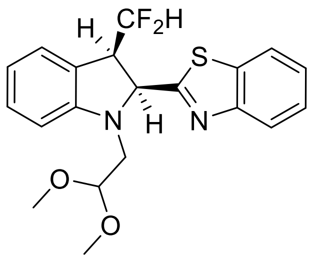

2-(3-(difluoromethyl)-1-(2,2-dimethoxyethyl)indolin-2-yl)benzo[*d*]thiazole (3z): Colorless oil. Purification by flash column chromatography (eluent: EtOAc/Petroleum ether = 1/20). ^{1}H NMR (500 MHz, $CDCl_3$) δ 8.04 (d, J = 8.0 Hz, 1H), 7.84 (d, J = 7.5 Hz, 1H), 7.52-7.48 (m, 1H), 7.43-7.39 (m, 1H), 7.31-7.28 (m, 1H), 7.27-7.25 (m, 1H), 6.86-6.82 (m, 1H), 6.75 (d, J = 8.0 Hz, 1H), 5.77 (td, J = 55.5, 6.0 Hz, 1H), 5.62 (d, J = 9.5 Hz, 1H), 4.61 (dd, J = 6.5, 3.5 Hz, 1H), 4.24-4.14 (m, 1H), 3.42 (dd, J = 15.5, 4.0 Hz, 1H), 3.35 (s, 3H), 3.31 (s, 3H), 3.21 (dd, J = 15.0, 6.5 Hz, 1H); ^{13}C NMR (125 MHz, $CDCl_3$) δ 169.2, 153.1, 151.4, 134.9, 129.7, 126.4 (d, $J_{C\text{-}F}$ = 2.8 Hz), 126.4, 125.7, 123.4, 123.1 (d, $J_{C\text{-}F}$ = 7.1 Hz), 121.9, 119.5, 115.3 (t, $J_{C\text{-}F}$ = 240.5 Hz), 108.3, 102.6, 67.3 (dd, $J_{C\text{-}F}$ = 7.3, 2.9 Hz), 54.6, 53.9, 50.2 (t, $J_{C\text{-}F}$ = 22.1 Hz), 49.1; ^{19}F NMR (470 MHz, $CDCl_3$) δ -114.9 (d, J = 289.5 Hz), -120.7 (d, J = 289.5 Hz). FT-IR: ν (cm^{-1}) 2934, 1484, 1243, 1047, 758, 728. HRMS [ESI] calcd for $C_{20}H_{21}F_2N_2O_2S$ [M+H]$^+$ 391.1286, found 391.1289.

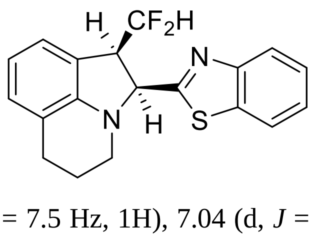

2-(1-(difluoromethyl)-1,2,5,6-tetrahydro-4H-pyrrolo[3,2,1-ij]quinolin-2-yl)benzo[*d*]thiazole (3aa): Colorless oil. Purification by flash column chromatography (eluent: EtOAc/Petroleum ether = 1/20). ^{1}H NMR (500 MHz, $CDCl_3$) δ 8.04 (d, J = 8.0 Hz, 1H), 7.90 (d, J = 7.5 Hz, 1H), 7.53-7.49 (m, 1H), 7.44-7.40 (m, 1H), 7.14 (d, J = 7.5 Hz, 1H), 7.04 (d, J = 7.5 Hz, 1H), 6.82 (t, J = 7.5 Hz, 1H), 5.78 (td, J = 55.5, 5.5 Hz, 1H), 5.14 (d, J = 9.5 Hz, 1H), 4.08-3.98 (m, 1H), 3.40-3.35 (m, 1H), 2.84-2.71 (m, 3H), 2.29-2.19 (m, 1H), 2.15-2.08 (m, 1H); ^{13}C NMR (125 MHz, $CDCl_3$) δ 169.8, 153.5, 149.5, 135.1, 128.8, 126.4, 125.6, 124.3, 123.2, 122.5 (d, $J_{C\text{-}F}$ = 6.4 Hz), 122.0, 121.1, 120.6, 114.8 (t, $J_{C\text{-}F}$ = 241.5 Hz), 69.5 (dd, $J_{C\text{-}F}$ = 6.8, 3.1 Hz), 50.4 (t, $J_{C\text{-}F}$ = 21.9 Hz), 46.4, 23.8, 23.0; ^{19}F NMR (470 MHz, $CDCl_3$) δ -

115.9 (d, J = 285.8 Hz), -121.1 (d, J = 285.8 Hz). FT-IR: ν (cm^{-1}) 2927, 1598, 1418, 1205, 752, 687. HRMS [ESI] calcd for $C_{19}H_{17}F_2N_2S$ [M+H]$^+$ 343.1075, found 343.1065.

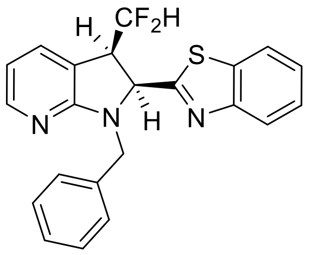

2-(1-benzyl-3-(difluoromethyl)-2,3-dihydro-1*H*-pyrrolo[2,3-*b*]pyridin-2-yl)benzo[*d*]thiazole (3ab): Colorless oil. Purification by flash column chromatography (eluent: EtOAc/Petroleum ether = 1/20). ^{1}H NMR (400 MHz, $CDCl_3$) δ 8.17-8.14 (m, 1H), 8.04 (d, J = 8.0 Hz, 1H), 7.89 (d, J = 8.0 Hz, 1H), 7.55-7.50 (m, 1H), 7.49-7.42 (m, 2H), 7.27-7.25 (m, 3H), 7.18-7.14 (m, 2H), 6.69 (dd, J = 7.2, 5.2 Hz, 1H), 5.77 (td, J = 55.2, 5.6 Hz, 1H), 5.38 (d, J = 15.2 Hz, 1H), 5.30 (d, J = 10.0 Hz, 1H), 4.13-3.99 (m, 2H); ^{13}C NMR (100 MHz, $CDCl_3$) δ 169.1, 163.5, 154.3, 149.4, 137.2, 136.2, 135.0 (d, J_{C-F} = 2.6 Hz), 129.9, 129.8, 128.8, 127.5, 126.9, 124.6, 123.0, 117.8 (d, J_{C-F} = 7.0 Hz), 116.6 (t, J_{C-F} = 240.8 Hz), 115.5, 63.0 (dd, J_{C-F} = 7.0, 3.3 Hz), 48.8 (t, J_{C-F} = 22.5 Hz), 48.4; ^{19}F NMR (376 MHz, $CDCl_3$) δ -115.3 (d, J = 289.5 Hz), -121.2 (d, J = 289.5 Hz). FT-IR: ν (cm^{-1}) 2923, 1603, 1483, 1048, 756, 728. HRMS [ESI] calcd for $C_{22}H_{18}F_2N_3S$ [M+H]$^+$ 394.1184, found 394.1176.

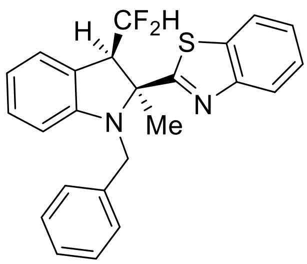

2-(1-benzyl-3-(difluoromethyl)-2-methylindolin-2-yl)benzo[*d*]thiazole (3ac): Colorless oil. Purification by flash column chromatography (eluent: EtOAc/Petroleum ether = 1/20). ^{1}H NMR (400 MHz, $CDCl_3$) δ 8.07 (d, J = 8.0 Hz, 1H), 7.79 (d, J = 8.0 Hz, 1H), 7.52-7.47 (m, 1H), 7.44-7.34 (m, 5H), 7.33-7.27 (m, 2H), 7.14 (t, J = 7.6 Hz, 1H), 6.83 (t, J = 7.2 Hz, 1H), 6.31 (d, J = 8.0 Hz, 1H), 5.76 (td, J = 55.6, 6.4 Hz, 1H), 4.27 (d, J = 16.4 Hz, 1H), 4.11 (d, J = 16.4 Hz, 1H), 4.00-3.90 (m, 1H), 2.09 (s, 3H); ^{13}C NMR (100 MHz, $CDCl_3$) δ 171.4, 152.8, 151.2, 138.2, 135.0, 129.6, 128.8, 127.2, 126.7, 126.3, 125.9 (d, J_{C-F} = 3.4 Hz), 125.8, 123.5, 123.2 (d, J_{C-F} = 8.4 Hz), 121.8, 119.5, 115.7 (t, J_{C-F} = 239.9 Hz), 108.9, 73.7 (dd, J_{C-F} = 8.9, 1.2 Hz), 57.8 (t, J_{C-F} = 21.3 Hz), 49.7, 24.4; ^{19}F NMR (376 MHz, $CDCl_3$) δ -115.4 (d, J = 293.3 Hz), -118.8 (d, J = 293.3 Hz). FT-IR: ν (cm^{-1}) 2983, 1645, 1482, 1048, 905, 724. HRMS [ESI] calcd for $C_{24}H_{21}F_2N_2S$ [M+H]$^+$ 407.1388, found 407.1389.

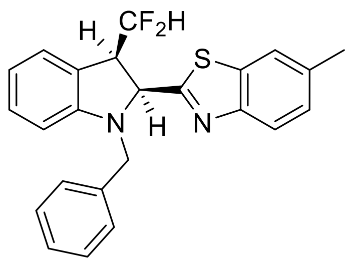

2-(1-benzyl-3-(difluoromethyl)indolin-2-yl)-6-methylbenzo[*d*]thiazole (3ad): Colorless oil. Purification by flash column chromatography (eluent: EtOAc/Petroleum ether = 1/20). ^{1}H NMR (500 MHz, $CDCl_3$) δ 7.92 (d, J = 8.0 Hz, 1H), 7.64 (s, 1H), 7.36-7.26 (m, 7H), 7.19 (t, J = 7.5 Hz, 1H), 6.86 (t, J = 7.5 Hz, 1H), 6.56 (d, J = 8.0 Hz, 1H), 5.82 (td, J = 55.5, 5.5 Hz, 1H), 5.36 (d, J = 9.5 Hz, 1H), 4.55 (d, J = 16.0 Hz, 1H), 4.17-4.08 (m, 2H), 2.49 (s, 3H); ^{13}C NMR (125 MHz, $CDCl_3$) δ 167.8, 152.2, 151.3, 137.0, 136.0, 135.2, 129.7, 128.8, 128.0, 127.8, 127.6, 126.6 (d, J_{C-F} = 2.4 Hz), 123.8 (d, J_{C-F} = 6.4 Hz), 122.8, 121.7, 120.0, 115.1 (t, J_{C-F} = 241.1 Hz), 109.7, 67.7 (dd, J_{C-F} = 6.4, 2.8 Hz), 52.5, 50.0 (t, J_{C-F} = 21.9 Hz), 21.7; ^{19}F NMR (470 MHz, $CDCl_3$) δ -115.7 (d, J = 287.2 Hz), -121.1 (d, J = 287.2 Hz). FT-IR: ν (cm^{-1}) 2921, 1603, 1482, 1241, 1043, 815, 729. HRMS [ESI] calcd for $C_{24}H_{21}F_2N_2S$ [M+H]$^+$ 407.1388, found 407.1392.

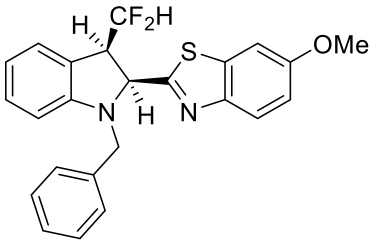

2-(1-benzyl-3-(difluoromethyl)indolin-2-yl)-6-methoxybenzo[*d*]thiazole (3ae): Colorless oil. Purification by flash column chromatography (eluent: EtOAc/Petroleum ether = 1/20). ^{1}H NMR (400 MHz, $CDCl_3$) δ 7.91 (d, *J* = 9.2 Hz, 1H), 7.35-7.28 (m, 7H), 7.21-7.16 (m, 1H), 7.10 (dd, *J* = 8.8, 2.4 Hz, 1H), 6.88-6.83 (m, 1H), 6.55 (d, *J* = 8.0 Hz, 1H), 5.83 (td, *J* = 55.6, 5.6 Hz, 1H), 5.34 (d, *J* = 9.6 Hz, 1H), 4.54 (d, *J* = 16.0 Hz, 1H), 4.16-4.04 (m, 2H), 3.87 (s, 3H); ^{13}C NMR (100 MHz, $CDCl_3$) δ 166.2, 158.2, 152.2, 147.7, 137.0, 136.4, 129.7, 128.8, 127.8, 127.6, 126.6 (d, $J_{C\text{-}F}$ = 2.5 Hz), 123.8, 123.8 (d, $J_{C\text{-}F}$ = 2.5 Hz), 120.0, 115.9, 115.5 (t, $J_{C\text{-}F}$ = 241.2 Hz), 109.7, 104.3, 67.7 (dd, $J_{C\text{-}F}$ = 7.0, 3.2 Hz), 55.9, 52.5, 50.1 (t, $J_{C\text{-}F}$ = 22.2 Hz); ^{19}F NMR (376 MHz, $CDCl_3$) δ -115.7 (d, *J* = 287.3 Hz), -121.1 (d, *J* = 287.3 Hz). FT-IR: ν (cm^{-1}) 2919, 1603, 1259, 1056, 752, 730. HRMS [ESI] calcd for $C_{24}H_{21}F_2N_2OS$ $[M+H]^+$ 423.1337, found 423.1334.

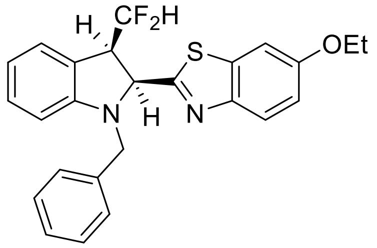

2-(1-benzyl-3-(difluoromethyl)indolin-2-yl)-6-ethoxybenzo[*d*]thiazole (3af): Colorless oil. Purification by flash column chromatography (eluent: EtOAc/Petroleum ether = 1/20). ^{1}H NMR (500 MHz, $CDCl_3$) δ 7.90 (d, *J* = 9.0 Hz, 1H), 7.35-7.27 (m, 7H), 7.18 (t, *J* = 8.0 Hz, 1H), 7.09 (dd, *J* = 9.0, 2.5 Hz, 1H), 6.86 (t, *J* = 7.5 Hz, 1H), 6.55 (d, *J* = 8.0 Hz, 1H), 5.82 (td, *J* = 55.5, 6.0 Hz, 1H), 5.33 (d, *J* = 9.5 Hz, 1H), 4.54 (d, *J* = 15.5 Hz, 1H), 4.15-4.05 (m, 4H), 1.45 (t, *J* = 7.0 Hz, 3H); ^{13}C NMR (125 MHz, $CDCl_3$) δ 166.0, 157.5, 152.2, 147.6, 137.0, 136.4, 129.7, 128.8, 127.8, 127.6, 126.6 (d, $J_{C\text{-}F}$ = 1.5 Hz), 123.8, 123.8 (d, $J_{C\text{-}F}$ = 7.0 Hz), 120.0, 116.3, 115.7 (t, $J_{C\text{-}F}$ = 241.1 Hz), 109.7, 104.9, 67.7 (dd, $J_{C\text{-}F}$ = 6.5, 2.6 Hz), 64.2, 52.5, 50.0 (t, $J_{C\text{-}F}$ = 21.9 Hz), 14.9; ^{19}F NMR (376 MHz, $CDCl_3$) δ -115.8 (d, *J* = 287.6 Hz), -121.1 (d, *J* = 287.6 Hz). FT-IR: ν (cm^{-1}) 2965, 1719, 1271, 1110, 841, 710. HRMS [ESI] calcd for $C_{25}H_{23}F_2N_2OS$ $[M+H]^+$ 437.1494, found 437.1501.

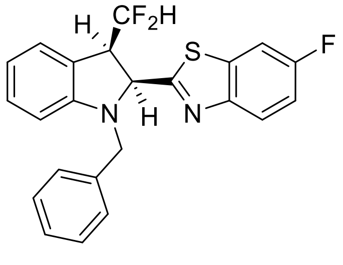

2-(1-benzyl-3-(difluoromethyl)indolin-2-yl)-6-fluorobenzo[*d*]thiazole (3ag): Colorless oil. Purification by flash column chromatography (eluent: EtOAc/Petroleum ether = 1/20). ^{1}H NMR (500 MHz, $CDCl_3$) δ 7.98 (dd, *J* = 9.0, 5.0 Hz, 1H), 7.55-7.53 (m, 1H), 7.36-7.28 (m, 6H), 7.25-7.17 (m, 2H), 6.87 (t, *J* = 7.5 Hz, 1H), 6.57 (d, *J* = 8.0 Hz, 1H), 5.81 (td, *J* = 55.5, 6.0 Hz, 1H), 5.34 (d, *J* = 9.5 Hz, 1H), 4.55 (d, *J* = 15.5 Hz, 1H), 4.17-4.08 (m, 2H); ^{13}C NMR (125 MHz, $CDCl_3$) δ 168.9 (d, $J_{C\text{-}F}$ = 3.5 Hz), 160.8 (d, $J_{C\text{-}F}$ = 245.1 Hz), 152.1, 149.9, 136.8, 136.1 (d, $J_{C\text{-}F}$ = 10.9 Hz), 129.8, 128.9, 127.8, 127.7, 126.7 (d, $J_{C\text{-}F}$ = 2.0 Hz), 124.3 (d, $J_{C\text{-}F}$ = 9.6 Hz), 123.7 (d, $J_{C\text{-}F}$ = 6.3 Hz), 120.2, 115.6 (t, $J_{C\text{-}F}$ = 241.1 Hz), 115.2 (d, $J_{C\text{-}F}$ = 24.6 Hz), 109.8, 108.2 (d, $J_{C\text{-}F}$ = 26.5 Hz), 67.6 (dd, $J_{C\text{-}F}$ = 6.4, 2.8 Hz), 52.6, 50.1 (t, $J_{C\text{-}F}$ = 22.0 Hz); ^{19}F NMR (470 MHz, $CDCl_3$) δ -115.1 (s), -115.8 (d, *J* = 287.6 Hz), -121.1 (d, *J* = 287.6 Hz). FT-IR: ν (cm^{-1}) 2921, 1654, 1484, 1048, 756, 726. HRMS [ESI] calcd for $C_{23}H_{18}F_3N_2S$ $[M+H]^+$ 411.1137, found 411.1132.

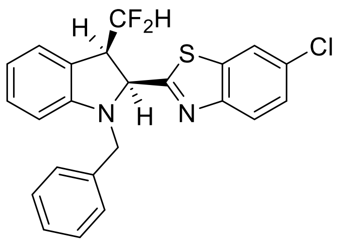

2-(1-benzyl-3-(difluoromethyl)indolin-2-yl)-6-chlorobenzo[*d*]thiazole (3ah): Colorless oil. Purification by flash column chromatography (eluent: EtOAc/Petroleum ether = 1/20). ^{1}H NMR (400 MHz, $CDCl_3$) δ 7.94 (d, *J* = 8.8 Hz, 1H), 7.83 (d, *J* = 2.0 Hz, 1H), 7.46 (dd, *J* = 8.8, 2.0 Hz, 1H), 7.36-7.28 (m, 6H), 7.22-7.17 (m, 1H), 6.90-6.85 (m, 1H), 6.58 (d, *J* = 8.0 Hz, 1H), 5.80 (td, *J* = 55.2, 5.6 Hz, 1H), 5.34 (d, *J* = 9.6 Hz, 1H), 4.55 (d, *J* = 15.6 Hz, 1H), 4.18-4.06 (m, 2H); ^{13}C NMR (100 MHz, $CDCl_3$) δ 169.9, 152.0, 151.8, 136.8, 136.3, 131.8, 129.8, 128.9, 127.8, 127.7, 127.3, 126.7 (d, $J_{C\text{-}F}$ = 2.2 Hz), 124.1, 123.6 (d, $J_{C\text{-}F}$ = 6.3 Hz), 121.6, 120.3, 115.0 (t, $J_{C\text{-}F}$ = 241.6 Hz), 109.8, 67.6 (dd, $J_{C\text{-}F}$ = 6.5, 2.9 Hz), 52.7, 50.1 (t, $J_{C\text{-}F}$ = 21.8 Hz); ^{19}F NMR (470 MHz, $CDCl_3$) δ -115.8 (d, *J* = 288.0 Hz), -121.1 (d, *J* = 288.0 Hz). FT-IR: ν (cm^{-1}) 2981, 1535, 1483, 1239, 1044, 747. HRMS [ESI] calcd for $C_{23}H_{18}ClF_2N_2S$ $[M+H]^+$ 427.0842, found 427.0843.

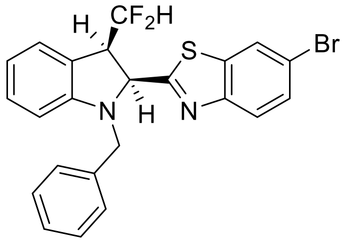

2-(1-benzyl-3-(difluoromethyl)indolin-2-yl)-6-bromobenzo[*d*]thiazole (3ai): Colorless oil. Purification by flash column chromatography (eluent: EtOAc/Petroleum ether = 1/20). ^{1}H NMR (500 MHz, $CDCl_3$) δ 7.99 (s, 1H), 7.88 (d, *J* = 8.5 Hz, 1H), 7.60 (d, *J* = 8.5 Hz, 1H), 7.35-7.28 (m, 6H), 7.19 (t, *J* = 8.0 Hz, 1H), 6.87 (t, *J* = 7.5 Hz, 1H), 6.58 (d, *J* = 8.0 Hz, 1H), 5.80 (td, *J* = 55.5, 5.5 Hz, 1H), 5.34 (d, *J* = 9.5 Hz, 1H), 4.54 (d, *J* = 15.5 Hz, 1H), 4.17-4.08 (m, 2H); ^{13}C NMR (125 MHz, $CDCl_3$) δ 170.0, 152.1, 152.0, 136.7, 136.7, 130.0, 129.8, 128.9, 127.8, 127.7, 126.7 (d, $J_{C\text{-}F}$ = 1.4 Hz), 124.6, 124.5, 123.6 (d, $J_{C\text{-}F}$ = 6.1 Hz), 120.3, 119.5, 115.0 (t, $J_{C\text{-}F}$ = 241.1 Hz), 109.8, 67.6 (dd, $J_{C\text{-}F}$ = 6.4, 2.6 Hz), 52.7, 50.1 (t, $J_{C\text{-}F}$ = 21.8 Hz); ^{19}F NMR (470 MHz, $CDCl_3$) δ -115.9 (d, *J* = 287.6 Hz), -121.1 (d, *J* = 287.6 Hz). FT-IR: ν (cm^{-1}) 2927, 1554, 1476, 1303, 858, 750. HRMS [ESI] calcd for $C_{23}H_{18}BrF_2N_2S$ $[M+H]^+$ 471.0337, found 471.0332.

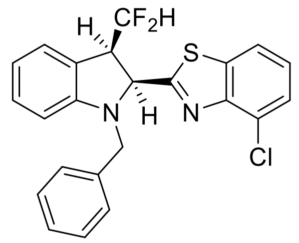

2-(1-benzyl-3-(difluoromethyl)indolin-2-yl)-4-chlorobenzo[*d*]thiazole (3aj): Colorless oil. Purification by flash column chromatography (eluent: EtOAc/Petroleum ether = 1/20). ^{1}H NMR (500 MHz, $CDCl_3$) δ 7.75 (d, *J* = 8.0 Hz, 1H), 7.52 (d, *J* = 8.0 Hz, 1H), 7.38-7.29 (m, 7H), 7.20-7.16 (m, 1H), 6.90-6.86 (m, 1H), 6.54 (d, *J* = 8.0 Hz, 1H), 5.83 (td, *J* = 55.0, 5.0 Hz, 1H), 5.48 (d, *J* = 10.0 Hz, 1H), 4.54 (d, *J* = 16.0 Hz, 1H), 4.23-4.14 (m, 2H); ^{13}C NMR (125 MHz, $CDCl_3$) δ 171.1, 152.3, 150.4, 137.0, 136.6, 129.8, 128.9, 128.0, 127.6, 126.8, 126.7, 126.2, 123.7 (d, $J_{C\text{-}F}$ = 4.6 Hz), 120.6, 120.4, 114.9 (t, $J_{C\text{-}F}$ = 241.4 Hz), 110.1, 68.0 (dd, $J_{C\text{-}F}$ = 5.5, 3.8 Hz), 53.3, 50.0 (t, $J_{C\text{-}F}$ = 21.9 Hz); ^{19}F NMR (470 MHz, $CDCl_3$) δ -116.6 (d, *J* = 287.2 Hz), -121.6 (d, *J* = 287.2 Hz). FT-IR: ν (cm^{-1}) 2997, 1598, 1388, 1205, 906, 752. HRMS [ESI] calcd for $C_{23}H_{18}ClF_2N_2S$ $[M+H]^+$ 427.0842, found 427.0839.

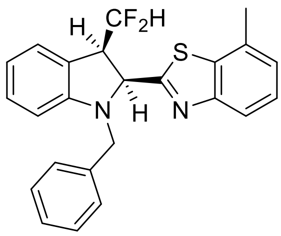

2-(1-benzyl-3-(difluoromethyl)indolin-2-yl)-7-methylbenzo[*d*]thiazole (3ak): Colorless oil. Purification by flash column chromatography (eluent: EtOAc/Petroleum ether = 1/20). ^{1}H NMR (400 MHz, CDCl$_3$) δ 7.72-7.66 (m, 1H), 7.38-7.28 (m, 8H), 7.23-7.16 (m, 1H), 6.89-6.84 (m, 1H), 6.54 (d, *J* = 8.0 Hz, 1H), 5.84 (td, *J* = 55.6, 5.6 Hz, 1H), 5.42 (d, *J* = 9.6 Hz, 1H), 4.54 (d, *J* = 15.6 Hz, 1H), 4.22-4.11 (m, 2H), 2.76 (s, 3H); ^{13}C NMR (100 MHz, CDCl$_3$) δ 167.6, 152.7, 152.3, 137.2, 134.9, 133.4, 129.7, 128.8, 127.7, 127.5, 126.9, 126.6 (d, $J_{C\text{-}F}$ = 2.5 Hz), 125.6, 123.8 (d, $J_{C\text{-}F}$ = 6.1 Hz), 120.0, 119.4, 115.2 (t, $J_{C\text{-}F}$ = 241.2 Hz), 109.6, 68.0 (dd, $J_{C\text{-}F}$ = 6.5, 3.5 Hz), 52.6, 50.1 (t, $J_{C\text{-}F}$ = 22.2 Hz), 18.6; ^{19}F NMR (376 MHz, CDCl$_3$) δ -115.7 (d, *J* = 286.5 Hz), -121.2 (d, *J* = 286.5 Hz). FT-IR: ν (cm-1) 2956, 1645, 1433, 1231, 1045, 782. HRMS [ESI] calcd for $C_{24}H_{21}F_2N_2S$ $[M+H]^+$ 407.1388, found 407.1386.

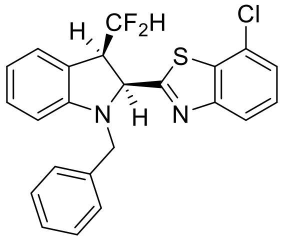

2-(1-benzyl-3-(difluoromethyl)indolin-2-yl)-7-chlorobenzo[*d*]thiazole (3al): Colorless oil. Purification by flash column chromatography (eluent: EtOAc/Petroleum ether = 1/20). ^{1}H NMR (500 MHz, CDCl$_3$) δ 7.94 (d, *J* = 8.0 Hz, 1H), 7.46 (t, *J* = 8.0 Hz, 1H), 7.43-7.40 (m, 1H), 7.35-7.28 (m, 6H), 7.23-7.19 (m, 1H), 6.90-6.86 (m, 1H), 6.62 (d, *J* = 8.0 Hz, 1H), 5.84 (td, *J* = 55.5, 6.0 Hz, 1H), 5.35 (d, *J* = 10.0 Hz, 1H), 4.61 (d, *J* = 15.5 Hz, 1H), 4.20-4.10 (m, 2H); ^{13}C NMR (125 MHz, CDCl$_3$) δ 170.3, 153.9, 151.9, 136.6, 135.4, 129.9, 128.9, 127.9, 127.7, 127.4, 127.3, 126.7 (d, $J_{C\text{-}F}$ = 2.5 Hz), 125.4, 123.5 (d, $J_{C\text{-}F}$ = 6.5 Hz), 121.7, 120.2, 115.0 (t, $J_{C\text{-}F}$ = 241.3 Hz), 109.8, 67.3 (dd, $J_{C\text{-}F}$ = 7.3, 2.9 Hz), 52.4, 50.1 (t, $J_{C\text{-}F}$ = 22.1 Hz); ^{19}F NMR (470 MHz, CDCl$_3$) δ -115.5 (d, *J* = 287.2 Hz), -121.0 (d, *J* = 287.2 Hz). FT-IR: ν (cm^{-1}) 2907, 1598, 1344, 1205, 865, 752. HRMS [ESI] calcd for $C_{23}H_{18}ClF_2N_2S$ $[M+H]^+$ 427.0842, found 427.0839.

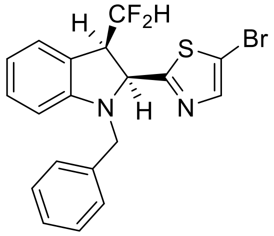

2-(1-benzyl-3-(difluoromethyl)indolin-2-yl)-5-bromothiazole (3am): Colorless oil. Purification by flash column chromatography (eluent: EtOAc/Petroleum ether = 1/20). ^{1}H NMR (400 MHz, CDCl$_3$) δ 7.69 (s, 1H), 7.36-7.28 (m, 6H), 7.20-7.15 (m, 1H), 6.87-6.85 (m, 1H), 6.54 (d, *J* = 8.0 Hz, 1H), 5.73 (td, *J* = 55.6, 5.6 Hz, 1H), 5.20 (d, *J* = 9.6 Hz, 1H), 4.50 (d, *J* = 15.6 Hz, 1H), 4.11-3.98 (m, 2H); ^{13}C NMR (100 MHz, CDCl$_3$) δ 169.6, 152.0, 144.4, 136.8, 129.8, 128.9, 127.8, 127.7, 126.6 (d, $J_{C\text{-}F}$ = 2.0 Hz), 123.6 (d, $J_{C\text{-}F}$ = 5.9 Hz), 120.2, 115.1 (t, $J_{C\text{-}F}$ = 240.9 Hz), 110.2, 109.7, 67.4 (dd, $J_{C\text{-}F}$ = 6.1, 3.2 Hz), 52.4, 49.9 (t, $J_{C\text{-}F}$ = 21.9 Hz); ^{19}F NMR (376 MHz, CDCl$_3$) δ -116.4 (d, *J* = 288.0 Hz), -121.5 (d, *J* = 288.0 Hz). FT-IR: ν (cm^{-1}) 2930, 1603, 1482, 1057, 997, 728. HRMS [ESI] calcd for $C_{19}H_{16}BrF_2N_2S$ $[M+H]^+$ 421.0180, found 421.0172.

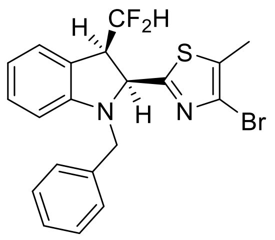

2-(1-benzyl-3-(difluoromethyl)indolin-2-yl)-4-bromo-5-methylthiazole (3an): Colorless oil. Purification by flash column chromatography (eluent: EtOAc/Petroleum ether = 1/20). ^{1}H NMR (500 MHz, $CDCl_3$) δ 7.37-7.25 (m, 6H), 7.18-7.13 (m, 1H), 6.86-6.82 (m, 1H), 6.51 (d, J = 8.0 Hz, 1H), 5.75 (td, J = 55.5, 5.5 Hz, 1H), 5.17 (d, J = 9.5 Hz, 1H), 4.49 (d, J = 16.0 Hz, 1H), 4.09 (d, J = 16.0 Hz, 1H), 4.05-3.95 (m, 1H), 2.41 (s, 3H); ^{13}C NMR (125 MHz, $CDCl_3$) δ 167.0, 152.1, 152.1, 136.9, 129.7, 128.9, 127.7, 127.6, 126.7 (d, J_{C-F} = 1.1 Hz), 123.7 (d, J_{C-F} = 5.9 Hz), 120.2, 115.1 (t, J_{C-F} = 241.1 Hz), 109.8, 105.6, 67.7 (dd, J_{C-F} = 5.5, 3.8 Hz), 52.6, 49.8 (t, J_{C-F} = 21.8 Hz), 15.9; ^{19}F NMR (470 MHz, $CDCl_3$) δ -116.5 (d, J = 287.2 Hz), -121.7 (d, J = 287.2 Hz). FT-IR: ν (cm^{-1}) 2979, 1550, 1483, 1241, 1044, 729. HRMS [ESI] calcd for $C_{20}H_{18}BrF_2N_2S$ $[M+H]^+$ 435.0337, found 435.0328.

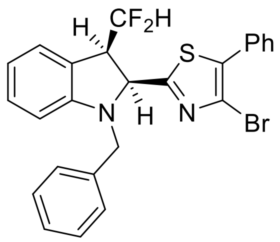

2-(1-benzyl-3-(difluoromethyl)indolin-2-yl)-4-bromo-5-phenylthiazole (3ao): Colorless oil. Purification by flash column chromatography (eluent: EtOAc/Petroleum ether = 1/20). ^{1}H NMR (500 MHz, $CDCl_3$) δ 7.93-7.90 (m, 2H), 7.49-7.45 (m, 2H), 7.43-7.39 (m, 1H), 7.37-7.29 (m, 6H), 7.20-7.16 (m, 1H), 6.89-6.84 (m, 1H), 6.55 (d, J = 8.0 Hz, 1H), 5.84 (td, J = 55.0, 5.0 Hz, 1H), 5.27 (d, J = 9.5 Hz, 1H), 4.56 (d, J = 15.5 Hz, 1H), 4.16 (d, J = 16.0 Hz, 1H), 4.13-4.04 (m, 1H); ^{13}C NMR (125 MHz, $CDCl_3$) δ 167.7, 152.9, 152.1, 136.9, 133.3, 129.7, 128.9, 128.8, 128.7, 128.5, 127.7, 127.7, 126.7 (d, J_{C-F} = 1.3 Hz), 123.7 (d, J_{C-F} = 5.4 Hz), 120.3, 115.1 (t, J_{C-F} = 241.3 Hz), 109.9, 104.9, 67.7 (dd, J_{C-F} = 5.5, 3.9 Hz), 52.8, 49.9 (t, J_{C-F} = 22.0 Hz); ^{19}F NMR (470 MHz, $CDCl_3$) δ -116.5 (d, J = 285.8 Hz), -121.7 (d, J = 285.8 Hz). FT-IR: ν (cm^{-1}) 2924, 1600, 1445, 1138, 769, 738. HRMS [ESI] calcd for $C_{25}H_{20}BrF_2N_2S$ $[M+H]^+$ 497.0493, found 497.0495.

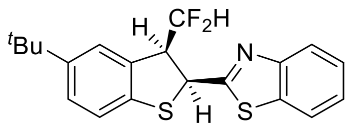

2-(5-(*tert*-butyl)-3-(difluoromethyl)-2,3-dihydrobenzo[*b*]thiophen-2-yl)benzo[*d*]thiazole (4a): Colorless oil. Purification by flash column chromatography (eluent: EtOAc/Petroleum ether = 1/20). ^{1}H NMR (500 MHz, $CDCl_3$) δ 8.04 (d, J = 8.0 Hz, 1H), 7.80 (d, J = 8.0 Hz, 1H), 7.50-7.46 (m, 1H), 7.40 (s, 1H), 7.39-7.35 (m, 1H), 7.33 (dd, J = 8.5 Hz, 1H), 7.22 (dd, J = 8.0 Hz, 1H), 5.88 (td, J = 56.0, 5.5 Hz, 1H), 5.21-5.19 (m, 1H), 4.56-4.48 (m, 1H), 1.30 (s, 9H); ^{13}C NMR (125 MHz, $CDCl_3$) δ 171.5, 153.2, 149.2, 137.8, 135.9, 135.5, 127.1, 126.4, 125.4, 123.5, 123.3, 122.4, 121.8, 115.7 (t, J_{C-F} = 244.1 Hz), 56.5 (t, J_{C-F} = 22.3 Hz), 52.4 (t, J_{C-F} = 3.6 Hz), 34.7, 31.5; ^{19}F NMR (376 MHz, $CDCl_3$) δ -114.9 (d, J = 293.7 Hz), -119.4 (d, J = 293.7 Hz). FT-IR: ν (cm^{-1}) 2969, 1437, 1243, 1057, 759, 728. HRMS [ESI] calcd for $C_{20}H_{20}F_2NS_2$ $[M+H]^+$ 376.1000, found 376.1001.

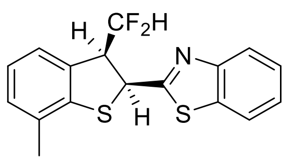

2-(3-(difluoromethyl)-7-methyl-2,3-dihydrobenzo[*b*]thiophen-2-yl)benzo[*d*]thiazole (4b): Colorless oil. Purification by flash column chromatography (eluent: EtOAc/Petroleum ether = 1/20). ^{1}H NMR (500 MHz, $CDCl_3$) δ 7.97 (d, J = 8.0 Hz, 1H), 7.77 (d, J = 8.0 Hz, 1H), 7.48-7.44 (m, 1H), 7.39-7.35 (m, 1H), 7.24 (d, J = 8.5 Hz, 1H), 7.16-7.12 (m, 2H), 6.18 (ddd, J = 56.5, 54.5, 6.0 Hz, 1H), 5.44 (d, J = 7.0 Hz, 1H), 4.38-4.29 (m, 1H), 2.31 (s,

3H); ^{13}C NMR (125 MHz, $CDCl_3$) δ 168.4, 152.5, 140.0, 135.1, 133.7 (d, J_{C-F} = 7.8 Hz), 132.9, 130.1, 126.4, 126.1, 125.9, 123.5, 123.3 (d, J_{C-F} = 5.1 Hz), 121.7, 115.7 (t, J_{C-F} = 241.4 Hz), 56.4 (t, J_{C-F} = 22.0 Hz), 51.5 (dd, J_{C-F} = 7.9, 1.9 Hz), 20.7; ^{19}F NMR (470 MHz, $CDCl_3$) δ -115.2 (d, J = 294.2 Hz), -119.3 (d, J = 294.2 Hz). FT-IR: ν (cm^{-1}) 2977, 1503, 1332, 1205, 991, 823. HRMS [ESI] calcd for $C_{17}H_{14}F_2NS_2$ [M+H]$^+$ 334.0530, found 334.0533.

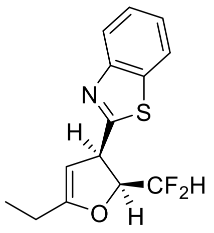

2-(2-(difluoromethyl)-5-ethyl-2,3-dihydrofuran-3-yl)benzo[*d*]thiazole (5a): Colorless oil. Purification by flash column chromatography (eluent: EtOAc/Petroleum ether = 1/20). ^{1}H NMR (500 MHz, CD_3CN) δ 8.02-7.97 (m, 2H), 7.55-7.51 (m, 1H), 7.47-7.43 (m, 1H), 5.97 (ddd, J = 55.5, 53.0, 5.0 Hz, 1H), 5.11-5.08 (m, 1H), 5.01-4.93 (m, 1H), 4.83-4.79 (m, 1H), 2.33 (q, J = 7.5 Hz, 2H), 1.20 (t, J = 7.5 Hz, 3H); ^{13}C NMR (125 MHz, CD_3CN) δ 172.5, 164.9, 154.6, 136.3, 127.1, 126.1, 123.7, 122.9, 114.8 (dd, J_{C-F} = 241.4, 236.8 Hz), 97.9, 82.5 (dd, J_{C-F} = 27.5, 20.3 Hz), 48.4 (dd, J_{C-F} = 4.4, 1.9 Hz), 21.7, 11.2; ^{19}F NMR (470 MHz, CD_3CN) δ -126.5 (d, J = 296.1 Hz), -128.8 (d, J = 296.1 Hz). FT-IR: ν (cm^{-1}) 2963, 1634, 1533, 1055, 907, 727. HRMS [ESI] calcd for $C_{14}H_{14}F_2NOS$ [M+H]$^+$ 282.0759, found 282.0763.

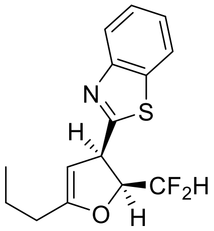

2-(2-(difluoromethyl)-5-propyl-2,3-dihydrofuran-3-yl)benzo[*d*]thiazole (5b): Colorless oil. Purification by flash column chromatography (eluent: EtOAc/Petroleum ether = 1/20). ^{1}H NMR (500 MHz, CD_3CN) δ 7.99-7.94 (m, 2H), 7.52-7.48 (m, 1H), 7.44-7.40 (m, 1H), 5.94 (ddd, J = 55.5, 53.0, 4.5 Hz, 1H), 5.09-5.06 (m, 1H), 4.98-4.89 (m, 1H), 4.80-4.76 (m, 1H), 2.28 (t, J = 7.5 Hz, 2H), 1.67-1.58 (m, 2H), 1.02 (t, J = 7.0 Hz, 3H); ^{13}C NMR (125 MHz, CD_3CN) δ 172.5, 163.3, 154.6, 136.3, 127.1, 126.1, 123.7, 122.9, 114.8 (dd, J_{C-F} = 242.2, 237.4 Hz), 98.9, 82.4 (dd, J_{C-F} = 28.1, 20.8 Hz), 48.5 (dd, J_{C-F} = 4.1, 2.4 Hz), 30.2, 20.5, 13.9; ^{19}F NMR (470 MHz, CD_3CN) δ -126.5 (d, J = 296.1 Hz), -128.9 (d, J = 296.1 Hz). FT-IR: ν (cm^{-1}) 2923, 1434, 1503, 1155, 897, 657. HRMS [ESI] calcd for $C_{15}H_{16}F_2NOS$ [M+H]$^+$ 296.0915, found 296.0920.

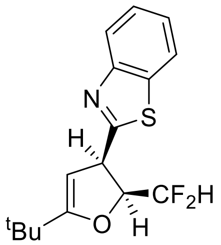

2-(5-(*tert*-butyl)-2-(difluoromethyl)-2,3-dihydrofuran-3-yl)benzo[*d*]thiazole (5c): Colorless oil. Purification by flash column chromatography (eluent: EtOAc/Petroleum ether = 1/20). ^{1}H NMR (500 MHz, CD_3CN) δ 7.98-7.93 (m, 2H), 7.52-7.48 (m, 1H), 7.44-7.40 (m, 1H), 5.88 (ddd, J = 55.0, 53.0, 5.0 Hz, 1H), 5.07-5.04 (m, 1H), 4.95-4.86 (m, 1H), 4.75 (dd, J = 9.5, 2.5 Hz, 1H), 1.22 (s, 9H); ^{13}C NMR (125 MHz, CD_3CN) δ 172.6, 171.2, 154.6, 136.3, 127.1, 126.1, 123.7, 122.9, 114.8 (dd, J_{C-F} = 241.6, 237.0 Hz), 96.3, 82.6 (dd, J_{C-F} = 27.6, 20.1 Hz), 48.4 (dd, J_{C-F} = 4.4, 1.6 Hz), 33.0, 27.9; ^{19}F NMR (470 MHz, CD_3CN) δ -126.4 (d, J = 296.6 Hz), -128.7 (d, J = 296.6 Hz). FT-IR: ν (cm^{-1}) 2909, 1540, 1397, 1041, 758, 728. HRMS [ESI] calcd for $C_{16}H_{18}F_2NOS$ [M+H]$^+$ 310.1072, found 310.1070.

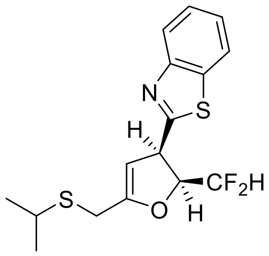

2-(2-(difluoromethyl)-5-((isopropylthio)methyl)-2,3-dihydrofuran-3-yl)benzo[*d*]thiazole (5d): Colorless oil. Purification by flash column chromatography (eluent: EtOAc/Petroleum ether = 1/20). ^{1}H NMR (500 MHz, CD_3CN) δ 7.98 (d, *J* = 8.0 Hz, 1H), 7.96 (d, *J* = 8.0 Hz, 1H), 7.53-7.49 (m, 1H), 7.45-7.41 (m, 1H), 5.97 (ddd, *J* = 55.0, 52.5, 5.0 Hz, 1H), 5.29-5.27 (m, 1H), 5.03-4.95 (m, 1H), 4.86-4.82 (m, 1H), 3.40 (s, 2H), 3.16-3.10 (m, 1H), 1.30 (d, *J* = 7.0 Hz, 6H); ^{13}C NMR (125 MHz, CD_3CN) δ 171.6, 159.8, 154.5, 136.2, 127.2, 126.2, 123.7, 123.0, 114.7 (dd, $J_{C\text{-}F}$ = 241.6, 237.0 Hz), 101.1, 82.8 (dd, $J_{C\text{-}F}$ = 28.1, 20.9 Hz), 48.5 (dd, $J_{C\text{-}F}$ = 4.3, 1.8 Hz), 35.6, 27.3, 23.3, 23.3; ^{19}F NMR (470 MHz, CD_3CN) δ -121.4 (d, *J* = 296.1 Hz), -123.6 (d, *J* = 296.1 Hz). FT-IR: ν (cm^{-1}) 2957, 1503, 1313, 1059, 766, 789. HRMS [ESI] calcd for $C_{16}H_{18}F_2NOS_2$ $[M+H]^+$ 342.0792, found 342.0782.

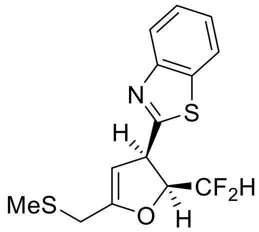

2-(2-(difluoromethyl)-5-((methylthio)methyl)-2,3-dihydrofuran-3-yl)benzo[*d*]thiazole (5e): Colorless oil. Purification by flash column chromatography (eluent: EtOAc/Petroleum ether = 1/20). ^{1}H NMR (400 MHz, $CDCl_3$) δ 8.01 (d, *J* = 8.0 Hz, 1H), 7.87 (d, *J* = 8.0 Hz, 1H), 7.51-7.46 (m, 1H), 7.42-7.37 (m, 1H), 5.88 (ddd, *J* = 55.6, 53.2, 5.2 Hz, 1H), 5.25-5.23 (m, 1H), 5.02-4.93 (m, 1H), 4.88-4.83 (m, 1H), 3.32 (s, 2H), 2.26 (s, 3H); ^{13}C NMR (100 MHz, $CDCl_3$) δ 169.8, 159.0, 153.7, 135.4, 126.4, 125.5, 123.4, 121.8, 113.2 (dd, $J_{C\text{-}F}$ = 244.0, 239.4 Hz), 100.2, 82.3 (dd, $J_{C\text{-}F}$ = 28.6, 21.1 Hz), 48.2 (dd, $J_{C\text{-}F}$ = 4.3, 1.1 Hz), 30.4, 16.2; ^{19}F NMR (376 MHz, $CDCl_3$) δ -125.8 (d, *J* = 299.3 Hz), -127.4 (d, *J* = 299.3 Hz). FT-IR: ν (cm^{-1}) 2917, 1543, 1411, 1205, 996, 752. HRMS [ESI] calcd for $C_{14}H_{14}F_2NOS_2$ $[M+H]^+$ 314.0479, found 314.0480.

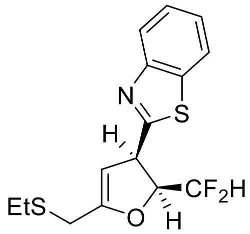

2-(2-(difluoromethyl)-5-((ethylthio)methyl)-2,3-dihydrofuran-3-yl)benzo[*d*]thiazole (5f): Colorless oil. Purification by flash column chromatography (eluent: EtOAc/Petroleum ether = 1/20). ^{1}H NMR (500 MHz, $CDCl_3$) δ 8.01 (d, *J* = 8.0 Hz, 1H), 7.87 (d, *J* = 8.0 Hz, 1H), 7.51-7.47 (m, 1H), 7.42-7.38 (m, 1H), 5.88 (ddd, *J* = 55.5, 53.5, 5.5 Hz, 1H), 5.32-5.26 (m, 1H), 5.01-4.94 (m, 1H), 4.88-4.82 (m, 1H), 4.19 (s, 2H), 3.65 (q, *J* = 7.0 Hz, 2H), 1.28 (t, *J* = 7.0 Hz, 3H); ^{13}C NMR (125 MHz, $CDCl_3$) δ 169.8, 159.2, 153.6, 135.4, 126.4, 125.5, 123.4, 121.8, 113.2 (dd, $J_{C\text{-}F}$ = 244.0, 239.4 Hz), 100.4, 82.3 (dd, $J_{C\text{-}F}$ = 28.4, 21.0 Hz), 67.0, 65.0, 47.9 (dd, $J_{C\text{-}F}$ = 4.6, 1.8 Hz), 15.3; ^{19}F NMR (376 MHz, $CDCl_3$) δ -125.7 (d, *J* = 299.4 Hz), -127.3 (d, *J* = 299.4 Hz). FT-IR: ν (cm^{-1}) 2916, 1508, 1446, 1205, 1007, 752. HRMS [ESI] calcd for $C_{15}H_{16}F_2NOS_2$ $[M+H]^+$ 328.0636, found 328.0640.

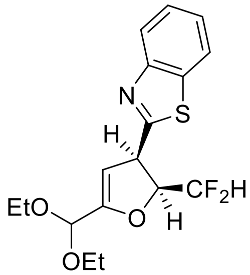

2-(5-(diethoxymethyl)-2-(difluoromethyl)-2,3-dihydrofuran-3-yl)benzo[*d*]thiazole (5g): Colorless oil. Purification by flash column chromatography (eluent: EtOAc/Petroleum ether = 1/20). ^{1}H NMR (500 MHz, $CDCl_3$) δ 8.00 (d, *J* = 8.5 Hz, 1H), 7.87 (d, *J* = 8.0 Hz, 1H), 7.51-7.47 (m, 1H), 7.42-7.38 (m, 1H), 5.90 (ddd, *J* = 55.0, 53.5, 5.5 Hz, 1H), 5.46 (s, 1H), 5.19-5.16 (m, 1H), 5.03-4.96 (m, 1H), 4.89-4.85 (m, 1H), 3.81-3.73 (m, 2H), 3.72-3.62 (m, 2H), 1.31-1.25 (m, 6H); ^{13}C NMR (125 MHz, $CDCl_3$) δ 169.5, 158.6, 153.6, 135.4, 126.4, 125.5, 123.4, 121.8,

113.1 (dd, J_{C-F} = 244.0, 239.3 Hz), 101.3, 95.8, 82.4 (dd, J_{C-F} = 28.9, 21.6 Hz), 62.2, 61.7, 47.7 (dd, J_{C-F} = 4.6, 1.9 Hz), 15.4, 15.3; ^{19}F NMR (470 MHz, $CDCl_3$) δ -125.8 (d, *J* = 298.5 Hz), -127.5 (d, *J* = 298.5 Hz). FT-IR: ν (cm^{-1}) 2925, 1545, 1321, 1023, 895, 761. HRMS [ESI] calcd for $C_{17}H_{20}F_2NO_3S$ [M+H]$^+$ 356.1126, found 356.1135.

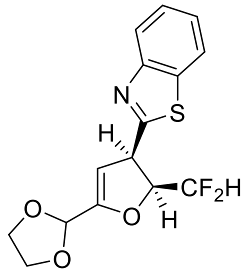

2-(2-(difluoromethyl)-5-(1,3-dioxolan-2-yl)-2,3-dihydrofuran-3-yl)benzo[*d*]thiazole (5h): Colorless oil. Purification by flash column chromatography (eluent: EtOAc/Petroleum ether = 1/20). ^{1}H NMR (500 MHz, $CDCl_3$) δ 8.01 (d, *J* = 8.5 Hz, 1H), 7.86 (d, *J* = 8.0 Hz, 1H), 7.51-7.47 (m, 1H), 7.42-7.38 (m, 1H), 5.87 (ddd, *J* = 55.5, 53.5, 5.5 Hz, 1H), 5.67 (s, 1H), 5.46-5.40 (m, 1H), 5.03-4.95 (m, 1H), 4.86 (dd, *J* = 10.5, 2.5 Hz, 1H), 4.17-4.11 (m, 2H), 4.06-4.01 (m, 2H); ^{13}C NMR (125 MHz, $CDCl_3$) δ 168.9, 158.5, 153.6, 135.4, 126.5, 125.6, 123.4, 121.8, 113.0 (dd, J_{C-F} = 244.0, 239.4 Hz), 101.7, 97.3, 82.7 (dd, J_{C-F} = 29.1, 21.9 Hz), 65.6, 65.5, 47.9 (dd, J_{C-F} = 4.5, 1.0 Hz); ^{19}F NMR (470 MHz, $CDCl_3$) δ -125.9 (d, *J* = 300.3 Hz), -127.0 (d, *J* = 300.3 Hz). FT-IR: ν (cm^{-1}) 2938, 1520, 1251, 1170, 861, 729. HRMS [ESI] calcd for $C_{15}H_{14}F_2NO_3S$ [M+H]$^+$ 326.0657, found 326.0659.

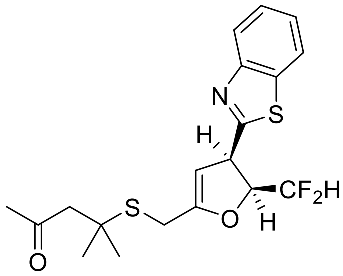

4-(((4-(benzo[*d*]thiazol-2-yl)-5-(difluoromethyl)-4,5-dihydrofuran-2-yl)methyl)thio)-4-methylpentan-2-one (5i): Colorless oil. Purification by flash column chromatography (eluent: EtOAc/Petroleum ether = 1/20). ^{1}H NMR (500 MHz, $CDCl_3$) δ 8.00 (d, *J* = 8.0 Hz, 1H), 7.87 (d, *J* = 8.0 Hz, 1H), 7.51-7.46 (m, 1H), 7.42-7.38 (m, 1H), 5.87 (ddd, *J* = 55.5, 53.5, 5.4 Hz, 1H), 5.26-5.24 (m, 1H), 5.00-4.92 (m, 1H), 4.86-4.81 (m, 1H), 3.47-3.40 (m, 2H), 2.80 (s, 2H), 2.19 (s, 3H), 1.50 (s, 6H); ^{13}C NMR (125 MHz, $CDCl_3$) δ 206.7, 169.8, 159.5, 153.6, 135.3, 126.4, 125.5, 123.4, 121.9, 113.2 (dd, J_{C-F} = 243.9, 238.9 Hz), 100.2, 82.3 (dd, J_{C-F} = 28.4, 21.0 Hz), 54.7, 48.1 (dd, J_{C-F} = 4.5, 1.9 Hz), 44.7, 32.3, 28.4, 28.4, 25.2; ^{19}F NMR (470 MHz, $CDCl_3$) δ -125.8 (d, *J* = 299.4 Hz), -127.5 (d, *J* = 299.4 Hz). FT-IR: ν (cm^{-1}) 2977, 1635, 1240, 1044, 761, 730. HRMS [ESI] calcd for $C_{19}H_{22}F_2NO_2S_2$ [M+H]$^+$ 398.1055, found 398.1061.

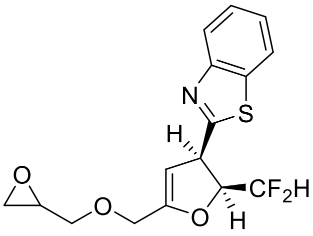

2-(2-(difluoromethyl)-5-((oxiran-2-ylmethoxy)methyl)-2,3-dihydrofuran-3-yl)benzo[*d*]thiazole (5j): Colorless oil. Purification by flash column chromatography (eluent: EtOAc/Petroleum ether = 1/20). 1H NMR (500 MHz, $CDCl_3$) δ 8.01 (d, *J* = 8.0 Hz, 1H), 7.87 (d, *J* = 8.0 Hz, 1H), 7.51-7.47 (m, 1H), 7.42-7.38 (m, 1H), 5.89 (ddd, *J* = 55.5, 53.0, 5.5 Hz, 1H), 5.35-5.31 (m, 1H), 5.02-4.92 (m, 1H), 4.88-4.84 (m, 1H), 4.32-4.22 (m, 2H), 3.92 (dt, *J* = 11.5, 3.0 Hz, 1H), 3.57-3.51 (m, 1H), 3.25-3.20 (m, 1H), 2.86-2.82 (m, 1H), 2.69-2.65 (m, 1H); ^{13}C NMR (125 MHz, $CDCl_3$) δ 169.5, 158.5, 153.6, 135.3, 126.4, 125.5, 123.4, 121.8, 113.2 (dd, J_{C-F} = 244.0, 238.8 Hz), 101.0, 82.3 (dd, J_{C-F} = 28.9, 21.1 Hz), 71.8, 65.6, 50.8, 47.9 (dd, J_{C-F} = 4.4, 1.0 Hz), 44.3; ^{19}F NMR (470 MHz, $CDCl_3$) δ -125.4 (d, *J* = 298.0 Hz), -127.4 (d, *J* = 298.0 Hz). FT-IR: ν (cm^{-1}) 2920, 1532, 1242, 1043, 760, 729. HRMS [ESI] calcd for $C_{16}H_{16}F_2NO_3S$ [M+H]$^+$ 340.0813, found 340.0820.

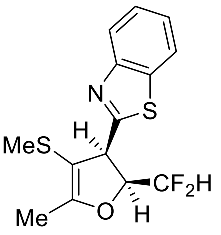

2-(2-(difluoromethyl)-5-methyl-4-(methylthio)-2,3-dihydrofuran-3-yl)benzo[*d*]thiazole (5k): Colorless oil. Purification by flash column chromatography (eluent: EtOAc/Petroleum ether = 1/20). ^{1}H NMR (400 MHz, $CDCl_3$) δ 8.03 (d, *J* = 8.0 Hz, 1H), 7.87 (d, *J* = 8.4 Hz, 1H), 7.51-7.46 (m, 1H), 7.42-7.37 (m, 1H), 5.92 (td, *J* = 54.8, 6.0 Hz, 1H), 4.88-4.79 (m, 1H), 4.77-4.72 (m, 1H), 2.12 (d, *J* = 1.2 Hz, 3H), 2.06 (s, 3H); ^{13}C NMR (100 MHz, $CDCl_3$) δ 168.7, 160.9, 153.6, 135.6, 126.3, 125.5, 123.5, 121.9, 113.5 (t, $J_{C\text{-}F}$ = 240.7 Hz), 106.0, 81.0 (dd, $J_{C\text{-}F}$ = 27.1, 26.1 Hz), 51.0 (t, $J_{C\text{-}F}$ = 2.9 Hz), 18.0, 12.5; ^{19}F NMR (376 MHz, $CDCl_3$) δ -125.9 (d, *J* = 303.4 Hz), -126.0 (d, *J* = 303.4 Hz). FT-IR: ν (cm^{-1}) 2941, 1532, 1453, 1156, 759, 696. HRMS [ESI] calcd for $C_{14}H_{14}F_2NOS_2$ $[M+H]^+$ 314.0479, found 314.0488.

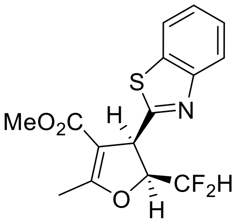

methyl 4-(benzo[*d*]thiazol-2-yl)-5-(difluoromethyl)-2-methyl-4,5-dihydrofuran-3-carboxylate (5l): Colorless oil. Purification by flash column chromatography (eluent: EtOAc/Petroleum ether = 1/20). ^{1}H NMR (500 MHz, $CDCl_3$) δ 8.01 (d, *J* = 8.0 Hz, 1H), 7.85 (d, *J* = 8.0 Hz, 1H), 7.50-7.46 (m, 1H), 7.41-7.37 (m, 1H), 5.91 (ddd, *J* = 56.0, 53.0, 4.5 Hz, 1H), 4.98-4.89 (m, 2H), 3.60 (s, 3H), 2.43 (s, 3H); ^{13}C NMR (125 MHz, $CDCl_3$) δ 170.8, 168.9, 164.7, 153.6, 135.4, 126.3, 125.5, 123.4, 121.8, 112.9 (dd, $J_{C\text{-}F}$ = 243.1, 239.3 Hz), 106.6, 82.2 (dd, $J_{C\text{-}F}$ = 29.8, 23.1 Hz), 51.5, 47.5 (dd, $J_{C\text{-}F}$ = 4.6, 1.9 Hz), 14.3; ^{19}F NMR (470 MHz, $CDCl_3$) δ -126.0 (d, *J* = 303.2 Hz), -126.9 (d, *J* = 303.2 Hz). FT-IR: ν (cm^{-1}) 2937, 1598, 1335, 1205, 954, 752. HRMS [ESI] calcd for $C_{15}H_{14}F_2NO_3S$ $[M+H]^+$ 326.0657, found 326.0660.

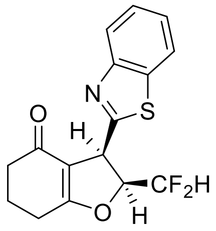

3-(benzo[*d*]thiazol-2-yl)-2-(difluoromethyl)-3,5,6,7-tetrahydrobenzofuran-4(2H)-one (5m): Colorless oil. Purification by flash column chromatography (eluent: EtOAc/Petroleum ether = 1/20). ^{1}H NMR (500 MHz, $CDCl_3$) δ 7.98 (d, *J* = 8.0 Hz, 1H), 7.83 (d, *J* = 7.5 Hz, 1H), 7.48-7.43 (m, 1H), 7.38-7.34 (m, 1H), 6.01 (td, *J* = 54.0, 2.5 Hz, 1H), 5.67-5.60 (m, 1H), 4.93-4.90 (m, 1H), 2.65-2.55 (m, 2H), 2.45-2.35 (m, 2H), 2.14-2.08 (m, 2H); ^{13}C NMR (125 MHz, $CDCl_3$) δ 194.2, 178.1, 169.6, 152.7, 135.9, 126.2, 125.4, 123.2, 121.8, 114.8, 114.2 (t, $J_{C\text{-}F}$ = 243.9 Hz), 86.1 (dd, $J_{C\text{-}F}$ = 26.3, 24.1 Hz), 43.9 (dd, $J_{C\text{-}F}$ = 3.5, 1.6 Hz), 36.7, 24.0, 21.5; ^{19}F NMR (470 MHz, $CDCl_3$) δ -131.3 (d, *J* = 294.7 Hz), -132.1 (d, *J* = 294.7 Hz). FT-IR: ν (cm^{-1}) 2955, 1504, 1345, 1117, 894, 658. HRMS [ESI] calcd for $C_{16}H_{14}F_2NO_2S$ $[M+H]^+$ 322.0708, found 322.0711.

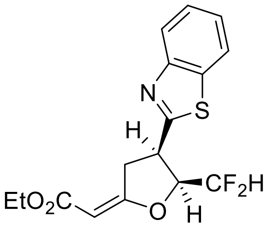

ethyl (*E*)-2-(4-(benzo[d]thiazol-2-yl)-5-(difluoromethyl)dihydrofuran-2(3*H*)-ylidene)acetate (5n): Colorless oil. Purification by flash column chromatography (eluent: EtOAc/Petroleum ether = 1/20). ^{1}H NMR (500 MHz, $CDCl_3$) δ 8.02 (d, *J* = 8.0 Hz, 1H), 7.87 (d, *J* = 8.0 Hz, 1H), 7.53-7.47 (m, 1H), 7.43-7.38 (m, 1H), 5.88 (ddd, *J* = 55.5, 53.5, 5.5 Hz, 1H), 5.33 (s, 1H), 5.02-4.94 (m, 1H), 4.90-4.85 (m, 1H), 4.81 (d, *J* = 14.0 Hz, 1H), 4.79 (d, *J* = 13.5 Hz, 1H), 2.44 (q, *J* = 7.5 Hz, 2H), 1.19 (t, *J* = 7.5 Hz, 3H); ^{13}C NMR (125 MHz, $CDCl_3$) δ 174.0, 169.1, 156.7, 153.6, 135.3, 126.5, 125.6, 123.4, 121.9, 113.1 (dd, $J_{C\text{-}F}$ = 244.0, 238.8 Hz), 101.6, 82.3 (dd, $J_{C\text{-}F}$ = 29.1, 21.1 Hz), 58.2, 47.9 (dd, $J_{C\text{-}F}$ = 4.6, 1,0 Hz), 27.5, 9.2; ^{19}F NMR (470 MHz, $CDCl_3$) δ -125.8 (d, *J* = 299.9

Hz), -127.4 (d, J = 299.9 Hz). FT-IR: ν (cm^{-1}) 2922, 1505, 1325, 1190, 934, 752. HRMS [ESI] calcd for $C_{16}H_{16}F_2NO_3S$ [M+H]$^+$ 340.0813, found 340.0816.

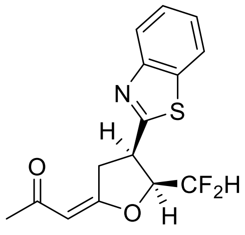

(*E*)-1-(4-(benzo[d]thiazol-2-yl)-5-(difluoromethyl)dihydrofuran-2(3*H*)-ylidene)propan-2-one (5o): White solid, m.p. 105-106 ºC. Purification by flash column chromatography (eluent: EtOAc/Petroleum ether = 1/20). ^{1}H NMR (500 MHz, $CDCl_3$) δ 8.00 (d, J = 8.0 Hz, 1H), 7.88 (d, J = 8.5 Hz, 1H), 7.53-7.48 (m, 1H), 7.44-7.40 (m, 1H), 6.10-5.86 (m, 2H), 4.95-4.80 (m, 1H), 4.33-4.27 (m, 1H), 3.77-3.63 (m, 2H), 2.20 (s, 3H); ^{13}C NMR (125 MHz, $CDCl_3$) δ 197.7, 172.3, 165.8, 153.2, 134.8, 126.7, 125.8, 123.4, 121.8, 113.5 (dd, J_{C-F} = 244.3, 240.0 Hz), 101.0, 81.3 (dd, J_{C-F} = 26.5, 21.0 Hz), 42.5 (t, J_{C-F} = 2.3 Hz), 36.7, 31.6; ^{19}F NMR (470 MHz, $CDCl_3$) δ -127.5 (d, J = 299.4 Hz), -128.9 (d, J = 299.4 Hz). FT-IR: ν (cm^{-1}) 3017, 1498, 1205, 1123, 845, 689. HRMS [ESI] calcd for $C_{15}H_{14}F_2NO_2S$ [M+H]$^+$ 310.0708, found 310.0710.

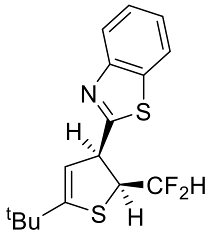

2-(5-(*tert*-butyl)-2-(difluoromethyl)-2,3-dihydrothiophen-3-yl)benzo[*d*]thiazole (6): Colorless oil. Purification by flash column chromatography (eluent: EtOAc/Petroleum ether = 1/20). ^{1}H NMR (500 MHz, $CDCl_3$) δ 8.01 (d, J = 8.0 Hz, 1H), 7.85 (d, J = 8.0 Hz, 1H), 7.50-7.46 (m, 1H), 7.40-7.36 (m, 1H), 5.83 (td, J = 56.5, 5.5 Hz, 1H), 5.30-5.10 (m, 1H), 4.76-4.74 (m, 1H), 4.24-4.17 (m, 1H), 1.26 (s, 9H); ^{13}C NMR (125 MHz, $CDCl_3$) δ 172.1, 158.2, 153.3, 135.2, 126.3, 125.3, 123.2, 121.8, 114.8 (t, J_{C-F} = 244.1 Hz), 114.0, 55.6 (t, J_{C-F} = 22.0 Hz), 52.1 (t, J_{C-F} = 3.8 Hz), 35.0, 30.1; ^{19}F NMR (470 MHz, $CDCl_3$) δ -120.1 (d, J = 276.8 Hz), -120.2 (d, J = 276.8 Hz). FT-IR: ν (cm^{-1}) 2965, 1586, 1458, 1120, 757, 728. HRMS [ESI] calcd for $C_{16}H_{18}F_2NS_2$ [M+H]$^+$ 326.0843, found 326.0834.

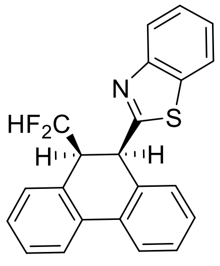

2-(10-(difluoromethyl)-9,10-dihydrophenanthren-9-yl)benzo[*d*]thiazole (7a): Colorless oil. Purification by flash column chromatography (eluent: EtOAc/Petroleum ether = 1/20). ^{1}H NMR (500 MHz, $CDCl_3$) δ 7.94 (d, J = 8.5 Hz, 1H), 7.87-7.82 (m, 2H), 7.58 (d, J = 8.0 Hz, 1H), 7.49-7.31 (m, 7H), 7.26-7.22 (m, 1H), 6.78 (td, J = 55.0, 6.5 Hz, 1H), 5.00 (d, J = 4.5 Hz, 1H), 3.91-3.84 (m, 1H); ^{13}C NMR (125 MHz, $CDCl_3$) δ 169.9, 152.7, 136.0, 135.3, 134.7, 134.1, 130.6 (d, J_{C-F} = 7.3 Hz), 129.4, 128.9, 128.7, 128.6, 126.7 (d, J_{C-F} = 4.6 Hz), 126.0, 125.1, 124.9, 124.8, 123.1, 121.4, 117.5 (t, J_{C-F} = 239.3 Hz), 46.6 (t, J_{C-F} = 21.3 Hz), 44.8 (t, J_{C-F} = 6.3 Hz); ^{19}F NMR (470 MHz, $CDCl_3$) δ -116.5 (d, J = 290.0 Hz), -120.7 (d, J = 290.0 Hz). FT-IR: ν (cm^{-1}) 3064, 1454, 1242, 1042, 759, 740. HRMS [ESI] calcd for $C_{22}H_{16}F_2NS$ [M+H]$^+$ 364.0966, found 364.0976.

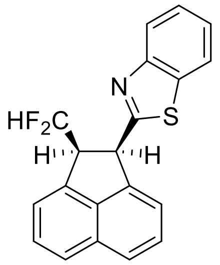

2-(2-(difluoromethyl)-1,2-dihydroacenaphthylen-1-yl)benzo[*d*]thiazole (7b): Colorless oil. Purification by flash column chromatography (eluent: EtOAc/Petroleum ether = 1/20). ^{1}H NMR (500 MHz, $CDCl_3$) δ 8.01 (d, J = 8.5 Hz, 1H), 7.83 (d, J = 8.0 Hz, 1H), 7.80 (d, J = 8.0 Hz, 1H), 7.77 (d, J = 8.0 Hz, 1H) 7.61-7.52 (m, 3H), 7.49-7.45 (m, 2H), 7.39-7.35 (m, 1H), 6.23 (td, J = 56.0, 4.5 Hz, 1H), 5.42 (d, J = 4.0 Hz, 1H), 4.79-4.70 (m, 1H); ^{13}C NMR

(125 MHz, $CDCl_3$) δ 172.2, 153.3, 142.5, 138.1, 137.8 (d, $J_{C\text{-}F}$ = 4.3 Hz), 135.4, 131.8, 128.6, 128.4, 126.4, 125.3, 125.0, 124.8, 123.3, 121.8, 121.3, 116.8 (t, $J_{C\text{-}F}$ = 242.0 Hz), 56.0 (t, $J_{C\text{-}F}$ = 21.0 Hz), 49.4 (t, $J_{C\text{-}F}$ = 5.1 Hz); ^{19}F NMR (470 MHz, $CDCl_3$) δ -119.4 (d, J = 280.6 Hz), -120.4 (d, J = 280.6 Hz). FT-IR: ν (cm^{-1}) 2929, 1734, 1311, 1237, 1043, 756, 729. HRMS [ESI] calcd for $C_{20}H_{14}F_2NS$ $[M+H]^+$ 338.0810, found 338.0814.

5. Product transformations

5.1 Product epimerization

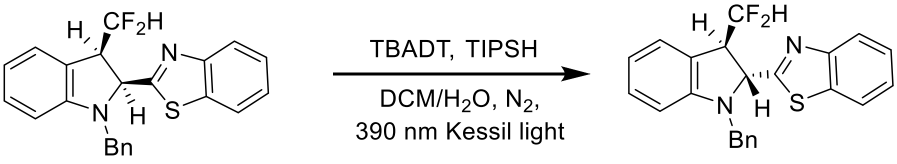

***cis*-3a** ***trans*-3a**, 60%

To a flame-dried reaction flask was added *cis*-**3a** (0.2 mmol) and TBADT (4 mol %), which was subjected to evacuation/ flushing with N_2 for 3 times. DCM/H_2O (1:1) was added to the mixture via syringe, and then TIPSH (40 mol %) was added. The reaction was irradiated by 390 nm Kessil light and stirred at r.t. for 24 h until the starting material had been consumed as determined by TLC. The aqueous layer was extracted with DCM. The combine organic layers were washed with brine, dried over Na_2SO_4, concentrated in vacuo, and purified by flash column chromatography on silica gel (eluent: ethyl acetate/petroleum ether) to give the corresponding product *trans*-**3a**.

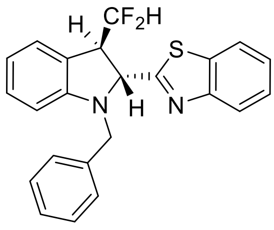

2-(1-benzyl-3-(difluoromethyl)indolin-2-yl)benzo[*d*]thiazole (*trans*-**3a**): Colorless oil. Purification by flash column chromatography (eluent: EtOAc/Petroleum ether = 1/20). ^{1}H NMR (500 MHz, $CDCl_3$) δ 8.02 (d, J = 8.0 Hz, 1H), 7.86 (d, J = 8.0 Hz, 1H), 7.51-7.47 (m, 1H), 7.42-7.38 (m, 1H), 7.31-7.24 (m, 6H), 7.21-7.17 (m, 1H), 6.85-6.81 (m, 1H), 6.57 (d, J = 8.0 Hz, 1H), 6.02 (td, J = 56.0, 4.5 Hz, 1H), 5.14 (d, J = 7.5 Hz, 1H), 4.58 (d, J = 15.5 Hz, 1H), 4.24 (d, J = 16.0 Hz, 1H), 4.00-3.92 (m, 1H); ^{13}C NMR (125 MHz, $CDCl_3$) δ 173.0, 153.3, 151.5, 136.9, 135.2, 129.8, 128.8, 127.8, 127.6, 126.4, 125.8, 125.6, 123.5, 122.8 (d, $J_{C\text{-}F}$ = 2.8 Hz), 122.1, 119.6, 116.9 (t, $J_{C\text{-}F}$ = 242.8 Hz), 109.0, 66.5 (t, $J_{C\text{-}F}$ = 5.4 Hz), 53.8 (t, $J_{C\text{-}F}$ = 21.4 Hz), 52.0; ^{19}F NMR (470 MHz, $CDCl_3$) δ -119.4 (d, J = 282.9 Hz), -121.3 (d, J = 282.9 Hz). FT-IR: ν (cm^{-1}) 2953, 1454, 1205, 1145, 752, 687. HRMS [ESI] calcd for $C_{23}H_{19}F_2N_2S$ $[M+H]^+$ 393.1232, found 393.1240.

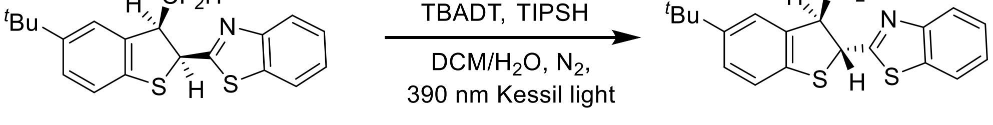

***cis*-4a** ***trans*-4a**, 46%

To a flame-dried reaction flask was added *cis*-**4a** (0.2 mmol) and TBADT (4 mol %), which was subjected to evacuation/ flushing with N_2 for 3 times. DCM/H_2O (1:1) was added to the mixture via syringe, and then TIPSH (40 mol %) was added. The reaction was irradiated by 390 nm Kessil light

and stirred at r.t. for 24 h until the starting material had been consumed as determined by TLC. The aqueous layer was extracted with DCM. The combine organic layers were washed with brine, dried over Na_2SO_4, concentrated in vacuo, and purified by flash column chromatography on silica gel (eluent: ethyl acetate/petroleum ether) to give the corresponding product *trans*-**4a**.

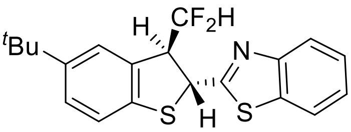

2-(5-(*tert*-butyl)-3-(difluoromethyl)-2,3-dihydrobenzo[*b*]thiophen-2-yl)benzo[*d*]thiazole (*trans*-**4a**): Colorless oil. Purification by flash column chromatography (eluent: EtOAc/Petroleum ether = 1/20). ^{1}H NMR (500 MHz, $CDCl_3$) δ 7.98 (d, *J* = 8.0 Hz, 1H), 7.79 (d, *J* = 8.0 Hz, 1H), 7.48-7.44 (m, 1H), 7.39-7.31 (m, 3H), 7.21 (d, *J* = 8.5 Hz, 1H), 5.97 (td, *J* = 56.5, 6.0 Hz, 1H), 5.27-5.25 (m, 1H), 4.50-4.41 (m, 1H), 1.30 (s, 9H); ^{13}C NMR (125 MHz, $CDCl_3$) δ 173.5, 153.4, 149.2, 136.6, 135.5, 133.2 (d, J_{C-F} = 5.9 Hz), 127.2, 126.4, 125.5, 124.5, 123.3, 122.2, 121.8, 115.3 (t, J_{C-F} = 242.9 Hz), 58.5 (t, J_{C-F} = 26.1 Hz), 50.7 (dd, J_{C-F} = 7.3, 3.1 Hz), 34.7, 31.5; ^{19}F NMR (470 MHz, $CDCl_3$) δ -118.6 (d, *J* = 280.1 Hz), -123.0 (d, *J* = 280.1 Hz). FT-IR: ν (cm^{-1}) 2969, 1437, 1243, 1057, 759, 728. HRMS [ESI] calcd for $C_{20}H_{20}F_2NS_2$ [M+H]$^+$ 376.1000, found 376.1007.

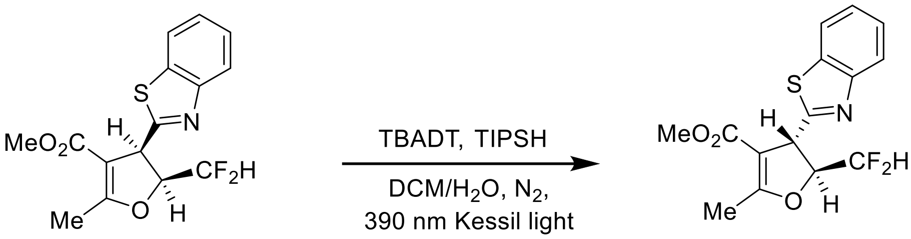

***cis*-5l** ***trans*-5l**, 54%

To a flame-dried reaction flask was added *cis*-**5l** (0.2 mmol) and TBADT (4 mol %), which was subjected to evacuation/ flushing with N_2 for 3 times. DCM/H_2O (1:1) was added to the mixture via syringe, and then TIPSH (40 mol %) was added. The reaction was irradiated by 390 nm Kessil light and stirred at r.t. for 24 h until the starting material had been consumed as determined by TLC. The aqueous layer was extracted with DCM. The combine organic layers were washed with brine, dried over Na_2SO_4, concentrated in vacuo, and purified by flash column chromatography on silica gel (eluent: ethyl acetate/petroleum ether) to give the corresponding product *trans*-**5l**.

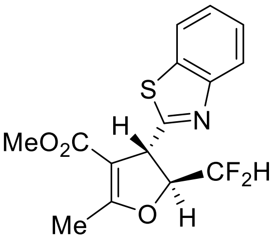

methyl 4-(benzo[*d*]thiazol-2-yl)-5-(difluoromethyl)-2-methyl-4,5-dihydrofuran-3-carboxylate (*trans*-5l): Colorless oil. Purification by flash column chromatography (eluent: EtOAc/Petroleum ether = 1/20). ^{1}H NMR (500 MHz, $CDCl_3$) δ 8.01 (d, *J* = 8.0 Hz, 1H), 7.85 (d, *J* = 8.0 Hz, 1H), 7.49-7.45 (m, 1H), 7.40-7.35 (m, 1H), 5.94 (td, *J* = 55.0, 3.0 Hz, 1H), 5.21-5.14 (m, 1H), 4.94-4.90 (m, 1H), 3.67 (s, 3H), 2.37 (s, 3H); ^{13}C NMR (125 MHz, $CDCl_3$) δ 171.5, 170.3, 164.8, 153.2, 135.5, 126.3, 125.4, 123.3, 121.8, 113.0 (t, J_{C-F} = 244.0 Hz), 105.1, 84.4 (t, J_{C-F} = 26.1 Hz), 51.4, 46.9 (t, J_{C-F} = 3.1 Hz), 14.3; ^{19}F NMR (470 MHz, $CDCl_3$) δ -130.7 (d, *J* = 294.2 Hz), -132.9 (d, *J* = 294.2 Hz). FT-IR: ν (cm^{-1}) 2937, 1598, 1335, 1205, 954, 752. HRMS [ESI] calcd for $C_{15}H_{14}F_2NO_3S$ [M+H]$^+$ 326.0657, found 326.0660.

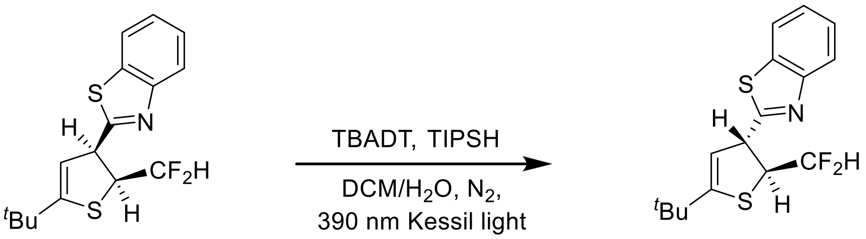

***cis*-6** **_trans_-6**, 40%

To a flame-dried reaction flask was added *cis*-**6** (0.2 mmol) and TBADT (4 mol %), which was subjected to evacuation/ flushing with N_2 for 3 times. DCM/H_2O (1:1) was added to the mixture via syringe, and then TIPSH (40 mol %) was added. The reaction was irradiated by 390 nm Kessil light and stirred at r.t. for 24 h until the starting material had been consumed as determined by TLC. The aqueous layer was extracted with DCM. The combine organic layers were washed with brine, dried over Na_2SO_4, concentrated in vacuo, and purified by flash column chromatography on silica gel (eluent: ethyl acetate/petroleum ether) to give the corresponding product *trans*-**6**.

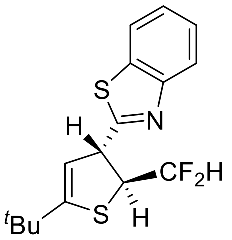

2-(5-(*tert*-butyl)-2-(difluoromethyl)-2,3-dihydrothiophen-3-yl)benzo[*d*]thiazole (*trans*-6): Colorless oil. Purification by flash column chromatography (eluent: EtOAc/Petroleum ether = 1/20). ^{1}H NMR (500 MHz, $CDCl_3$) δ 8.03 (d, *J* = 8.0 Hz, 1H), 7.86 (d, *J* = 8.5 Hz, 1H), 7.50-7.47 (m, 1H), 7.42-7.38 (m, 1H), 5.80 (td, *J* = 55.5, 6.5 Hz, 1H), 5.56 (t, *J* = 3.0 Hz, 1H), 4.89 (dd, *J* = 9.0, 3.0 Hz, 1H), 4.53-4.45 (m, 1H), 1.29 (s, 9H); ^{13}C NMR (125 MHz, $CDCl_3$) δ 168.8, 158.6, 153.3, 135.4, 126.3, 125.5, 123.4, 121.8, 116.0, 115.6 (t, $J_{C\text{-}F}$ = 242.0 Hz), 54.6 (dd, $J_{C\text{-}F}$ = 24.6, 21.3 Hz), 52.4 (d, $J_{C\text{-}F}$ = 5.1 Hz), 35.2, 30.0; ^{19}F NMR (470 MHz, $CDCl_3$) δ -113.3 (d, *J* = 287.2 Hz), -117.3 (d, *J* = 287.2 Hz). FT-IR: ν (cm^{-1}) 2965, 1586, 1458, 1120, 757, 728. HRMS [ESI] calcd for $C_{16}H_{18}F_2NS_2$ $[M+H]^+$ 326.0843, found 326.0848.

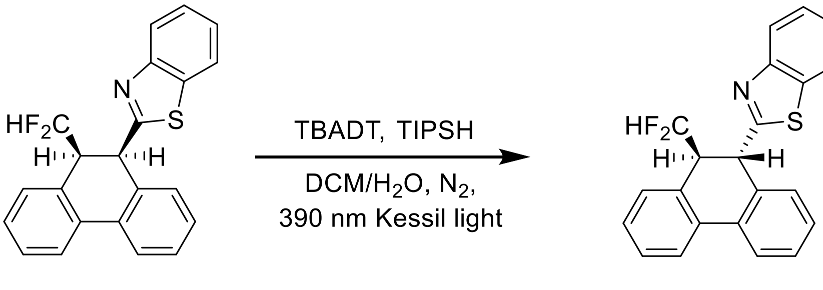

***cis*-7a** **_trans_-7a**, 50%

To a flame-dried reaction flask was added *cis*-**7a** (0.2 mmol) and TBADT (4 mol %), which was subjected to evacuation/ flushing with N_2 for 3 times. DCM/H_2O (1:1) was added to the mixture via syringe, and then TIPSH (40 mol %) was added. The reaction was irradiated by 390 nm Kessil light and stirred at r.t. for 24 h until the starting material had been consumed as determined by TLC. The aqueous layer was extracted with DCM. The combine organic layers were washed with brine, dried over Na_2SO_4, concentrated in vacuo, and purified by flash column chromatography on silica gel (eluent: ethyl acetate/petroleum ether) to give the corresponding product *trans*-**7a**.

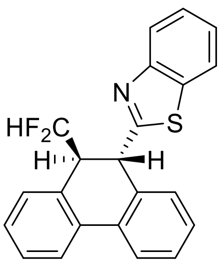

2-(10-(difluoromethyl)-9,10-dihydrophenanthren-9-yl)benzo[*d*]thiazole (*trans*-7a): Colorless oil. Purification by flash column chromatography (eluent: EtOAc/Petroleum ether = 1/20). ^{1}H NMR (500 MHz, $CDCl_3$) δ 7.94 (d, J = 8.0 Hz, 1H), 7.90 (d, J = 8.0 Hz, 1H), 7.84 (d, J = 8.0 Hz, 1H), 7.59 (d, J = 8.0 Hz, 1H), 7.53-7.47 (m, 2H), 7.43-7.36 (m, 3H), 7.33 (d, J = 7.5 Hz, 1H), 7.28 (d, J = 7.0 Hz, 1H), 7.24 (d, J = 8.0 Hz, 1H), 5.61 (td, J = 56.5, 7.0 Hz, 1H), 4.93-4.90 (m, 1H), 4.14-4.06 (m, 1H); ^{13}C NMR (125 MHz, $CDCl_3$) δ 172.9, 153.1, 135.4, 133.8, 133.6, 133.1, 132.0, 130.4, 129.7, 129.5, 129.3 (d, J_{C-F} = 6.5 Hz), 128.8, 128.7, 126.1, 124.9, 124.6, 124.1, 122.9, 121.5, 116.1 (dd, J_{C-F} = 245.6, 242.0 Hz), 48.9 (t, J_{C-F} = 21.1 Hz), 43.8 (dd, J_{C-F} = 6.5, 2.9 Hz); ^{19}F NMR (470 MHz, $CDCl_3$) δ -119.1 (d, J = 278.2 Hz), -121.0 (d, J = 278.7 Hz). FT-IR: ν (cm^{-1}) 3064, 1454, 1242, 1042, 759, 740. HRMS [ESI] calcd for $C_{22}H_{16}F_2NS$ $[M+H]^+$ 364.0966, found 364.0971.

5.2 Aromatization via dehetarylation

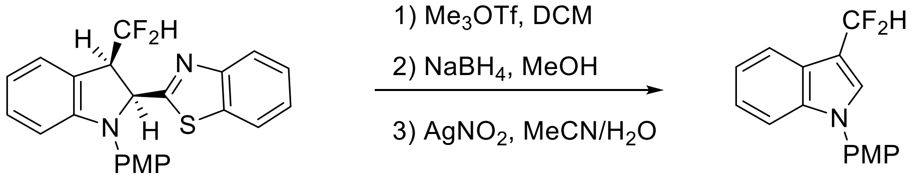

3w **8**, 68%

To a flame-dried reaction flask was added **3w** (0.2 mmol), activated 4 Å powdered molecular sieves (300 mg), and anhydrous CH_2Cl_2 (2 mL). The reaction was stirred at r.t. for 10 min, and then Me_3OTf (1.0 mmol) was added in twice. The suspension was stirred at r.t. for 4 h and then concentrated to dryness without filtering off the molecular sieves, giving the crude *N*-methylbenzothiazolium salt. To a cooled (0 °C), stirred suspension of the above *N*-methylbenzothiazolium salt in CH_3OH (2 mL) was added $NaBH_4$ (0.5 mmol). The mixture was stirred at r.t. for an additional 30 min, diluted with acetone, filtered through a pad of Celite, and concentrated, giving the crude benzothiazoline. To a vigorously stirred solution of the above benzothiazoline in CH_2Cl_2 (0.6 mL) and CH_3CN (3.0 mL) were added H_2O (0.36 mL) and then $AgNO_3$ (0.6 mmol). The mixture was stirred at r.t. until the benzothiazoline were completely consumed as determined by TLC, and then diluted with 1 M phosphate buffer at pH 7 (0.1 mL). Stirring was continued for an additional 15 min, and then the reaction mixture was diluted with 1 M phosphate buffer at pH 7 (5 mL) and the suspension was extracted with EtOAc (2 × 10 mL), and the combined organic layers were dried over Na_2SO_4, filtered through a pad of Celite, and concentrated. The residue was eluted from a short column of silica gel (EtOAc/petroleum ether =1:40) to give the corresponding products **8**.

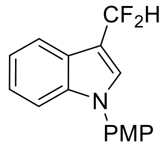

3-(difluoromethyl)-1-(4-methoxyphenyl)-1*H*-indole (8): Colorless oil. Purification by flash column chromatography (eluent: EtOAc/Petroleum ether = 1/20). ^{1}H NMR (500 MHz, $CDCl_3$) δ 7.83 (d, J = 8.0 Hz, 1H), 7.47 (t, J = 2.5 Hz, 1H), 7.42 (d, J = 7.5 Hz, 1H), 7.40-7.37 (m, 2H), 7.29-7.22 (m, 2H), 7.06-7.03 (m, 2H), 7.02 (t, J = 56.5 Hz, 1H), 3.89 (s, 3H); ^{13}C NMR (125 MHz, $CDCl_3$) δ 159.0, 137.1, 131.8, 127.9 (t, J_{C-F} = 8.5 Hz), 126.4, 125.2 (t, J_{C-F} = 2.6 Hz), 123.5, 121.4, 120.0, 115.0, 113.2, 111.3 (t,

$J_{C\text{-}F}$ = 25.9 Hz), 111.0, 55.8; ^{19}F NMR (470 MHz, $CDCl_3$) δ -107.2 (s). FT-IR: ν (cm-1) 3323, 1618, 1378, 1105, 945, 852. HRMS [ESI] calcd for $C_{16}H_{14}F_2NO$ $[M+H]^+$ 274.1038, found 274.1043.

5.3 Transformation of CF_2H group

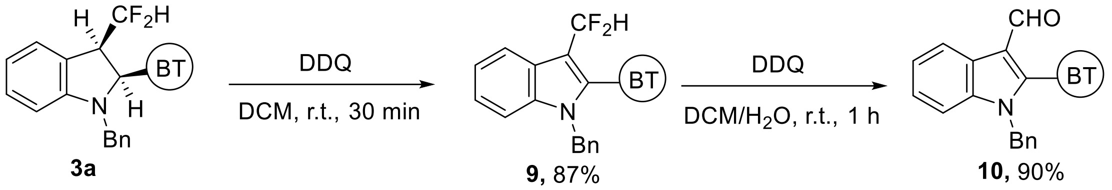

To a flame-dried reaction flask was added **3a** (0.2 mmol) and DDQ (0.4 mmol), which was subjected to evacuation/ flushing with N_2 for 3 times. DCM (2 mL) was added to the mixture via syringe, then the reaction was stirred at r.t. for 30 min until the starting material had been consumed as determined by TLC. The aqueous layer was extracted with DCM. The combine organic layers were washed with brine, dried over Na_2SO_4, concentrated in vacuo, and purified by flash column chromatography on silica gel (eluent: ethyl acetate/petroleum ether) to give the corresponding product **9**.

To a flame-dried reaction flask was added **9** (0.2 mmol) and DDQ (0.2 mmol), which was subjected to evacuation/ flushing with N_2 for 3 times. DCM/H_2O (2/0.1 mL) was added to the mixture via syringe, then the reaction was stirred at r.t. for 1 h until the starting material had been consumed as determined by TLC. The aqueous layer was extracted with DCM. The combine organic layers were washed with brine, dried over Na_2SO_4, concentrated in vacuo, and purified by flash column chromatography on silica gel (eluent: ethyl acetate/petroleum ether) to give the corresponding product **10**.

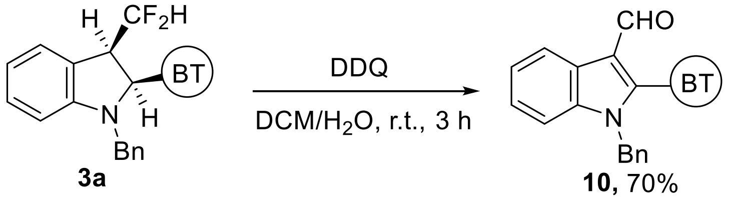

To a flame-dried reaction flask was added **3a** (0.2 mmol) and DDQ (0.4 mmol), which was subjected to evacuation/ flushing with N_2 for 3 times. DCM/H_2O (2/0.1 mL) was added to the mixture via syringe, then the reaction was stirred at r.t. for 3 h until the starting material had been consumed as determined by TLC. The aqueous layer was extracted with DCM. The combine organic layers were washed with brine, dried over Na_2SO_4, concentrated in vacuo, and purified by flash column chromatography on silica gel (eluent: ethyl acetate/petroleum ether) to give the corresponding product **10**.

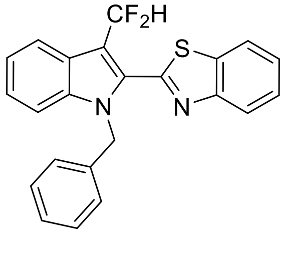

2-(1-benzyl-3-(difluoromethyl)-1*H*-indol-2-yl)benzo[*d*]thiazole (9): Colorless oil. ^{1}H NMR (500 MHz, $CDCl_3$) δ 8.16 (d, J = 8.5 Hz, 1H), 8.04 (d, J = 8.0 Hz, 1H), 7.93 (d, J = 8.0 Hz, 1H), 7.59-7.55 (m, 1H), 7.49-7.45 (m, 1H), 7.38-7.28 (m, 3H), 7.25-7.14 (m, 4H), 7.05-7.01 (m, 2H), 5.76 (s, 2H); ^{13}C NMR (125 MHz, $CDCl_3$) δ 155.9, 153.6, 137.9, 137.1, 135.9, 131.8 (t, J = 10.0 Hz), 128.9, 127.7, 127.0, 126.5, 126.2,

125.1, 124.6, 124.2, 122.1, 121.7, 121.6, 113.0 (t, J = 229.4 Hz), 112.4 (t, J = 27.3 Hz), 111.1, 48.3; ^{19}F NMR (470 MHz, $CDCl_3$) δ -105.3 (s). FT-IR: ν (cm^{-1}) 3279, 1618, 1476, 1235, 1078, 845. HRMS [ESI] calcd for $C_{23}H_{17}F_2N_2S$ $[M+H]^+$ 391.1075, found 391.1077.

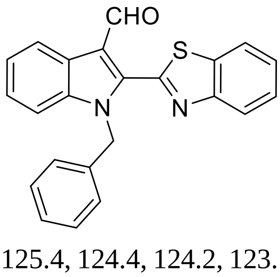

2-(benzo[*d*]thiazol-2-yl)-1-benzyl-1*H*-indole-3-carbaldehyde (10): Colorless oil. ^{1}H NMR (500 MHz, $CDCl_3$) δ 10.41 (s, 1H), 8.55-8.52 (m, 1H), 8.19 (d, J = 8.0 Hz, 1H), 7.97 (d, J = 8.0 Hz, 1H), 7.62-7.57 (m, 1H), 7.53-7.49 (m, 1H), 7.41-7.37 (m, 3H), 7.25-7.20 (m, 3H), 7.07-7.03 (m, 2H), 5.79 (s, 2H); ^{13}C NMR (125 MHz, $CDCl_3$) δ 186.5, 155.0, 153.6, 139.8, 137.8, 136.4, 136.3, 128.9, 127.9, 127.2, 126.6, 126.6, 125.8, 125.4, 124.4, 124.2, 123.1, 121.6, 118.7, 111.2, 48.6. FT-IR: ν (cm^{-1}) 3373, 1728, 1424, 1275, 1034, 831. HRMS [ESI] calcd for $C_{23}H_{17}N_2OS$ $[M+H]^+$ 369.1056, found 369.1061.

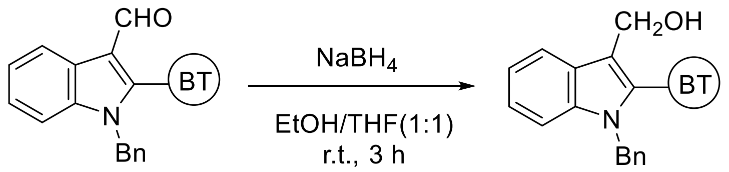

10 **11**, 95%

To a flame-dried reaction flask was added **10** (0.2 mmol) and EtOH/THF (1/1 mL), then $NaBH_4$ (0.4 mmol) was added to the mixture. The reaction was stirred at r.t. for 3 h until the starting material had been consumed as determined by TLC. The aqueous layer was extracted with EtOAc. The combine organic layers were washed with brine, dried over Na_2SO_4, concentrated in vacuo, and purified by flash column chromatography on silica gel (eluent: ethyl acetate/petroleum ether) to give the corresponding product **11**.

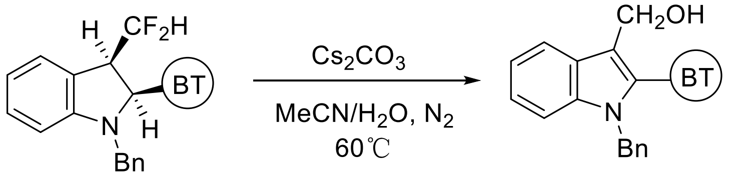

3a **11**, 68%

To a flame-dried reaction flask was added **3a** (0.2 mmol) and Cs_2CO_3 (0.4 mmol), then DCM/H_2O (2/0.1 mL) was added to the mixture. The reaction was stirred at rt for 3 h until the starting material had been consumed as determined by TLC. The aqueous layer was extracted with EtOAc. The combine organic layers were washed with brine, dried over Na_2SO_4, concentrated in vacuo, and purified by flash column chromatography on silica gel (eluent: ethyl acetate/petroleum ether) to give the corresponding product **11**.

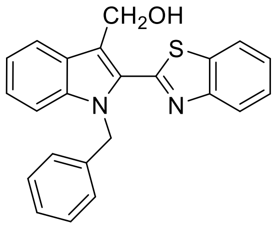

(2-(benzo[*d*]thiazol-2-yl)-1-benzyl-1*H*-indol-3-yl)methanol (11): Colorless oil. ^{1}H NMR (500 MHz, $CDCl_3$) δ 8.11 (d, J = 8.0 Hz, 1H), 7.88-7.85 (m, 2H), 7.56-7.51 (m, 1H), 7.44-7.39 (m, 1H), 7.31-7.27 (m, 4H), 7.26-7.22 (m, 2H), 7.09-7.06 (m, 2H), 5.82 (s, 2H), 5.02 (s, 2H), 4.36 (br, 1H); ^{13}C NMR (125 MHz, $CDCl_3$) δ 158.1, 152.8, 138.5, 137.5, 135.1, 131.6, 129.0, 127.6, 126.9, 126.9, 126.2, 125.7, 125.0, 123.5,

121.6, 121.3, 121.0, 120.2, 110.8, 55.3, 48.5. FT-IR: ν (cm-1) 3479, 1698, 1435, 1138, 934, 831. HRMS [ESI] calcd for $C_{23}H_{19}N_2OS$ $[M+H]^+$ 371.1213, found 371.1219.

6. Single-crystal X-ray diffraction analysis

6.1 For compound 3r

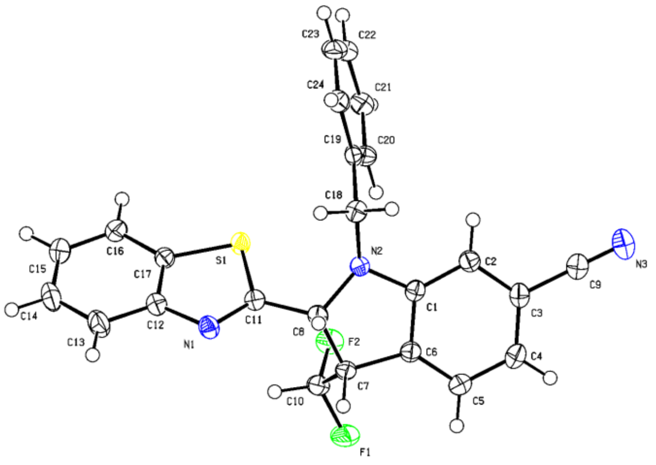

Figure S2. Single-crystal structure of 3r

Bond precision:	C-C = 0.0021 A		Wavelength=1.54178
Cell:	a=9.8850(2)	b=11.0446(2)	c=11.2308(2)
	alpha=107.722(1)	beta=104.448(1)	gamma=111.117(1)
Temperature:	173 K		
	Calculated	Reported	
Volume	996.54(4)	996.54(3)	
Space group	P -1	P -1	
Hall group	-P 1	-P 1	
Moiety formula	C24 H17 F2 N3 S	C24 H17 F2 N3 S	
Sum formula	C24 H17 F2 N3 S	C24 H17 F2 N3 S	
Mr	417.47	417.46	
Dx,g cm-3	1.391	1.391	
Z	2	2	
Mu (mm-1)	1.737	1.737	
F000	432.0	432.0	
F000'	433.94		
h,k,lmax	11,13,13	11,13,13	
Nref	3654	3654	
Tmin,Tmax	0.795,0.784	0.699,0.753	
Tmin'	0.721		

Correction method = # Reported T Limits: Tmin=0.699 Tmax=0.753

AbsCorr = MULTI-SCAN

Data completenesss = 0.998	Theta(max) = 68.312
R(reflections) = 0.0308 (3442)	wR2(reflections) = 0.0838 (3645)
S = 1.035	Npar = 274

6.2 For compound 5o

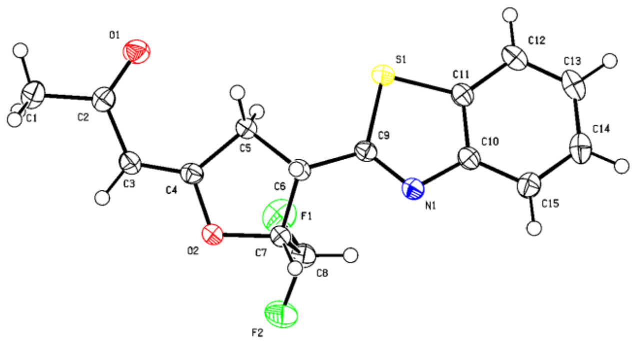

Figure S3. Single-crystal structure of 5o

Bond precision:	C-C = 0.0020 A		Wavelength=1.54178
Cell:	a=5.4759(1)	b=10.6882(2)	c=12.1415(3)
	alpha=92.345(1)	beta=96.685(1)	gamma=101.692(1)
Temperature:	173 K		

	Calculated	Reported
Volume	689.62(3)	689.61(2)
Space group	P -1	P -1
Hall group	-P 1	-P 1
Moiety formula	C15 H13 F2 N O2 S	C15 H13 F2 N O2 S
Sum formula	C15 H13 F2 N O2 S	C15 H13 F2 N O2 S
Mr	309.32	309.32
Dx,g cm-3	1.490	1.490
Z	2	2
Mu (mm-1)	2.350	2.350
F000	320.0	320.0
F000'	433.94	
h,k,lmax	6,12,14	6,12,14
Nref	2543	2538
Tmin,Tmax	0.656,0.687	0.646,0.753
Tmin'	0.595	

Correction method = # Reported T Limits: Tmin=0.646 Tmax=0.753

AbsCorr = MULTI-SCAN	
Data completenesss = 0.998	Theta(max) = 68.283
R(reflections) = 0.0310 (2396)	wR2(reflections) = 0.0868 (2538)
S = 0.983	Npar = 191

6.3 For compound *trans*-4a

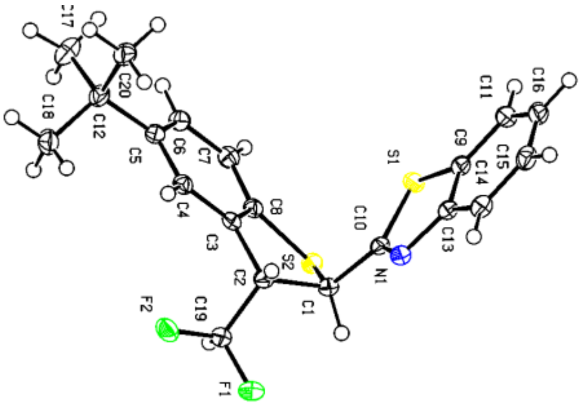

Figure S4. Single-crystal structure of *trans*-4a

Bond precision:	C-C = 0.0020 A		Wavelength=1.54184
Cell:	a=11.3756(1)	b=12.7920(2)	c=13.8892(2)
	alpha=78.573(1)	beta=66.760(1)	gamma=74.049(1)
Temperature:	100 K		
	Calculated	Reported	
Volume	1776.39(4)	1776.39(4)	
Space group	P -1	P -1	
Hall group	-P 1	-P 1	
Moiety formula	C20 H19 F2 N S2	C20 H19 F2 N S	
Sum formula	C20 H19 F2 N S2	C20 H19 F2 N S	
Mr	375.48	375.48	
Dx,g cm-3	1.404	1.404	
Z	4	4	
Mu (mm-1)	2.909	2.909	
F000	784.0	784.0	
F000'	788.74		
h,k,lmax	14,16,17	14,15,17	
Nref	7583	7084	
Tmin,Tmax	0.622,0.665	0.728,1.000	
Tmin'	0.564		

Correction method = # Reported T Limits: Tmin=0.728 Tmax=1.000

AbsCorr = MULTI-SCAN

Data completenesss = 0.934	Theta(max) = 77.514
R(reflections) = 0.0312 (6759)	wR2(reflections) = 0.0883 (7084)
S = 1.044	Npar = 458

7. Mechanistic studies

7.1 Radical trap experiments

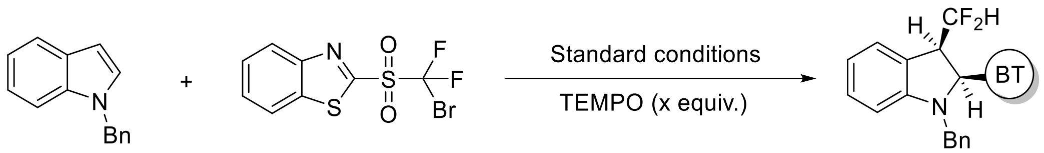

1a 2a 3a

TEMPO (2.0 equiv), **3a** (45%)
TEMPO (4.0 equiv), **3a** (15%)

Indole **1a** (0.4 mmol), sulfone reagent **2a** (0.2 mmol), sodium ascorbate (0.5 mmol) and TEMPO (x equiv) were loaded in a Schlenk tube, which was subjected to evacuation/ flushing with N_2 for 3 times. Dry DME (8.0 mL)/ H_2O (1.0 mL) was then added to the tube via syringe. The reaction was irradiated by 456 nm Kessil light and stirred at r.t. until the starting material had been consumed as determined by TLC. The reaction mixture was quenched with H_2O, and the aqueous layer was extracted with DCM. The combine organic layers were washed with brine, dried over Na_2SO_4, concentrated in vacuo, and purified by flash column chromatography on silica gel (eluent: ethyl acetate/petroleum ether) to give the corresponding product **3a**. The results indicate that the addition of free radical scavengers effectively suppresses the conversion.

7.2 Deuterium-labeling experiments

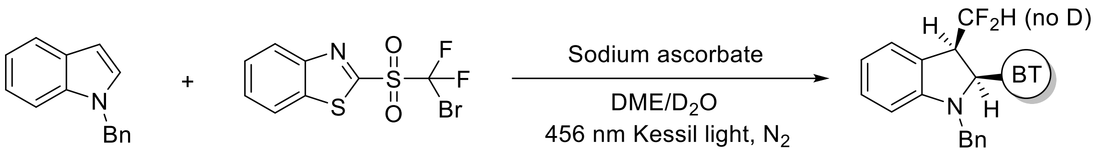

1a 2a **3a**, 72%

To a flame-dried Schlenk tube was loaded indole **1a** (0.4 mmol), sulfone reagent **2a** (0.2 mmol), and sodium ascorbate (0.5 mmol), which was subjected to evacuation/ flushing with N_2 for 3 times. Dry DME (8.0 mL)/ D_2O (1.0 mL) was added to the tube via syringe. The reaction was irradiated by 456 nm Kessil light and stirred at r.t. until the starting material had been consumed as determined by TLC. The reaction mixture was quenched with H_2O, and the aqueous layer was extracted with DCM. The combine organic layers were washed with brine, dried over Na_2SO_4, concentrated in vacuo, and purified by flash column chromatography on silica gel (eluent: ethyl acetate/petroleum ether) to give the corresponding product **3a**. The result indicates that no deuterated products are generated.

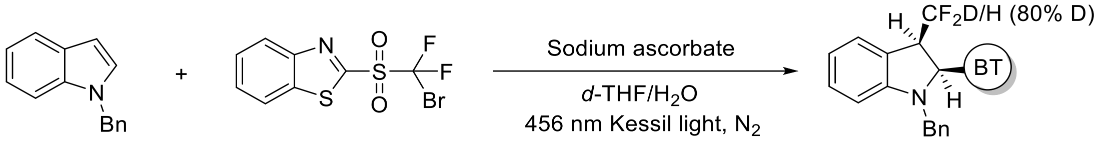

1a **2a** **3a**, 48%

To a flame-dried Schlenk tube was loaded indole **1a** (0.4 mmol), sulfone reagent **2a** (0.2 mmol), and sodium ascorbate (0.5 mmol), which was subjected to evacuation/ flushing with N_2 for 3 times. Dry *d*-THF (8.0 mL)/ H_2O (1.0 mL) was added to the tube via syringe. The reaction was irradiated by 456 nm Kessil light and stirred at r.t. until the starting material had been consumed as determined by TLC. The reaction mixture was quenched with H_2O, and the aqueous layer was extracted with DCM. The combine organic layers were washed with brine, dried over Na_2SO_4, concentrated in vacuo, and purified by flash column chromatography on silica gel (eluent: ethyl acetate/petroleum ether) to give the corresponding product **3a**. The result indicates that a deuterated product with 80% D-incorporation is obtained.

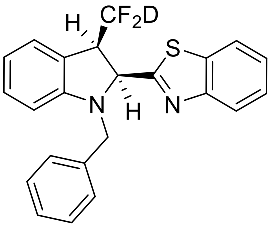

2-(1-benzyl-3-(difluoromethyl-*d*)indolin-2-yl)benzo[*d*]thiazole (3a): Colorless oil. ^{1}H NMR (500 MHz, $CDCl_3$) δ 8.04 (d, J = 8.5 Hz, 1H), 7.86 (d, J = 8.0 Hz, 1H), 7.53-7.49 (m, 1H), 7.44-7.40 (m, 1H), 7.36-7.28 (m, 6H), 7.21-7.17 (m, 1H), 6.88-6.84 (m, 1H), 6.56 (d, J = 8.0 Hz, 1H), 5.81 (td, J = 55.5, 5.5 Hz, 0.2 H), 5.38 (d, J = 9.5 Hz, 1H), 4.55 (d, J = 15.5 Hz, 1H), 4.17-4.09 (m, 2H); ^{13}C NMR (125 MHz, $CDCl_3$) δ 169.1, 153.2, 152.2, 136.9, 135.0, 129.6, 128.7, 127.7, 127.5, 126.5 (d, J_{C-F} = 1.5 Hz), 126.3, 125.6, 123.6 (d, J_{C-F} = 6.9 Hz), 123.2, 121.9, 120.0, 109.6, 67.6 (dd, J_{C-F} = 6.0, 2.9 Hz), 52.4, 49.9 (t, J_{C-F} = 22.0 Hz); ^{19}F NMR (470 MHz, $CDCl_3$) δ (*non-deuterated product*) -115.8 (d, J = 287.6 Hz), -121.1 (d, J = 287.6 Hz); (*deuterated product*) -116.5 (dt, J = 288.6, 8.5 Hz), -121.9 (dt, J = 287.6, 8.5 Hz). FT-IR: ν (cm^{-1}) 2945, 1554, 1312, 1029, 833, 690. HRMS [ESI] calcd for $C_{23}H_{18}DF_2N_2S$ $[M+H]^+$ 394.1294, found 394.1298.

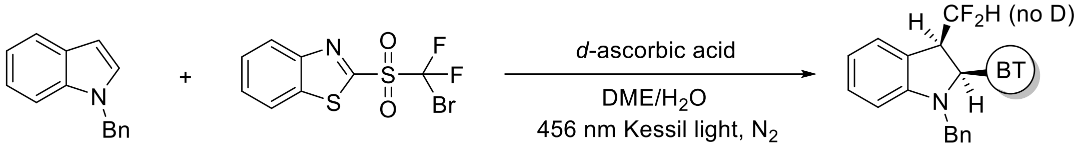

1a **2a** **3a**, 38%

To a flame-dried Schlenk tube was loaded indole **1a** (0.4 mmol), sulfone reagent **2a** (0.2 mmol), and the deuterated ascorbic acid (0.5 mmol), which was subjected to evacuation/ flushing with N_2 for 3 times. Dry DME (8.0 mL)/ H_2O (1.0 mL) was added to the tube via syringe. The reaction was irradiated by 456 nm Kessil light and stirred at r.t. until the starting material had been consumed as determined by TLC. The reaction mixture was quenched with H_2O, and the aqueous layer was extracted with DCM. The combine organic layers were washed with brine, dried over Na_2SO_4, concentrated in vacuo, and purified by flash column chromatography on silica gel (eluent: ethyl acetate/petroleum ether) to give the corresponding product **3a**. The result indicates that no deuterated products are generated.

7.3 Photolysis experiment

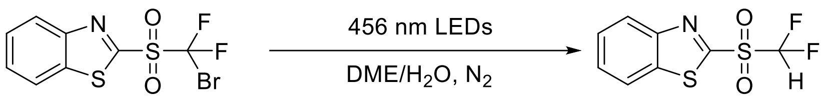

2a **2a'**, 45%

2a (0.2 mmol) was loaded in a flask, which was subjected to evacuation/ flushing with N_2 for 3 times. Dry DME (8.0 mL)/ H_2O (1.0 mL) was added to the tube via syringe. The reaction was irradiated by 456 nm Kessil light and stirred at r.t. Then concentrated in vacuo, and purified by flash column chromatography on silica gel (eluent: ethyl acetate/petroleum ether) to give the **2a'**.

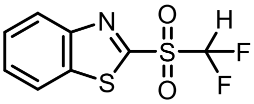

2a': 46% yield, white solid. Purification by flash column chromatography (eluent: EtOAc/Petroleum ether = 1/4). ^{1}H NMR (400 MHz, $CDCl_3$) δ 8.33 – 8.31 (m, 1H), 8.09 – 8.06 (m, 1H), 7.71 – 7.67 (m, 2H), 6.60 (t, J = 53.2 Hz, 1H); ^{13}C NMR (125 MHz, $CDCl_3$) δ 158.9, 153.1, 138.0, 129.1, 128.4, 126.4, 122.5, 114.6 (t, $J_{C\text{-}F}$ = 286.4 Hz); ^{19}F NMR (471 MHz, $CDCl_3$) δ -121.4.

7.4 UV-Vis measurement

Stock solution of **2a** were prepared of 0.05 of the concentration used in the reaction. The solutions were prepared in the presence of air using acetone as solvent.

Mixture 2a: 8.7 mg **2a** was placed in a 10 mL brown reagent bottle equipped with a magnetic stirring bar, and then dry DME (8.0 mL)/ H_2O (1.0 mL) was added. The mixture was stirred at room temperature until solid was completely dissolved. **2a** (0.006 M).

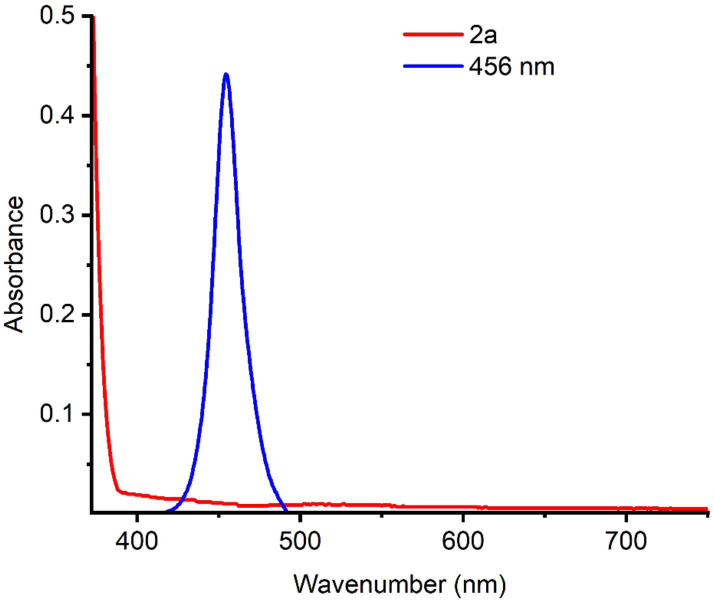

Figure S5. UV-vis spectra of 2a and emission spectra of light sources.

7.5 Quantum yield measurements

Determination of the light intensity at 456 nm

The photon flux of the kessil light (40 W, λ max = 456 nm) was determined by standard ferrioxalate actinometry following a modified literature procedure of Yoon[13] and Glorius[14]. A 0.15 M solution

of ferrioxalate was prepared by dissolving potassium ferrioxalate hydrate (0.737 g) in H_2SO_4 (10 mL of a 0.05 M solution). A buffered solution of 1,10-phenanthroline was prepared by dissolving 1,10-phenanthroline (5.0 mg) and sodium acetate (1.13 g) in H_2SO_4 (5.0 mL of a 0.5 M solution). Both solutions were stored in the dark. To determine the photon flux of the LED, the ferrioxalate solution (3.0 mL) was placed in a cuvette and irradiated for 60 seconds at λmax = 456 nm. After irradiation, the phenanthroline solution (0.525 mL) was added to the cuvette and the mixture was allowed to stir in the dark for 1 h to allow the ferrous ions to completely coordinate to the phenanthroline. The absorbance of the solution was measured at 510 nm. The same procedure was repeated two more times. A nonirradiated sample was also prepared and the absorbance at 510 nm was measured. The average of the absorption of the irradiated and non-irradiated samples was determined and used to calculate the generated amount of Fe(II) according to the Lambert-Beer law (equation 1),

$$\textbf{mol Fe}^{2+} = (V \times \Delta A_{510nm}) / (l \times \varepsilon) \qquad (1)$$

where V is the total volume ($3.525*10^{-3}$ L), ΔA_{510nm} the difference between absorbance of irradiated samples and the non-irradiated (control) ones (at λ = 510 nm), l is the path length of the cuvette (1.0 cm), and ε is the molar attenuation coefficient of the ferrioxalate actinometer λ = 510 nm (11100 L· mol–1·cm–1)[15]. The photonflux can be calculated using equation 2,

$$\textbf{photo flux} = \text{mol } Fe^{2+} / (\Phi_F \times t \times f) \qquad (2)$$

where Φ_F is the quantum yield of the ferrioxalate actinometer (1.11 at λ = 436 nm)[16] and t is the irradiation time (60 s). The fraction of light absorbed at λ = 456 nm by the actinometer (f) is calculated by using equation 3. A_{456nm} is the absorbance of the ferrioxalate solution at λ = 456 nm.

$$f = 1 - 10^{-}A_{456\,nm} \qquad (3)$$

The absorbance (A_{456nm}) of the ferrioxalate solution was measured to be > 3 indicating that > 99.9% of the photons are absorbed (f > 0.999). The photon flux Φq was therefore calculated to be $5.3*10^{-9}$ einstein s^{-1} as an average of three experiments.

Determination of the quantum yield

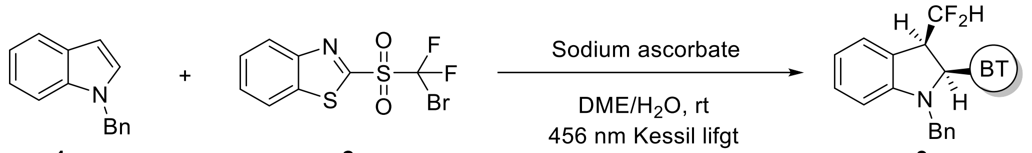

1a 2a 3a

The reaction mixture was stirred and irradiated by blue LED (λmax = 456 nm) for 900 s. The yield of product was determined by ^1H NMR analysis using dibromomethane as an internal standard. The yield of **3a** was determined to be 20.0% (0.02×10^{-3} mol of **3a**). The reaction quantum yield (Φ) was determined using equation 4 where the photon flux is 5.3×10^{-9} einsteins s^{-1} (determined by actinometry as described above), t is the reaction time (900 s) and f is the fraction of incident light absorbed by the catalyst, determined using (equation 3).

$$\textbf{Quantum Yield} = \text{moles of product formed} / (\text{flux} \times f \times t) \qquad (4)$$

$$= 0.02 \times 10^{-3} / (5.3 \times 10^{-9} \times 1 \times 900) = 4.2$$

Conclusion: Although the light on-off experiments showed that the product formation occurred only during the periods of constant light irradiation. Typical lifetime of radical chain process can be on the second or sub-second timescale, which means chain processes can terminate faster than the

timescale of the analytical measurement used. The quantum yield measurement ($\Phi = 4.2$) indicates that a radical chain process is involved during the reaction.

8. NMR spectra

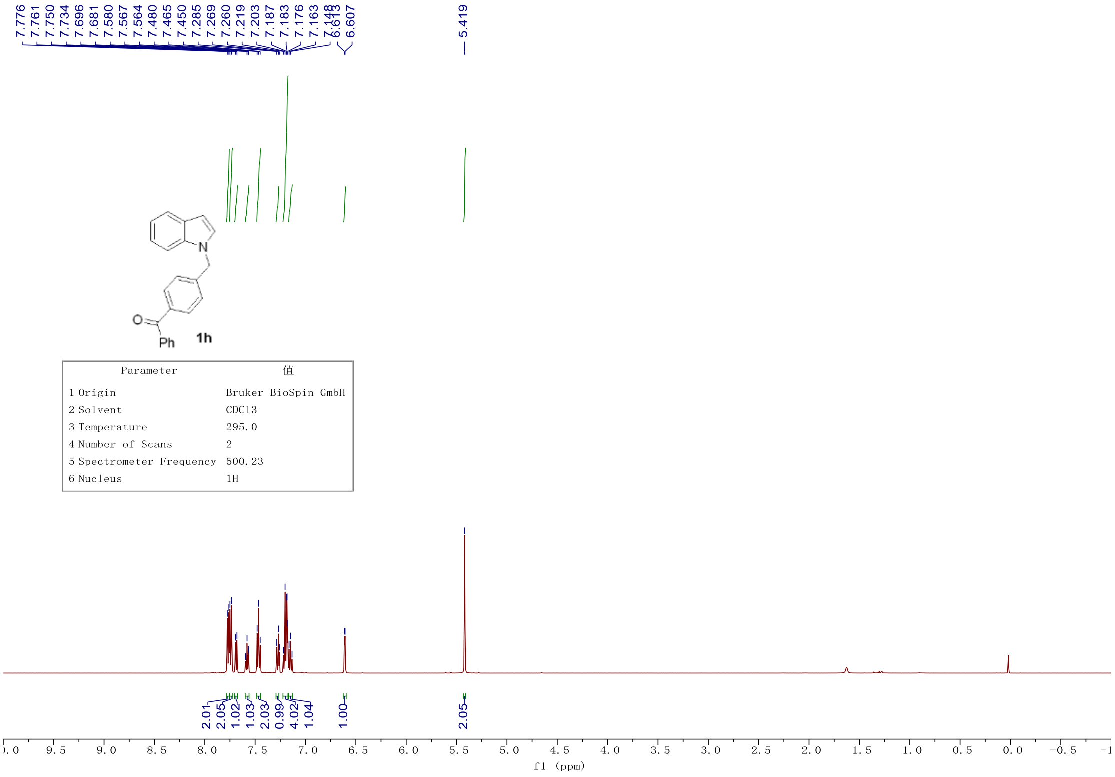

Figure S6. ^{1}H-NMR of **1h**

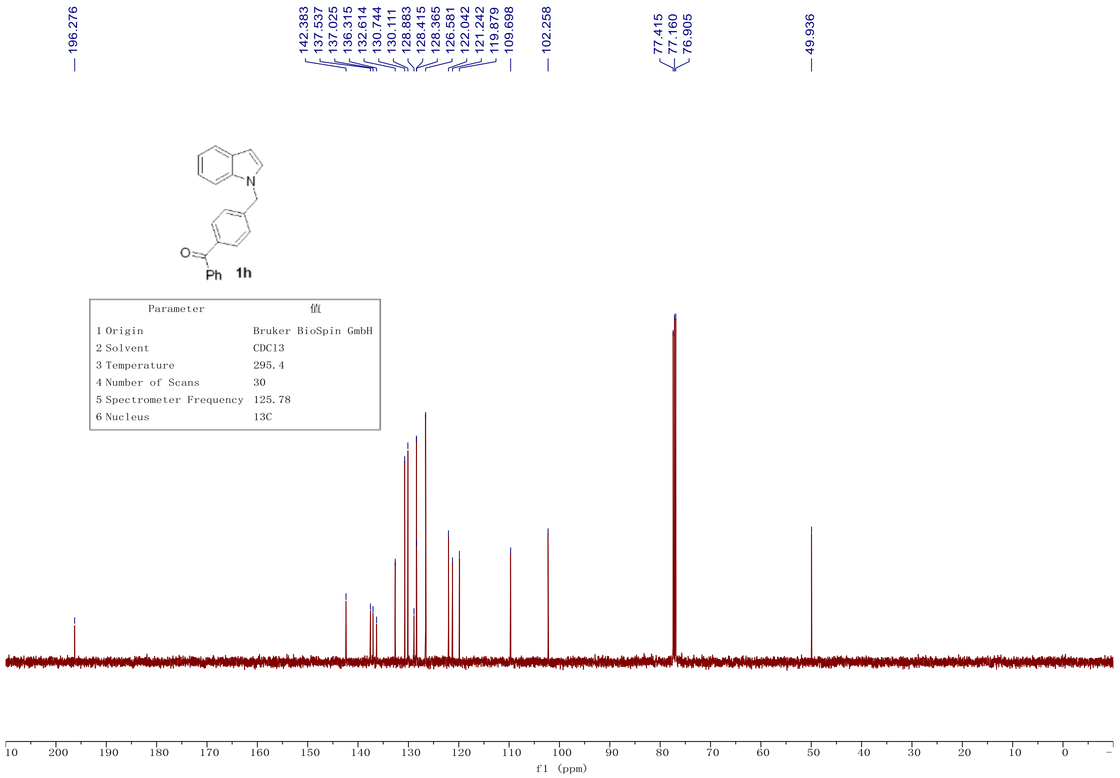

Figure S7. ^{13}C-NMR of **1h**

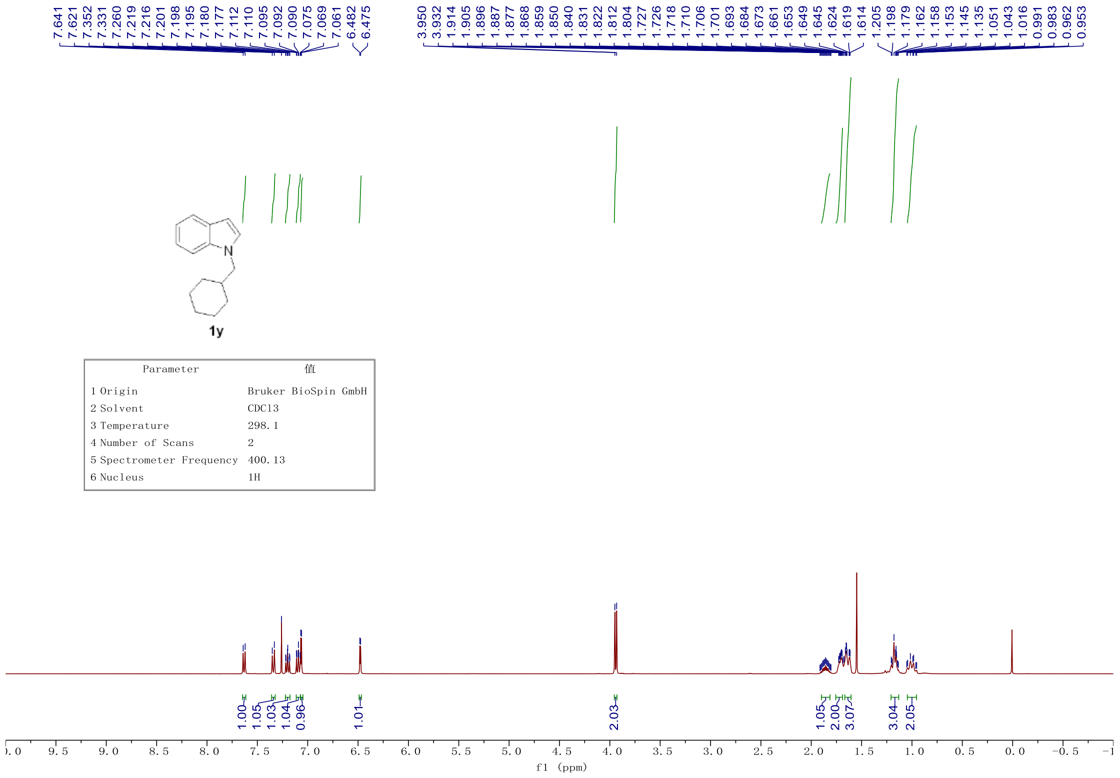

Figure S8. ^{1}H-NMR of **1y**

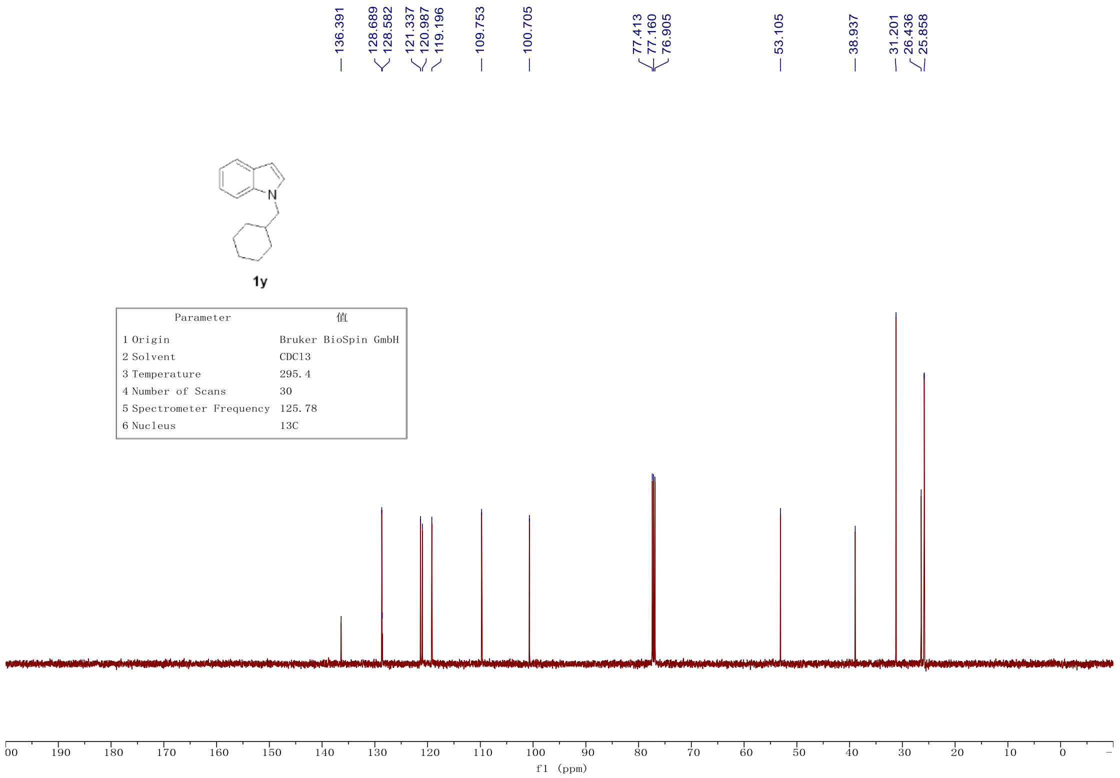

Figure S9. ^{13}C-NMR of **1y**

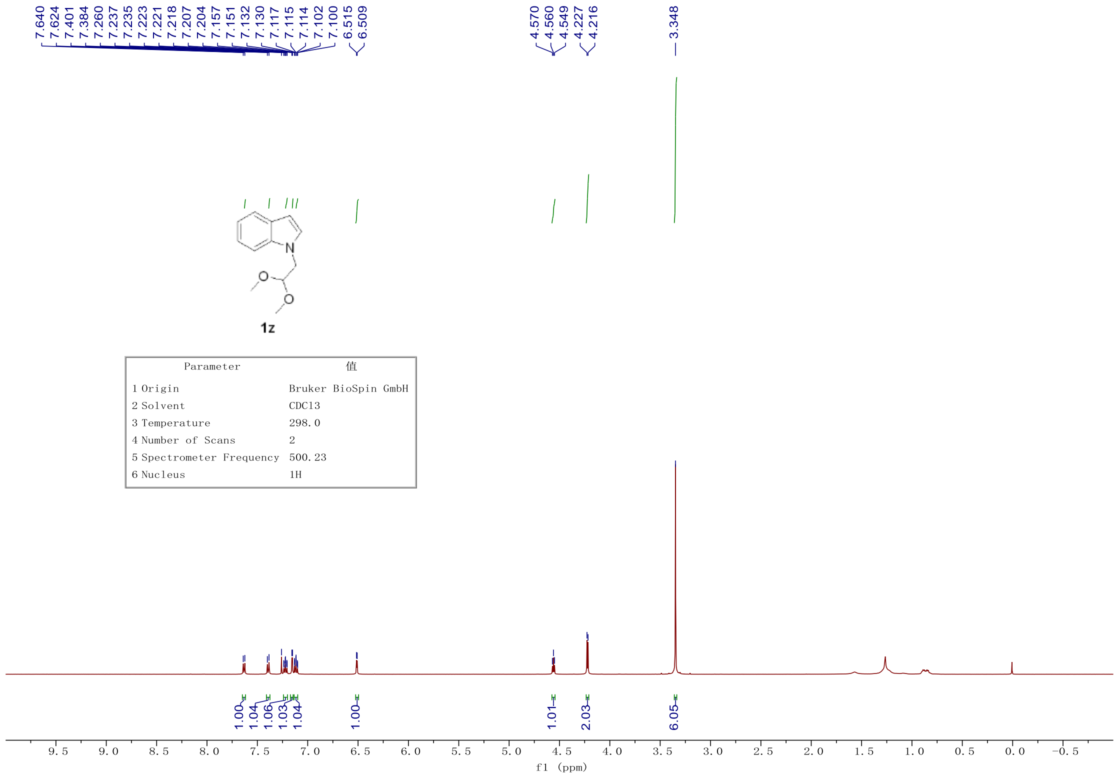

Figure S10. ^{1}H-NMR of **1z**

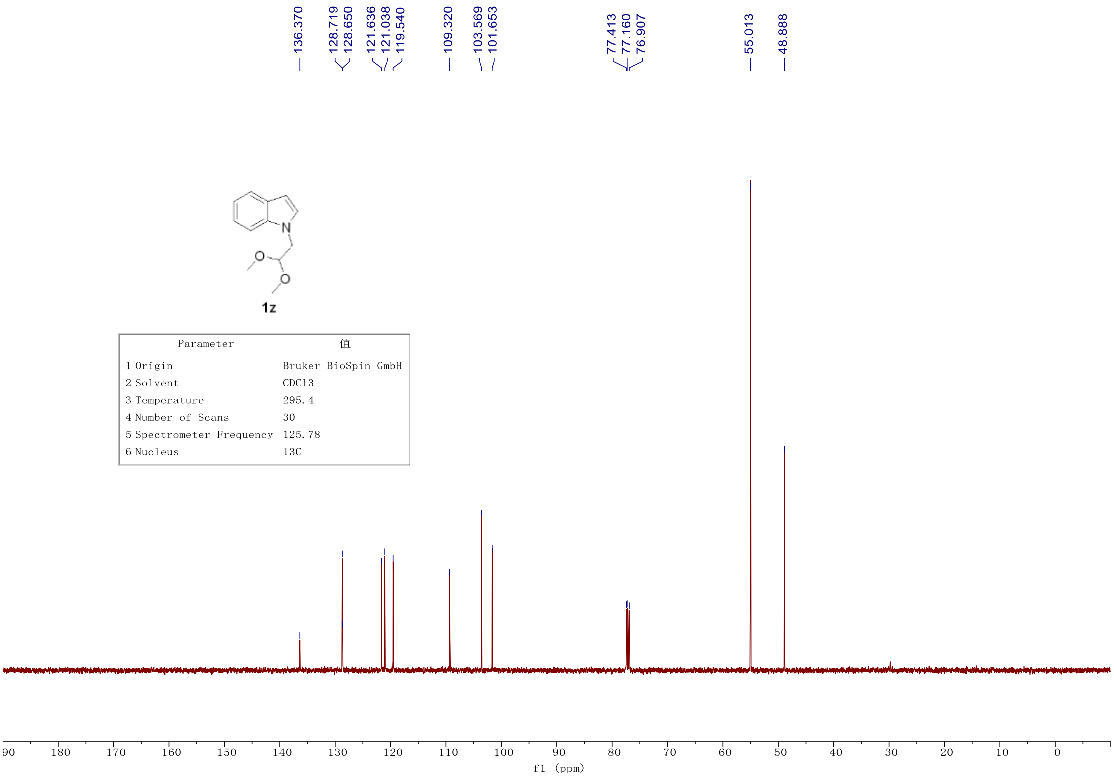

Figure S11. ^{13}C-NMR of **1z**

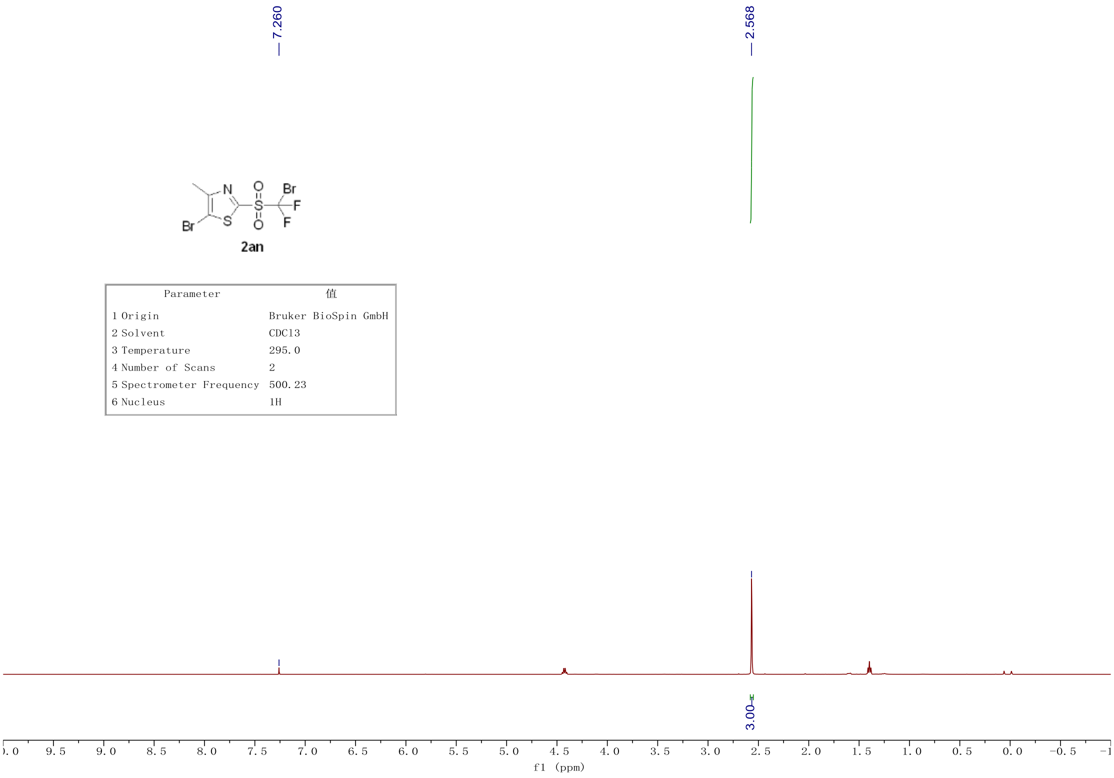

Figure S12. ^{1}H-NMR of **2an**

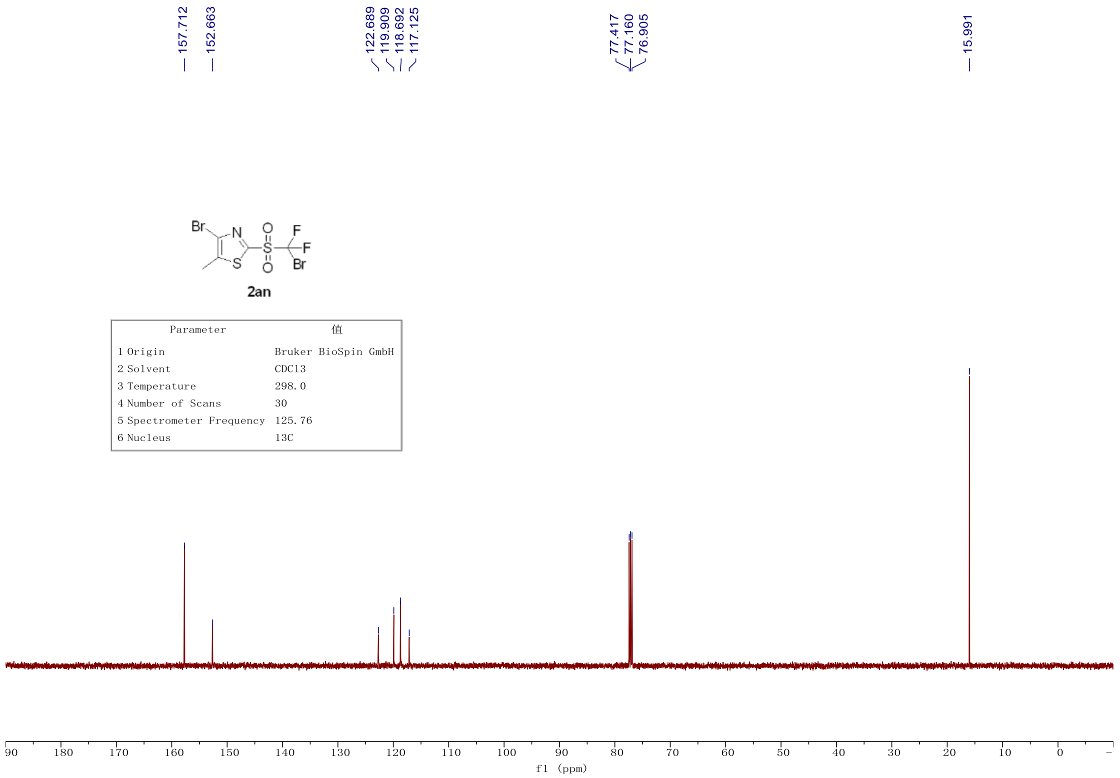

Figure S13. ^{13}C-NMR of **2an**

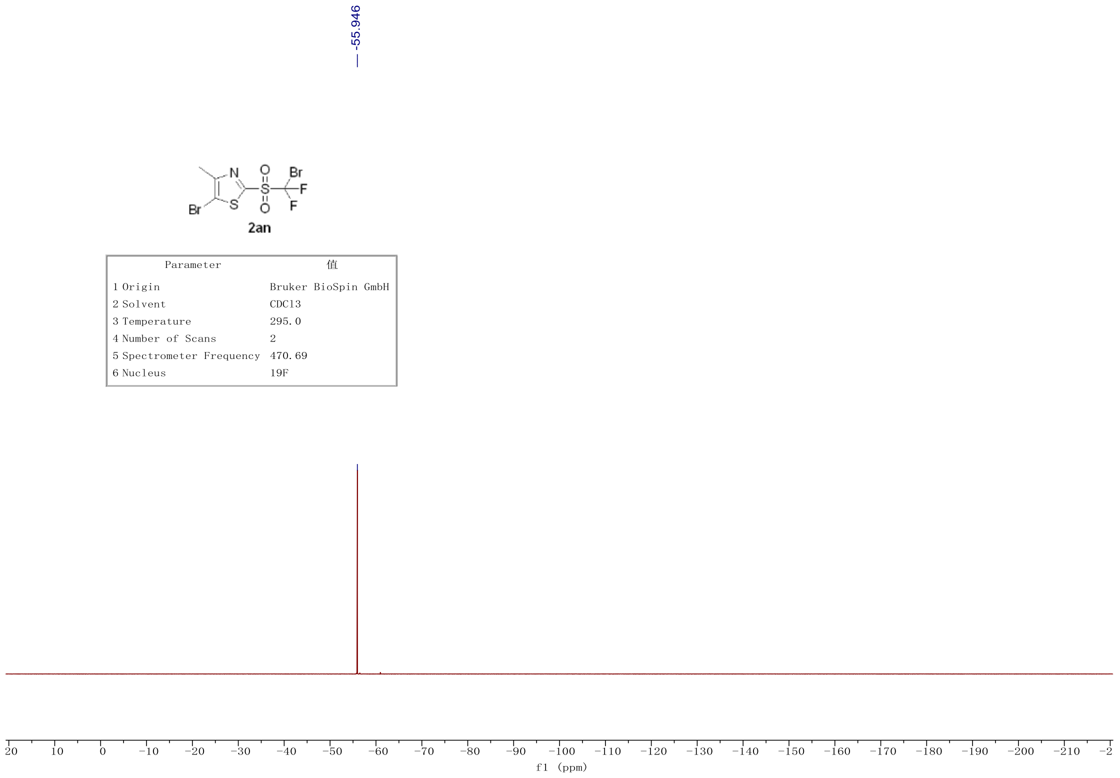

Figure S14 ^{19}F-NMR of **2an**

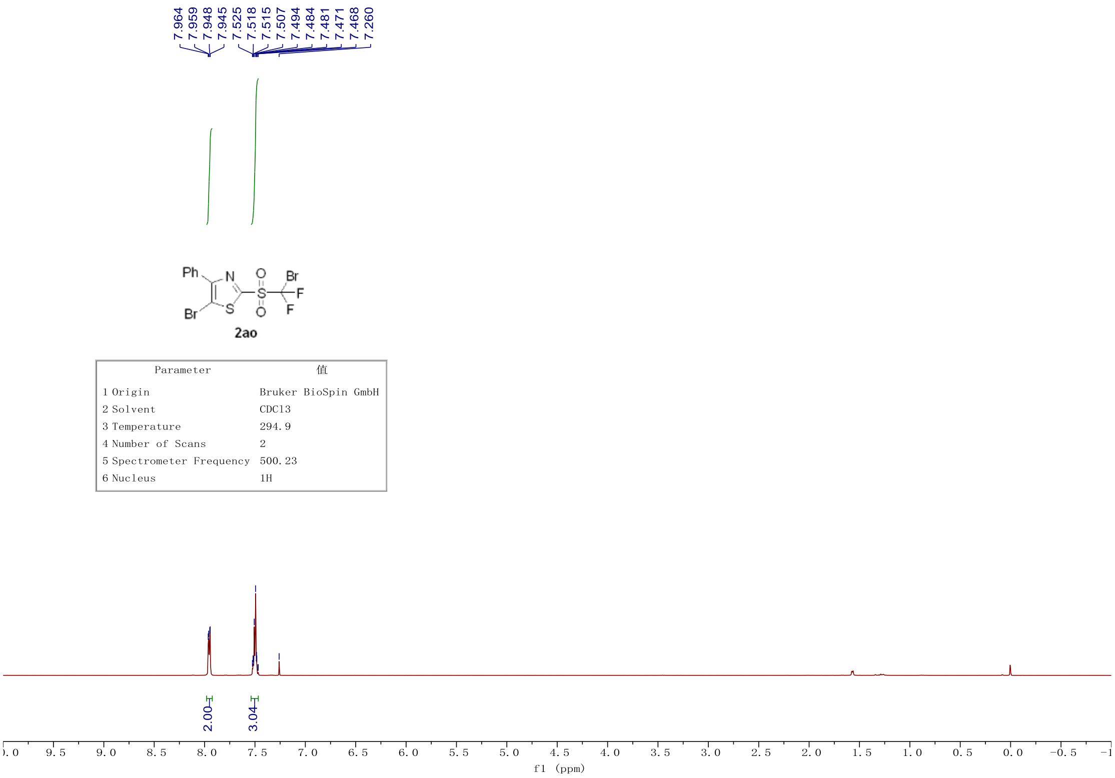

Figure S15. ^{1}H-NMR of **2ao**

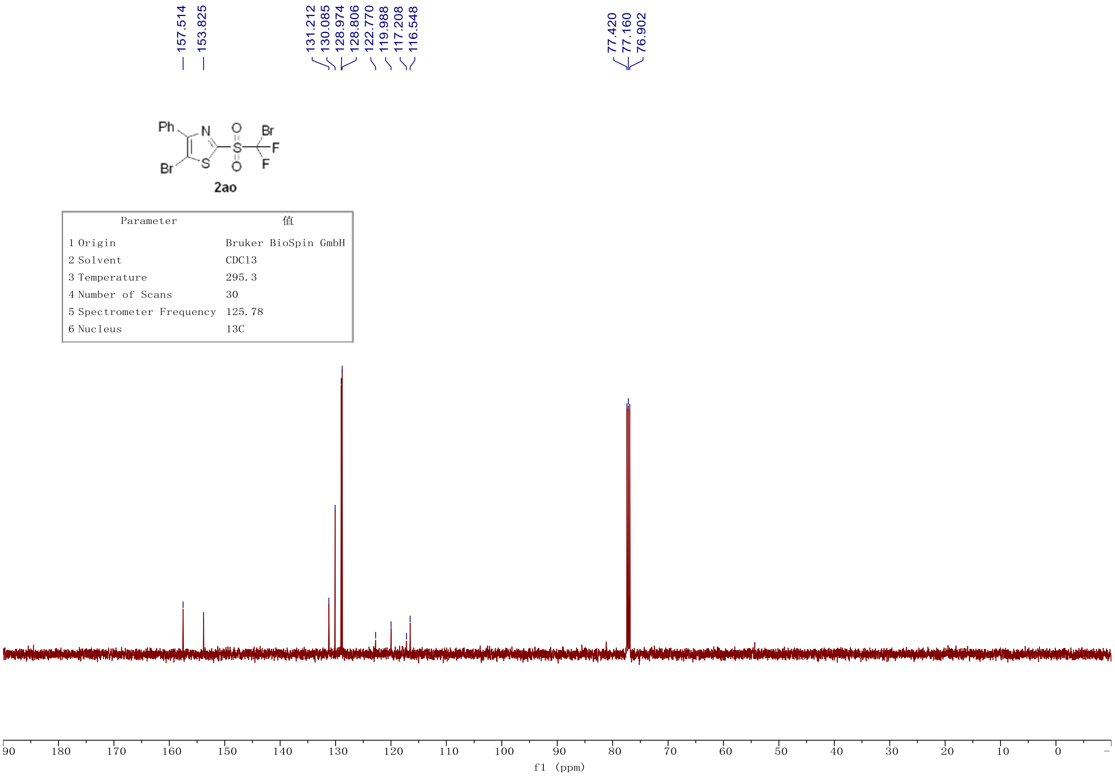

Figure S16. ^{13}C-NMR of **2ao**

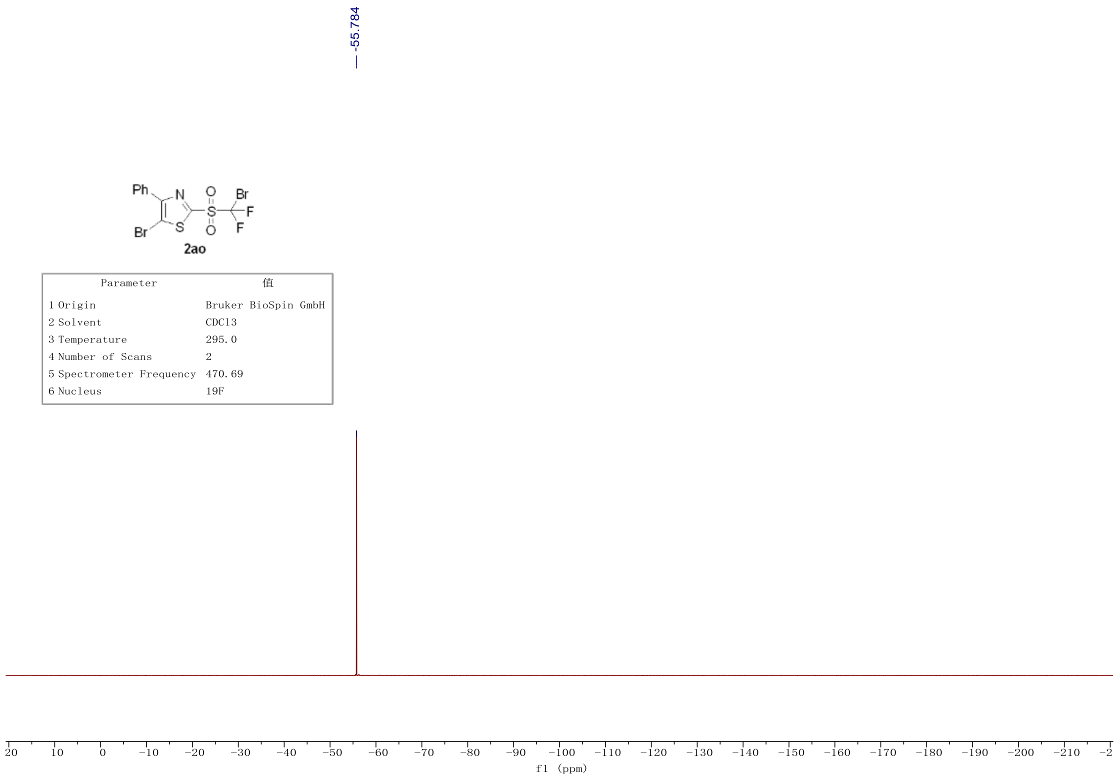

Figure S17. ^{19}F-NMR of **2ao**

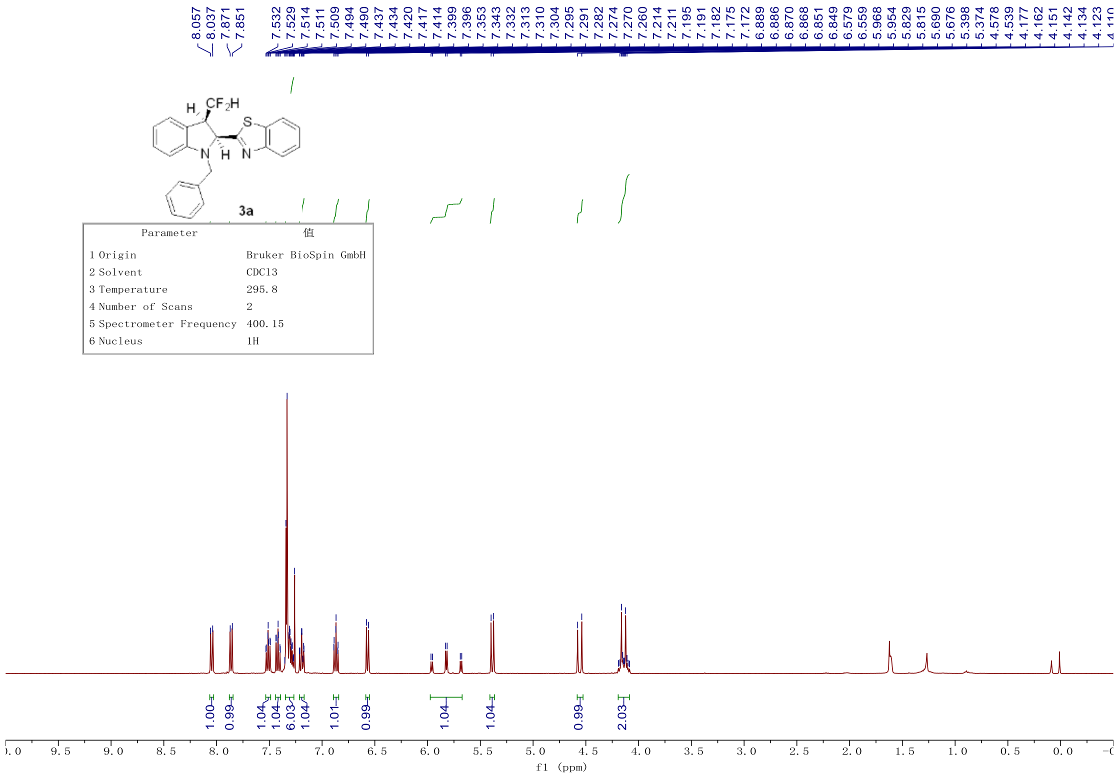

Figure S18. ^{1}H-NMR of **3a**

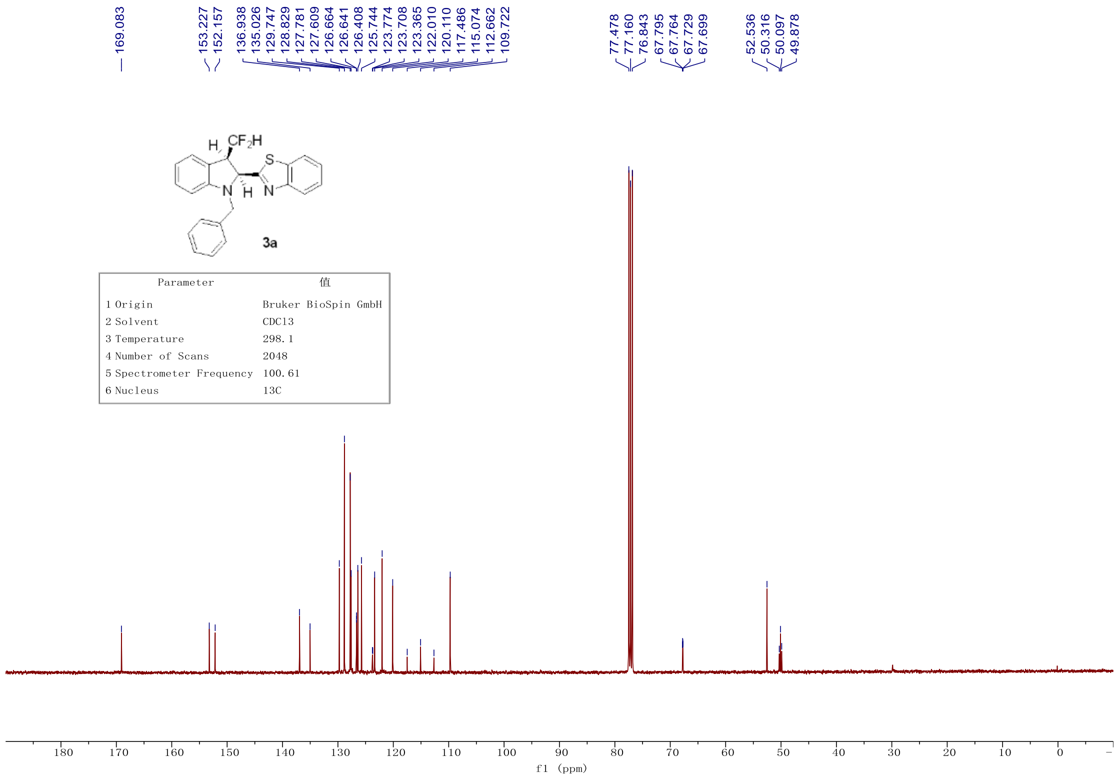

Figure S19. ^{13}C-NMR of **3a**

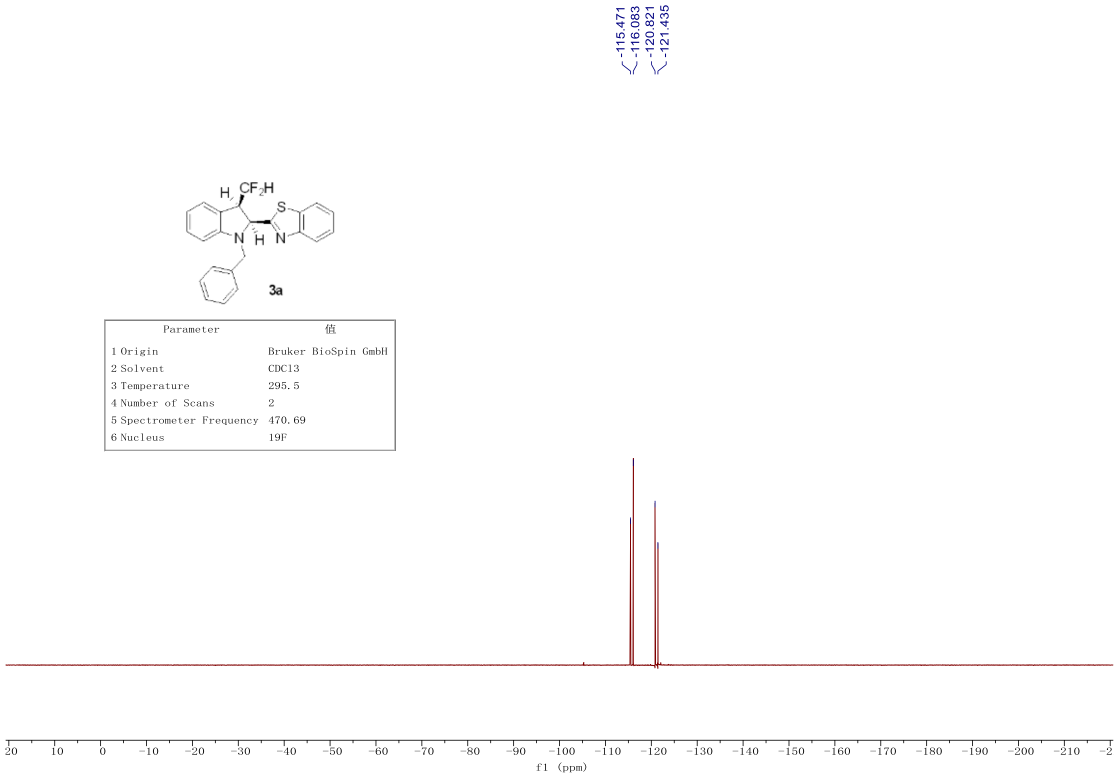

Figure S20. ^{19}F-NMR of **3a**

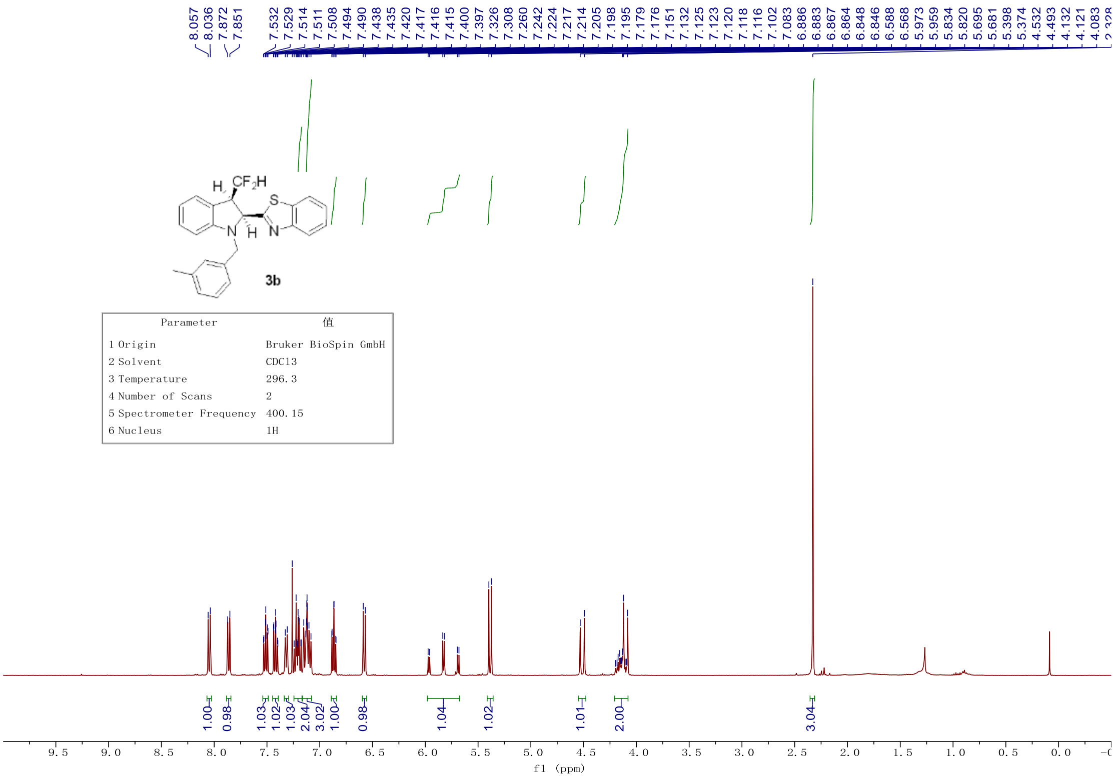

Figure S21. ^{1}H-NMR of **3b**

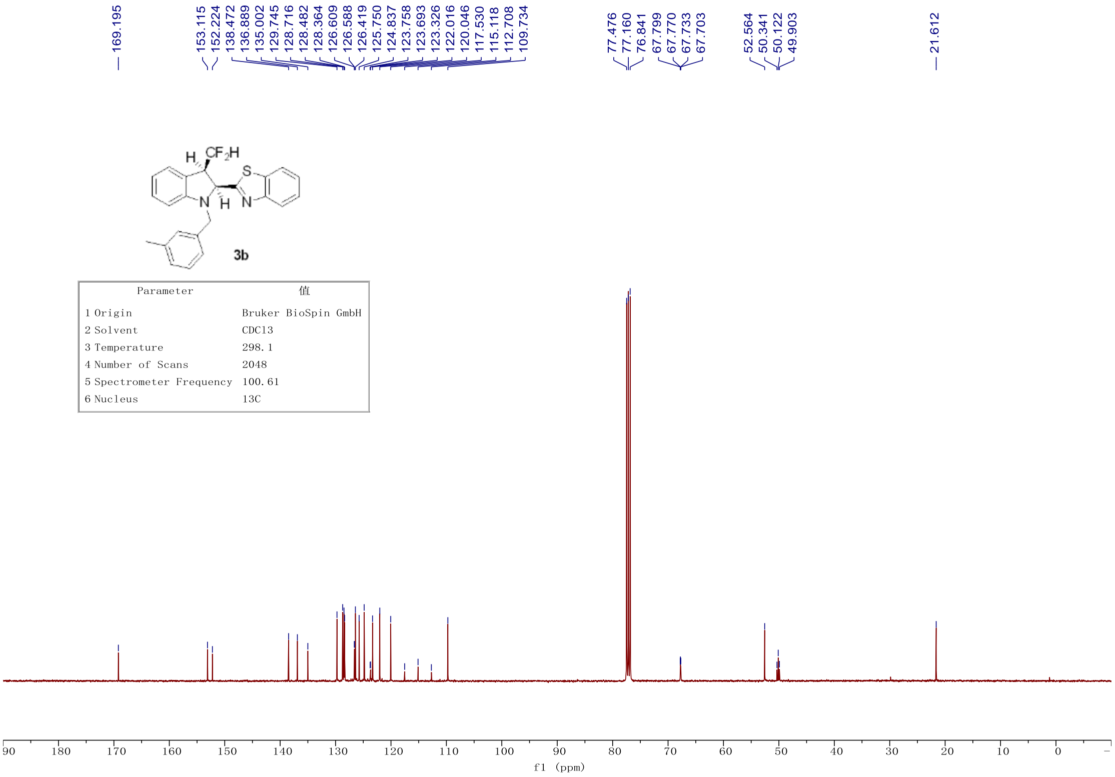

Figure S22. ^{13}C-NMR of **3b**

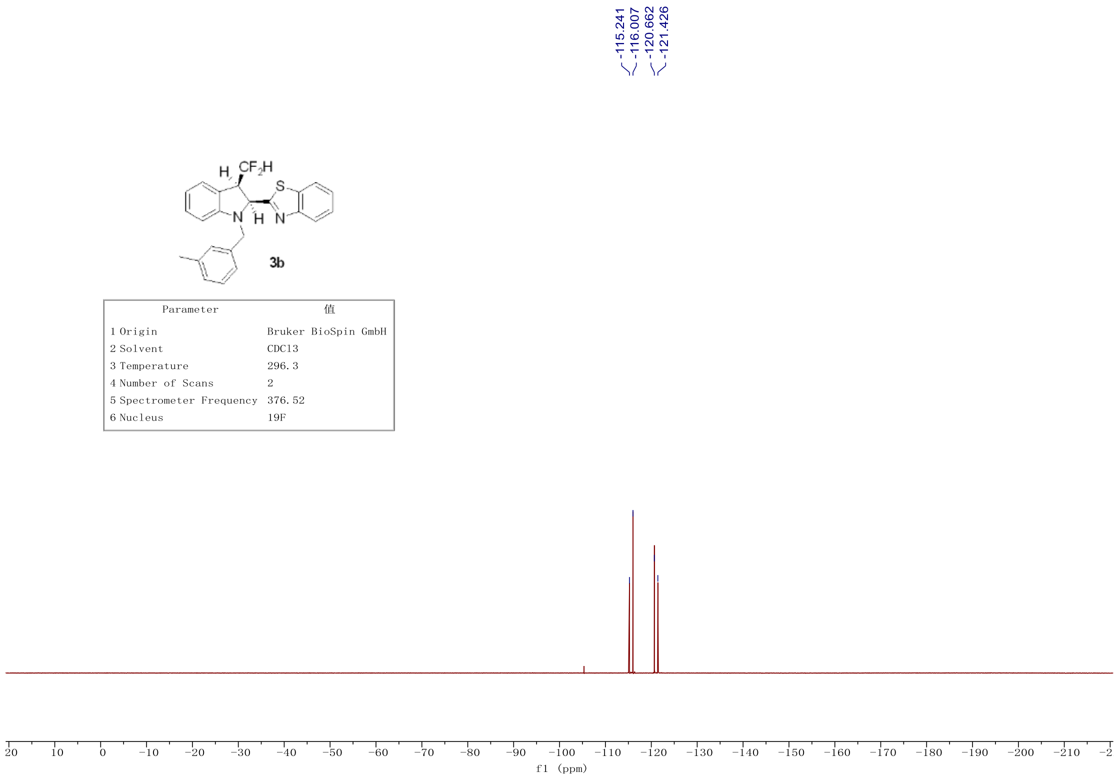

Figure S23. ^{19}F-NMR of **3b**

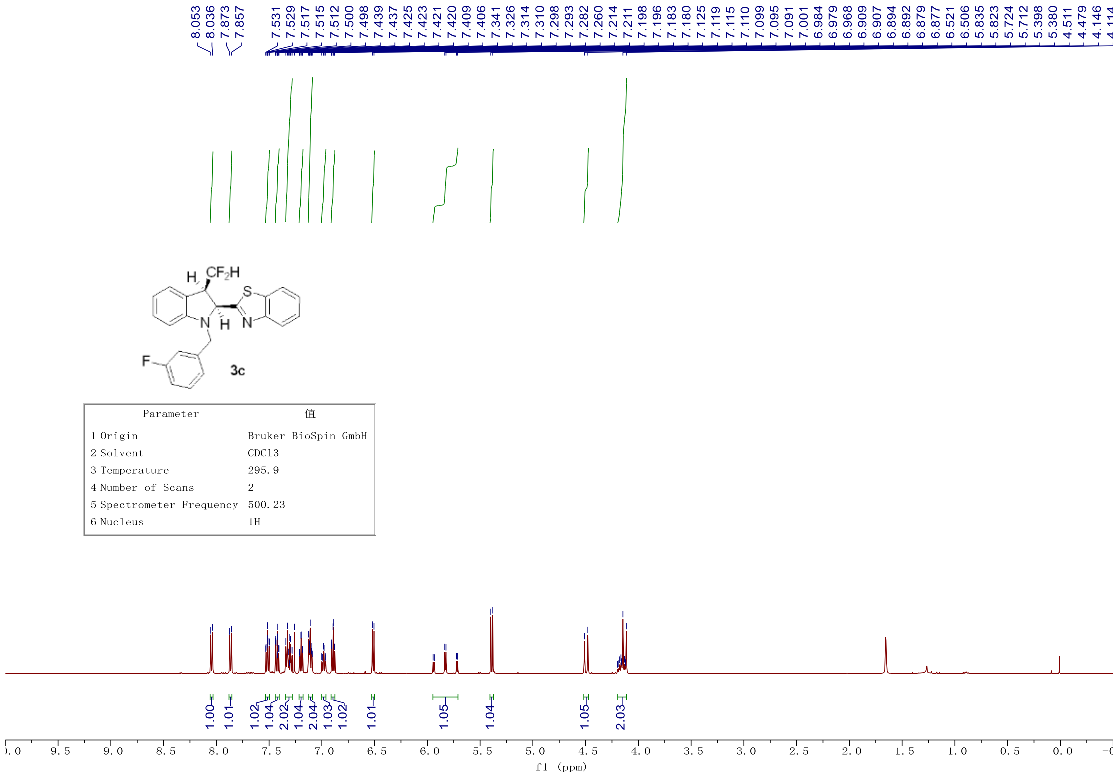

Figure S24. ^{1}H-NMR of **3c**

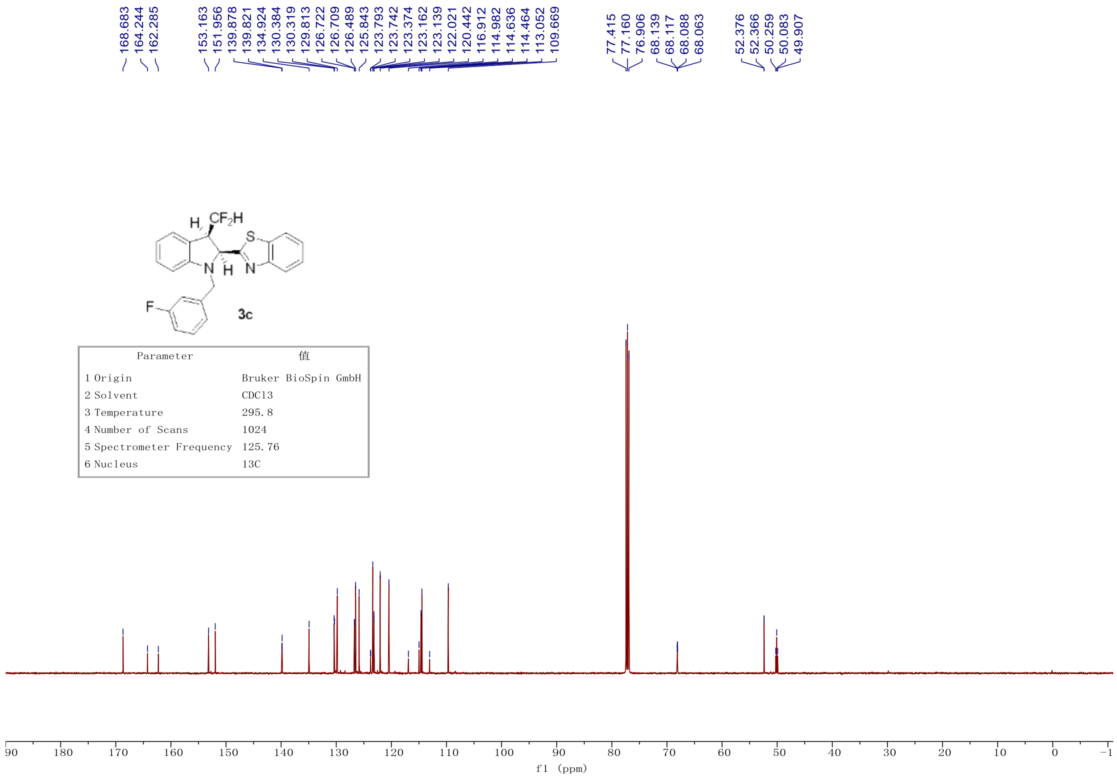

Figure S25. ^{13}C-NMR of **3c**

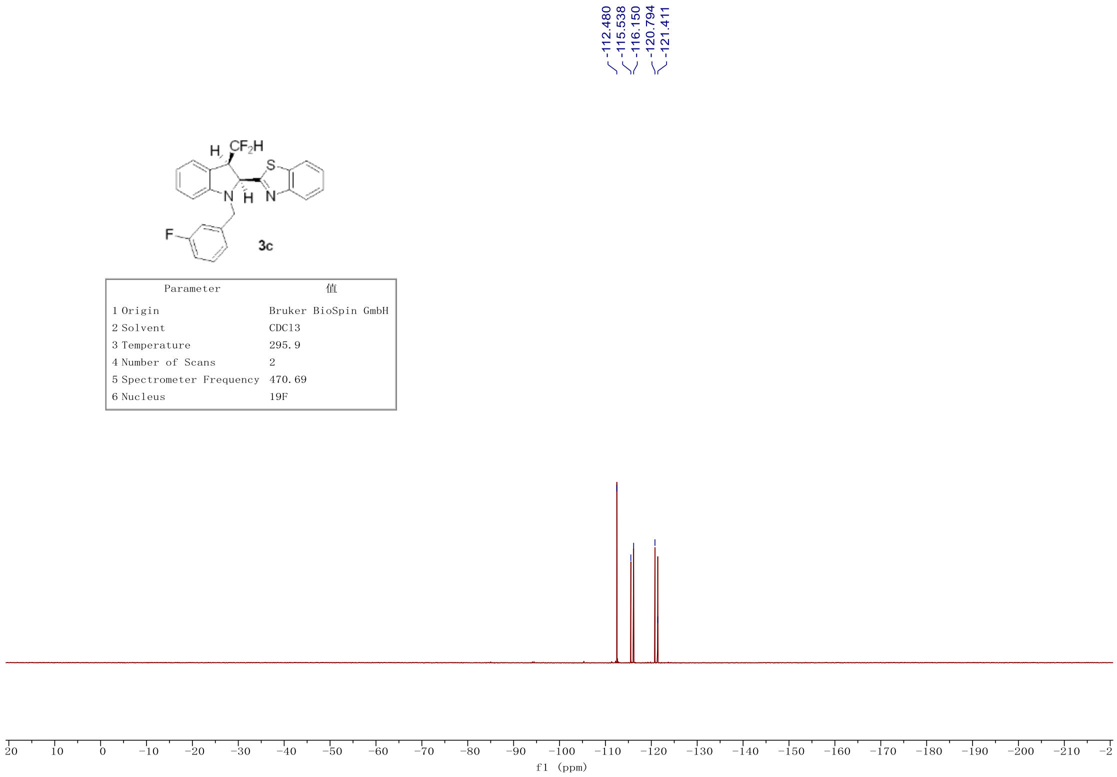

Figure S26. ^{19}F-NMR of **3c**

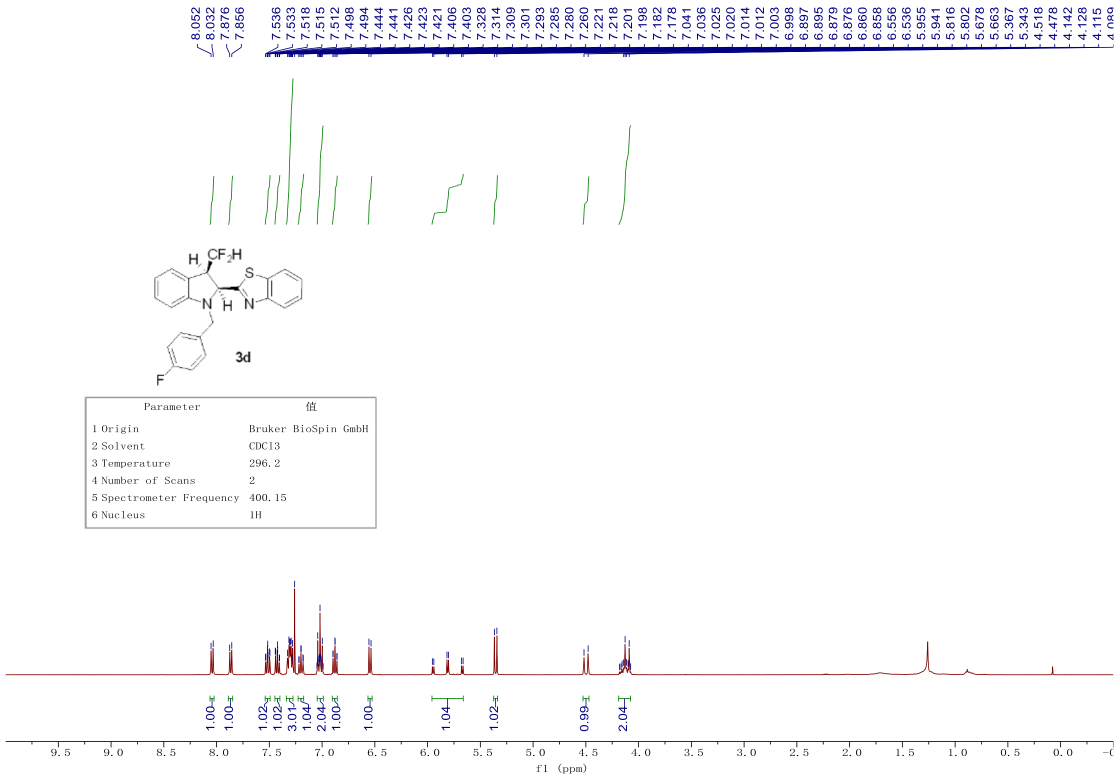

Figure S27. ^{1}H-NMR of **3d**

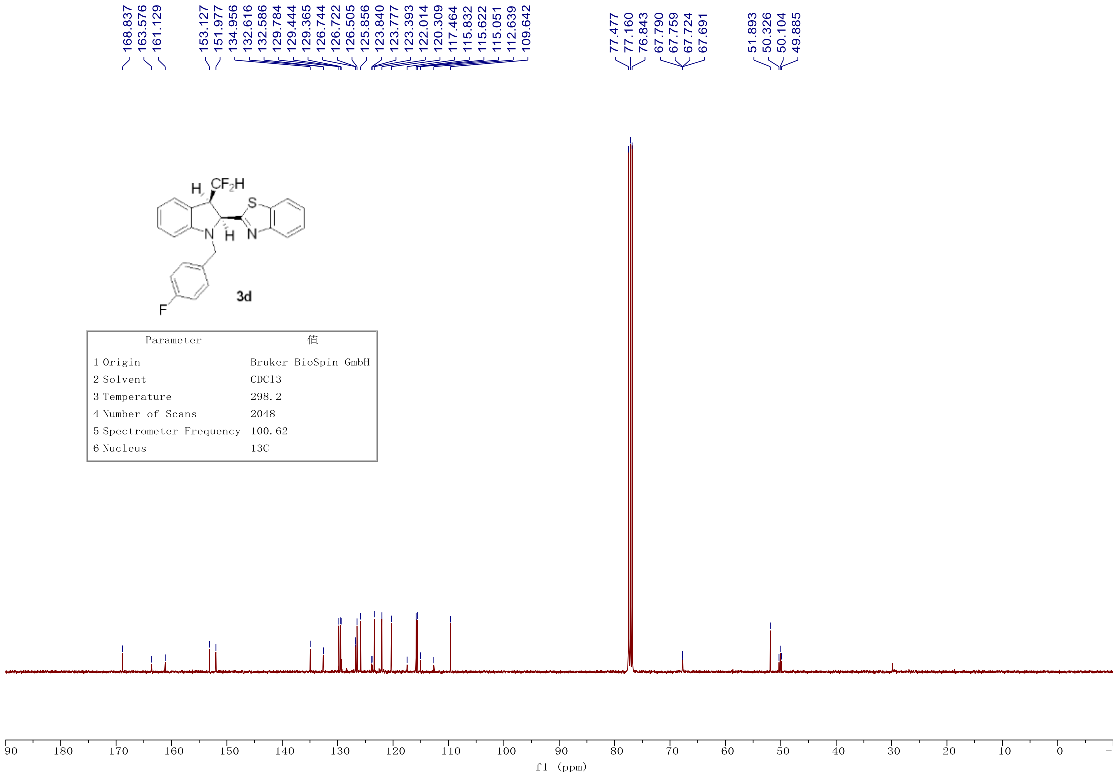

Figure S28. ^{13}C-NMR of **3d**

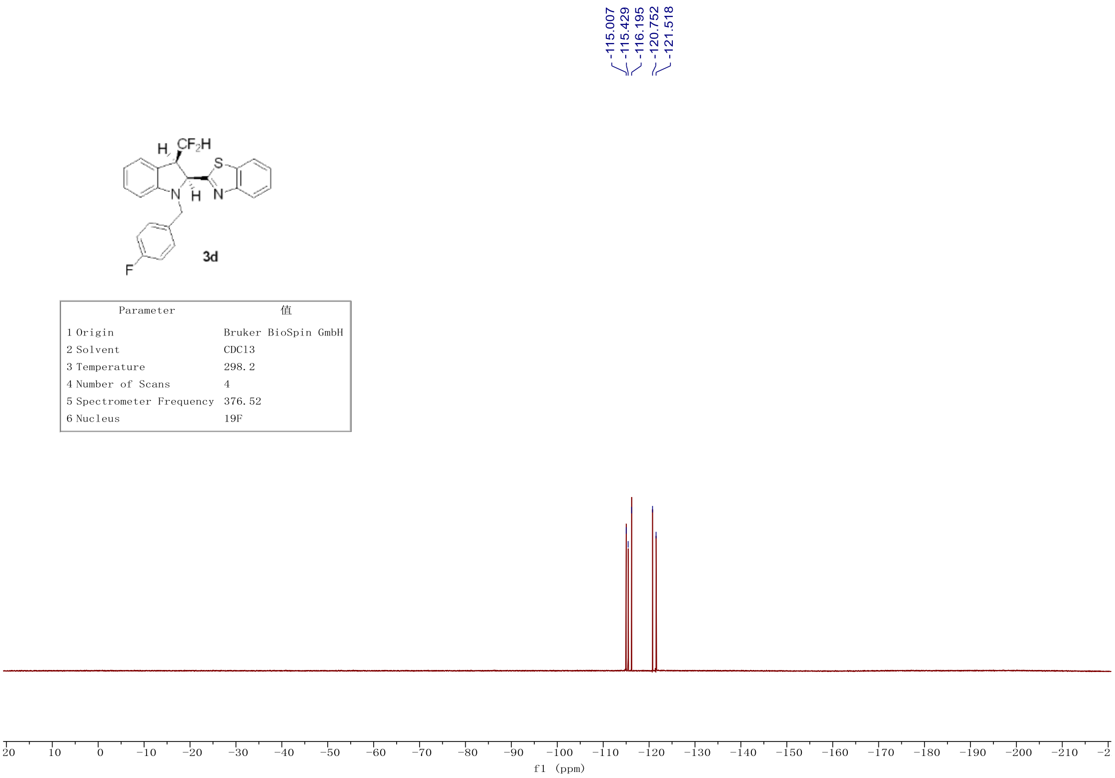

Figure S29. ^{19}F-NMR of **3d**

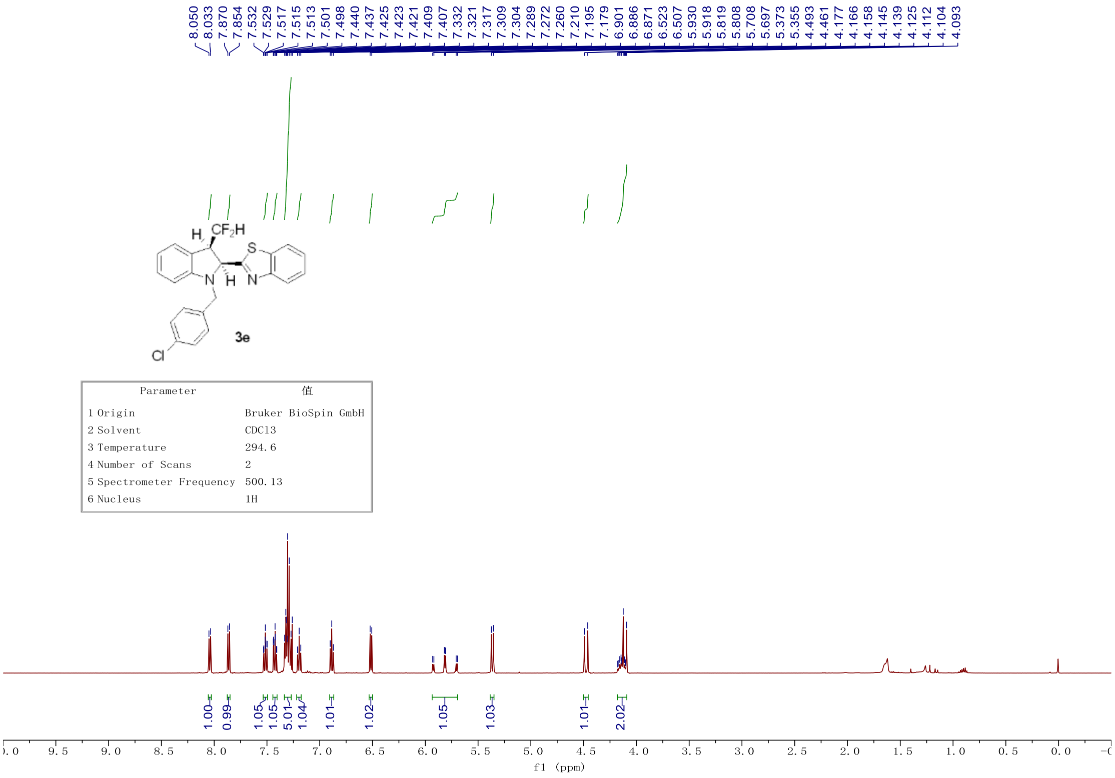

Figure S30. ^{1}H-NMR of **3e**

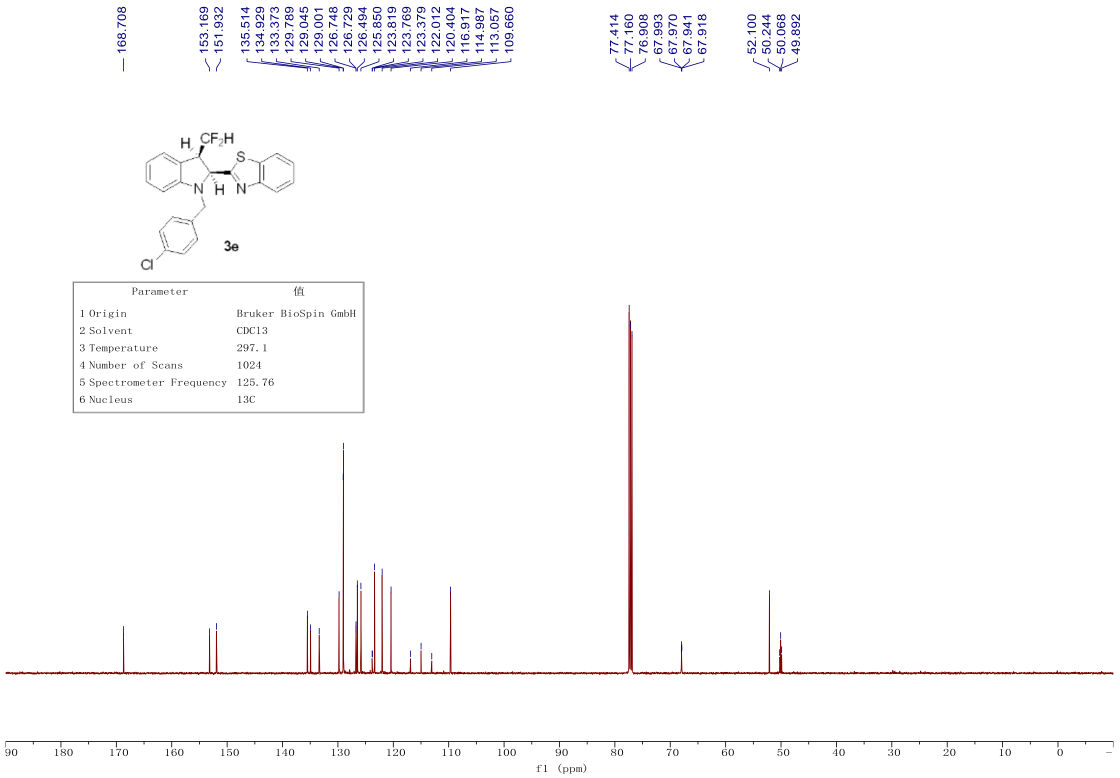

Figure S31. ^{13}C-NMR of **3e**

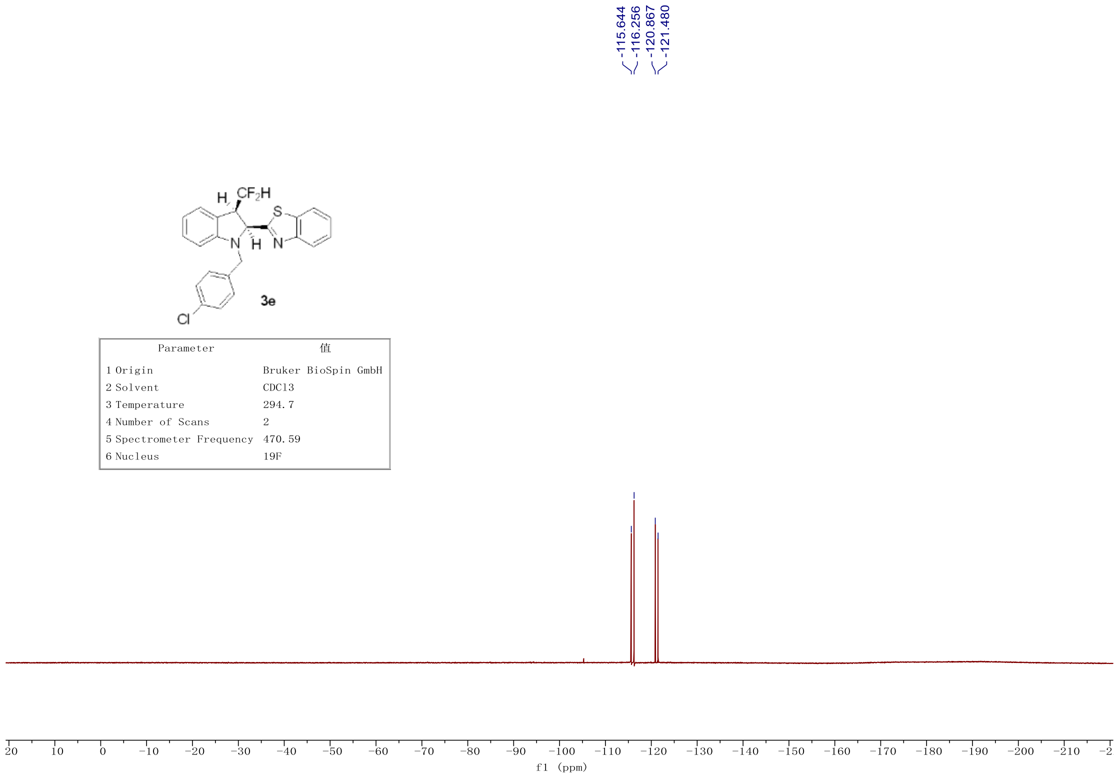

Figure S32. ^{19}F-NMR of **3e**

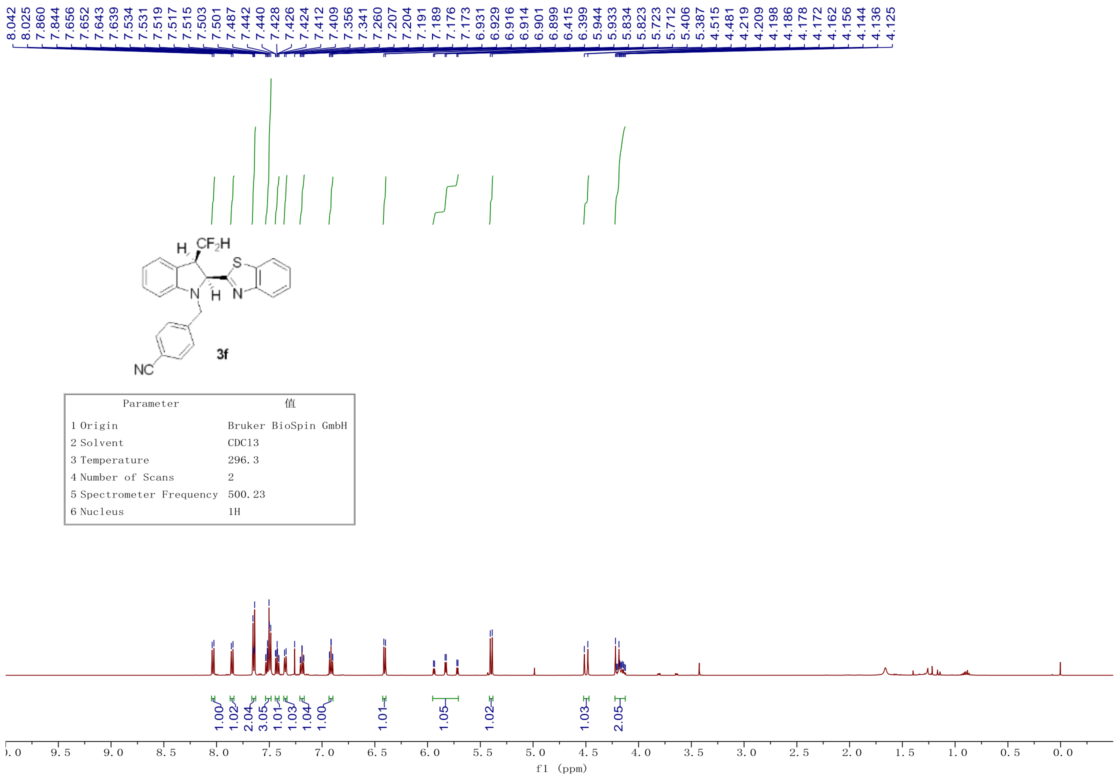

Figure S33. ^{1}H-NMR of **3f**

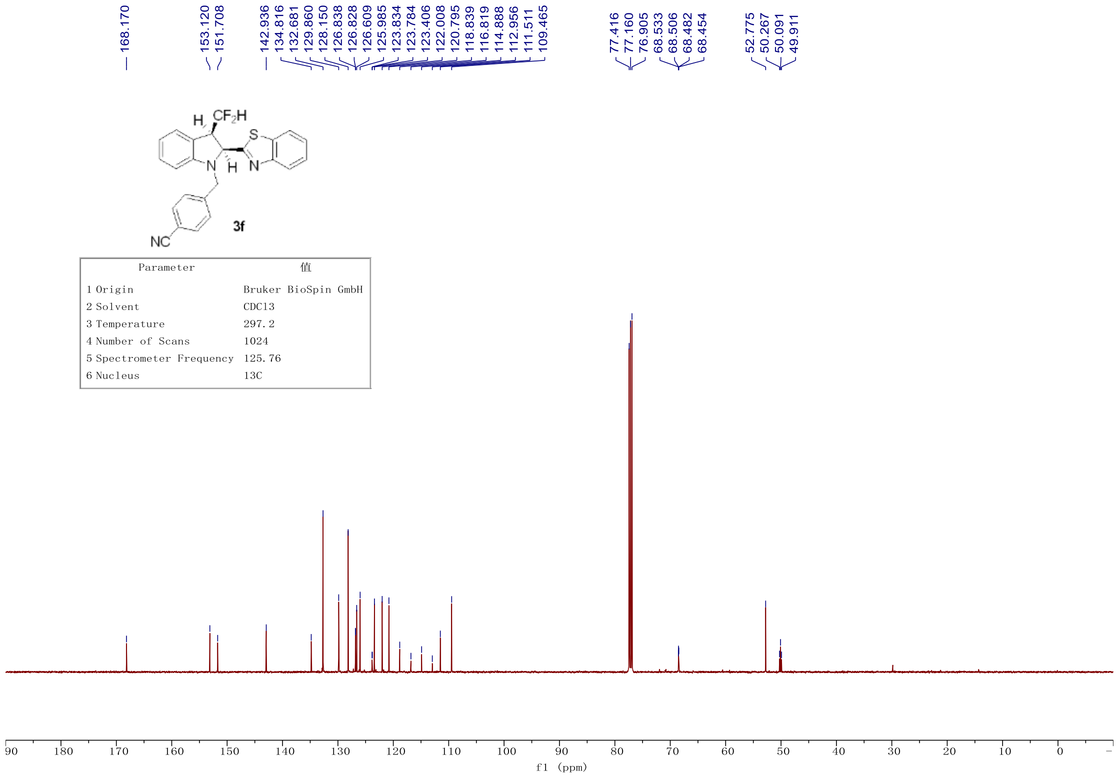

Figure S34. ^{13}C-NMR of **3f**

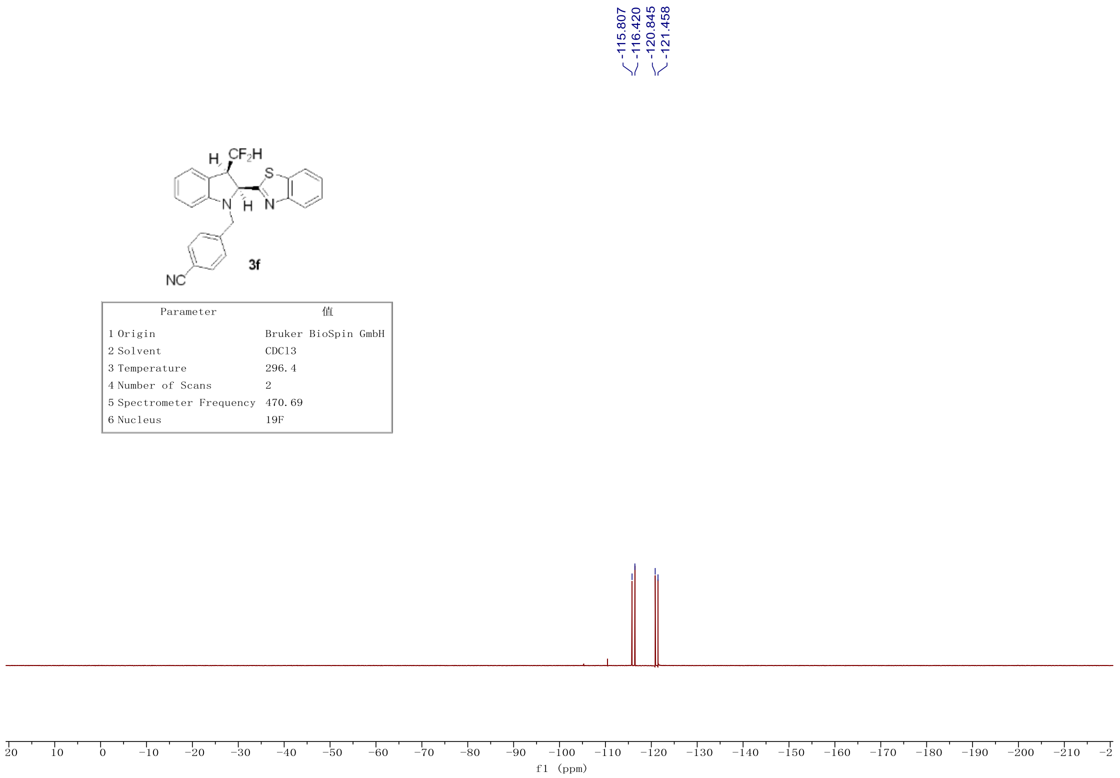

Figure S35. ^{19}F-NMR of **3f**

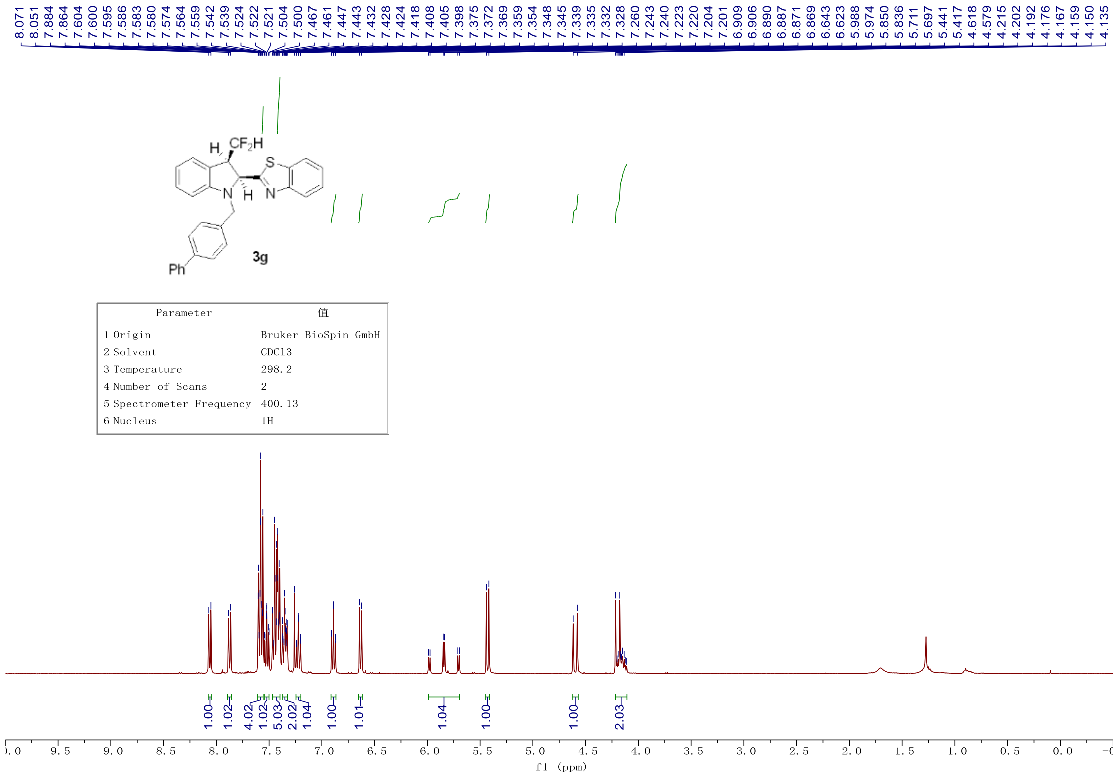

Figure S36. ^{1}H-NMR of **3g**

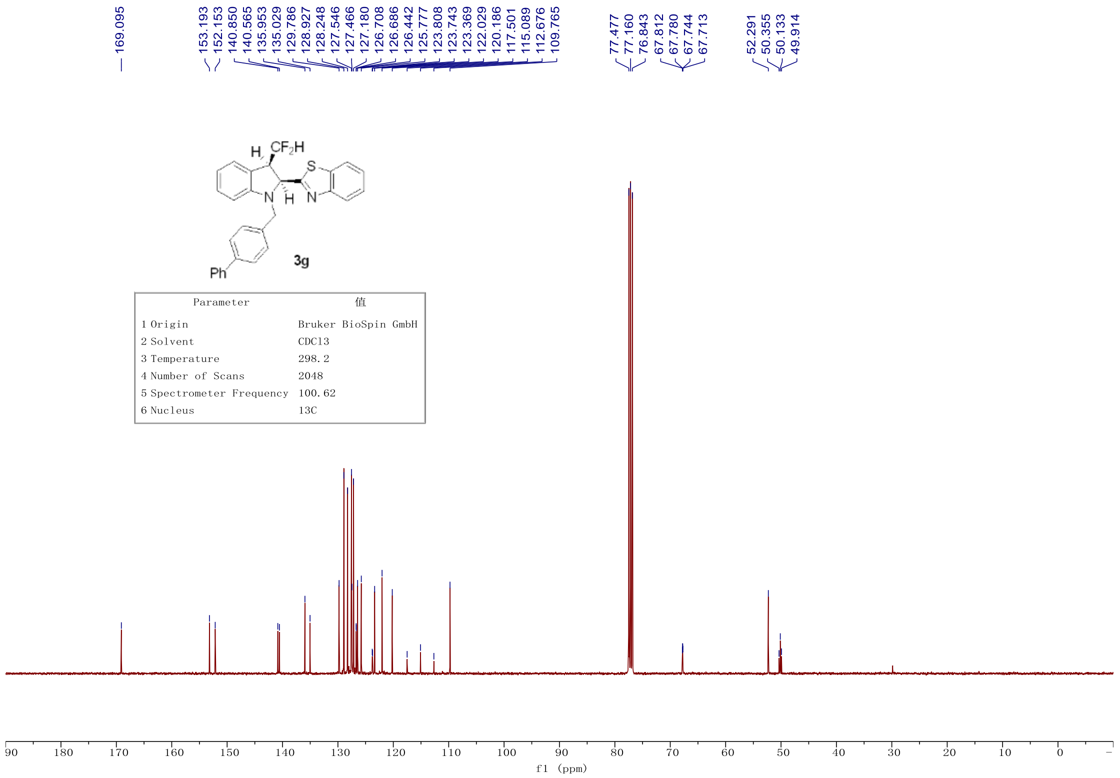

Figure S37. ^{13}C-NMR of **3g**

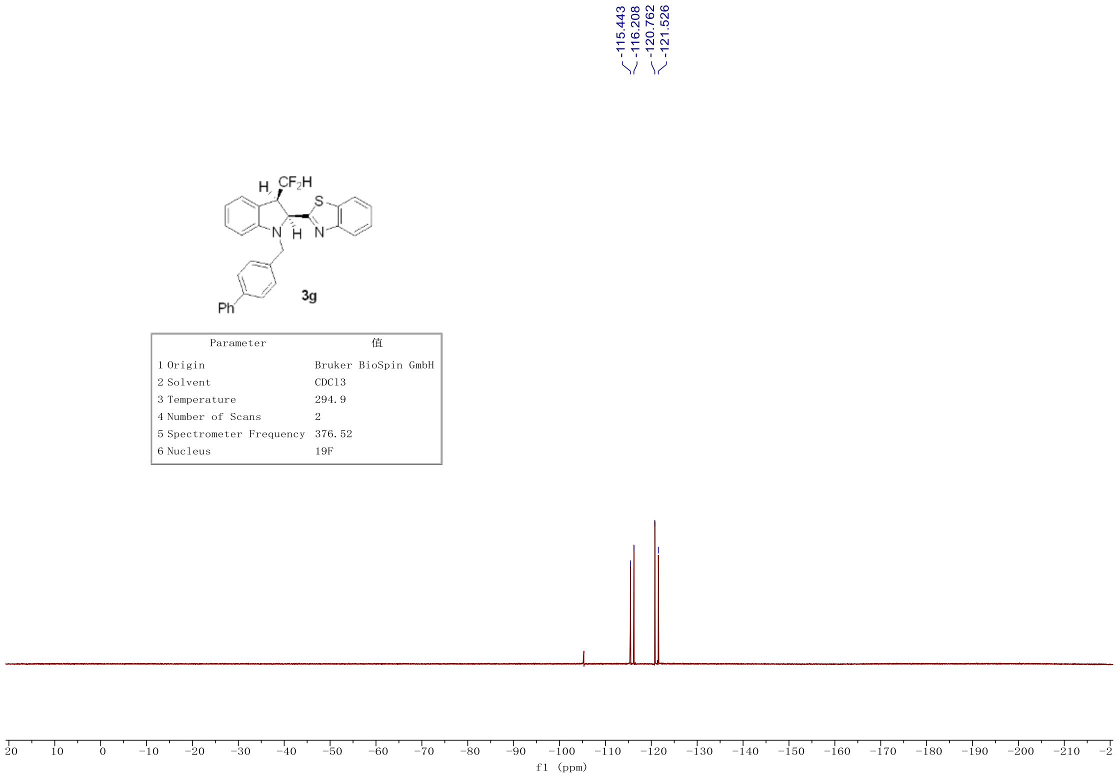

Figure S38. ^{19}F-NMR of **3g**

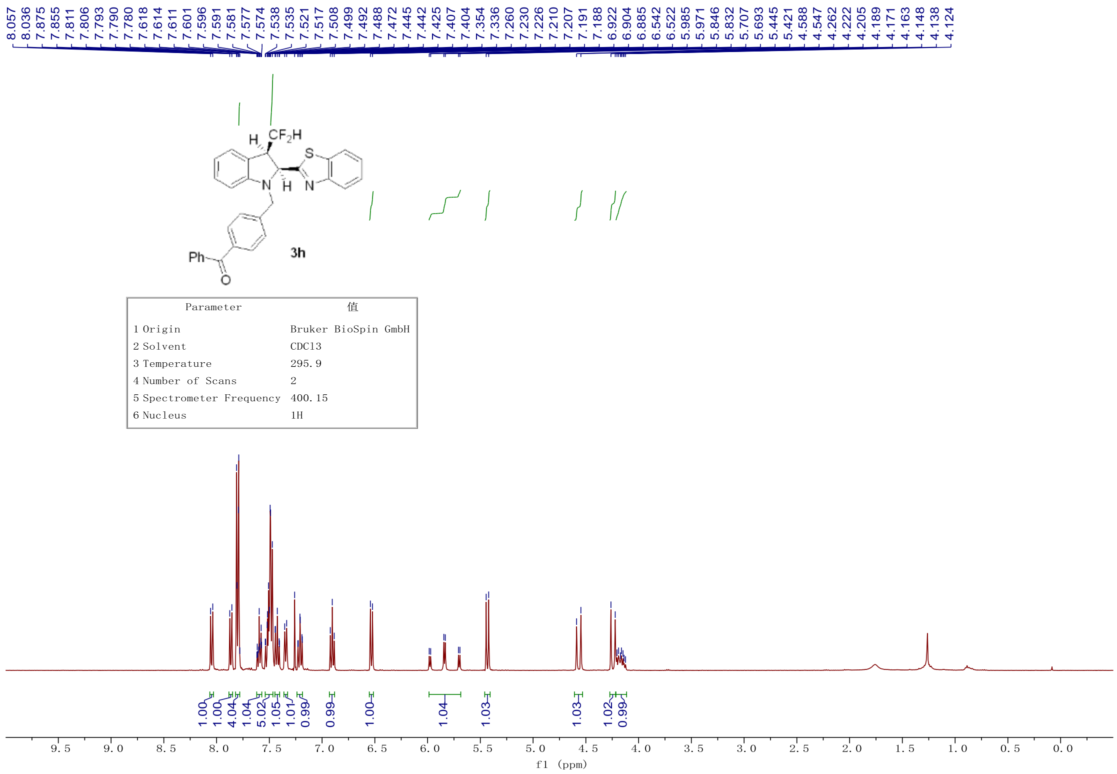

Figure S39. ^{1}H-NMR of **3h**

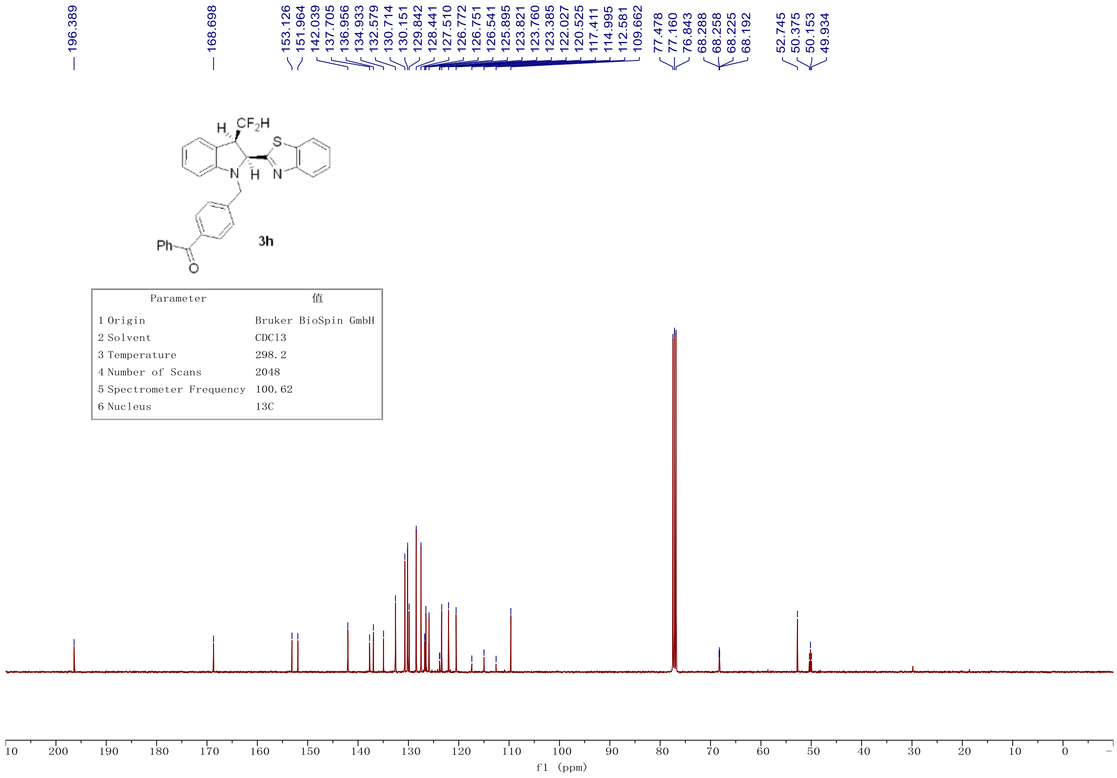

Figure S40. ^{13}C-NMR of **3h**

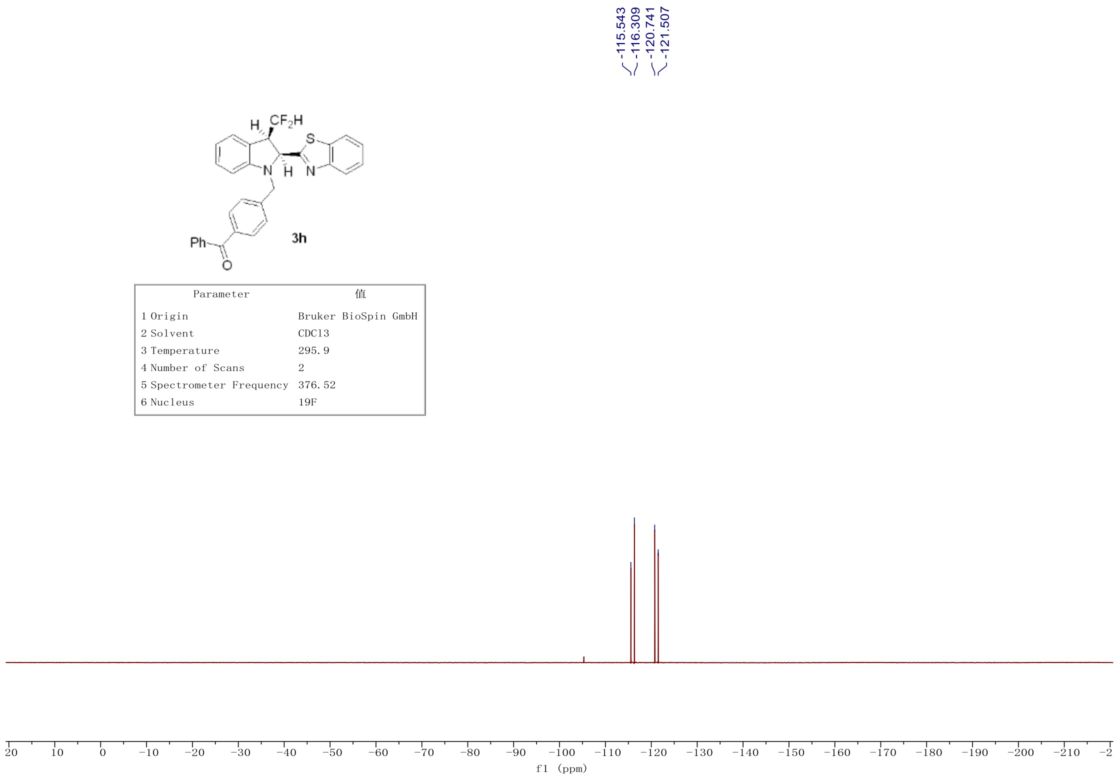

Figure S41. ^{19}F-NMR of **3h**

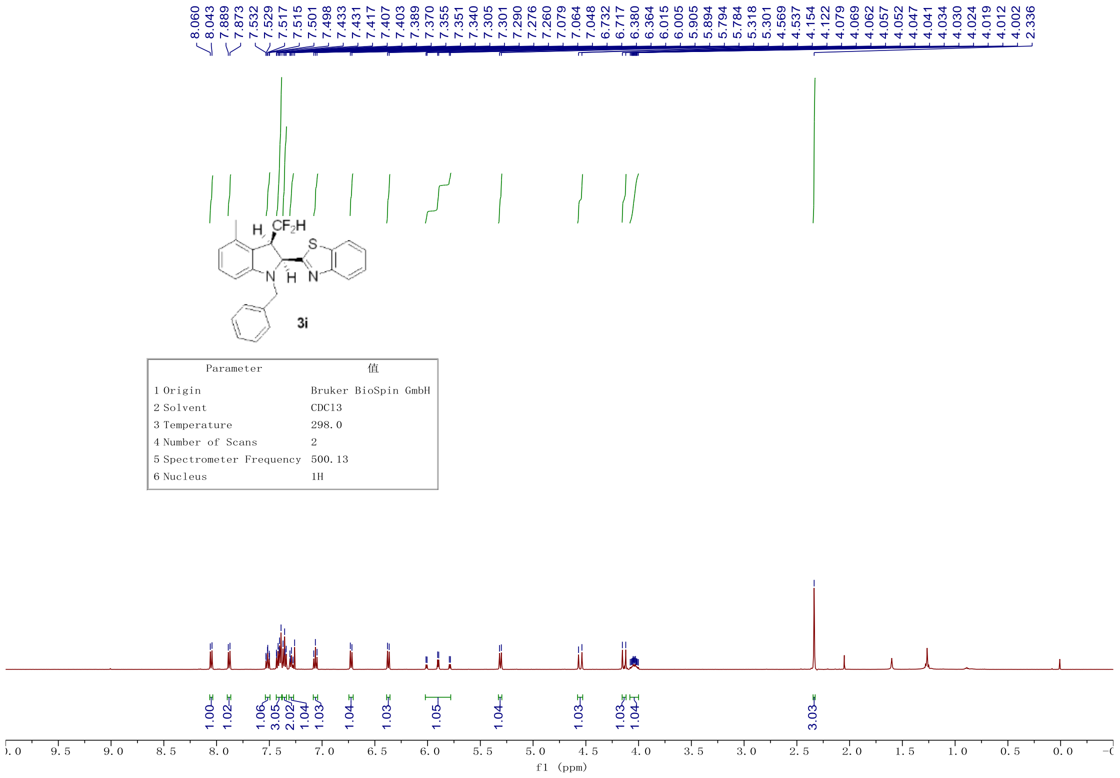

Figure S42. ^{1}H-NMR of **3i**

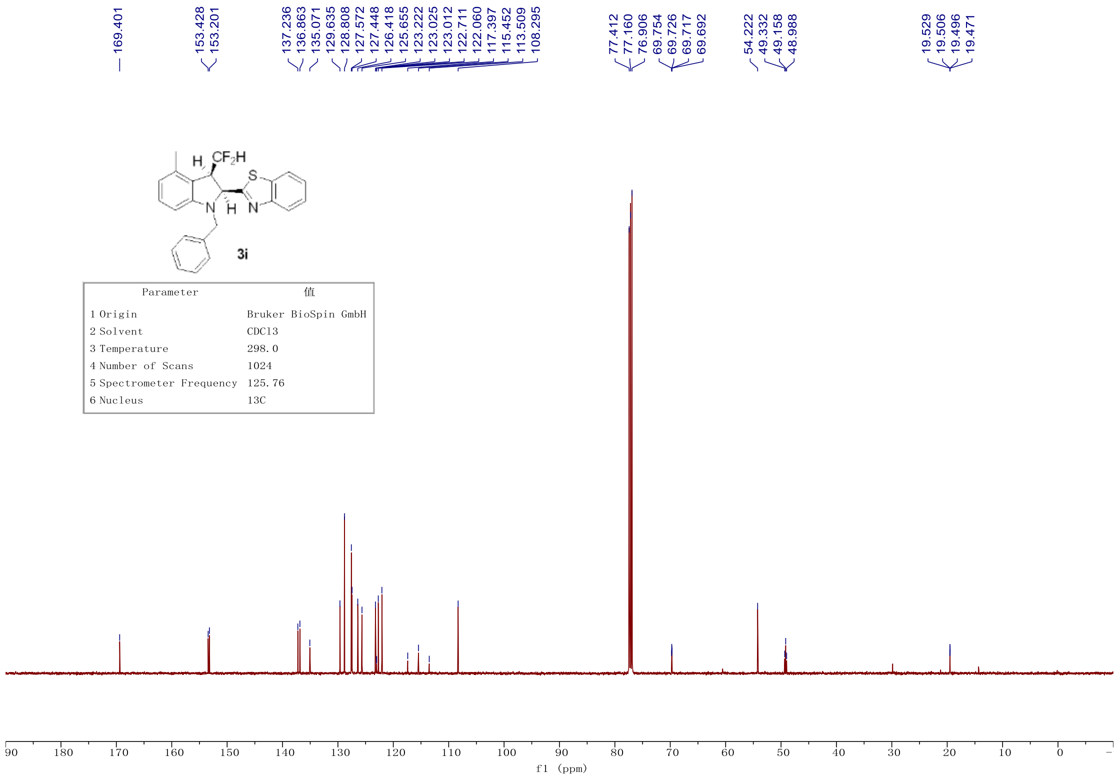

Figure S43. ^{13}C-NMR of **3i**

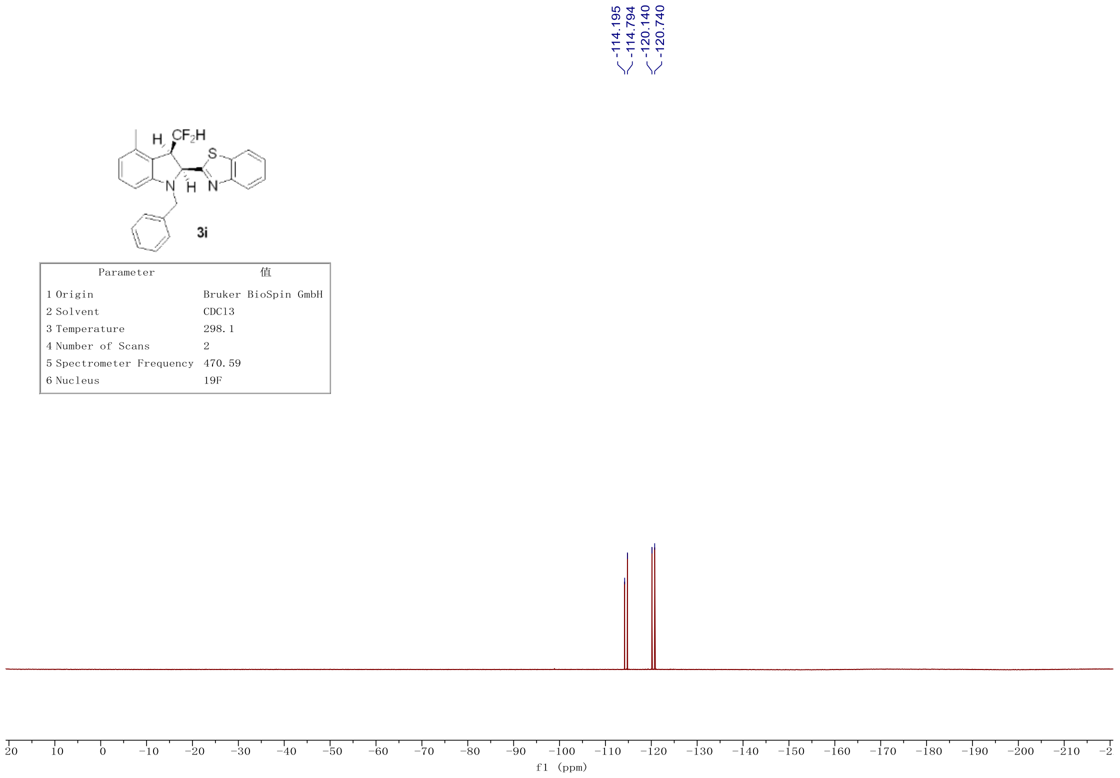

Figure S44. ^{19}F-NMR of **3i**

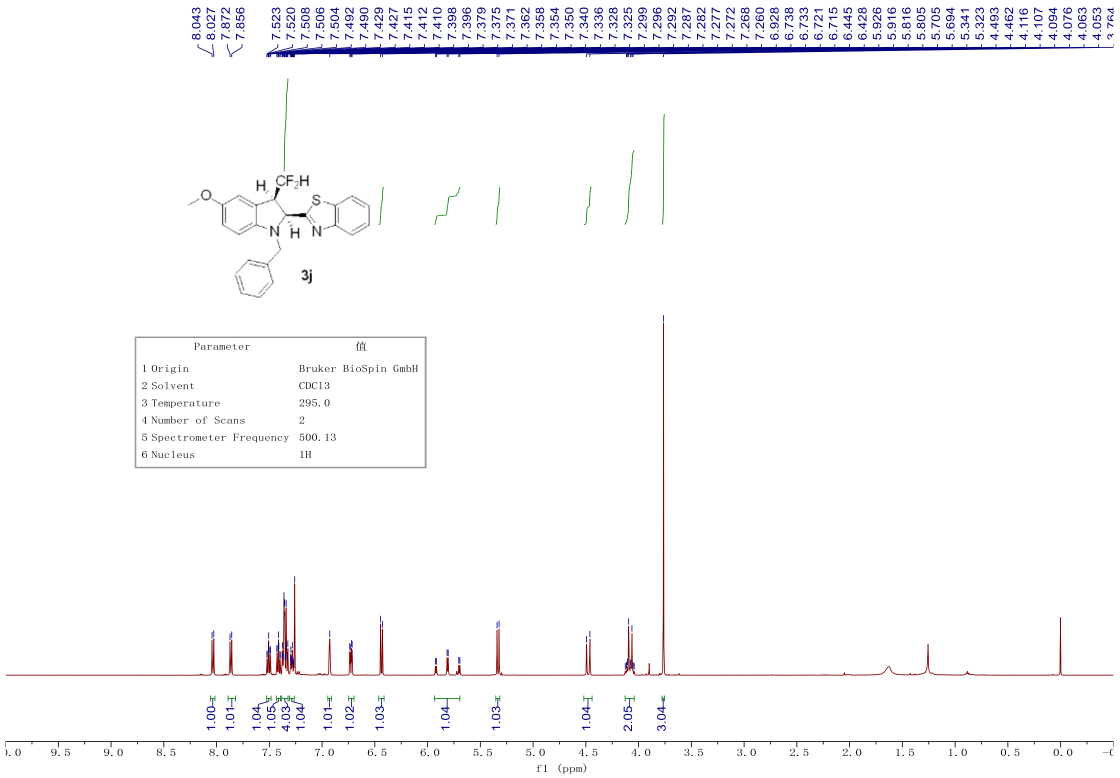

Figure S45. ^{1}H-NMR of **3j**

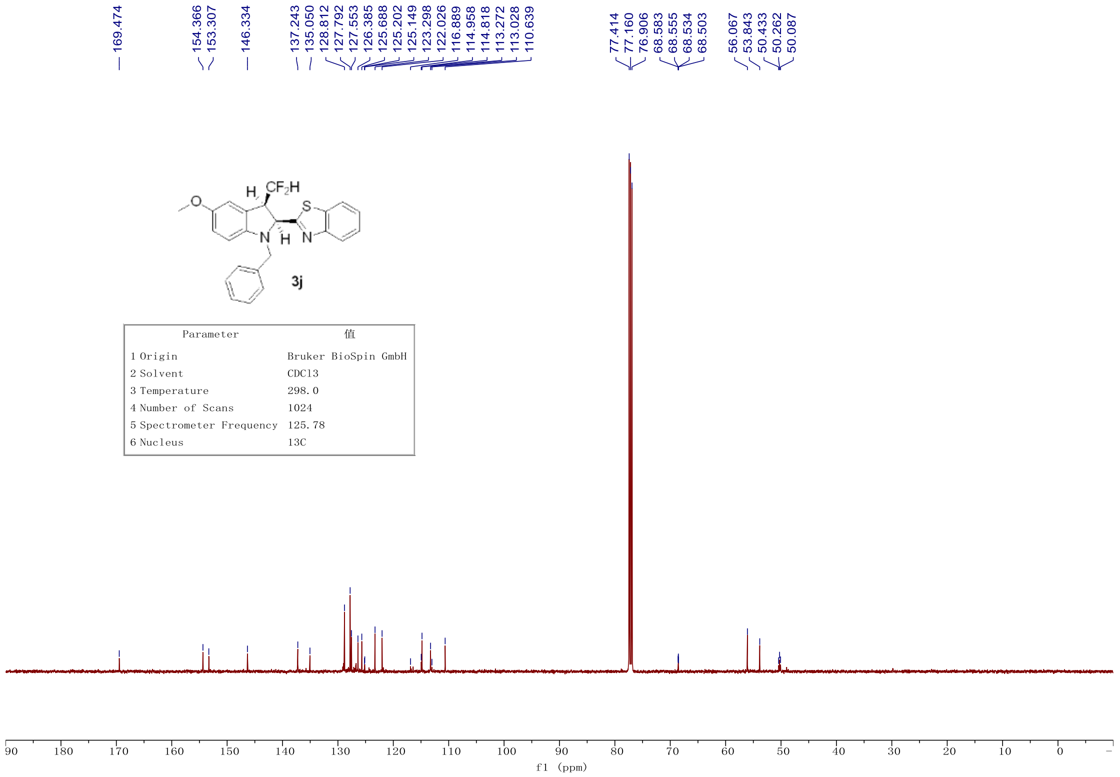

Figure S46. ^{13}C-NMR of **3j**

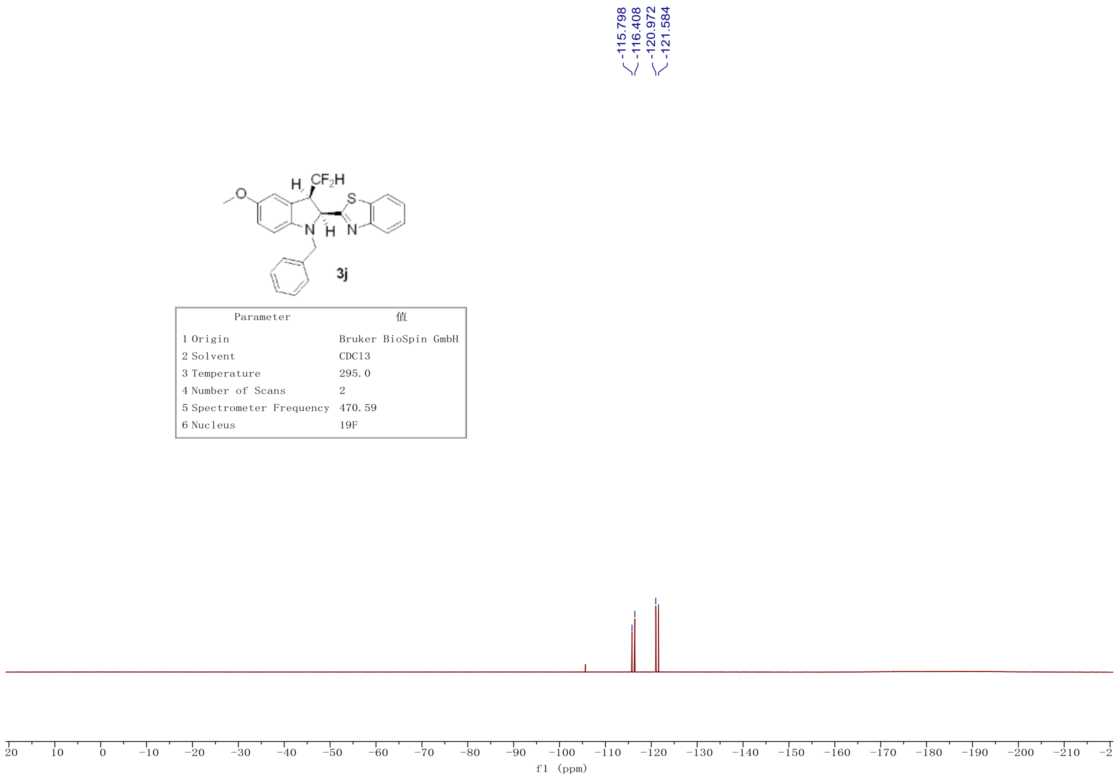

Figure S47. ^{19}F-NMR of **3j**

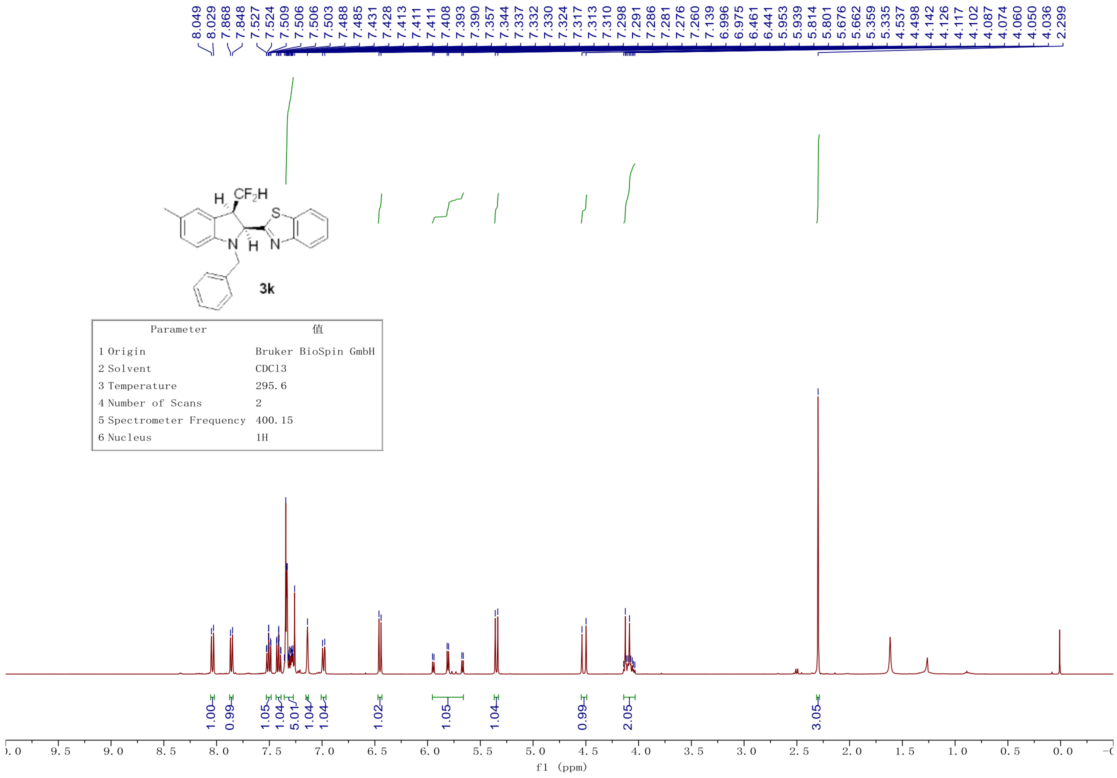

Figure S48. ^{1}H-NMR of **3k**

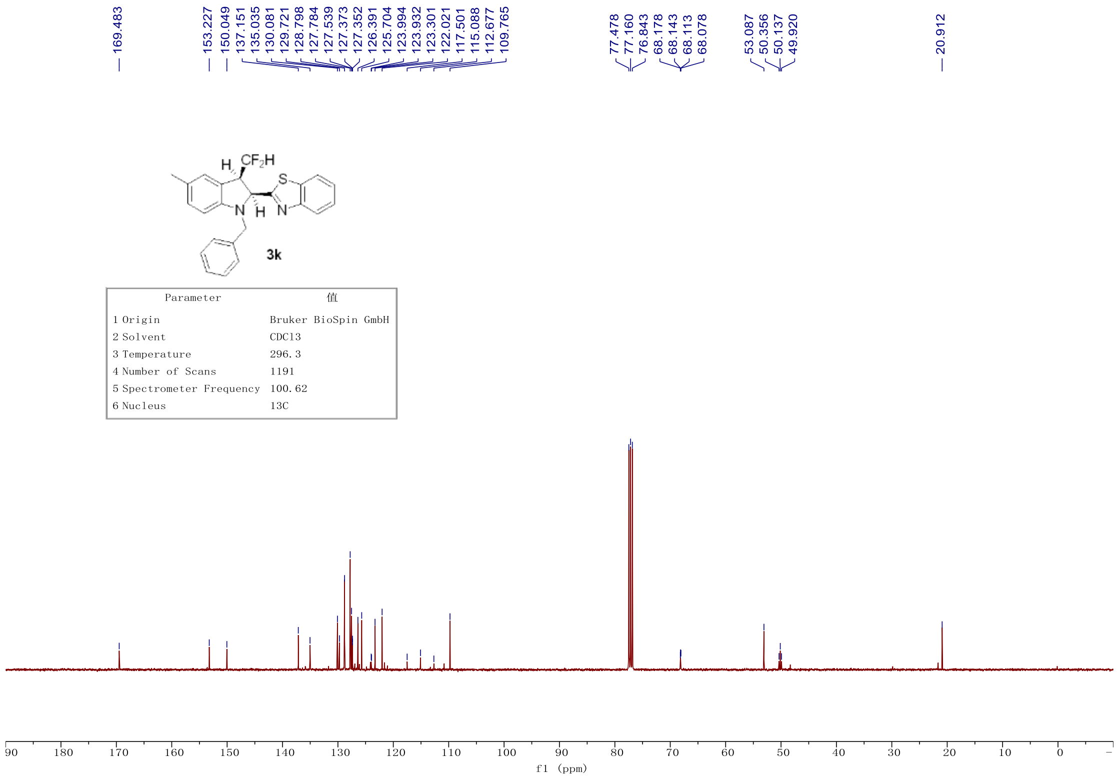

Figure S49. ^{13}C-NMR of **3k**

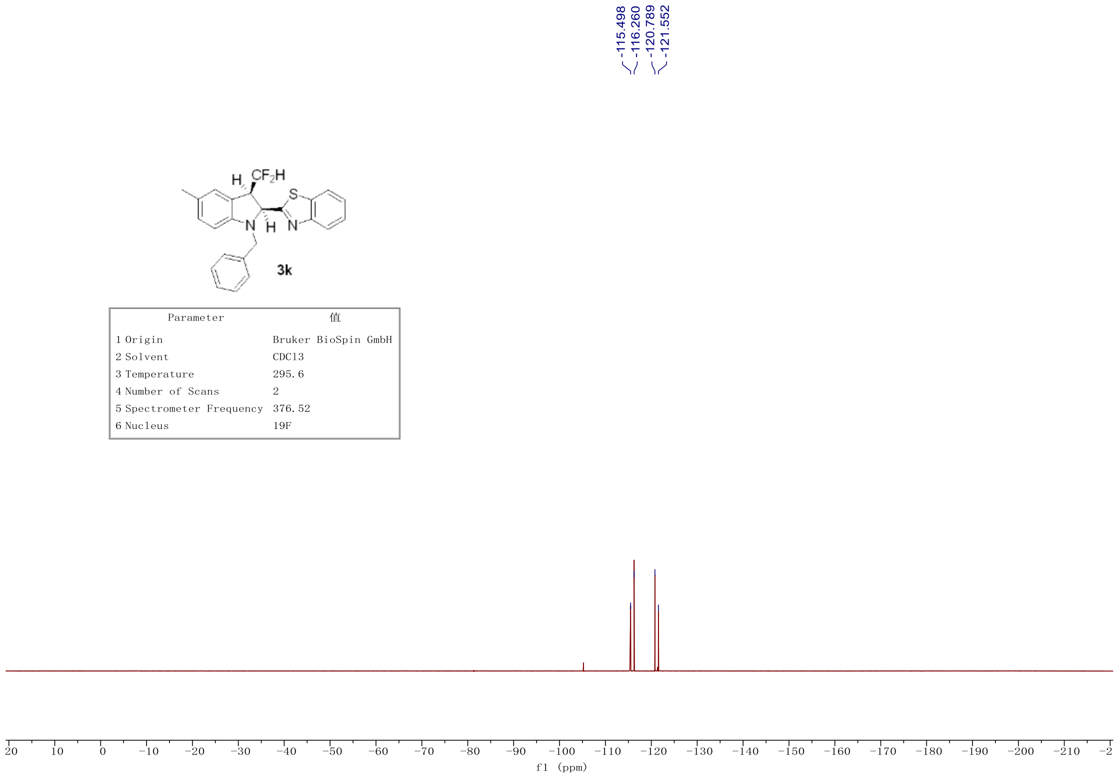

Figure S50. ^{19}F-NMR of **3k**

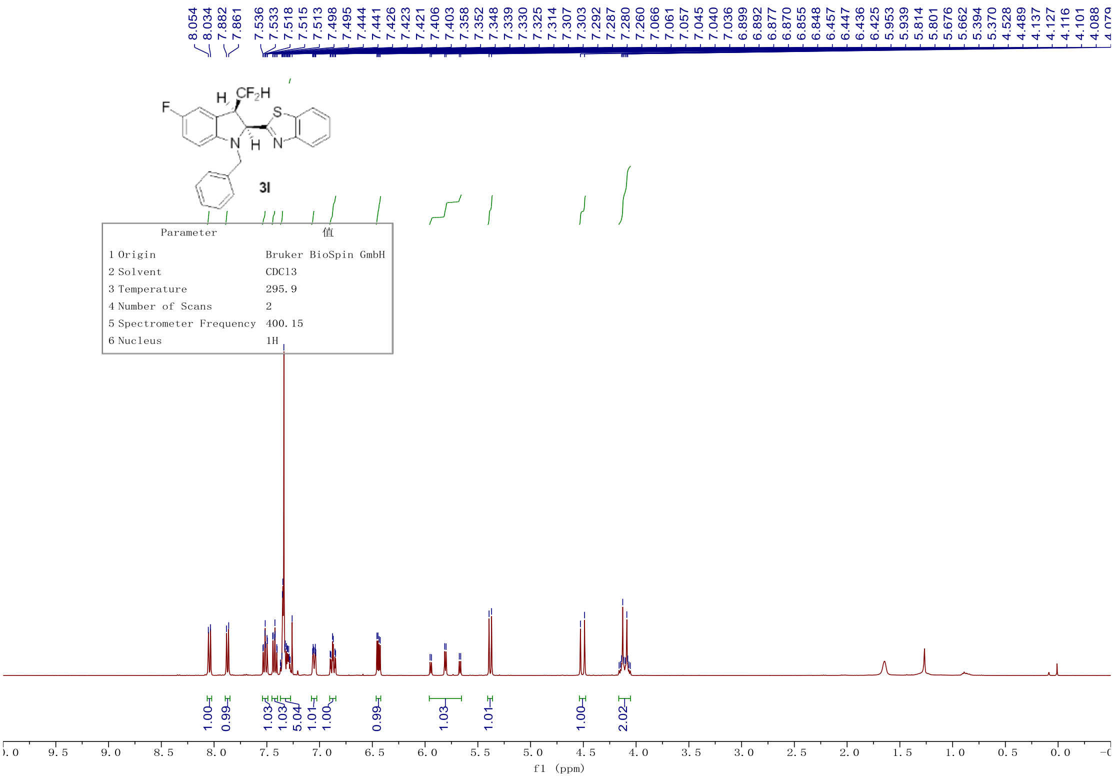

Figure S51. ^{1}H-NMR of **3l**

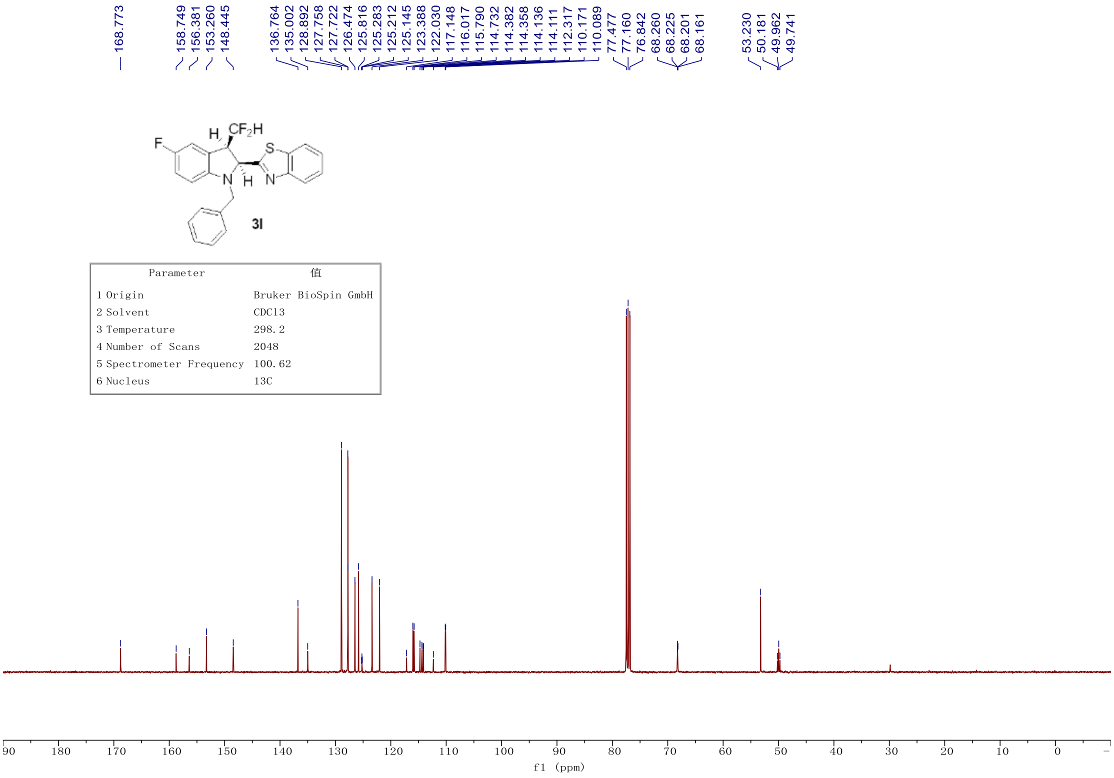

Figure S52. ^{13}C-NMR of **3l**

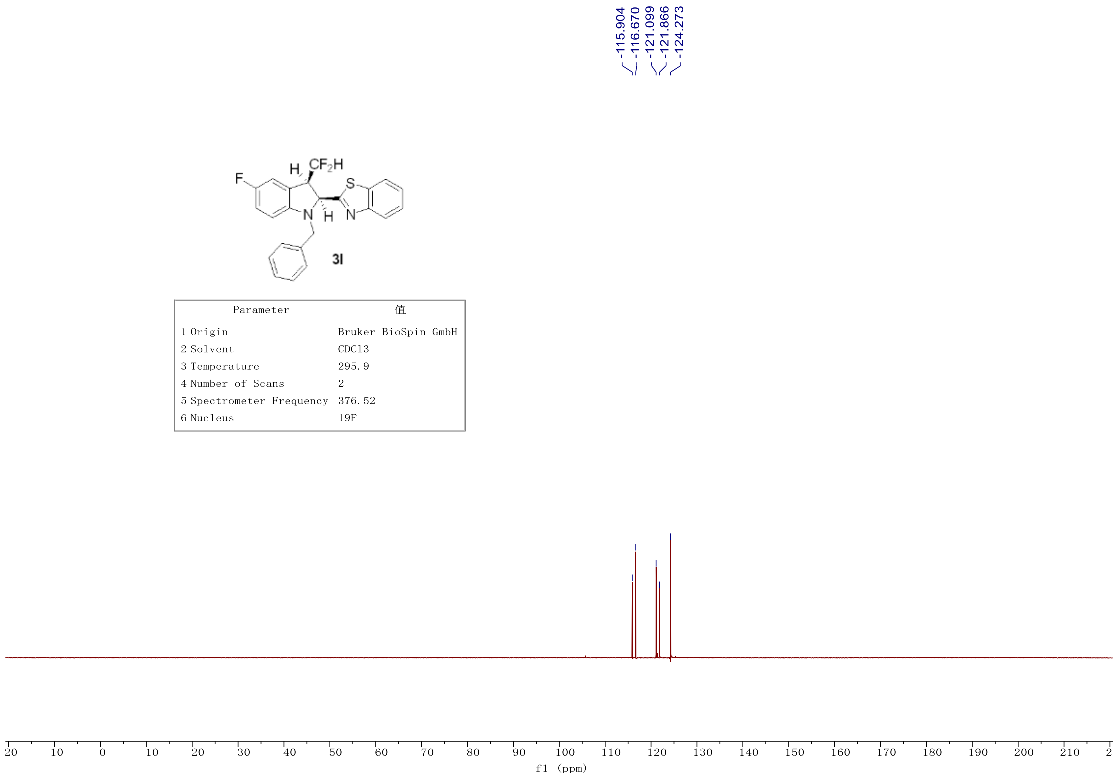

Figure S53. ^{19}F-NMR of **3l**

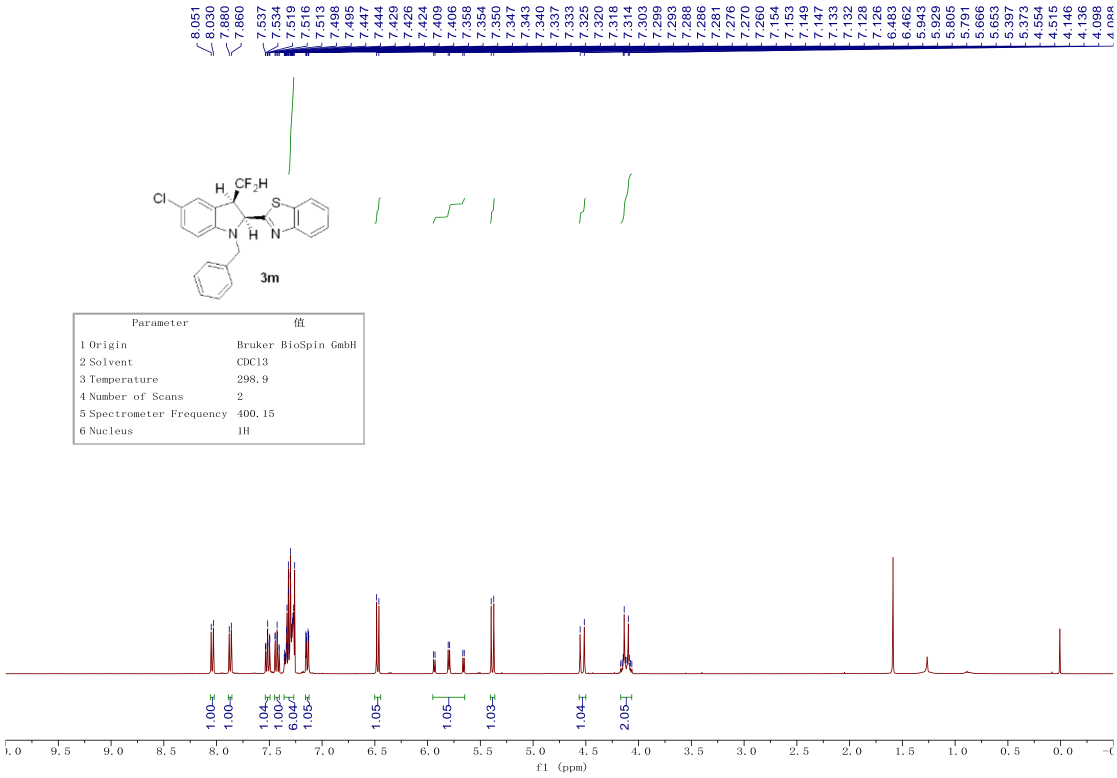

Figure S54. ^{1}H-NMR of **3m**

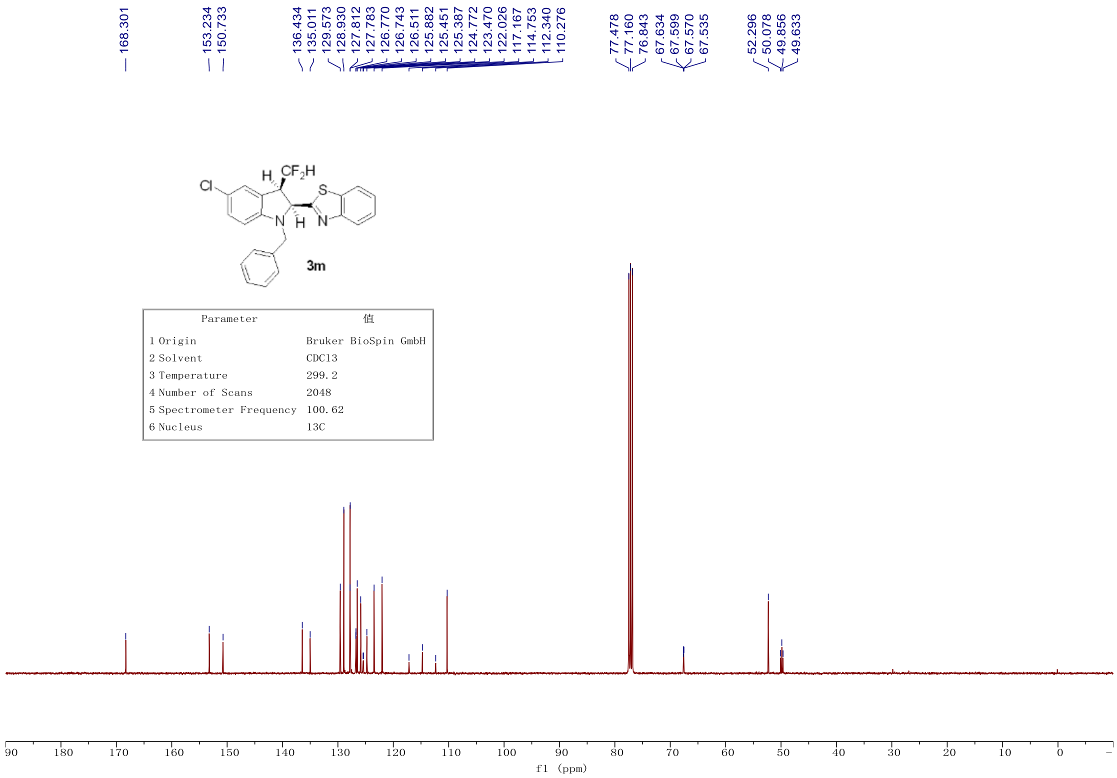

Figure S55. ^{13}C-NMR of **3m**

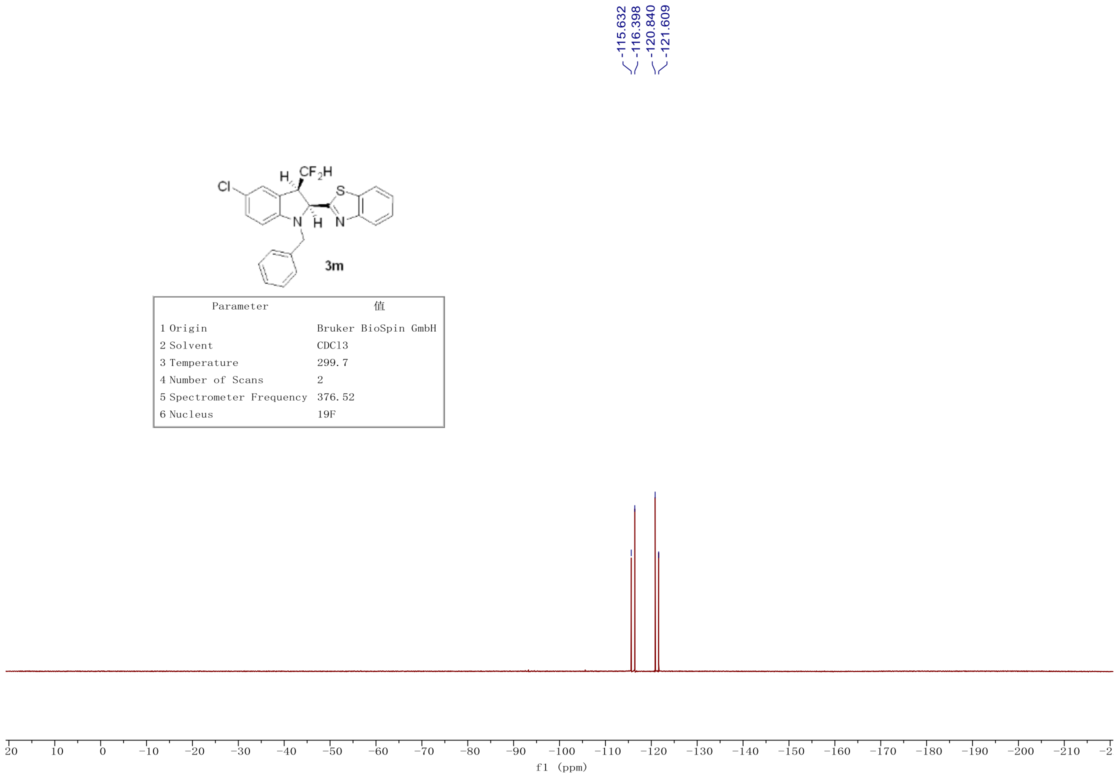

Figure S56. ^{19}F-NMR of **3m**

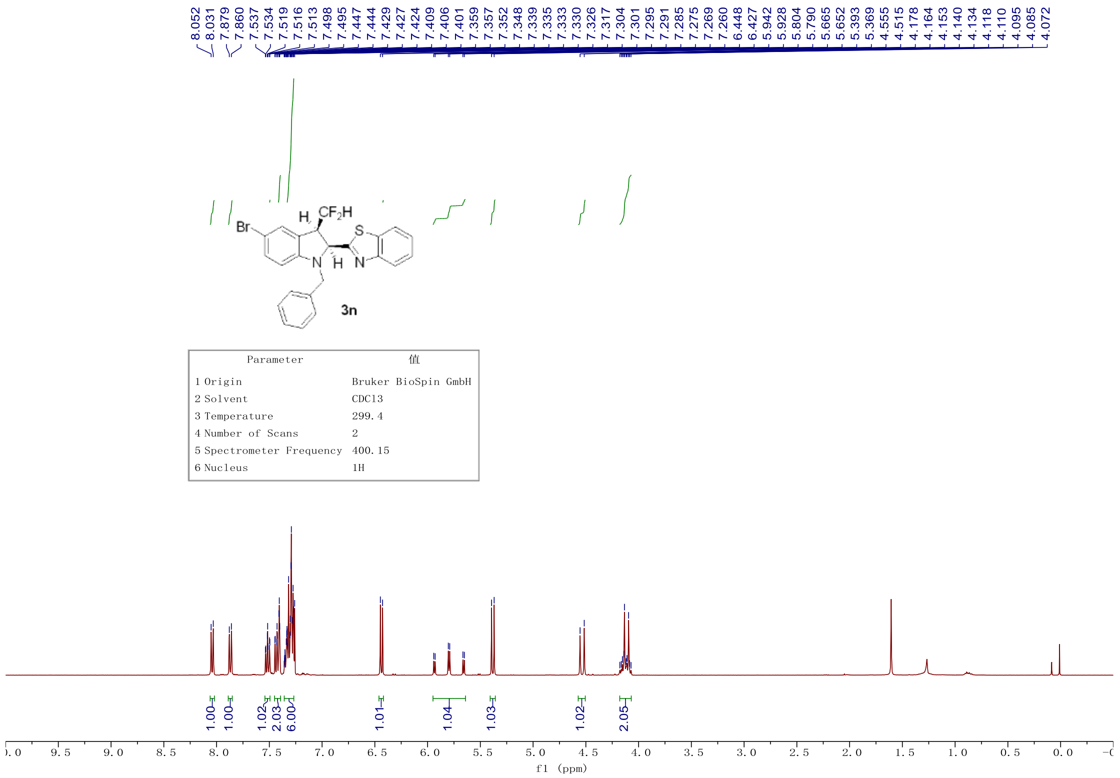

Figure S57. ^{1}H-NMR of **3n**

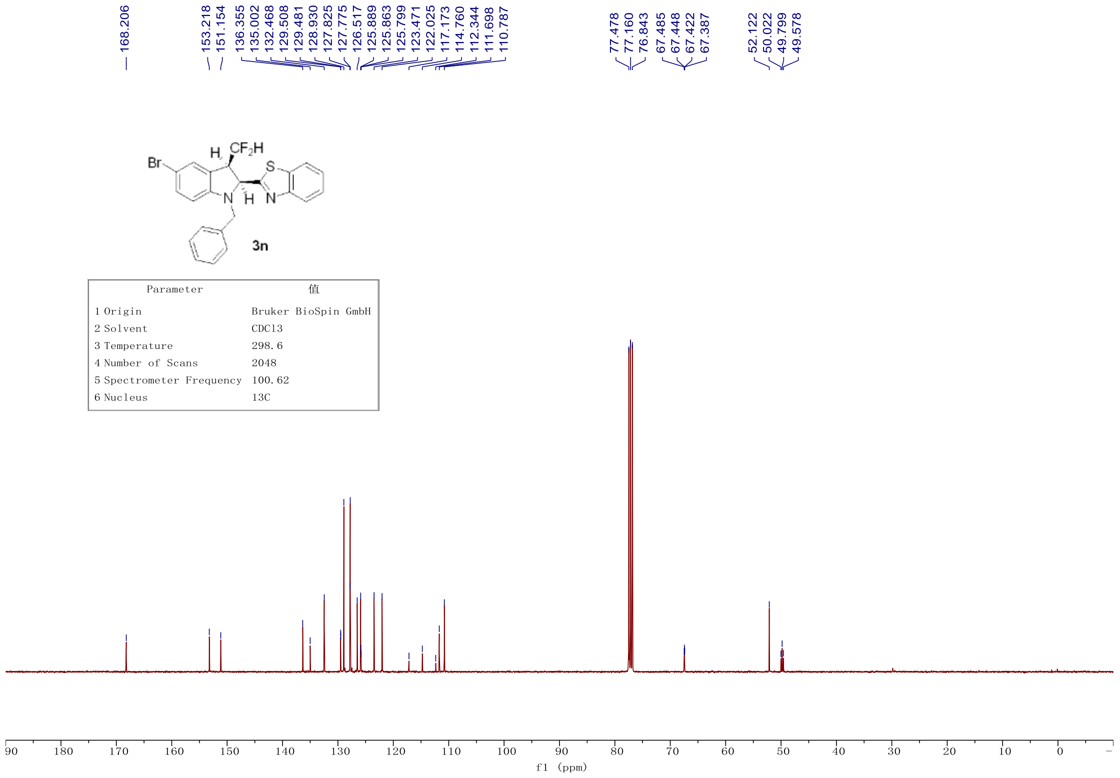

Figure S58. ^{13}C-NMR of **3n**

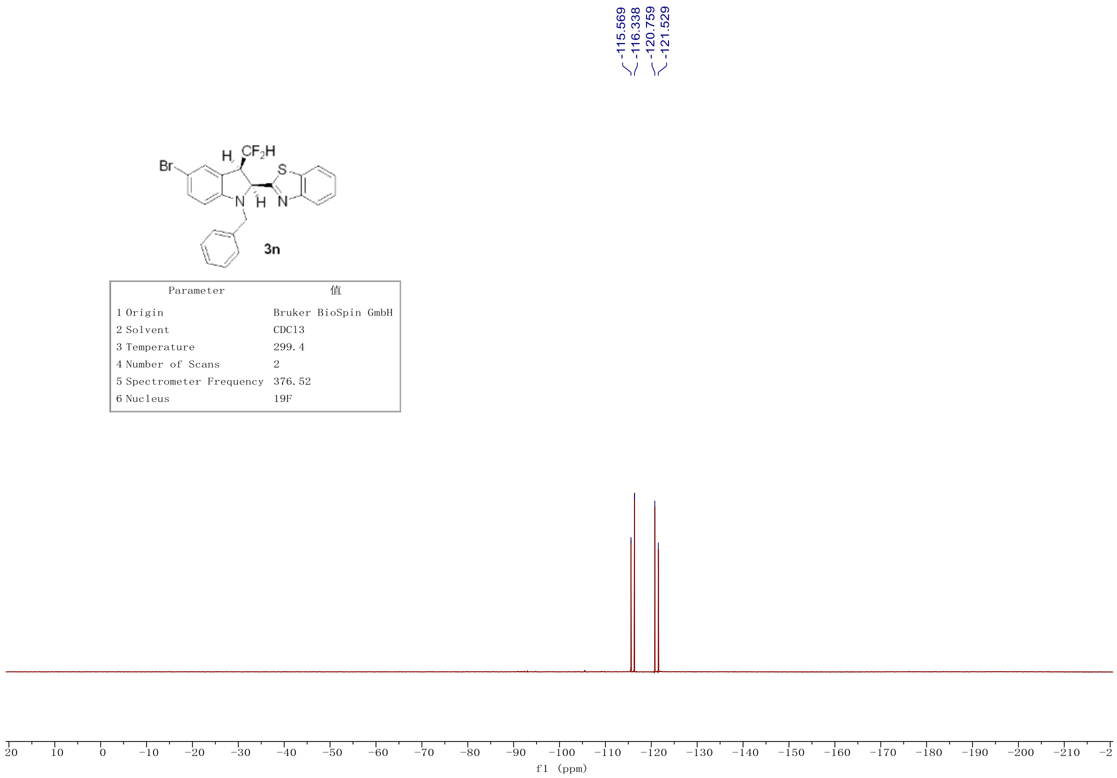

Figure S59. ^{19}F-NMR of **3n**

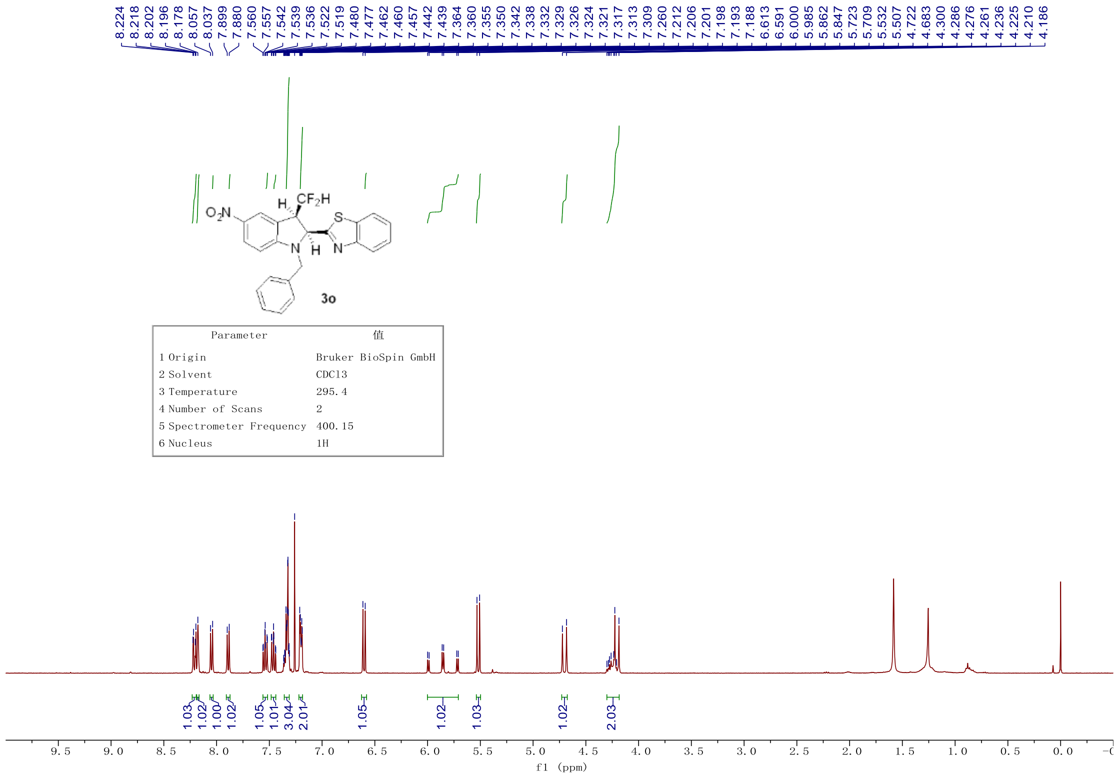

Figure S60. ^{1}H-NMR of **3o**

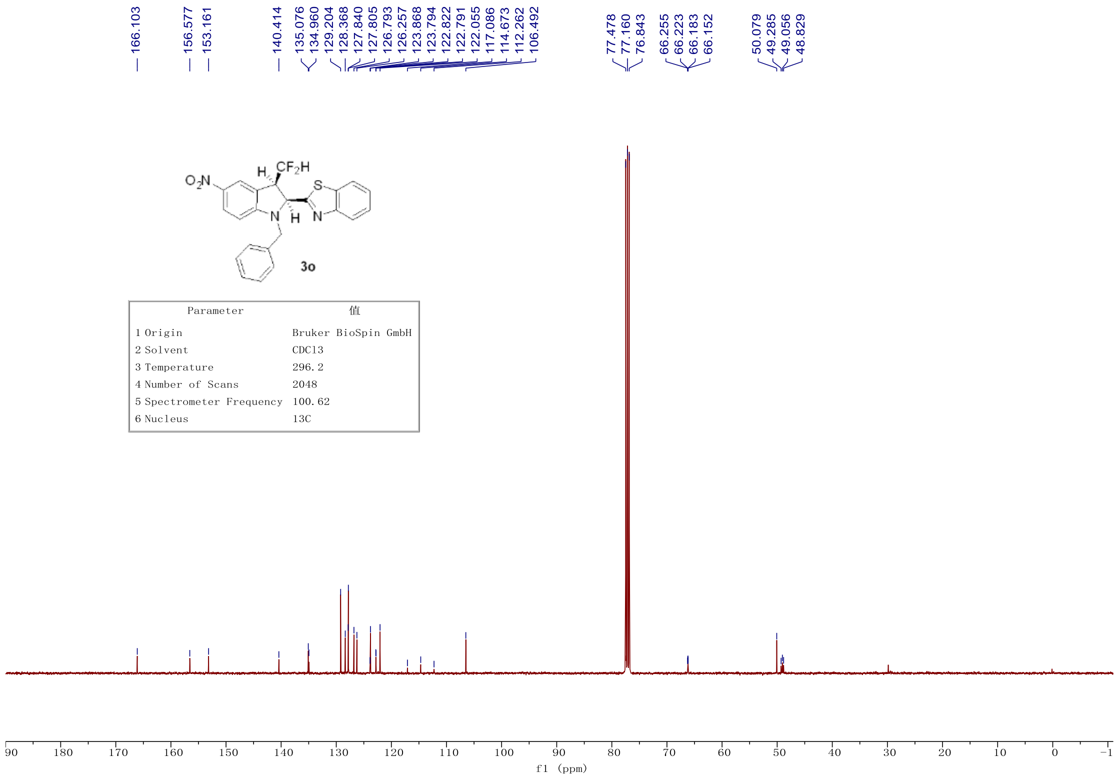

Figure S61. ^{13}C-NMR of **3o**

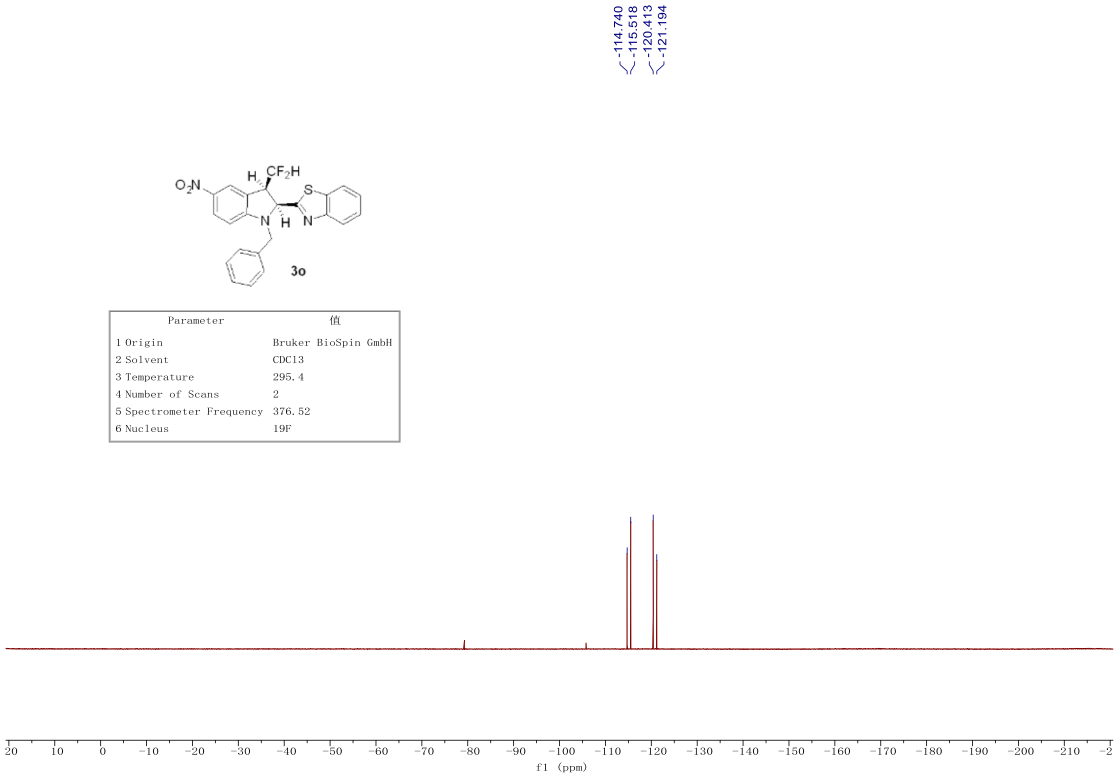

Figure S62. ^{19}F-NMR of **3o**

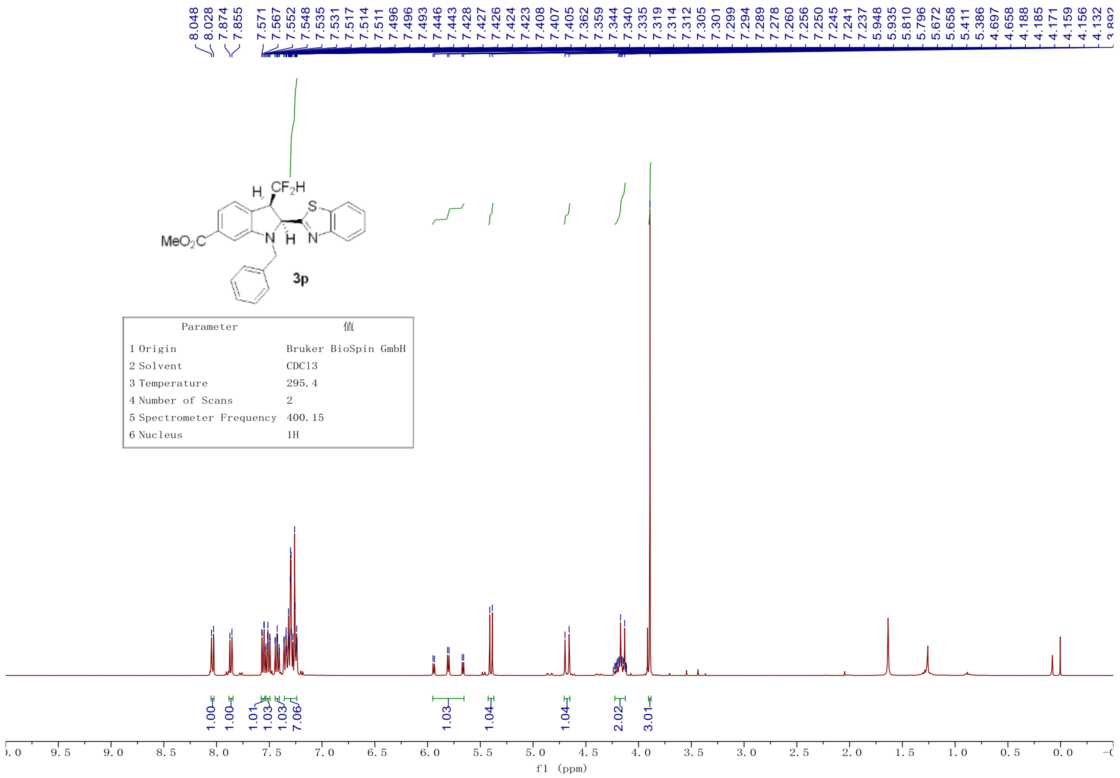

Figure S63. ^{1}H-NMR of **3p**

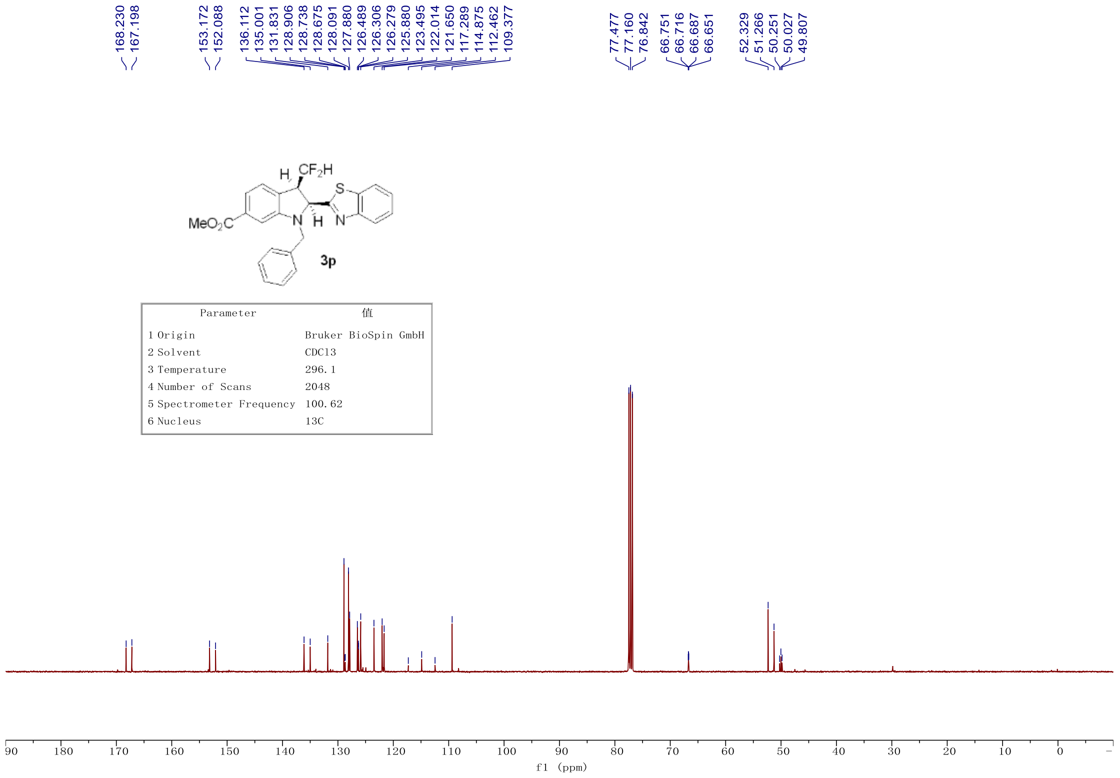

Figure S64. ^{13}C-NMR of **3p**

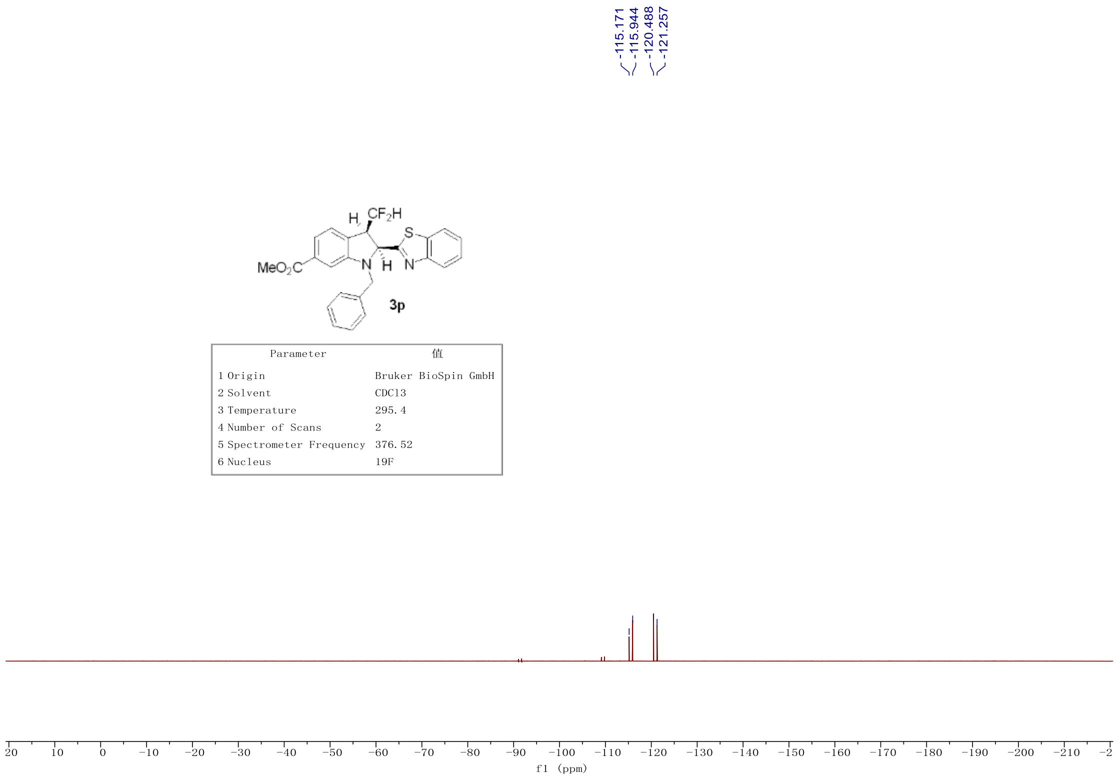

Figure S65. ^{19}F-NMR of **3p**

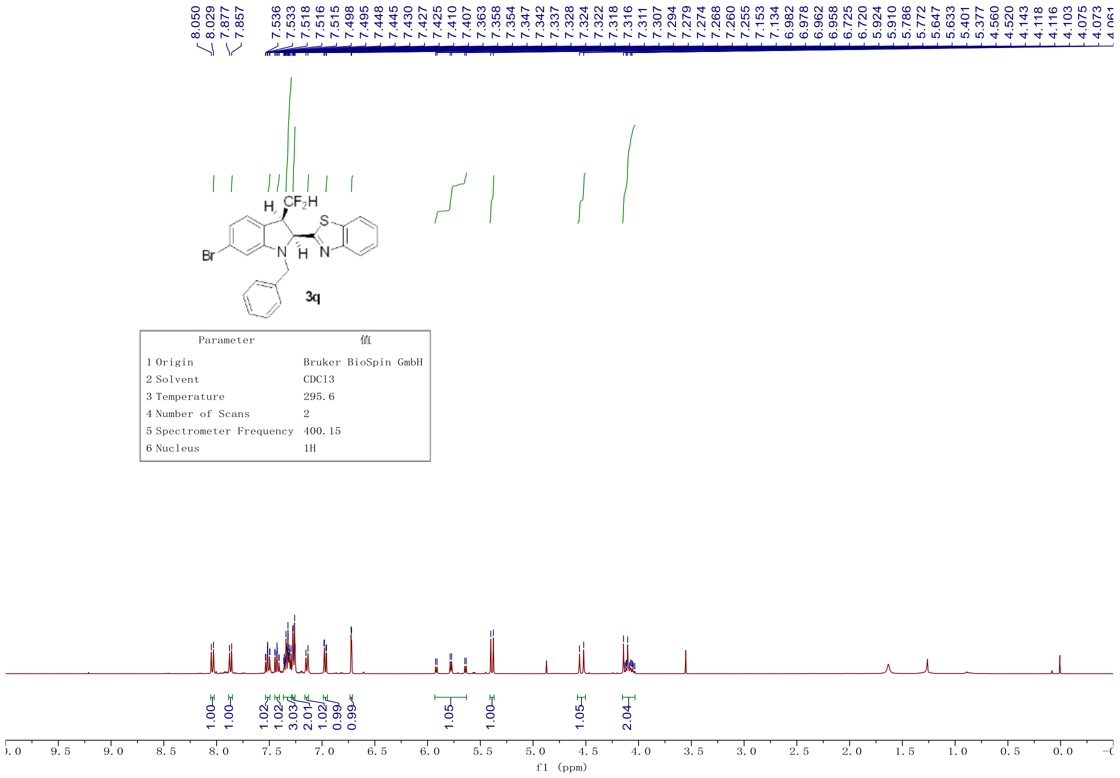

Figure S66. ^{1}H-NMR of **3q**

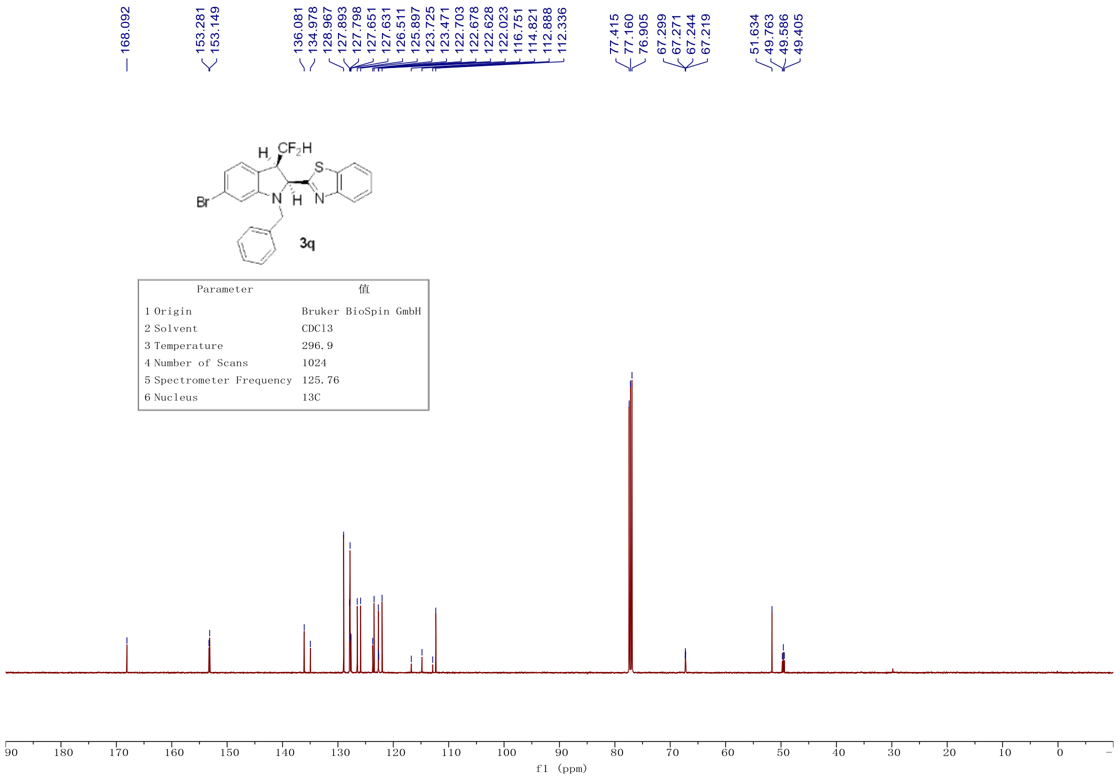

Figure S67. ^{13}C-NMR of **3q**

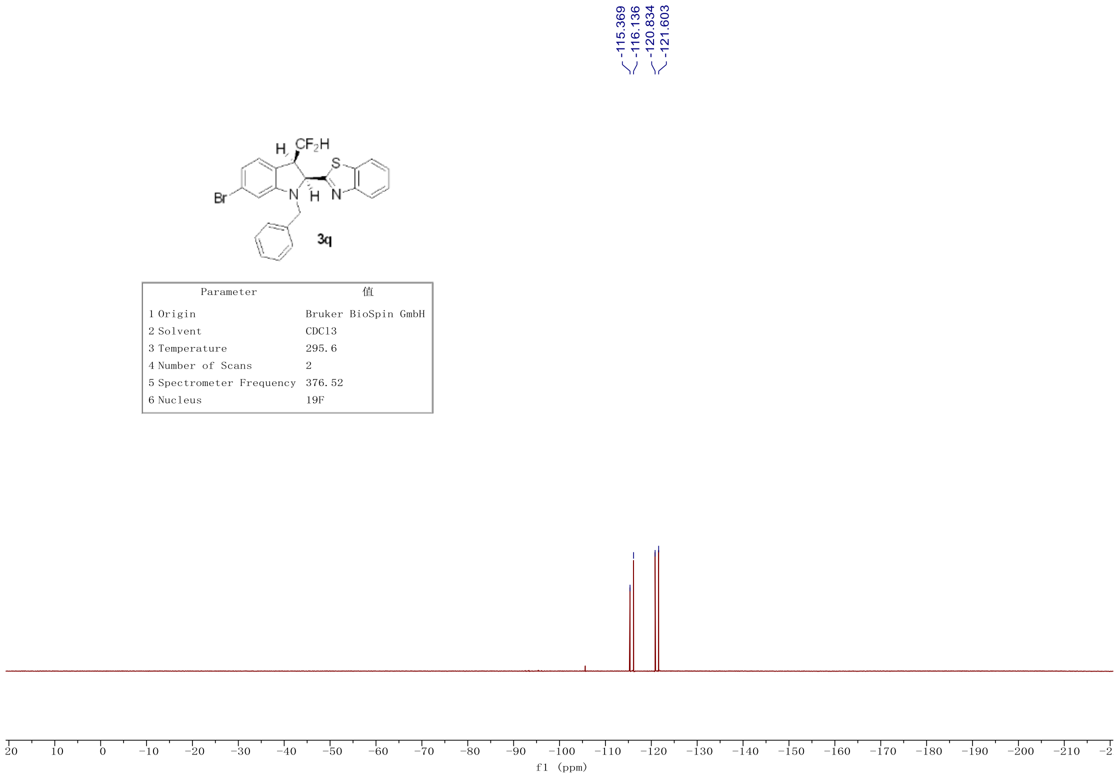

Figure S68. ^{19}F-NMR of **3q**

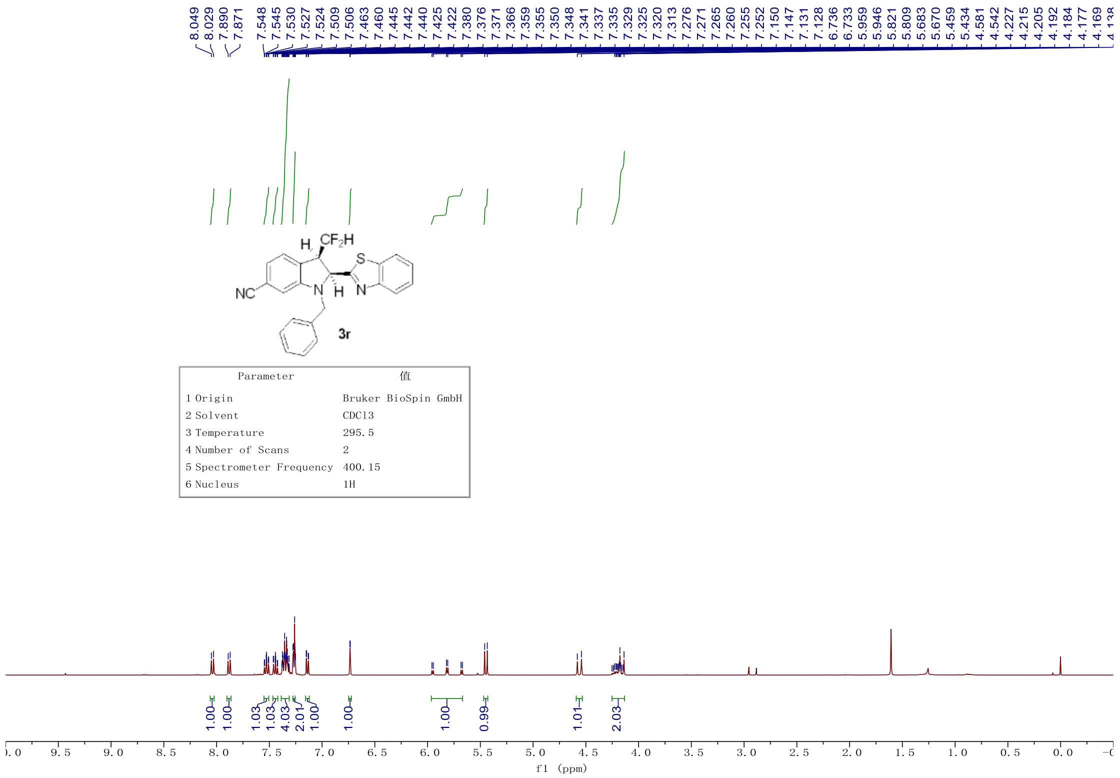

Figure S69. ^{1}H-NMR of **3r**

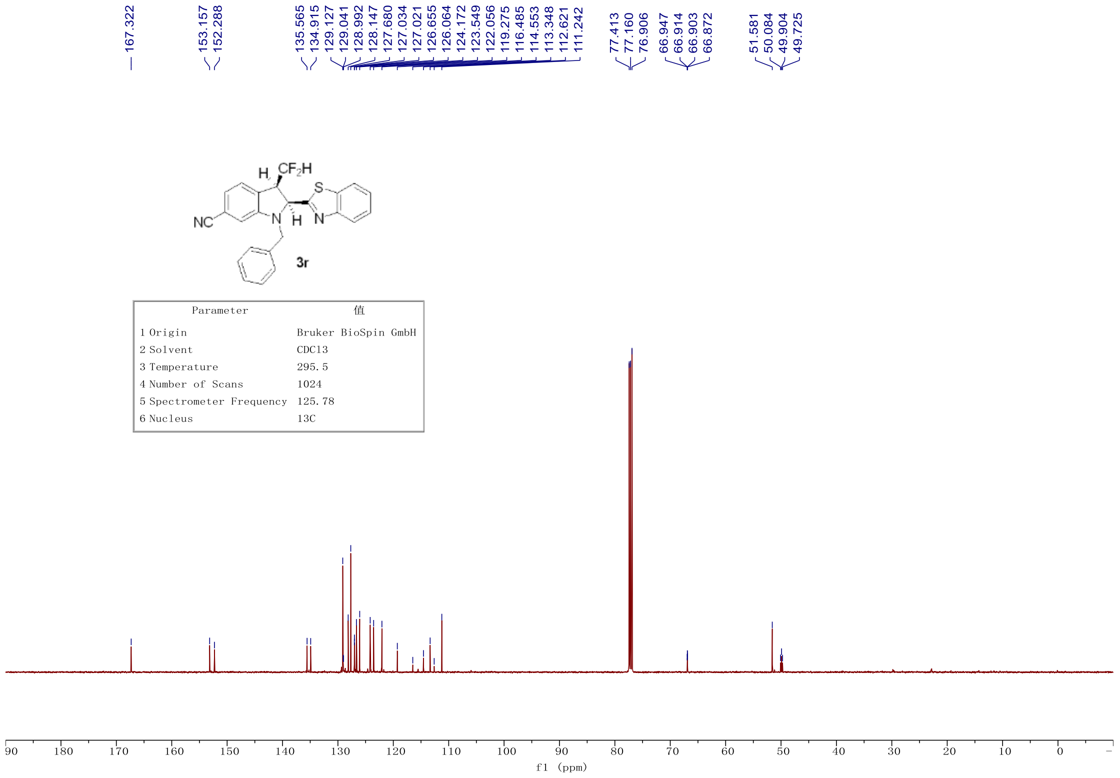

Figure S70. ^{13}C-NMR of **3r**

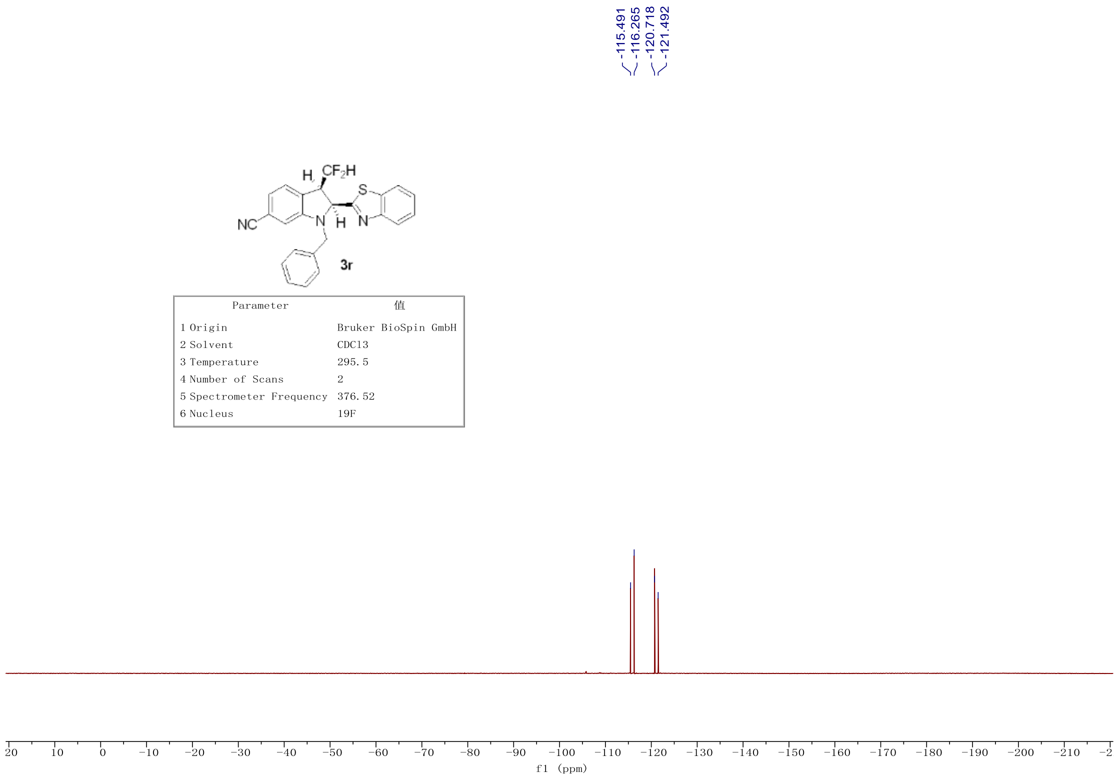

Figure S71. ^{19}F-NMR of **3r**

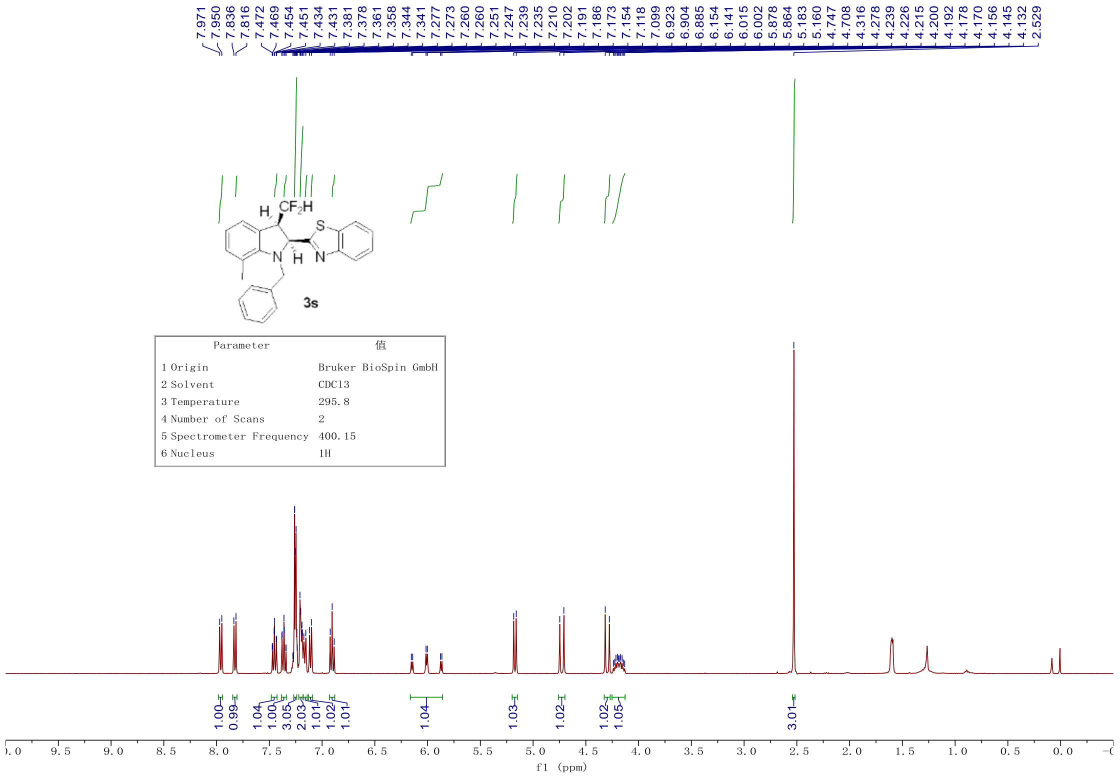

Figure S72. ^{1}H-NMR of **3s**

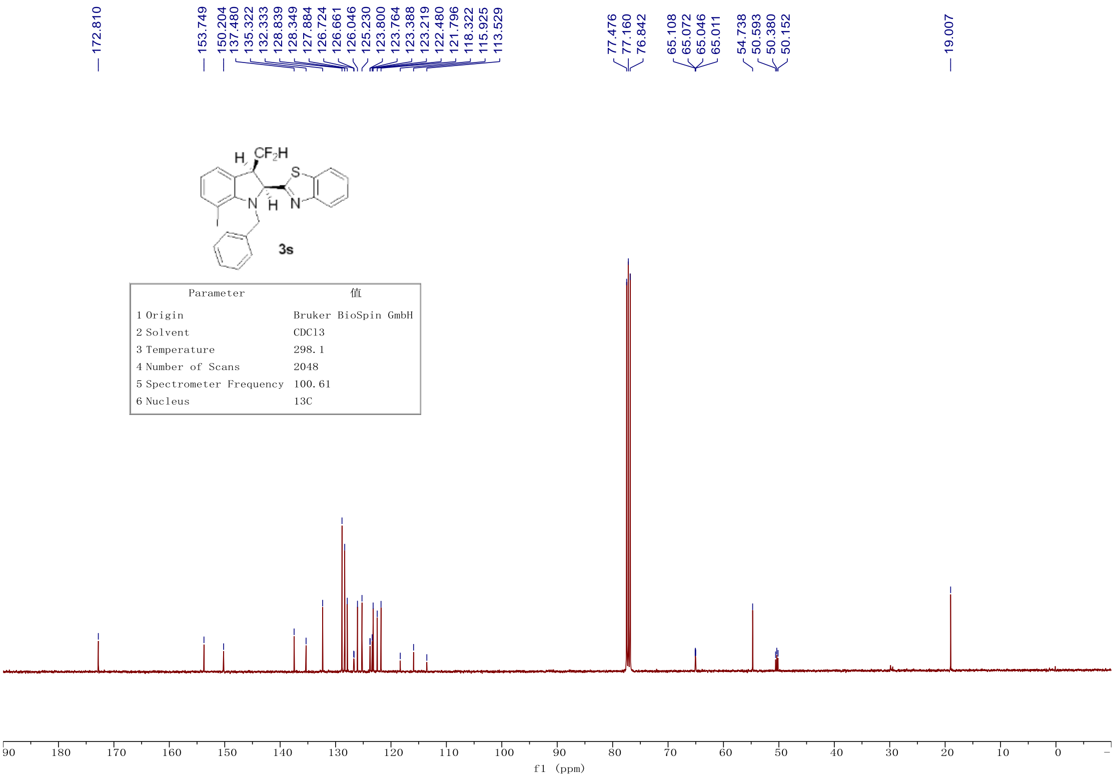

Figure S73. ^{13}C-NMR of **3s**

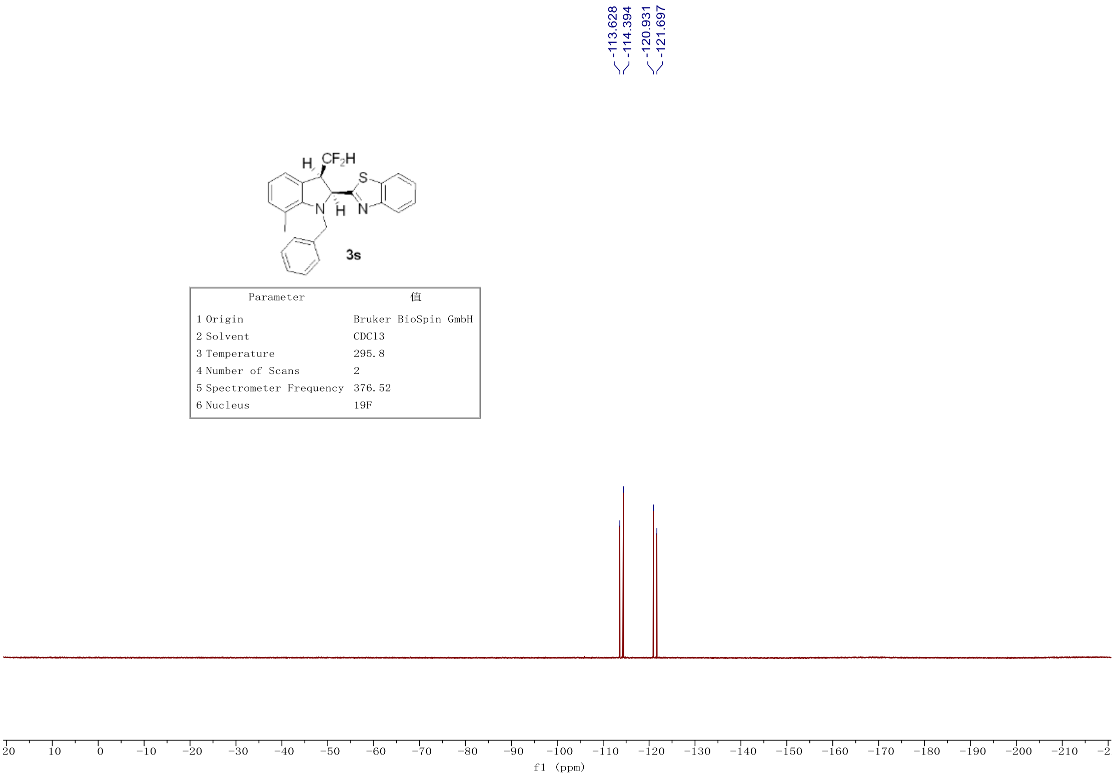

Figure S74. ^{19}F-NMR of **3s**

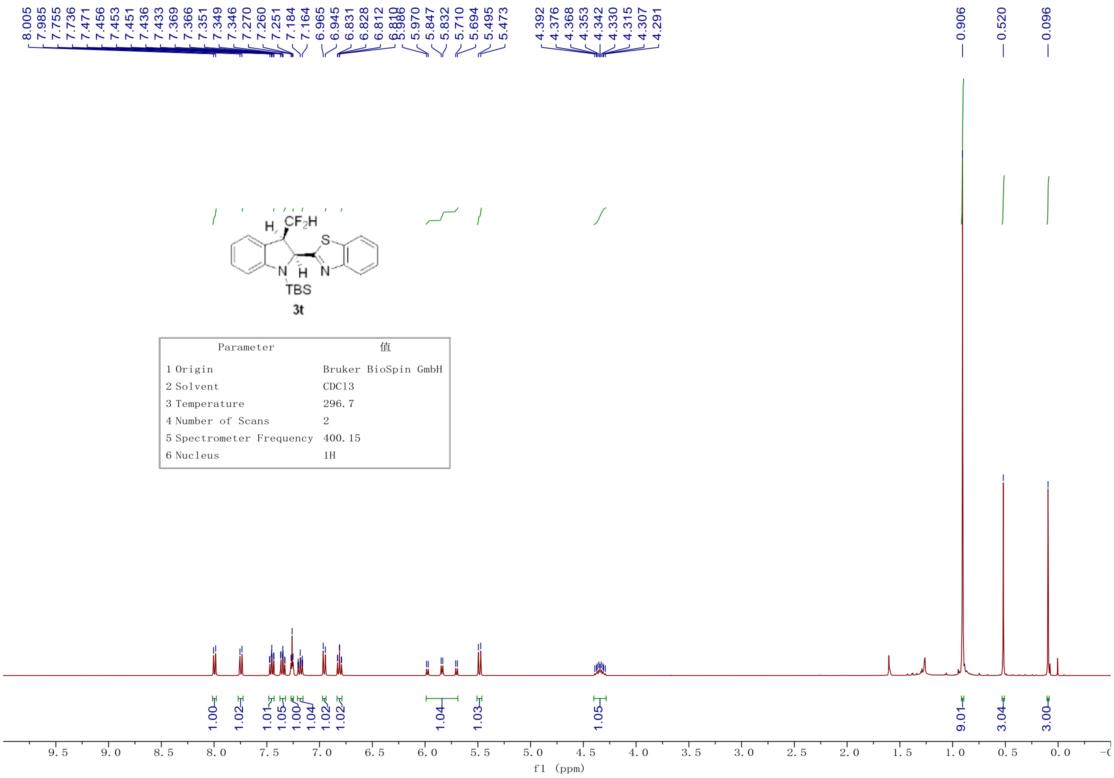

Figure S75. ^{1}H-NMR of **3t**

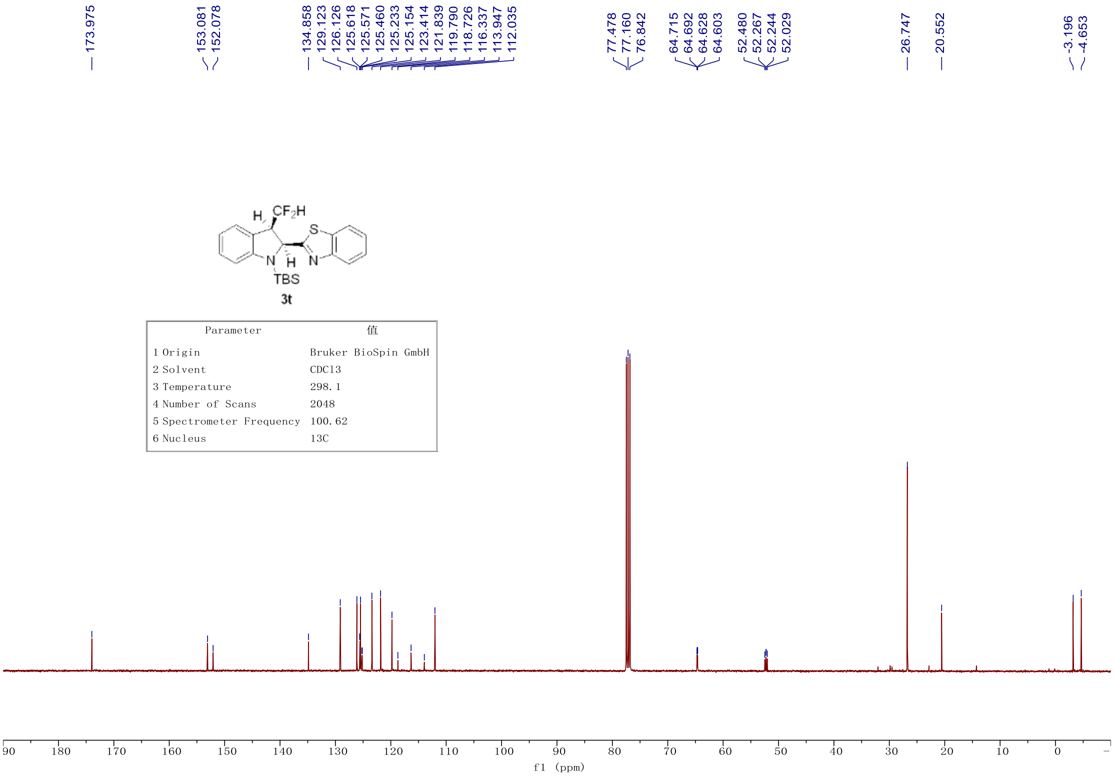

Figure S76. ^{13}C-NMR of **3t**

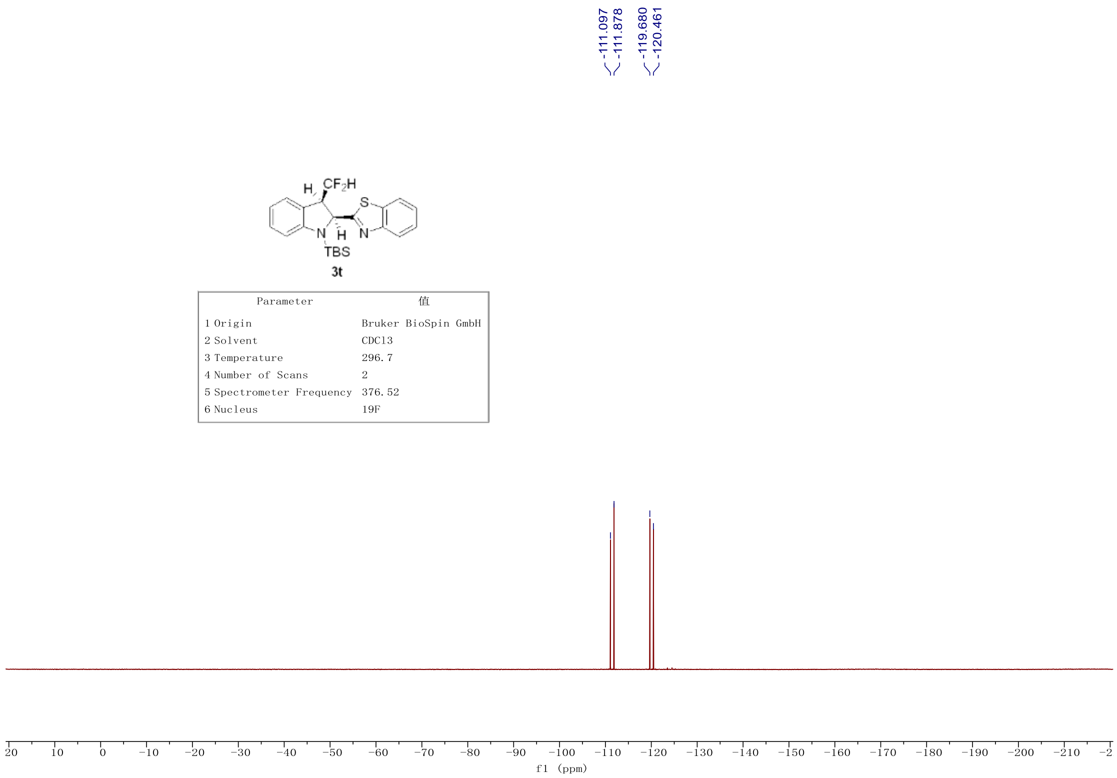

Figure S77. ^{19}F-NMR of **3t**

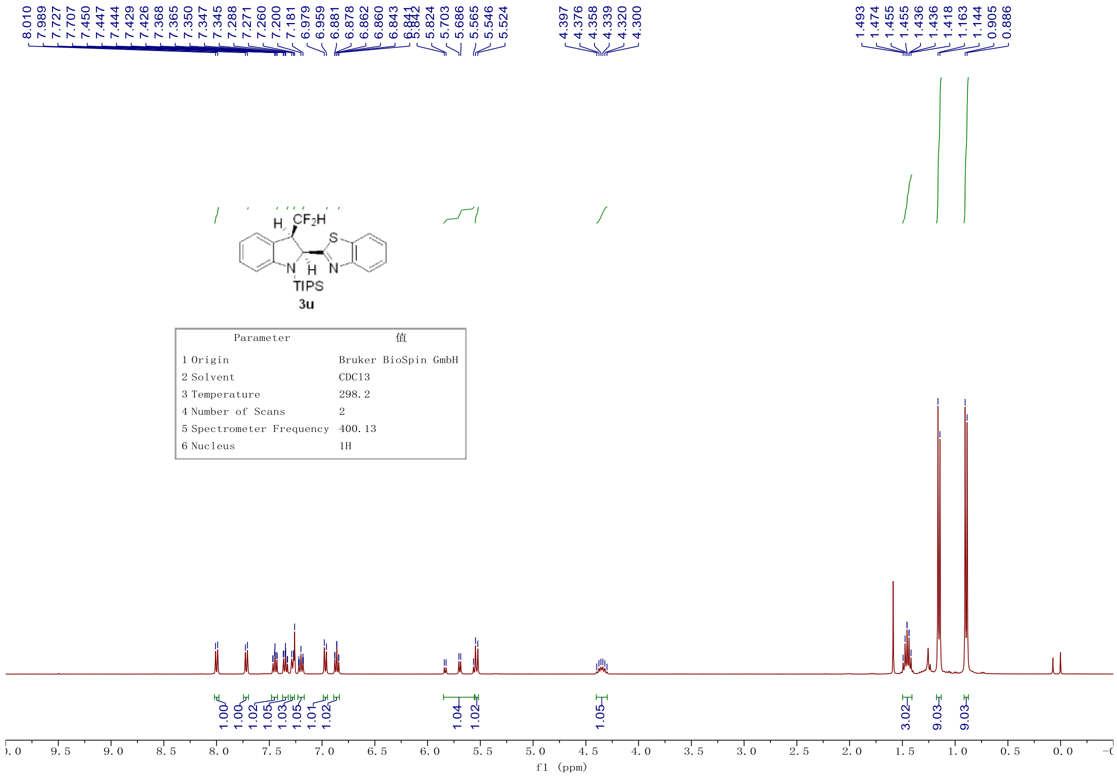

Figure S78. ^{1}H-NMR of **3u**

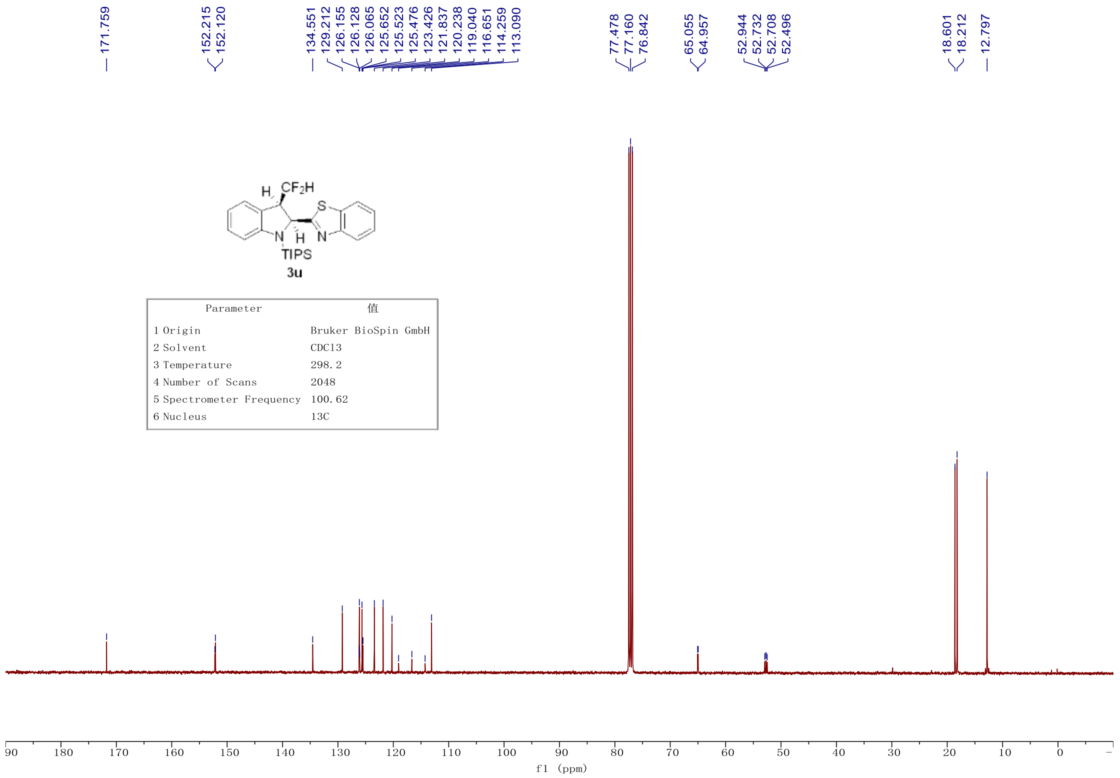

Figure S79. ^{13}C-NMR of **3u**

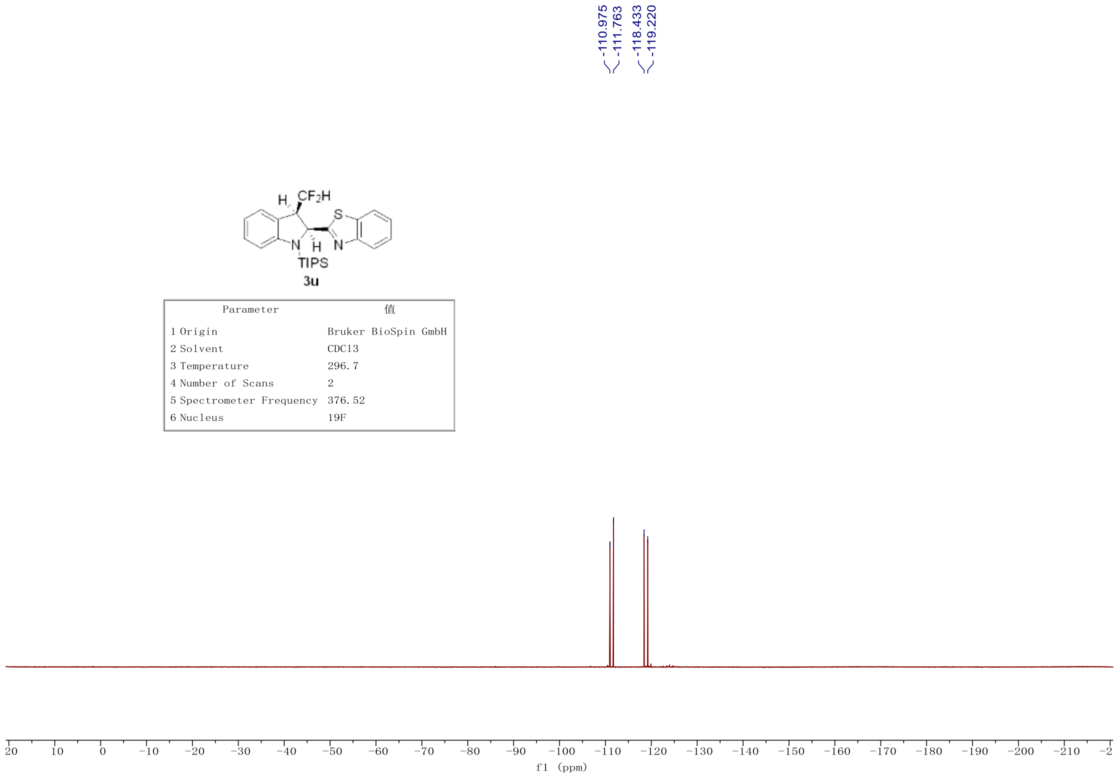

Figure S80. ^{19}F-NMR of **3u**

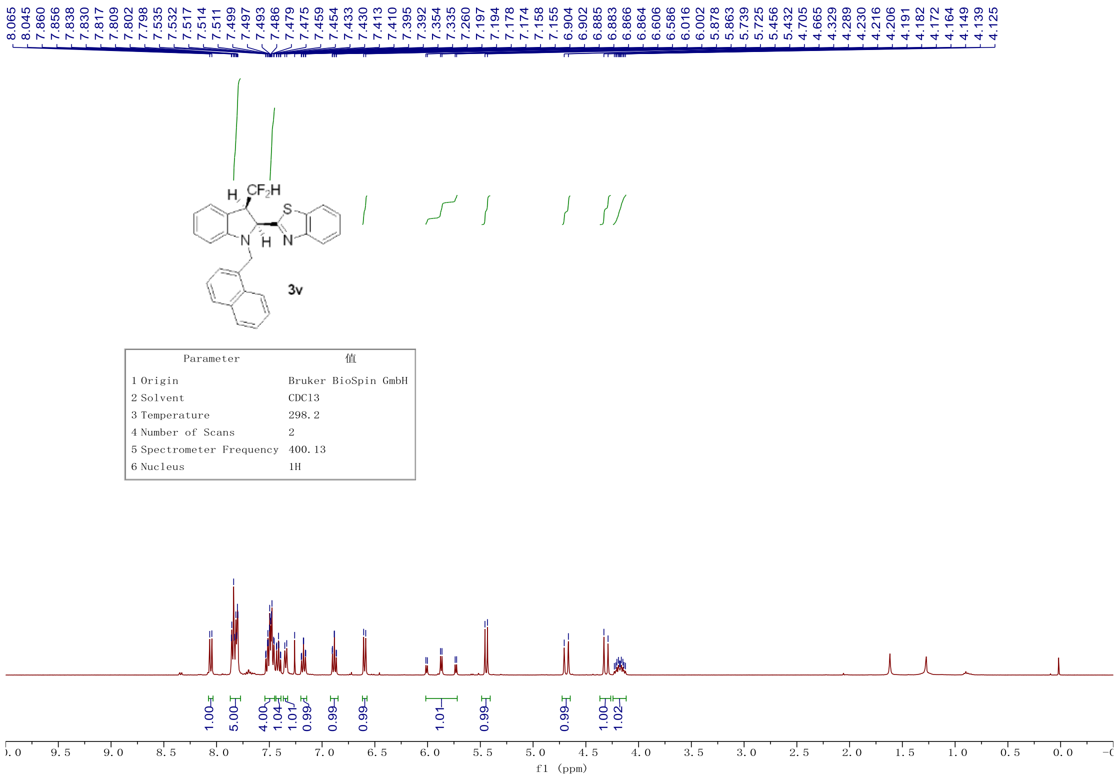

Figure S81. ^{1}H-NMR of **3v**

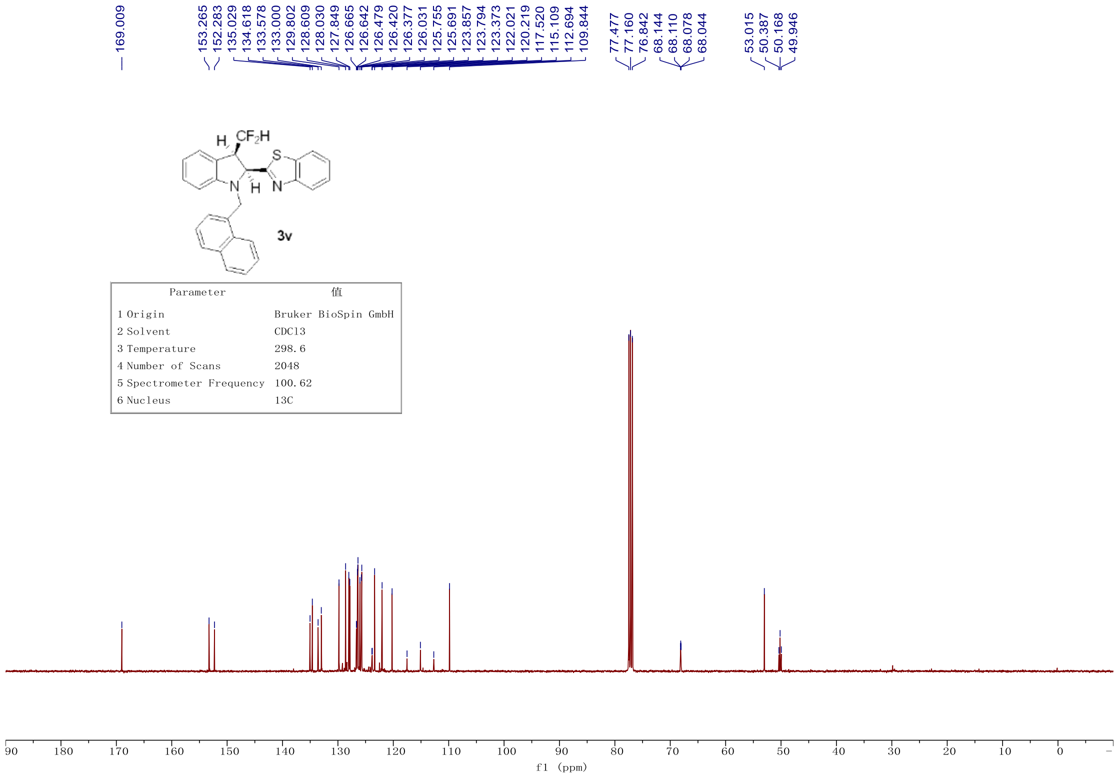

Figure S82. ^{13}C-NMR of **3v**

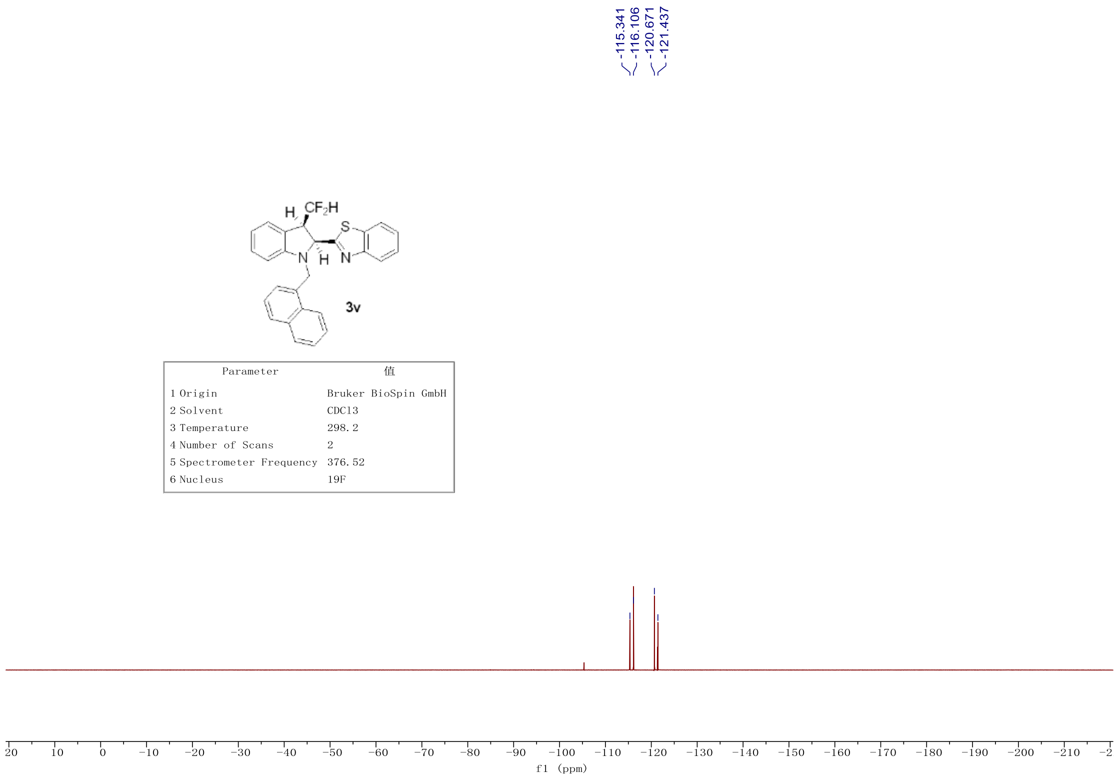

Figure S83. ^{19}F-NMR of **3v**

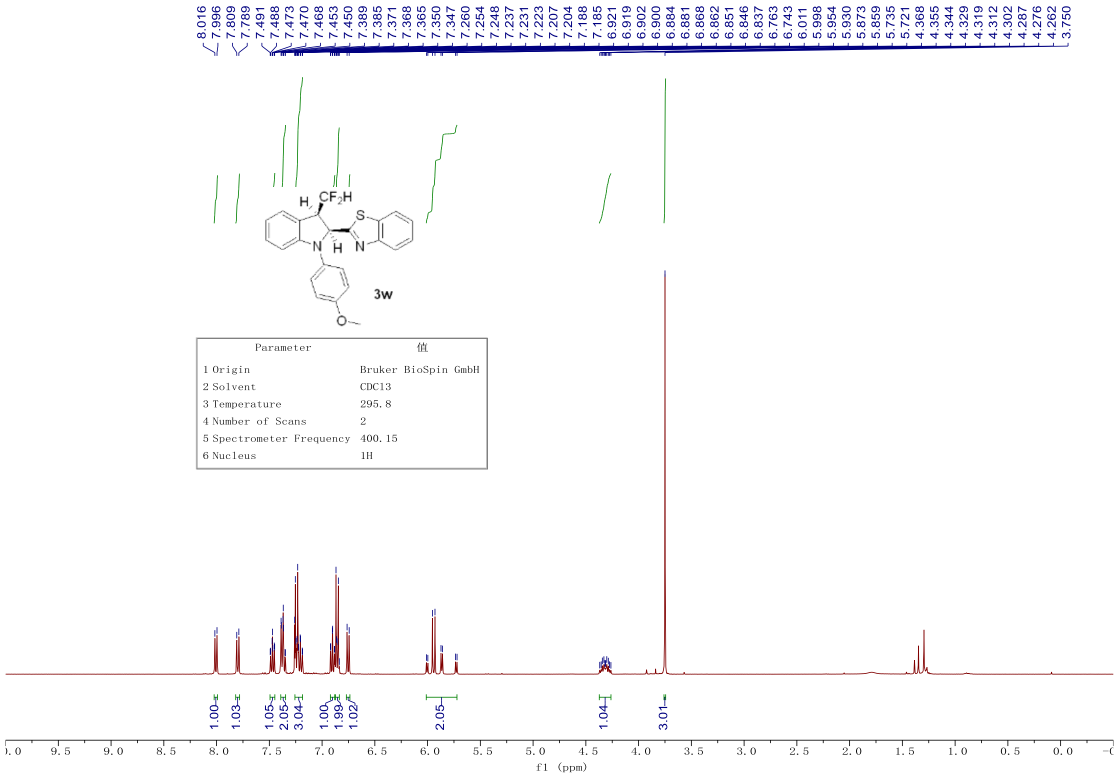

Figure S84. ^{1}H-NMR of **3w**

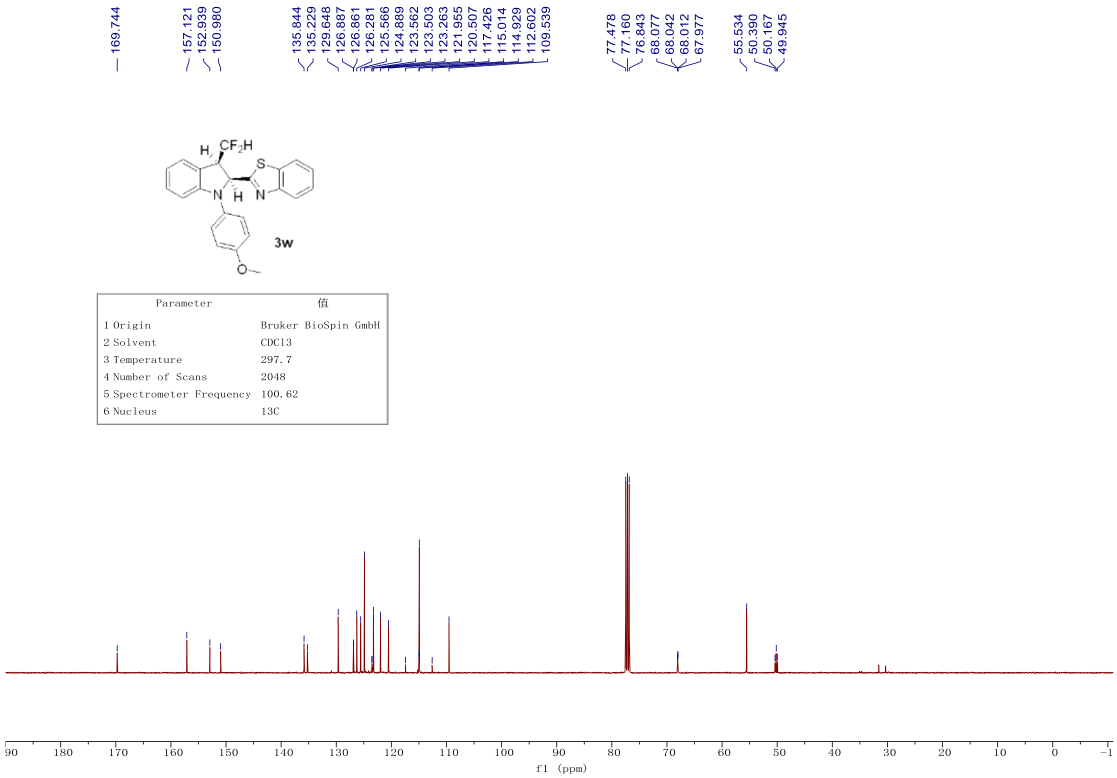

Parameter	值
1 Origin	Bruker BioSpin GmbH
2 Solvent	CDCl3
3 Temperature	297.7
4 Number of Scans	2048
5 Spectrometer Frequency	100.62
6 Nucleus	13C

Figure S85. ^{13}C-NMR of **3w**

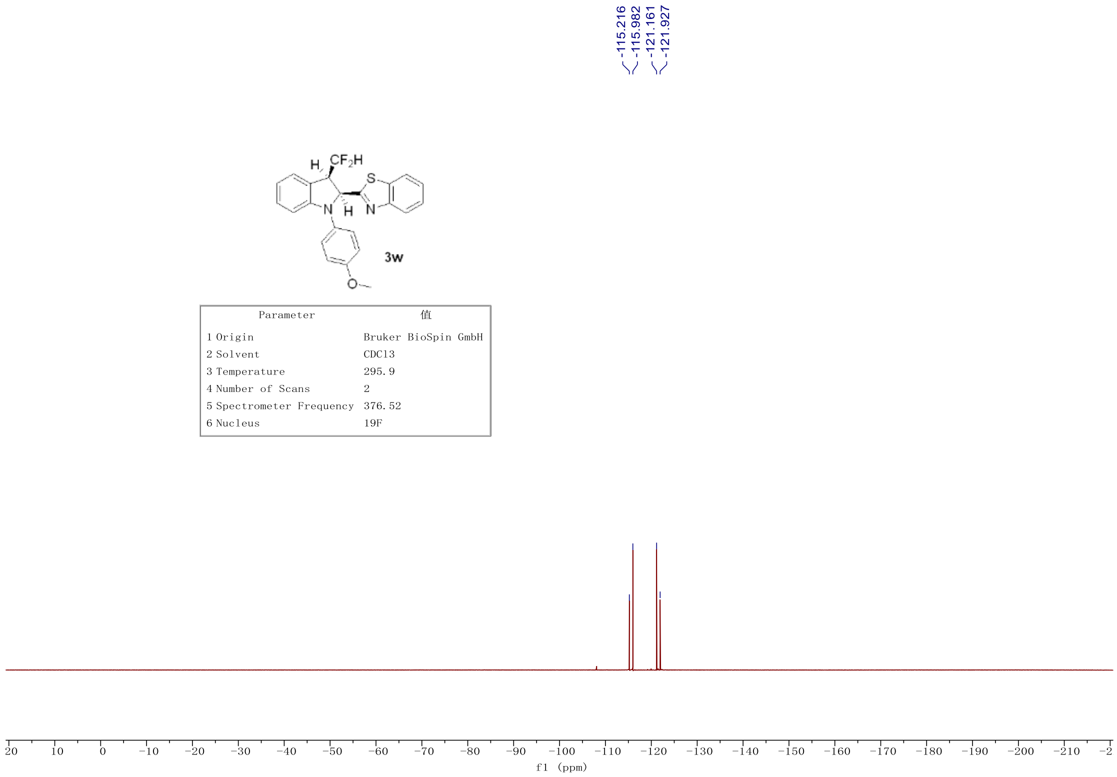

Parameter	值
1 Origin	Bruker BioSpin GmbH
2 Solvent	CDCl3
3 Temperature	295.9
4 Number of Scans	2
5 Spectrometer Frequency	376.52
6 Nucleus	19F

Figure S86. ^{19}F-NMR of **3w**

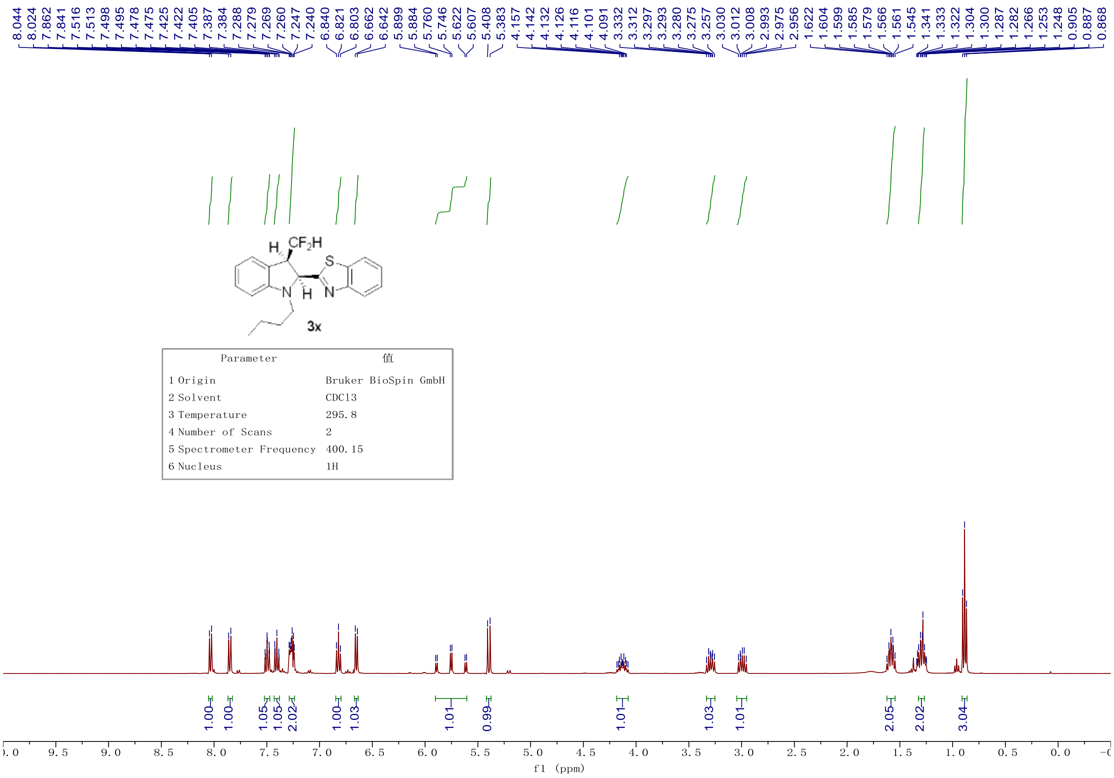

Figure S87. ^{1}H-NMR of **3x**

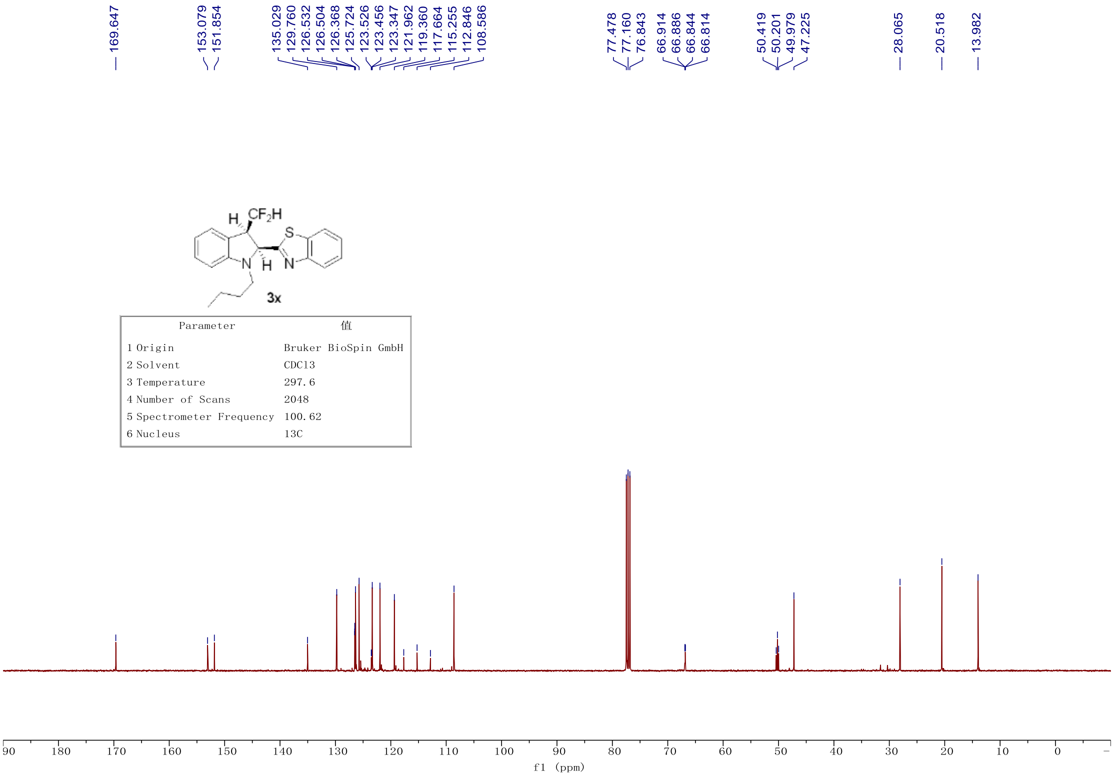

Figure S88. ^{13}C-NMR of **3x**

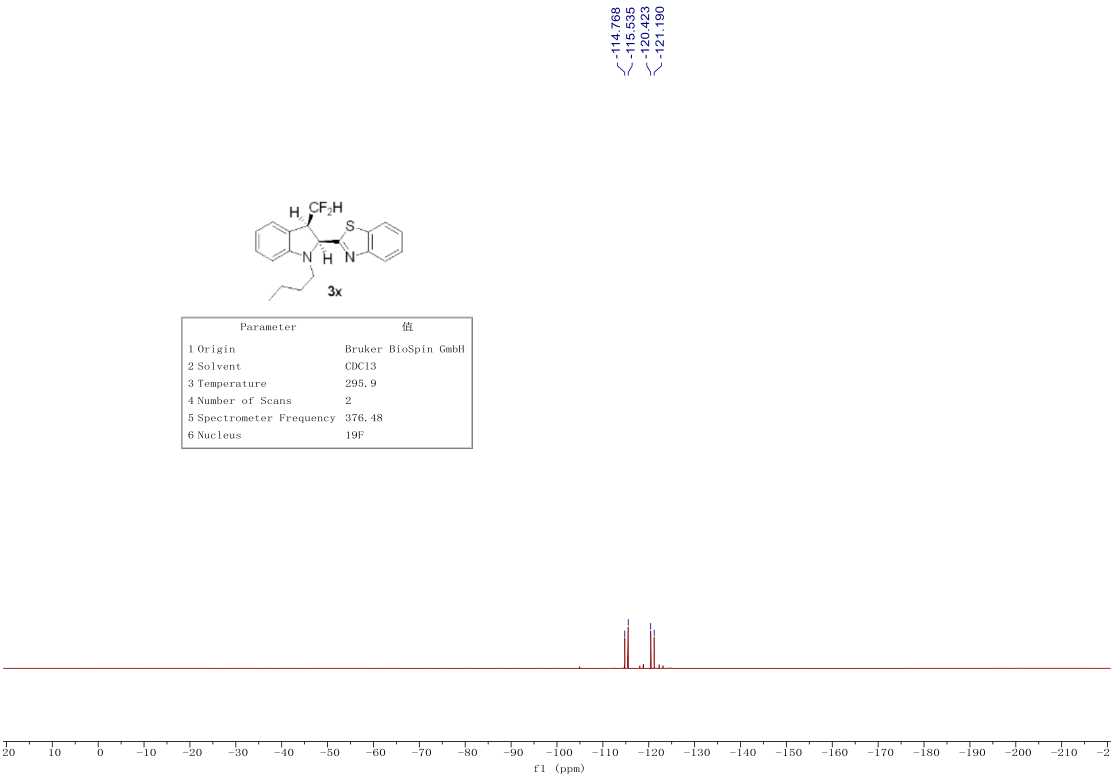

Figure S89. ^{19}F-NMR of **3x**

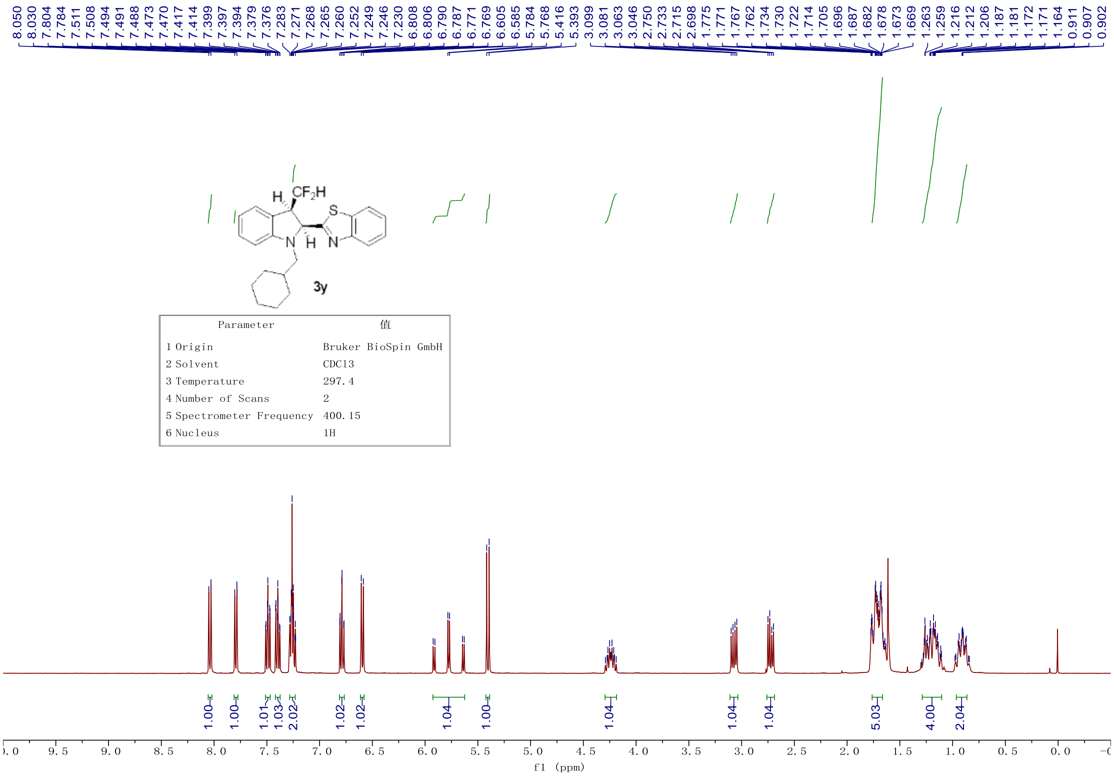

Figure S90. ^{1}H-NMR of **3y**

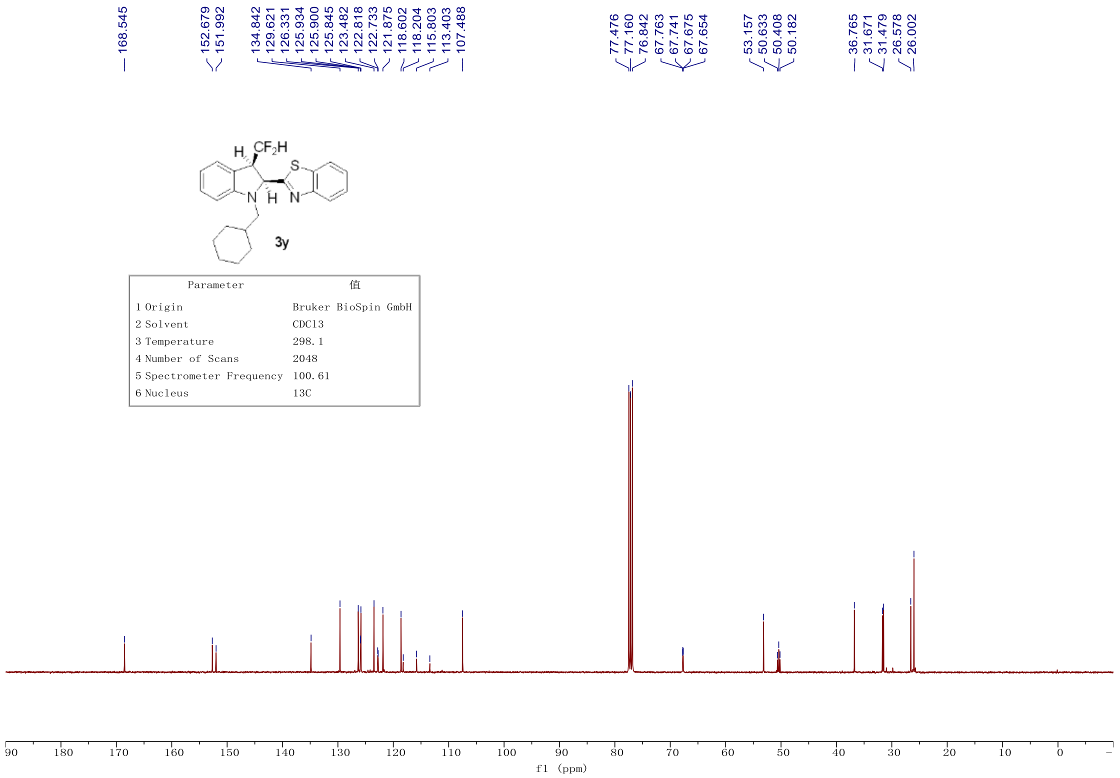

Figure S91. ^{13}C-NMR of **3y**

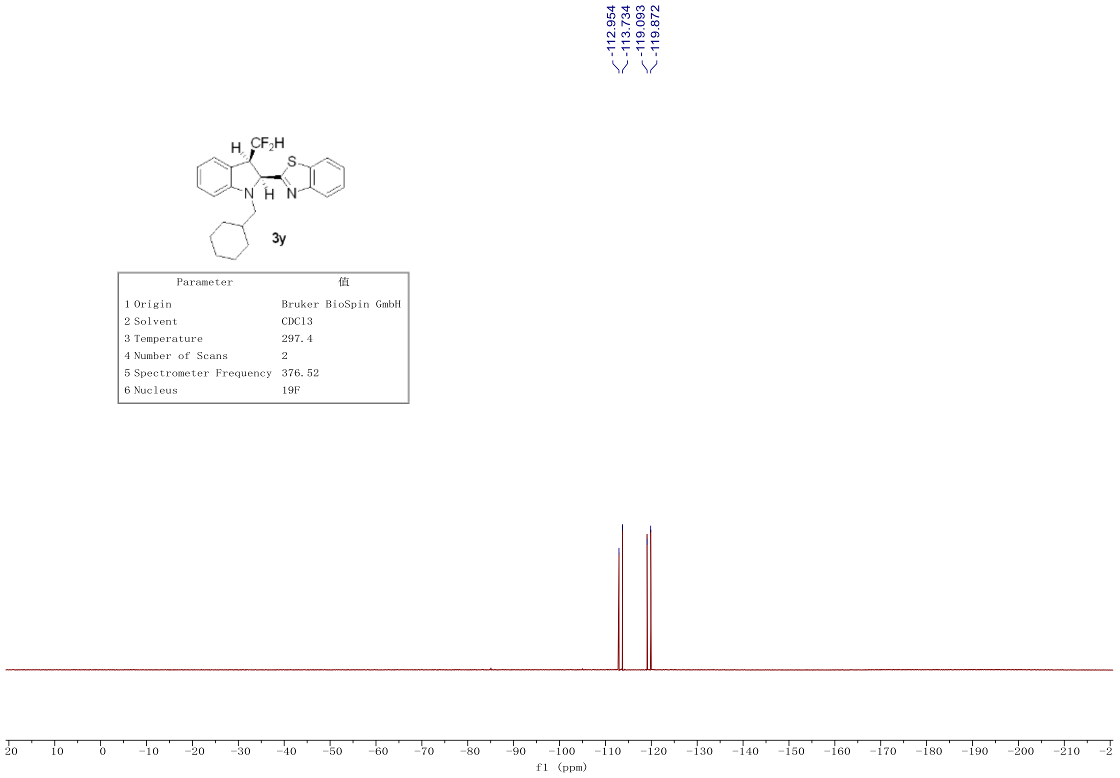

Figure S92. ^{19}F-NMR of **3y**

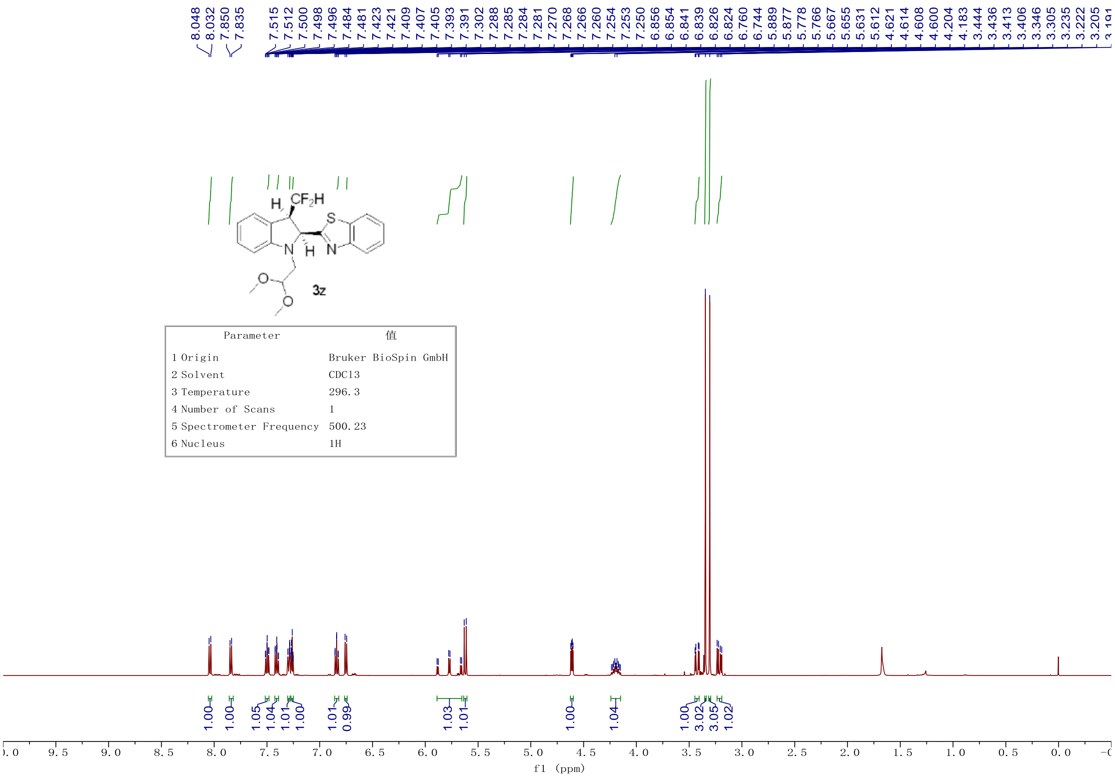

Figure S93. ^{1}H-NMR of **3z**

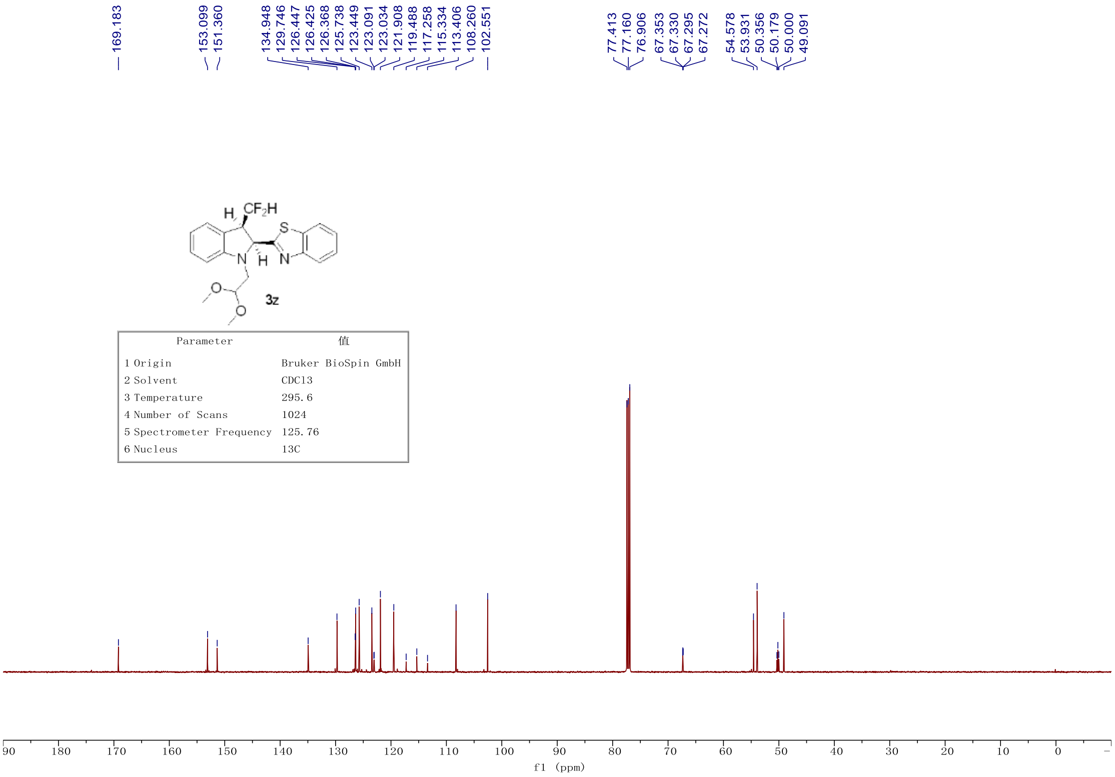

Figure S94. ^{13}C-NMR of **3z**

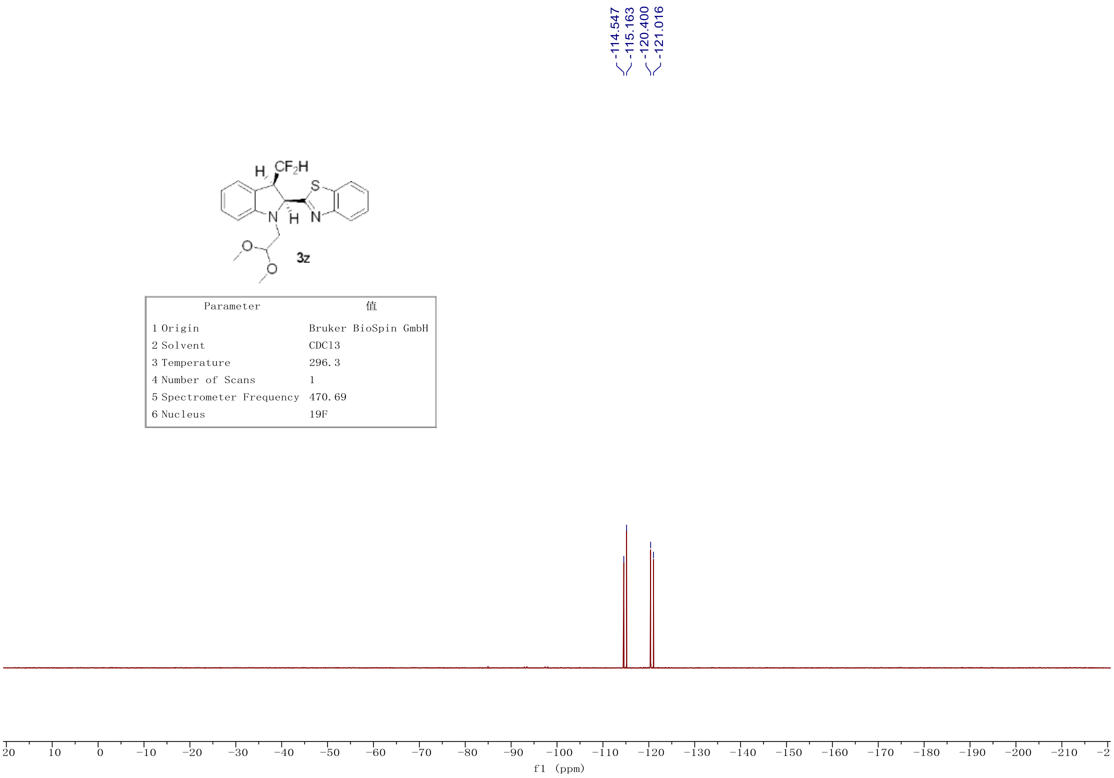

Figure S95. ^{19}F-NMR of **3z**

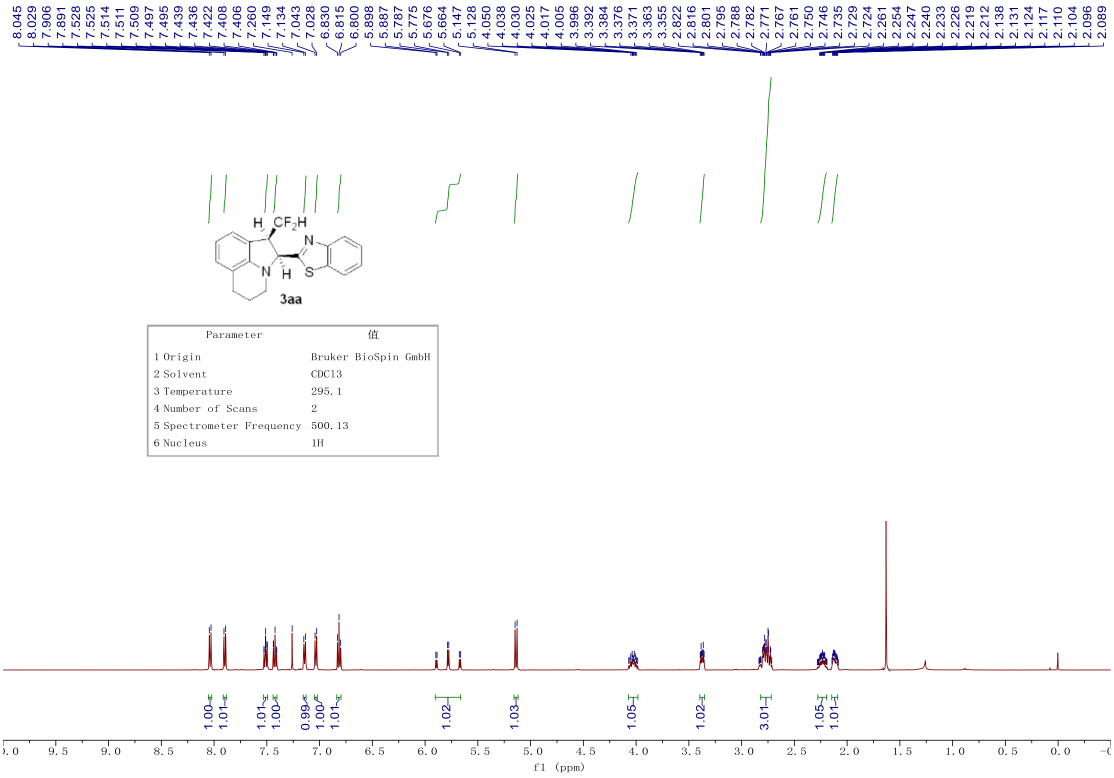

Figure S96. ^{1}H-NMR of **3aa**

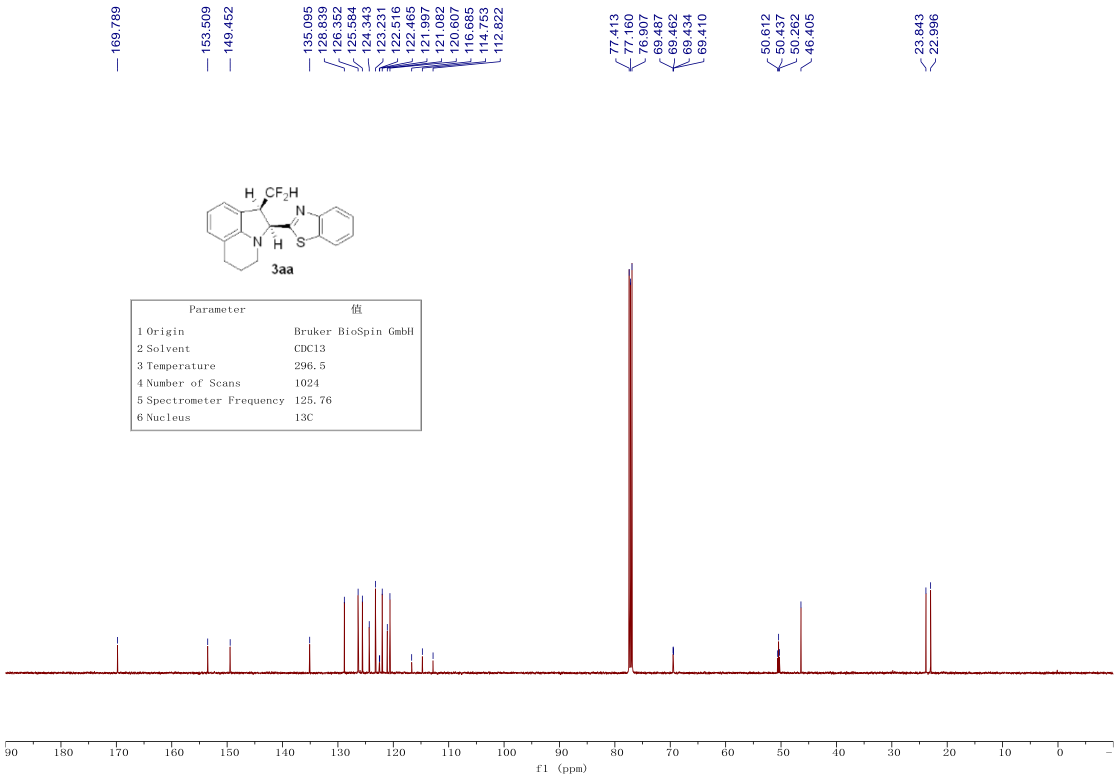

Figure S97. ^{13}C-NMR of **3aa**

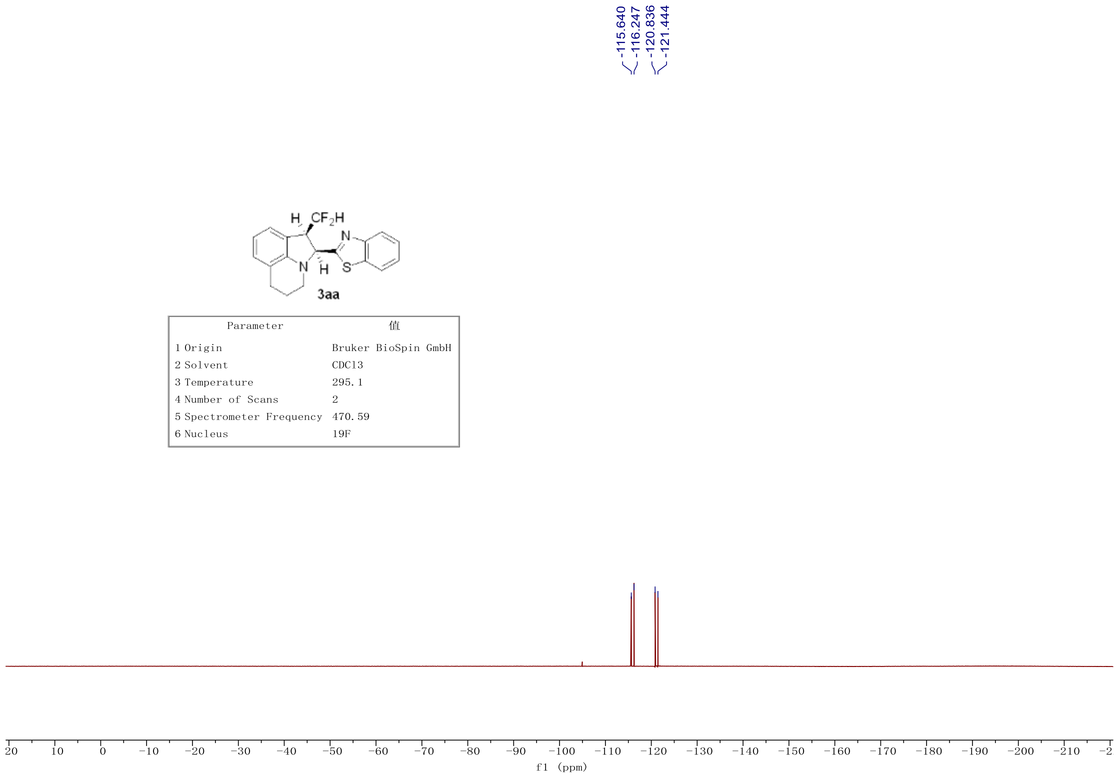

Figure S98. ^{19}F-NMR of **3aa**

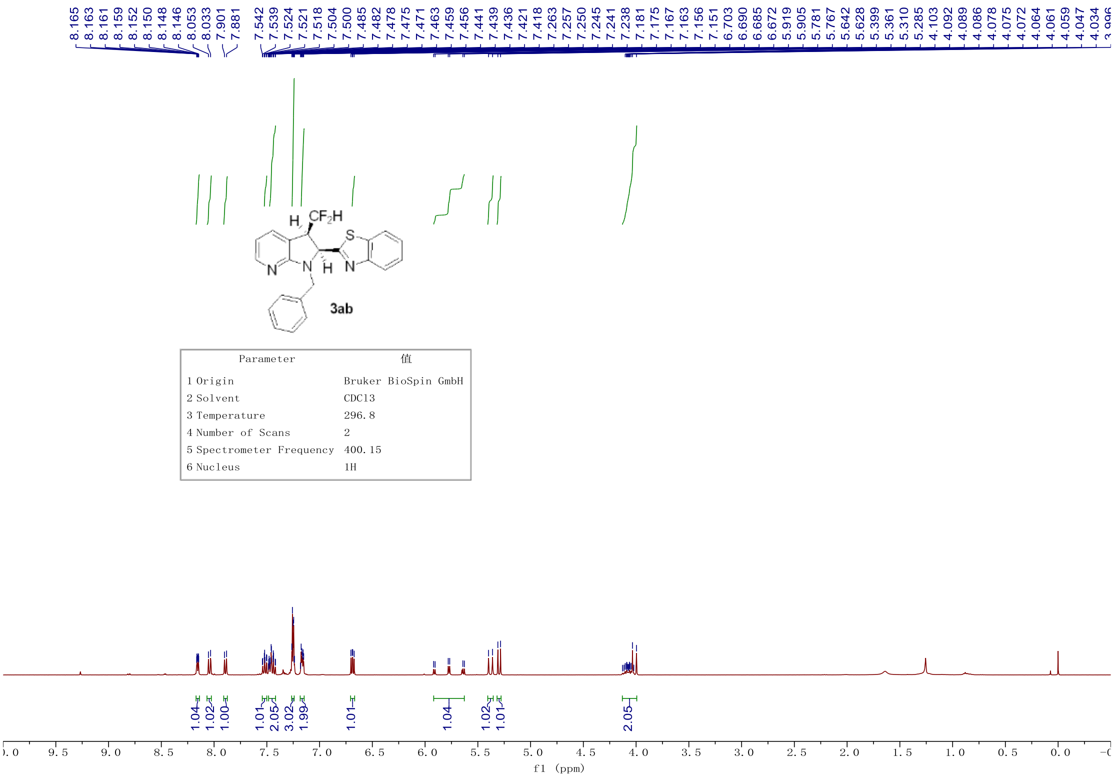

Figure S99. ^{1}H-NMR of **3ab**

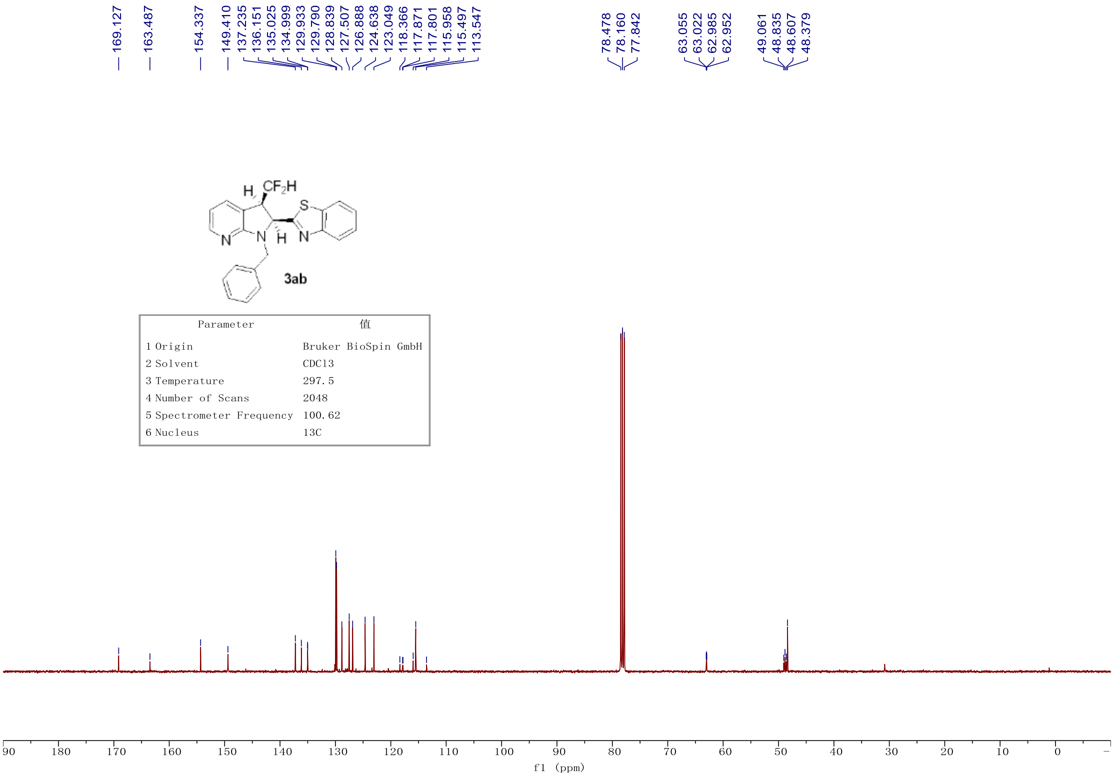

Figure S100. ^{13}C-NMR of **3ab**

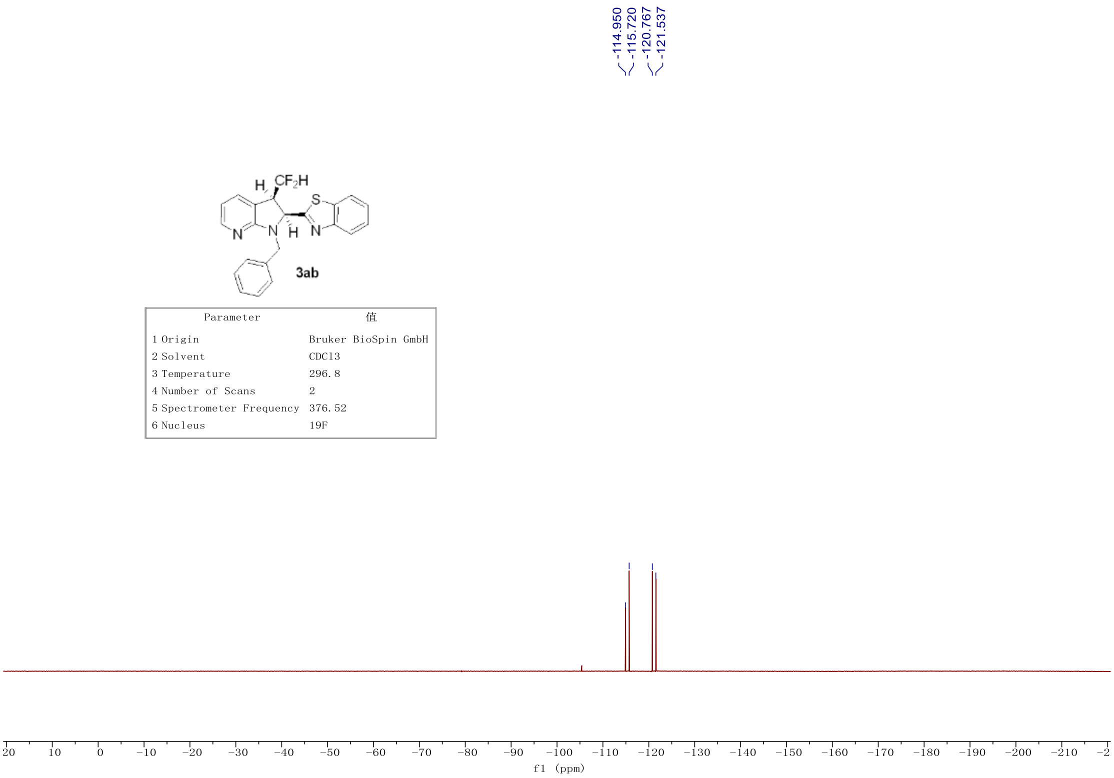

Figure S101. ^{19}F-NMR of **3ab**

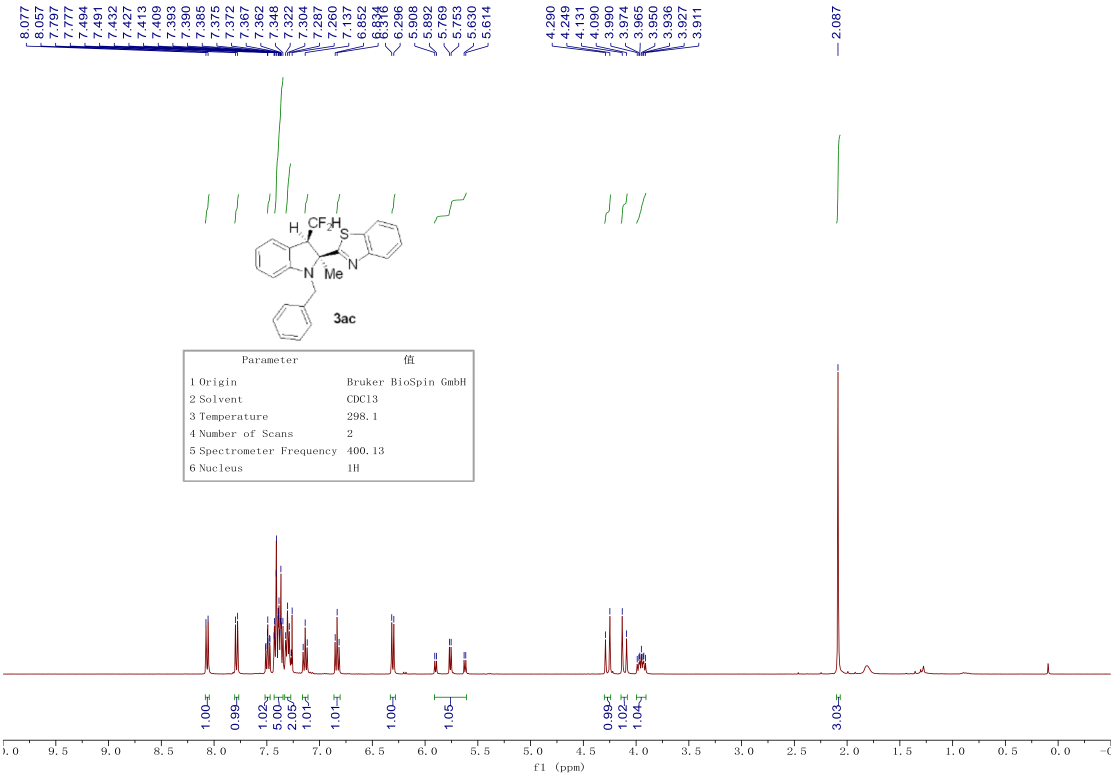

Figure S102. ^{1}H-NMR of **3ac**

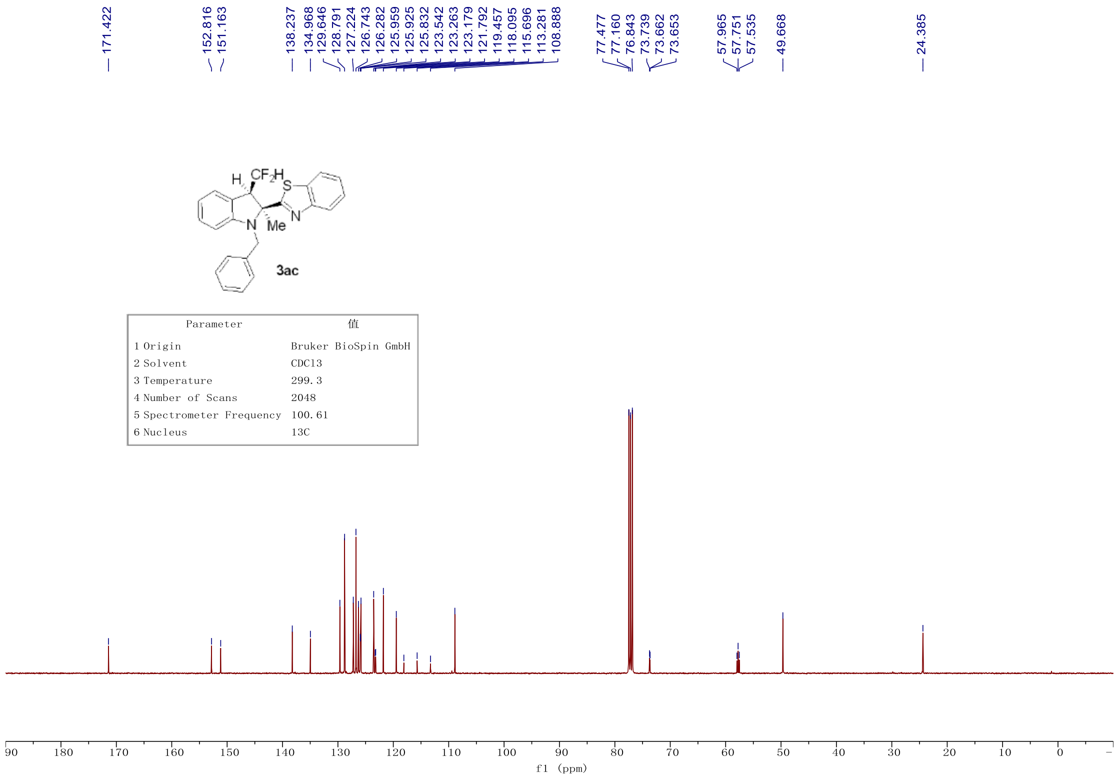

Figure S103. ^{13}C-NMR of **3ac**

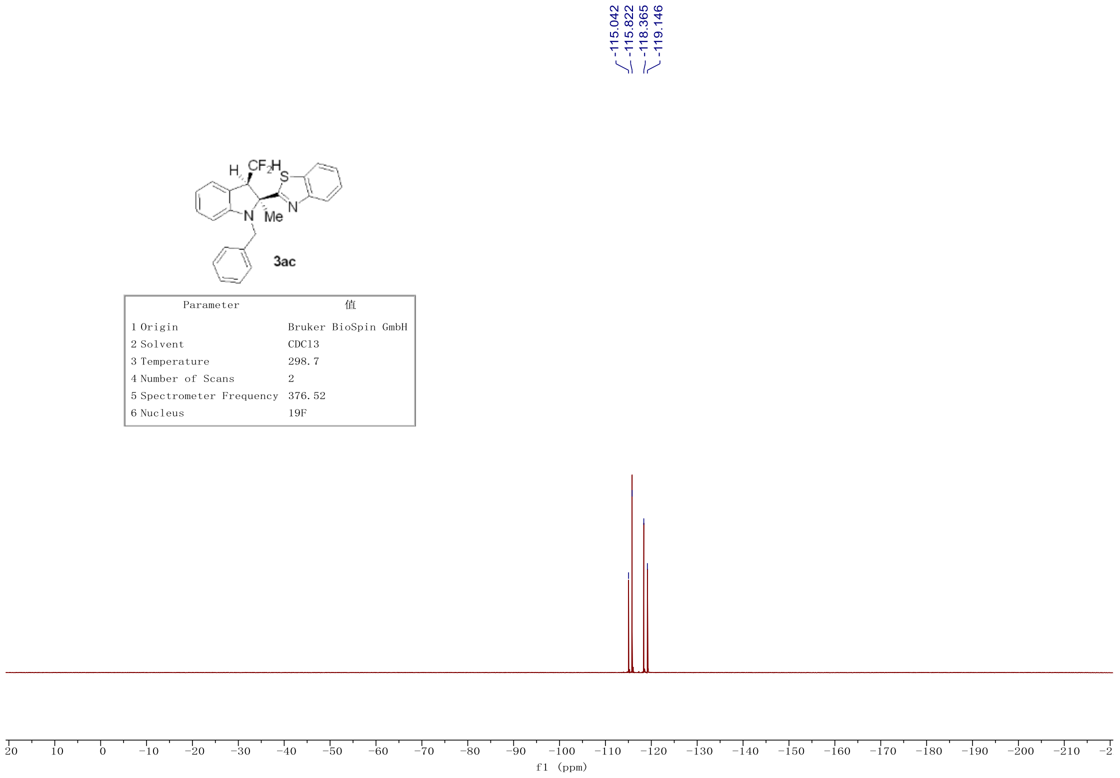

Figure S104. ^{19}F-NMR of **3ac**

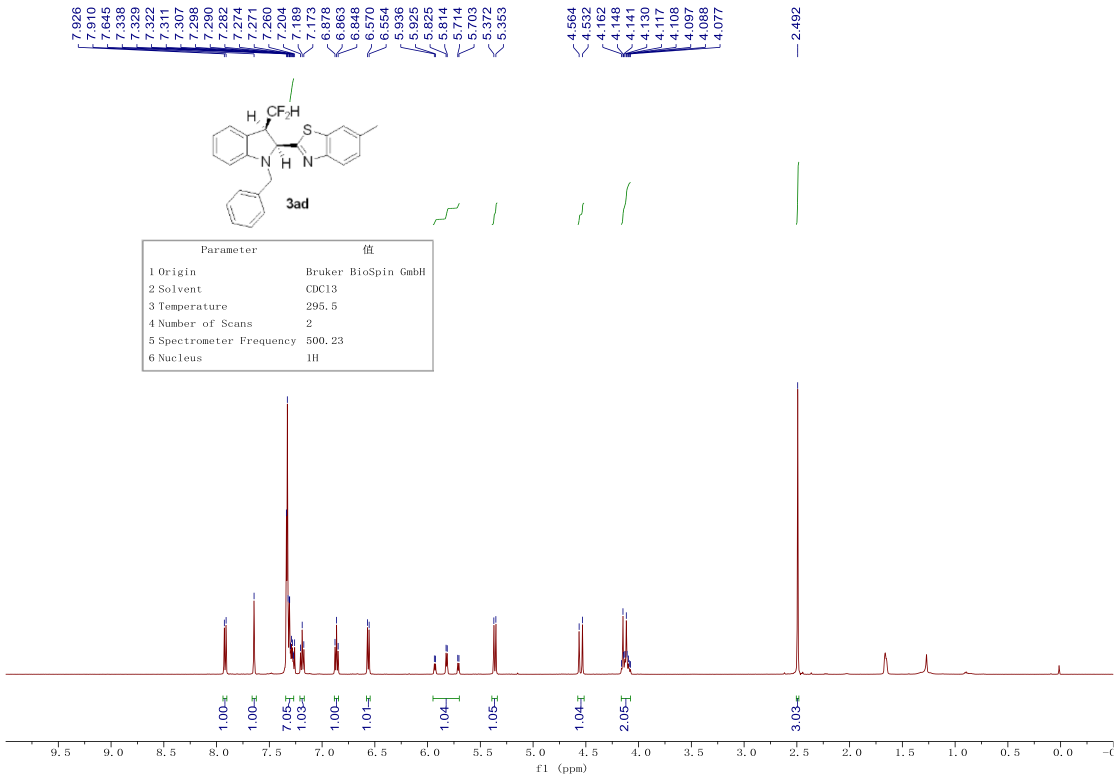

Figure S105. ^{1}H-NMR of **3ad**

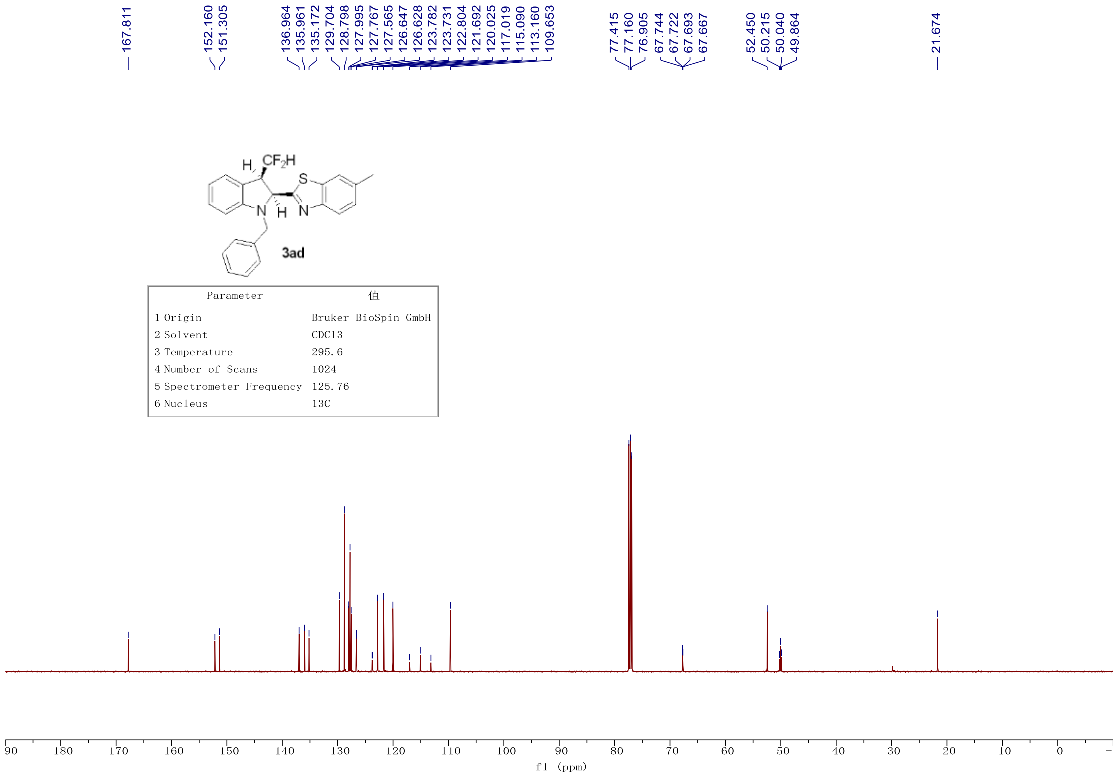

Figure S106. ^{13}C-NMR of **3ad**

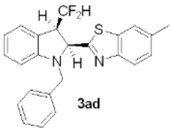

3ad

Parameter	值
1 Origin	Bruker BioSpin GmbH
2 Solvent	CDCl3
3 Temperature	295.5
4 Number of Scans	2
5 Spectrometer Frequency	470.69
6 Nucleus	19F

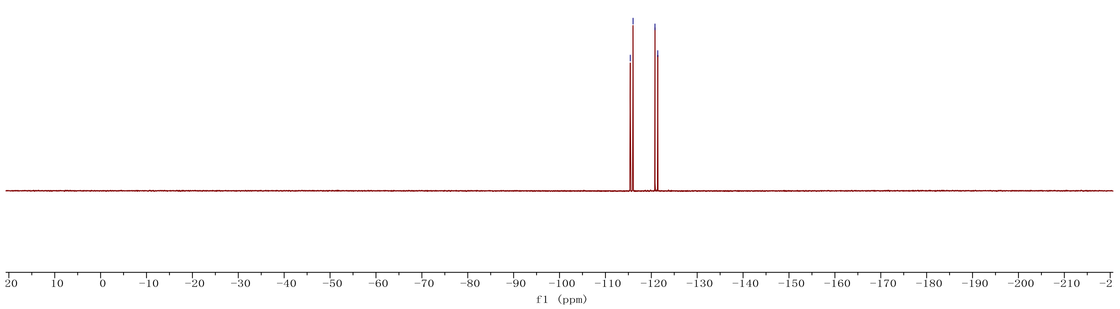

Figure S107. ^{19}F-NMR of **3ad**

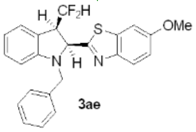

3ae

Parameter	值
1 Origin	Bruker BioSpin GmbH
2 Solvent	CDCl3
3 Temperature	297.5
4 Number of Scans	2
5 Spectrometer Frequency	400.15
6 Nucleus	1H

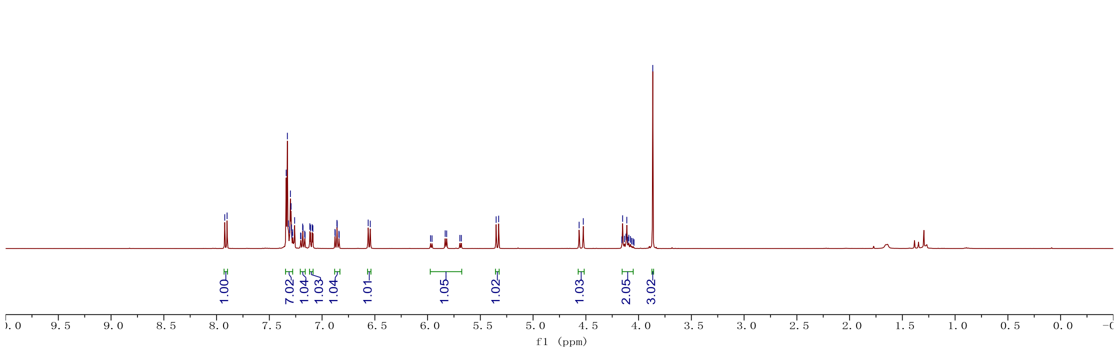

Figure S108. ^{1}H-NMR of **3ae**

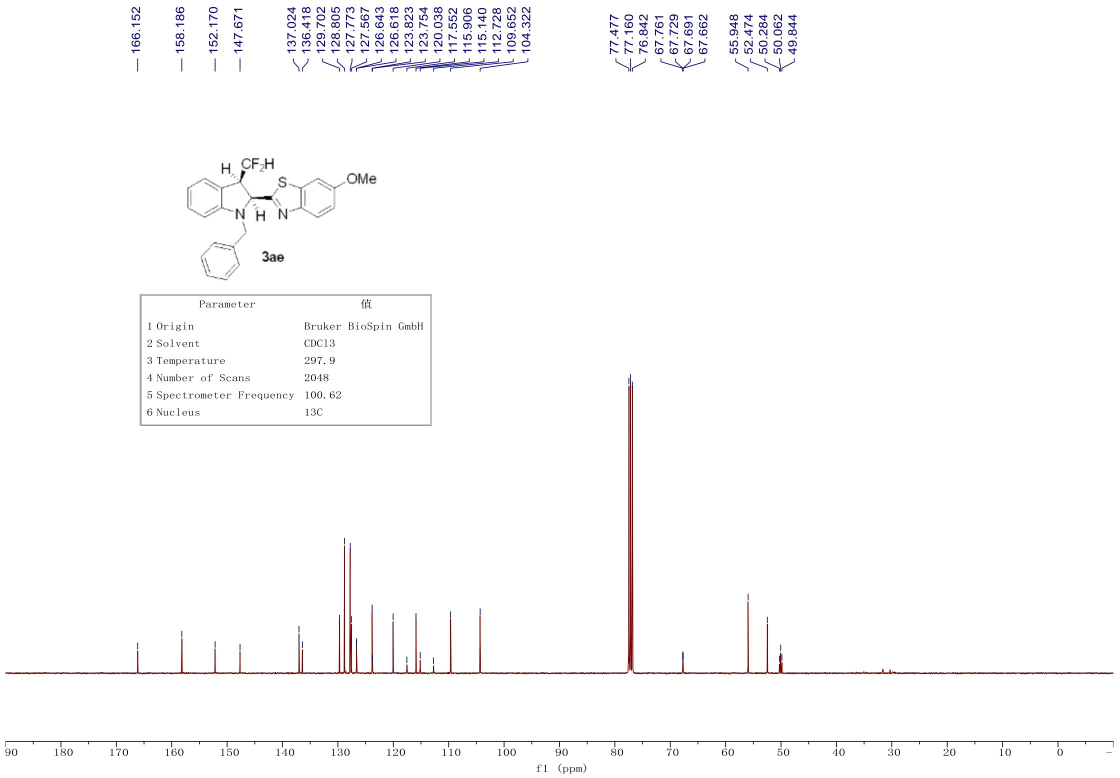

Figure S109. ^{13}C-NMR of **3ae**

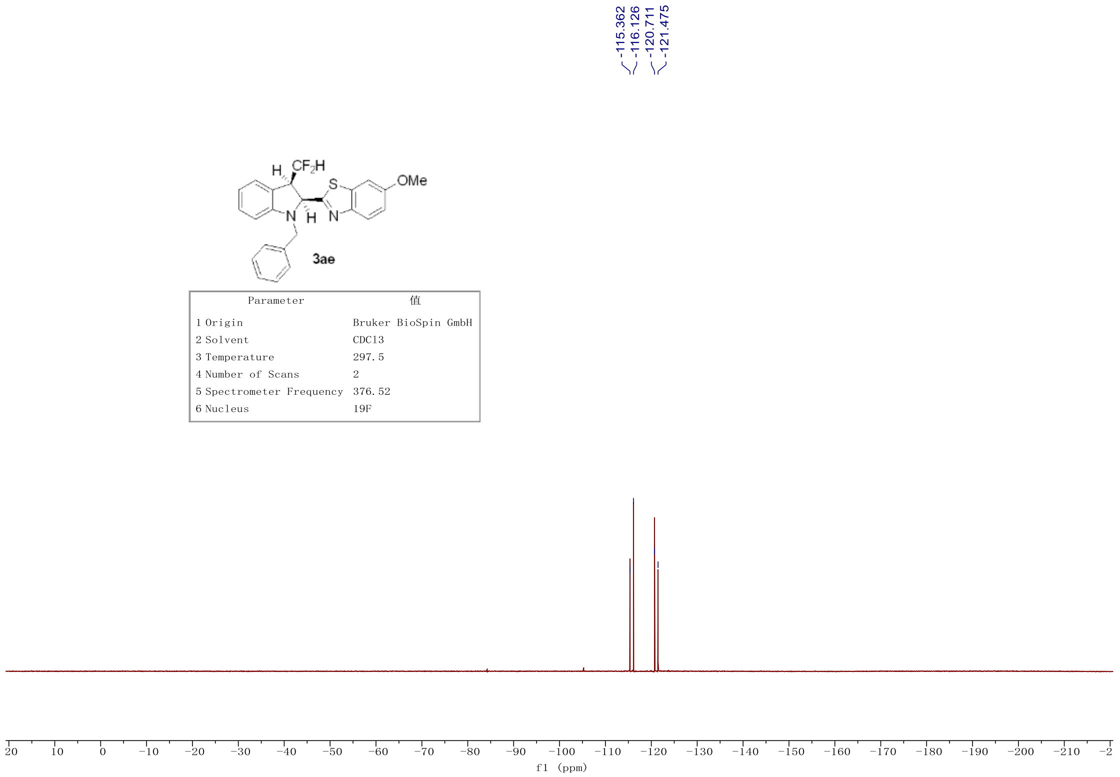

Figure S110. ^{19}F-NMR of **3ae**

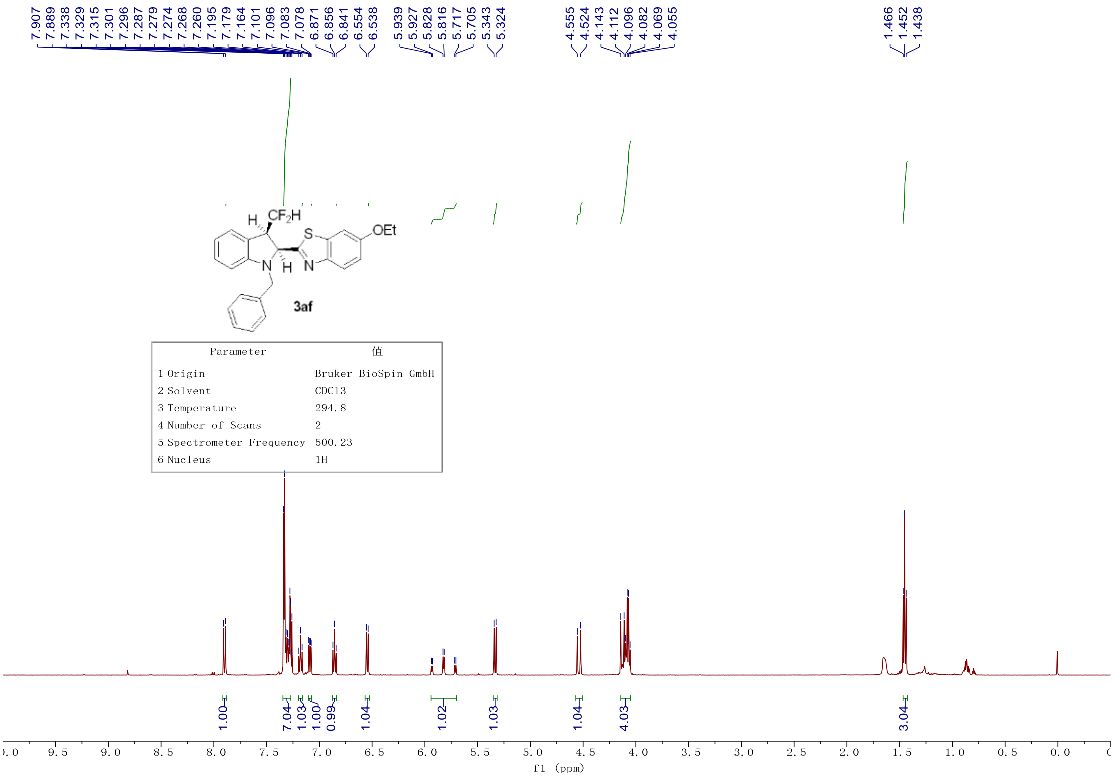

Figure S111. ^{1}H-NMR of **3af**

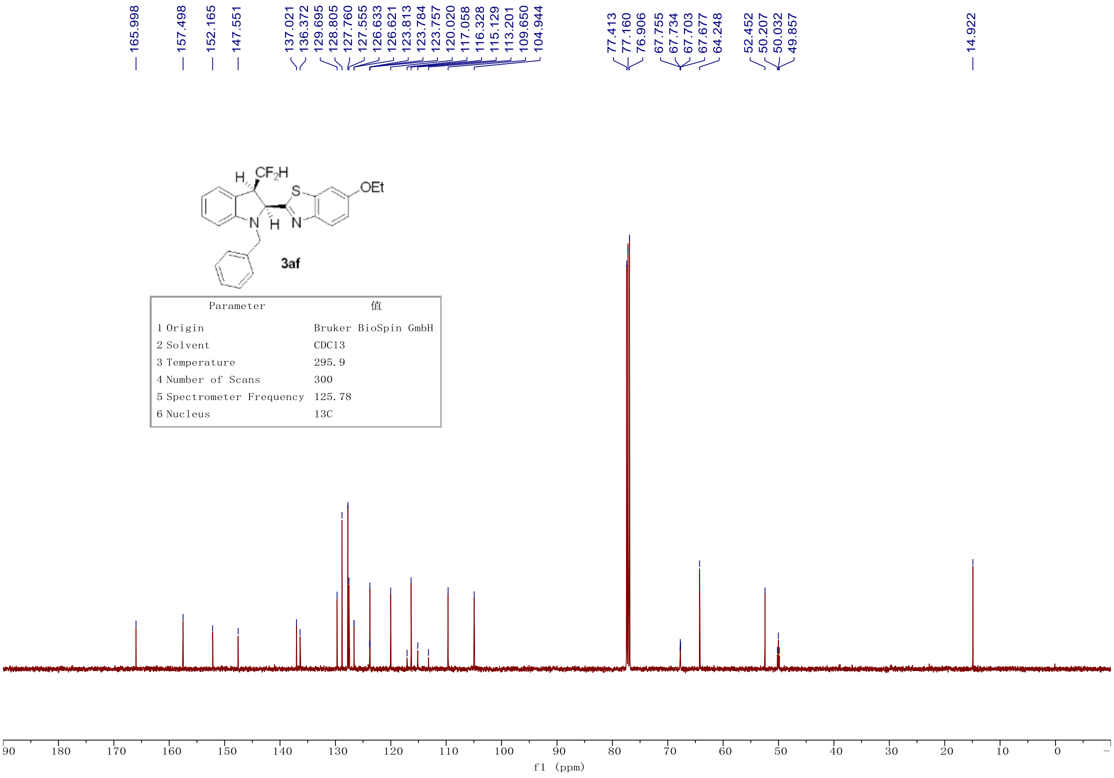

Figure S112. ^{13}C-NMR of **3af**

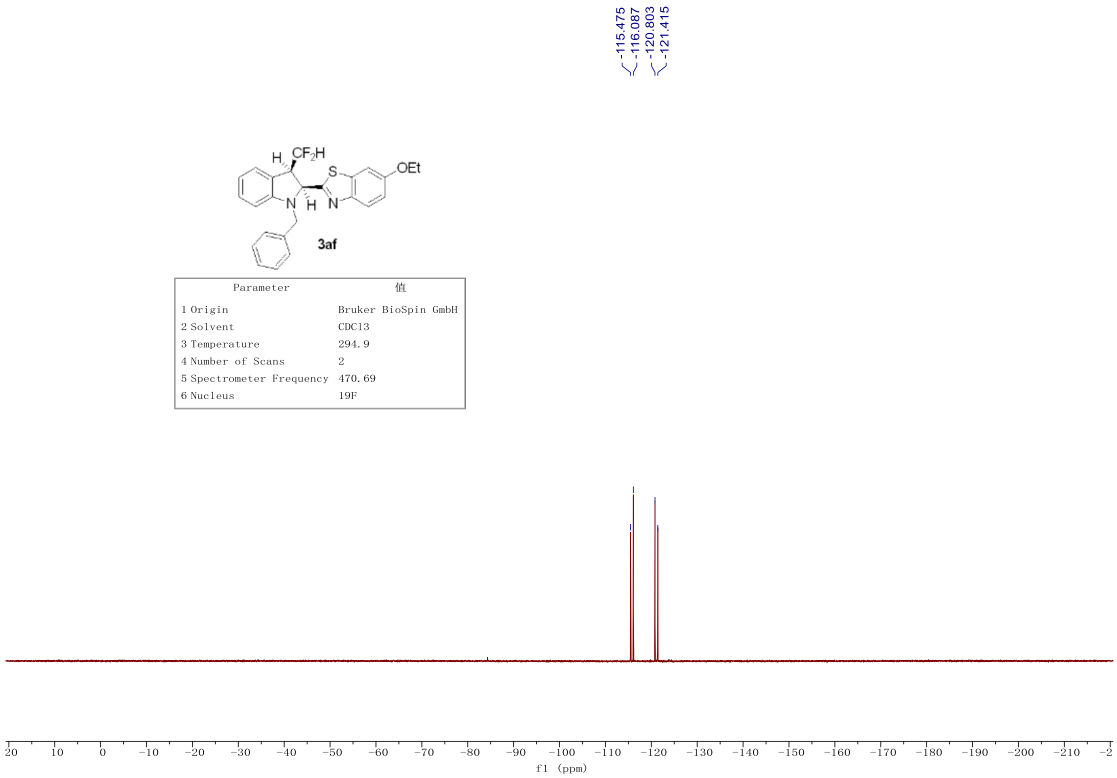

Figure S113. ^{19}F-NMR of **3af**

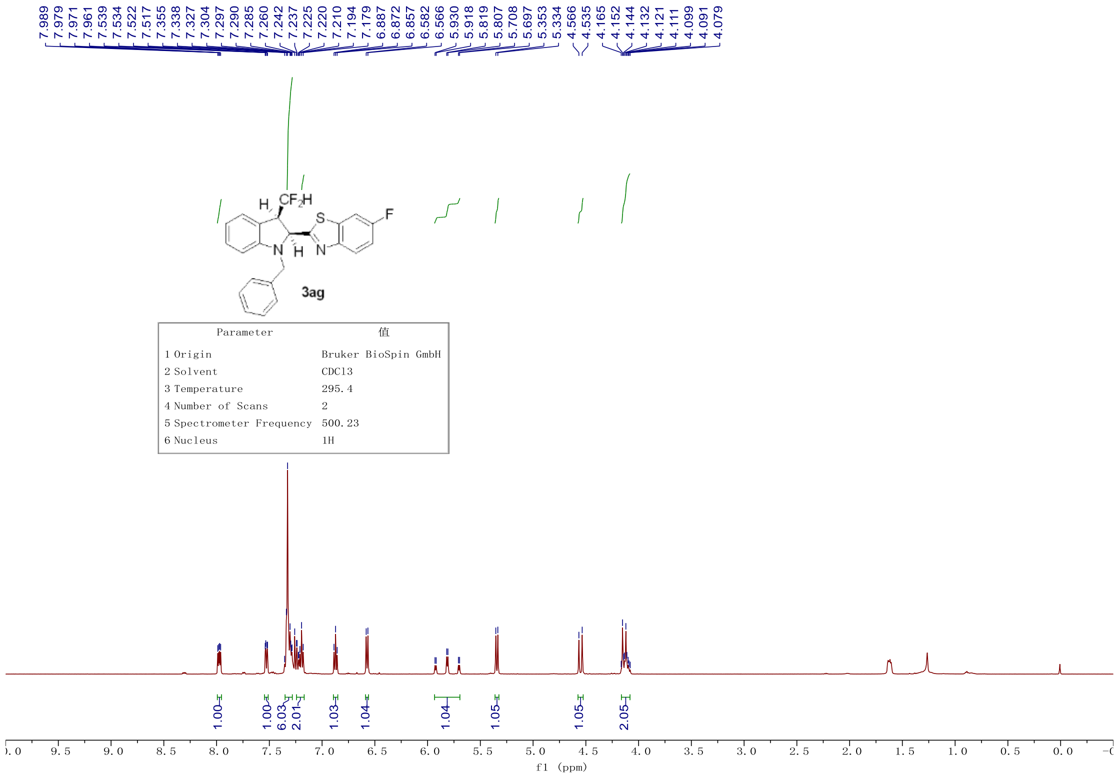

Figure S114. ^{1}H-NMR of **3ag**

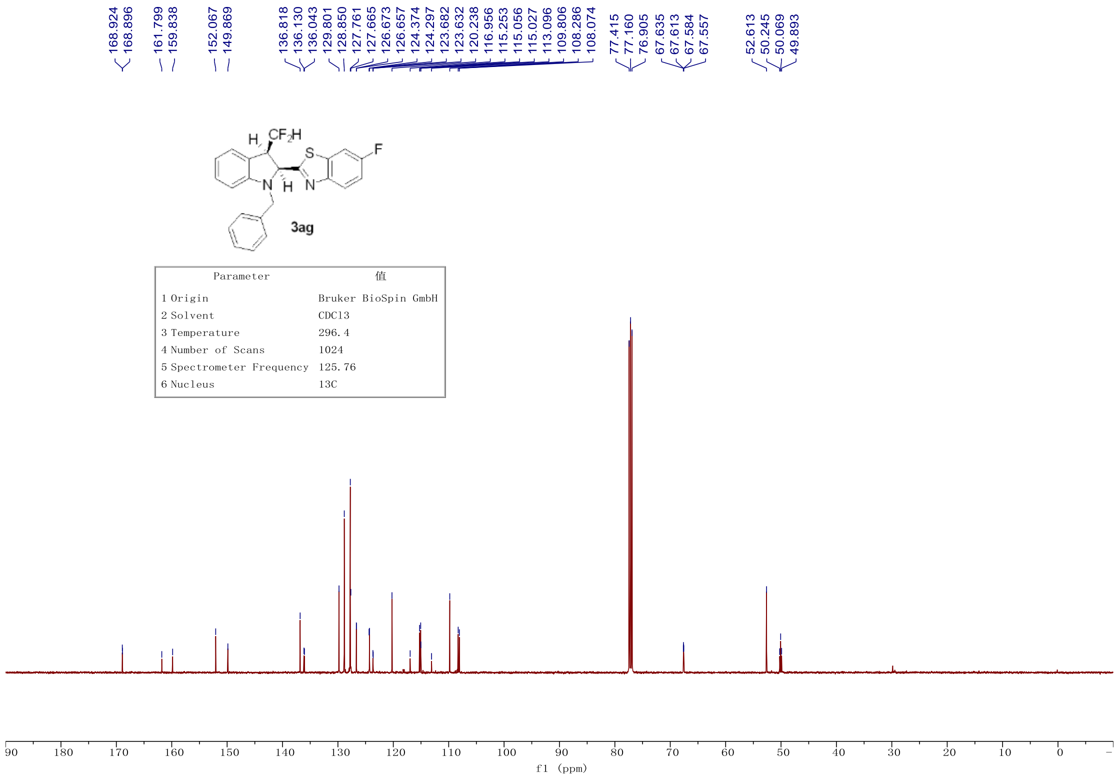

Parameter	值
1 Origin	Bruker BioSpin GmbH
2 Solvent	CDCl3
3 Temperature	296.4
4 Number of Scans	1024
5 Spectrometer Frequency	125.76
6 Nucleus	13C

Figure S115. ^{13}C-NMR of **3ag**

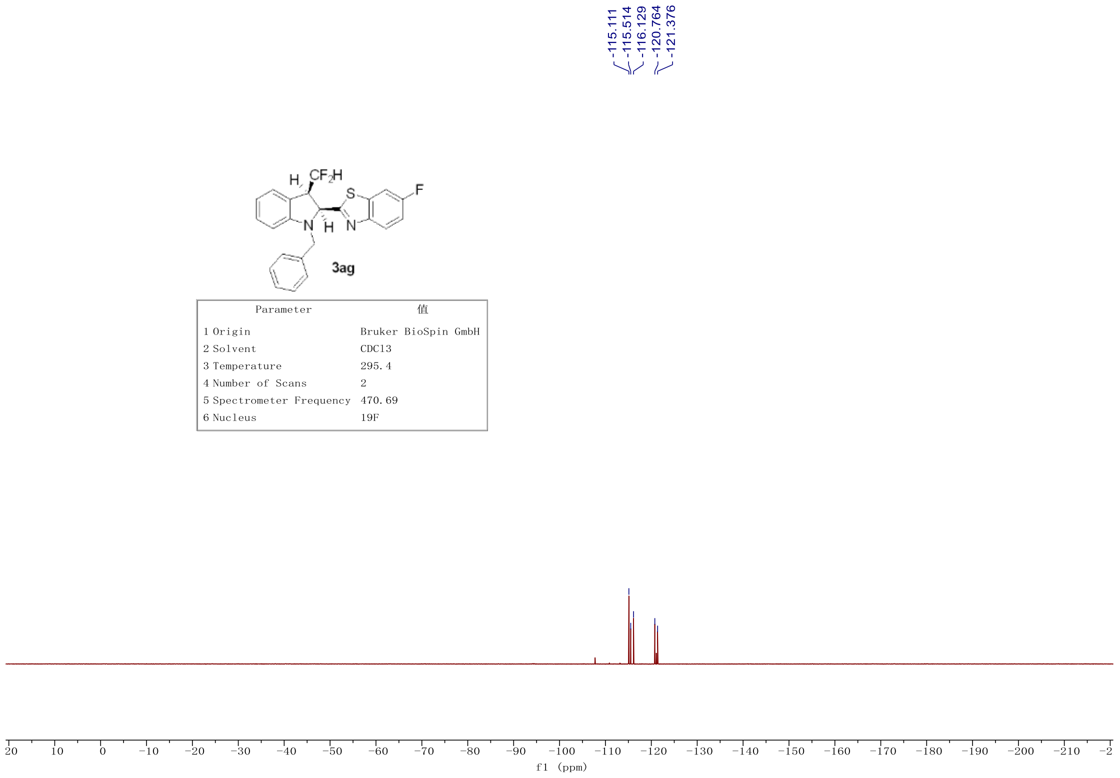

Parameter	值
1 Origin	Bruker BioSpin GmbH
2 Solvent	CDCl3
3 Temperature	295.4
4 Number of Scans	2
5 Spectrometer Frequency	470.69
6 Nucleus	19F

Figure S116. ^{19}F-NMR of **3ag**

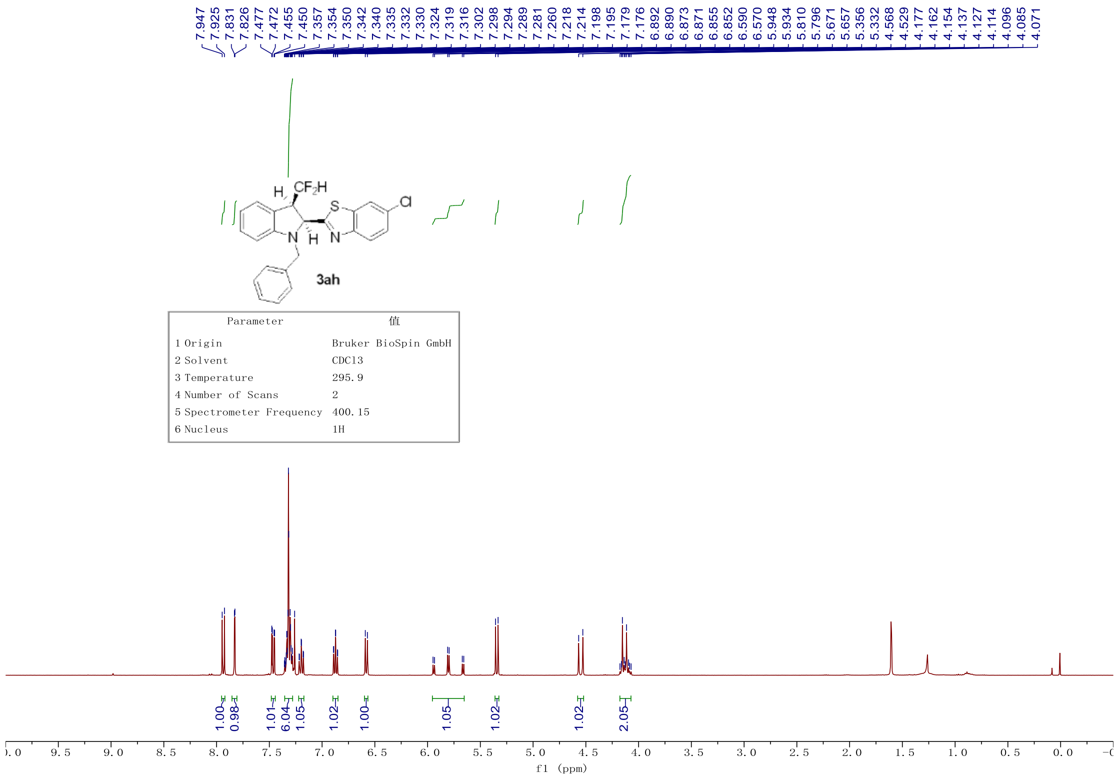

Figure S117. ^{1}H-NMR of **3ah**

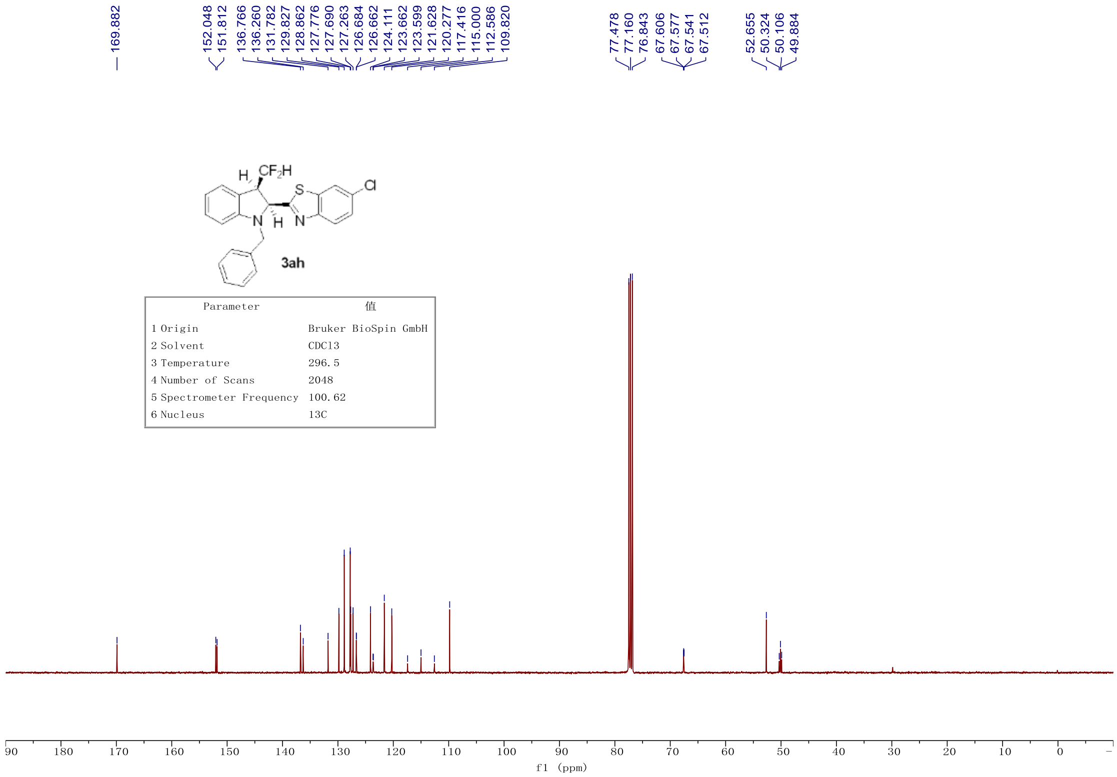

Figure S118. ^{13}C-NMR of **3ah**

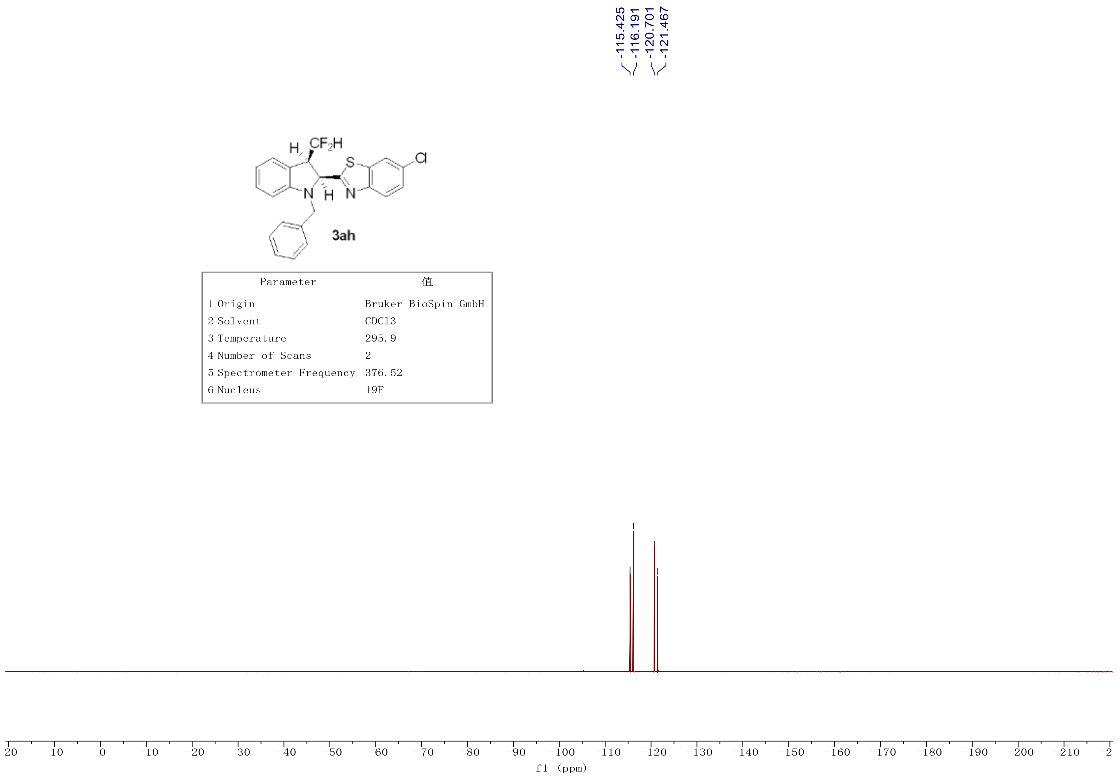

Figure S119. ^{19}F-NMR of **3ah**

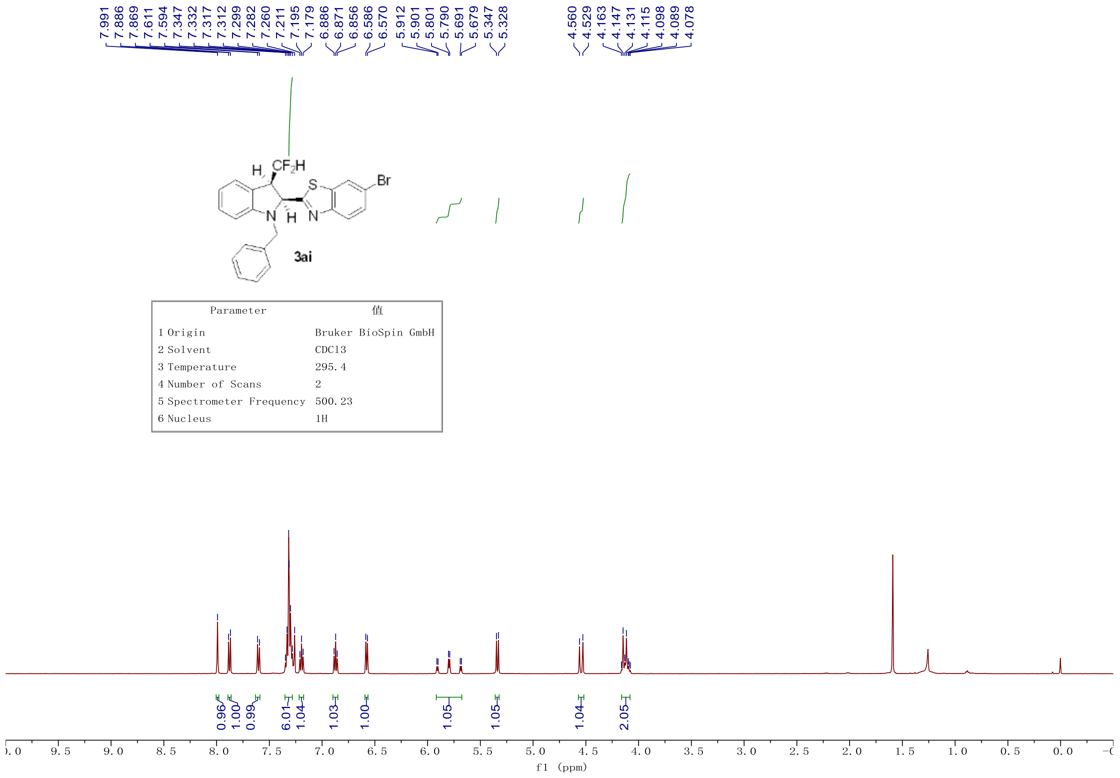

Figure S120. ^{1}H-NMR of **3ai**

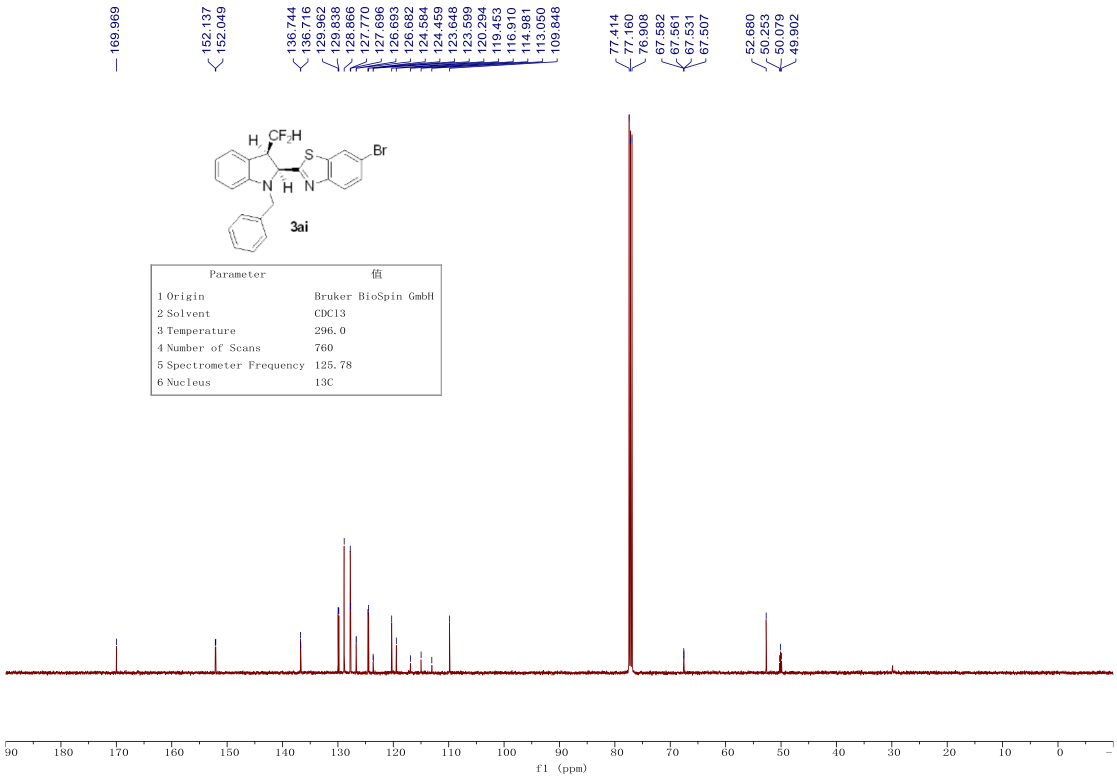

Figure S121. ^{13}C-NMR of **3ai**

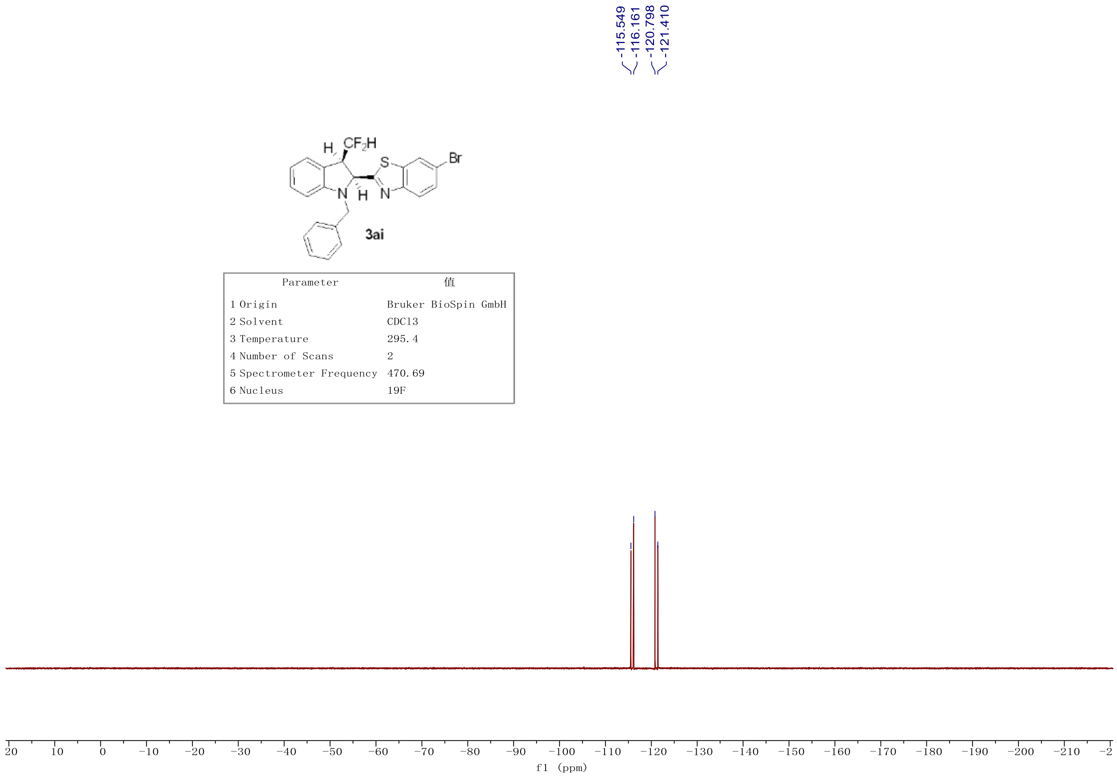

Figure S122. ^{19}F-NMR of **3ai**

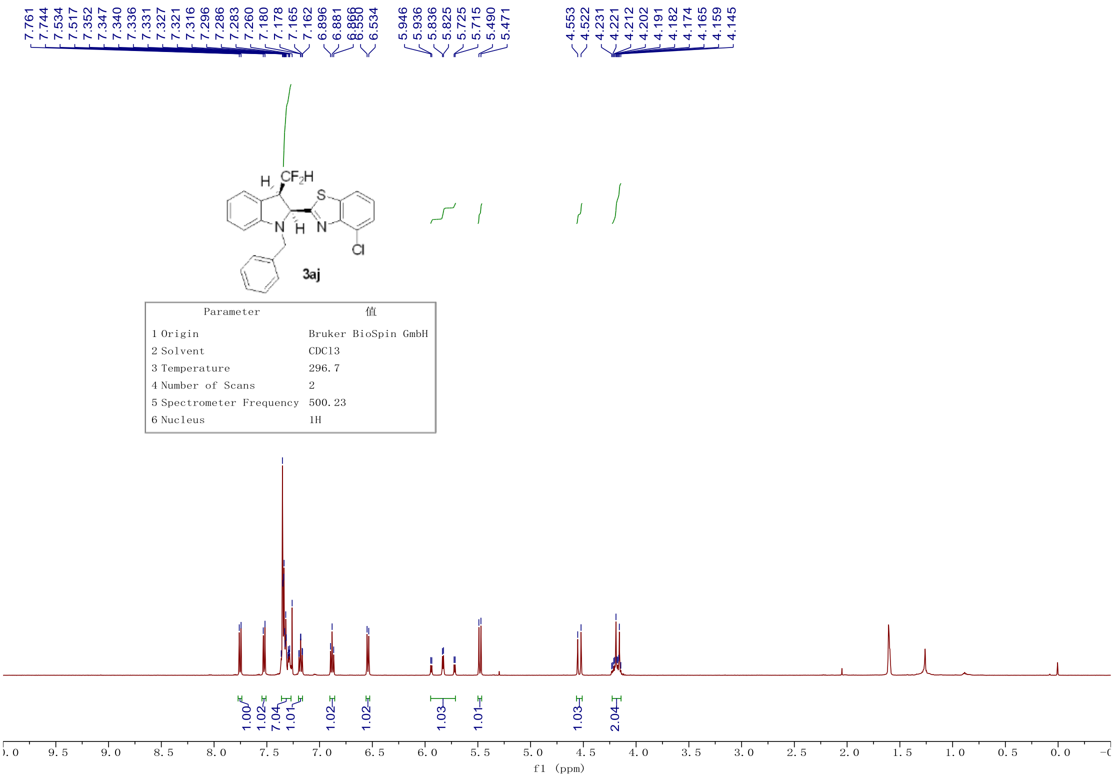

Figure S123. ^{1}H-NMR of **3aj**

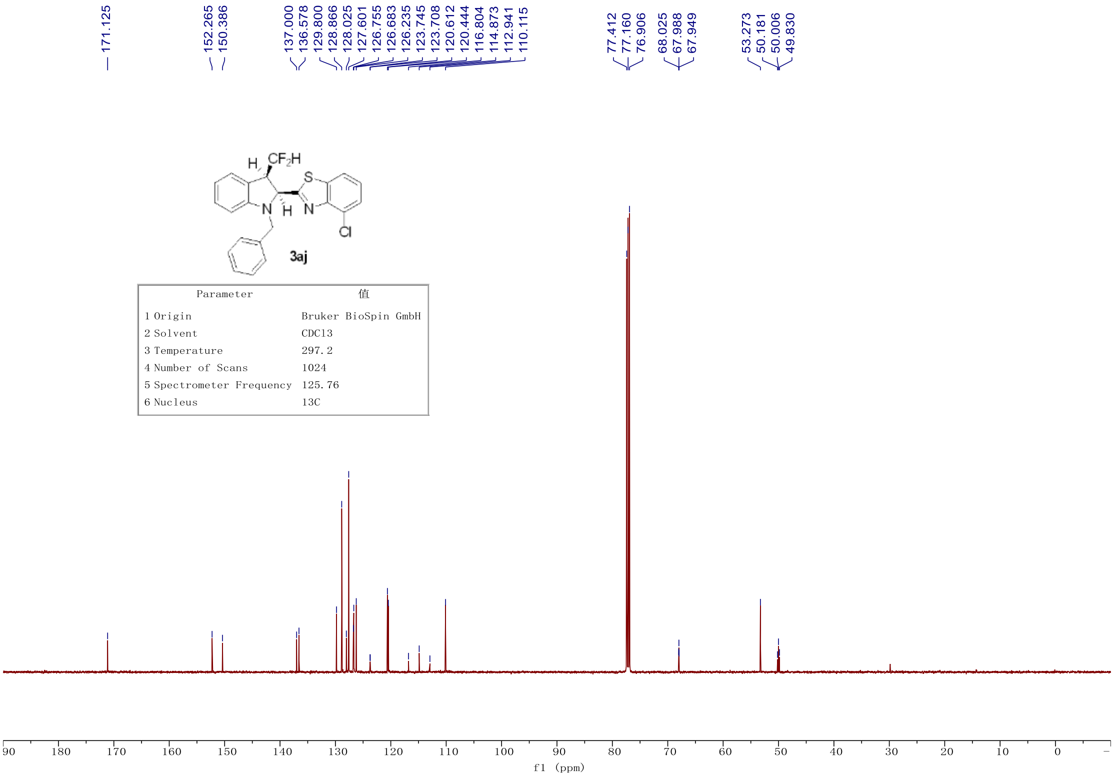

Figure S124. ^{13}C-NMR of **3aj**

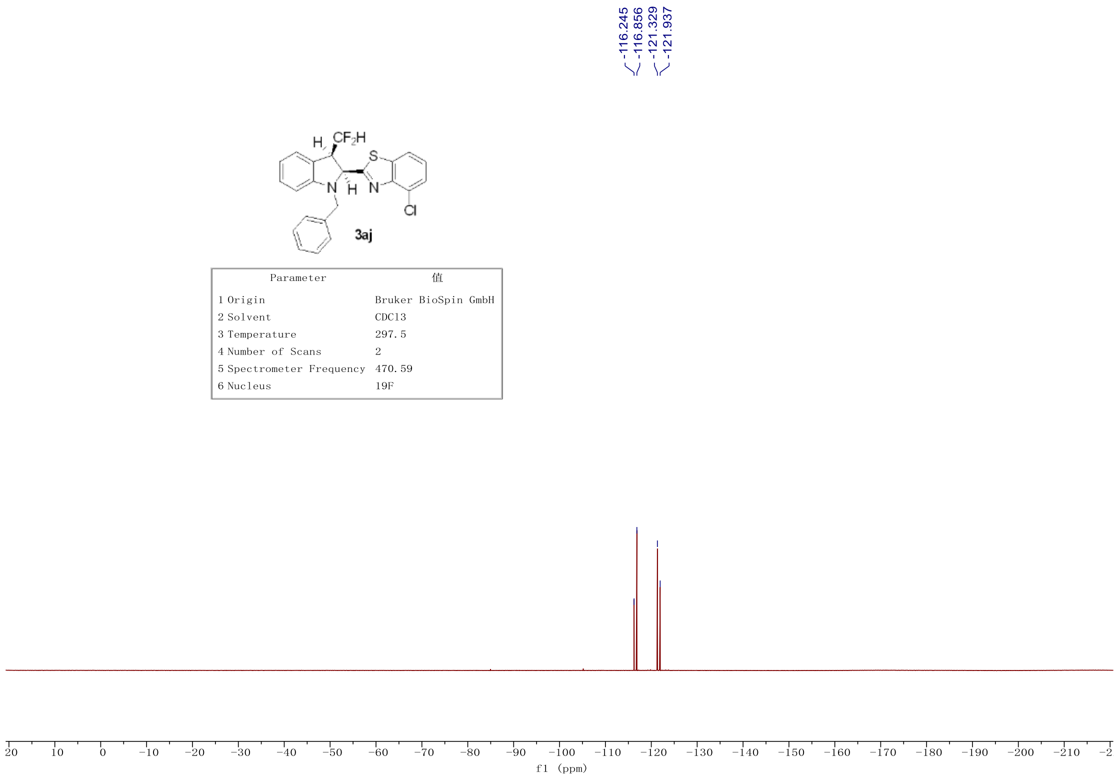

Figure S125. ^{19}F-NMR of **3aj**

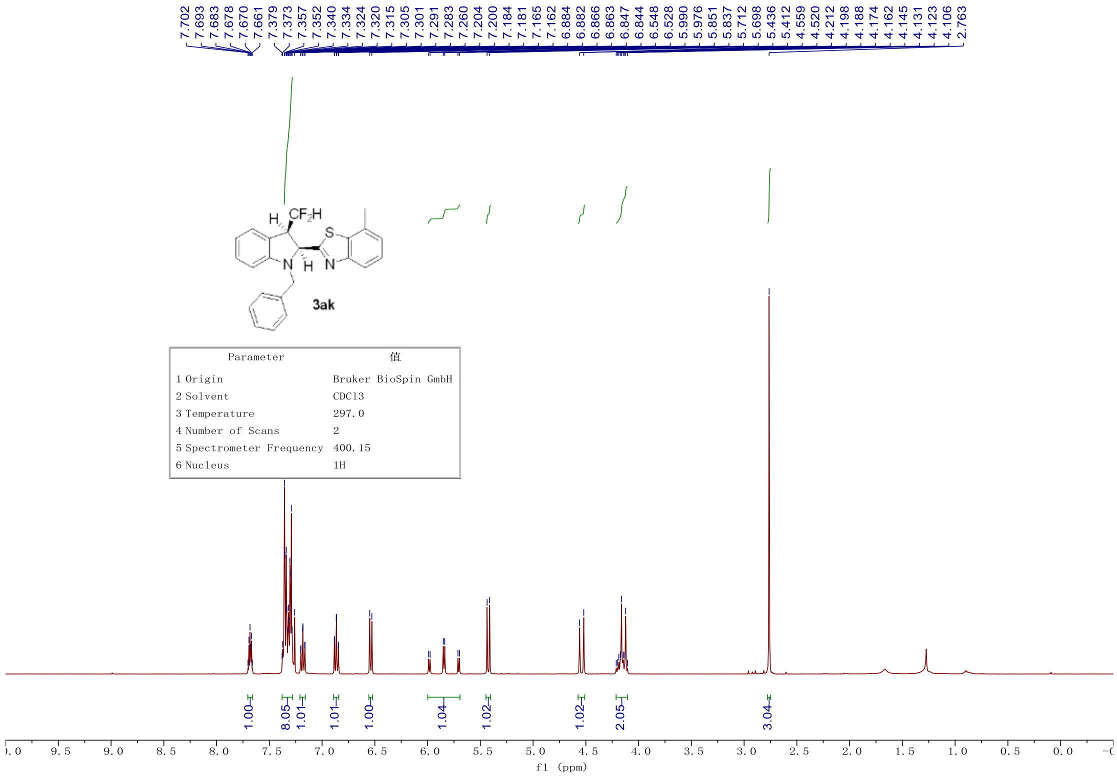

Figure S126. ^{1}H-NMR of **3ak**

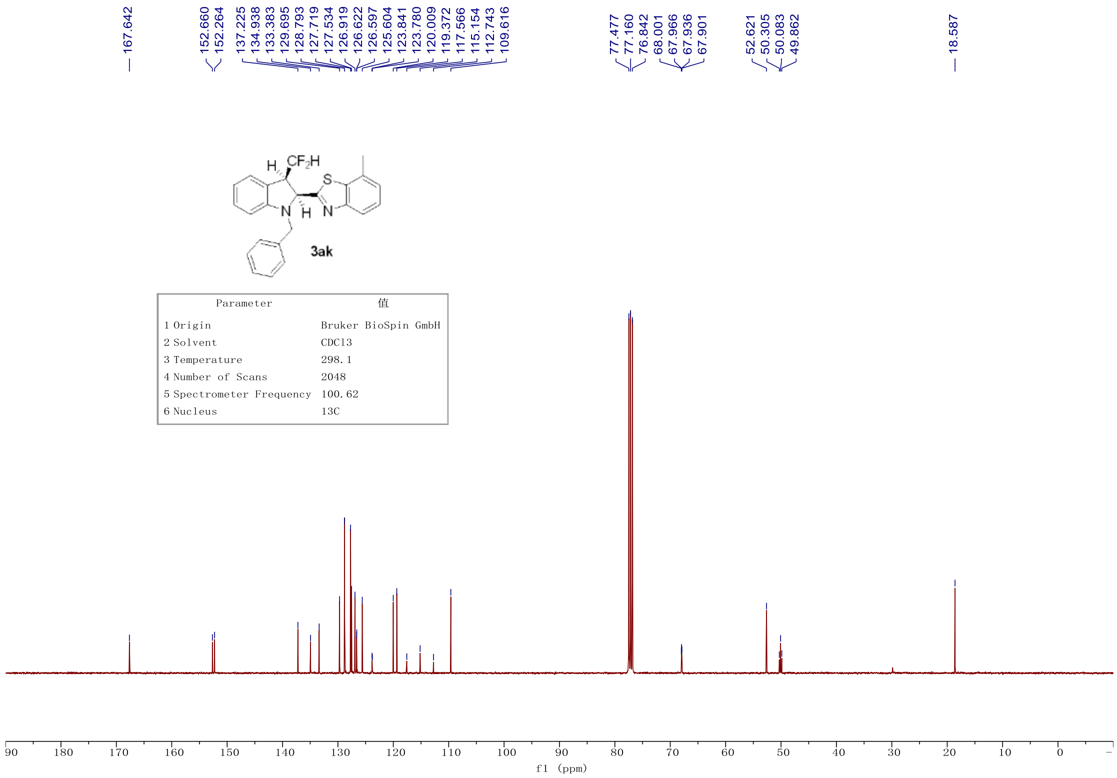

Figure S127. ^{13}C-NMR of **3ak**

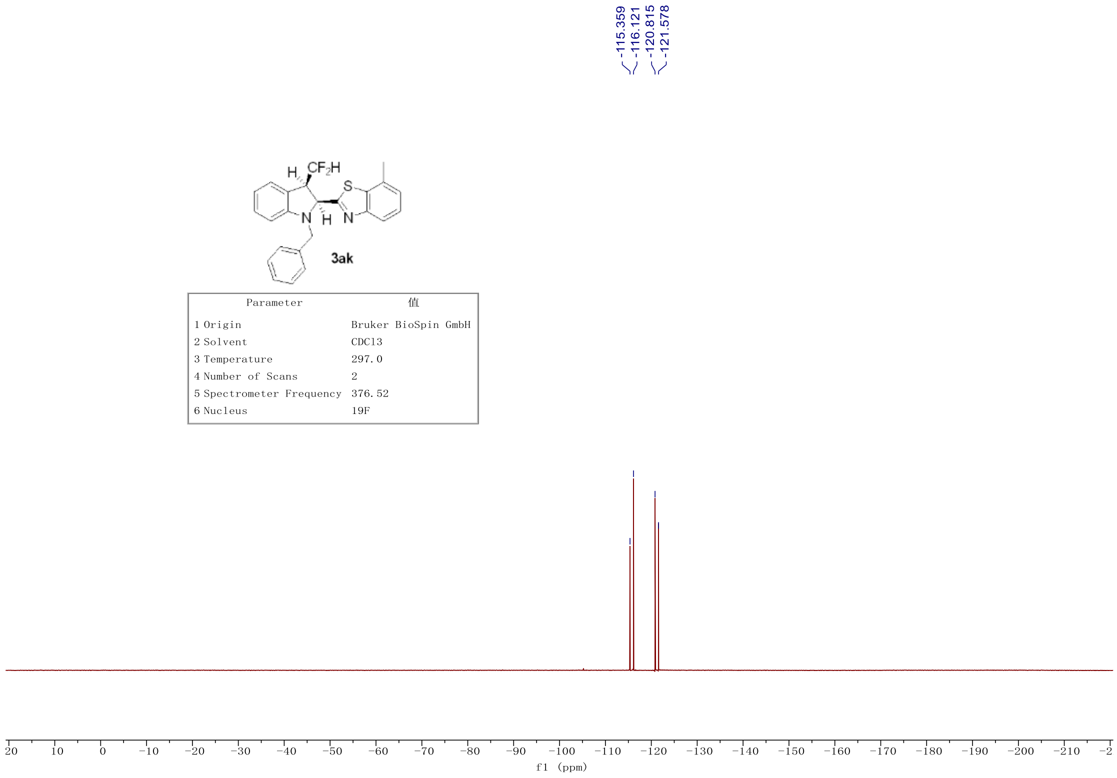

Figure S128. ^{19}F-NMR of **3ak**

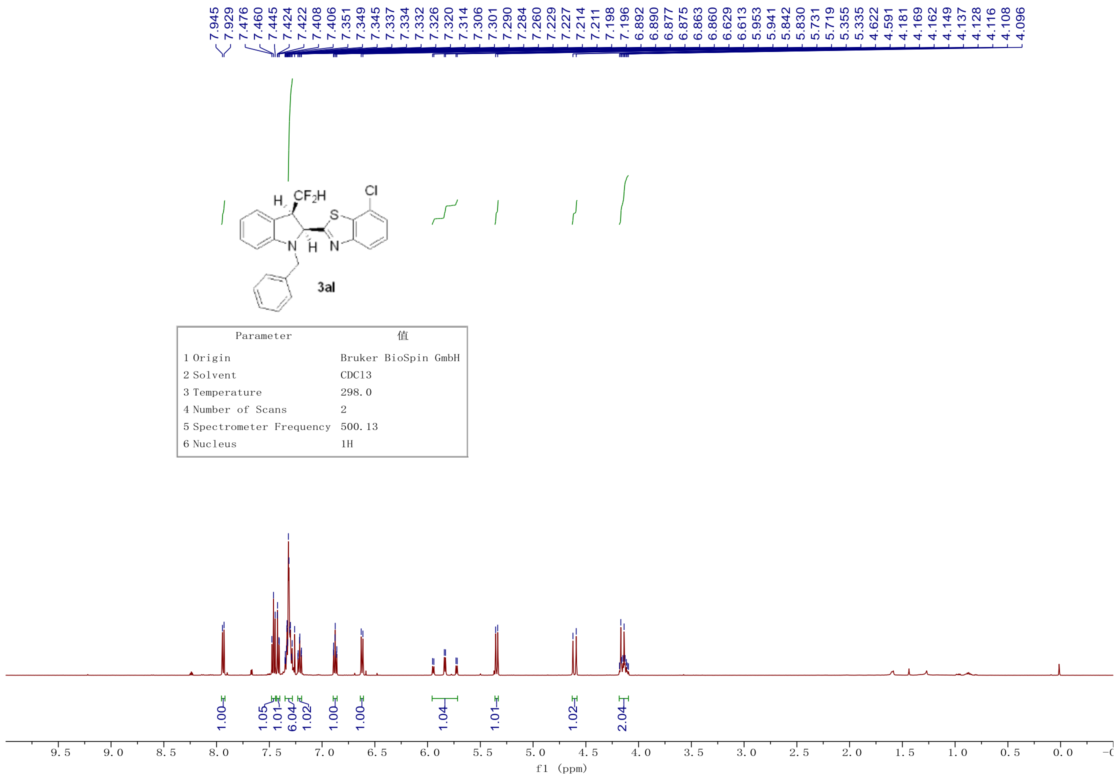

Figure S129. ^{1}H-NMR of **3al**

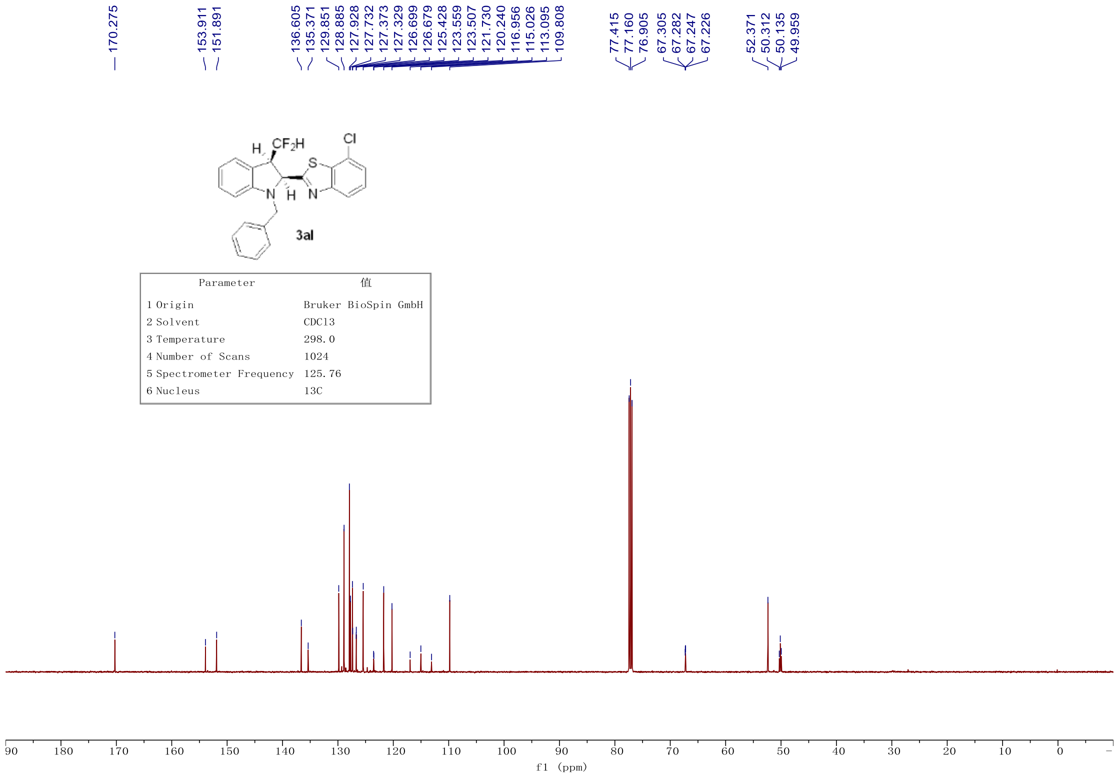

Figure S130. ^{13}C-NMR of **3al**

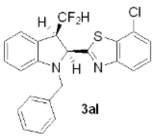

Parameter	值
1 Origin	Bruker BioSpin GmbH
2 Solvent	CDCl3
3 Temperature	298.1
4 Number of Scans	2
5 Spectrometer Frequency	470.59
6 Nucleus	19F

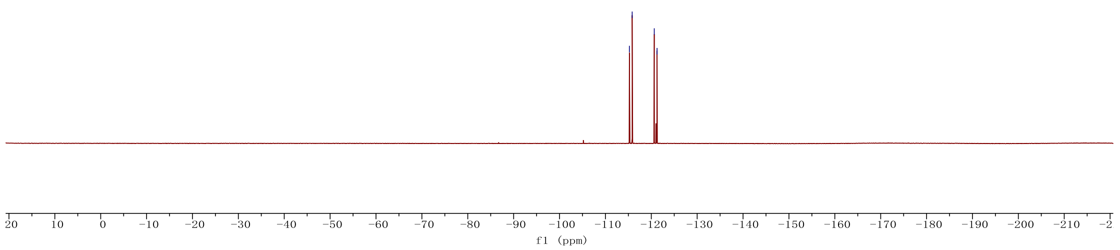

Figure S131. ^{19}F-NMR of **3al**

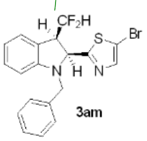

Parameter	值
1 Origin	Bruker BioSpin GmbH
2 Solvent	CDCl3
3 Temperature	298.1
4 Number of Scans	2
5 Spectrometer Frequency	400.15
6 Nucleus	1H

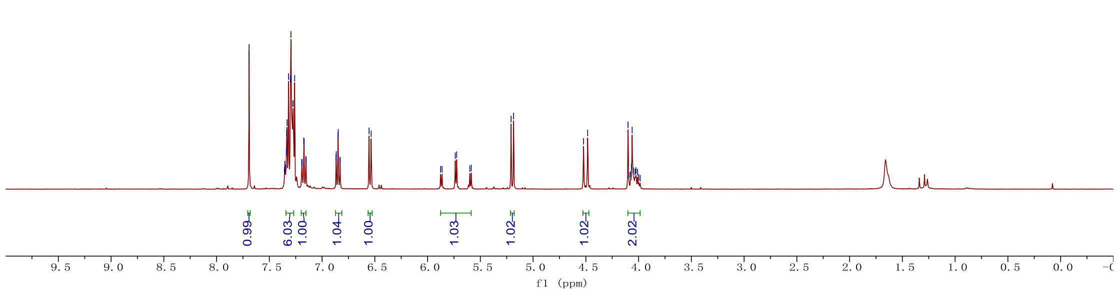

Figure S132. ^{1}H-NMR of **3am**

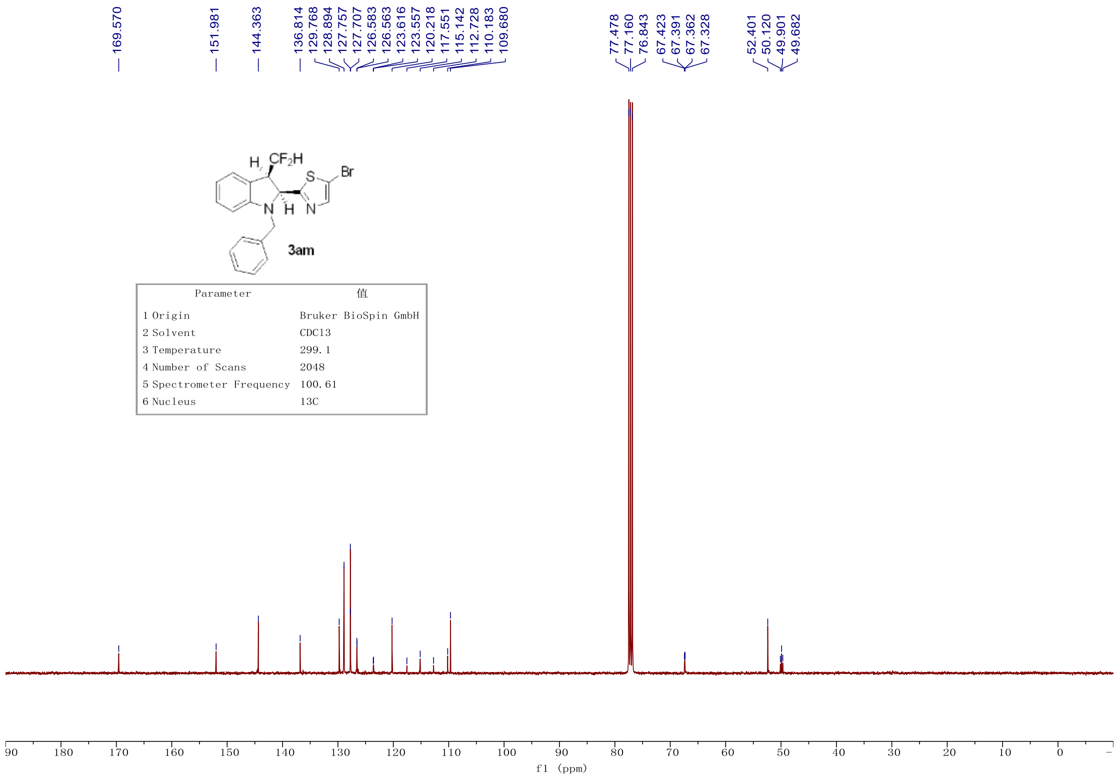

Figure S133. ^{13}C-NMR of **3am**

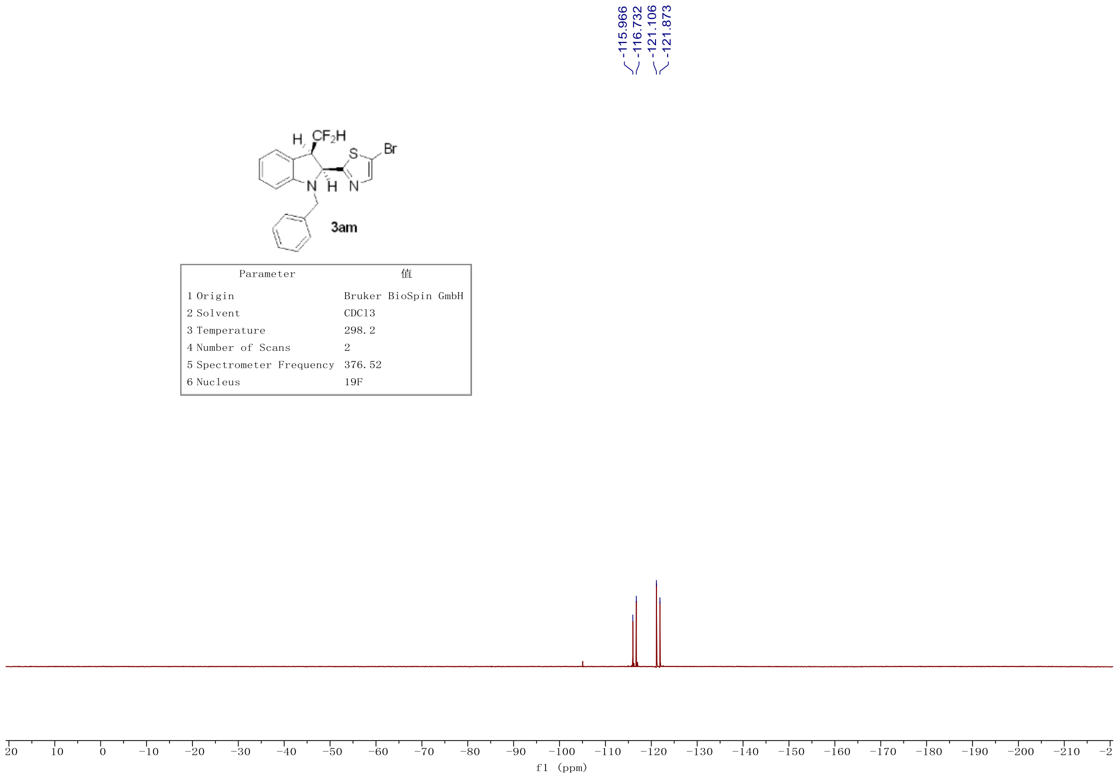

Figure S134. ^{19}F-NMR of **3am**

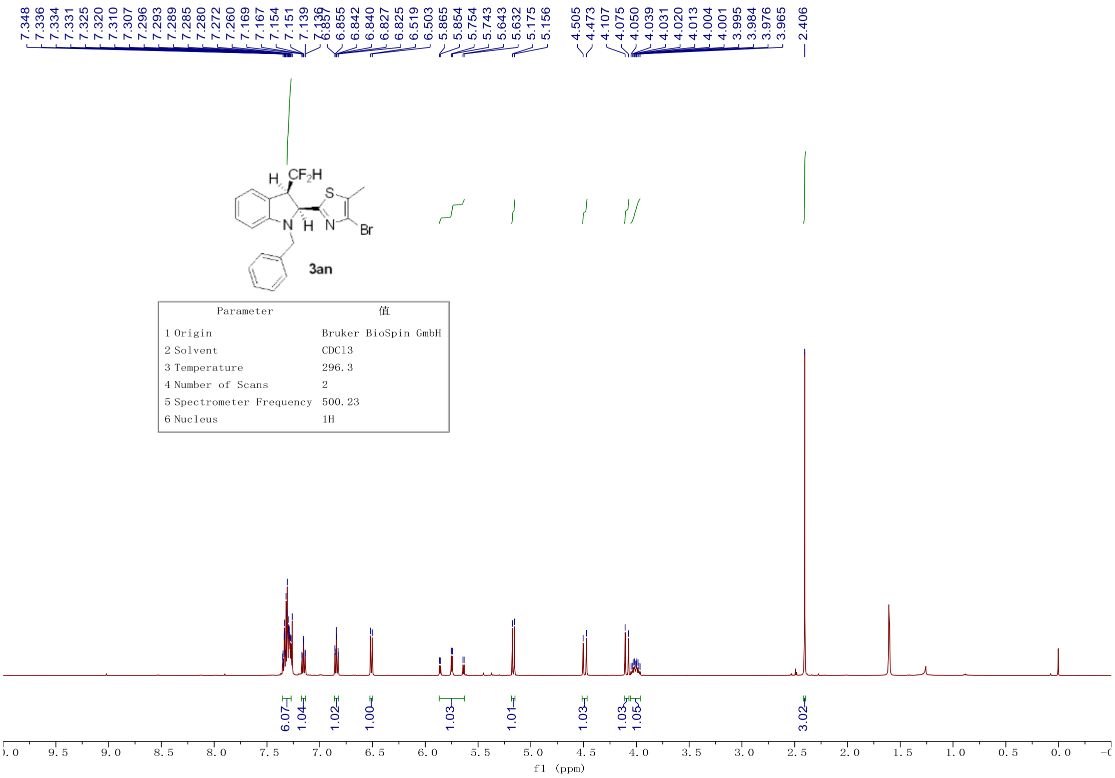

Figure S135. ^{1}H-NMR of **3an**

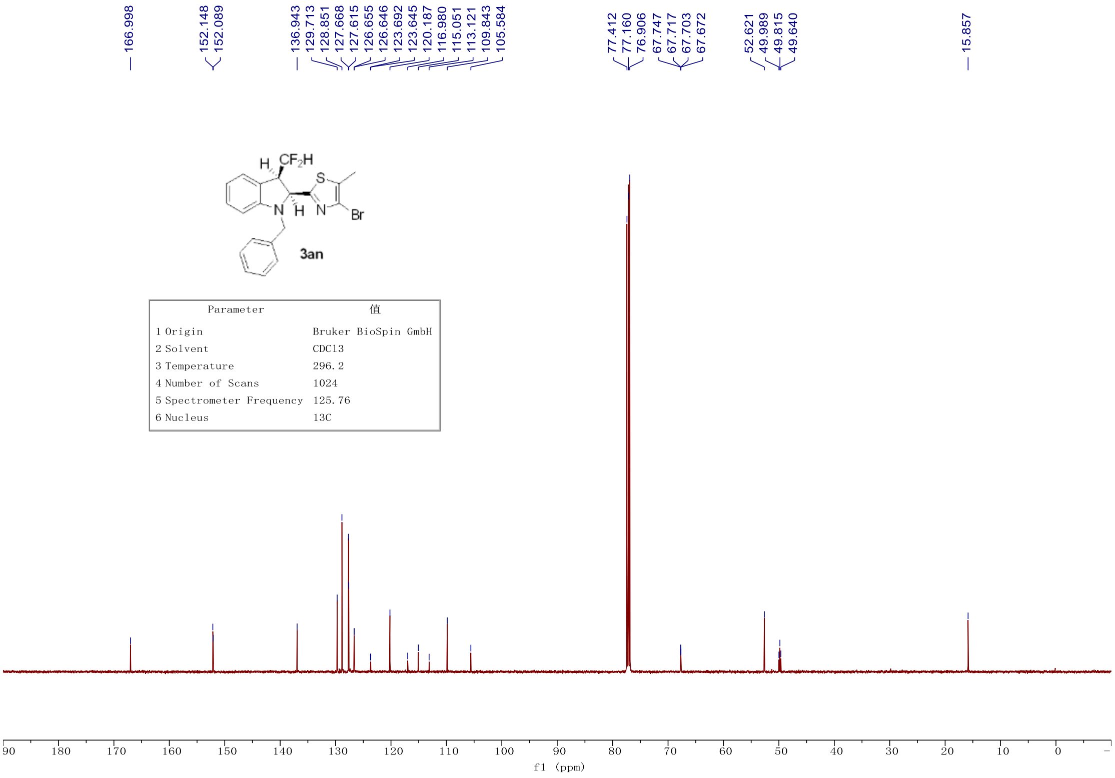

Figure S136. ^{13}C-NMR of **3an**

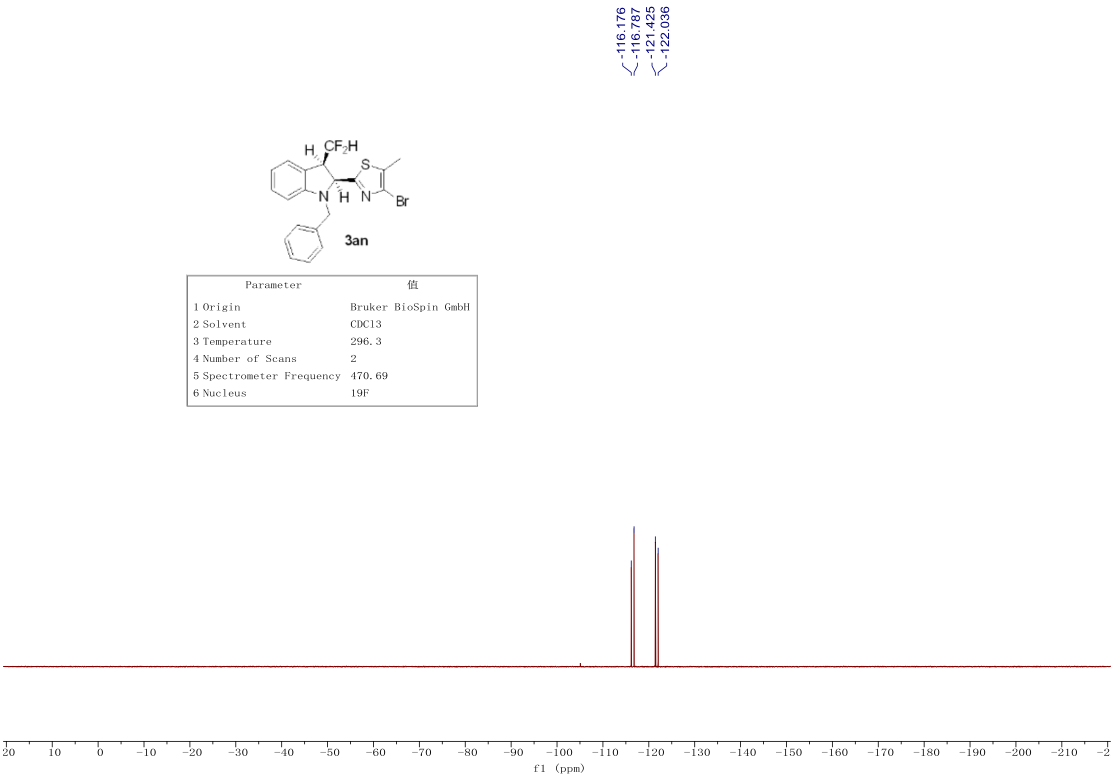

Figure S137. ^{19}F-NMR of **3an**

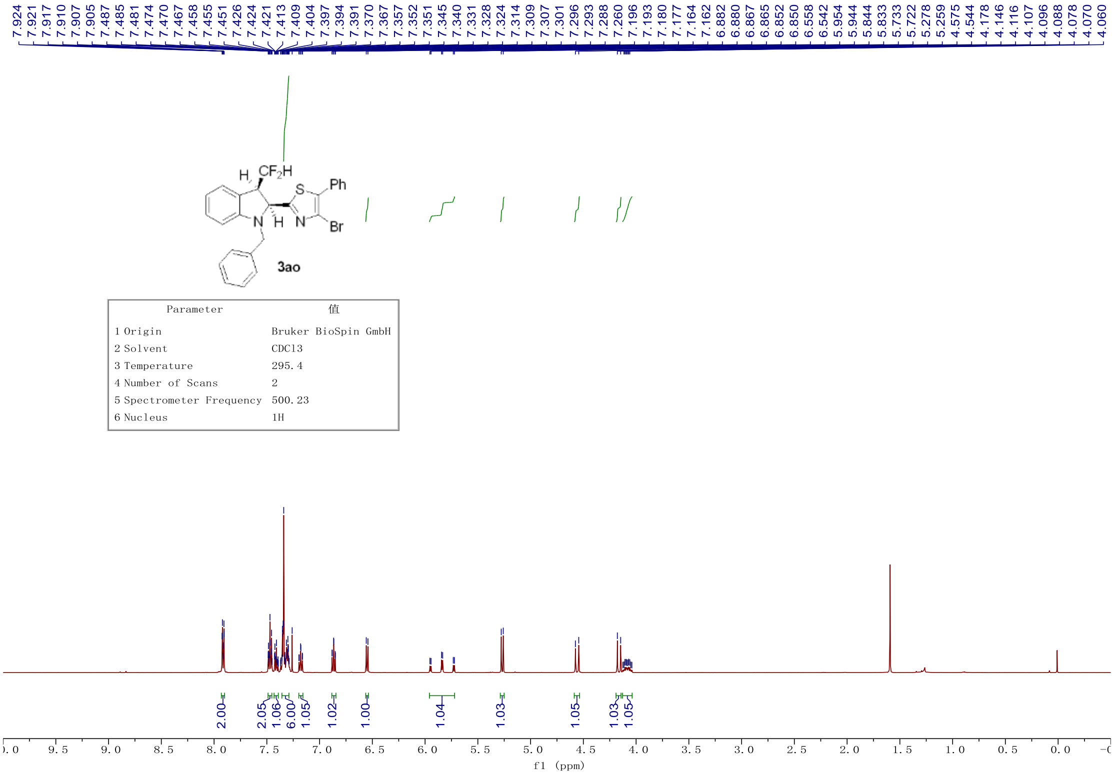

Figure S138. ^{1}H-NMR of **3ao**

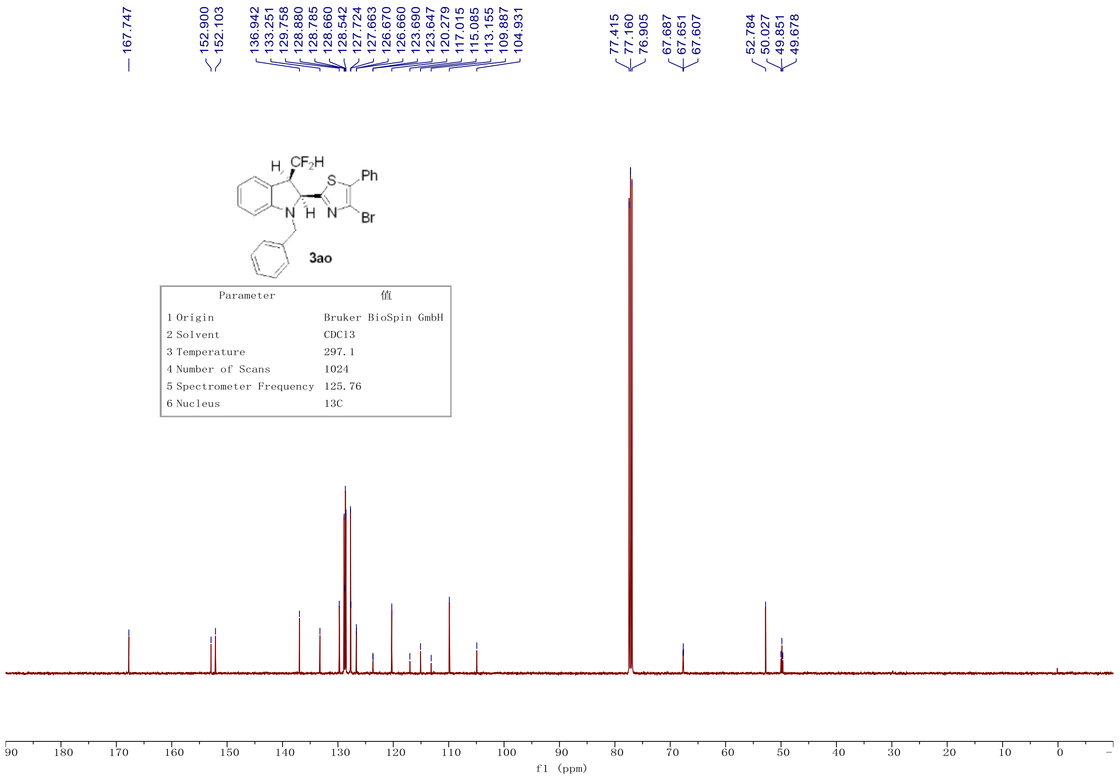

Figure S139. ^{13}C-NMR of **3ao**

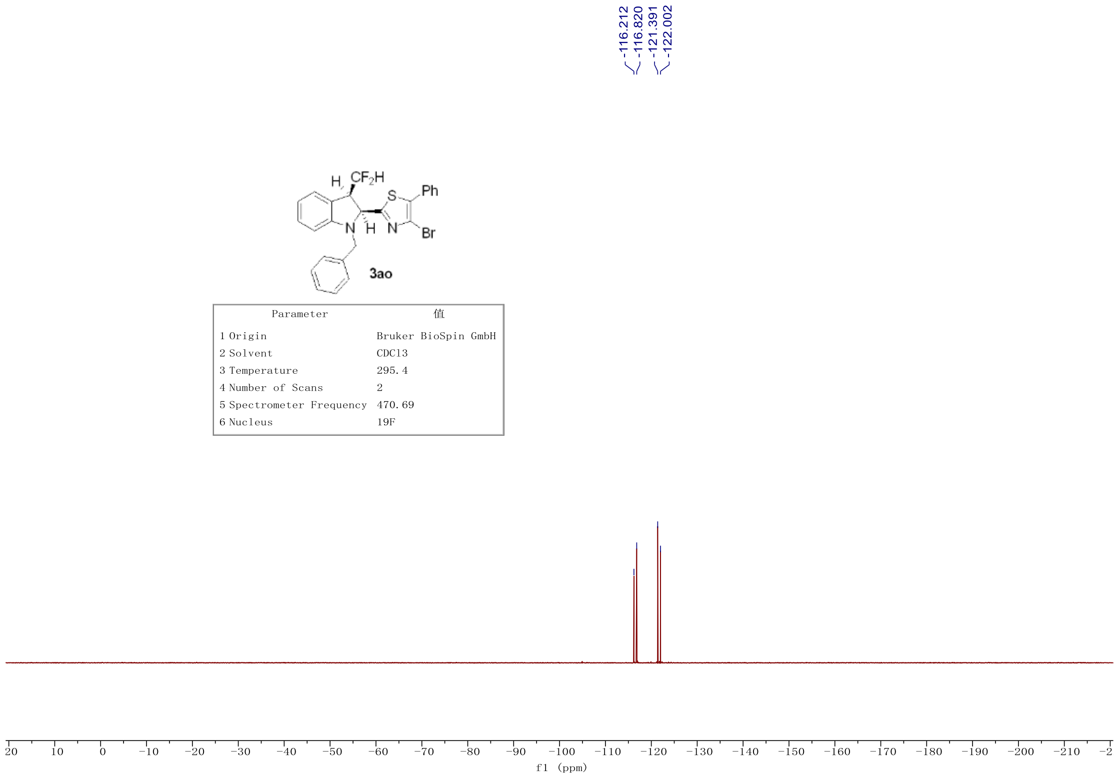

Figure S140. ^{19}F-NMR of **3ao**

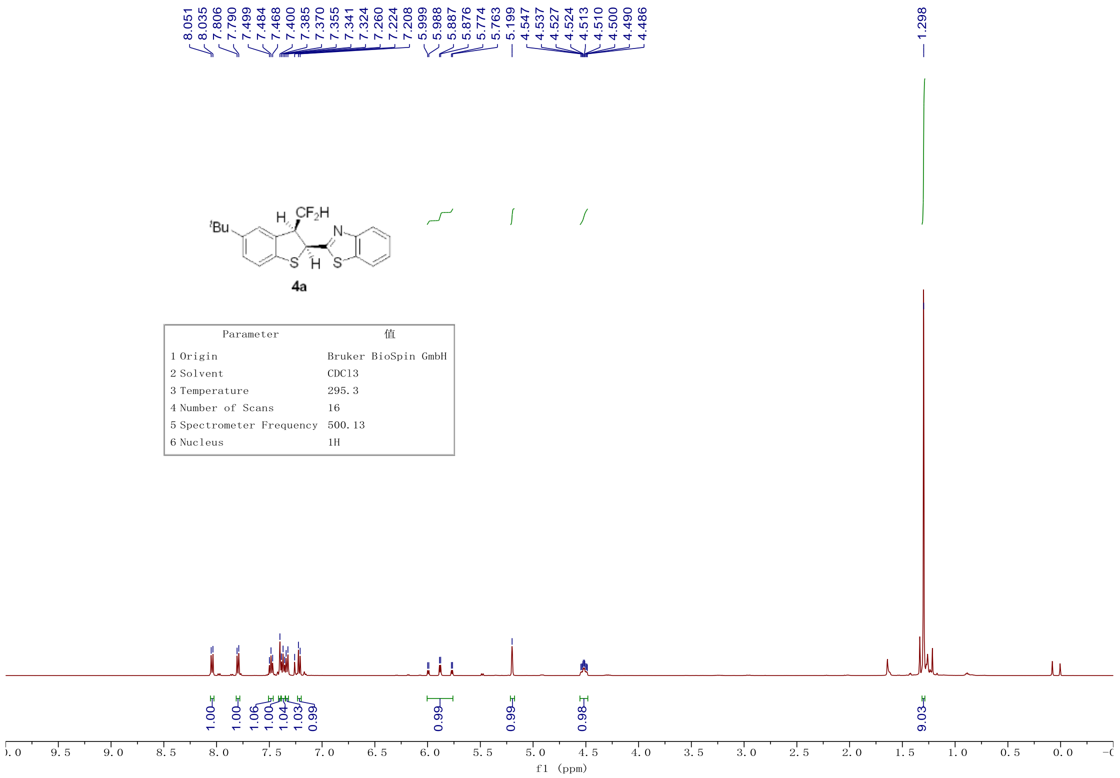

Figure S141. ^{1}H-NMR of **4a**

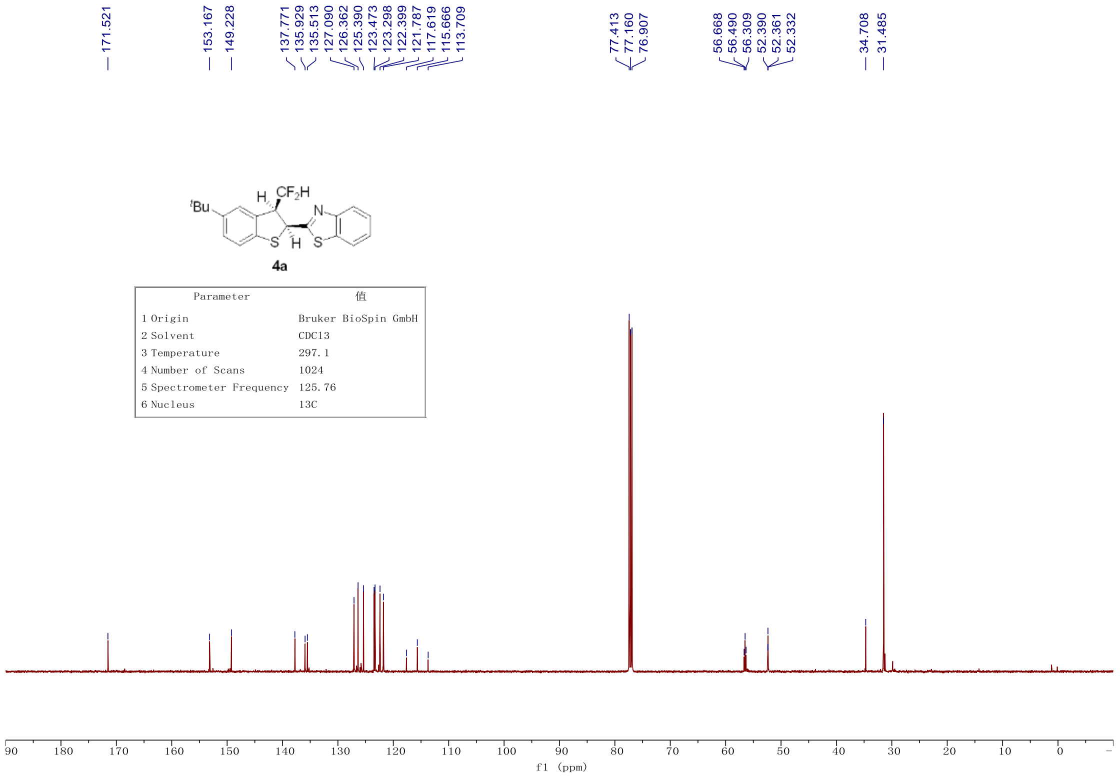

Figure S142. ^{13}C-NMR of **4a**

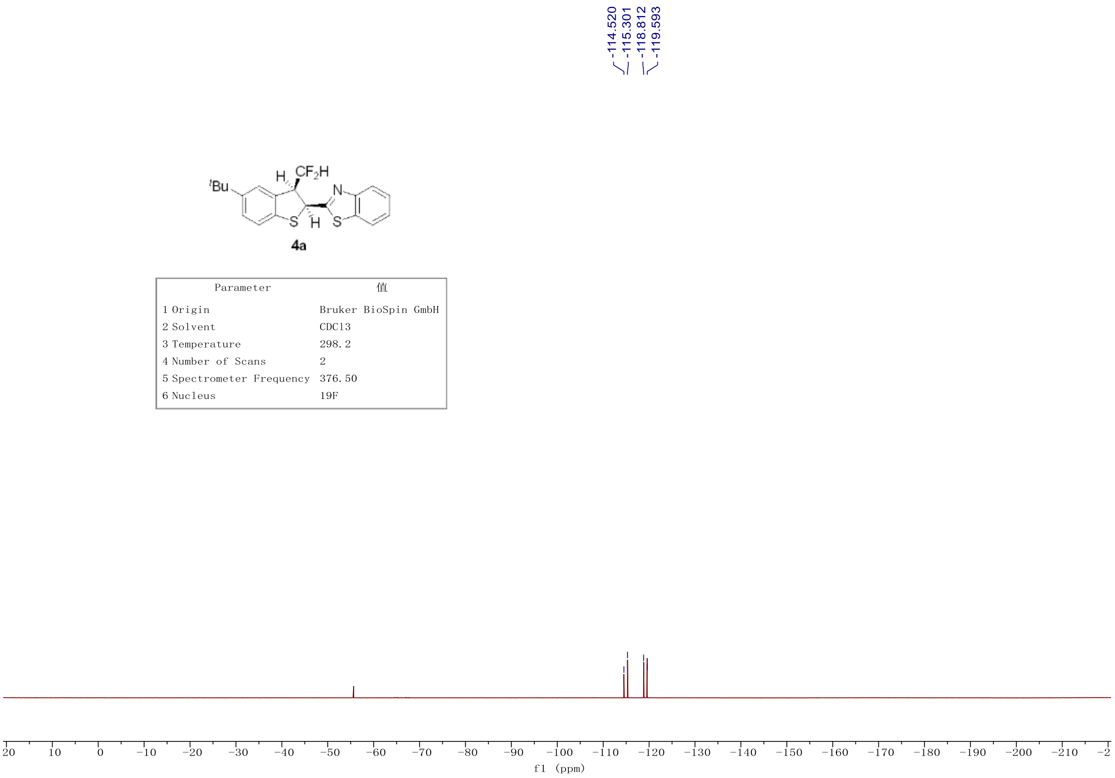

Figure S143. ^{19}F-NMR of **4a**

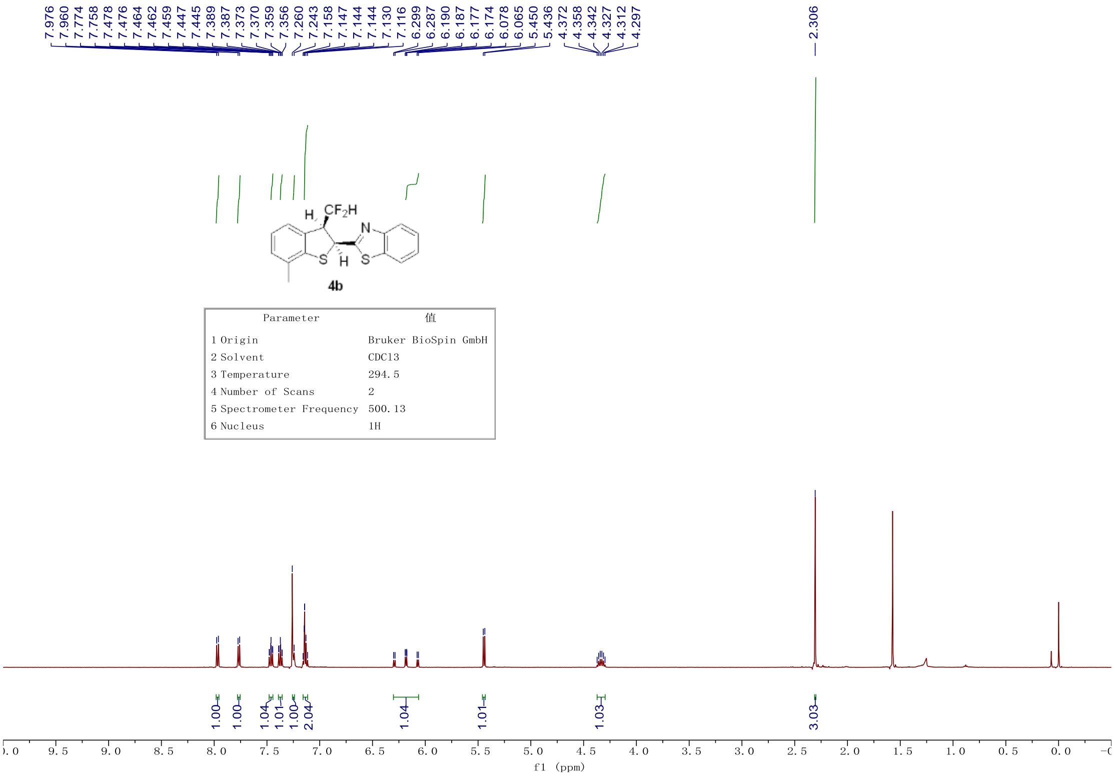

Figure S144. ^{1}H-NMR of **4b**

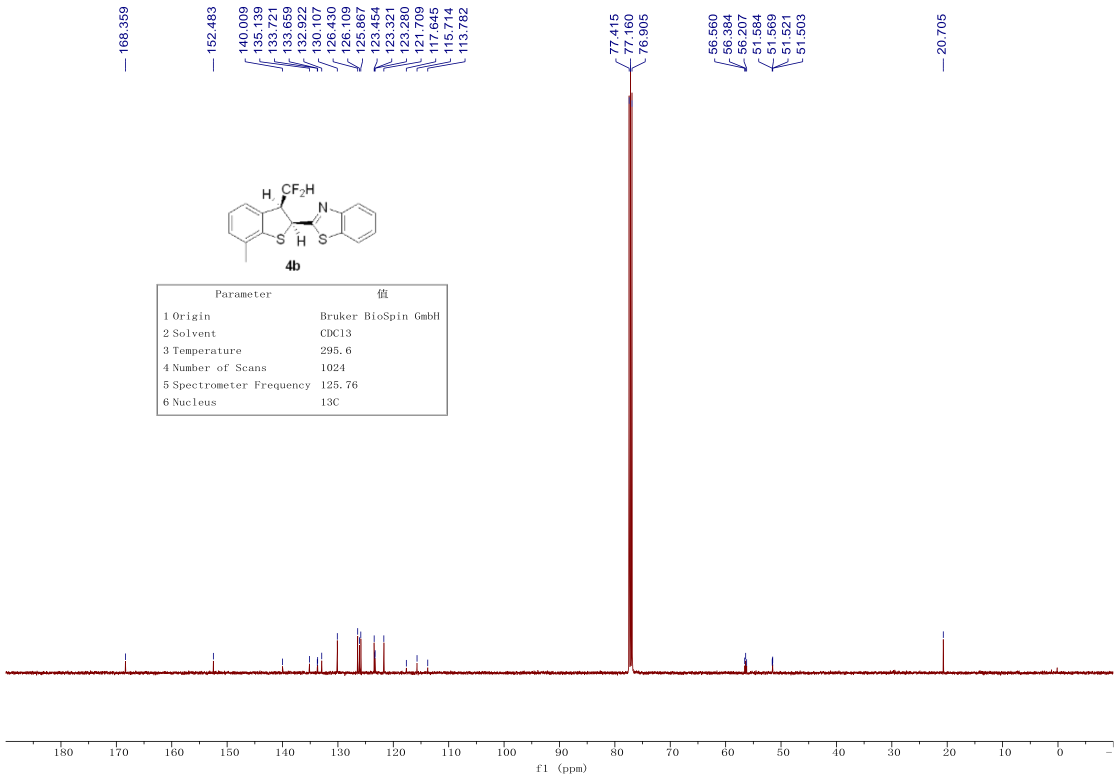

Figure S145. ^{13}C-NMR of **4b**

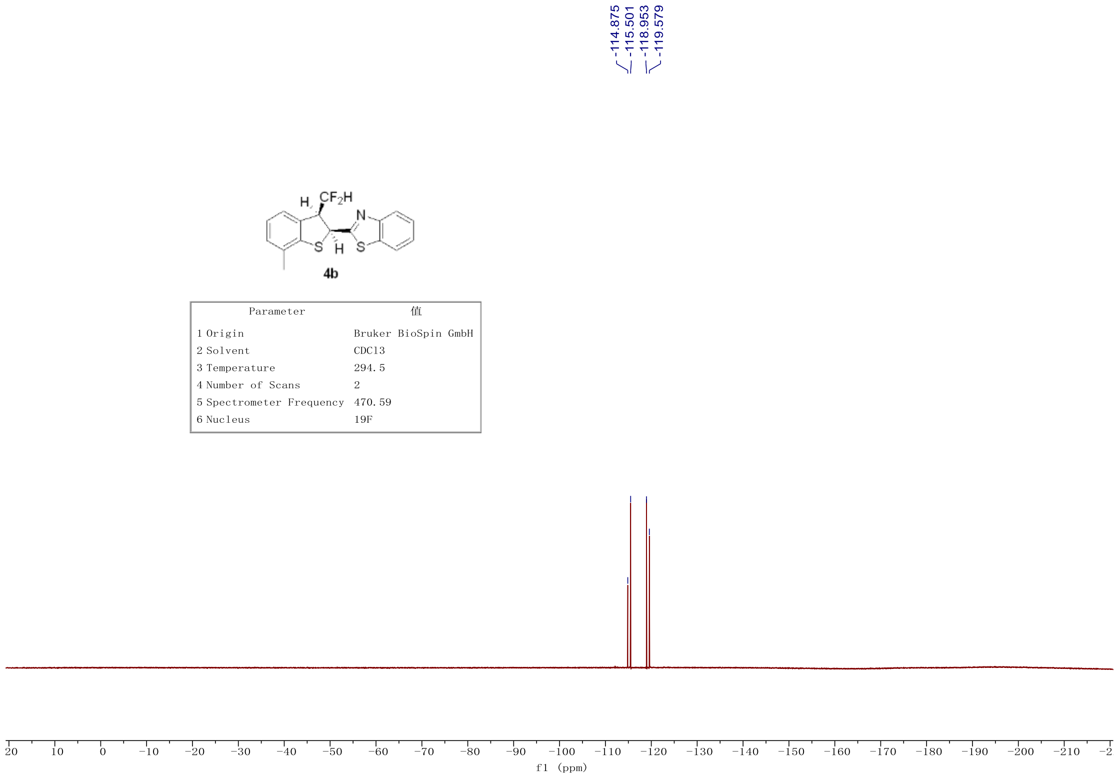

Figure S146. ^{19}F-NMR of **4b**

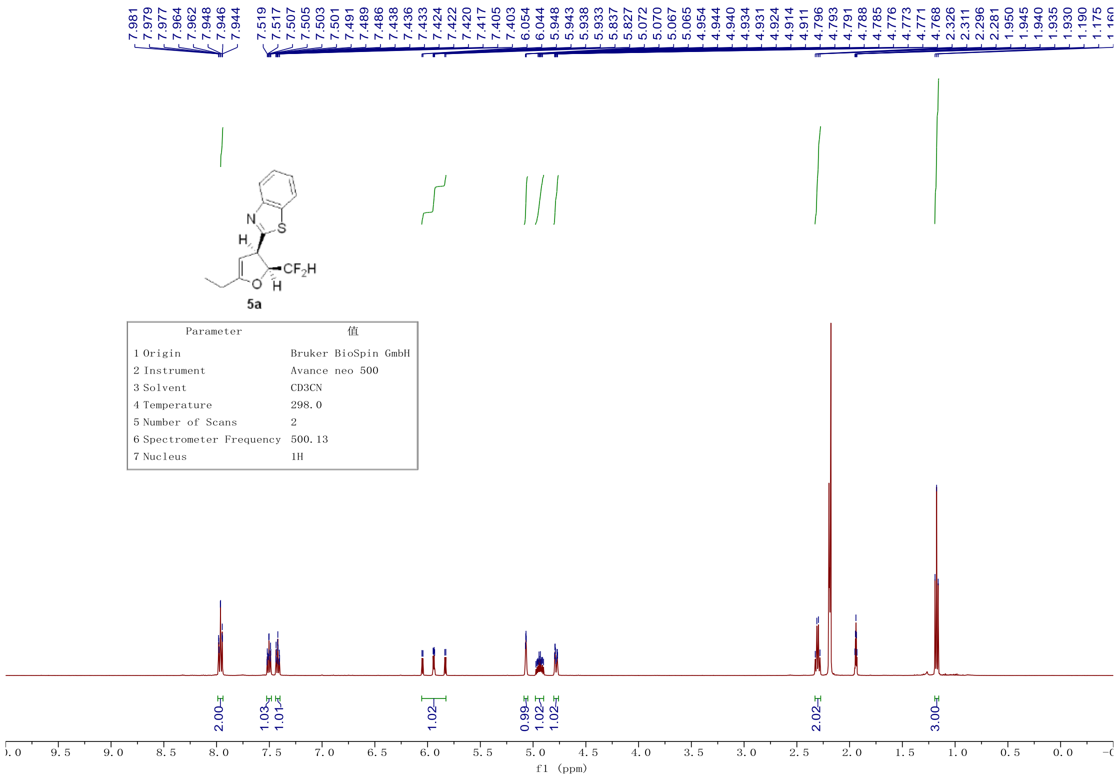

Figure S147. ^{1}H-NMR of **5a**

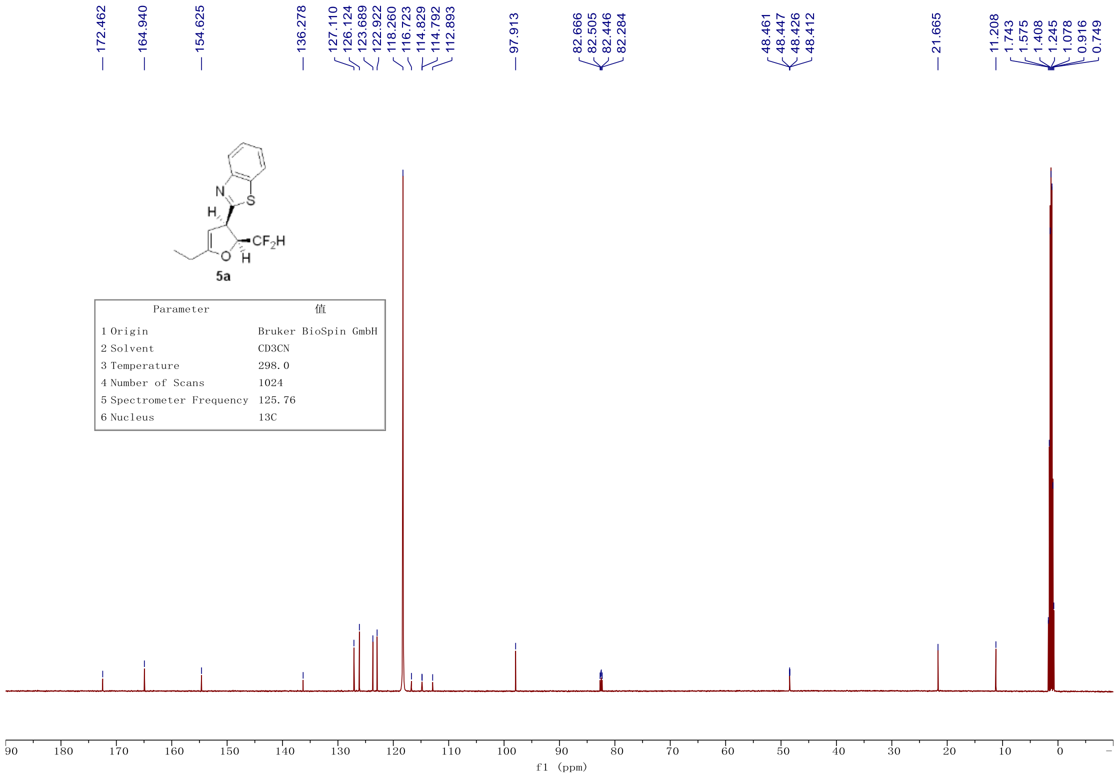

Figure S148. ^{13}C-NMR of **5a**

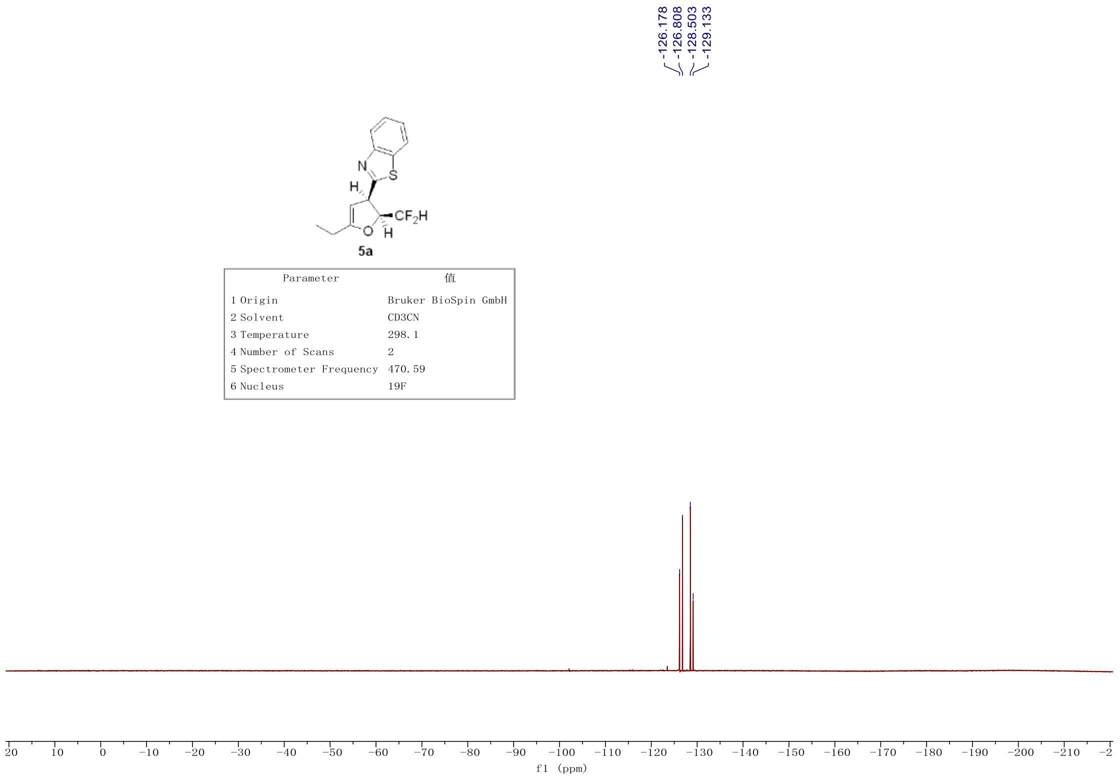

Figure S149. ^{19}F-NMR of **5a**

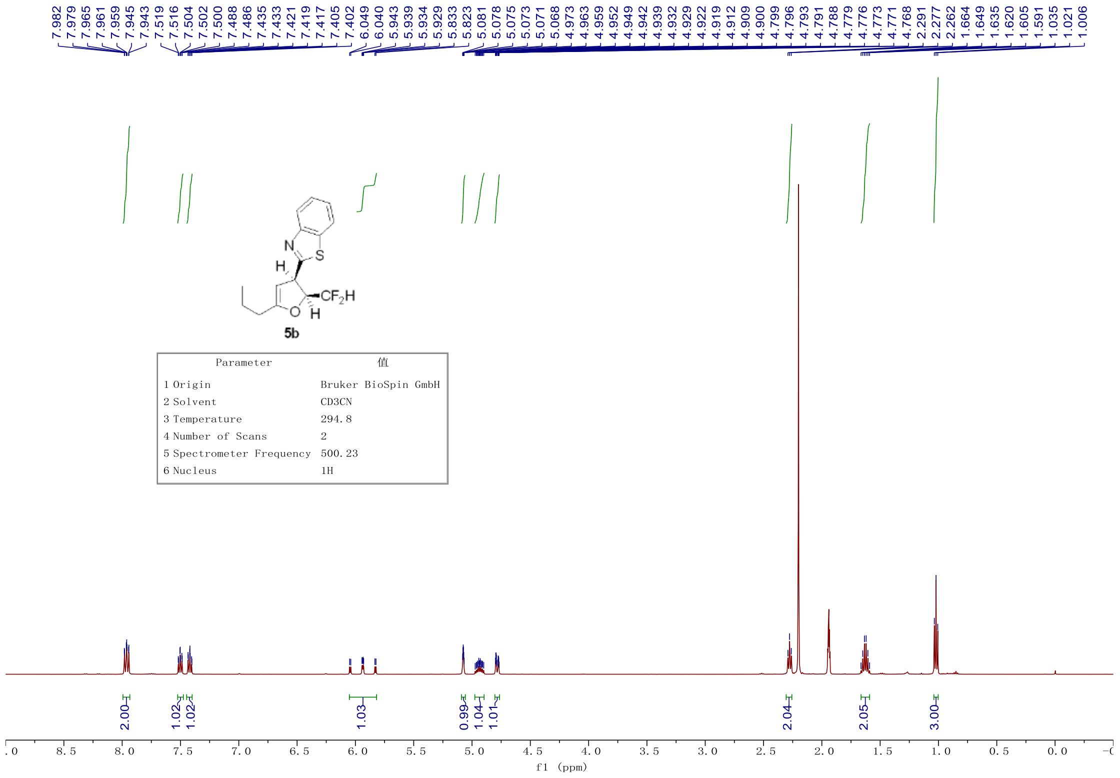

Figure S150. ^{1}H-NMR of **5b**

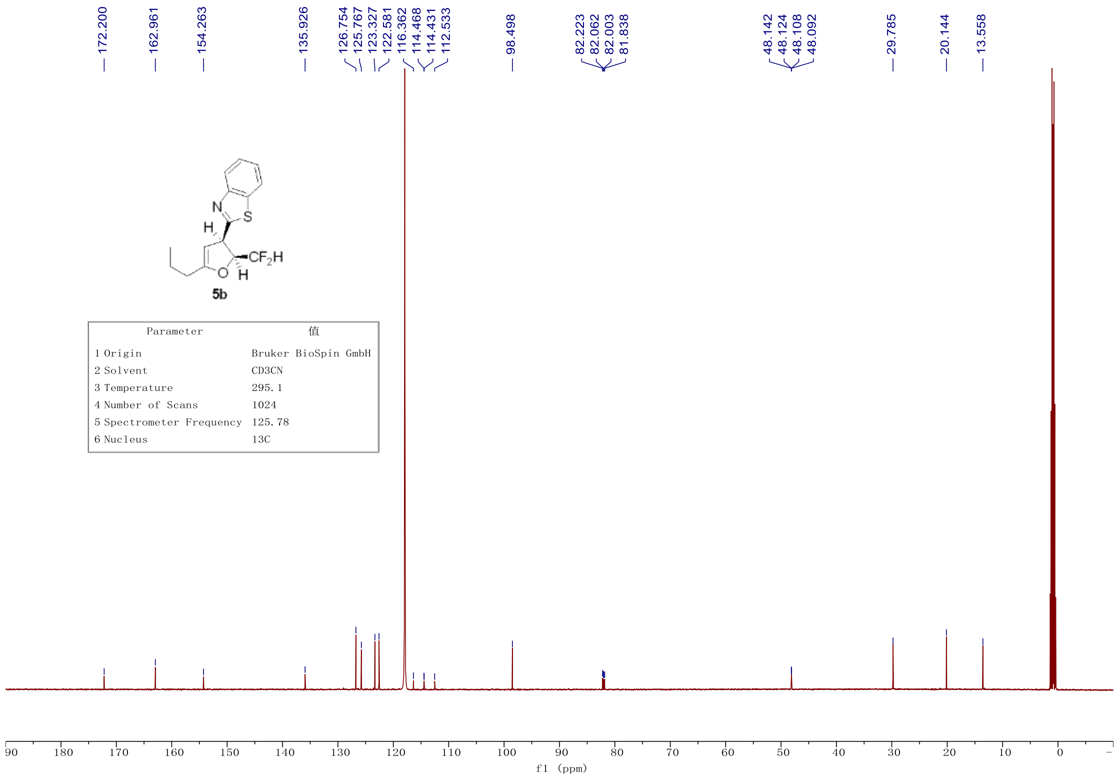

Figure S151. ^{13}C-NMR of **5b**

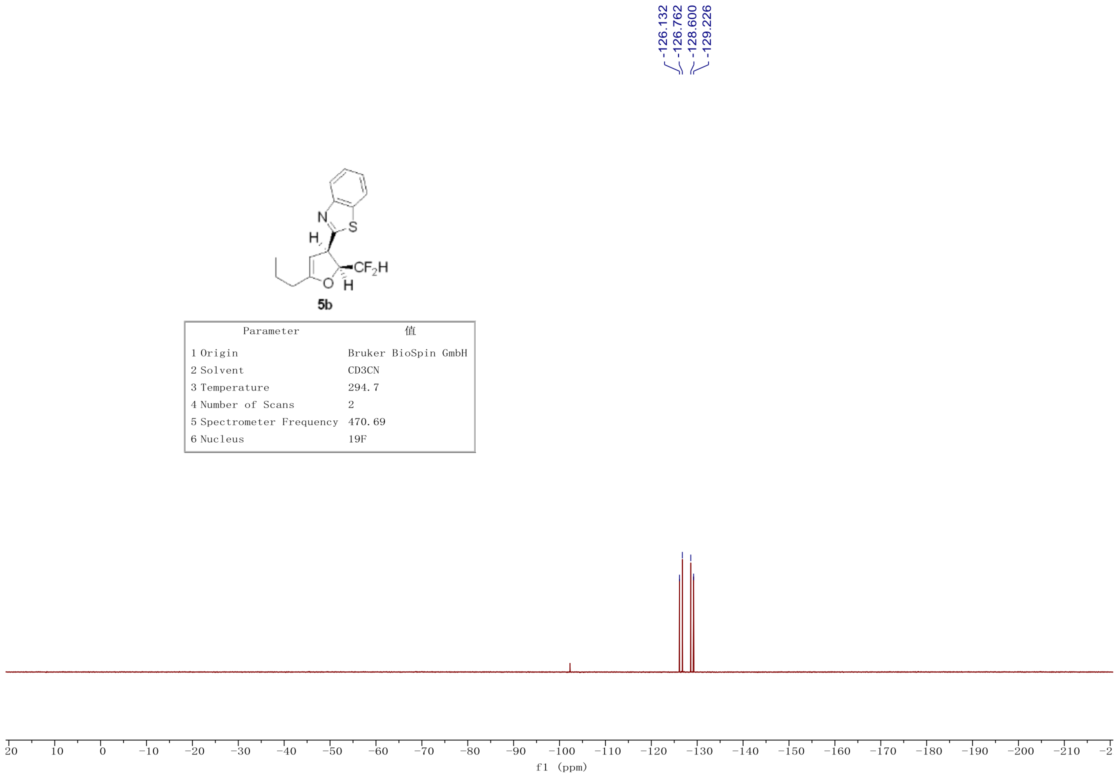

Figure S152. ^{19}F-NMR of **5b**

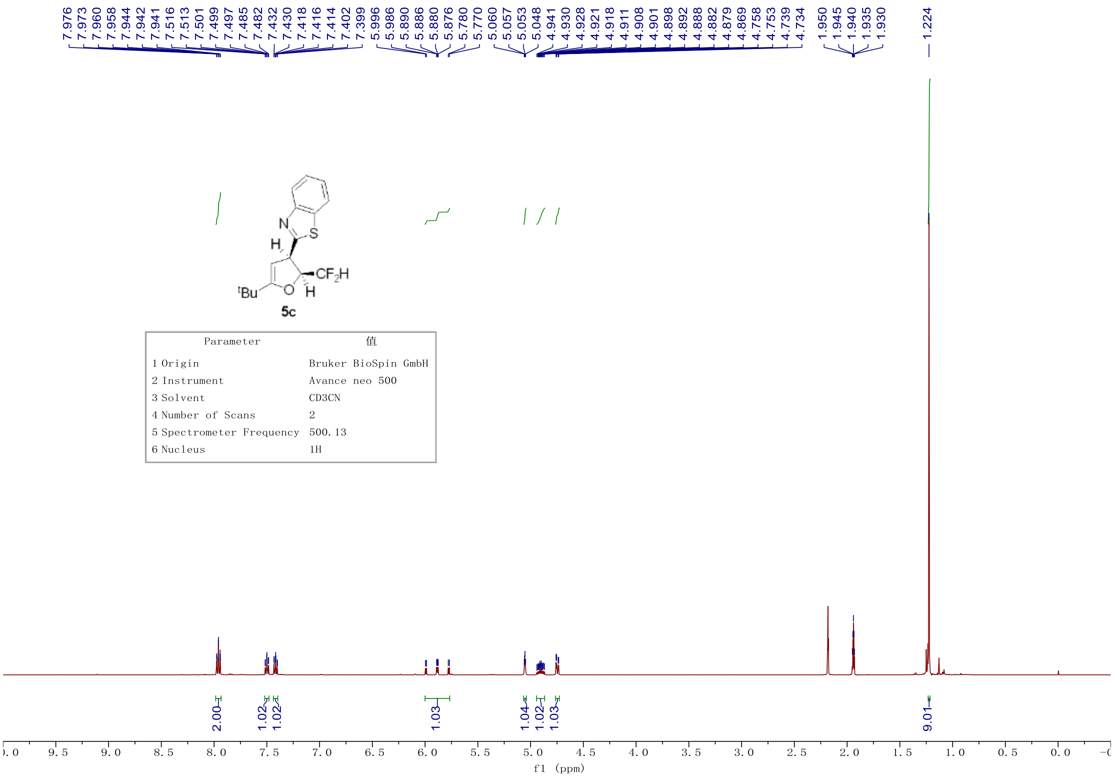

Figure S153. ^{1}H-NMR of **5c**

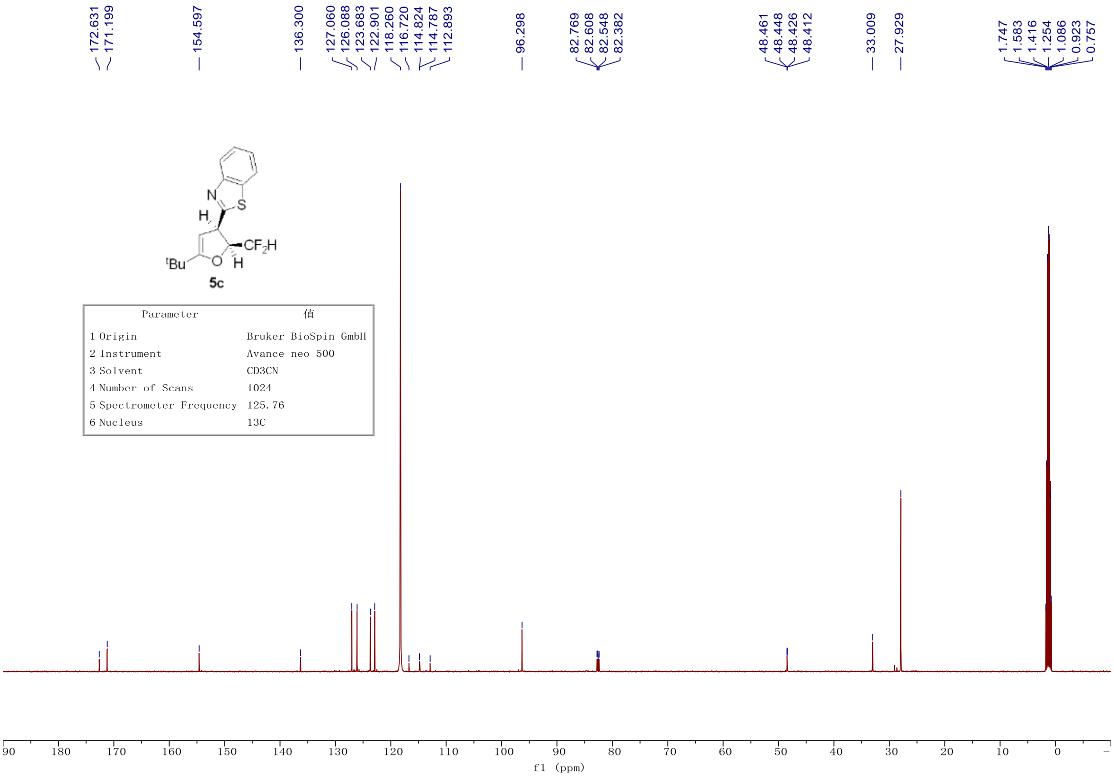

Figure S154. ^{13}C-NMR of **5c**

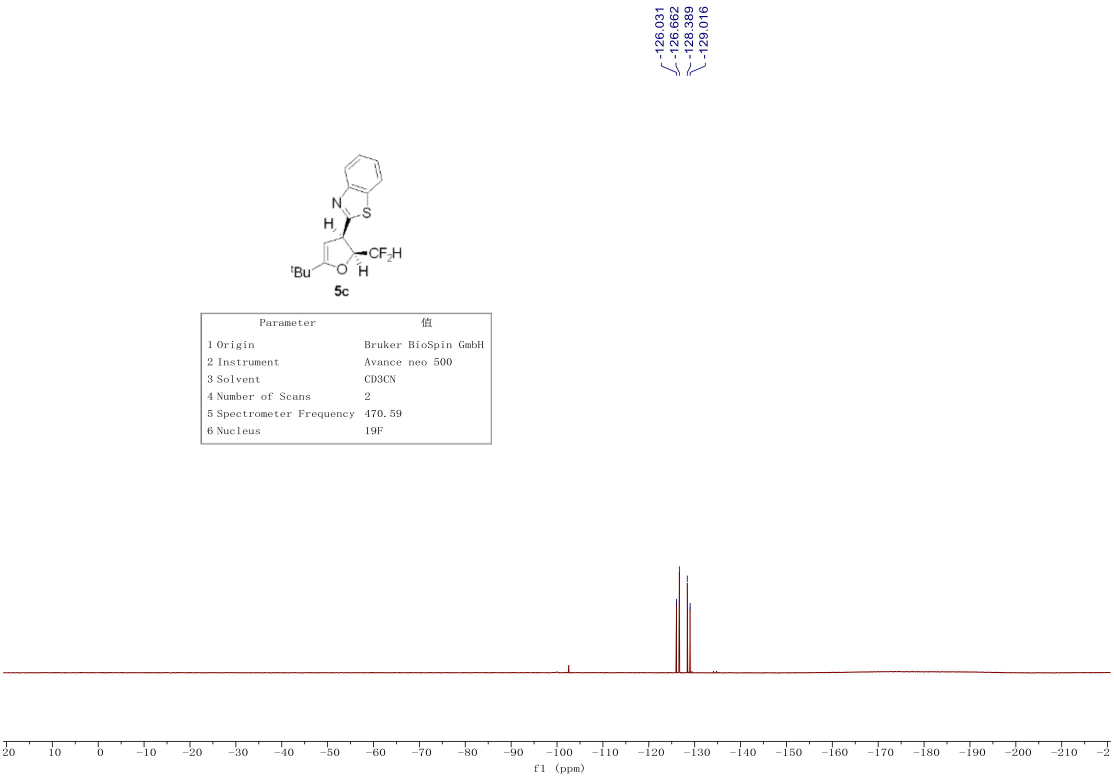

Figure S155. ^{19}F-NMR of **5c**

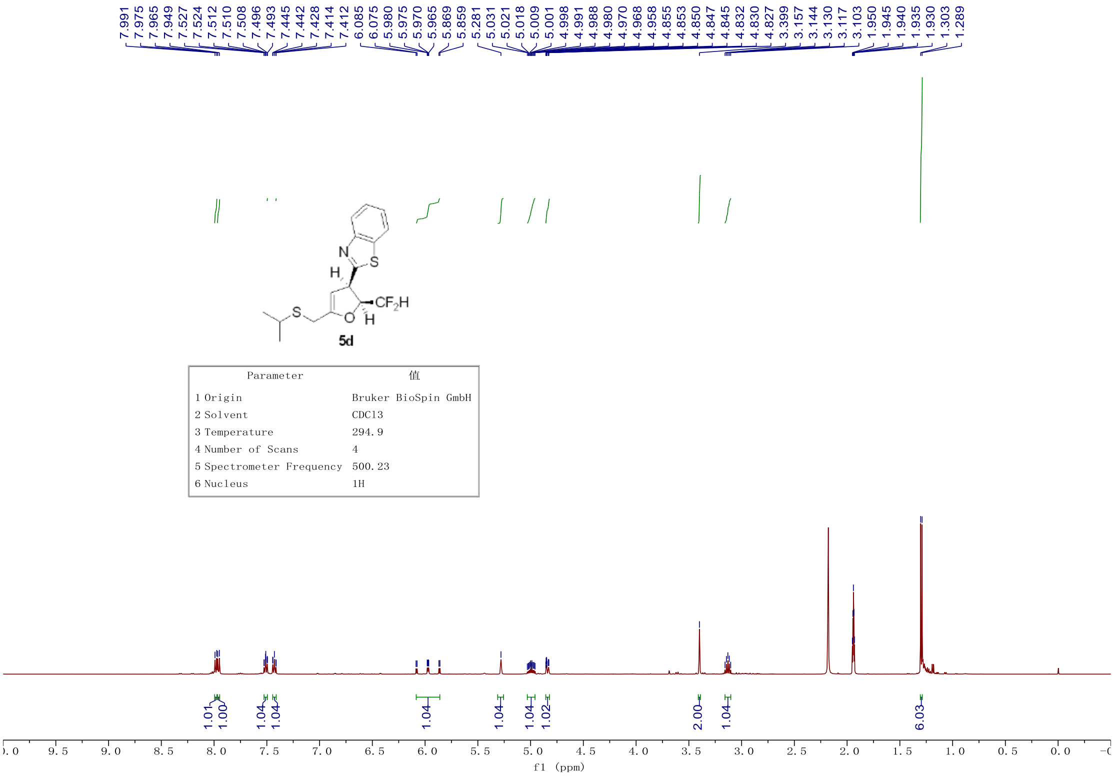

Figure S156. ^{1}H-NMR of **5d**

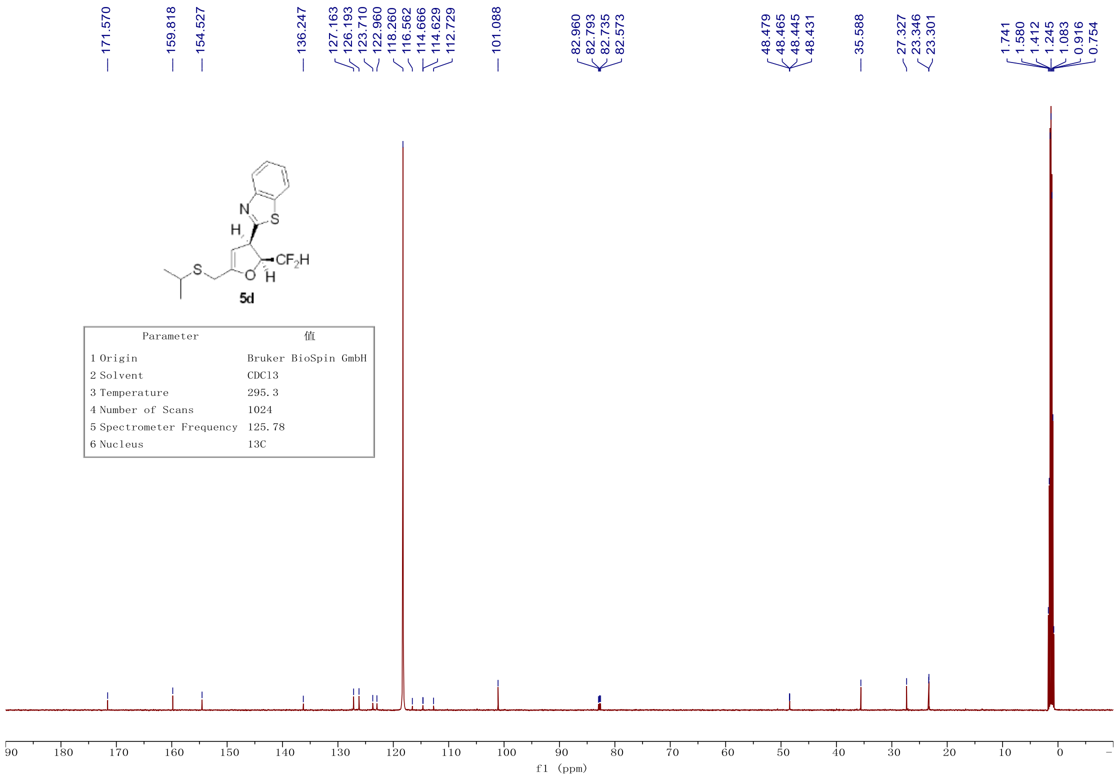

Parameter	值
1 Origin	Bruker BioSpin GmbH
2 Solvent	CDCl3
3 Temperature	295.3
4 Number of Scans	1024
5 Spectrometer Frequency	125.78
6 Nucleus	13C

Figure S157. ^{13}C-NMR of **5d**

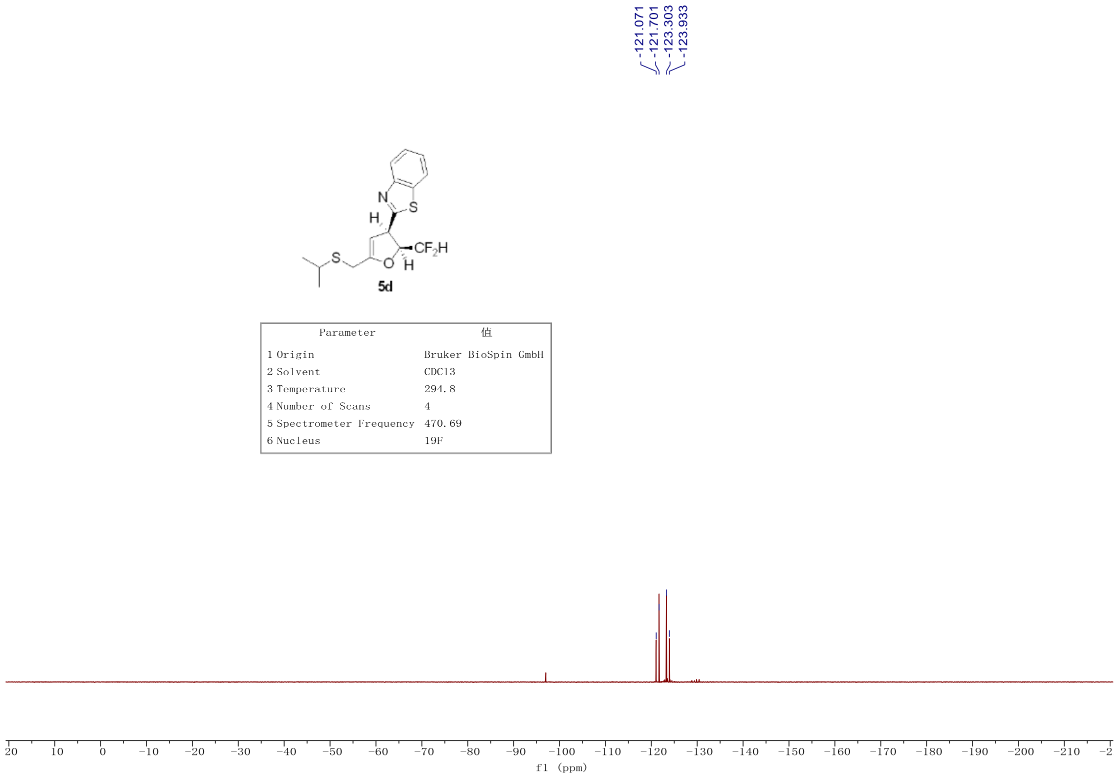

Parameter	值
1 Origin	Bruker BioSpin GmbH
2 Solvent	CDCl3
3 Temperature	294.8
4 Number of Scans	4
5 Spectrometer Frequency	470.69
6 Nucleus	19F

Figure S158. ^{19}F-NMR of **5d**

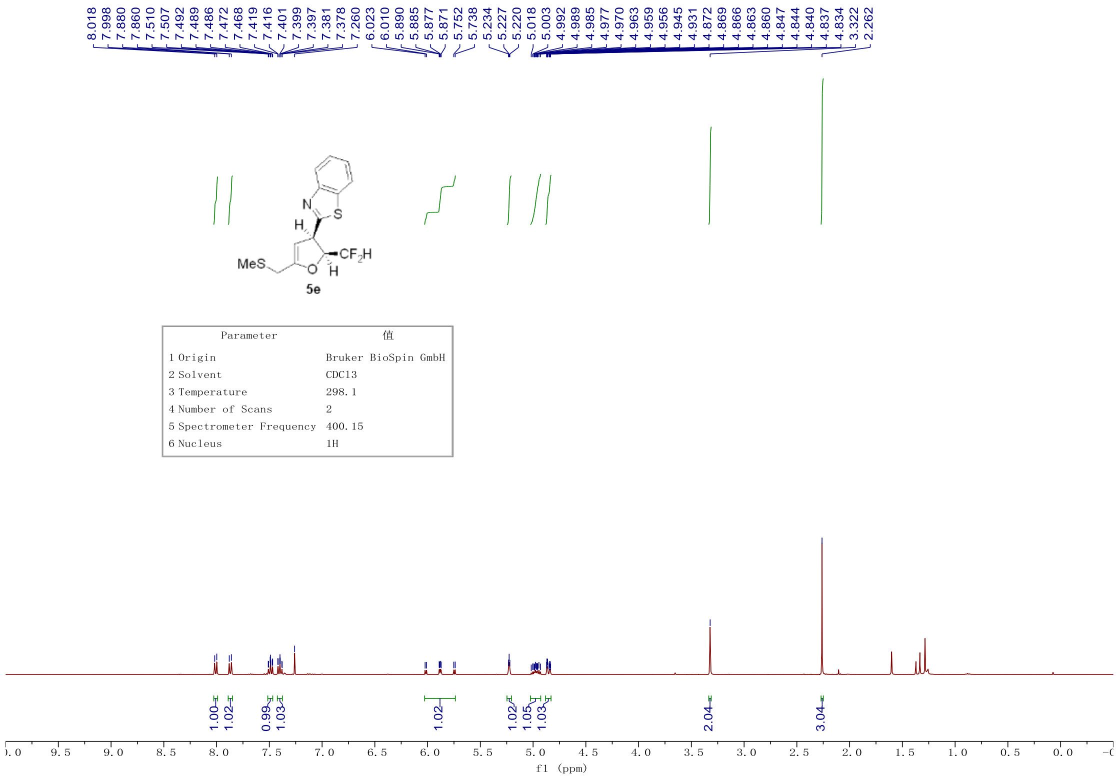

Figure S159. ^{1}H-NMR of **5e**

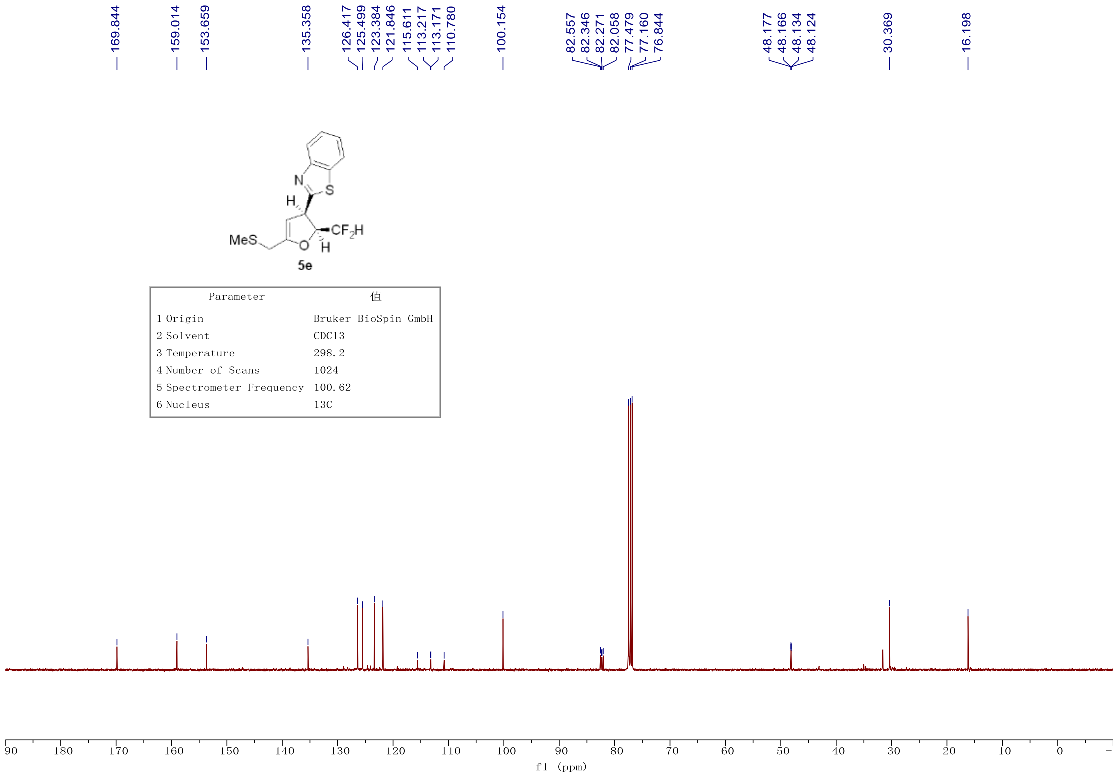

Figure S160. ^{13}C-NMR of **5e**

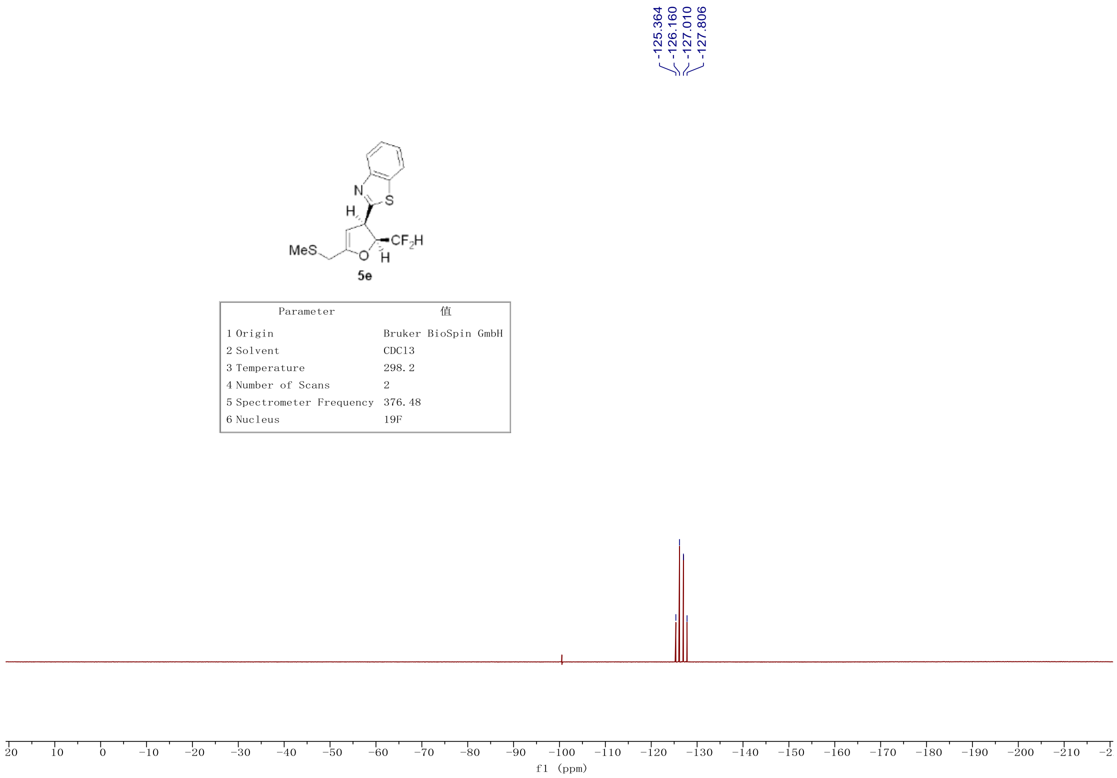

Figure S161. ^{19}F-NMR of **5e**

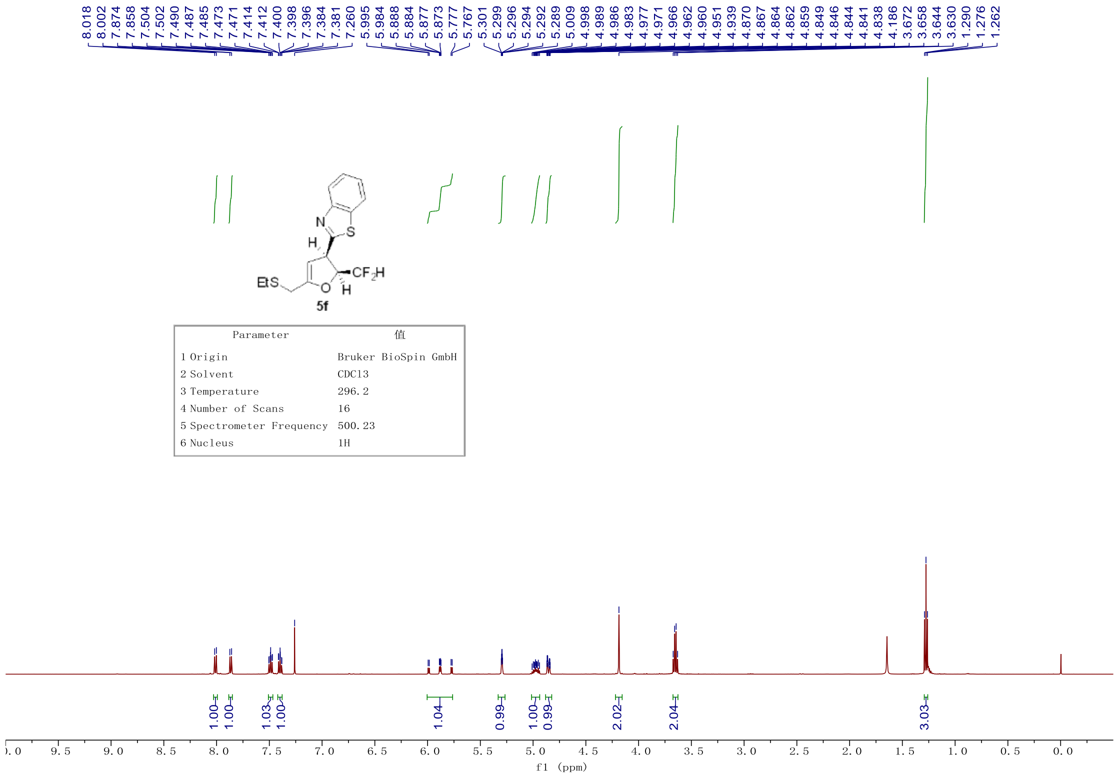

Figure S162. ^{1}H-NMR of **5f**

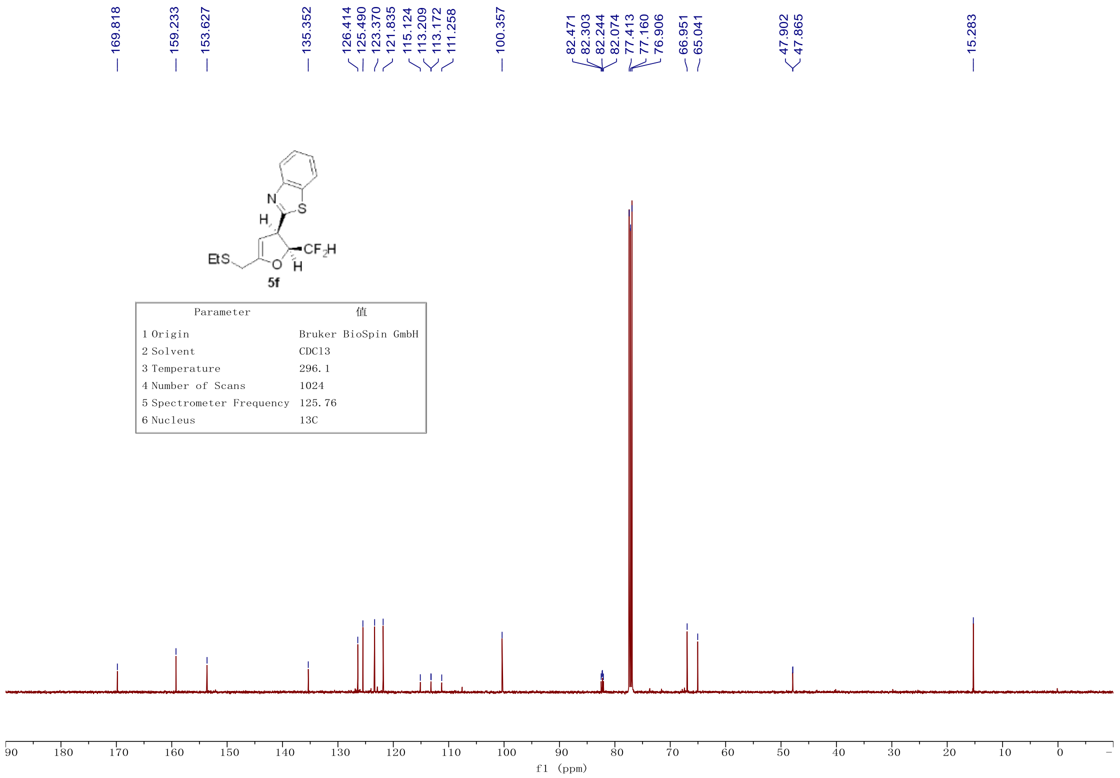

Figure S163. ^{13}C-NMR of **5f**

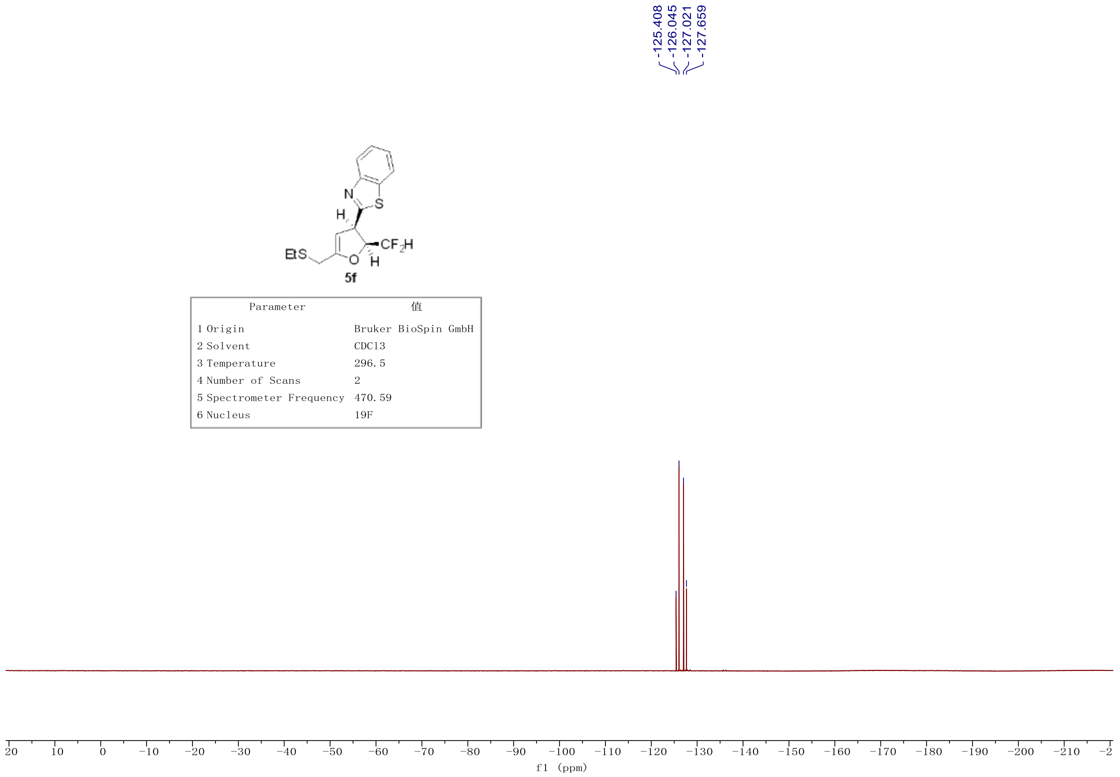

Figure S164. ^{19}F-NMR of **5f**

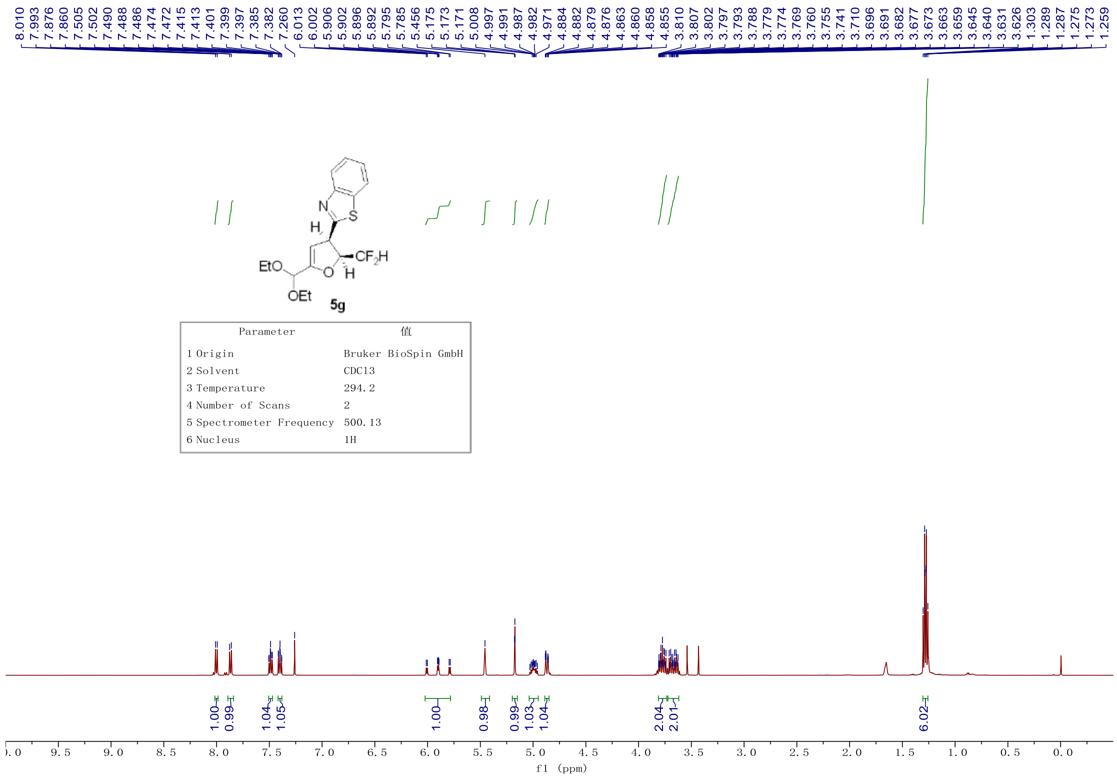

Figure S165. ^{1}H-NMR of **5g**

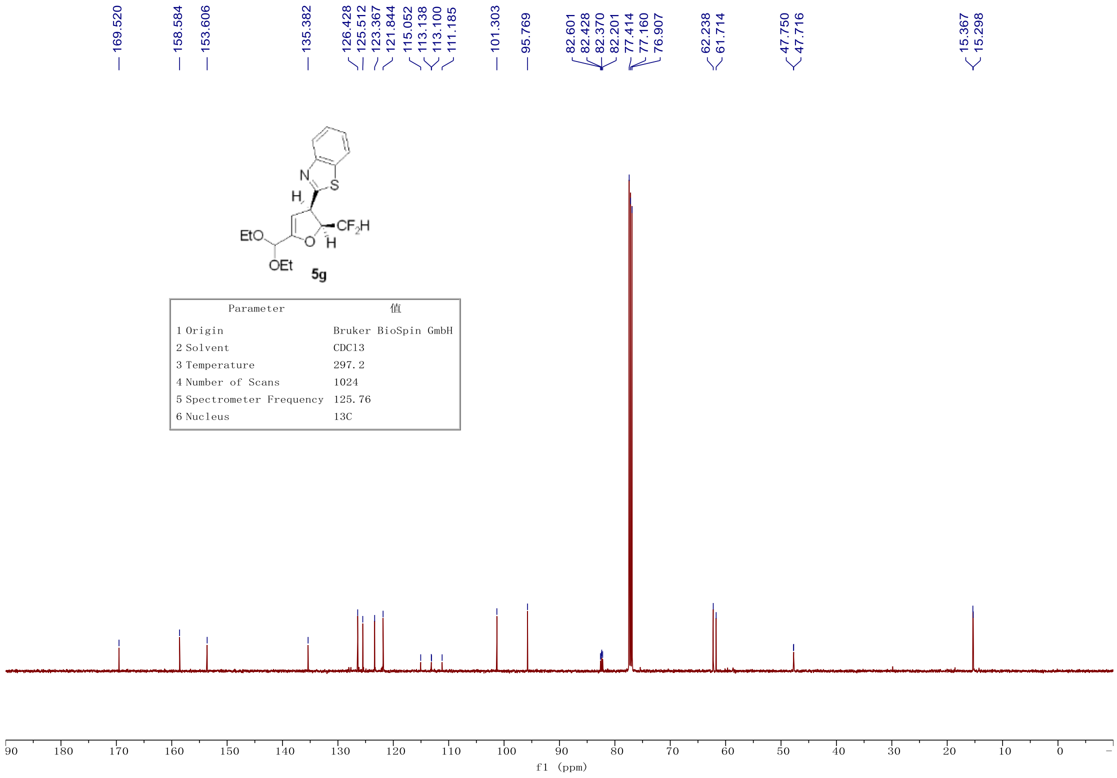

Figure S166. ^{13}C-NMR of **5g**

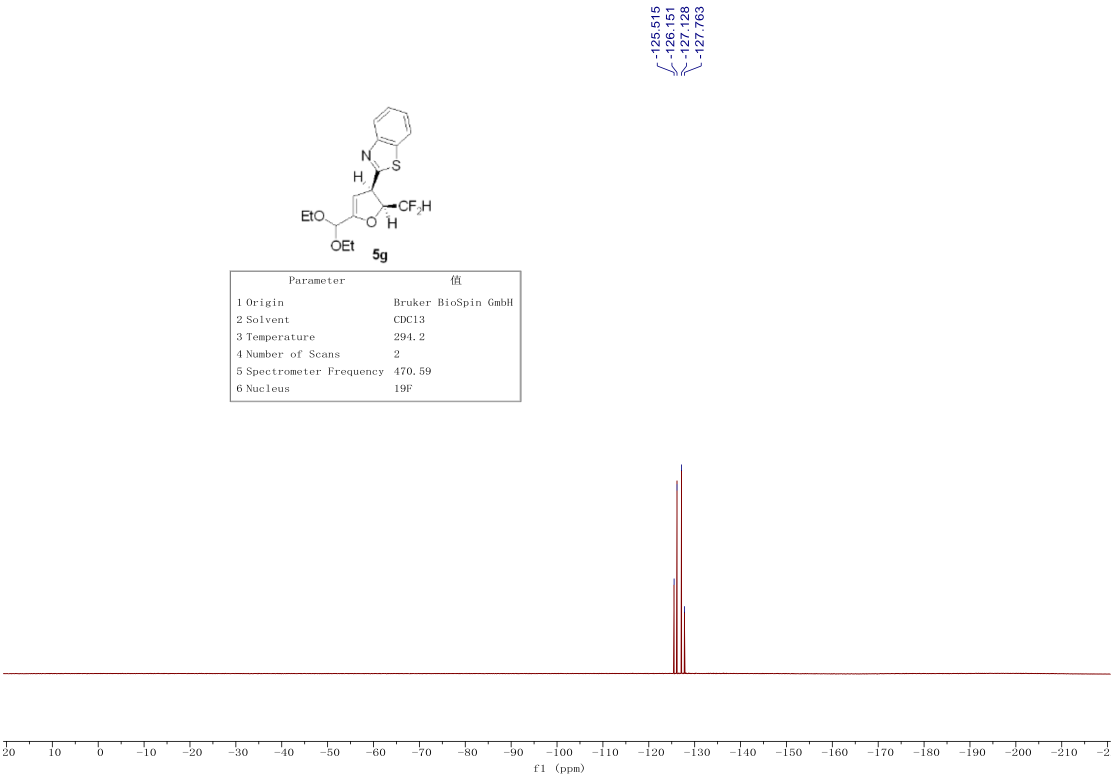

Figure S167. ^{19}F-NMR of **5g**

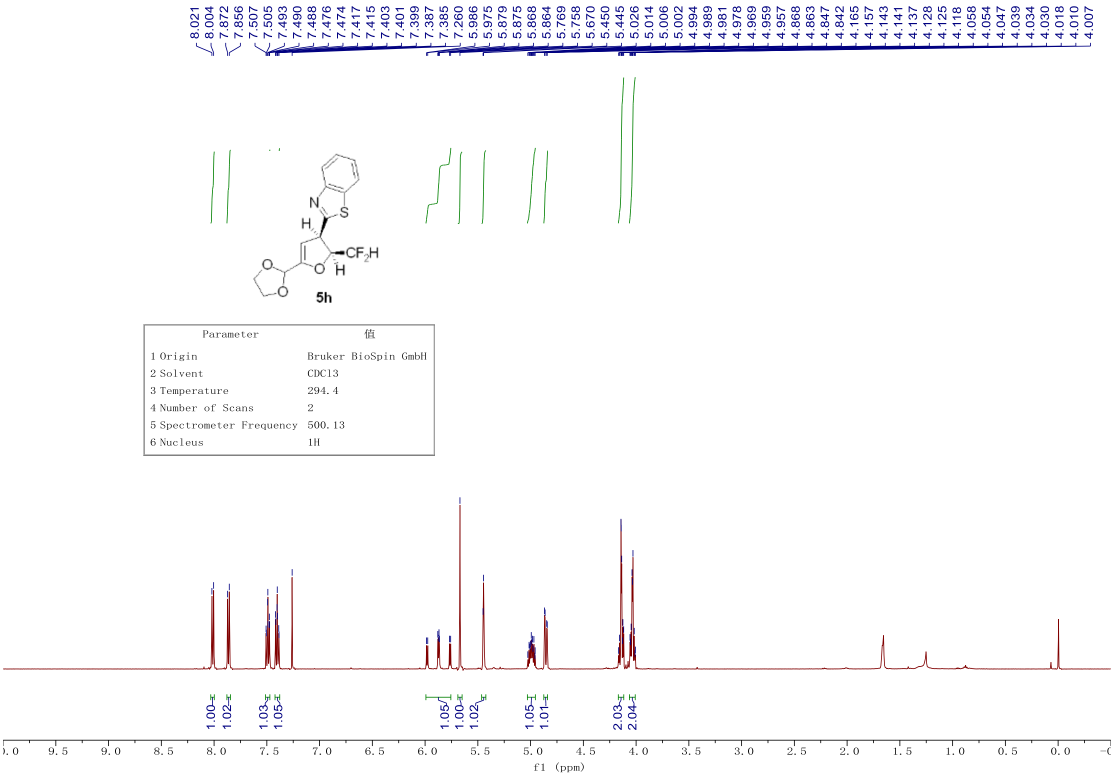

Figure S168. ^{1}H-NMR of **5h**

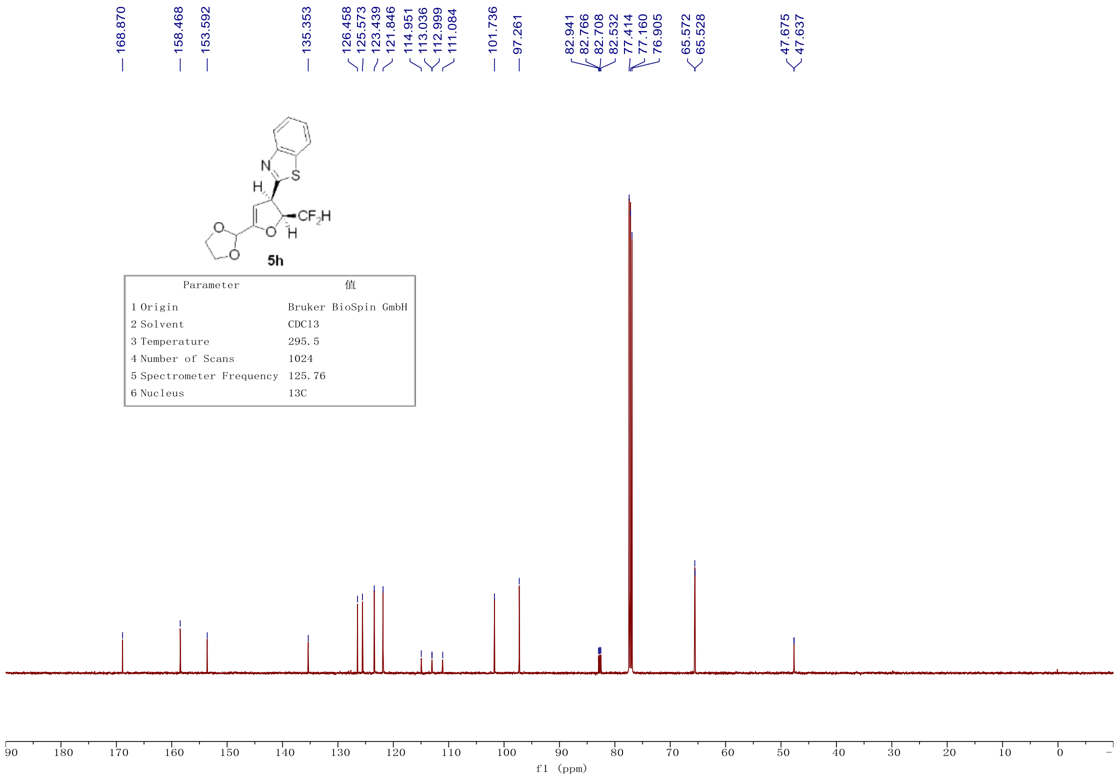

Figure S169. ^{13}C-NMR of **5h**

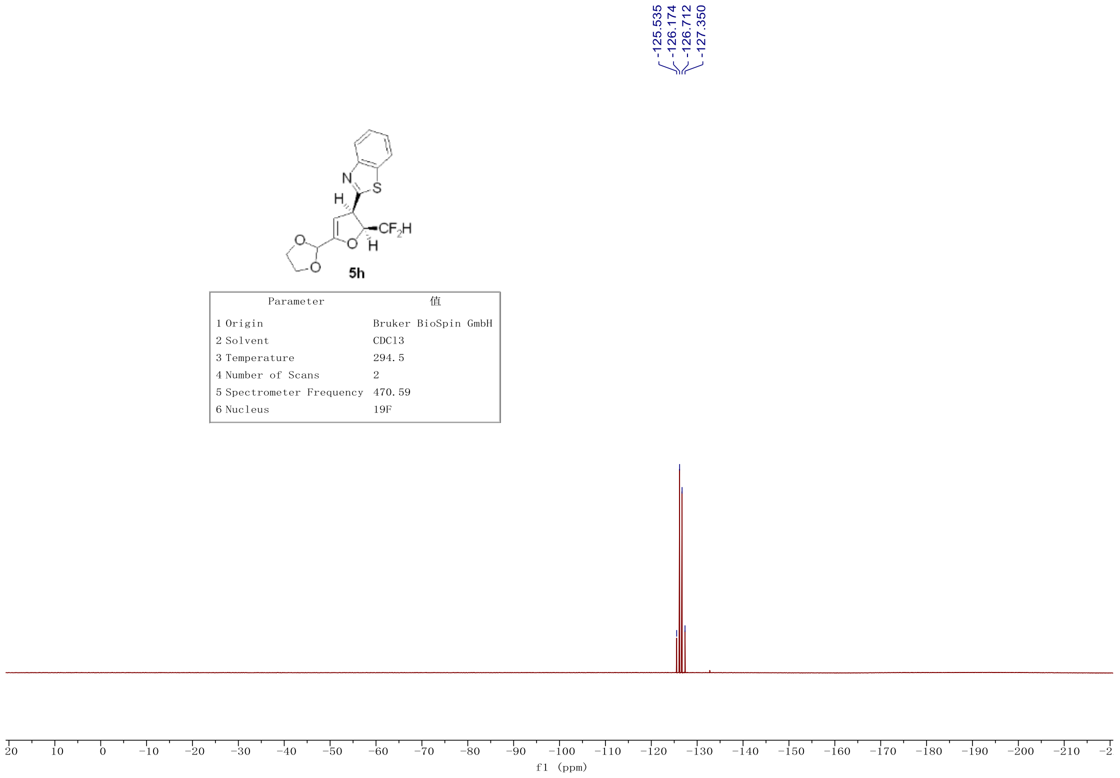

Figure S170. ^{19}F-NMR of **5h**

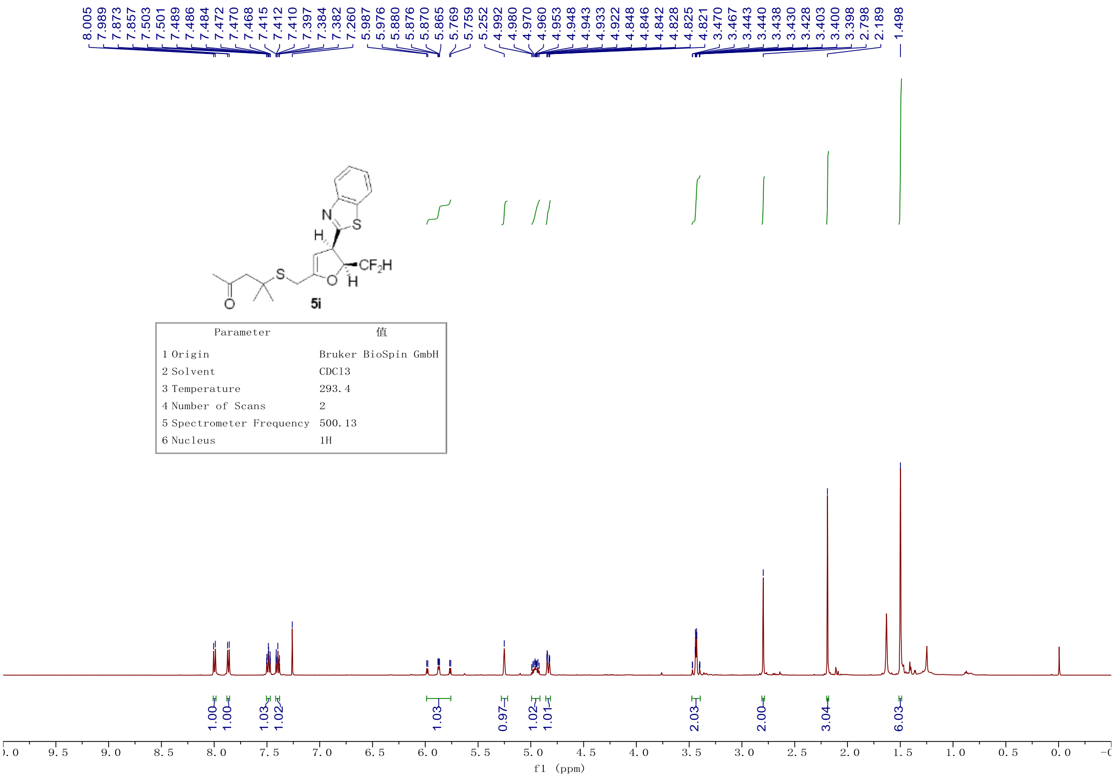

Figure S171. ^{1}H-NMR of **5i**

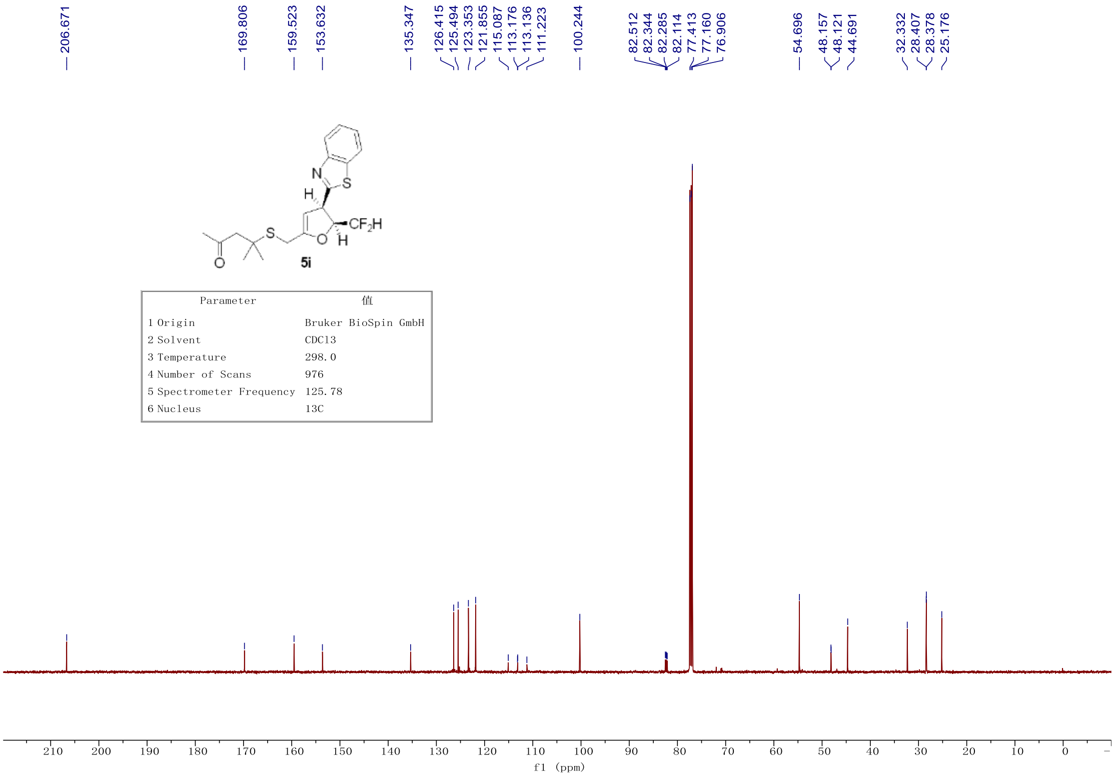

Figure S172. ^{13}C-NMR of **5i**

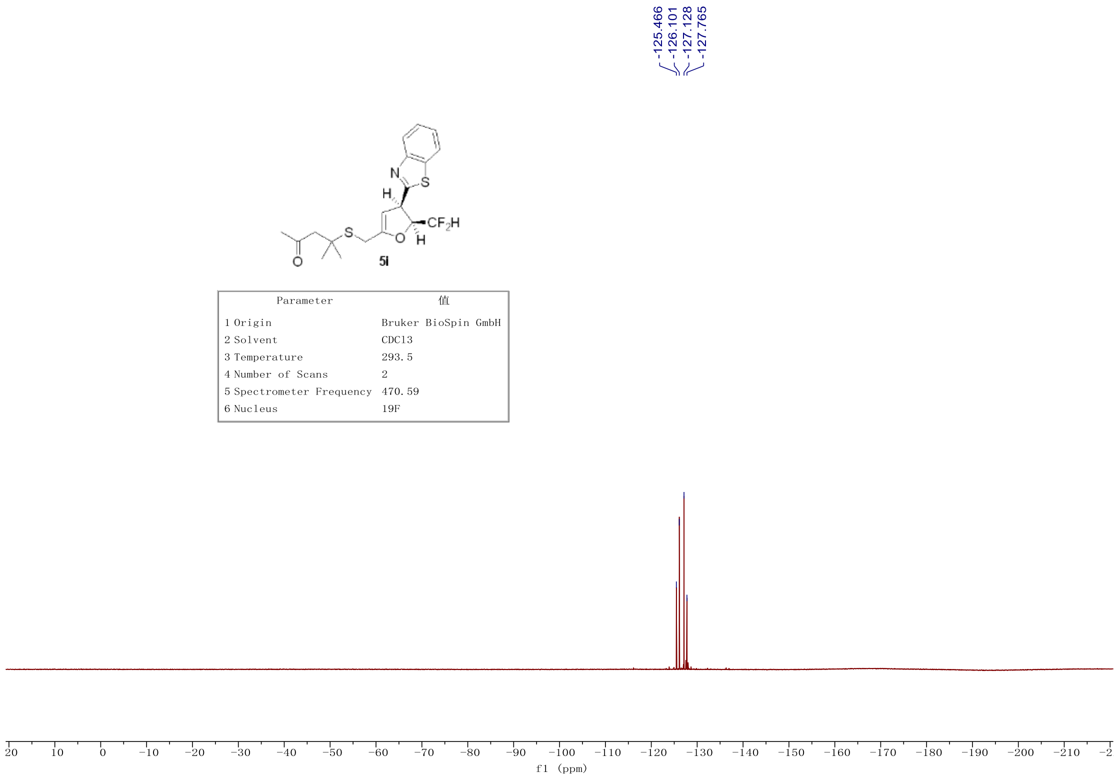

Figure S173. ^{19}F-NMR of **5i**

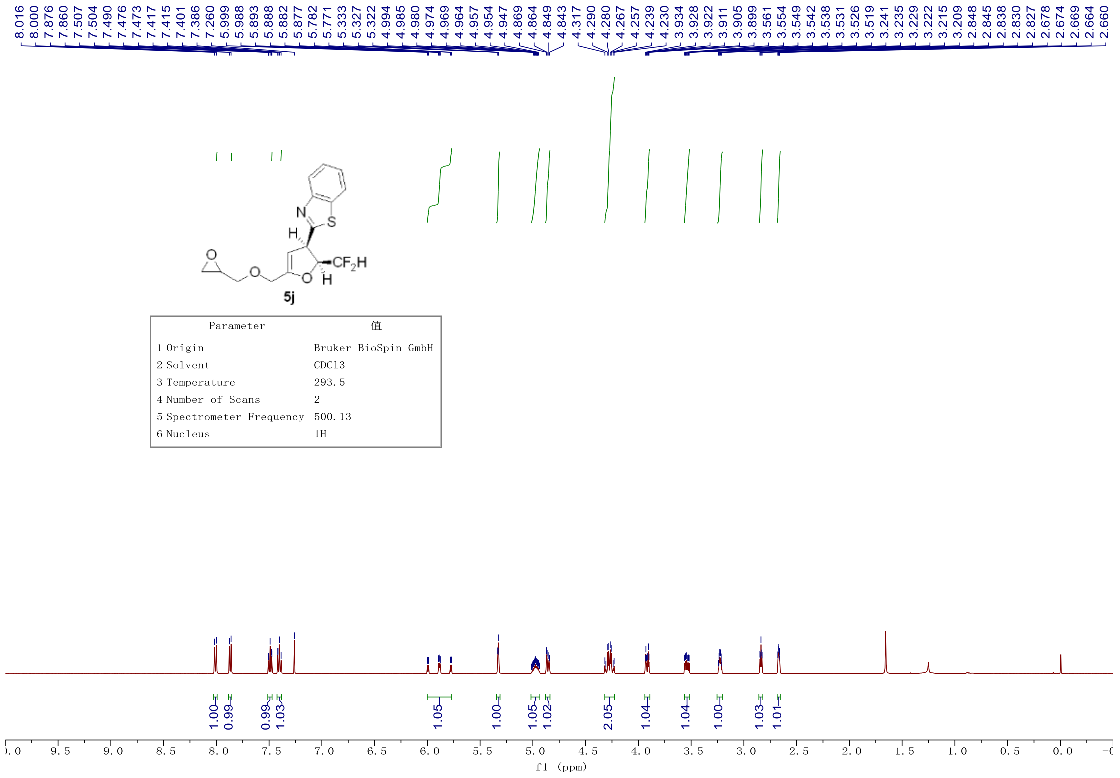

Figure S174. ^{1}H-NMR of **5j**

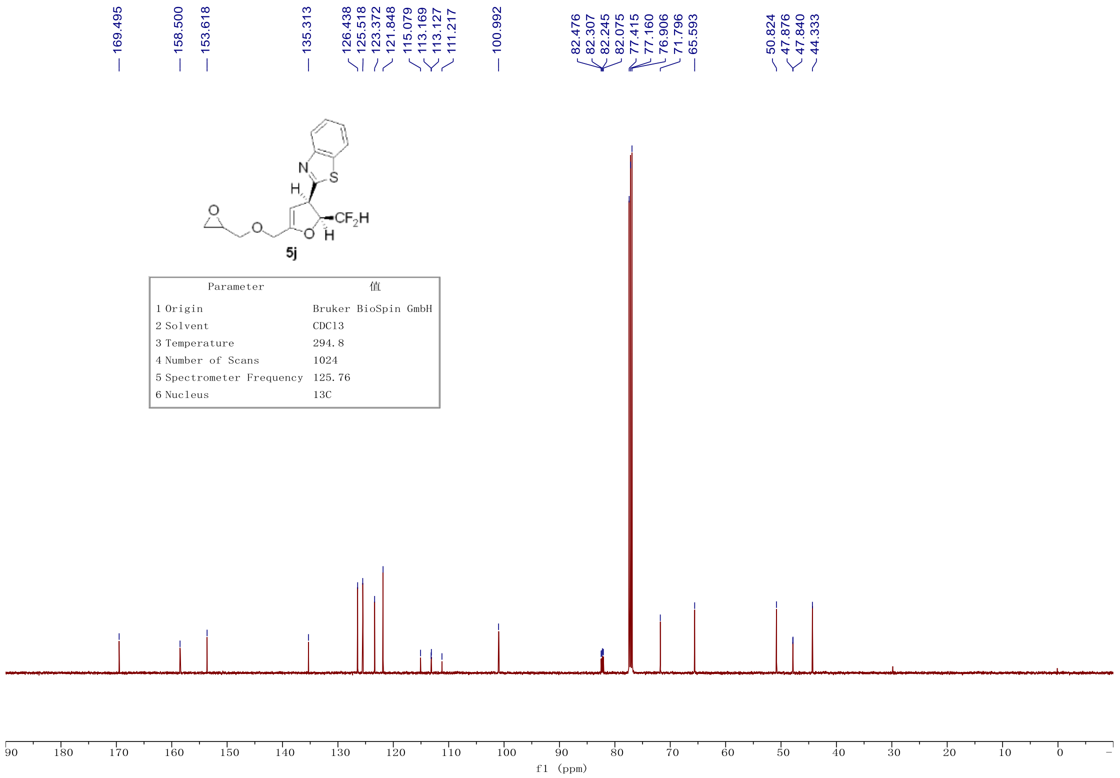

Figure S175. ^{13}C-NMR of **5j**

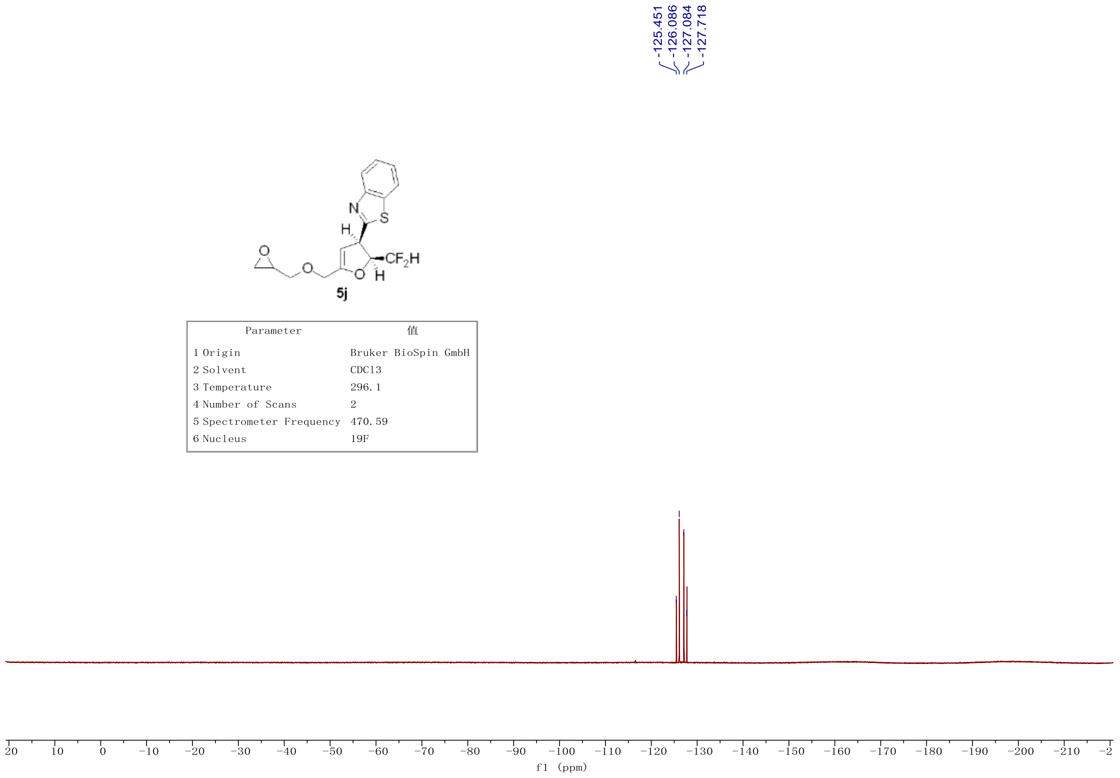

Figure S176. ^{19}F-NMR of **5j**

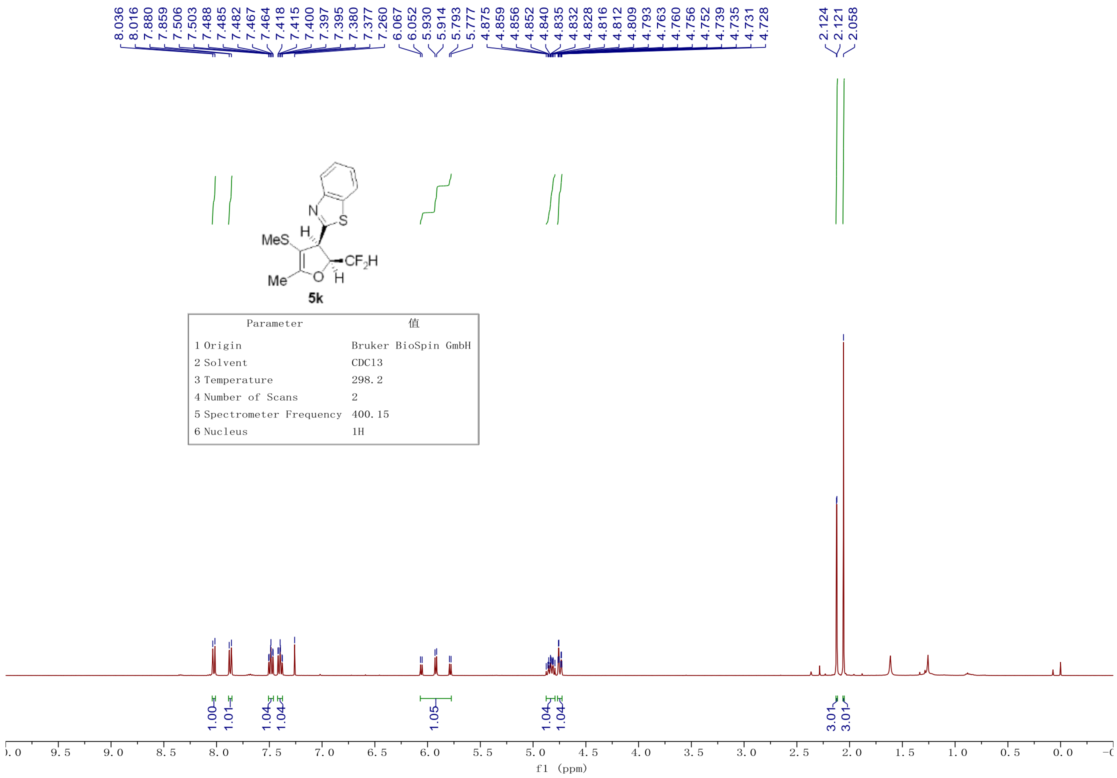

Figure S177. ^{1}H-NMR of **5k**

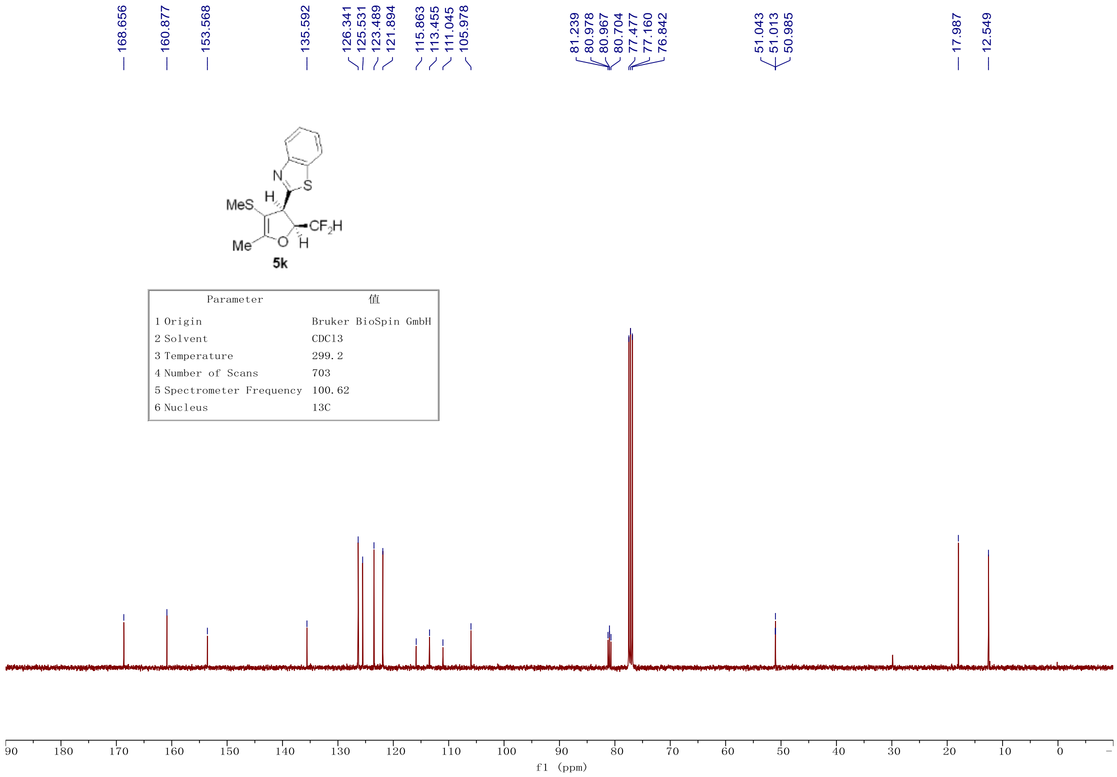

Figure S178. ^{13}C-NMR of **5k**

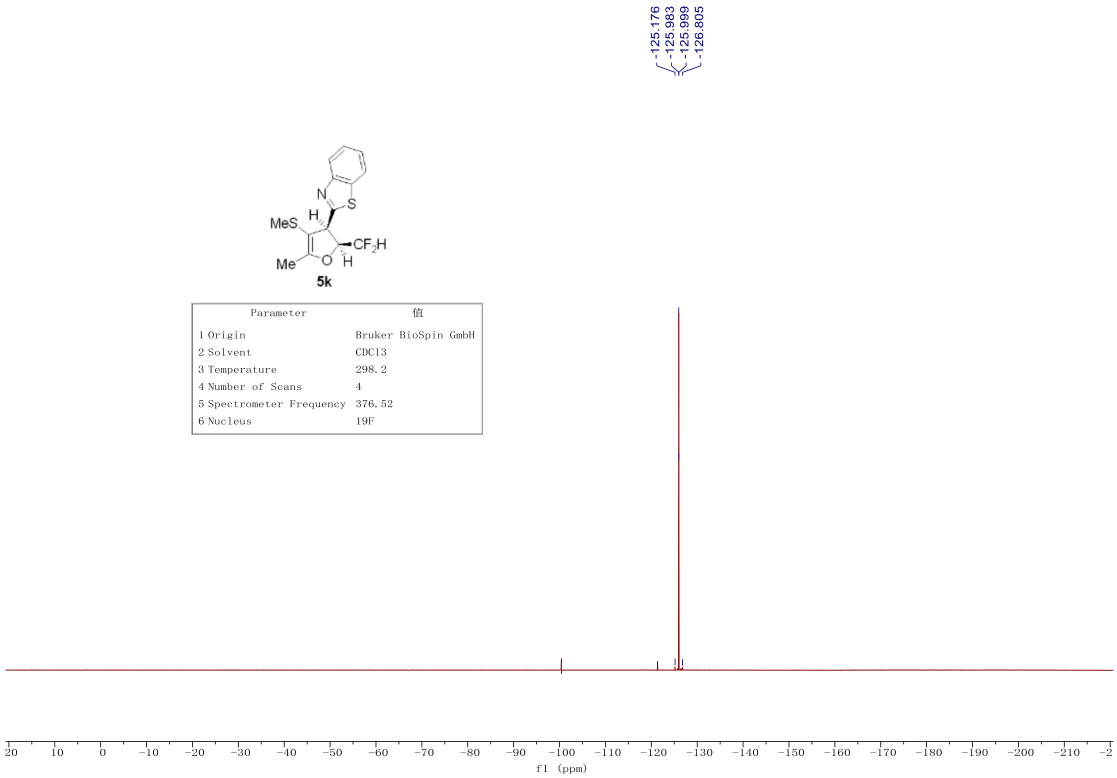

Figure S179. ^{19}F-NMR of **5k**

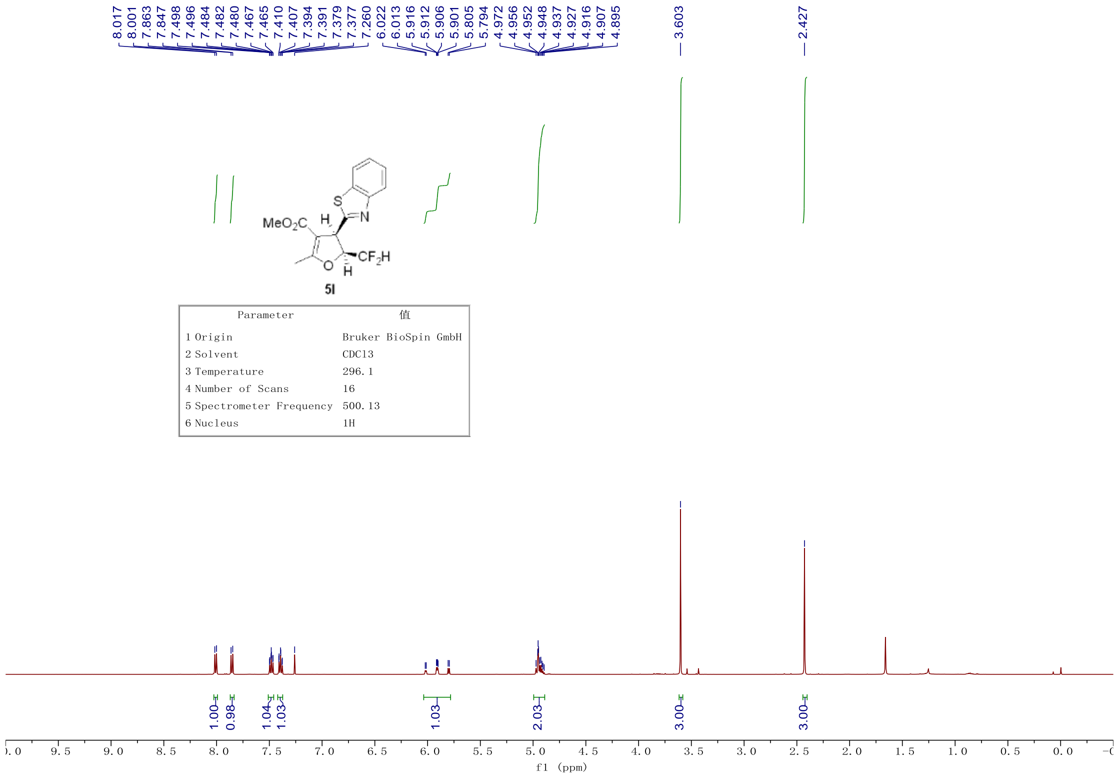

Figure S180. ^{1}H-NMR of **5l**

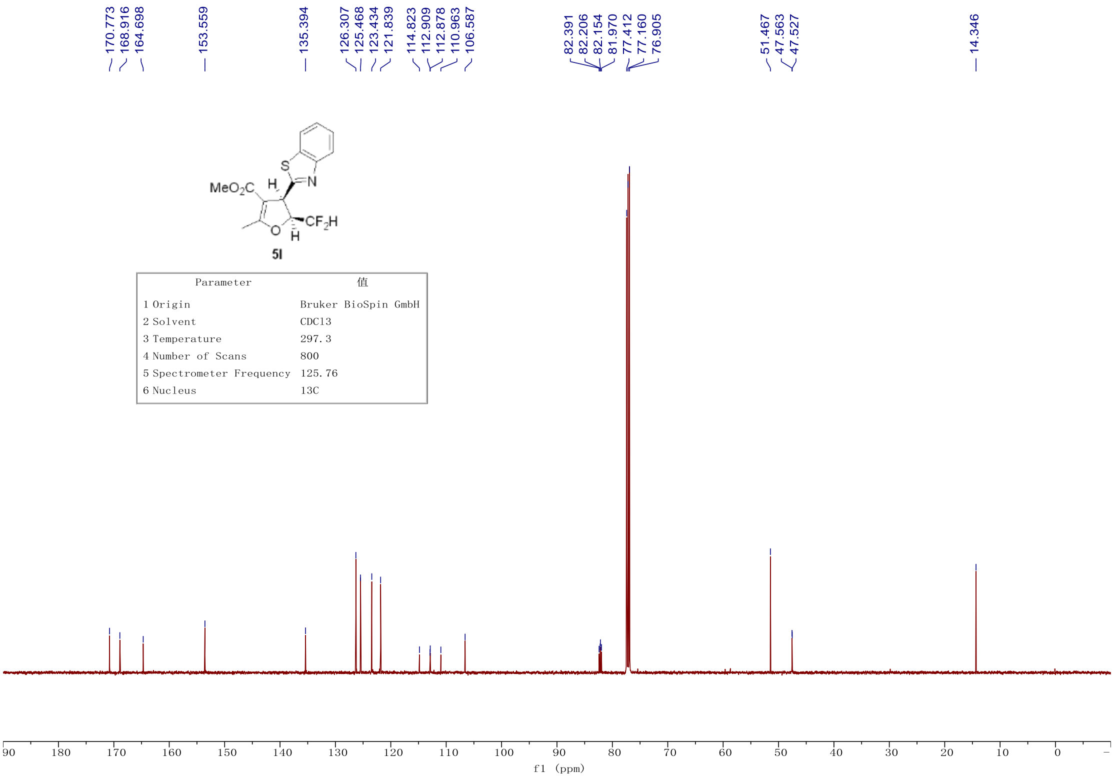

Parameter	值
1 Origin	Bruker BioSpin GmbH
2 Solvent	CDCl3
3 Temperature	297.3
4 Number of Scans	800
5 Spectrometer Frequency	125.76
6 Nucleus	13C

Figure S181. ^{13}C-NMR of **5l**

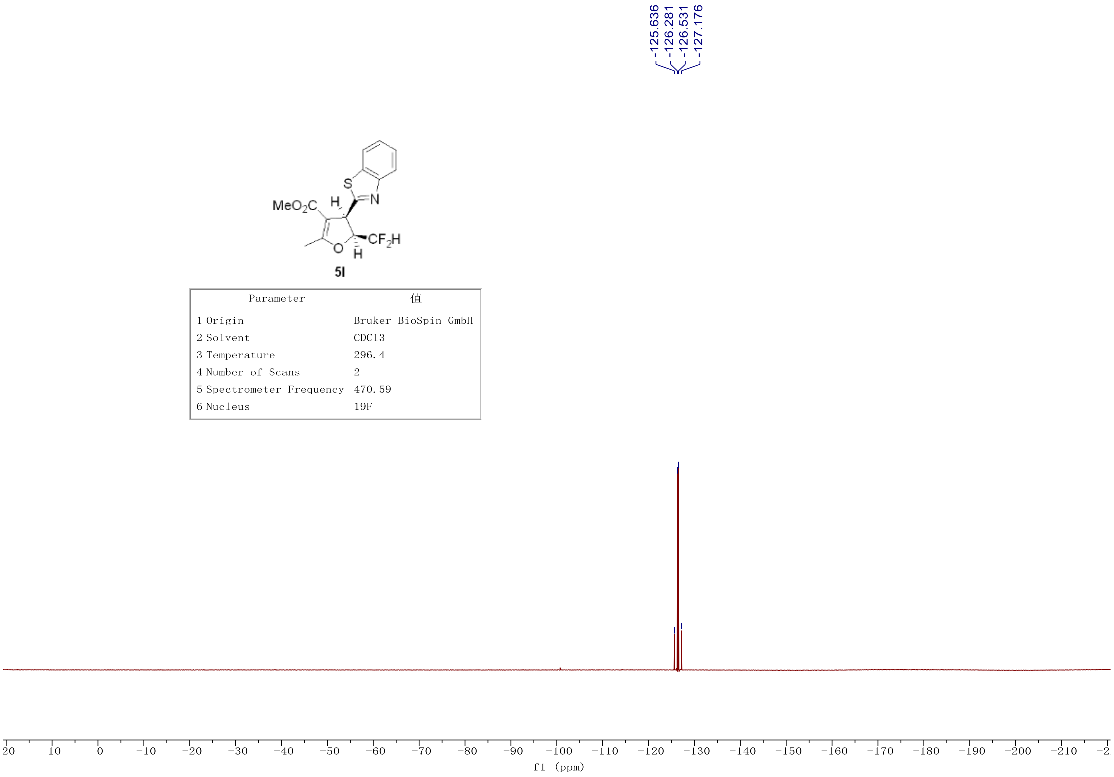

Parameter	值
1 Origin	Bruker BioSpin GmbH
2 Solvent	CDCl3
3 Temperature	296.4
4 Number of Scans	2
5 Spectrometer Frequency	470.59
6 Nucleus	19F

Figure S182. ^{19}F-NMR of **5l**

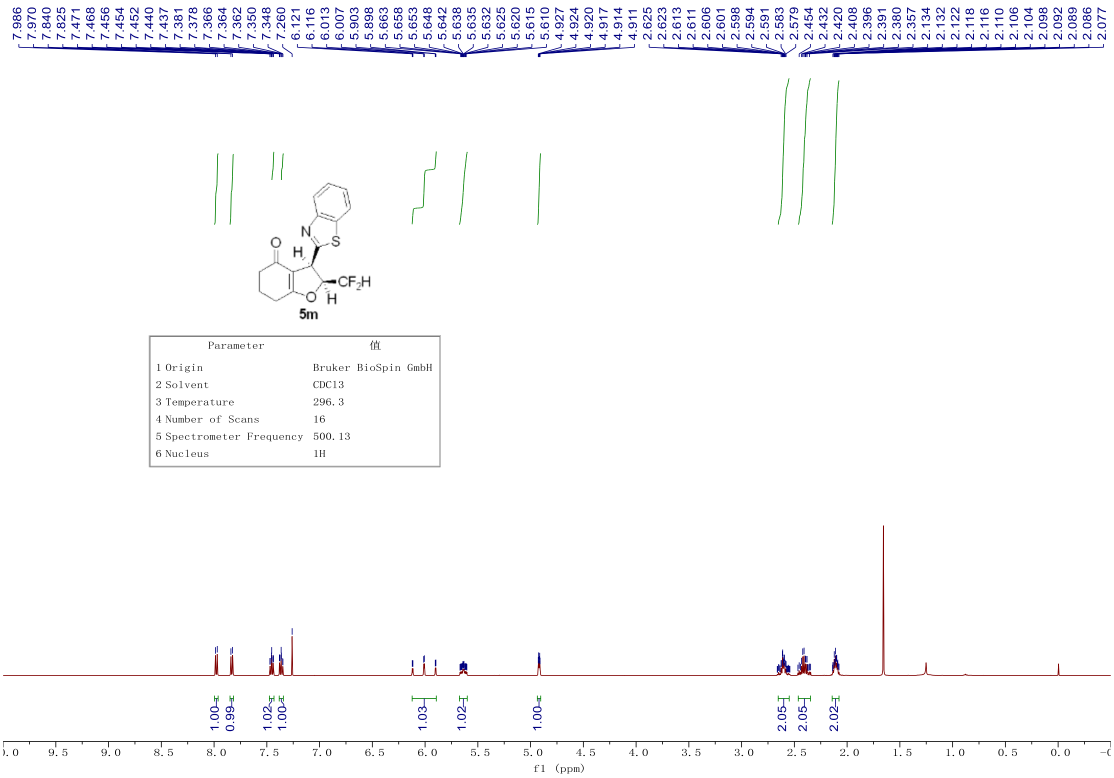

Figure S183. ^{1}H-NMR of **5m**

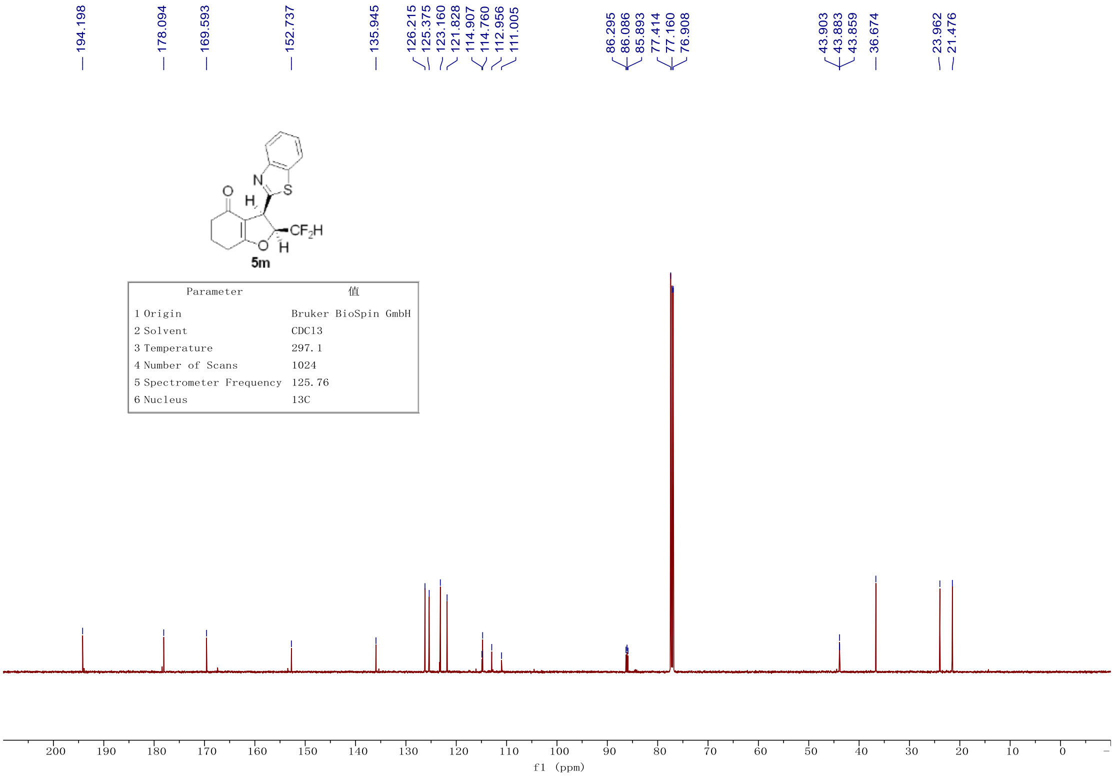

Figure S184. ^{13}C-NMR of **5m**

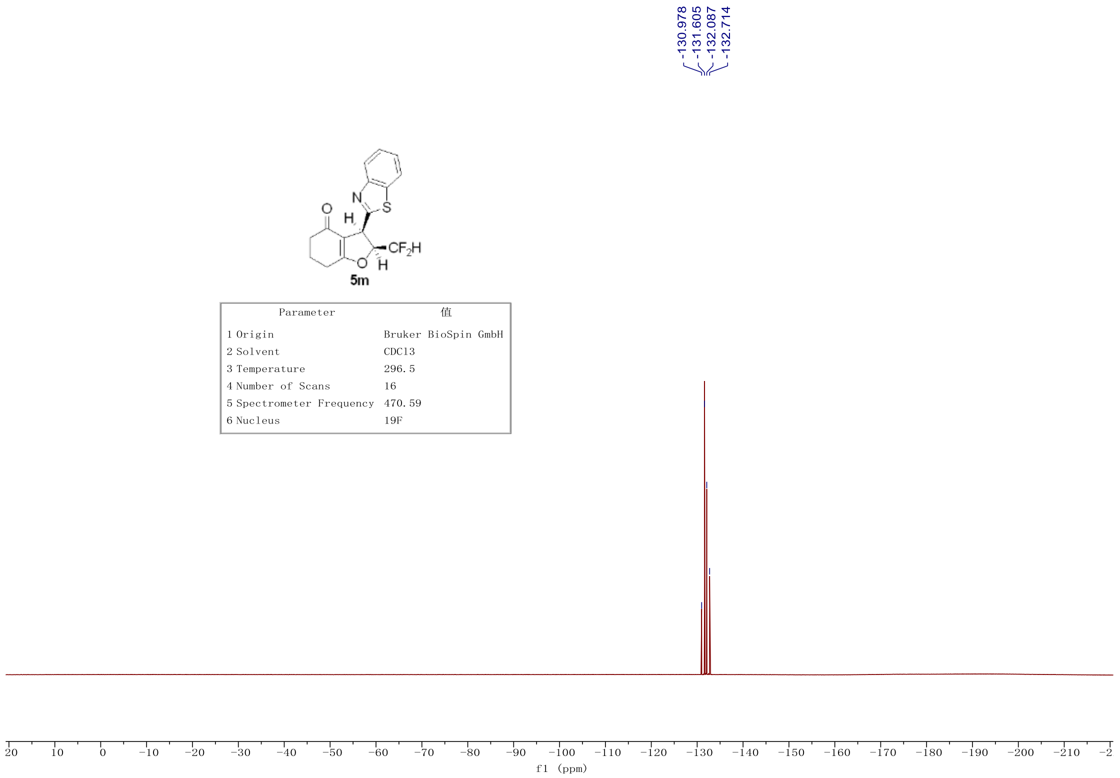

Figure S185. ^{19}F-NMR of **5m**

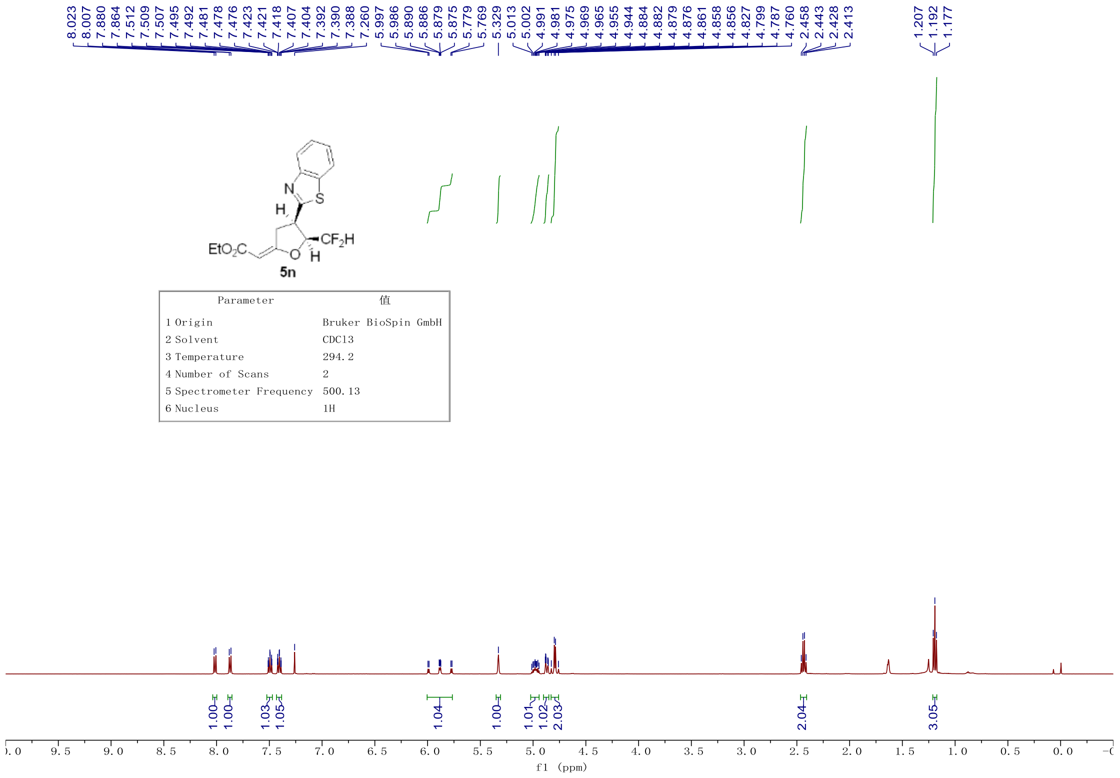

Figure S186. ^{1}H-NMR of **5n**

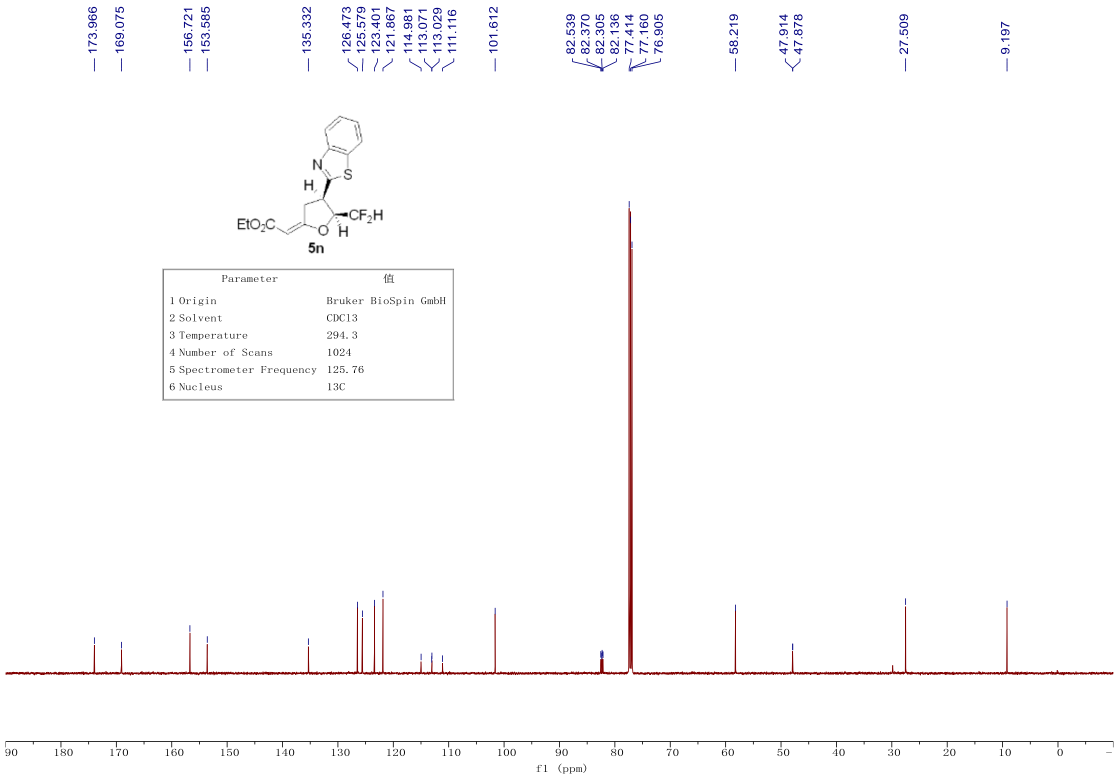

Figure S187. ^{13}C-NMR of **5n**

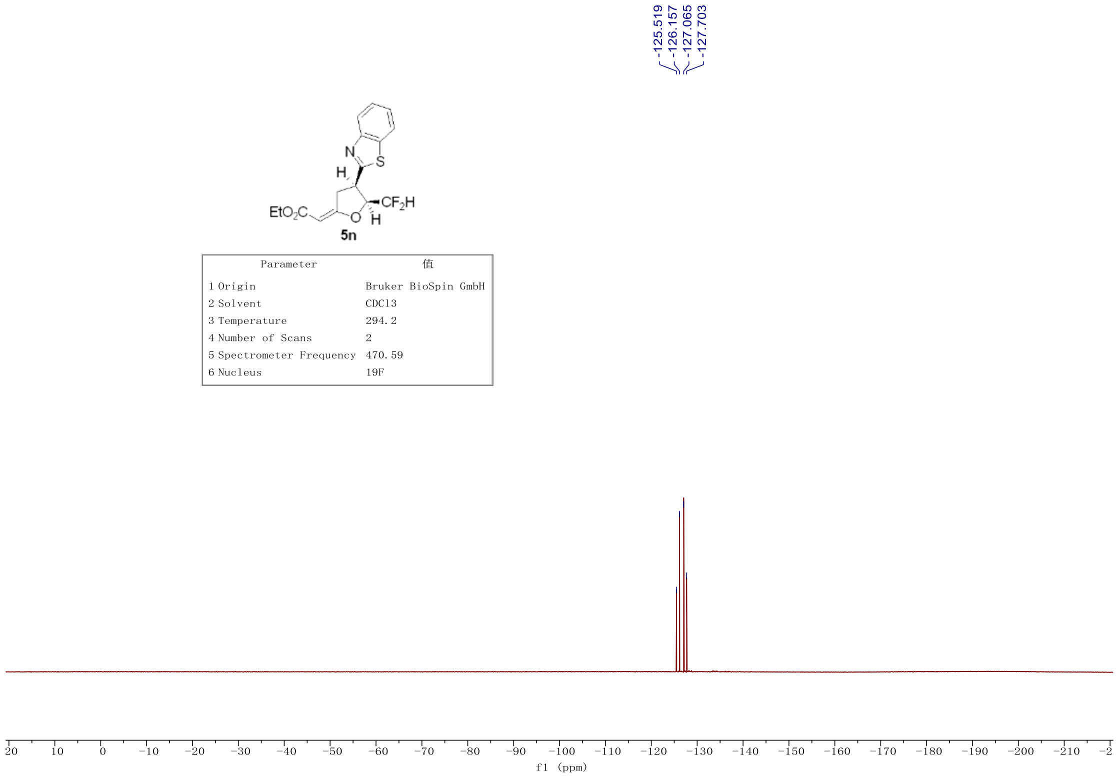

Figure S188. ^{19}F-NMR of **5n**

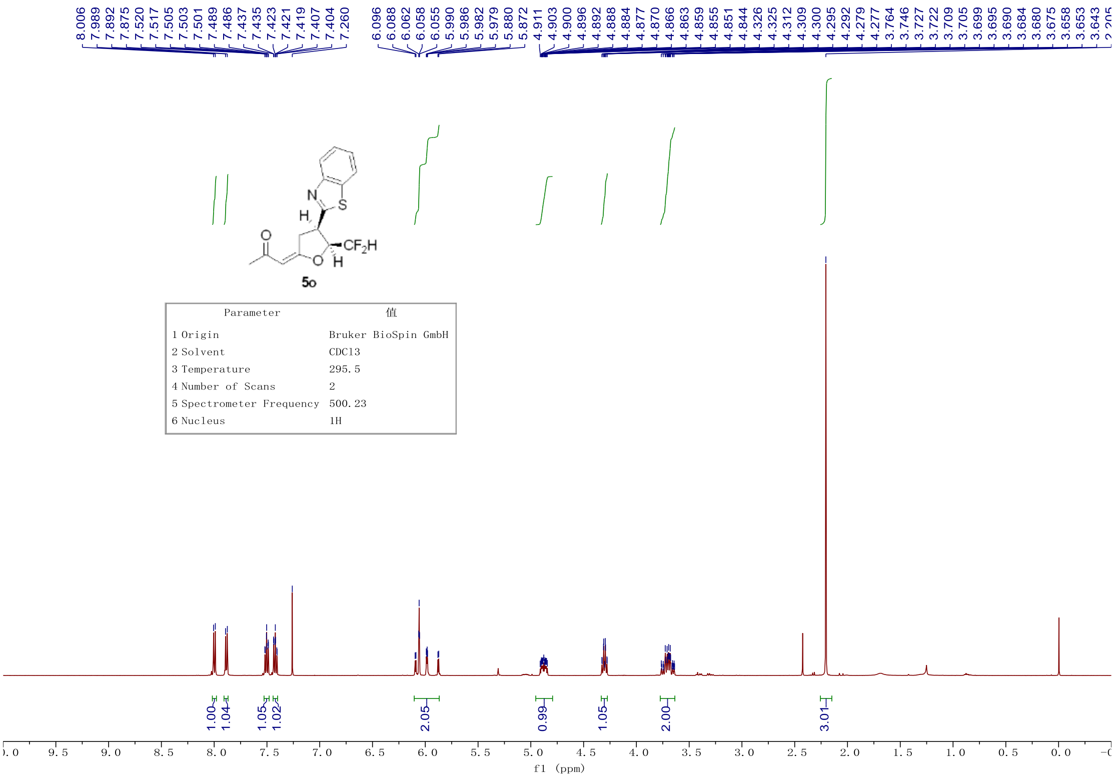

Figure S189. ^{1}H-NMR of **5o**

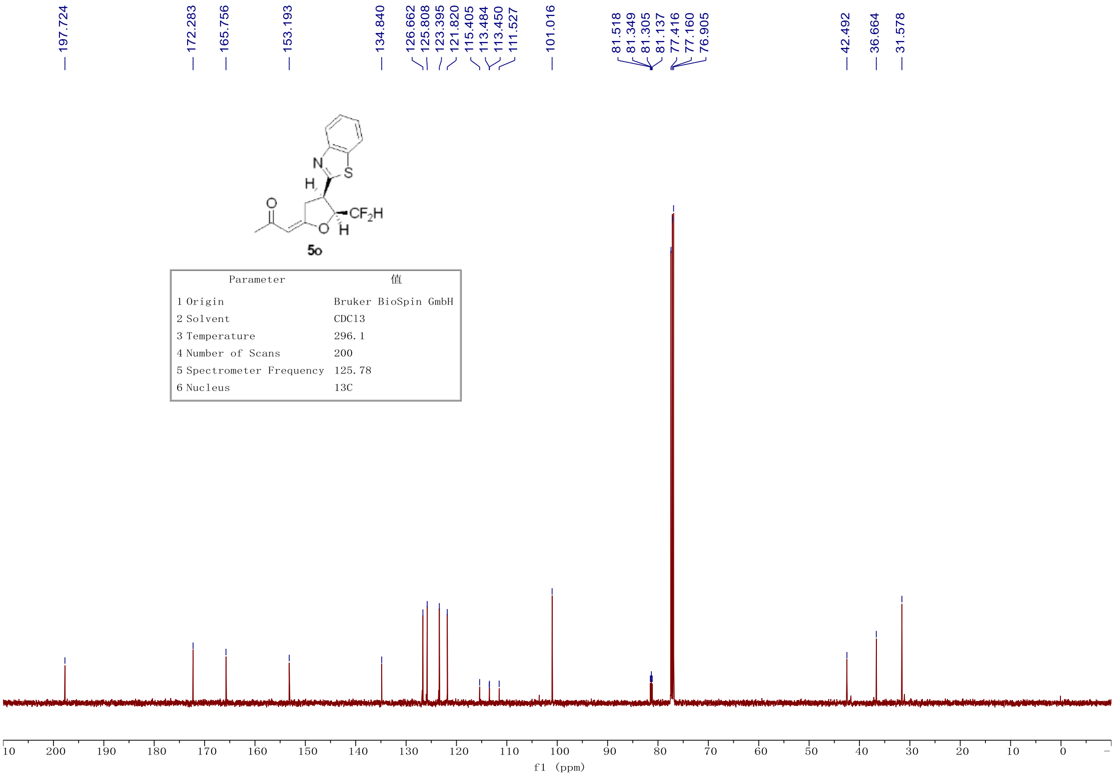

Figure S190. ^{13}C-NMR of **5o**

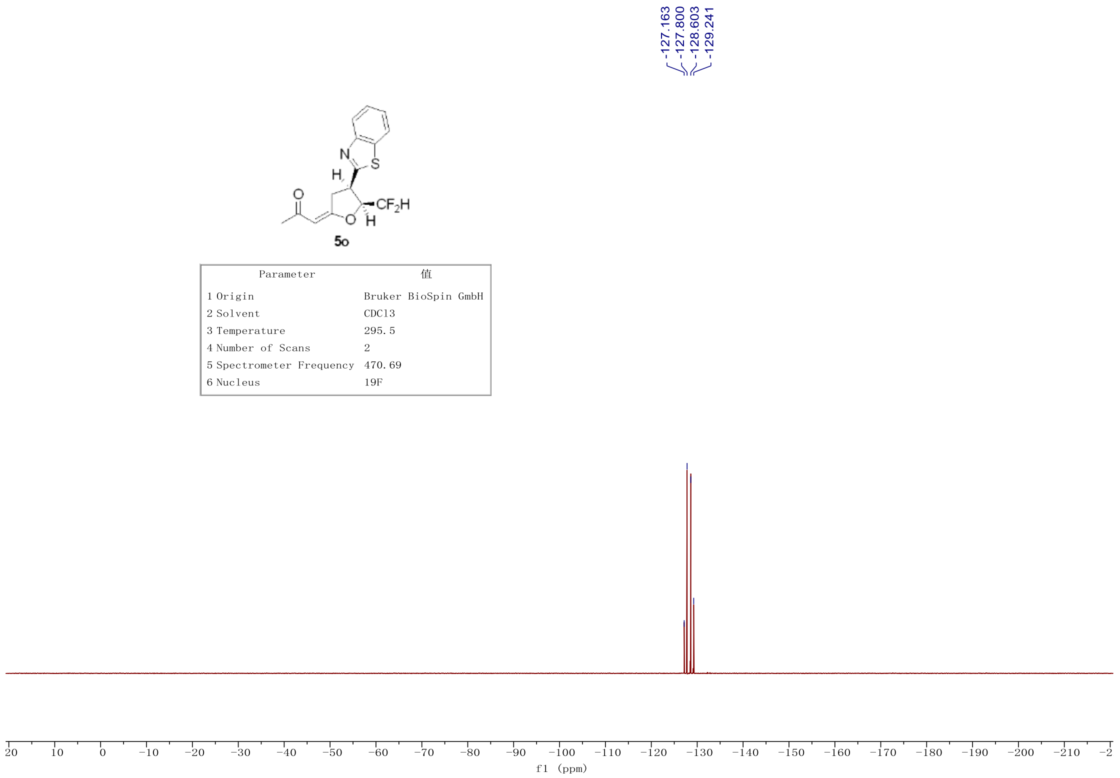

Figure S191. ^{19}F-NMR of **5o**

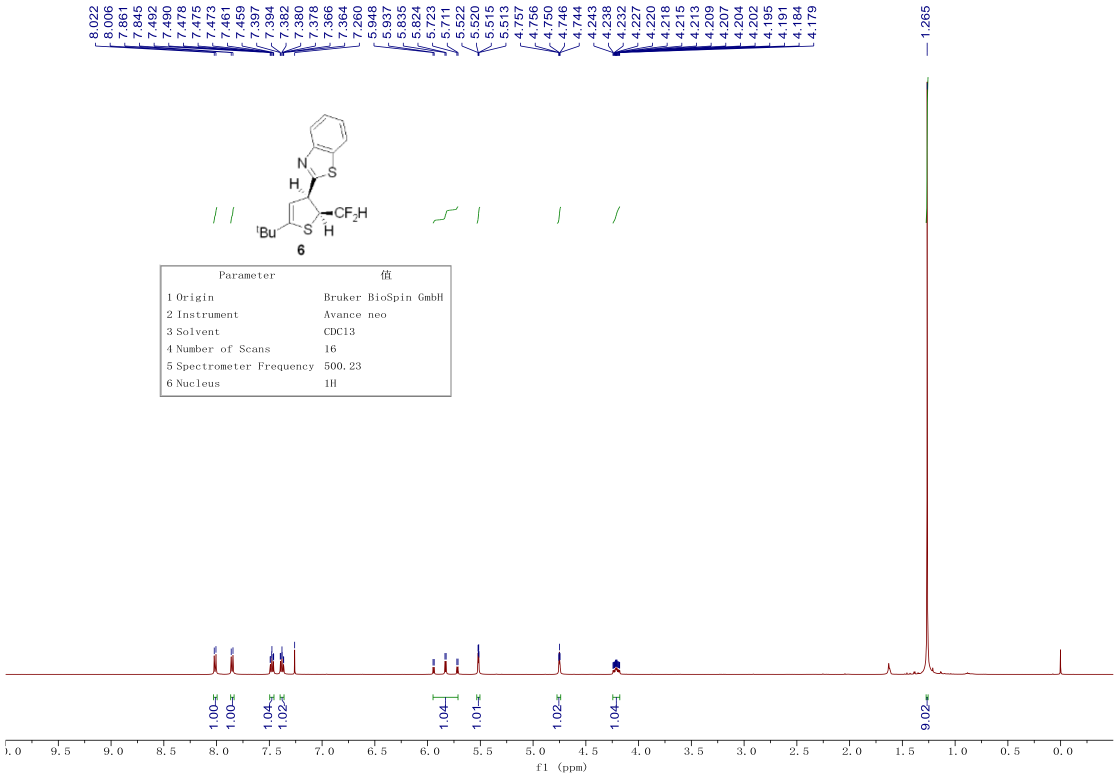

Figure S192. ^{1}H-NMR of **6**

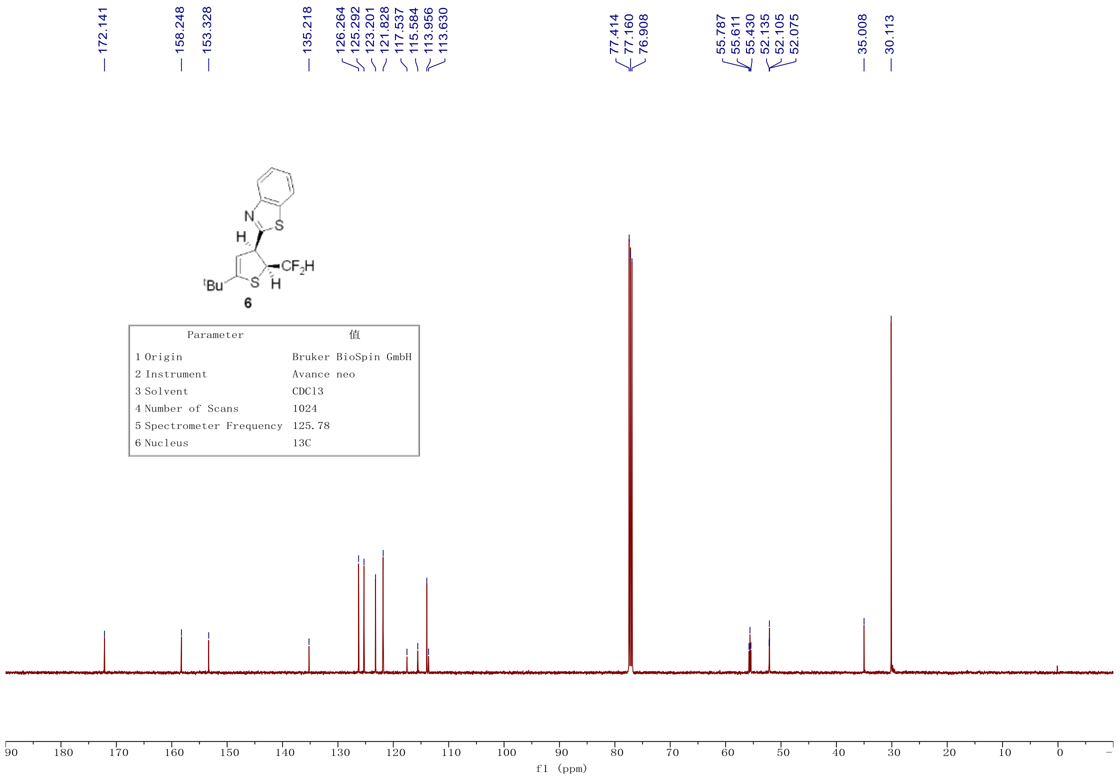

Figure S193. ^{13}C-NMR of **6**

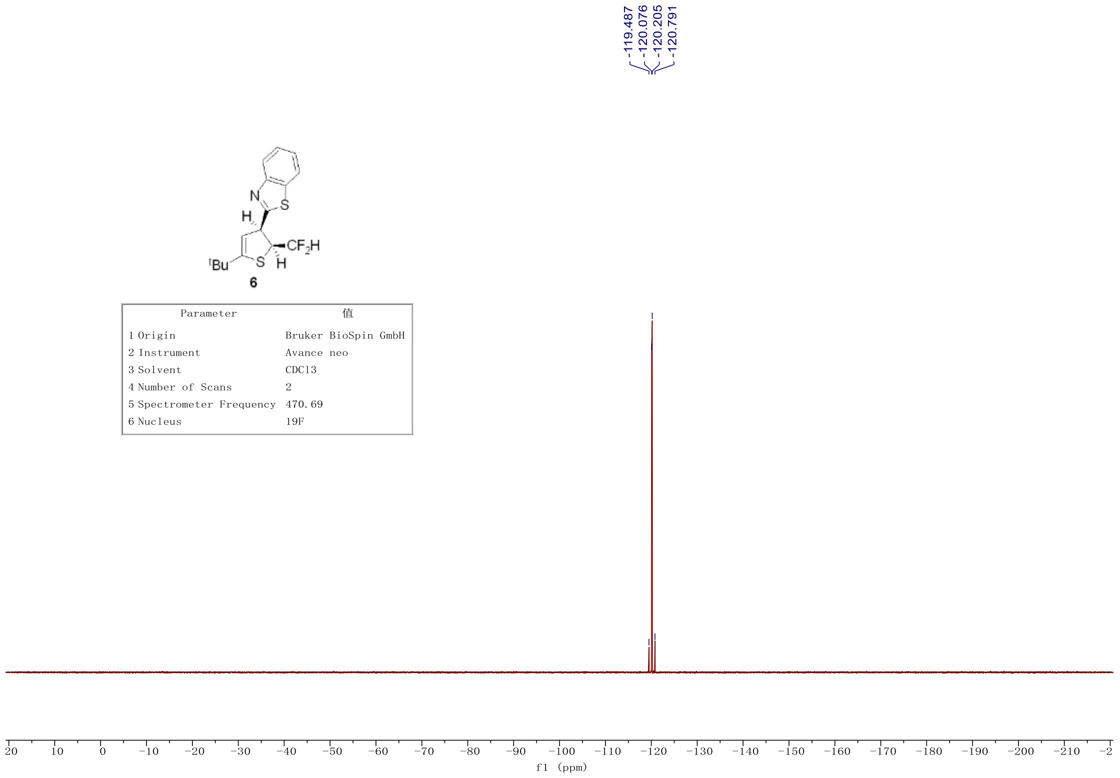

Figure S194. ^{19}F-NMR of **6**

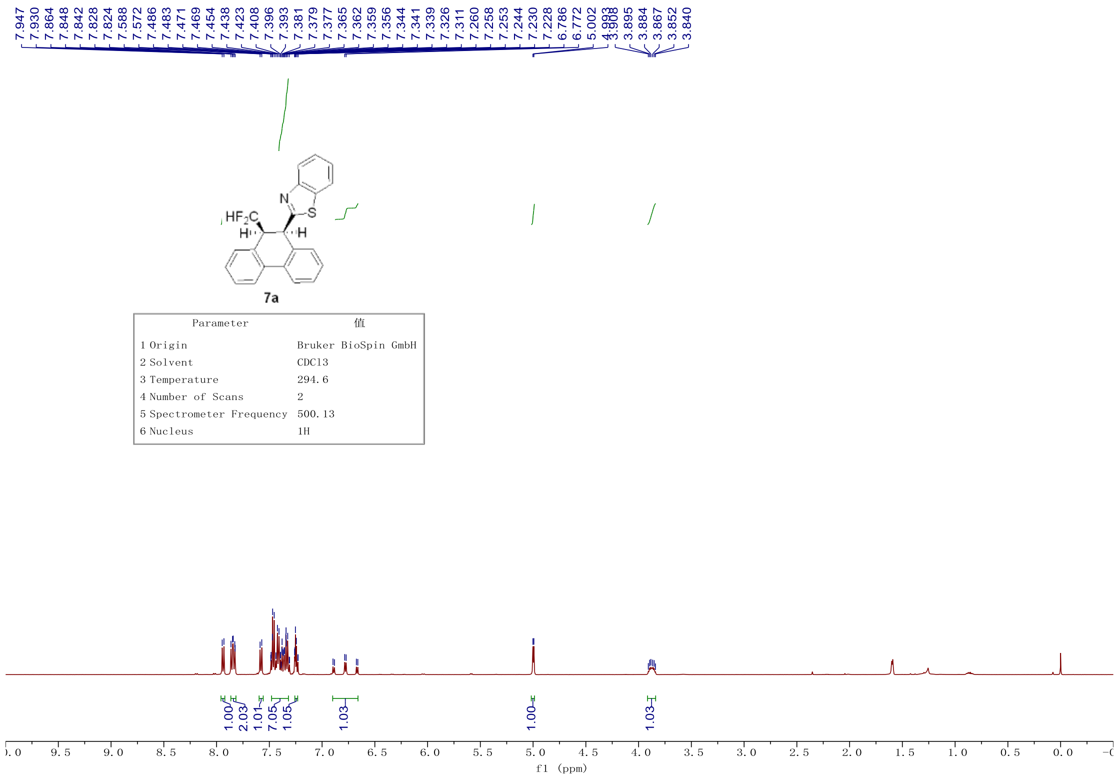

Figure S195. ^{1}H-NMR of **7a**

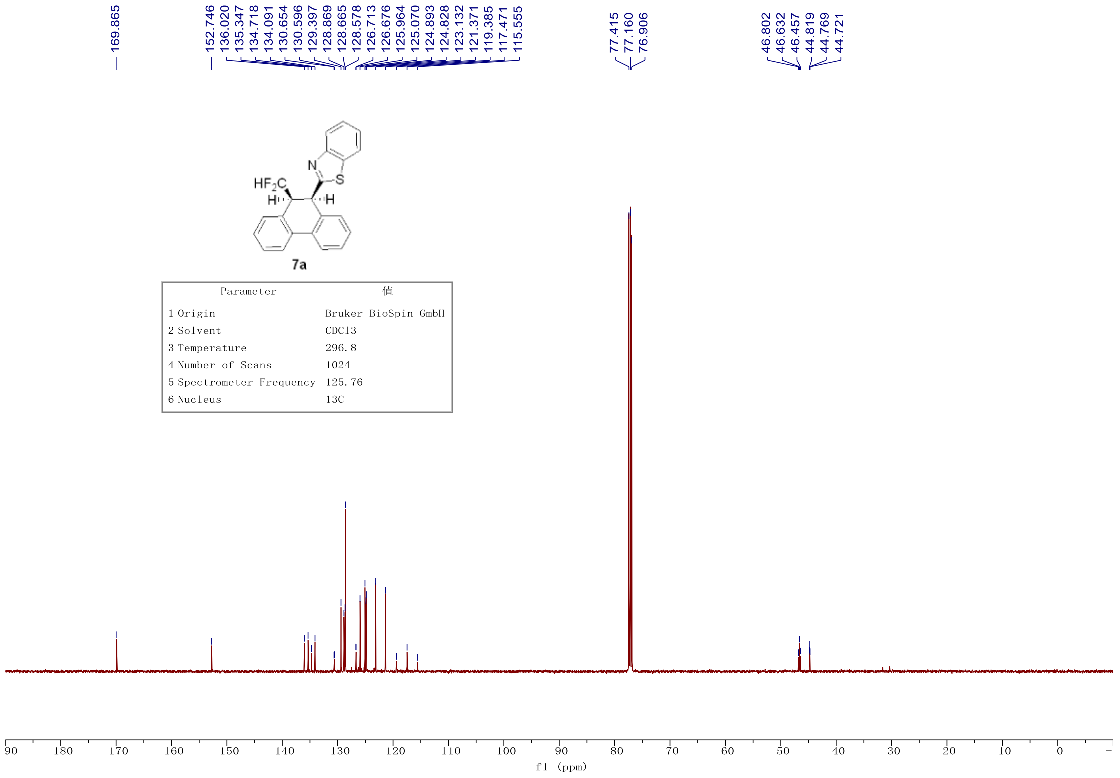

Figure S196. ^{13}C-NMR of **7a**

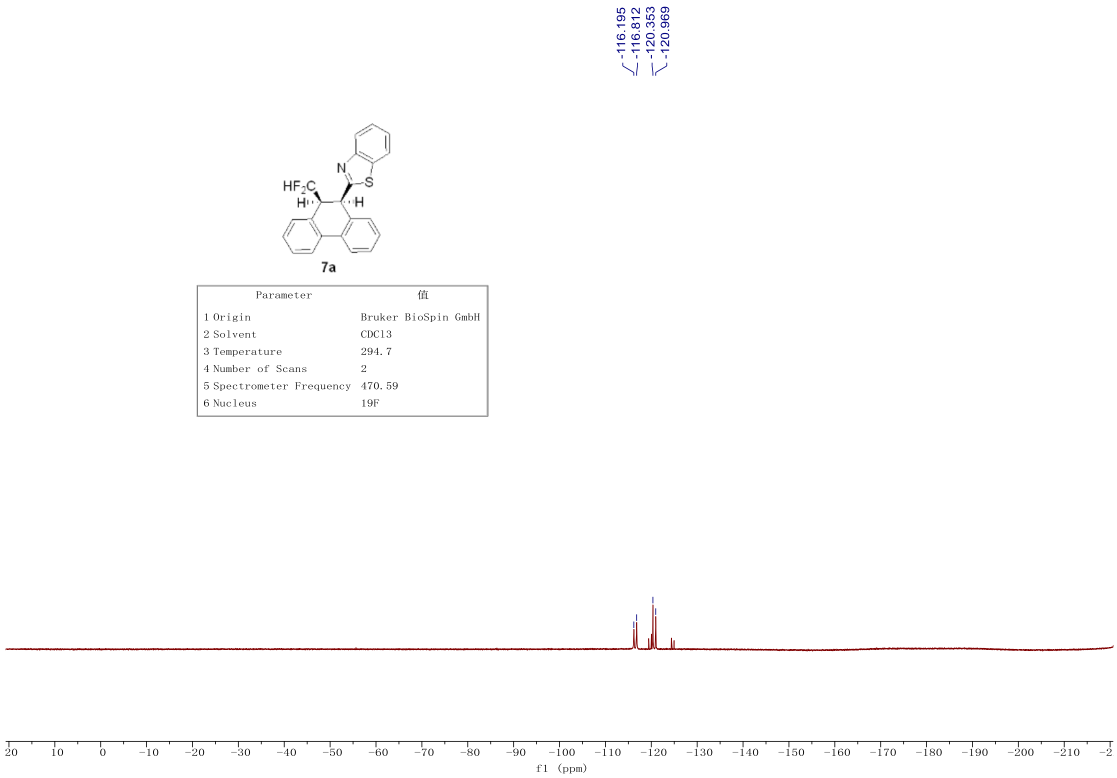

Figure S197. ^{19}F-NMR of **7a**

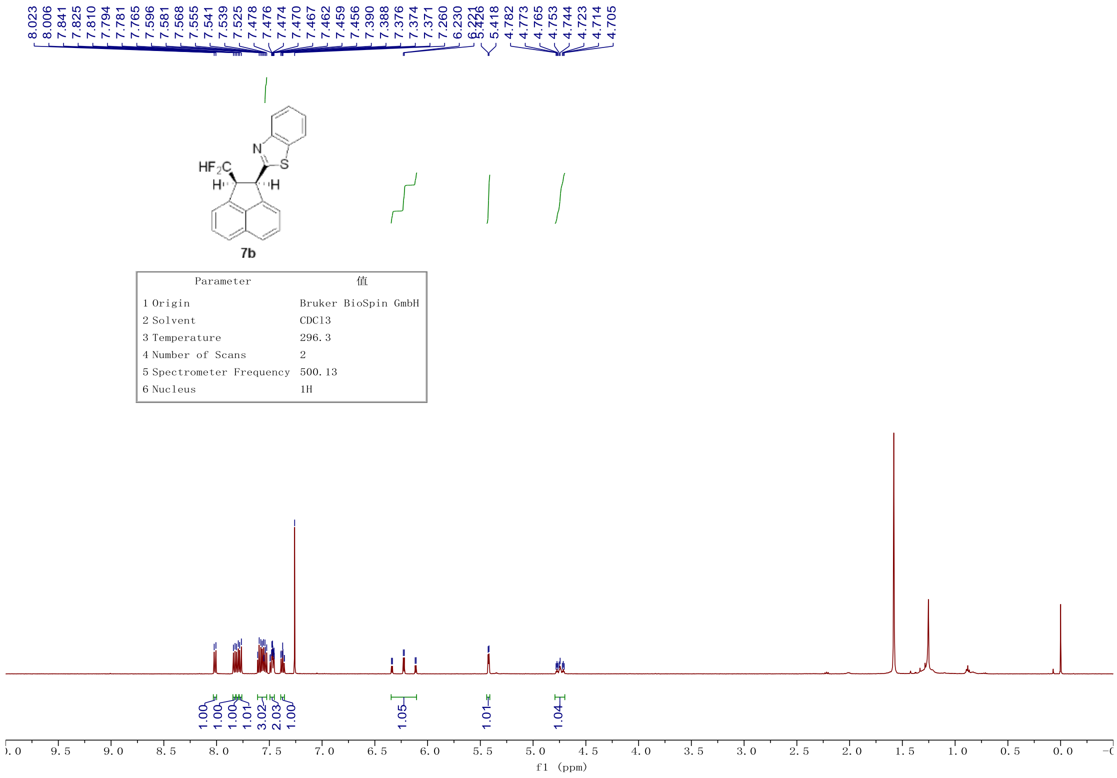

Figure S198. ^{1}H-NMR of **7b**

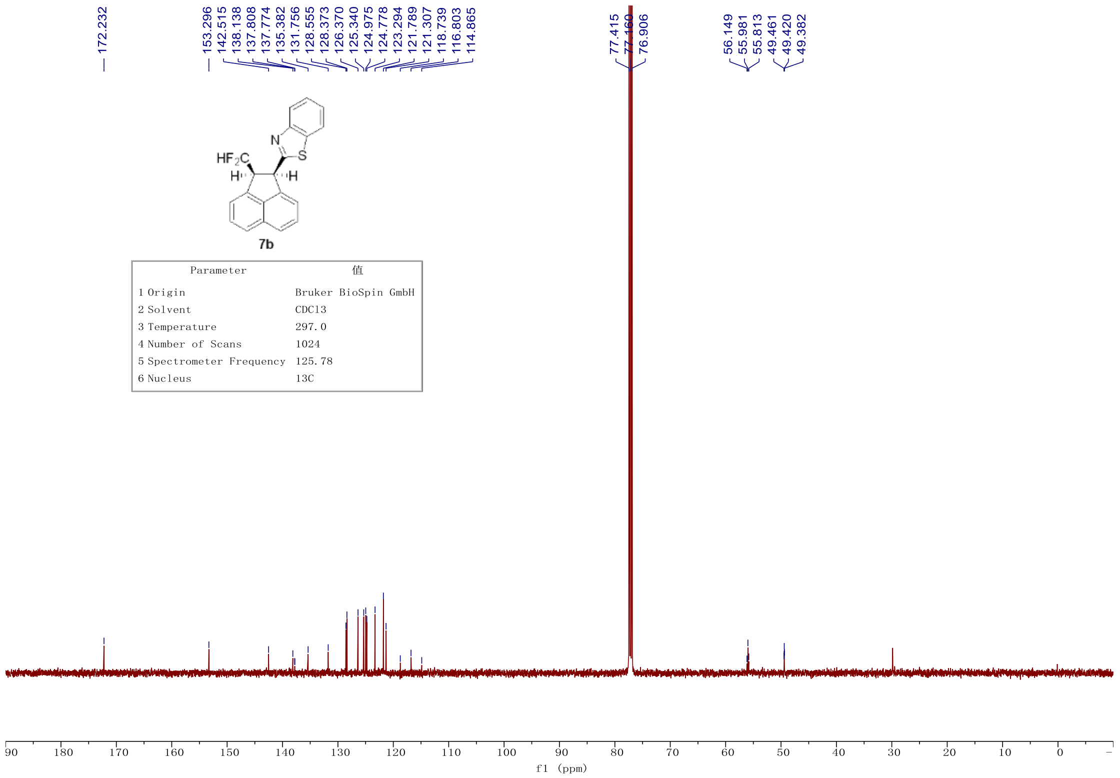

Figure S199. ^{13}C-NMR of **7b**

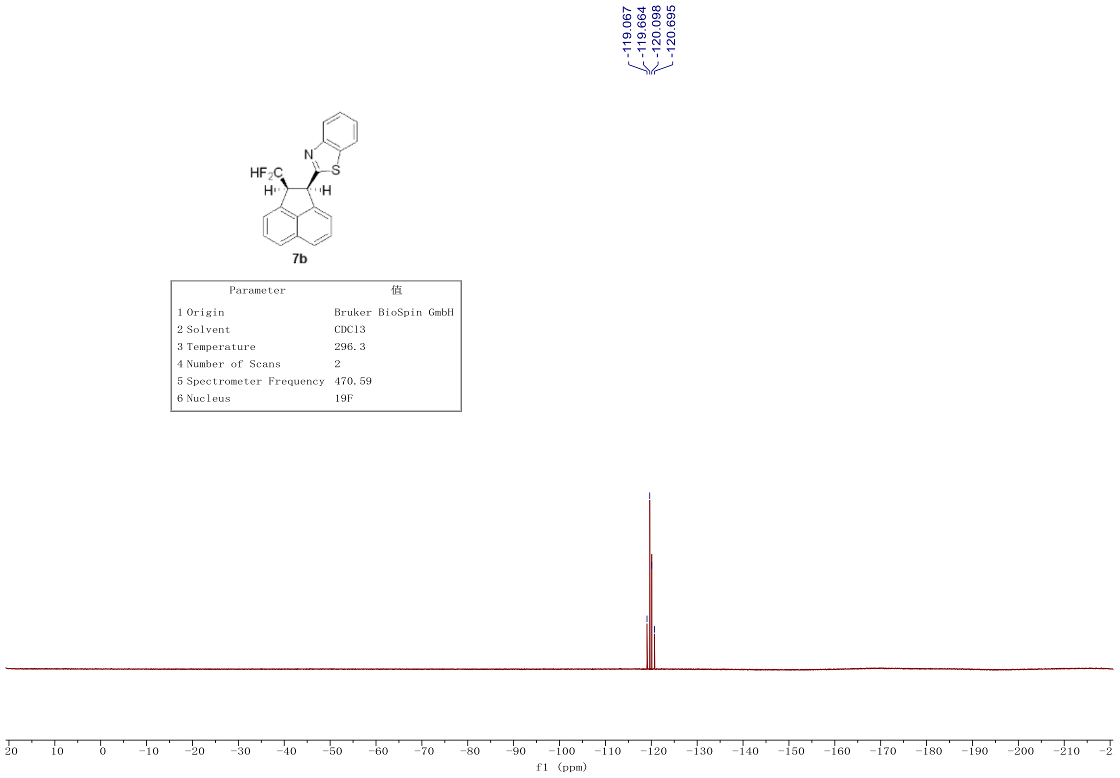

Figure S200. ^{19}F-NMR of **7b**

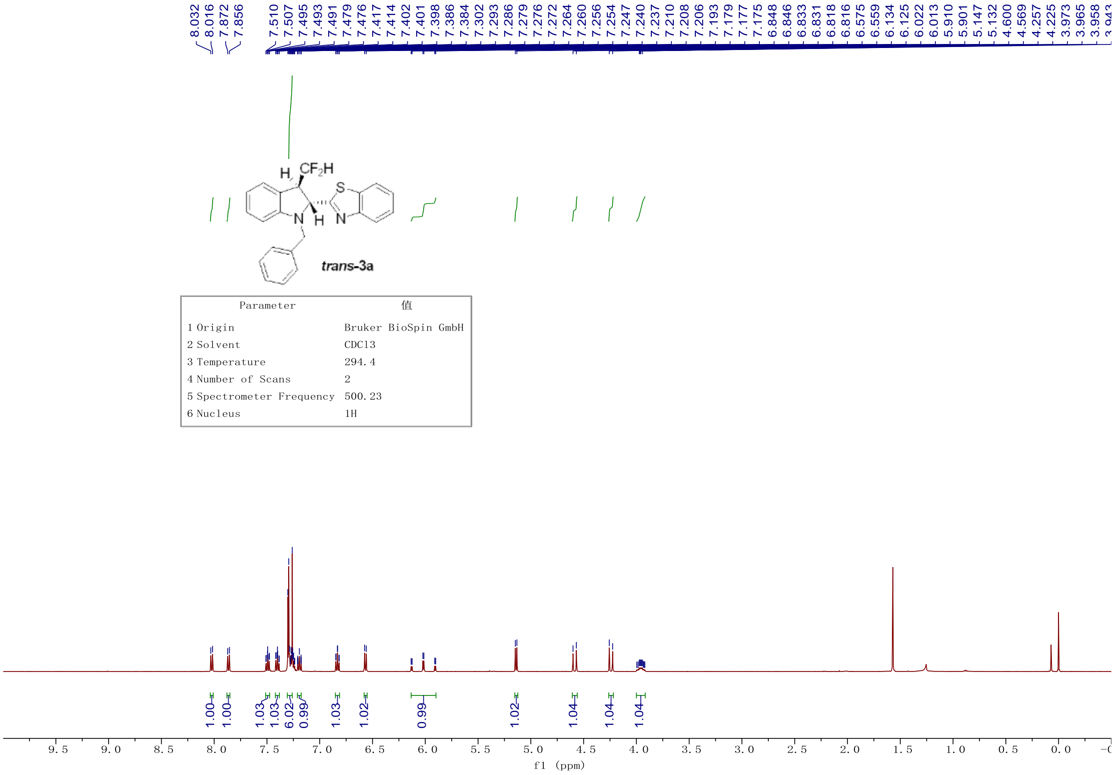

Figure S201. ^{1}H-NMR of ***trans*-3a**

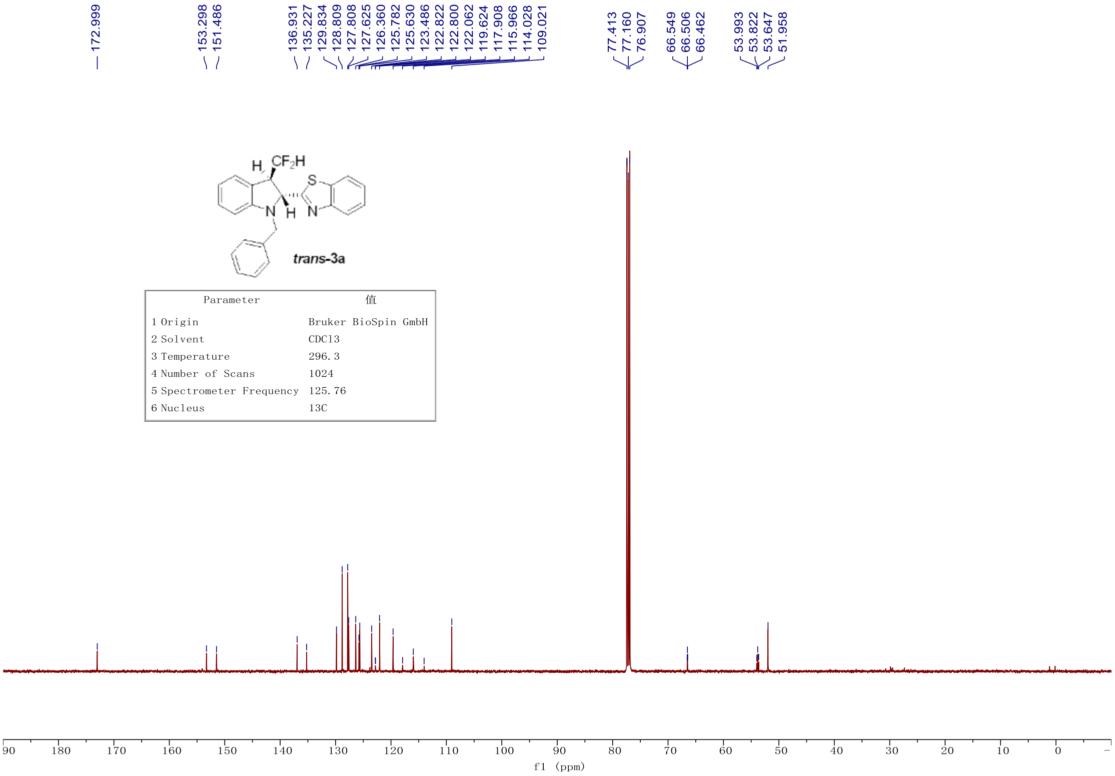

Figure S202. ^{13}C-NMR of ***trans*-3a**

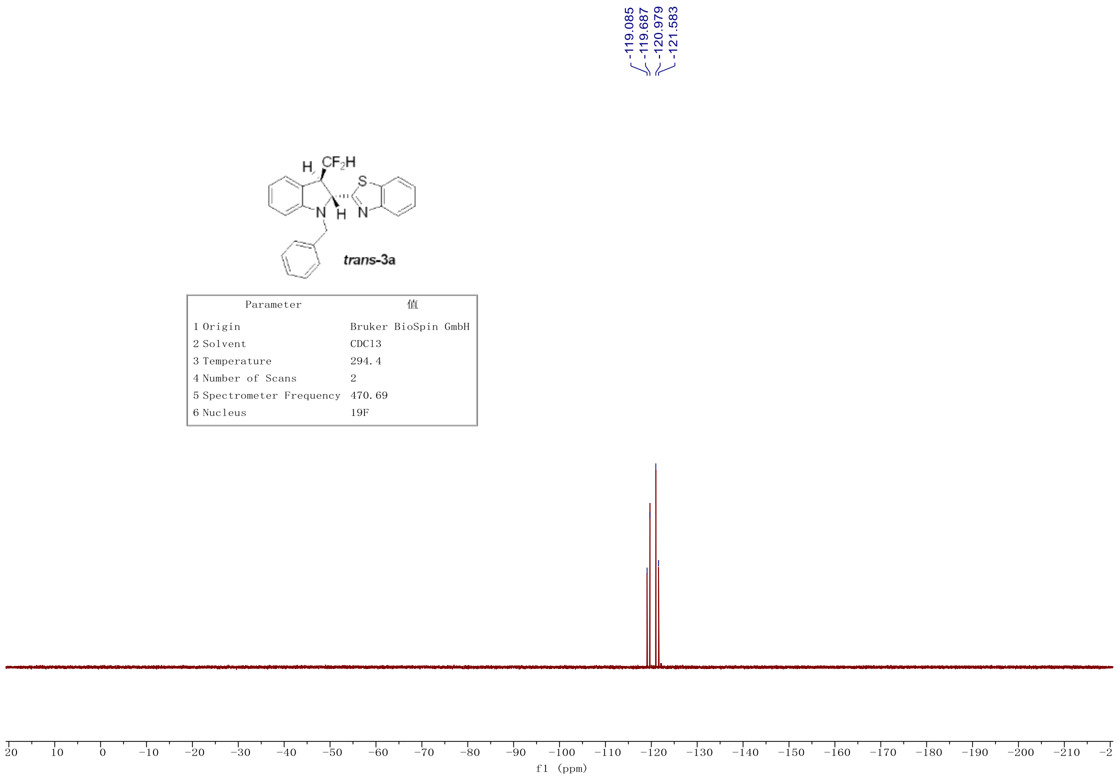

Figure S203. ^{19}F-NMR of ***trans*-3a**

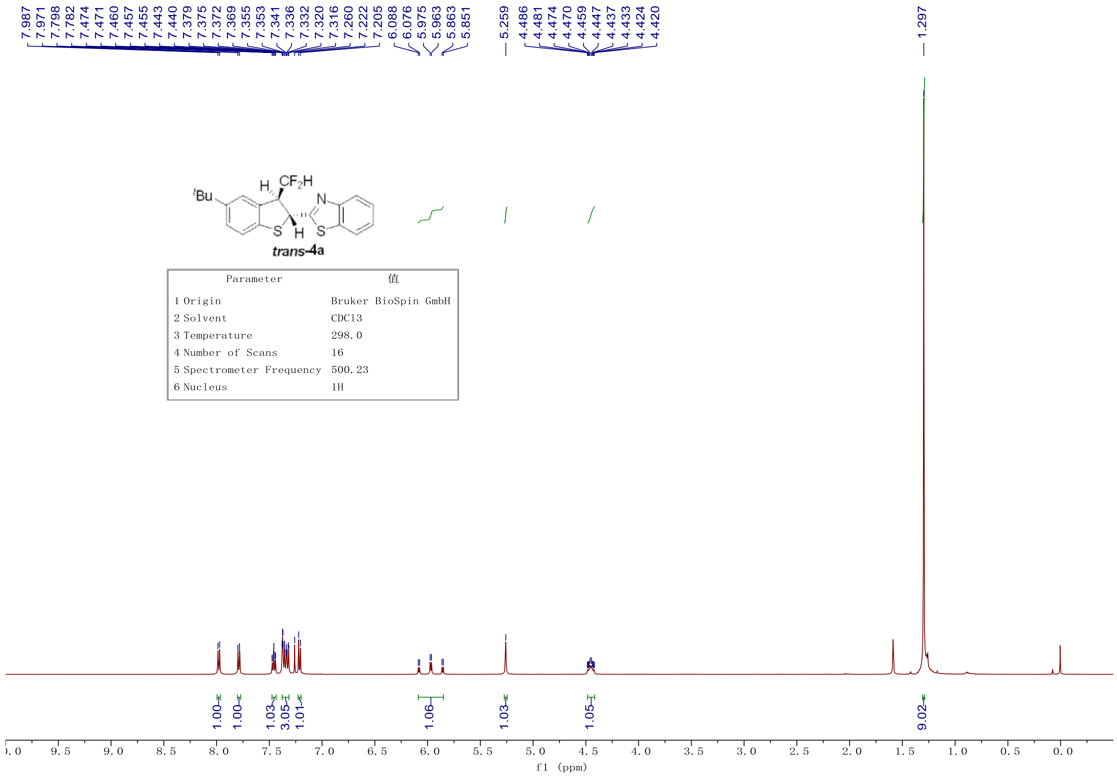

Figure S204. ^{1}H-NMR of ***trans*-4a**

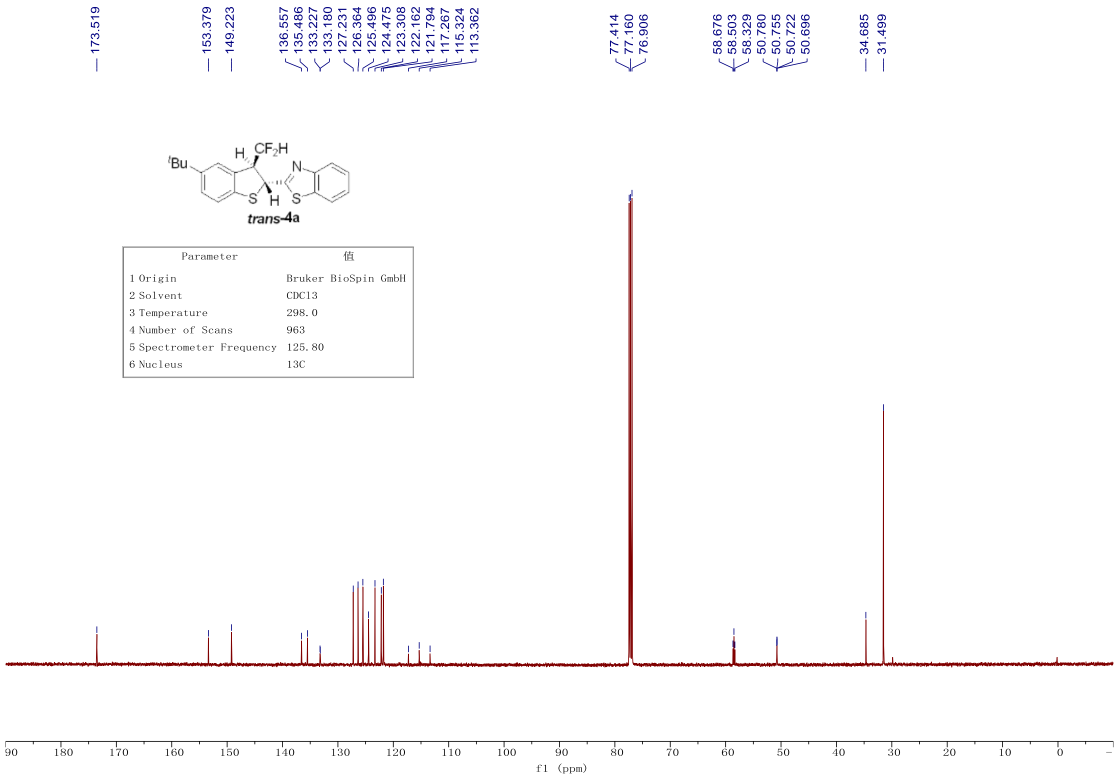

Figure S205. ^{13}C-NMR of ***trans*-4a**

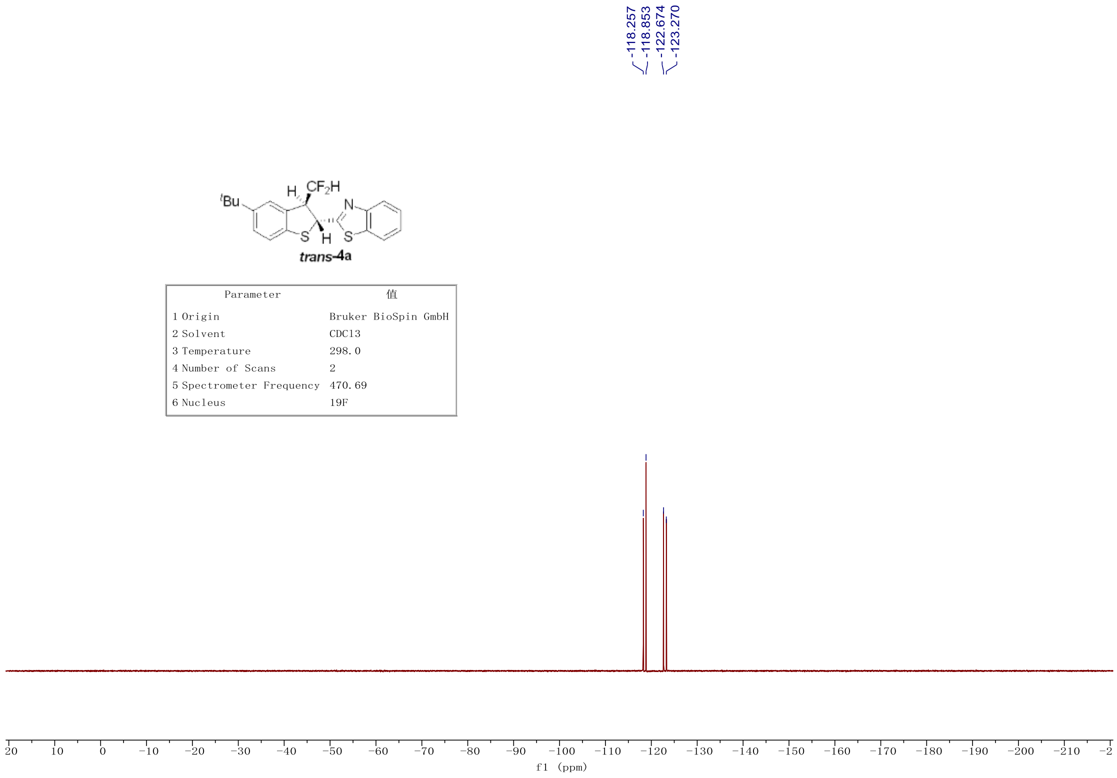

Figure S206. ^{19}F-NMR of ***trans*-4a**

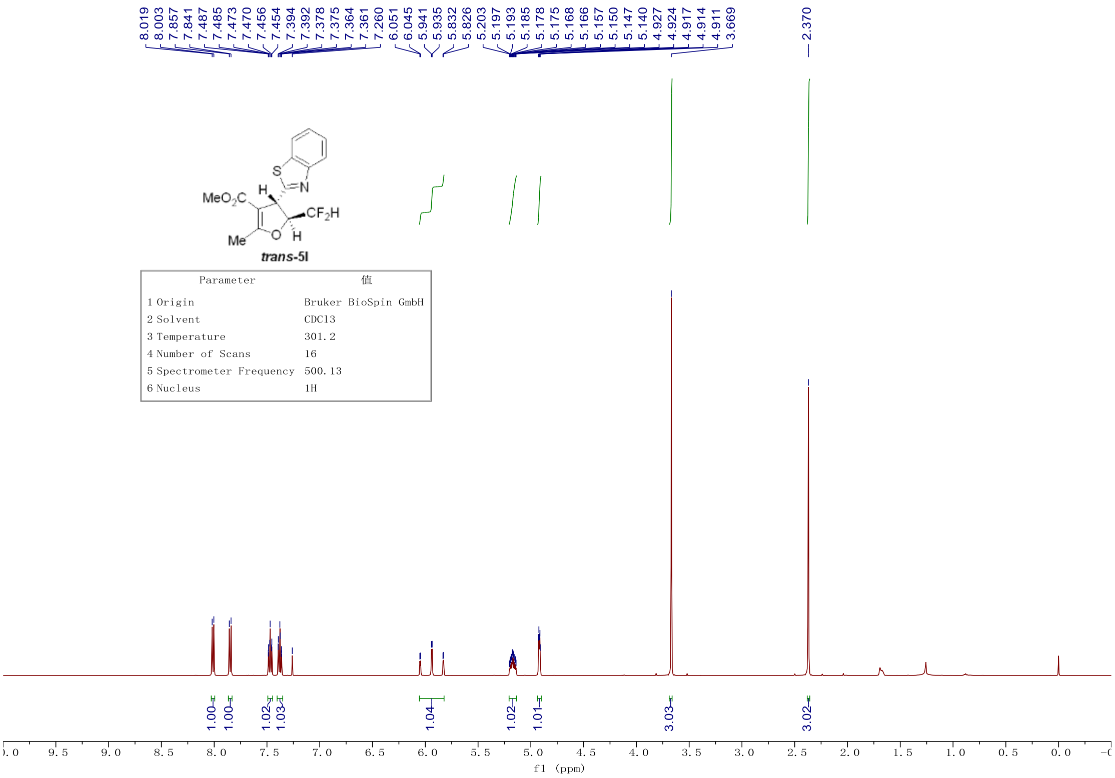

Figure S207. ^{1}H-NMR of ***trans*-5l**

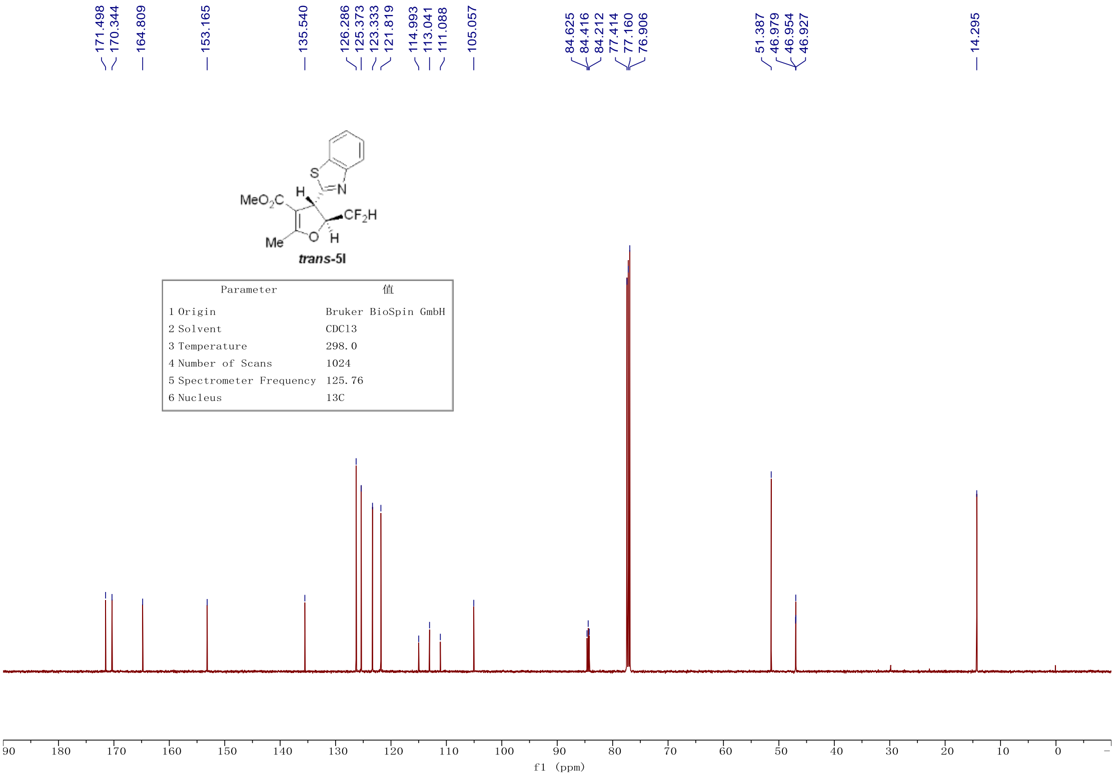

Figure S208. ^{13}C-NMR of ***trans*-5l**

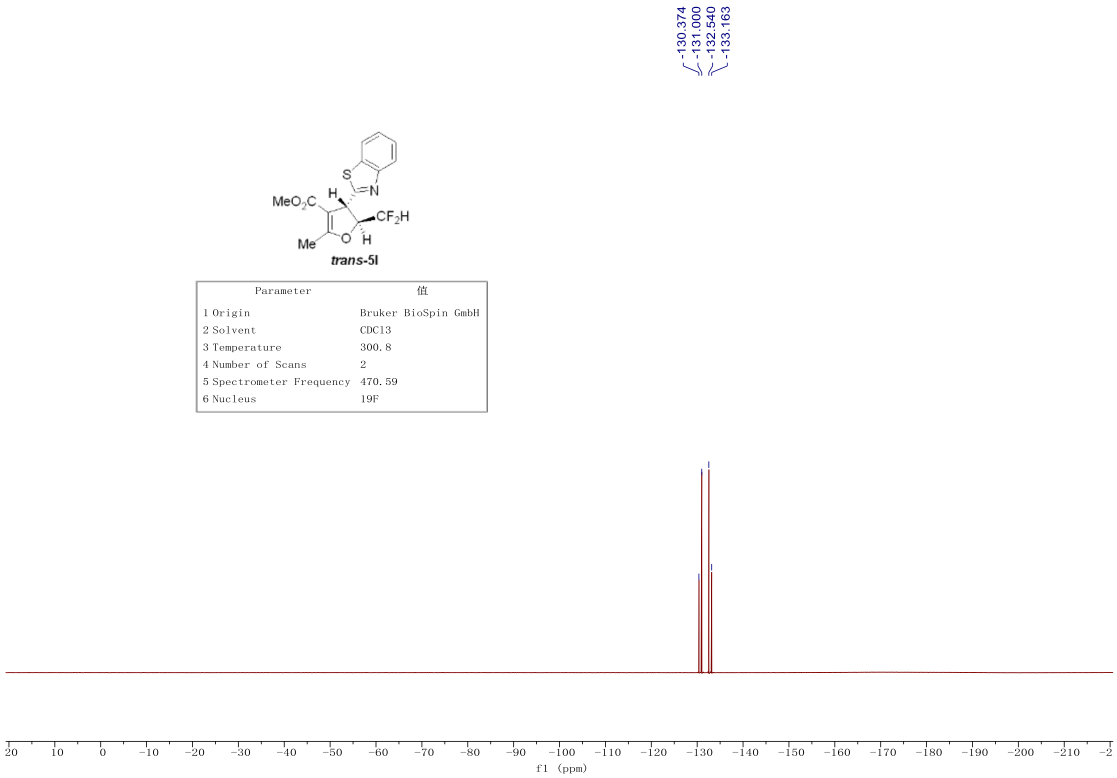

Figure S209. ^{19}F-NMR of ***trans*-5l**

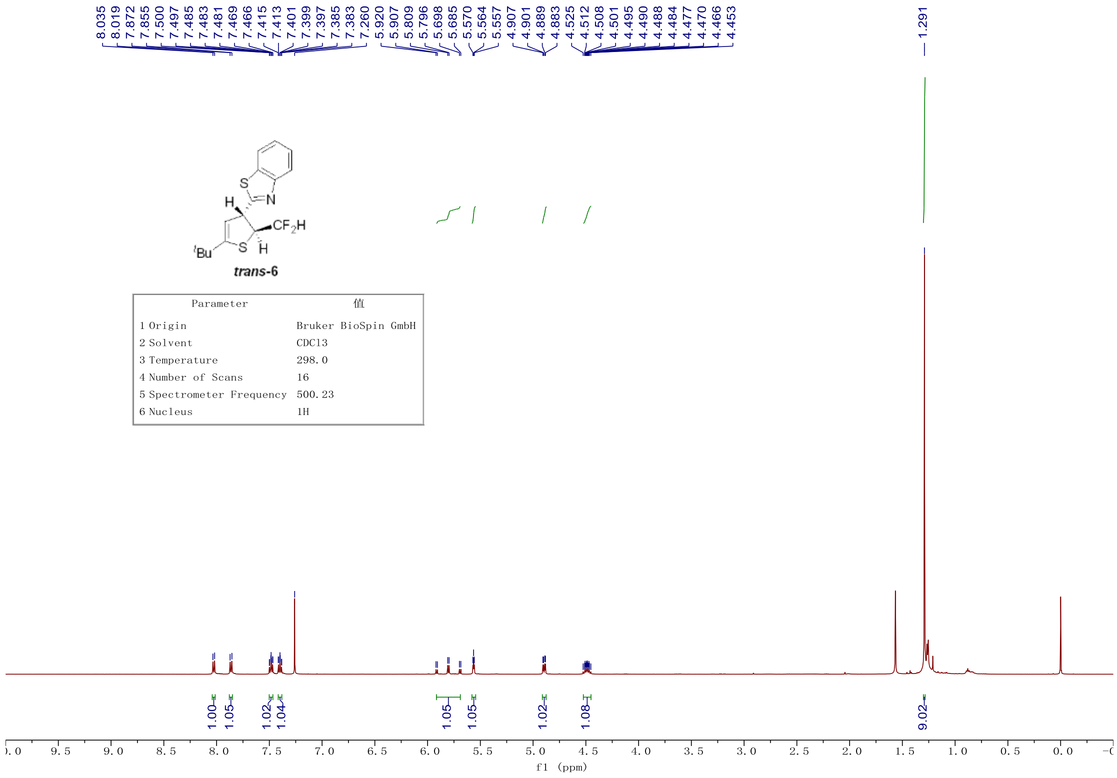

Figure S210. ^{1}H-NMR of ***trans*-6**

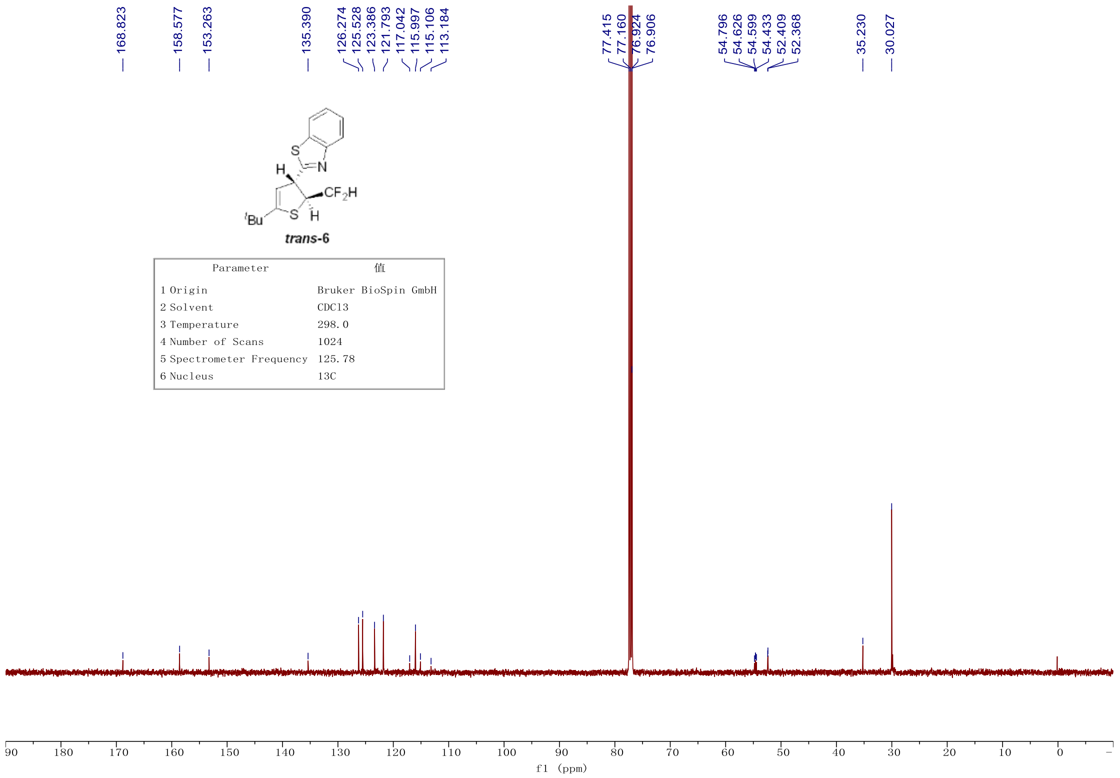

Parameter	值
1 Origin	Bruker BioSpin GmbH
2 Solvent	CDCl3
3 Temperature	298.0
4 Number of Scans	1024
5 Spectrometer Frequency	125.78
6 Nucleus	13C

Figure S211. ^{13}C-NMR of ***trans*-6**

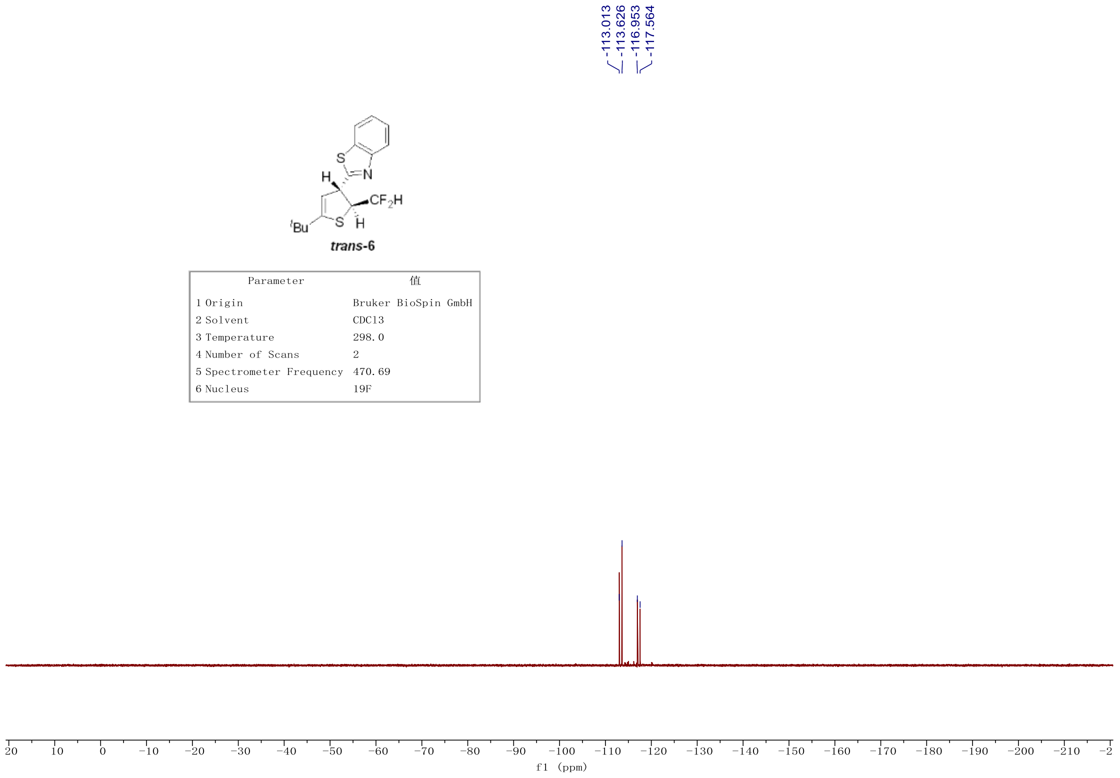

Parameter	值
1 Origin	Bruker BioSpin GmbH
2 Solvent	CDCl3
3 Temperature	298.0
4 Number of Scans	2
5 Spectrometer Frequency	470.69
6 Nucleus	19F

Figure S212. ^{19}F-NMR of ***trans*-6**

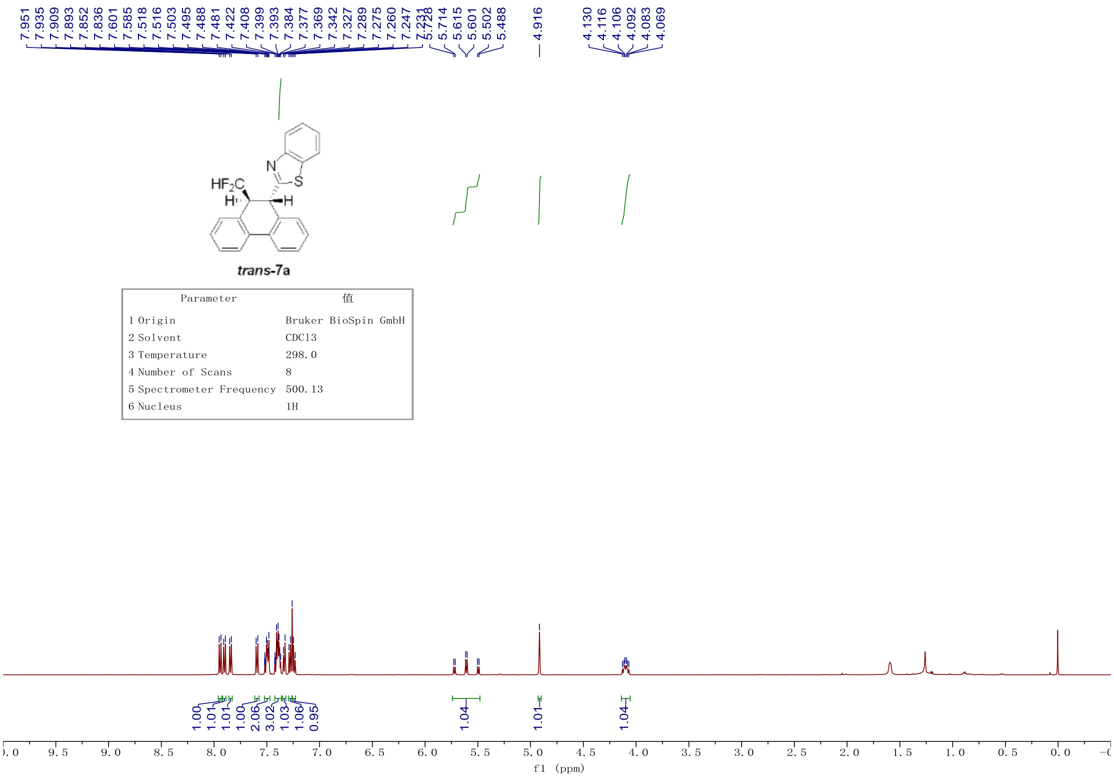

Figure S213. ^{1}H-NMR of ***trans*-7a**

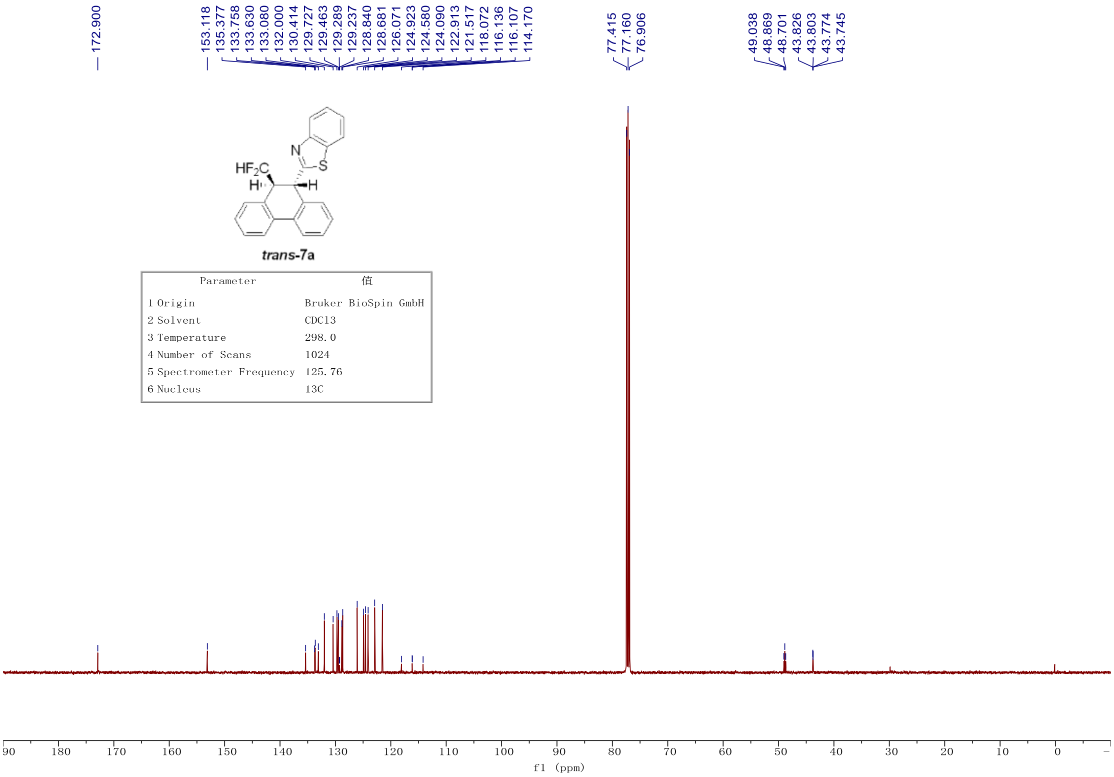

Figure S214. ^{13}C-NMR of ***trans*-7a**

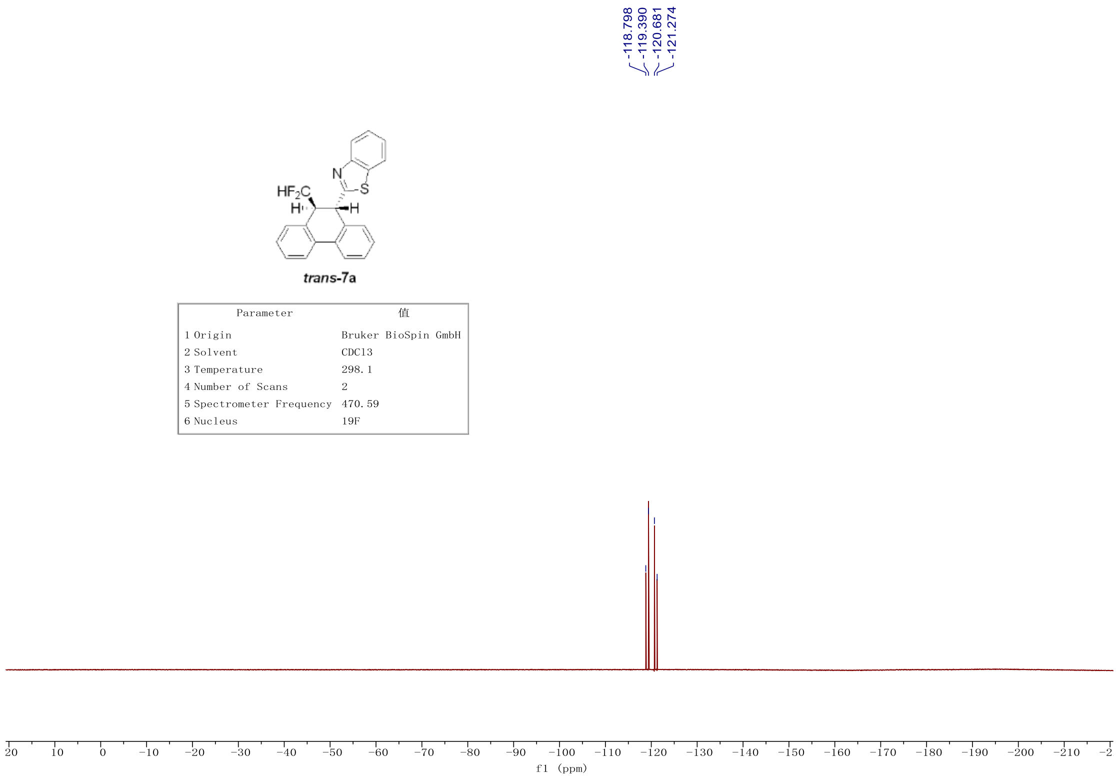

Figure S215. ^{19}F-NMR of ***trans*-7a**

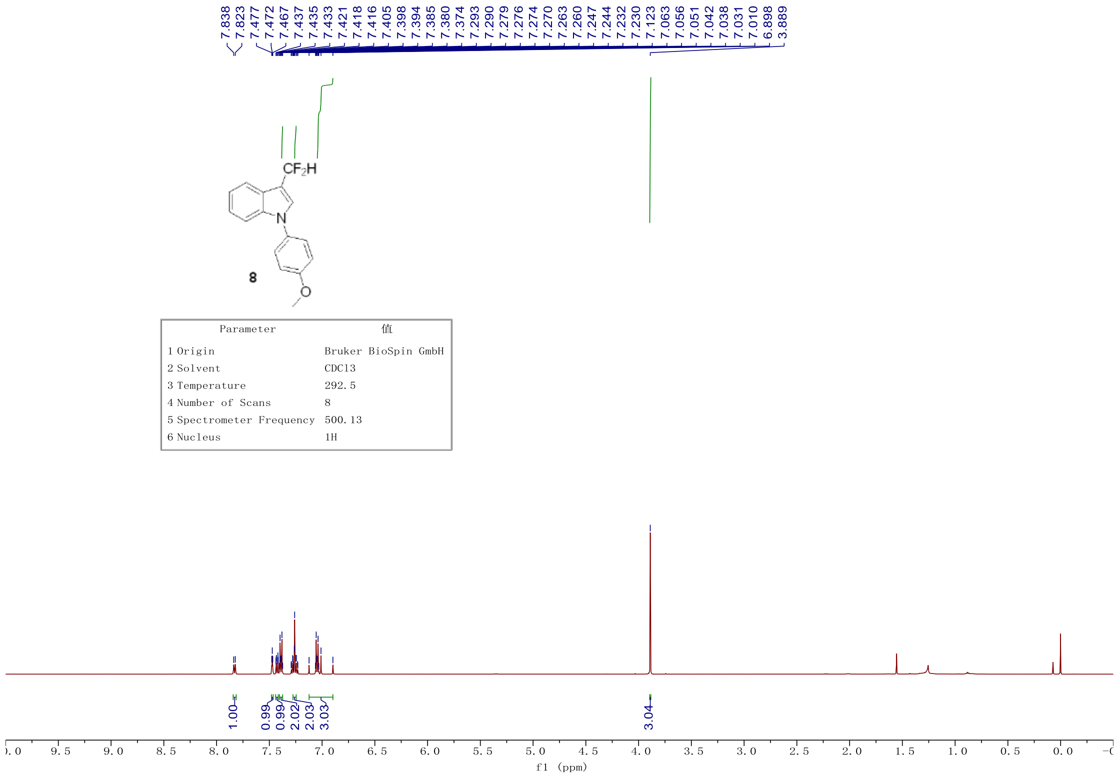

Figure S216. ^{1}H-NMR of **8**

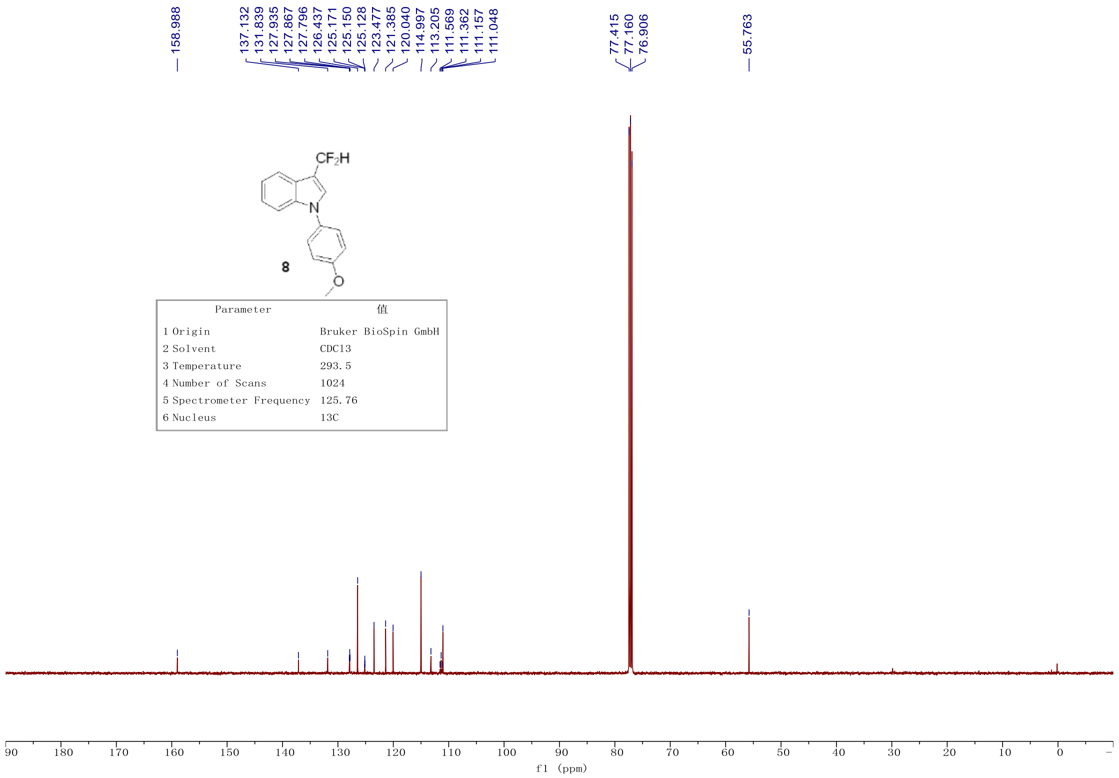

Figure S217. ^{13}C-NMR of **8**

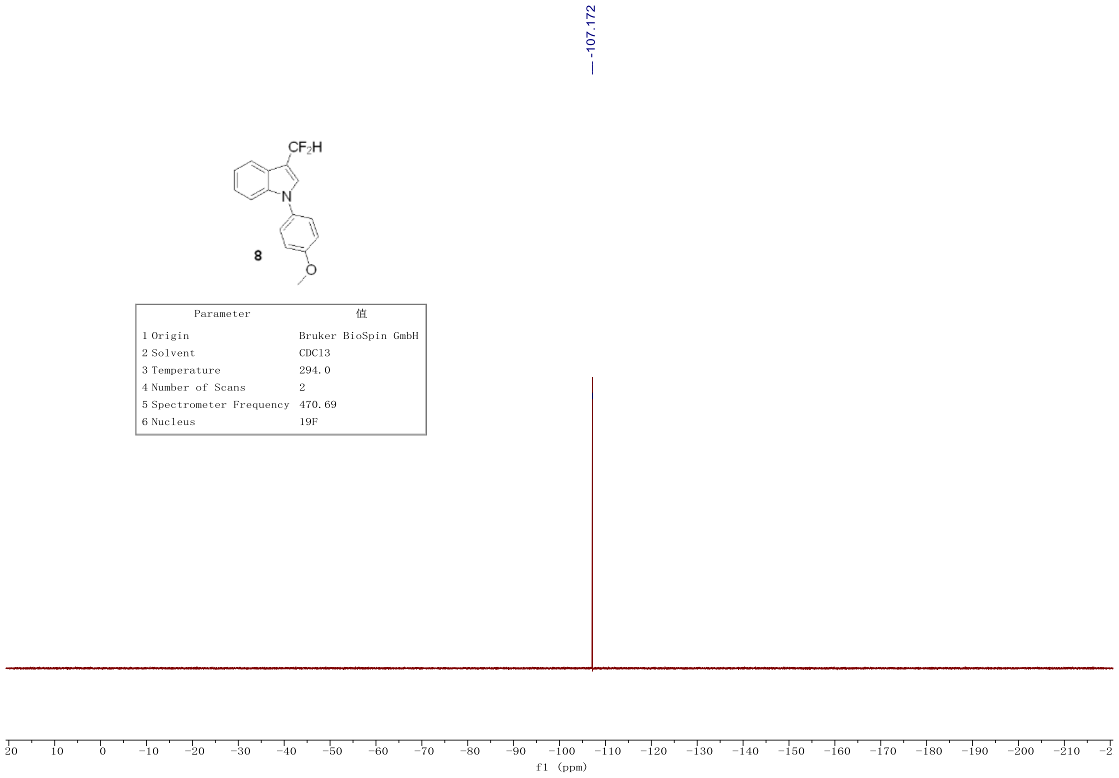

Figure S218 ^{19}F-NMR of **8**

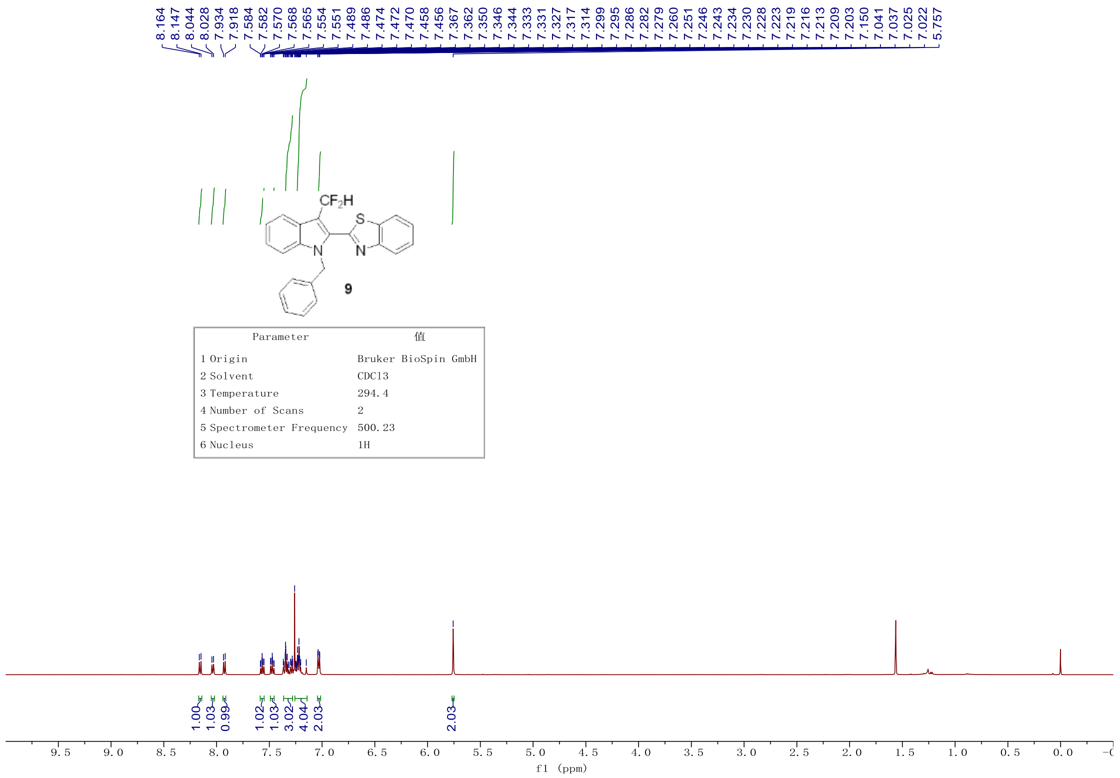

Figure S219. ^{1}H-NMR of **9**

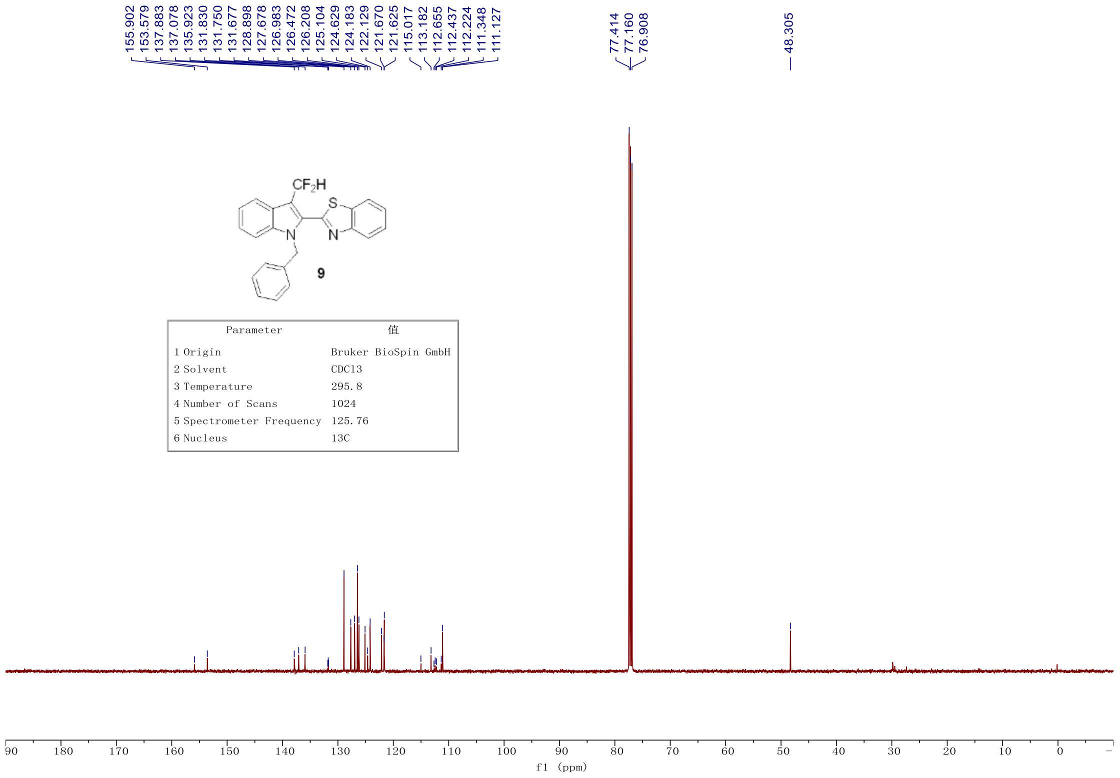

Figure S220. ^{13}C-NMR of **9**

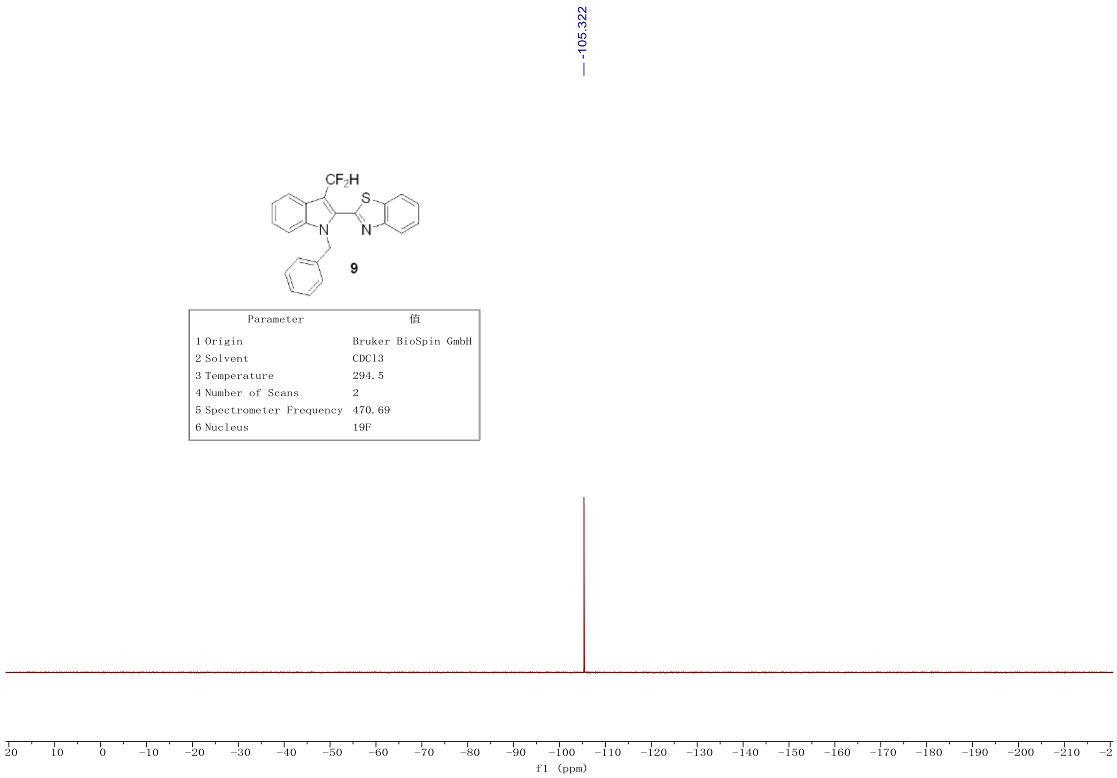

Figure S221. ^{19}F-NMR of **9**

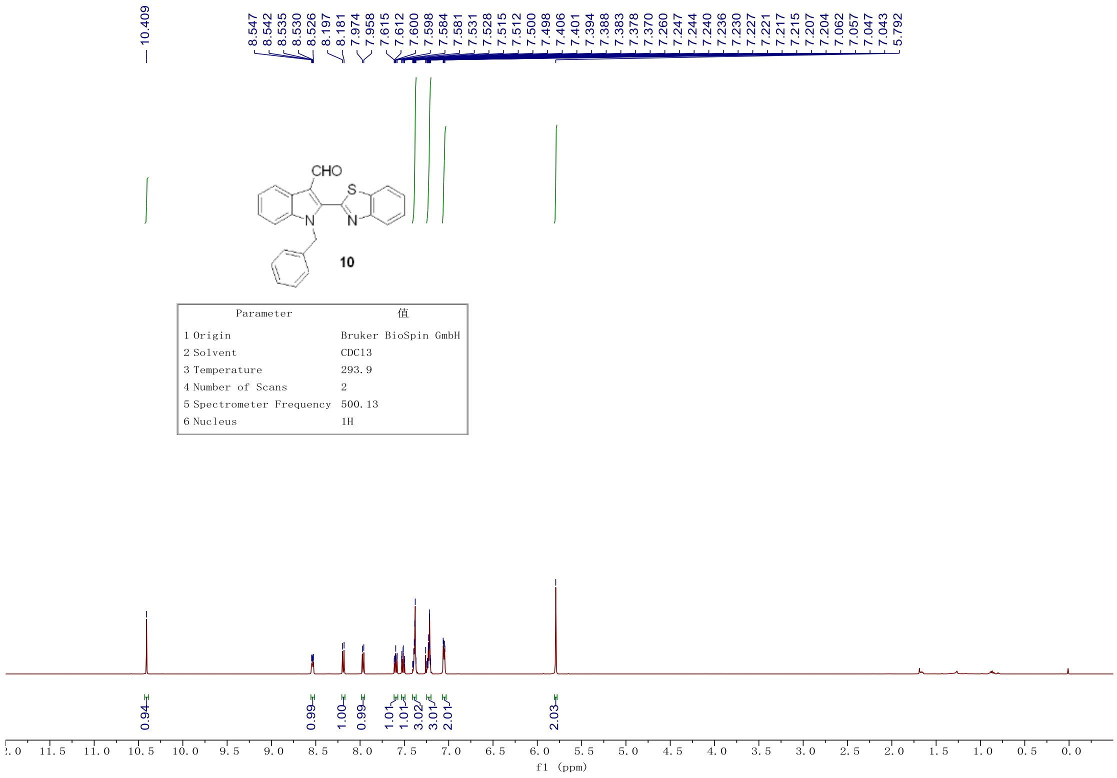

Figure S222. ^{1}H-NMR of **10**

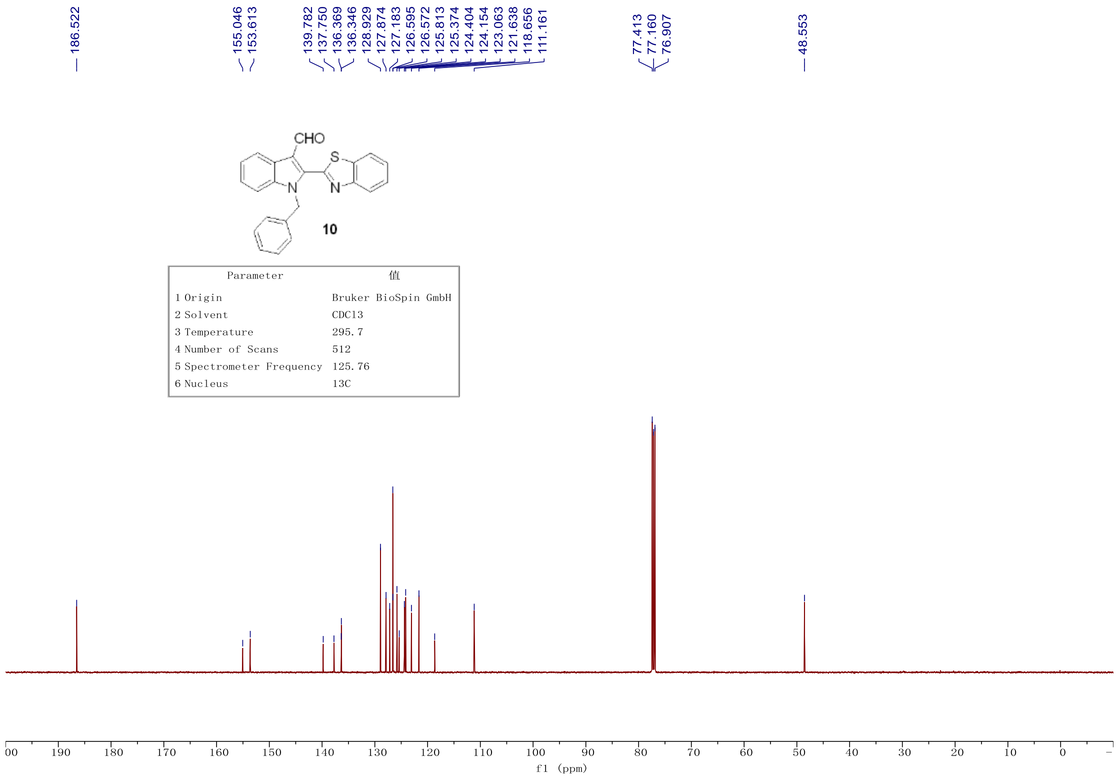

Figure S223. ^{13}C-NMR of **10**

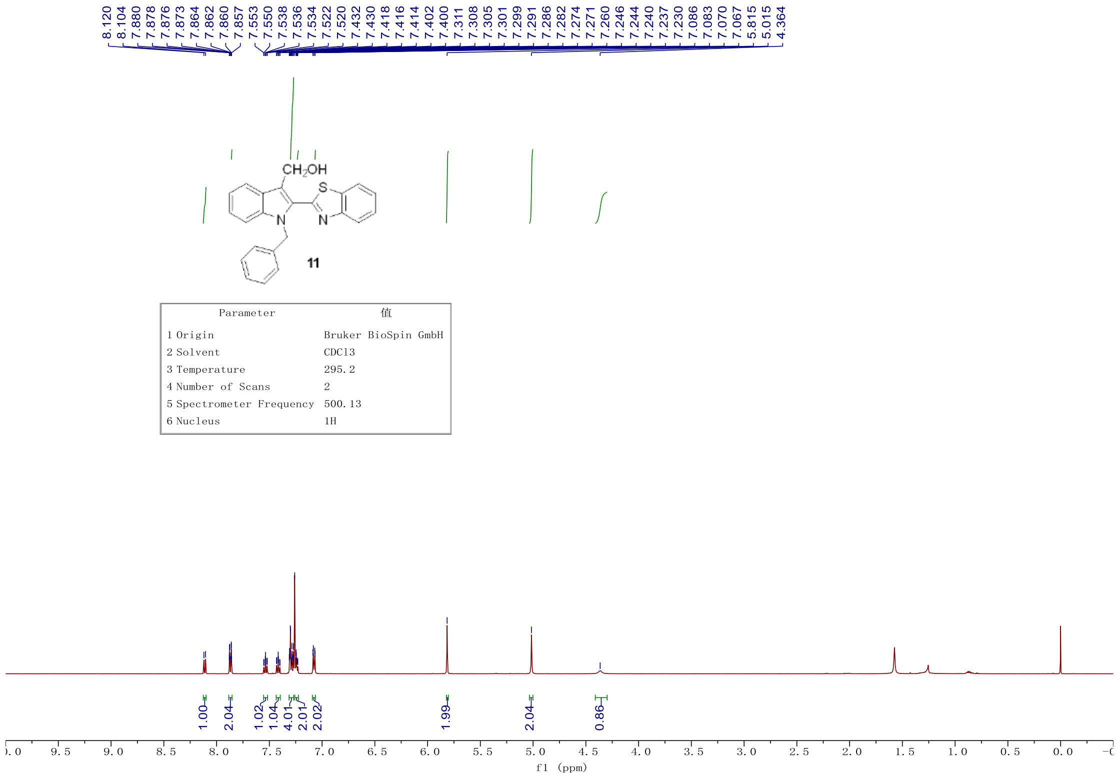

Figure S224. ^{1}H-NMR of **11**

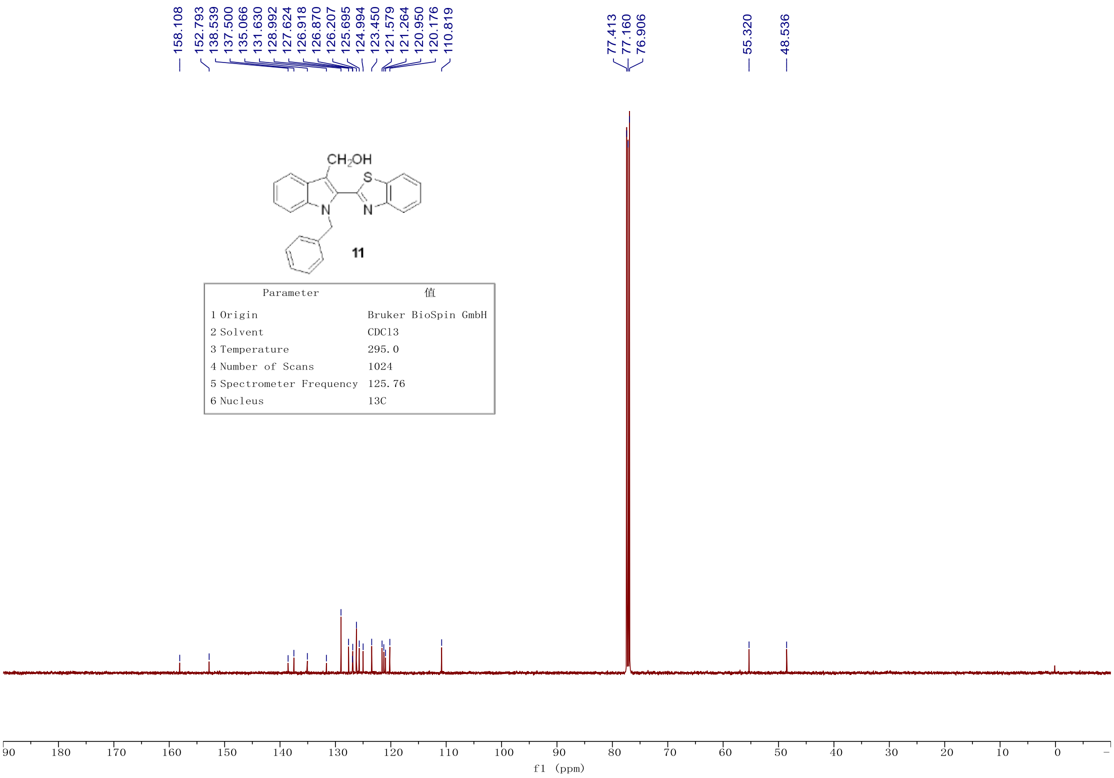

Figure S225. ^{13}C-NMR of **11**

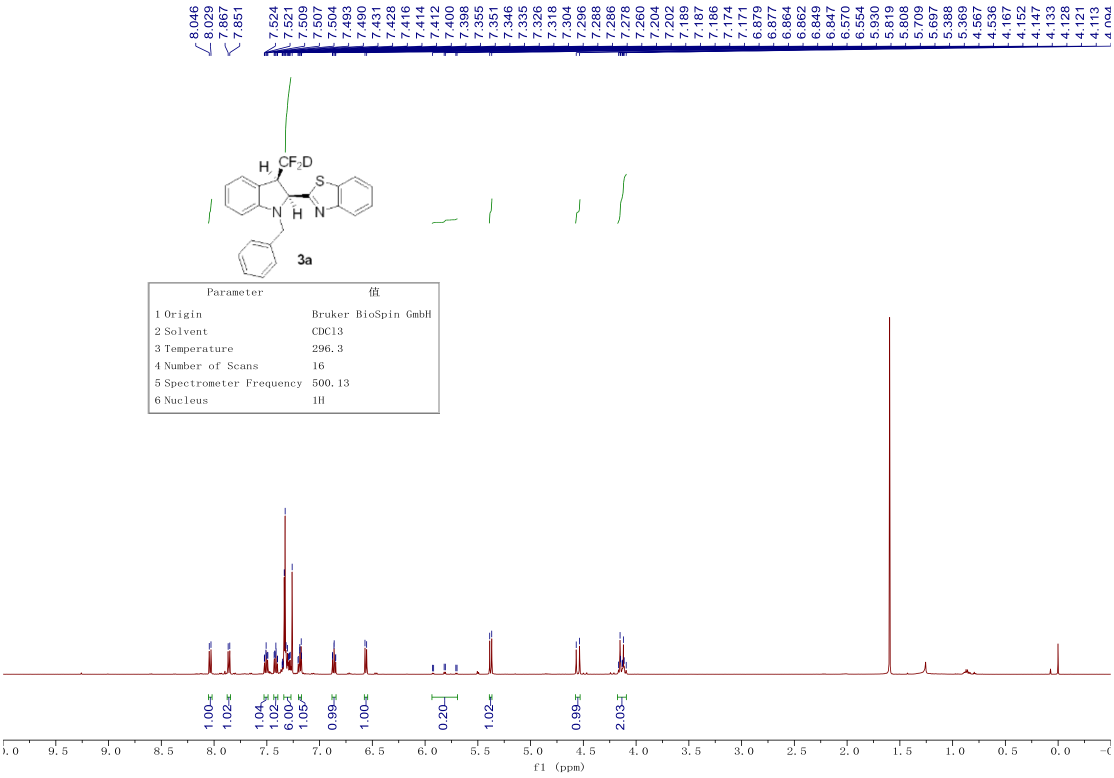

Figure S226. ^{1}H-NMR of ***d*-3a**

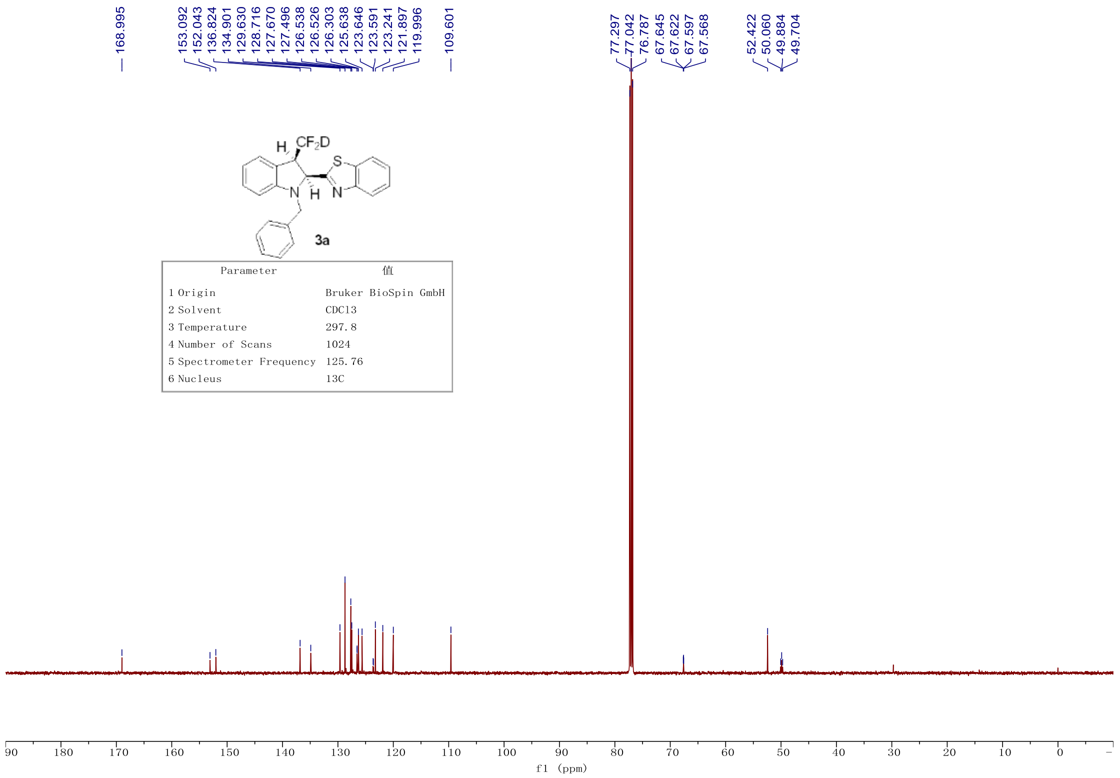

Figure S227. ^{13}C-NMR of ***d*-3a**

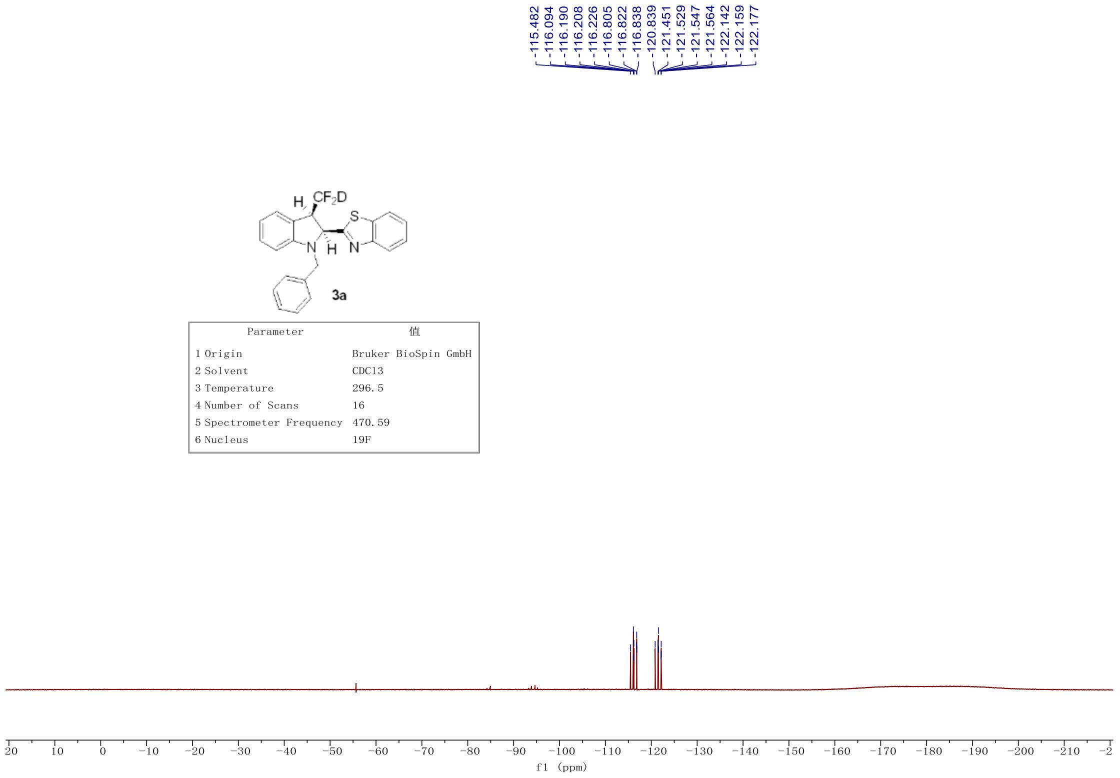

Figure S228. ^{19}F-NMR of ***d*-3a**

9. DFT calculations

All DFT calculations were performed with the Gaussian16 program.[17] Conformational searches for all molecules were carried out using the xTB and CREST programs at the theoretical level of GFN2-xTB.[18–21] Geometry optimizations were performed by using ωB97XD exchange-correlation functional in conjunction with def2-SVP basis set under vacuum conditions.[22-24] Frequency calculations were performed at the same theoretical level to verify that the ground state corresponds to a minimum point and the transition state corresponds to a saddle point, and to obtain the zero point energy (ZPE) and thermochemical correction values at 298.15 K and 1 atm pressure. All transition states were confirmed by intrinsic reaction coordinate (IRC) calculations to connect the reactants and products. Single point energy calculations were carried out at the ωB97XD/def2-TZVPP level in combination with the SMD solvation model (DME).[25] The Gibbs free energy was obtained by performing Grimme-type quasi-harmonic correction by using the GoodVibes program.[26,27] The geometric structures were illustrated by the CYLview 2.0 program.[28] Electronic structure analyses were performed with the Multiwfn 3.8 (dev) code.[29,30] The isosurface maps of spin density and molecular orbitals (MOs) were rendered by Visual Molecular Dynamics (VMD 1.9.3) software based on the files exported from Multiwfn.[31]

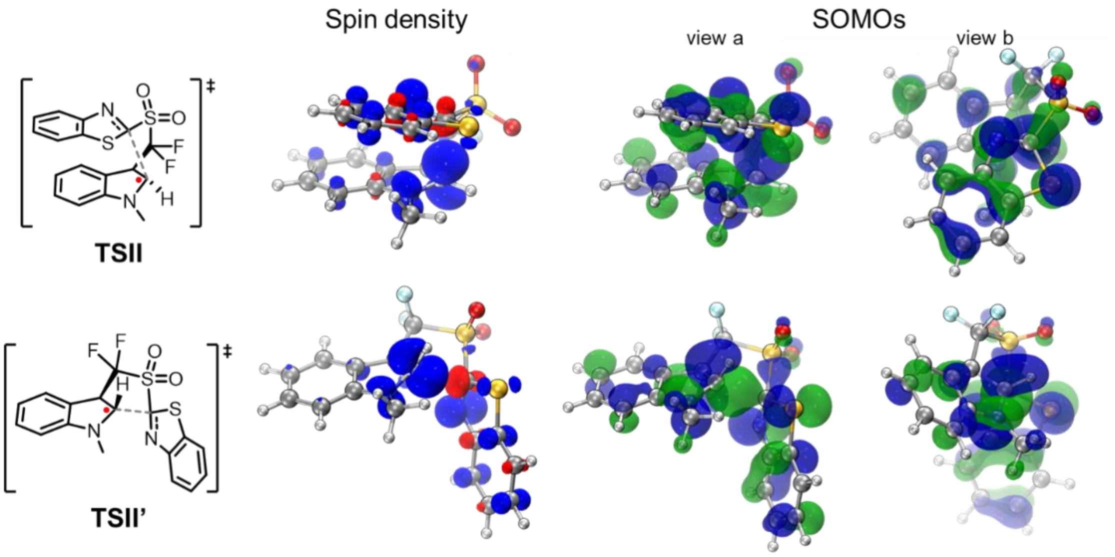

Figure S228. Spin density distribution diagrams (blue and red represent α and β spins respectively) and SOMO (highest singly occupied molecular orbital) diagrams (green and blue represent the positive and negative phases respectively) of **TSII** and **TSII'**.

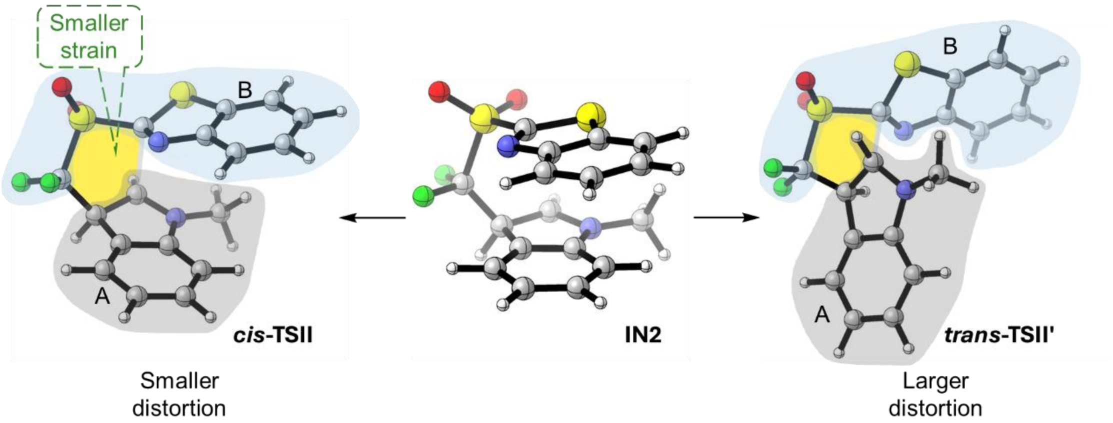

TS (kcal mol^{-1})	$\Delta E^{\neq}$	$\Delta E_{dist\text{-}A}$	$\Delta E_{dist\text{-}B}$	$\Delta E_{dist\text{-}tot}$	ΔE_{int}
**TSII**	3.1	0.1	4.1	4.2	-1.1
**TSII'**	21.0	8.2	8.5	16.7	4.3

Figure S229. The distortion/interaction analysis of **TSII** and **TSII'**.

Table S15. Energies.

Structure	E_SPC	E	ZPE	H_SPC	T.S	T.qh-S	G(T)_SPC	qh-G(T)_SPC
**IN1**	-1508.515322	-1507.349227	0.114678	-1508.387005	0.055788	0.053587	-1508.442793	-1508.440591
**TSI**	-1911.673085	-1910.069031	0.274223	-1911.376277	0.077836	0.072494	-1911.454113	-1911.448771
**IN2**	-1911.704087	-1910.110337	0.275685	-1911.406146	0.073267	0.070139	-1911.479413	-1911.476286
**TSII**	-1911.699211	-1910.103478	0.276630	-1911.401683	0.069906	0.067362	-1911.471589	-1911.469045
**TSII'**	-1911.670567	-1910.075100	0.276381	-1911.372984	0.071111	0.068372	-1911.444094	-1911.441356
**IN3**	-1911.721380	-1910.129869	0.278015	-1911.422251	0.070727	0.068031	-1911.492978	-1911.490283
**IN3'**	-1911.709709	-1910.118039	0.277910	-1911.410537	0.071000	0.068346	-1911.481537	-1911.478883
**TSIII**	-1911.709871	-1910.122194	0.276898	-1911.411933	0.070720	0.068000	-1911.482653	-1911.479933
**TSIII'**	-1911.706336	-1910.11693	0.27699	-1911.408365	0.070464	0.067855	-1911.478829	-1911.47622
**IN4**	-1363.059554	-1361.840383	0.267307	-1362.773635	0.066389	0.063453	-1362.840024	-1362.837088
**IN4'**	-1363.062109	-1361.842651	0.267257	-1362.776153	0.067113	0.063838	-1362.843267	-1362.839991
**3**	-1363.732371	-1362.510473	0.280613	-1363.433259	0.065056	0.062428	-1363.498315	-1363.495687
**3'**	-1363.735487	-1362.514097	0.280912	-1363.436042	0.065499	0.062691	-1363.501541	-1363.498733
**1**	-403.156595	-402.710584	0.159323	-402.988513	0.041356	0.041136	-403.029869	-403.029649
**SO_2**	-548.658922	-548.287691	0.007234	-548.647686	0.028843	0.028844	-548.676530	-548.676530
**DME**	-308.895738	-308.532308	0.142388	-308.744635	0.040874	0.040300	-308.785509	-308.784935
**$DME^{\cdot}$**	-308.234040	-307.871521	0.128552	-308.096454	0.042623	0.041615	-308.139078	-308.138069

E_SPC= single point energy; E= electronic energy; ZPE = zero point energy; H_SPC = enthalpy; G(T)_SPC = Gibbs free energy; qh-G(T)_SPC = Gibbs free energy with Grimme-type quasi-harmonic correction. Methods: *ω*B97XD/def2-TZVPP-SMD(DME)// *ω*B97XD/def2-SVP. All units are in a.u.

Cartesian Coordinates

19

**IN1**	Eopt	-1507.349227	
C	-2.661566	0.808694	0.806190
C	-0.353792	-0.217962	-0.363992
C	1.949168	-0.685236	0.128242
C	3.251105	-1.055750	0.428564
C	1.653728	0.622414	-0.333234
C	4.248880	-0.115533	0.267109
C	2.677608	1.555575	-0.490698
C	3.964937	1.174565	-0.186486
F	-2.286521	2.058437	0.558779
F	-2.116394	0.434998	1.967721
O	-2.561567	0.209004	-1.777446
S	-2.137287	-0.326245	-0.528432
O	-2.504367	-1.656562	-0.164802
S	0.481287	-1.624579	0.228944
N	0.331296	0.834059	-0.582064
H	3.470512	-2.051273	0.778784
H	5.269045	-0.382680	0.496150
H	2.439062	2.545455	-0.843176
H	4.771608	1.882215	-0.300177

38

**TSI**	Eopt	-1910.069031	
C	1.169331	1.567695	0.465165
C	1.686653	-0.968419	-1.860310
C	2.961209	0.773723	-0.410429
C	1.327805	-2.303550	-1.894272
C	2.465818	-0.527896	-0.801194
C	-1.530089	1.104326	-0.344575
C	3.737086	0.545425	0.732897
C	1.725359	-3.186887	-0.892297
C	2.868349	-1.431340	0.206661
C	-3.006368	-0.797885	-0.269252
C	-3.810635	-1.926040	-0.312298
C	2.500546	-2.768210	0.173018
C	-3.213973	0.206153	0.708170
C	-4.819698	-2.044715	0.622363
C	-4.239841	0.066260	1.641027
C	-5.031563	-1.059209	1.587343
F	0.669002	0.533355	1.189174
F	1.475775	2.537104	1.339575
O	0.292414	1.788717	-2.033876

S	-0.128214	2.151423	-0.708662
O	-0.407996	3.530060	-0.451689
S	-1.669390	-0.353645	-1.295535
N	-2.341863	1.250073	0.628176
N	3.633726	-0.743106	1.122616
C	4.204506	-1.316820	2.305072
H	1.358755	-0.273301	-2.616137
H	3.058968	1.615383	-1.070030
H	0.720590	-2.670718	-2.707462
H	1.418815	-4.220735	-0.947561
H	-3.646724	-2.685986	-1.059267
H	2.799227	-3.456788	0.948536
H	-5.457258	-2.915590	0.606054
H	-4.389644	0.837226	2.378928
H	-5.830813	-1.184235	2.301961
H	4.854227	-2.155463	2.044600
H	4.788059	-0.563745	2.830786
H	3.415371	-1.680097	2.967860
H	4.283744	1.260809	1.316578

38

IN2 Eopt -1910.110337

C	2.244920	-1.125806	0.844002
C	-0.159721	0.689488	2.107431
C	2.300875	0.344047	1.278698
C	-1.322026	1.427559	1.911541
C	0.929952	0.962652	1.314400
C	-0.295487	-1.056509	-0.638552
C	2.980651	1.202837	0.269147
C	-1.376822	2.418176	0.937368
C	0.880451	1.978803	0.337161
C	-2.516700	-0.159954	-0.925629
C	-3.747960	0.426400	-1.160048
C	-0.286278	2.712840	0.140601
C	-2.329481	-1.065956	0.147793
C	-4.795270	0.106293	-0.316647
C	-3.402539	-1.378454	0.982337
C	-4.622305	-0.786763	0.740692
F	3.481200	-1.650887	0.712660
F	1.594181	-1.896378	1.737142
O	1.628081	-2.766742	-1.187100
S	1.435453	-1.397300	-0.837105
O	1.942786	-0.390928	-1.728703

S	-1.015383	0.052736	-1.785720
N	-1.062546	-1.544028	0.264341
N	2.102355	2.083619	-0.271919
C	2.375210	2.877544	-1.432293
H	-0.120407	-0.090362	2.851572
H	2.800387	0.343784	2.260334
H	-2.192118	1.223970	2.514993
H	-2.295150	2.968610	0.798665
H	-3.879237	1.114933	-1.979166
H	-0.347112	3.479853	-0.614603
H	-5.763197	0.554965	-0.480025
H	-3.252133	-2.070320	1.794569
H	-5.462071	-1.015897	1.379163
H	1.858168	3.833808	-1.361283
H	3.446767	3.051196	-1.508966
H	2.040680	2.347392	-2.328177
H	3.775461	1.741513	0.741382

38

TSII Eopt -1910.103478

C	-2.630685	-0.058608	-0.175409
C	-1.471485	3.019422	-0.537333
C	-1.849106	0.791058	0.840635
C	-0.544899	4.030340	-0.821185
C	-1.094826	1.990259	0.308395
C	-0.132367	-1.295626	-0.173534
C	-0.743873	0.029490	1.548933
C	0.729657	4.005307	-0.254701
C	0.186211	1.982234	0.877050
C	2.287393	-1.549668	-0.098813
C	3.658680	-1.711598	0.098682
C	1.118833	2.978139	0.609218
C	1.776790	-0.504476	-0.911469
C	4.524079	-0.811357	-0.517232
C	2.668237	0.393202	-1.522731
C	4.030419	0.233127	-1.317157
F	-3.879241	-0.290254	0.260018
F	-2.715885	0.502757	-1.380077
O	-2.137696	-2.237612	-1.705280
S	-1.850633	-1.755824	-0.370449
O	-2.216000	-2.510486	0.821523
S	0.971305	-2.472586	0.588863
N	0.413113	-0.450842	-1.006959

N	0.329495	0.862555	1.694129
C	1.519938	0.581975	2.458985
H	-2.464835	3.038196	-0.987037
H	-2.608658	1.118703	1.575066
H	-0.823288	4.843524	-1.493611
H	1.439800	4.800896	-0.490019
H	4.045969	-2.518450	0.723551
H	2.119105	2.954715	1.043478
H	5.601023	-0.920002	-0.374418
H	2.265323	1.200997	-2.135061
H	4.731756	0.927482	-1.784441
H	1.380526	-0.354934	3.011155
H	2.396567	0.468342	1.800914
H	1.713886	1.396526	3.174455
H	-0.938464	-0.730481	2.307744

38

TSII' Eopt -1910.075100

C	1.726965	1.822142	-0.014365
C	4.031486	-0.700833	0.621573
C	1.623197	0.355890	0.261895
C	4.733819	-1.913404	0.567207
C	2.715220	-0.683172	0.201723
C	-1.155665	0.960809	0.183464
C	0.521120	-0.196118	-0.609320
C	4.101950	-3.076146	0.130873
C	2.082174	-1.861245	-0.237318
C	-3.156797	-0.310862	-0.391248
C	-4.353333	-0.917180	-0.777806
C	2.761076	-3.072217	-0.274398
C	-2.568799	-0.575212	0.872652
C	-4.962782	-1.793930	0.112917
C	-3.201848	-1.469670	1.754458
C	-4.390996	-2.066986	1.368251
F	2.507781	2.473498	0.848281
F	2.119074	2.115833	-1.254061
O	-0.427914	3.168390	-0.992277
S	-0.068217	2.461593	0.218283
O	-0.105890	3.051742	1.544407
S	-2.150189	0.793421	-1.290019
N	-1.403652	0.092969	1.131338
N	0.764330	-1.584736	-0.641744
C	-0.020758	-2.492071	-1.442404

H	4.513057	0.211167	0.979152
H	1.251791	0.307430	1.308619
H	5.780958	-1.946510	0.872699
H	0.243817	0.280260	-1.558167
H	4.662225	-4.012978	0.098589
H	-4.799165	-0.711664	-1.752527
H	2.277756	-3.987799	-0.618705
H	-5.898941	-2.279403	-0.169919
H	-2.741390	-1.672464	2.722484
H	-4.892105	-2.761041	2.045934
H	-0.982306	-2.017752	-1.676232
H	0.495206	-2.754802	-2.382211
H	-0.228595	-3.416115	-0.883044

38

IN3 Eopt -1910.129869

C	1.306519	1.847301	-0.600550
C	3.579634	-0.377799	-0.976691
C	1.885248	0.966337	0.488850
C	4.103105	-1.639964	-1.234483
C	2.618471	-0.258073	-0.001712
C	-0.539211	0.308063	0.620405
C	0.781194	0.338925	1.392458
C	3.662085	-2.742466	-0.521709
C	2.174129	-1.369218	0.724588
C	-2.850072	-0.567094	0.303593
C	-4.168188	-0.955052	0.174281
C	2.695836	-2.626649	0.466246
C	-1.878918	-0.899591	-0.690961
C	-4.538633	-1.674765	-0.948791
C	-2.283999	-1.635604	-1.813154
C	-3.601976	-2.007296	-1.930007
F	1.932097	3.033795	-0.718825
F	1.278963	1.304208	-1.825963
O	-1.404541	2.349333	-1.092655
S	-0.488062	2.110676	-0.026405
O	-0.434750	3.030357	1.070667
S	-2.121215	0.331438	1.599426
N	-0.635469	-0.490661	-0.449026
N	1.271004	-0.985028	1.693042
C	0.407177	-1.917212	2.365244
H	3.915005	0.482805	-1.534202
H	2.542588	1.600356	1.094944

H	4.855006	-1.759015	-1.998514
H	4.074912	-3.716004	-0.739959
H	-4.888954	-0.695713	0.933096
H	2.358733	-3.495653	1.007937
H	-5.566876	-1.979564	-1.067294
H	-1.549218	-1.877627	-2.562925
H	-3.923400	-2.564611	-2.796925
H	-0.109173	-1.401767	3.173779
H	-0.337610	-2.352985	1.688782
H	1.012114	-2.717211	2.788230
H	0.616923	0.914396	2.309961

38

IN3' Eopt -1910.118039

C	1.383839	1.903403	0.045456
C	4.245341	0.142624	0.531427
C	1.629310	0.497341	0.507634
C	5.250195	-0.822566	0.389071
C	2.929030	-0.240261	0.351166
C	-0.716820	-0.059554	0.178622
C	0.683873	-0.399037	-0.304521
C	4.913327	-2.140861	0.086961
C	2.587513	-1.574471	0.056954
C	-3.055160	-0.539535	-0.542151
C	-4.306992	-0.863834	-1.055395
C	3.582440	-2.537855	-0.086307
C	-2.781681	-0.637430	0.864464
C	-5.299240	-1.278927	-0.168644
C	-3.817488	-1.059562	1.740794
C	-5.054224	-1.374104	1.218252
F	1.807978	2.839603	0.892828
F	1.864215	2.168271	-1.168463
O	-0.935890	2.434363	-1.350058
S	-0.536261	1.960729	-0.039219
O	-1.009892	2.562409	1.195005
S	-1.655397	0.043930	-1.387344
N	-1.552570	-0.301932	1.256967
N	1.200346	-1.726707	-0.060970
C	0.643043	-2.828862	-0.806643
H	4.493589	1.178971	0.769646
H	1.326356	0.476445	1.572730
H	6.297263	-0.541021	0.511947
H	0.785380	-0.134942	-1.380718

H	5.705097	-2.884713	-0.026837
H	-4.509829	-0.796683	-2.125309
H	3.340883	-3.572519	-0.333449
H	-6.286044	-1.538565	-0.557134
H	-3.599447	-1.134422	2.806758
H	-5.854888	-1.708048	1.880729
H	-0.452709	-2.788834	-0.749115
H	0.938631	-2.812686	-1.873802
H	0.966501	-3.781146	-0.364116

38

TSIII Eopt -1910.122194

C	1.146502	1.859913	-0.581140
C	3.569767	-0.142493	-1.090265
C	1.805635	0.993014	0.465531
C	4.215145	-1.337358	-1.389229
C	2.640710	-0.140088	-0.077731
C	-0.535488	0.023721	0.697204
C	0.793233	0.240557	1.382335
C	3.922913	-2.490728	-0.679666
C	2.343882	-1.303238	0.643241
C	-2.851366	-0.716907	0.324794
C	-4.185218	-1.049464	0.178113
C	2.987834	-2.493712	0.344180
C	-1.912223	-0.971092	-0.710630
C	-4.588024	-1.633245	-1.008864
C	-2.342983	-1.566123	-1.896075
C	-3.675310	-1.886362	-2.033150
F	1.750264	3.048377	-0.756641
F	1.016463	1.308595	-1.795566
O	-1.547857	2.527320	-0.910847
S	-0.633776	2.140515	0.125528
O	-0.449948	3.025583	1.247794
S	-2.064329	0.022624	1.685766
N	-0.642886	-0.603979	-0.433233
N	1.440018	-1.032078	1.645948
C	0.713184	-2.057139	2.343465
H	3.789645	0.757061	-1.643853
H	2.414619	1.664701	1.081912
H	4.945903	-1.364548	-2.182031
H	4.429309	-3.410882	-0.930041
H	-4.888516	-0.850418	0.970743
H	2.765525	-3.401821	0.881093

H	-5.626532	-1.893314	-1.144605
H	-1.624772	-1.747971	-2.678325
H	-4.023132	-2.338228	-2.949588
H	0.179414	-1.606087	3.178972
H	-0.009113	-2.569781	1.696745
H	1.419793	-2.788161	2.732619
H	0.610367	0.780092	2.318275

38

TSIII’ Eopt -1910.116930

C	1.241910	1.922339	0.130422
C	4.016016	0.325065	1.046447
C	1.455865	0.494043	0.514727
C	5.123170	-0.515757	0.992183
C	2.825336	-0.137037	0.542756
C	-0.726442	-0.332937	-0.262730
C	0.741720	-0.384936	-0.538368
C	5.009273	-1.788379	0.461051
C	2.703643	-1.423118	-0.003905
C	-3.142199	-0.770329	-0.373298
C	-4.505024	-0.947057	-0.531453
C	3.804963	-2.260871	-0.042934
C	-2.554236	-0.686551	0.921784
C	-5.289687	-1.038004	0.602413
C	-3.373150	-0.787159	2.051204
C	-4.726219	-0.958451	1.878881
F	1.352536	2.820451	1.116048
F	1.975169	2.358679	-0.904507
O	-0.689942	2.278725	-1.829601
S	-0.645198	1.826005	-0.467274
O	-1.498925	2.476290	0.483299
S	-1.923083	-0.622176	-1.596094
N	-1.222437	-0.519382	0.934666
N	1.400253	-1.674388	-0.416429
C	1.107680	-2.661411	-1.423550
H	4.092820	1.317720	1.462519
H	0.962358	0.350960	1.486066
H	6.073593	-0.171670	1.368357
H	0.904224	0.033565	-1.552582
H	5.876897	-2.430268	0.429360
H	-4.937153	-1.007043	-1.517419
H	3.740788	-3.254434	-0.456675
H	-6.355695	-1.170263	0.499474

H	-2.923876	-0.718949	3.027945
H	-5.369433	-1.030194	2.742657
H	1.474040	-2.365471	-2.415054
H	1.566359	-3.605378	-1.139513
H	0.028718	-2.802456	-1.476163

35

IN4 Eopt -1361.840383

C	0.625811	1.874789	-0.047645
C	3.746548	1.251743	-0.045583
C	-3.956195	-0.761651	-1.367833
C	1.341017	1.000830	0.934772
C	4.821734	0.575942	-0.606242
C	2.643151	0.522869	0.336993
C	-2.850836	-0.560795	-0.558306
C	-5.158747	-0.206969	-0.977134
C	0.595792	-0.335717	1.225605
C	-0.812117	-0.302972	0.732221
C	4.764930	-0.796838	-0.792988
C	2.574510	-0.862715	0.139540
C	-2.944393	0.187770	0.636819
C	-5.261848	0.534095	0.199101
C	3.650829	-1.533071	-0.423686
C	-4.167139	0.737267	1.011228
F	0.554996	1.478112	-1.329618
F	0.961915	3.177093	-0.028435
S	-1.219506	-1.118090	-0.776087
N	1.362335	-1.367252	0.546478
N	-1.771619	0.294708	1.326427
C	1.199992	-2.730432	0.975686
H	1.469088	1.598467	1.839560
H	0.571622	-0.516869	2.312131
H	3.776994	2.321363	0.098214
H	-3.872397	-1.333204	-2.278855
H	5.703820	1.123528	-0.899099
H	-6.032942	-0.348435	-1.594497
H	5.607032	-1.306779	-1.236974
H	-6.216518	0.955875	0.475171
H	3.622369	-2.599534	-0.581662
H	-4.231872	1.310026	1.922191
H	0.139644	-2.939832	1.109210
H	1.586822	-3.398198	0.208635
H	1.722987	-2.930870	1.920223

35

IN4' Eopt -1361.842651

C	-1.997839	2.320535	-0.547773
C	-3.043513	-0.468949	-1.779385
C	3.995919	-1.602978	-0.540154
C	-1.277907	1.029117	-0.599612
C	-3.875469	-1.574431	-1.649349
C	-2.217731	-0.140694	-0.729909
C	2.897288	-0.845652	-0.171356
C	5.218314	-0.968791	-0.645642
C	-0.541165	0.691105	0.766341
C	0.867707	0.328798	0.469374
C	-3.871333	-2.319209	-0.481043
C	-2.202873	-0.896800	0.447075
C	3.016372	0.538393	0.092549
C	5.347033	0.395003	-0.387623
C	-3.040843	-1.994945	0.579230
C	4.258890	1.156065	-0.018562
F	-1.271861	3.445304	-0.682527
F	-2.869847	2.498039	0.464531
S	1.246245	-1.347096	0.047626
N	-1.256513	-0.428891	1.329520
N	1.848042	1.146631	0.445629
C	-1.368052	-0.531086	2.758509
H	-0.556557	1.090841	-1.418336
H	-0.546452	1.557600	1.439832
H	-3.050973	0.127557	-2.678764
H	3.893204	-2.658154	-0.739555
H	-4.533309	-1.847717	-2.459485
H	6.087937	-1.539885	-0.933528
H	-4.527677	-3.172043	-0.391792
H	6.316730	0.860396	-0.479774
H	-3.046817	-2.589581	1.479087
H	4.342802	2.211720	0.182777
H	-1.996026	0.265263	3.177112
H	-1.802842	-1.493175	3.017348
H	-0.374075	-0.466437	3.202970

36

3 Eopt -1362.510473

C	0.834897	1.821958	-0.355802
C	3.941851	1.089150	0.190845
C	-4.051582	-0.467700	-1.441650

C	1.399844	0.955101	0.769851
C	5.045641	0.369277	-0.252146
C	2.748011	0.425361	0.348922
C	-2.917214	-0.394243	-0.651817
C	-5.269421	-0.178621	-0.858164
C	0.603575	-0.357913	1.004085
C	-0.822917	-0.306667	0.583781
C	4.933247	-0.981438	-0.533422
C	2.630855	-0.939924	0.066085
C	-2.994284	-0.036843	0.712778
C	-5.357622	0.176239	0.486763
C	3.731111	-1.655716	-0.377415
C	-4.232457	0.249388	1.279472
F	1.641495	2.889466	-0.566983
F	-0.374772	2.344221	-0.034692
S	-1.261122	-0.695184	-1.085607
N	1.332284	-1.375847	0.256476
N	-1.792488	-0.015284	1.358127
C	1.045126	-2.751813	0.570396
H	1.431610	1.571629	1.671207
H	0.614478	-0.582490	2.084867
H	4.018972	2.142755	0.409258
H	-3.979750	-0.740383	-2.482918
H	5.994119	0.868107	-0.375835
H	-6.167369	-0.227122	-1.455380
H	5.799455	-1.527596	-0.876643
H	-6.324988	0.397549	0.911784
H	3.663349	-2.709466	-0.596656
H	-4.285401	0.524845	2.320248
H	1.445337	-3.046986	1.549484
H	-0.034242	-2.898662	0.574971
H	1.475814	-3.391368	-0.197126
H	0.735522	1.283276	-1.310071

36

3' Eopt -1362.514097

C	-1.541451	2.381344	0.187202
C	-3.541233	0.339203	-1.299852
C	4.276818	-1.222665	-0.972464
C	-1.224146	0.964298	-0.279908
C	-4.544374	-0.622040	-1.338607
C	-2.439307	0.102953	-0.511684
C	3.113057	-0.705926	-0.428467

C	5.439923	-0.490714	-0.837126
C	-0.455546	0.177614	0.816119
C	0.985298	0.042433	0.469148
C	-4.425027	-1.788368	-0.602708
C	-2.311888	-1.076745	0.228434
C	3.109261	0.534186	0.248915
C	5.446884	0.734131	-0.171865
C	-3.312766	-2.033940	0.188325
C	4.293181	1.255096	0.373316
F	-2.402412	2.372960	1.233233
F	-2.140189	3.098379	-0.792178
S	1.508921	-1.380335	-0.429814
N	-1.095429	-1.125972	0.883004
N	1.887338	0.906867	0.732818
C	-0.907836	-1.890048	2.088297
H	-0.626694	1.057524	-1.194163
H	-0.534024	0.709009	1.779546
H	-3.630901	1.254691	-1.863883
H	4.268029	-2.170678	-1.486990
H	-5.421653	-0.454082	-1.943744
H	6.358772	-0.874333	-1.253943
H	-5.214450	-2.524593	-0.640172
H	6.372987	1.281708	-0.083551
H	-3.237691	-2.947800	0.756209
H	4.284408	2.200935	0.890500
H	-1.253634	-2.908164	1.924600
H	0.155031	-1.921625	2.327476
H	-1.449559	-1.460107	2.941080
H	-0.638391	2.929678	0.501372

19

1 Eopt -402.710584

C	-1.777597	1.132703	0.000014
C	0.708329	1.872408	0.000014
C	-2.571144	0.008077	0.000014
C	-0.392068	0.973022	0.000014
C	1.838110	1.112003	0.000014
C	-2.014229	-1.274123	0.000014
C	0.158462	-0.333349	0.000014
C	-0.648928	-1.464330	0.000014
N	1.519949	-0.217553	0.000014
C	2.449121	-1.309188	0.000014
H	-2.212967	2.120910	0.000014

H	-3.646088	0.109668	0.000014
H	-2.670538	-2.132158	0.000014
H	-0.224427	-2.457816	0.000014
H	2.311999	-1.932892	0.887014
H	2.311025	-1.933917	-0.886986
H	3.465494	-0.919113	-0.000986
H	0.658969	2.943497	0.000014
H	2.866548	1.421359	0.000014

3

SO_2 Eopt -548.287691

O	1.274885	-0.337794	-0.000000
S	0.000000	0.347158	0.000000
O	-1.274885	-0.356522	-0.000000

16

DME Eopt -308.532308

C	-2.870541	-0.317639	-0.095736
O	-1.599266	-0.187389	0.484313
C	-0.685314	0.494614	-0.343217
C	0.685364	0.501285	0.333614
O	1.598491	-0.199020	-0.479420
C	2.871031	-0.314923	0.101120
H	-2.824724	-0.886477	-1.032794
H	-3.487938	-0.851406	0.623750
H	-3.316175	0.663808	-0.303209
H	-0.585754	-0.017433	-1.311172
H	-1.042563	1.518847	-0.528624
H	1.043368	1.528996	0.496816
H	0.585708	0.010262	1.312429
H	2.827165	-0.860412	1.051938
H	3.486868	-0.866300	-0.606211
H	3.317013	0.671357	0.283248

15

DME$^{\bullet}$ Eopt -307.871521

C	-2.322438	0.825407	0.056884
O	-1.669219	-0.235649	-0.585901
C	-0.724383	-0.932843	0.234525
C	0.506194	-0.176509	0.553104
O	1.570495	-0.345699	-0.286123
C	2.652605	0.524986	-0.095673
H	-1.638353	1.648580	0.293813

H	-3.081584	1.183189	-0.636753
H	-2.805627	0.490994	0.984485
H	-1.231659	-1.236404	1.164357
H	-0.458235	-1.814863	-0.350327
H	0.498917	0.690056	1.195471
H	3.078028	0.415535	0.908683
H	3.403011	0.257767	-0.837742
H	2.353426	1.569684	-0.238835

Reference

1. Jacob, N.; Zaid, Y.; Oliveira, J. C. A.; Ackermann, L.; Wencel-Delord, J. Cobalt-Catalyzed Enantioselective C-H Arylation of Indoles. *J. Am. Chem. Soc.* **2022**, *144*, 798-806.
2. Xie, W.; Ning, S.; Liu, N.; Bai, Y.; Wang, S.; Wang, S.; Shi, L.; Che, X.; Xiang, J. Electrochemical Regioselective Bromination of Electron-Rich Aromatic Ring Using $^nBu_4NBr$. *Synlett* **2019**, *30*, 1313-1316.
3. Yadav. U.; Sakla, A. P.; Tokala, R.; Nyalam, S. T.; Khurana, A.; Digwal, C. S.; Talla, V.; Godugu, C.; Shankaraiah, N.; Kamal, A. Design and Synthesis of 5-Morpholino-Thiophene-Indole/Oxindole Hybrids as Cytotoxic Agents. *ChemistrySelect* **2020**, *5*, 4356-4363.
4. Long, Y.; Zheng, Y.; Xia, Y.; Qu, L.; Yang, Y.; Xiang, H.; Zhou, X. Nickel-Catalyzed Synthesis of an Aryl Nitrile via Aryl Exchange between an Aromatic Amide and a Simple Nitrile. *ACS Catal.* **2022**, *12*, 4688-4695.
5. Liu, B.; Liu, M.; Li, Q.; Li, Y.; Feng, K.; Zhou, Y. The Palladium-Catalyzed Direct C3-Cyanation of Indoles Using Acetonitrile as the Cyanide Source. *Org. Biomol. Chem.* **2022**, *18*, 6108-6114.
6. Des, A.; Watanabe, K.; Morimoto, H.; Ohshima, T. Boronic Acid Accelerated Three-Component Reaction for the Synthesis of α-Sulfanyl-Substituted Indole-3-acetic Acids. *Org. Lett.* **2017**, *19*, 5794-5797.
7. Yang, Y.; Gao, P.; Zhao, Y.; Shi, Z. Regiocontrolled Direct C-H Arylation of Indoles at the C4 and C5 Positions. *Angew. Chem. Int. Ed.* **2017**, *56*, 3966-3971.
8. Perrotta, D.; Wang, M.-M.; Waser, J. Lewis Acid Catalyzed Enantioselective Desymmetrization of Donor-Acceptor *meso*-Diaminocyclopropanes. *Angew. Chem. Int. Ed.* **2018**, *55*, 5120-5123.
9. Turnu, F.; Luridiana, A.; Cocco, A.; Porcu, S.; Frongia, A.; Sarais, G.; Secci, F. Catalytic Tandem Friedel-Crafts Alkylation/C4-C3 Ring-Contraction Reaction: An Efficient Route for the Synthesis of Indolyl Cyclopropanecarbaldehydes and Ketones. *Org. Lett.* **2019**, *21*, 7329-7332.
10. Echeverry-Gonzalez, C. A.; Villamizar, M. C. O.; Kouznetsov, V.; The Remarkable Selectivity of the 2-Arylquinoline-based Acyl Hydrazones toward Copper Salts: Exploration of their Catalytic Applizations in the Copper Catalyzed N-Arylation of Indole Derivatives and C1-Alkynylation of Tetrahydroisoquinolines via the A^3 Reaction. *New J. Chem.* **2021**, *45*, 243-250.
11. Laha, J. K.; Bhimpuria, R. A.; Prajapati, D. V.; Dayal, N.; Sharma, S. Palladium-Catalyzed Regioselective C-2 Arylation of 7-Azaindoles, Indoles, and Pyrroles with Arenes. *Chem. Commun.* **2016**, *52*, 4329-4332.
12. Yu, J.; Zhang, X.; Wu, X.; Liu, T.; Zhang, Z.-Q.; Wu, J.; Zhu, C. Metal-Free Radical Difunctionaization of Ethylene. *Chem* **2023**, *9*, 472-482.
13. Cismesia, M. A.; Yoon, T. P. Characterizing chain processes in visible light photoredox catalysis. *Chem. Sci.* **2015**, *6*, 5426-5434.
14. Quach, L.; Dutta, S.; Pflüger, P. M.; Sandfort, F.; Belotti, P.; Glorius, F. Visible-Light-Initiated Hydrooxygenation of Unactivated Alkenes--a Strategy for Anti-Markovnikov Hydrofunctionalization. *ACS Catal*. **2022**, *12*, 2499-2504.
15. Pozdnyakov, I. P.; Kel, O. V.; Plyusnin, V. F.; Grivin, V. P.; Bazhin, N. M. New Insight into Photochemistry of Ferrioxalate. *J. Phys. Chem. A*, **2008**, *112*, 8316-8322.

16. Dewanji, A.; Dalsen, L.; Rossi-Ashton, J. A.; Gasson, E.; Crisenza, G. E. M.; Procter, D. J. A General Arene C-H Functionalization Strategy via Electron Donor-Acceptor Complex Photoactivation. *Nat. Chem.* **2023**, *15,* 43-52.
17. Frisch, M. J.; Trucks, G. W.; Schlegel, H. B.; Scuseria, G. E.; Robb, M. A.; Cheeseman, J. R.; Scalmani, G.; Barone, V.; Petersson, G. A.; Nakatsuji, H.; Li, X.; Caricato, M.; Marenich, A. V.; Bloino, J.; Janesko, B. G.; Gomperts, R.; Mennucci, B.; Hratchian, H. P.; Ortiz, J. V.; Izmaylov, A. F.; Sonnenberg, J. L.; Williams-Young, D.; Ding, F.; Lipparini, F.; Egidi, F.; Goings, J.; Peng, B.; Petrone, A.; Henderson, T.; Ranasinghe, D.; Zakrzewski, V. G.; Gao, J.; Rega, N.; Zheng, G.; Liang, W.; Hada, M.; Ehara, M.; Toyota, K.; Fukuda, R.; Hasegawa, J.; Ishida, M.; Nakajima, T.; Honda, Y.; Kitao, O.; Nakai, H.; Vreven, T.; Throssell, K.; Montgomery, J. A., Jr.; Peralta, J. E.; Ogliaro, F.; Bearpark, M. J.; Heyd, J. J.; Brothers, E. N.; Kudin, K. N.; Staroverov, V. N.; Keith, T. A.; Kobayashi, R.; Normand, J.; Raghavachari, K.; Rendell, A. P.; Burant, J. C.; Iyengar, S. S.; Tomasi, J.; Cossi, M.; Millam, J. M.; Klene, M.; Adamo, C.; Cammi, R.; Ochterski, J. W.; Martin, R. L.; Morokuma, K.; Farkas, O.; Foresman, J. B.; Fox, D. J. *Gaussian 16, Revision A.03.* Gaussian, Inc., Wallingford CT **2016**.
18. Bannwarth, C.; Caldeweyher, E.; Ehlert, S.; Hansen, A.; Pracht, P.; Seibert, J.; Spicher, S.; Grimme, S. Extended Tight-Binding Quantum Chemistry Methods. *WIREs Comput. Mol. Sci.* **2021**, *11*, e1493.
19. Pracht, P.; Bohle, F.; Grimme, S. Automated Exploration of the Low-Energy Chemical Space with Fast Quantum Chemical Methods. *Phys. Chem. Chem. Phys.* **2020**, *22*, 7169–7192.
20. Pracht, P.; Grimme, S.; Bannwarth, C.; Bohle, F.; Ehlert, S.; Feldmann, G.; Gorges, J.; Müller, M.; Neudecker, T.; Plett, C.; Spicher, S.; Steinbach, P.; Wesołowski, P. A.; Zeller, F. CREST—A Program for the Exploration of Low-Energy Molecular Chemical Space. *J. Chem. Phys.* **2024**, *160*, 114110.
21. Bannwarth, C.; Ehlert, S.; Grimme, S. GFN2-xTB—An Accurate and Broadly Parametrized Self-Consistent Tight-Binding Quantum Chemical Method with Multipole Electrostatics and Density-Dependent Dispersion Contributions. *J. Chem. Theory Comput.* **2019**, *15*, 1652–1671.
22. Chai, J.-D.; Head-Gordon, M. Long-Range Corrected Hybrid Density Functionals with Damped Atom–Atom Dispersion Corrections. *Phys. Chem. Chem. Phys.* **2008**, *10*, 6615–6620.
23. Weigend, F.; Ahlrichs, R. Balanced Basis Sets of Split Valence, Triple Zeta Valence and Quadruple Zeta Valence Quality for H to Rn: Design and Assessment of Accuracy. *Phys. Chem. Chem. Phys.* **2005**, *7*, 3297–3305.
24. Weigend, F. Accurate Coulomb-Fitting Basis Sets for H to Rn. *Phys. Chem. Chem. Phys.* **2006**, *8*, 1057–1065.
25. Marenich, A. V.; Cramer, C. J.; Truhlar, D. G. Universal Solvation Model Based on Solute Electron Density and on a Continuum Model of the Solvent Defined by the Bulk Dielectric Constant and Atomic Surface Tensions. *J. Phys. Chem. B* **2009**, *113*, 6378–6396.

26. Luchini, G.; Alegre-Requena, J. V.; Funes-Ardoiz, I.; Paton, R. S. GoodVibes: Automated Thermochemistry for Heterogeneous Computational Chemistry Data. *F1000Res* **2020**, *9*, 291.
27. Grimme, S. Supramolecular Binding Thermodynamics by Dispersion-Corrected Density Functional Theory. *Chem. Eur. J.* **2012**, *18*, 9955–9964.
28. Legault, C. Y. *CYLview 20.* Université de Sherbrooke, **2020** (http://www.cylview.org).
29. Lu, T.; Chen, F. Multiwfn: A Multifunctional Wavefunction Analyzer. *J. Comput. Chem.* **2012**, *33*, 580–592.
30. Lu, T. A Comprehensive Electron Wavefunction Analysis Toolbox for Chemists, Multiwfn. *J. Chem. Phys.* **2024**, *161*, 082503.
31. Humphrey, W.; Dalke, A.; Schulten, K. VMD: Visual Molecular Dynamics. *J. Mol. Graph.* **1996**, *14*, 33–38.
